# Supplementary material for: Late-stage synthesis of heterobifunctional molecules for PROTAC applications via ruthenium-catalysed C‒H amidation
Source: Nat Commun. 2023 Dec 12;14:8222. doi: 10.1038/s41467-023-43789-9 (PMC10716378; doi:10.1038/s41467-023-43789-9)
Supplement: Supplementary file 1 — Supplementary Information [file 41467_2023_43789_MOESM1_ESM.pdf]

Supplementary Information

Late-stage synthesis of heterobifunctional molecules for PROTAC applications via ruthenium-catalysed C–H amidation

Daniele Antermite[1], Stig D. Friis[1], Johan R. Johansson[1], Okky Dwichandra Putra,[2] Lutz Ackermann[3,4]* and Magnus J. Johansson[1]*

magnus.j.johansson2@astrazeneca.com; lutz.ackermann@chemie.uni-goettingen.de

[1] Medicinal Chemistry, Research and Early Development, Cardiovascular, Renal and Metabolism (CVRM), BioPharmaceuticals R&D, AstraZeneca, Gothenburg, Sweden.
[2] Early Product Development and Manufacturing, Pharmaceutical Sciences R&D, AstraZeneca, Gothenburg, Sweden.
[3] Institut für Organische und Biomolekulare Chemie, Georg-August-Universität Göttingen, Göttingen, Germany.
[4] German Center for Cardiovascular Research (DZHK), Berlin, Germany.

Table of Contents

Supplementary Methods 2
1. General Information..... 2
2. Additional structures in SI 4
3. Unsuccessful dioxazolones 6
4. Reaction optimisation through High Throughput Experimentation (HTE) 7
4.1 Catalyst, solvent and additive(s) 8
4.2 Solvent and RCO_2H additive 9
4.3 Solvent and Ag replacement 10
4.4 Ag salt and PivOH equivalent 11
4.5 Characterisation of products **5, 6, 15** and **16** 12
5. LSF informer library 14
5.1 Type I inherent directing groups – Plate α 15
5.2 Type II inherent directing groups – Plate β 17
6. Single crystal X-ray diffraction and structure refinements for compound **8r** 19
7. Analysis of accessible exit vectors 21
8. Proposed de novo syntheses 24
9. Experimental details and characterisation data 27
9.1 Dioxazolone reagents (**4**, **9a-9j**, **13a-13e**, **17-26**) 27
9.2 General experimental conditions for late-stage C–H amidation 40
9.3 Late-stage functionalisation (**8a-8y**, **27-28**) 41
9.4 Late-stage linker installation (**10a-10k**) and derivatisation (**11**, **12**) 63
9.5 Single-step conjugation (**14a-14g**) 73
10. ^{1}H and ^{13}C NMR spectra for novel compounds 80
Supplementary References 165

Supplementary Methods

1. General Information

General reagent information. Anhydrous solvents were purchased from Sigma Aldrich, sparging with N_2 prior to use. Unless otherwise noted, all commercially available reagents were used as received. [Ru(*p*-cymene)Cl_2]$_2$, $AgSbF_6$, $AgPF_6$, PivOH and $(PhO)_2PO_2H$ were purchased from Strem Chemical Inc. or Sigma Aldrich. Reactions in sealed tubes were run in Biotage microwave vials (2–5 mL) with aluminium caps equipped with septa. Solids were either weighed by hand or using a Mettler Toledo Quantos system for automated solid dispensing. The dioxazolone reagents were typically stored at –20 °C under N_2 atmosphere; however, no decrease in purity was observed storing dioxazolone **4** at room temperature under air for > 3 months.

General purification information. Flash column chromatography purification was performed on Biotage Selekt automated system with pre-packed silica gel columns (5–10 g SiO_2 Sfär HC Duo). Unless otherwise stated, purification by preparative reverse phase HPLC was performed on a Kromasil C8 column (10 μm, 250x50 ID mm) with a flow rate of 100 mL/min over 25 or 30 minutes, using acidic mobile phase (A: H_2O/MeCN/HCO_2H 95/5/0.2, B: MeCN), or on a XBridge C18 column (10 μm, 250x50 ID mm) with a flow rate of 100 mL/min over 25 or 30 minutes, using basic mobile phase (A: H_2O/MeCN 95/5, 10 mM NH_4HCO_3, B: MeCN). UV detector Gilson UV/VIS-155 was used for UV-triggered collection of fractions at 240 or 254 nm wavelengths. Purification by supercritical fluid chromatography SFC was performed on a BEH column (5 μm, 250x30 ID mm) using basic mobile phase (A: CO_2, B: MeOH/H_2O/NH_3 95/5/0.2). Collection of fractions was performed at 254 nm wavelength.

General analytical information. Analytical thin-layer chromatography (TLC) was performed on precoated glass-backed silica gel plates. Visualisation of the developed chromatogram was performed by UV absorbance (254 nm) and stained with aqueous potassium permanganate solution, phosphomolybdic acid solution, or ninhydrin solution in ethanol. Analytical LC-MS was performed on a Waters Acquity UPLC system with a HSS C18 column (1.8 µm, 50 × 2.1 mm) using an acidic mobile phase at pH 3 (A: H_2O, 10 mM HCO_2H, 1 mM NH_3; B: MeCN/H_2O 95/5), or with a BEH C18 column (1.7 µm, 50 × 2.1 mm) using a basic mobile phase at pH 10 (A: H_2O, 5 mM NH_4HCO_3, 50 mM NH_3; B: MeCN/H_2O 95/5). For SFC-MS analysis a Waters Acquity UPC2 SFC-MS system with a BEH column was used (A: CO_2, B: MeOH/H_2O/NH_3 97/3/0.5). All new compounds were characterised by NMR spectroscopy and high-resolution mass spectrometry (HRMS). Nuclear magnetic resonance spectra (^{1}H, ^{13}C, ^{19}F, ^{11}B, COSY, HSQC, HMBC, NOESY and ROESY) were recorded on Bruker Ultrashield 500 or 600 MHz spectrometers with a Bruker Cryo Platform. NMR data is reported as follows: chemical shift (multiplicity [s = singlet, d = doublet, t = triplet, q = quartet, p = pentet, h = heptet, m = multiplet and br = broad]), coupling constant [in Hz] and integration. Chemical shifts for ^{1}H NMR spectra are reported in parts per million (ppm) with the residual solvent resonance as internal reference ($CDCl_3$: δ = 7.26 ppm, DMSO-d_6: δ = 2.50 ppm, CD_3OD: δ = 3.31 ppm, CD_3CN: δ = 1.94 ppm). ^{13}C NMR spectra were recorded with complete proton decoupling. Chemical shifts are reported in parts per million (ppm) with the solvent resonance as the internal reference ($^{13}CDCl_3$: δ = 77.0 ppm, $(^{13}CD_3)_2SO$: δ = 39.5 ppm, $^{13}CD_3OD$: δ = 49.0 ppm and $^{13}CD_3CN$: δ = 1.32 ppm). ^{19}F spectra were recorded with complete proton decoupling. HRMS data was recorded on a Waters Acquity UPLC System equipped with Acquity PDA and XEVO-QTOF mass spectrometer using electronspray ionisation (ESI) in positive or negative mode. A linear gradient 5-99% (A: H_2O, 10 mM HCO_2H, 1 mM HCO_2NH_4, B: MeCN/H_2O 95/5, 10 mM HCO_2H, 1 mM HCO_2NH_4) was run with a flow of 1 mL/min for 2.7 min at 45 °C on a Waters Acquity CSH C8 column (1.7 μm, 50x2.1 ID mm). Relative absorbance was recorded at 230 nm.

[Note: *For some low-MW dioxazolones it was not possible to detect any identifiable HRMS peak*.]

General safety considerations. Stoichiometric CO_2 gas is released as side-product during the reaction. Appropriate safety measure should be taken to mitigate pressure build-up. Keeping a 1:3 reaction volume to headspace ratio is recommended.

General software information. NMR data were collected using TopSpin v3 and IconNMR v5, and analysed using MesReNova v14. UPLC-MS data were collected and analysed using MassLynx v4. TIBCO Spotfire v11 was used for data visualisation and heat map generation. Collection and refinement of X-ray diffraction data was performed with CrysAlisPro 1.171.42.35a, Olex2.solve, Olex2 and ShelXL. Mercury v4 and MOE 2022.02 were used to visualise X-ray structures, and model protein surfaces.

2. Additional structures in SI

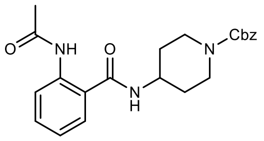

15

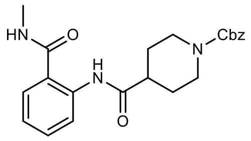

16

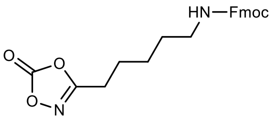

9a

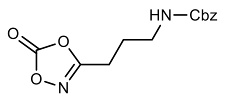

9b

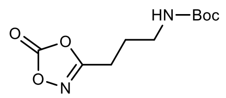

9c

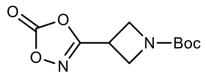

9d

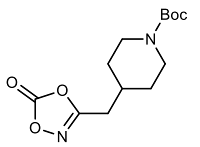

9e

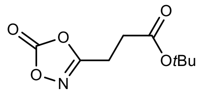

9f

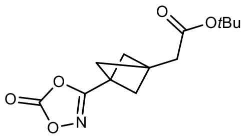

9g

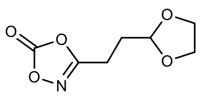

9h

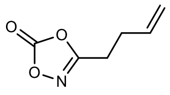

9i

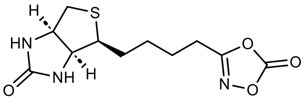

17

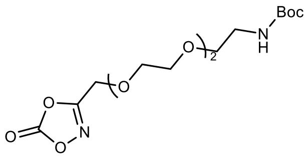

18

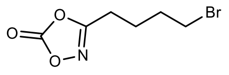

19

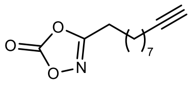

20

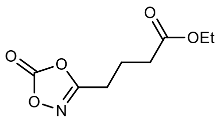

9j

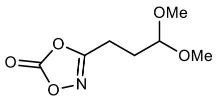

21

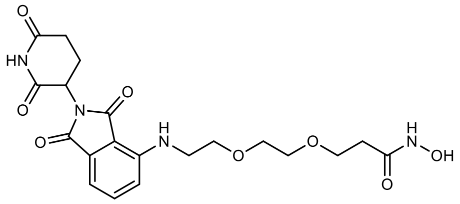

22

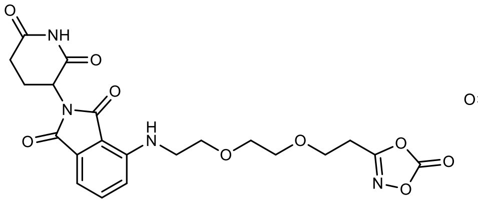

13a

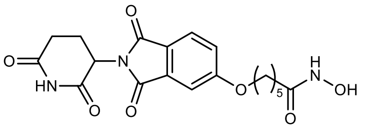

23

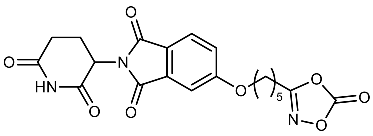

13b

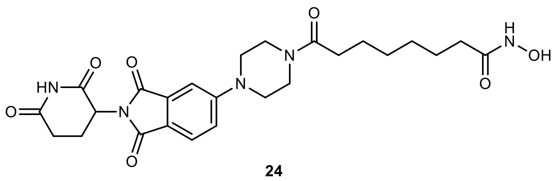

24

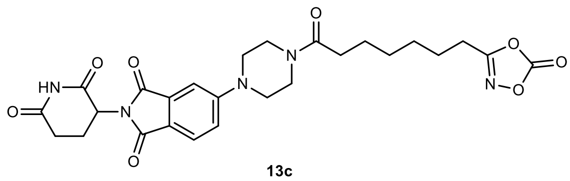

13c

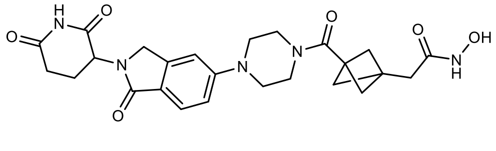

25

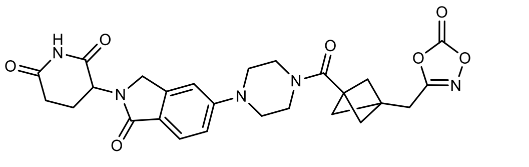

13d

Supplementary Figure 1. Additional structures in the Supplementary Information.

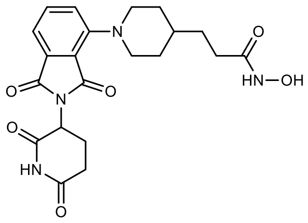

26

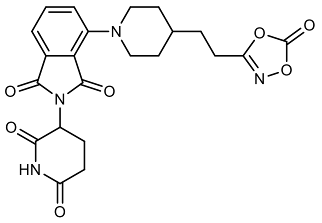

13e

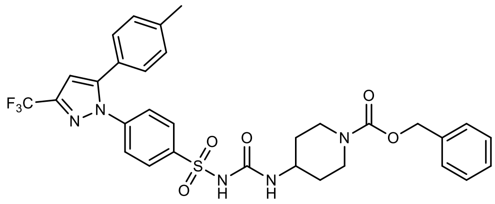

27

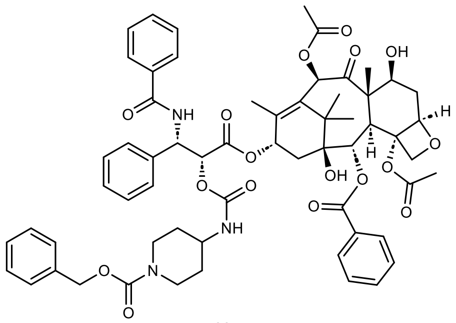

28

Supplementary Figure 1 (continue). Additional structures in the Supplementary Information.

3. Unsuccessful dioxazolones

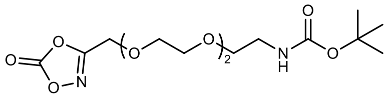

18

[no reaction]

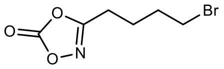

19

[no reaction]

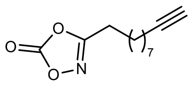

20

[no reaction]

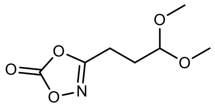

21

[acetal deprotection observed
under the reaction conditions]

Supplementary Figure 2. Dioxazolones unsuccessful in the ruthenium catalysed C–H amidation.

4. Reaction optimisation through High Throughput Experimentation (HTE)

Selected optimisation data for the ruthenium-catalysed C–H amidation is shown below in Supplementary Figures 1-4. The reactions were analysed by LCMS and the data shown is based on the relative intensities of the product peak(s) *vs.* substrate peak in the UV chromatogram; *i.e.* total conversion to product (mono + bis-functionalisation). For each screen, visualisation of the levels of mono-functionalisation is also provided, based on the relative intensity of the mono-functionalised product peak *vs.* bis-functionalisation peak in the UV chromatogram.

General procedure for reaction optimisation. The reactions were set-up using 96- or 24-wells Para-dox plates with 1 mL glass vials equipped with stirrer bars on a 0.025 mmol substrate scale. In a glovebox under nitrogen, the vials were charged with all the solid substrates, additives and catalysts using a Mettler Toledo Quantos system for automated solid dispensing. Dioxazolone **4** and any liquid reagent were then added as stock solutions in the required solvents for a total volume of 250 µl (0.1 M). The plate was sealed with a teflon film, taken out of the glovebox and heated under stirring (600 rpm). After 15-18 hours the reaction mixtures were allowed to cool down, diluted with DMSO (500 µl) and stirred at room temperature for 5 min. A 50 µl aliquot was extracted from each vial, transferred to a 96-well Greiner_V plastic plate and diluted with further DMSO (50 µl). The solids were centrifuged with Eppendorf Centrifuge 5810 R (room temperature, atmospheric pressure, 3000 rpm, 15 min). A 25 µl aliquote was extracted from each well and transferred to a fresh 96-well Greiner_V plastic plate. The wells were finally diluted with further DMSO (75 µl) and analysed by LCMS [Waters Acquity UPLC system, BEH C18 column (A: H_2O/MeCN/NH_3 = 95/5/0.2, B: MeCN)].

General purification procedure for optimisation substrates. To confirm the product(s) identity throughout the optimisation process, 3-5 successful reaction mixtures (> 50% conversion by LCMS) from relevant optimisation plates were combined after DMSO dilution, filtered and purified by preparative reverse phase HPLC. The relevant fractions were collected, combined and lyophilised to afford the desired product.

4.1 Catalyst, solvent and additive(s)

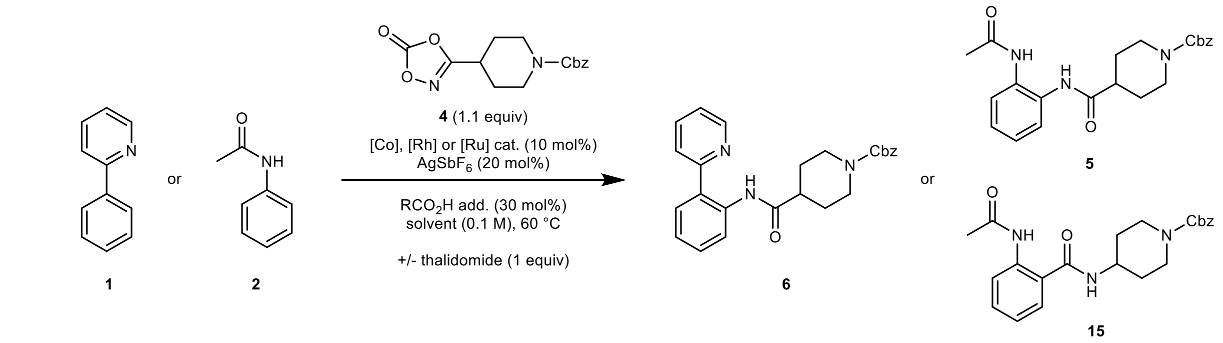

Substrate		Catalyst		Solvent		Additive(s)	
**A–D**	1	**1–4**	Cp*Co(MeCN)$_3$(SbF$_6$)$_2$ No AgSbF$_6$	**1,5,9**	DME	**A,E**	none
**E–H**	2	**5–8**	[Ru(*p*-cymene)Cl$_2$]$_2$ [a]	**2,6,10**	EtOAc	**B,F**	Thalidomide
		**9–12**	[Cp*RhCl$_2$]$_2$ [a]	**3,7,11**	DCE	**C,G**	Thalidomide + AdCO$_2$H
			[a] 5 mol% pre-catalyst.	**4,8,12**	TFE	**D,H**	Thalidomide + MesCO$_2$H

Results

Heat maps visualising conversion (%) and mono-functionalisation are shown below. In the ruthenium-catalysed reaction of substrate **2**, competitive formation of rearranged product **15** was observed in the absence of carboxylate additives (wells **E5–7**, **F5–7**). The ratio of product **5** *vs.* product **15** is shown on the relevant well(s).

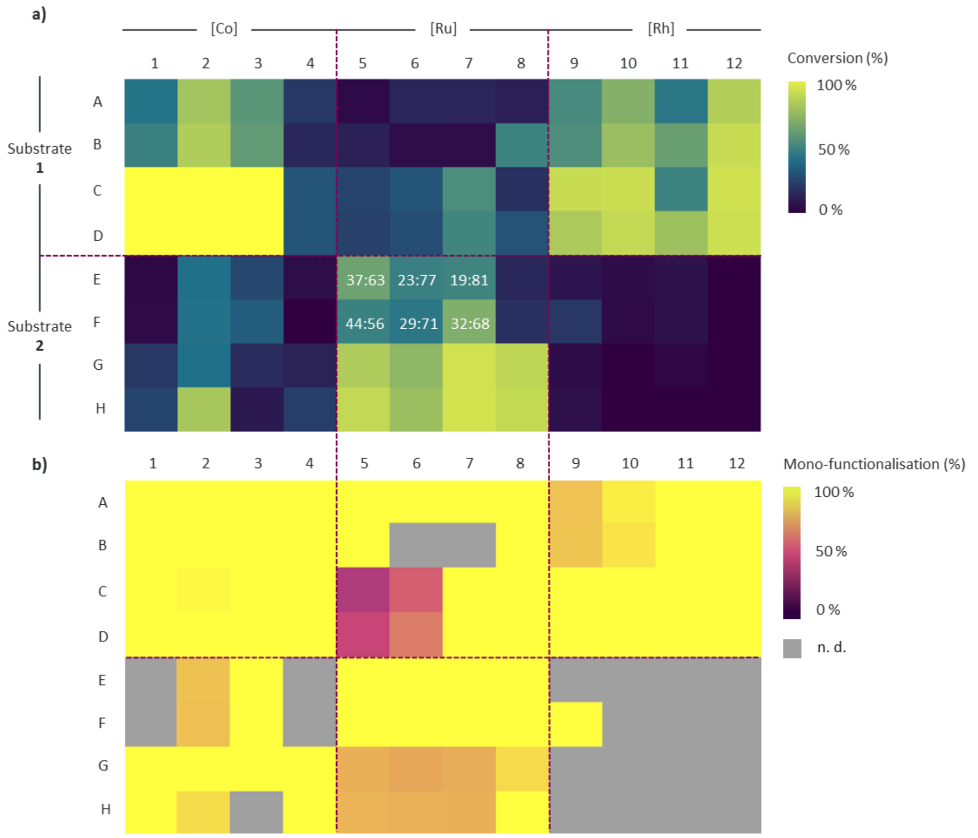

Supplementary Figure 3. Evaluation of cobalt, ruthenium and rhodium catalytic systems against a selection of solvents and acidic additives. **a)** Heat map for conversion (%): determined by LCMS, based on the relative intensities of the product peak(s) vs substrate peak in the UV chromatogram. **b)** Heat map for mono-functionalisation (%): determined by LCMS, based on the relative intensity of the mono-functionalised product peak vs bis-functionalisation peak in the UV chromatogram. n. d. = Not determined due to low conversion (< 10%).

4.2 Solvent and RCO_2H additive

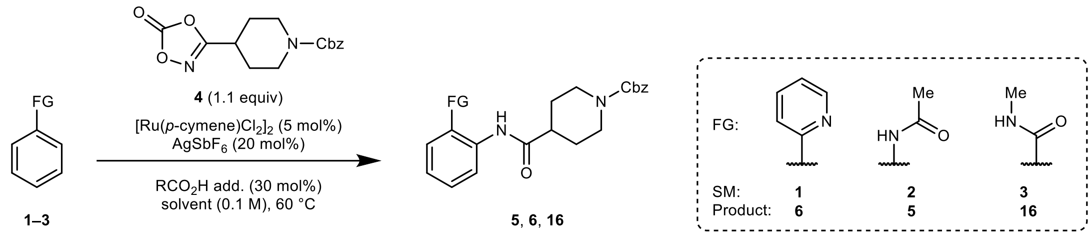

Substrate		Additive		Solvent	
**1–4**	1	**1,5,9**	PivOH	**A**	DME
**5–8**	2	**2,6,10**	$MesCO_2H$	**B**	EtOAc
**9–12**	3	**3,7,11**	Ac-Gly-OH	**C**	DCE
		**4,8,12**	$(PhO)_2PO_2H$	**D**	TFE
				**E**	HFIP
				**F**	TFE

Results

Heat maps visualising conversion (%) and mono-functionalisation are shown below. In the absence of $(PhO)_2PO_2H$ additive, a by-product consistent with the homocoupling of 2-phenylpyridine **1** was observed by LCMS (1.17 min, m/z: 308.13) in most of the solvent screened (wells A1–3, B1–3, C1–3, F1–3).[1]

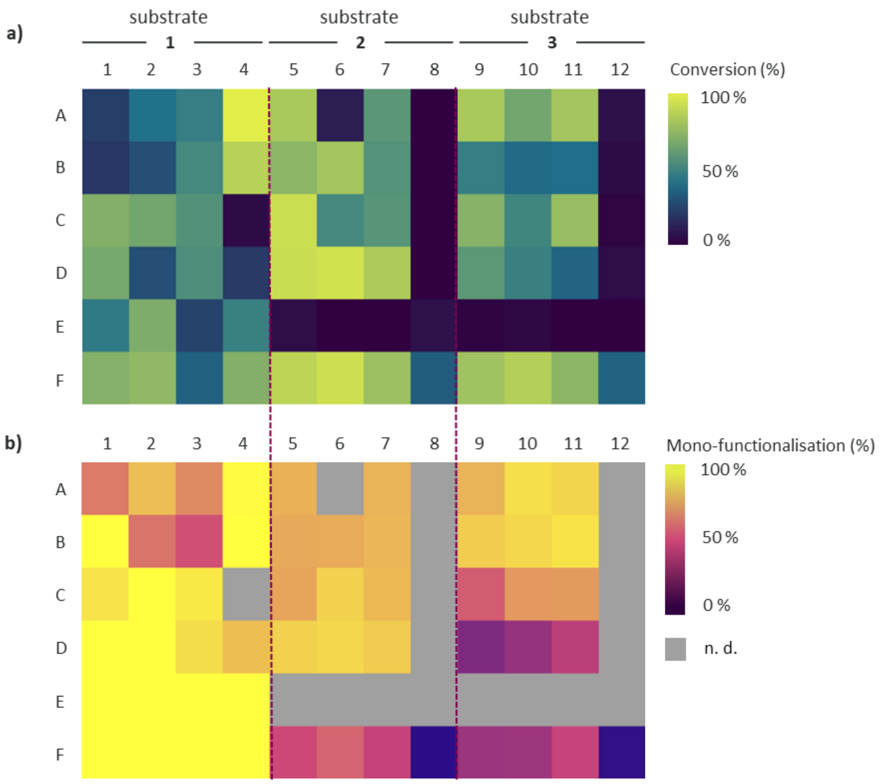

Supplementary Figure 4. Evaluation of solvents and acidic additives. **a)** Heat map for conversion (%): determined by LCMS, based on the relative intensities of the product peak(s) vs substrate peak in the UV chromatogram. **b)** Heat map for mono-functionalisation (%): determined by LCMS, based on the relative intensity of the mono-functionalised product peak vs bis-functionalisation peak in the UV chromatogram. n. d. = Not determined due to low conversion (< 10%).

4.3 Solvent and Ag replacement

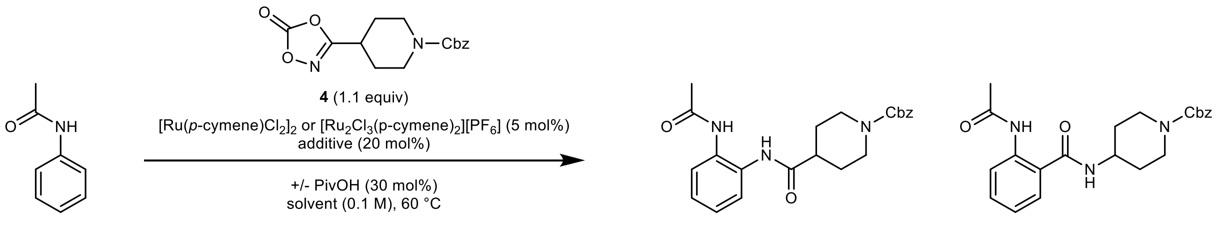

2 5 15

Additive		PivOH		Catalyst		Solvent	
**1,2**	$AgPF_6$	**1,3,5,7,9,11**	no	**1–6, 9–12**	[Ru(*p*-cymene)Cl_2]$_2$	**A**	2-MeTHF
**3,4**	KPF_6	**2,4,6,8,10,12**	yes	**7,8**	[Ru_2Cl_3(*p*-cymene) $_2$][PF_6]	**B**	DME
**5,6**	$NaPF_6$					**C**	EtOAc
**7,8**	none					**D**	DCE
**9,10**	$AgSbF_6$					**E**	TFE
**11,12**	$KSbF_6$					**F**	H_2O
						**G**	*t*-amylOH
						**H**	TFT

Results

Heat maps visualising conversion (%) and mono-functionalisation are shown below. Competitive formation of rearranged product **15** was observed in the absence of PivOH (columns **1** and **9**). The ratio of product **5** vs product **15** is shown on the relevant well(s).

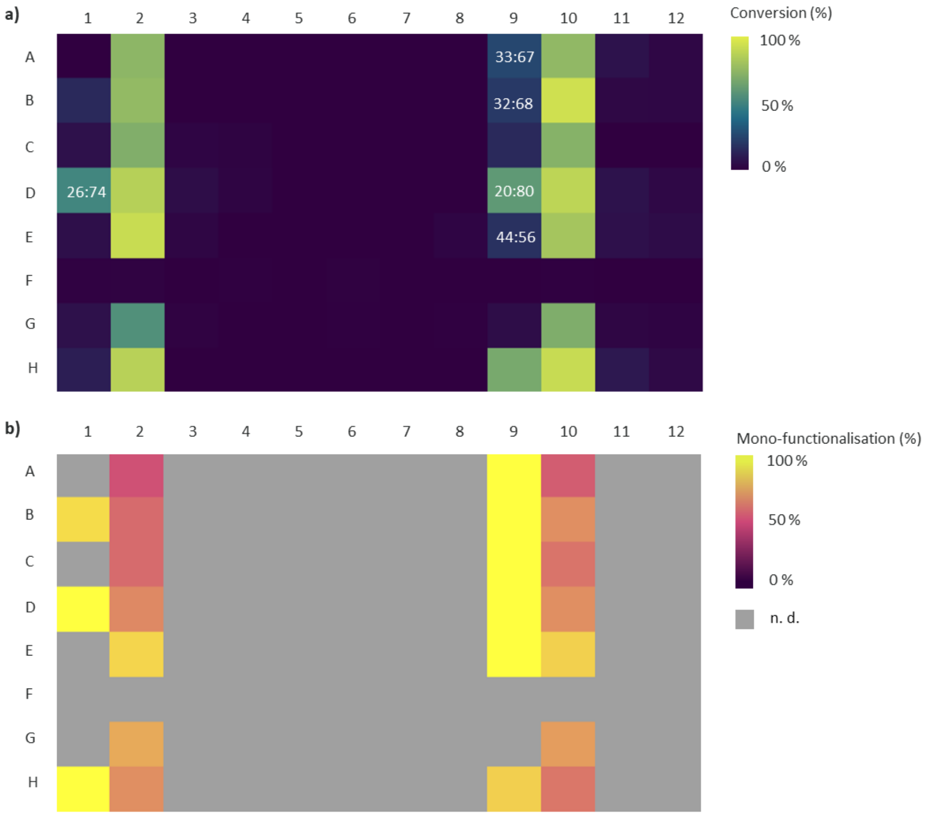

Supplementary Figure 5. Evaluation of solvents and silver salt replacements. **a)** Heat map for conversion (%): determined by LCMS, based on the relative intensities of the product peak(s) vs substrate peak in the UV chromatogram. **b)** Heat map for mono-functionalisation (%): determined by LCMS, based on the relative intensity of the mono-functionalised product peak vs bis-functionalisation peak in the UV chromatogram. n. d. = Not determined due to low conversion (< 10%).

4.4 Ag salt and PivOH equivalent

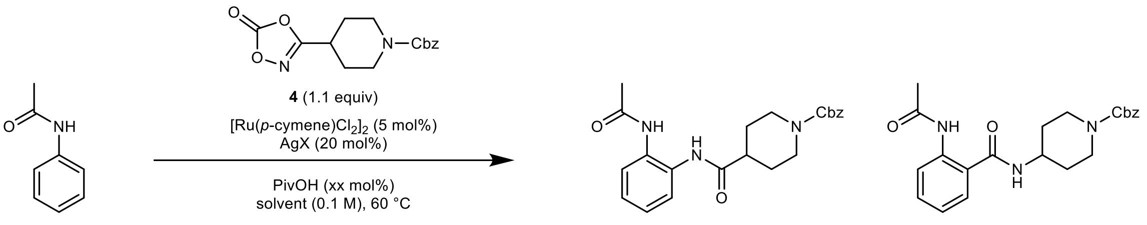

2 5 15

Ag salt		PivOH		Solvent	
**A**	none	**1**	0 mol%	**1**	DCE
**B**	$AgPF_6$	**2**	10 mol%	**2–6**	TFE
**C**	$AgSbF_6$	**3**	30 mol%		
**D**	$AgNTf_2$	**4**	50 mol%		
**E**	$AgBF_4$	**5**	100 mol%		
**F**	AgOTf	**6**	200 mol%		

Results

Heat maps visualising conversion (%) and mono-functionalisation are shown below. Competitive formation of rearranged product **15** was observed in the absence of PivOH (column **1**). The ratio of product **5** *vs.* product **15** is shown on the relevant well(s).

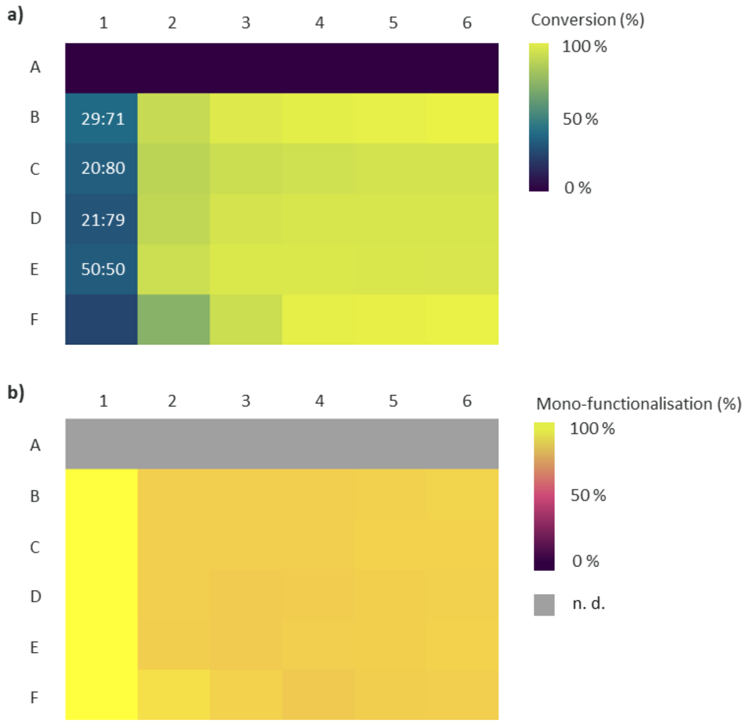

Supplementary Figure 6. Evaluation of silver salt and PivOH equivalents. **a)** Heat map for conversion (%): determined by LCMS, based on the relative intensities of the product peak(s) *vs.* substrate peak in the UV chromatogram. **b)** Heat map for mono-functionalisation (%): determined by LCMS, based on the relative intensity of the mono-functionalised product peak vs. bis-functionalisation peak in the UV chromatogram. n. d. = Not determined due to low conversion (< 10%).

4.5 Characterisation of products 5, 6, 15 and 16

Benzyl 4-((2-acetamidophenyl)carbamoyl)piperidine-1-carboxylate (5)

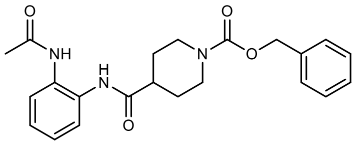

Prepared according to the general purification procedure for optimisation substrates, combining the reaction mixtures from wells G7, G8, H7 and H8 (Supplementary Figure 1). Purification by preparative reverse phase HPLC (15-65% MeCN in NH_4HCO_3 buffer, 240 nm) afforded amidated derivative **5** as a white solid (21.5 mg).

^{1}H NMR (500 MHz, $CDCl_3$) δ (ppm) 8.50 (s, 1 H), 8.17 (s, 1 H), 7.41–7.35 (m, 4 H), 7.35–7.29 (m, 2 H), 7.24 (dd, *J* = 7.2, 2.3 Hz, 1 H), 7.20–7.12 (m, 2 H), 5.14 (s, 2 H), 4.25 (br s, 2 H), 2.87 (br s, 2 H), 2.37 (tt, *J* = 11.3, 2.4 Hz, 1 H), 2.10 (s, 3 H), 1.90–1.76 (br m, 2 H), 1.67 (qd, *J* = 12.0, 4.3 Hz, 2 H); **^{13}C NMR** (126 MHz, $CDCl_3$) δ (ppm) 173.9, 170.0, 155.2, 136.7, 130.7, 130.5, 128.6 (2 C), 128.1, 128.0 (2 C), 126.5, 126.2, 125.8, 125.7, 67.2, 43.4 (3 C), 28.4 (2 C), 23.7; **HRMS** (m/z): $[M+H]^+$ calcd. for $C_{22}H_{25}N_3O_4$, 396.1918; found, 396.1923.

Benzyl 4-(2-acetamidobenzamido)piperidine-1-carboxylate (15)

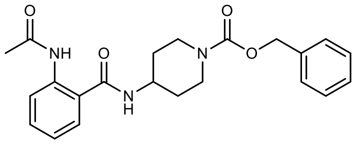

Prepared according to the general purification procedure for optimisation substrates, combining the reaction mixtures from wells E5–7 and F7 (Supplementary Figure 1). Purification by preparative reverse phase HPLC (15-65% MeCN in NH_4HCO_3 buffer, 240 nm) afforded amidated derivative **15** as a white solid (15.4 mg).

^{1}H NMR (500 MHz, $CDCl_3$) δ (ppm) 10.94 (s, 1 H), 8.54 (d, *J* = 8.4 Hz, 1 H), 7.45 (ddd, *J* = 8.7, 7.4, 1.5 Hz, 1 H), 7.42 (dd, *J* = 7.9, 1.5 Hz, 1 H), 7.40–7.29 (m, 5 H), 7.05 (td, *J* = 7.6, 1.2 Hz, 1 H), 6.28 (d, *J* = 7.7 Hz, 1 H), 5.13 (s, 2 H), 4.21 (br s, 2 H), 4.10 (dtt, *J* = 11.2, 7.3, 3.9 Hz, 1 H), 2.99 (br s, 2 H), 2.18 (s, 3 H), 2.10– 1.98 (m, 2 H), 1.46 (br s, 2 H); **^{13}C NMR** (126 MHz, $CDCl_3$) δ (ppm) 169.0, 168.5, 155.2, 139.5, 136.6, 132.7, 128.6 (2 C), 128.1, 128.0 (2 C), 126.5, 122.7, 121.6, 120.2, 67.3, 47.3, 42.9 (2 C), 31.9 (br, 2 C), 25.3; **HRMS** (m/z): $[M+H]^+$ calcd. for $C_{22}H_{25}N_3O_4$, 396.1918; found, 396.1943.

Benzyl 4-((2-(pyridin-2-yl)phenyl)carbamoyl)piperidine-1-carboxylate (6)

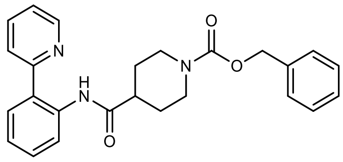

Prepared according to the general purification procedure for optimisation substrates, combining the reaction mixtures from wells C1–3 and D1–2 (Supplementary Figure 1). Purification by preparative reverse phase HPLC (35-75% MeCN in NH_4HCO_3 buffer, 254 nm) afforded amidated derivative **6** as a white solid (42.3 mg).

^{1}H NMR (500 MHz, $CDCl_3$) δ (ppm) 12.35 (s, 1 H), 8.59 (ddd, *J* = 4.9, 1.9, 0.9 Hz, 1 H), 8.55 (dd, *J* = 8.4, 1.3 Hz, 1 H), 7.85 (td, *J* = 7.8, 1.9 Hz, 1 H), 7.76 (dt, *J* = 8.2, 1.1 Hz, 1 H), 7.67 (dd, *J* = 7.9, 1.6 Hz, 1 H), 7.41 (ddd, *J* = 8.5, 7.3, 1.6 Hz, 1 H), 7.39–7.34 (m, 4 H), 7.34–7.30 (m, 1 H), 7.28 (ddd, *J* = 7.5, 4.9, 1.2 Hz, 1 H), 7.17 (td, *J* = 7.6, 1.3 Hz, 1 H), 5.15 (s, 2 H), 4.25 (br s, 2 H), 2.94 (br s,

2 H), 2.47 (tt, J = 11.4, 3.7 Hz, 1 H), 2.07–1.91 (br m, 2 H), 1.75 (br s, 2 H); **^{13}C NMR** (126 MHz, $CDCl_3$) δ (ppm) 172.7, 158.2, 155.2, 147.2, 137.9, 137.5, 136.8, 130.2, 128.8, 128.5 (2 C), 128.0, 127.9 (2 C), 125.6, 123.7, 123.1, 122.1 (2 C), 67.2, 44.7 (2 C), 43.6, 28.6 (br, 2 C); **HRMS** (m/z): $[M+H]^+$ calcd. for $C_{25}H_{25}N_3O_3$, 416.1969; found, 416.1972.

Benzyl 4-((2-(methylcarbamoyl)phenyl)carbamoyl)piperidine-1-carboxylate (16)

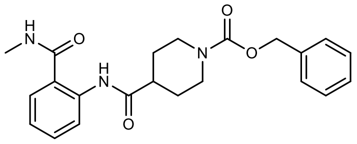

Prepared according to the general purification procedure for optimisation substrates, combining the reaction mixtures from wells A9–11 and B11 (Supplementary Figure 2). Purification by preparative reverse phase HPLC (15-65% MeCN in NH_4HCO_3 buffer, 254 nm) afforded amidated derivative **16** as a white solid (19.9 mg).

^{1}H NMR (500 MHz, $CDCl_3$) δ (ppm) 11.34 (s, 1 H), 8.55 (d, J = 7.7 Hz, 1 H), 7.47–7.39 (m, 2 H), 7.39–7.28 (m, 5 H), 7.02 (td, J = 7.6, 1.2 Hz, 1 H), 6.65 (q, J = 5.5 Hz, 1 H), 5.12 (s, 2 H), 4.24 (br s, 2 H), 2.97 (d, J = 4.8 Hz, 3 H), 2.88 (br s, 2 H), 2.46 (tt, J = 11.6, 3.7 Hz, 1 H), 1.97 (br s, 2 H), 1.75 (qd, J = 12.1, 4.3 Hz, 2 H); **^{13}C NMR** (126 MHz, $CDCl_3$) δ (ppm) 173.2, 169.7, 155.2, 139.5, 136.7, 132.5, 128.5 (2 C), 128.0, 127.9 (2 C), 126.6, 122.8, 121.5, 120.2, 67.2, 44.5 (2 C), 43.5, 28.4 (2 C), 26.8; **HRMS** (m/z): $[M+H]^+$ calcd. for $C_{22}H_{25}N_3O_4$, 396.1918; found, 396.1921.

5. LSF informer library

A library of 48 commercial drugs was evaluated in the C–H amidation reaction using dioxazolone **4** as coupling partner. The compounds were clustered into two groups based on the type of inherent functional group(s) available to direct the C–H activation (type I or II). Each group of 24 compounds was screened in a 96-wells Paradox plate against a selection of three reaction conditions, as outlined below (Supplementary Figures 5 and 6). The reactions were analysed by LCMS and the data shown is based on the relative intensities of the product peak(s) vs substrate peak and other major by-products in the UV chromatogram; i.e. relative product abundance.

Experimental set-up The reactions were set-up using 96-wells Para-dox plates with 1 mL glass vials equipped with stirrer bars on a 0.025 mmol substrate scale. On the benchtop, the vials were manually charged with the appropriate LSF substrates. The plates were moved into a glovebox under nitrogen, where (Ru(*p*-cymene)Cl_2)$_2$ (0.765 mg, 1.25 µmol) and all solid additives were added using a Mettler Toledo Quantos system for automated solid dispensing. Dioxazolone **4** was then added as stock solutions in the required solvents for a total volume of 250 µl (0.1 M). The plates were sealed with a teflon film, taken out of the glovebox and heated at 60 °C under stirring (600 rpm). After 16 hours the reaction mixtures were allowed to cool down, diluted with DMSO (500 µl) and stirred at room temperature for 5 min. A 50 µl aliquot was extracted from each vial, transferred to a 96-well Greiner_V plastic plate and diluted with further DMSO (50 µl). The solids were centrifuged with Eppendorf Centrifuge 5810 R (room temperature, atmospheric pressure, 3000 rpm, 15 min). A 25 µl aliquote was extracted from each well and transferred to a fresh 96-well Greiner_V plastic plate. The wells were finally diluted with further DMSO (75 µl) and analysed by LCMS both under basic and acidic conditions [Waters Acquity UPLC system, BEH C18 column (A: H_2O/MeCN/NH_3 = 95/5/0.2, B: MeCN) and HSS C18 column (A: H_2O/MeCN/HCO_2H = 95/5/0.2, B: MeCN)].

5.1 Type I inherent directing groups – Plate α

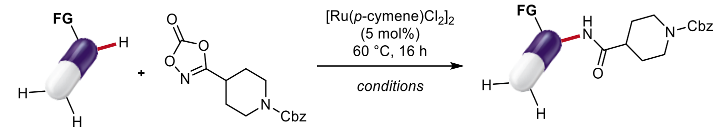

Type I FGs
24 drug molecules

4 (1.1 equiv)

Conditions

row	additive 30 mol%	AgX 20 mol%	solvent (0.1 M)
**A**	PivOH	$AgSbF_6$	DME:DMA 9:1
**B**	PivOH	$AgSbF_6$	DME
**C**	PivOH	$AgPF_6$	TFE

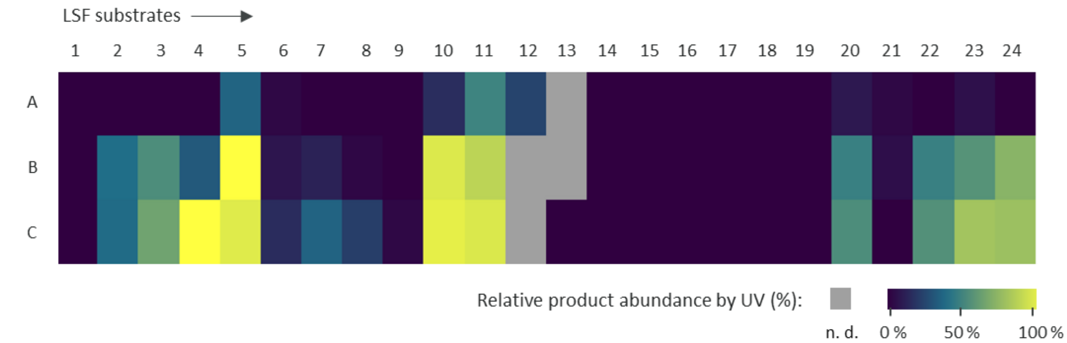

1 - *Leflumonide*

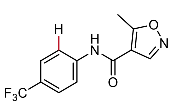

A [n.r.]
B [n.r.]
C [n.r.]

2 - *Flutamide*

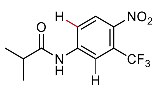

A [n.r.]
B [29%]
C [29%]

3 - *Tezacaftor*

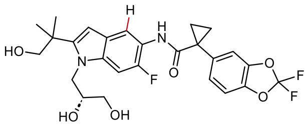

A [0%][a]
B [44%]
C [55%]

4 - *Tranilast*

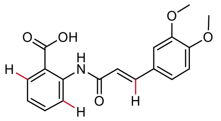

A [n.r.]
B [24%][b]
C [100%]

5 - *Tolvaptan*

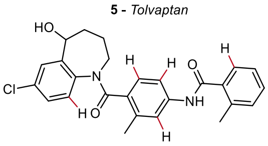

A [27%][c]
B [100%]
C [90%]

6 - *Boscalid*

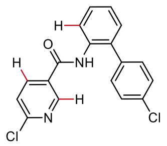

A [traces]
B [traces]
C [12%]

7 - *Rivaroxaban*

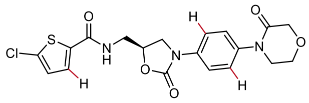

A [n.r.]
B [traces]
C [27%, 1:1 mono:bis]

8 - *Indomethacin*

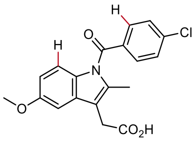

A [n.r.]
B [traces]
C [16%]

9 - *Vismodegib*

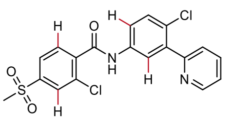

A [n.r.]
B [n.r.]
C [traces]

10 - *Tamibarotene*

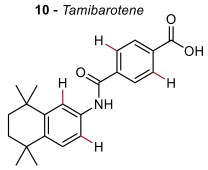

A [12%]
B [89%, 8:2 mono:bis]
C [92%]

11 - *Bezafibrate*

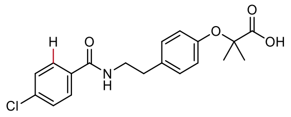

A [40%]
B [79%]
C [88%]

12 - *Paclitaxel*

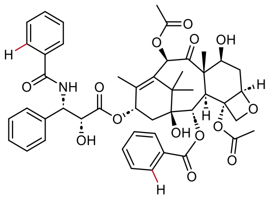

A [18%][d]
B [n.d., decomposition]
C [n.d., decomposition]

13 - *Betrixaban*

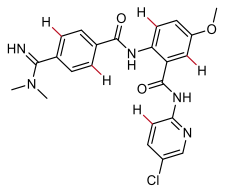

A [n.d., complex crude]
B [n.d., complex crude]
C [n.r.]

14 - *Apixaban*

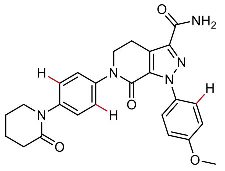

A [n.r.]
B [n.r.]
C [n.r.]

continue...

Supplementary Figure 7. LSF informer library focusing on type I inherent directing groups (24 drug compounds). The reactions were analysed by LCMS and the data shown in brackets is based on the relative intensities of the desired product peak vs substrate peak and other major by-products in the UV chromatogram (i.e. relative product abundance). n. d. = Not determined due to decomposition or significant by-product(s) formation. n. r. = No reaction (only unreacted starting material remaining). For each compound, potential C–H amidation sites are highlighted in red. [a] Two peaks consistent with -OH acylation were observed by LCMS (1st: 37%, not isolated; 1.44 min, m/z: 780.30. 2nd: 32%, not isolated; 1.46 min, m/z: 780.30). [b] Two peaks consistent with decarboxylation either of substrate (16%, not isolated; 1.07 min, m/z: 283.12) or desired product (49%, not isolated; 1.22 min, m/z: 543.24) were observed by LCMS. [c] A peak consistent with -OH acylation was observed by LCMS (19%, not isolated; 1.47 min, m/z: 709.24). [d] 39% -OH acylation was observed by LCMS (compound **28**).

...continue

15 - *Sonidegib*

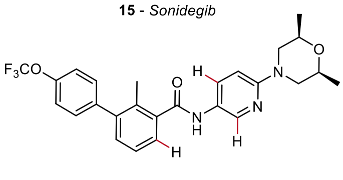

A [n.r.]
B [n.r.]
C [n.r.]

16 - *Axitinib*

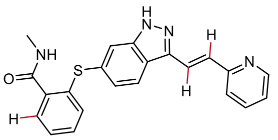

A [0%][e]
B [0%][e]
C [0%][e]

17 - *Sorafenib*

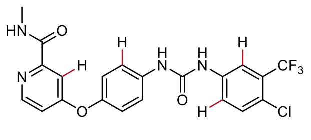

A [n.r.]
B [n.r.]
C [n.r.]

18 - *Gliquidone*

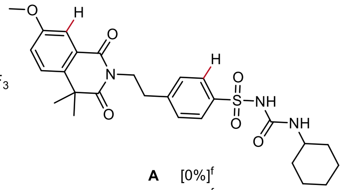

A [0%][f]
B [0%][f]
C [0%][g]

19 - *Ivacaftor*

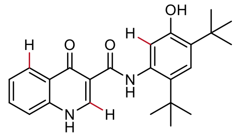

A [n.r.]
B [n.r.]
C [n.r.]

20 - *Tolmetin*

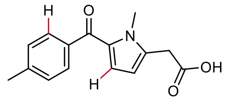

A [traces]
B [38%]
C [44%]

21 - *Warfarin*

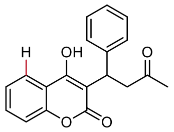

A [traces]
B [traces]
C [n.r.]

22 - *Fenofibrate*

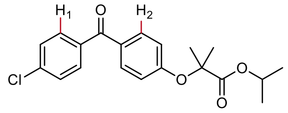

A [n.r.]
B [38%, 1:1 ratio H_1:H_2]
C [46%, 1:1 ratio H_1:H_2]

23 - *Trametinib*

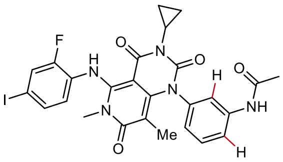

A [traces]
B [47%, 1:9 mono:bis]
C [71%, 7:3 mono:bis]

24 - *Atorvastatin*

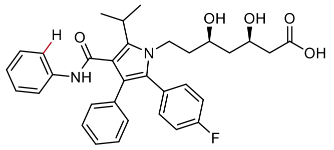

A [n.r.]
B [63%][h]
C [69%][h]

Supplementary Figure 7 (continue). LSF informer library focusing on type I inherent directing groups (24 drug compounds). The reactions were analysed by LCMS and the data shown in brackets is based on the relative intensities of the desired product peak vs substrate peak and other major by-products in the UV chromatogram (i.e. relative product abundance). n. d. = Not determined due to decomposition or significant by-product(s) formation. n. r. = No reaction (only unreacted starting material remaining). For each compound, potential C–H amidation sites are highlighted in red. [e] Exclusive -NH acylation was observed by LCMS (70–89%). [f] A peak consistent with sulphonylurea hydrolysis followed by acylation was observed by LCMS (47-49%, not isolated; 0.99 min, m/z = 662.76). [g] A peak consistent with sulphonylurea hydrolysis was observed by LCMS (73%, not isolated; 1.10 min, m/z = 402.47). [h] A peak consistent with dehydration of the desired product was observed by LCMS (21-31%, not isolated; 1.37 min, m/z = 800.93).

5.2 Type II inherent directing groups – Plate β

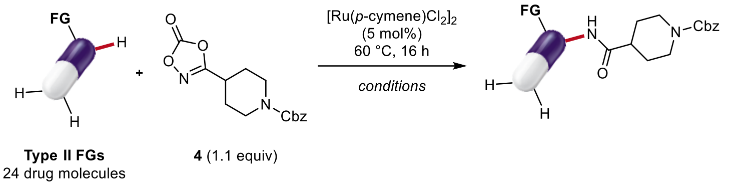

Conditions

row	additive 30 mol%	AgX 20 mol%	solvent (0.1 M)
**A**	$(PhO)_2PO_2H$	$AgSbF_6$	DME:DMA 9:1
**B**	$(PhO)_2PO_2H$	$AgSbF_6$	DME
**C**	PivOH	$AgPF_6$	TFE

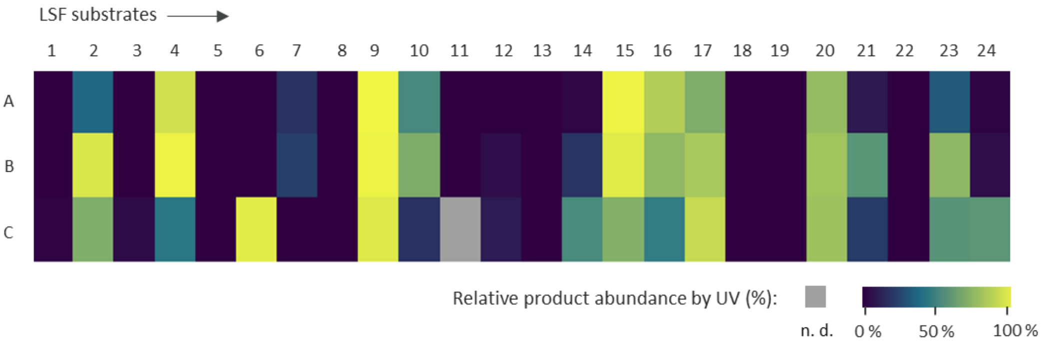

1 - *Telmisartan*

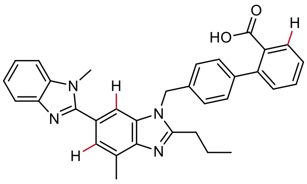

A [n.r.]
B [n.r.]
C [n.r.]

2 - *Pritelivir*

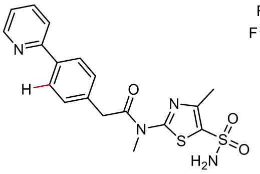

A [27%]
B [88%]
C [61%]

3 - *Lumacaftor*

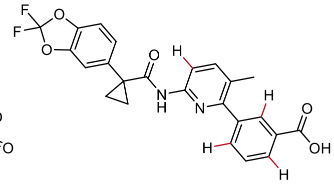

A [n.r.]
B [n.r.]
C [traces]

4 - *Atazanavir*

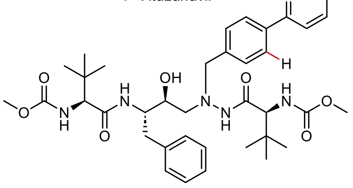

A [85%]
B [94%, 9:1 mono:bis]
C [34%]

5 - *Rimonabant*

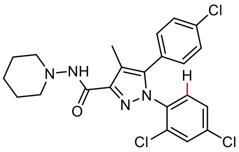

A [n.r.]
B [n.r.]
C [n.r.]

6 - *Celecoxib*

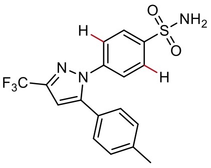

A [0%][a]
B [0%][a]
C [91%]

7 - *Levamisole*

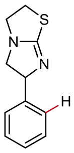

A [13%]
B [17%]
C [n.r.]

8 - *Capmatinib*

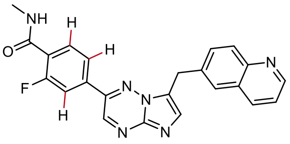

A [n.r.]
B [n.r.]
C [n.r.]

9 - *Oxaprozin*

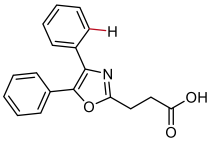

A [95%]
B [94%]
C [90%]

10 - *Zolpidem*

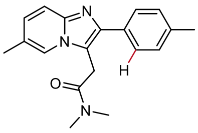

A [42%, 9:1 mono:bis][b]
B [60%, 8:2 mono:bis]
C [13%]

11 - *Valsartan*

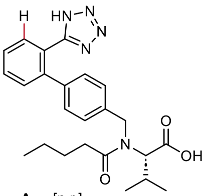

A [n.r.]
B [n.r.]
C [n.d., decomposition]

12 - *Ataluren*

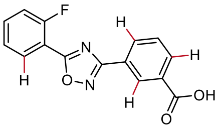

A [n.r.]
B [traces]
C [traces]

13 - *Febuxostat*

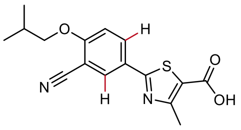

A [n.r.]
B [n.r.]
C [n.r.]

14 - *Parecoxib*

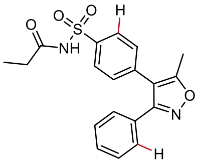

A [n.r.]
B [14%]
C [43%]

continue...

Supplementary Figure 8. LSF informer library focusing on type II inherent directing groups (24 drug compounds). The reactions were analysed by LCMS and the data shown in brackets is based on the relative intensities of the desired product peak vs substrate peak and other major by-products in the UV chromatogram (i.e. relative product abundance). n. d. = Not determined due to decomposition or significant by-product(s) formation. n. r. = No reaction (only unreacted starting material remaining). For each compound, potential C–H amidation sites are highlighted in red. [a] Exclusive sulphonamide acylation was observed by LCMS (68-81%, compound **27**). [b] A peak consistent with carboxamidation (C–C bond formation) was observed by LCMS (12%, not isolated; 1.20 min, m/z: 567.28).

...continue

15 - *Emapunil*

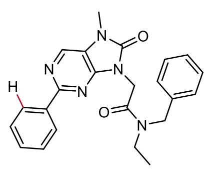

A [94%, 8:2 mono:bis]
B [90%, 8:2 mono:bis]
C [61%, 4:6 mono:bis]

16 - *Selexipag*

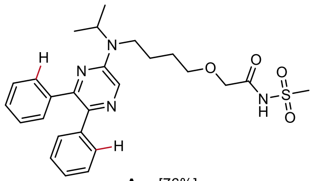

A [76%]
B [65%]
C [36%][c]

17 - *Sulfaphenazole*

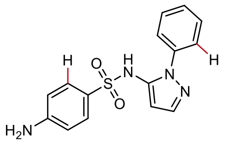

A [60%, 7:3 mono:bis][d]
B [73%, 7:3 mono:bis][d]
C [82%, 9:1 mono:bis]

18 - *Pyridafol*

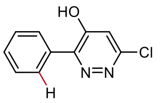

A [n.r.]
B [n.r.]
C [n.r.]

19 - *Nilotinib*

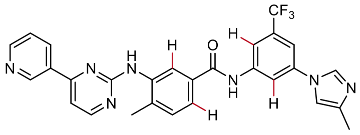

A [n.r.]
B [n.r.]
C [n.r.]

20 - *Niflumic acid*

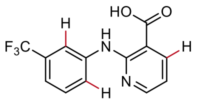

A [67%]
B [71%]
C [70%]

21 - *Diazepam*

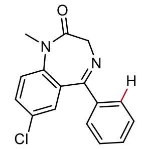

A [7%]
B [48%]
C [16%]

22 - *Minaprine hydrocloride*

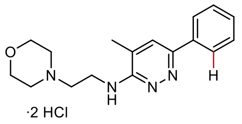

A [n.r.]
B [n.r.]
C [n.r.]

23 - *Talniflumate*

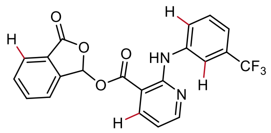

A [24%]
B [65%]
C [48%]

24 - *Diflufenican*

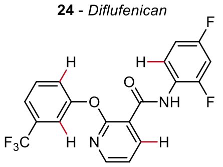

A [n.r.]
B [traces]
C [49%]

Supplementary Figure 8 (continue). LSF informer library focusing on type II inherent directing groups (24 drug compounds). The reactions were analysed by LCMS and the data shown in brackets is based on the relative intensities of the desired product peak vs substrate peak and other major by-products in the UV chromatogram (i.e. relative product abundance). n. d. = Not determined due to decomposition or significant by-product(s) formation. n. r. = No reaction (only unreacted starting material remaining). For each compound, potential C–H amidation sites are highlighted in red. [c] A peak consistent with a minor regioisomeric product was observed by LCMS (13%, not isolated; 1.07 min, m/z: 756.33). [d] A peak consistent with carboxamidation (C–C bond formation) was observed by LCMS (9-16%, not isolated; 0.84 min, m/z: 574.20).

6. Single crystal X-ray diffraction and structure refinements for compound 8r

Single crystals of compound **8r** were grown by slow solvent evaporation method using methanol solvent at 25 °C. A suitable single crystal was mounted on an XtaLab Synergy-S diffractometer (Rigaku, Japan) equipped with a HyPix-Arc 100 curve detector (Rigaku, Japan) and an Oxford Cryostream 800 (Oxford Cryosystem, UK). Data were measured using Cu $K\alpha$ radiation generated from a micro focus sealed tube (50 kV, 1 mA) at 100K. Measurement strategy was calculated using CrysAlisPro software 1.171.42.35a.[2] Data reduction and correction were performed using CrysAlisPro software[2] where numerical absorption correction based on gaussian integration over a multifaceted crystal model and empirical absorption correction using spherical harmonics, implemented in SCALE3 ABSPACK scaling algorithm were used. The structure was solved with Olex2.solve[3] structure solution program using the direct methods solution method within Olex2.[4] The model was refined on F_o^2 with ShelXL 2014.[5] All non-hydrogen atoms were refined anisotropically. All hydrogen atoms were determined geometrically and refined isotropically. CCDC 2251355 contains the supplementary crystallographic data for this paper. These data can be obtained free of charge via www.ccdc.cam.ac.uk/data_request/cif, or by emailing data_request@ccdc.cam.ac.uk, or by contacting The Cambridge Crystallographic Data Centre, 12 Union Road, Cambridge CB2 1EZ, UK; fax: +44 1223 336033.

Parameter	Compound 8r
Formula	$C_{40}H_{41}ClN_4O_6$
$D_{calc.}$/ g cm^{-3}	1.302
μ/mm^{-1}	1.368
Formula Weight	709.22
Colour	clear colourless
Shape	block-shaped
Size/mm^3	0.25×0.06×0.05
T/K	100(2)
Crystal System	monoclinic
Space Group	$P2_1/c$
a/Å	12.2128(2)
b/Å	23.7055(3)
c/Å	12.50160(10)
β/°	90.1160(10)
V/Å^3	3619.34(8)
Z,Z'	4, 1
Radiation type	CuK_a
Θ_{min}/°	3.619
Θ_{max}/°	79.443
Measured Reflections	26359
Independent Reflections	7222
Reflections I≥2σ(I)	6400
R_{int}	0.0304
Parameters	568
Restraints	130
Largest Peak	0.571
Deepest Hole	-0.377
GooF	1.100
wR_2 (all data)	0.1287
wR_2	0.1197
R_1 (all data)	0.0617
R_1	0.0542

Supplementary Table 1. Crystallographic data of compound **8r.**

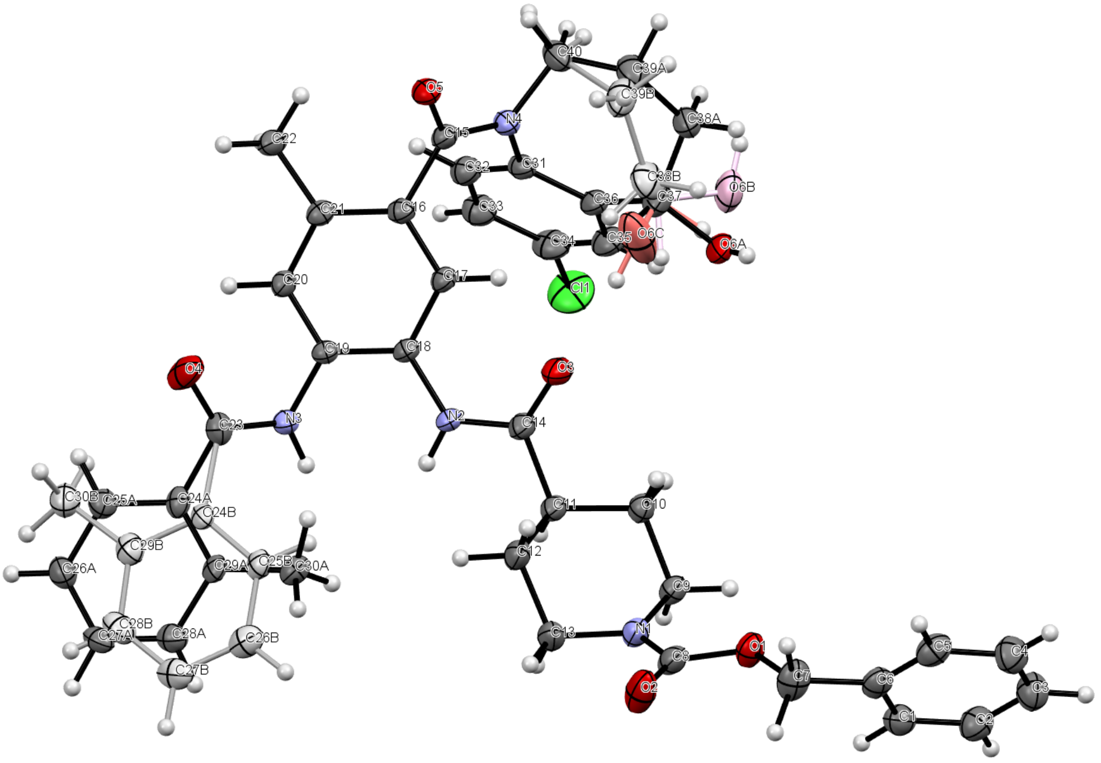

Supplementary Figure 9. The crystal structure of **8r** (50% probability ellipsoids).

7. Analysis of accessible exit vectors

The choice of exit vectors for linker attachment from a specific ligand is a critical parameter to develop successul PROTACs. In particular, the productive formation of a ternary complex and the subsequent degration cascade are only possible if the POI ligand is functionalised at a position that does not hamper binding to the target protein. In order to investigate whether the late-stage C–H amidation developed here can access suitable exit vectors for PROTAC generation, a number of published X-ray structures of successful LSF substrates (cf. Figure 4 in the main text) in complex with their protein targets were analysed. In several cases, C–H amidation occurs in a solvent exposed region of the ligand, in the presence of both type I and II directing groups (Supplementary Figures 8-11).

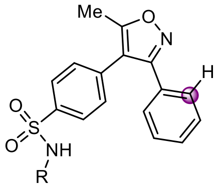

● = site of C–H amidation (cf. **8c**)

R = H **Valdecoxib**
R = COEt **Parecoxib**
(Type II DG)

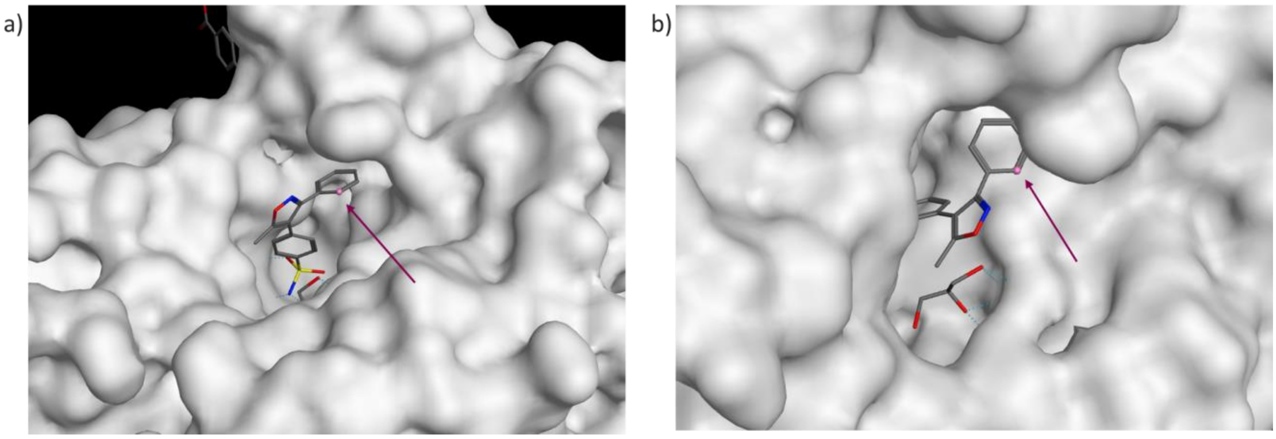

Supplementary Figure 10. X-ray crystal structure of the human Carbonic Anhydrase (CA, EC 4.2.1.1) isoform II (hCA II) in complex with valdecoxib.[6] The site of C–H amidation (highlighted in purple) is on a solvent exposed region of the ligand, suggesting this as suitable exit vector for linker attachment. a) Front view of the active site region in the hCA II–valdecoxib complex. b) Top view of the active site region in the hCA II–valdecoxib complex. Resolution = 1.40 Å. PDB structure: 2AW1; DOI: https://doi.org/10.2210/pdb2AW1/pdb. Protein surface modelled and image rendered using MOE 2022.02.

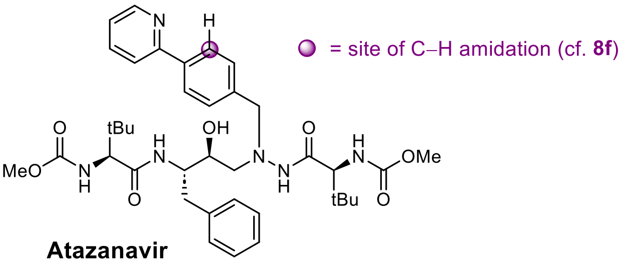

Atazanavir
(Type II DG)

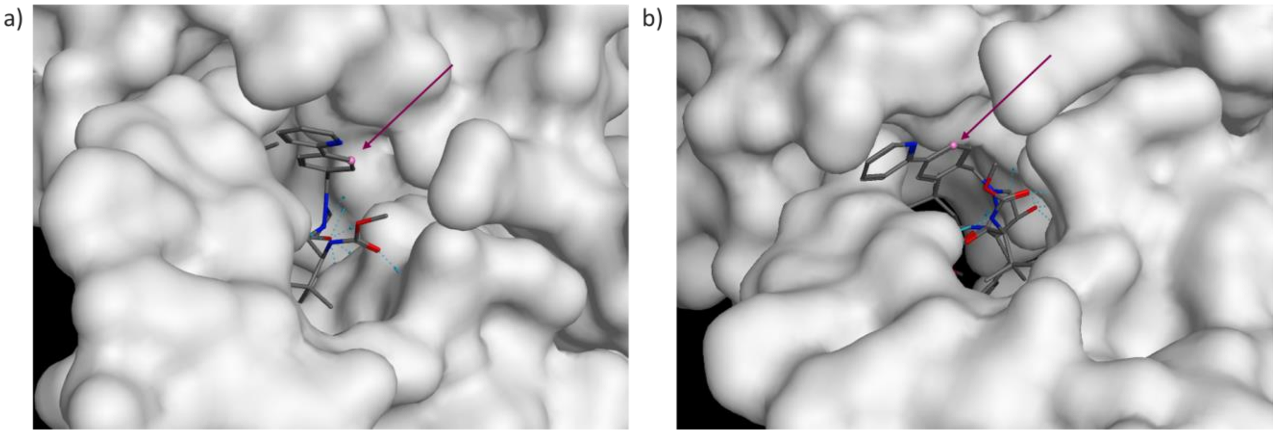

Supplementary Figure 11. X-ray crystal structure of the human immunodeficiency virus 1 (HIV-1) B protease in complex with atazanavir.[7] The site of C–H amidation (highlighted in purple) is on a solvent exposed region of the ligand, suggesting this as suitable exit vector for linker attachment. a) Front view of the active site region in the protease–atazanavir complex. b) Top view of the active site region in the protease–atazanavir complex. Resolution = 2.00 Å. PDB structure: 2AQU; DOI: https://doi.org/10.2210/pdb2AQU/pdb. Protein surface modelled and image rendered using MOE 2022.02.

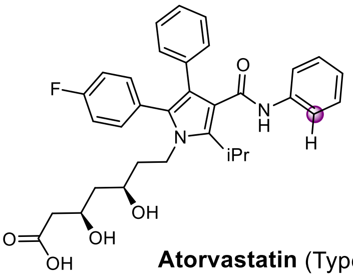

= site of C–H amidation (cf. **8p**)

Atorvastatin (Type I DG)

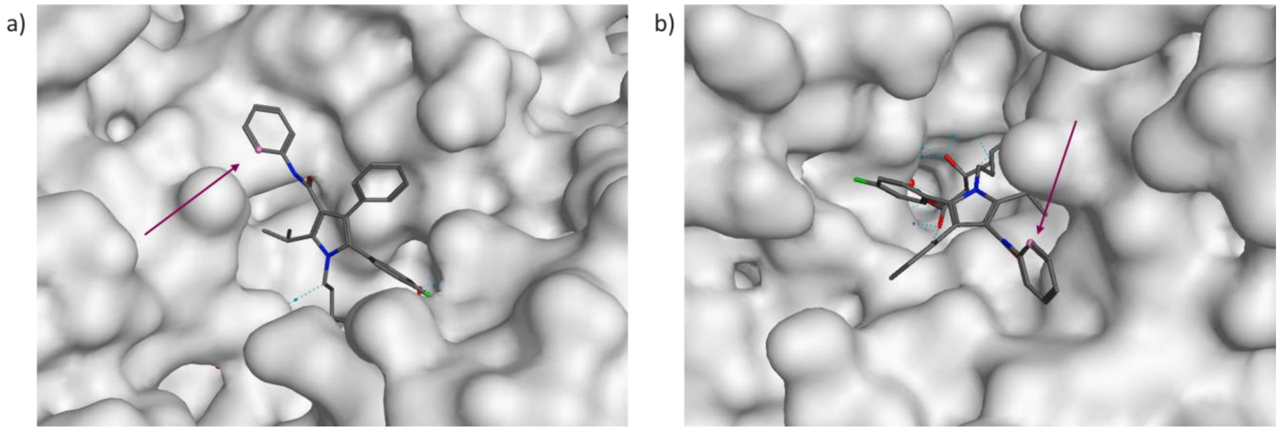

Supplementary Figure 12. X-ray crystal structure of the catalytic portion of the human HMG-CoA reductase (HMGR) with atorvastatin.[8] The site of C–H amidation (highlighted in purple) is on a solvent exposed region of the ligand, suggesting this as suitable exit vector for linker attachment. a) Front view of the binding pocket in the HMGR–atorvastatin complex. b) Top view of the binding pocket in the HMGR–atorvastatin complex. Resolution = 2.22 Å. PDB structure: 1HWK; DOI: https://doi.org/10.2210/pdb1HWK/pdb. Protein surface modelled and image rendered using MOE 2022.02.

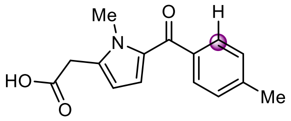

= site of C–H amidation (cf. **8v**)

Tolmetin (Type I DG)

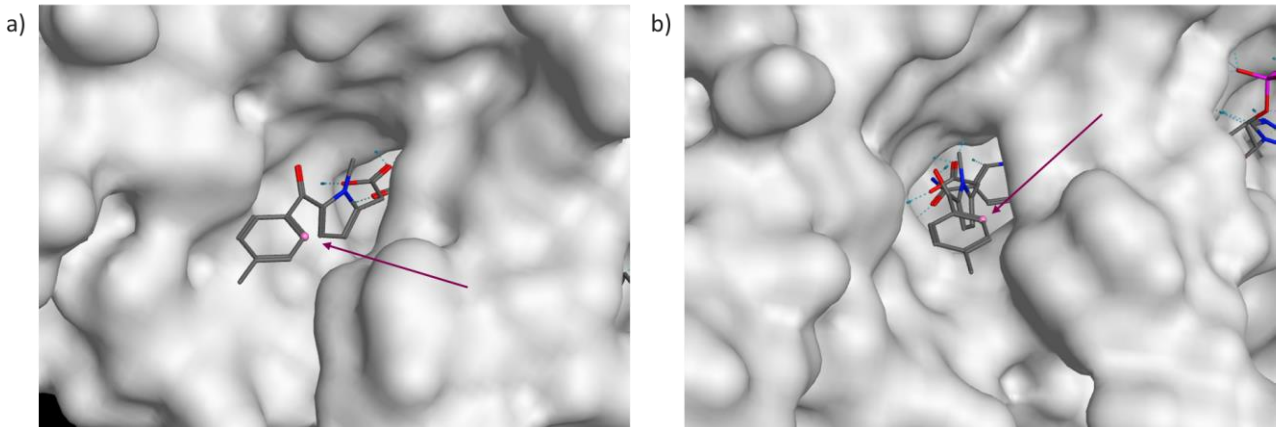

Supplementary Figure 13. X-ray crystal structure of the catalytic portion of the human aldose reductase with tolmetin.[9] The site of C–H amidation (highlighted in purple) is on a solvent exposed region of the ligand, suggesting this as suitable exit vector for linker attachment. a) Front view of the binding pocket in the reductase–tolmetin complex. b) Top view of the binding pocket in the reductase–tolmetin complex. Resolution = 1.80 Å. PDB structure: 3S3G; DOI: https://doi.org/10.2210/pdb3S3G/pdb. Protein surface modelled and image rendered using MOE 2022.02.

8. Proposed de novo syntheses

Sulfaphenazole derivative 14a

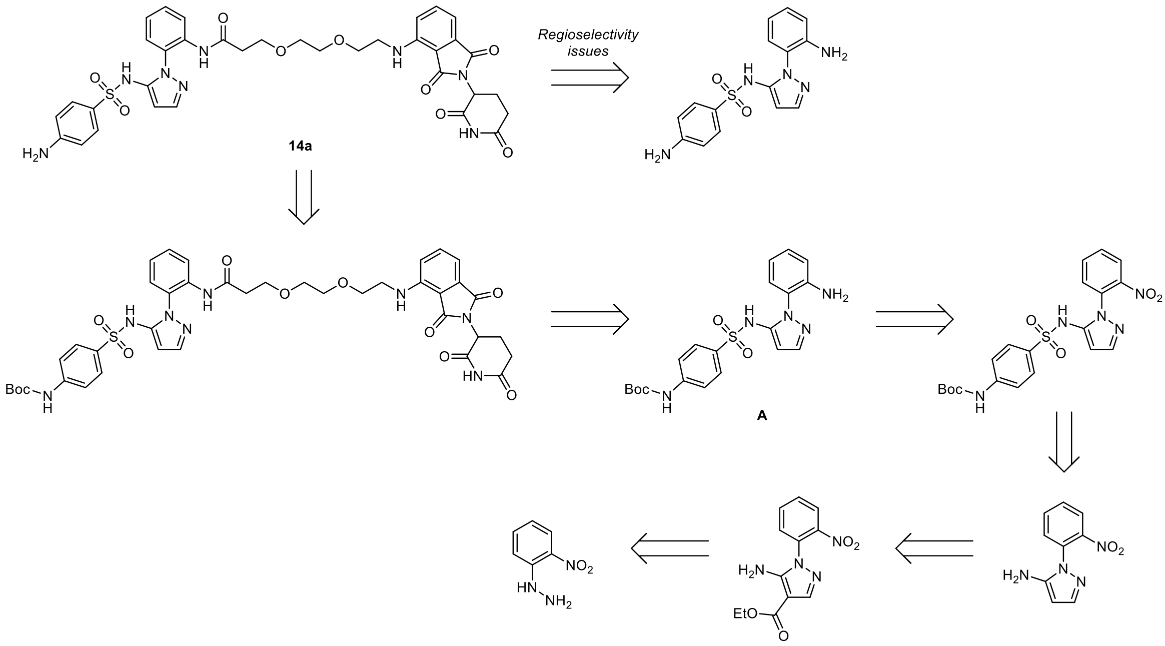

Supplementary Figure 14. Proposed route towards derivative **14a** based on sulfaphenazole and ethyl 5-amino-1-(2-nitrophenyl)-1*H*-pyrazole-4-carboxylate synthesis.[10,11,12] No known synthetic route of either **14a** or key intermediate **A**.

Emapunil derivative 14b

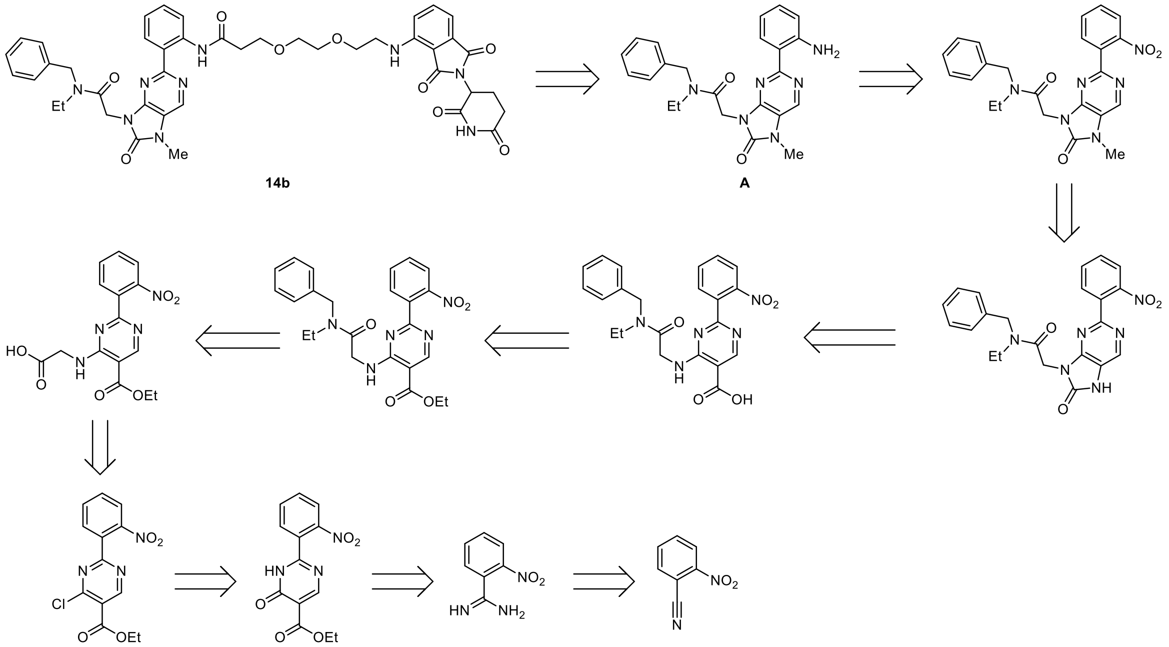

Supplementary Figure 15. Proposed route towards derivative **14b** based on based on the synthesis of emapunil and derivatives.[13,14] No known synthetic route of either **14b** or key intermediate **A**.

Celecoxib derivative 14c

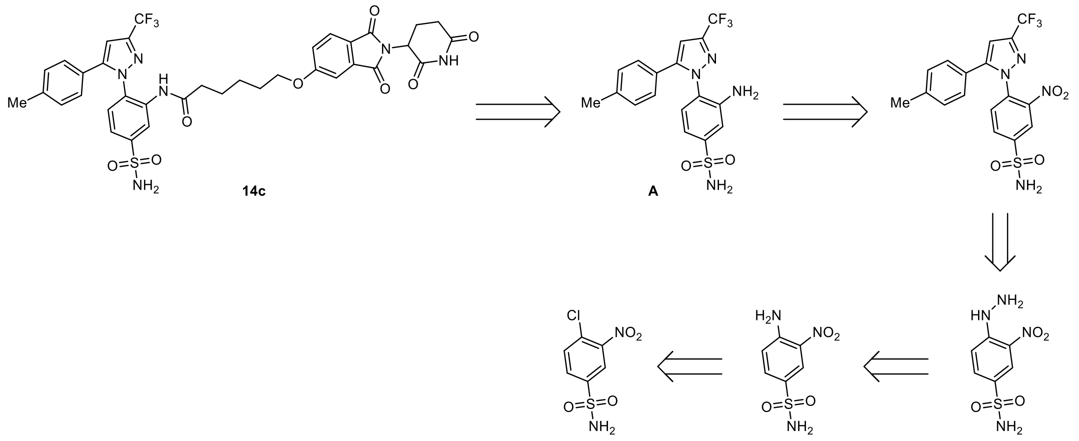

Supplementary Figure 16. Proposed route towards derivative **14c** based on the synthesis of celecoxib.[15] No known synthetic route of either **14c** or key intermediate **A**.

Oxaprozin derivatives 14d and 14g

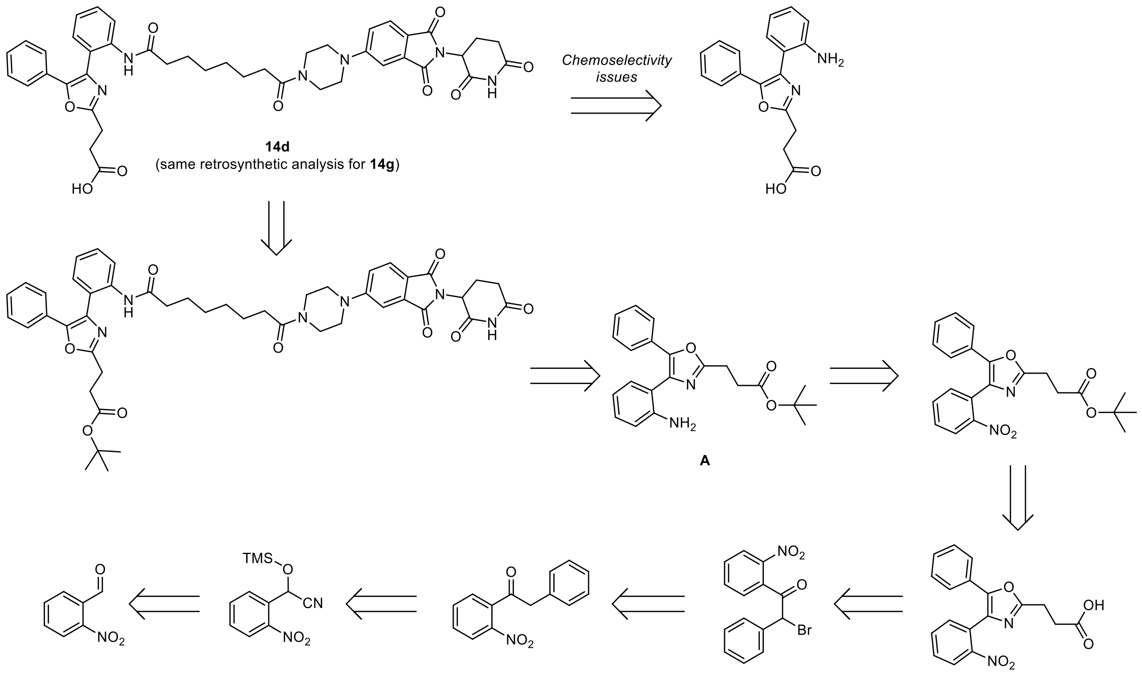

Supplementary Figure 17. Proposed route towards derivatives **14d** and **14g** based on the synthesis of oxaprozin.[16] No known synthetic route of either **14d/14g** or key intermediate **A**.

Atazanavir derivative 14e

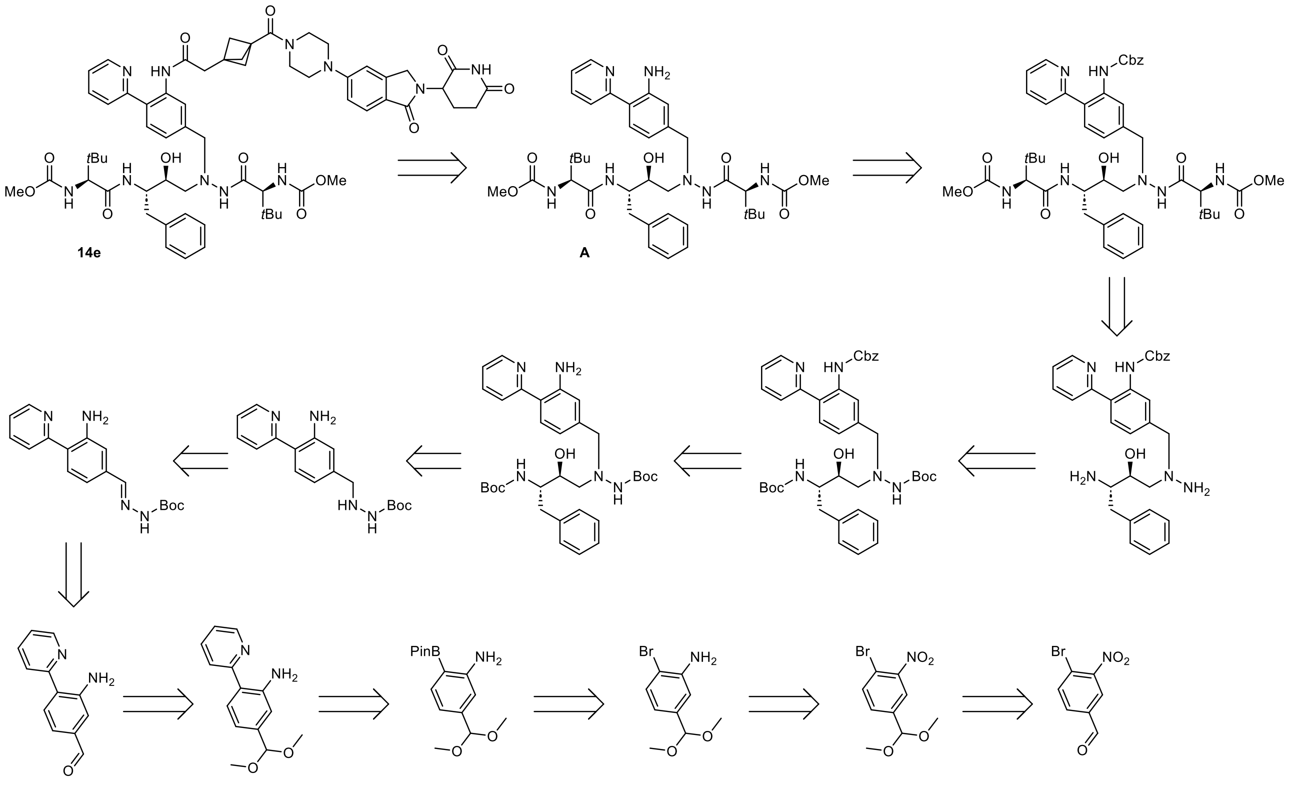

Supplementary Figure 18. Proposed route towards derivative **14e** based on the synthesis of atazanavir and 2-(pyridin-2-yl)aniline.[17,18] No known synthetic route of either **14e** or key intermediate **A**.

Tolvaptan derivative 14f

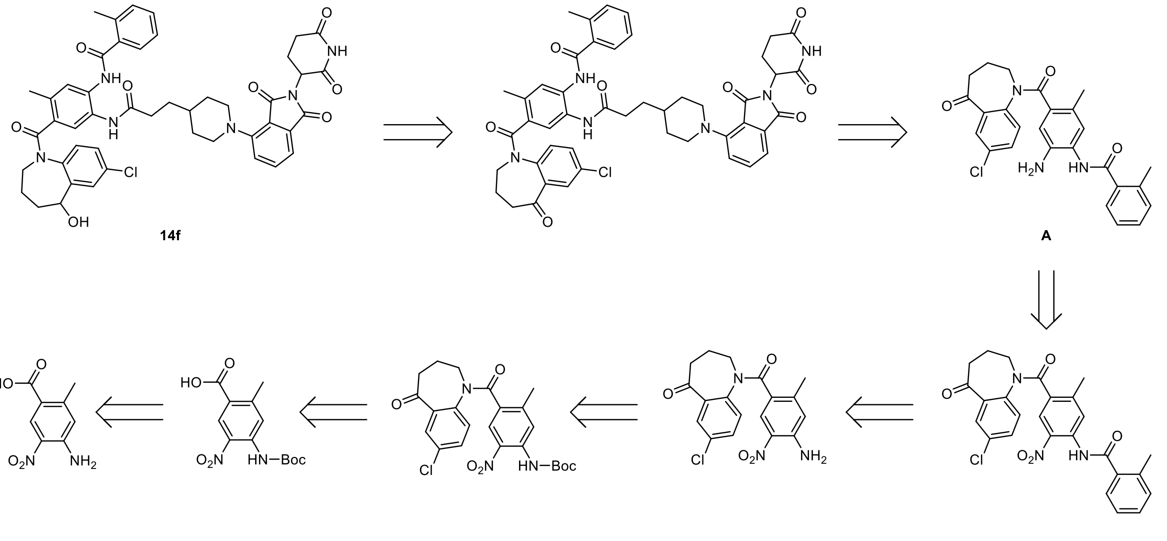

Supplementary Figure 19. Proposed route towards derivative **14f** based on the synthesis of tolvaptan and derivatives.[19] No known synthetic route of either **14f** or key intermediate **A**.

9. Experimental details and characterisation data

For characterisation of compounds **5**, **6**, **15** and **16**, see optimisation section (Supplementary Section 4.5).

9.1 Dioxazolone reagents (4, 9a-9j, 13a-13e, 17-26)

General scheme for dioxazolone synthesis

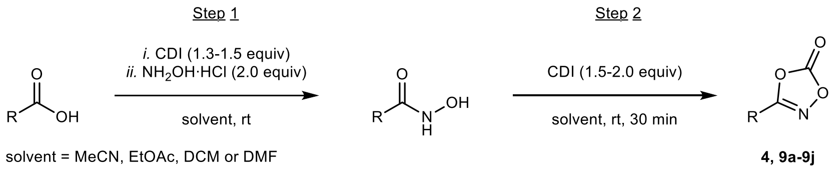

Benzyl 4-(5-oxo-1,4,2-dioxazol-3-yl)piperidine-1-carboxylate (4)

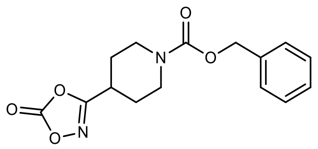

Stepwise procedure.
<u>Step 1:</u> In an oven-dried round bottom flask, 1,1'-carbonyl diimidazole (CDI, 2.72 g, 16.8 mmol, 1.3 equiv) was added to a solution of 1-((benzyloxy)carbonyl)piperidine-4-carboxylic acid (3.40 g, 12.9 mmol) in dry MeCN (0.3 M, 45 mL). After 2 hours of stirring at room temperature under a N_2 atmosphere, hydroxylamine hydrochloride (1.80 g, 25.9 mmol, 2.0 equiv) was added and the resulting mixture was stirred overnight at room temperature. Subsequently, the reaction mixture was diluted with 5% aq. $KHSO_4$ (40 mL) and extracted with EtOAc (3 x 60 mL). The combined organic layer was washed with brine (100 mL), dried over $MgSO_4$ and concentrated in vacuo to afford the corresponding hydroxamic acid as a white solid. This was used in the following step without further purification.
<u>Step 2:</u> The crude hydroxamic acid was dissolved in dry CH_2Cl_2 (0.1 M, 50 mL) and 1,1'-carbonyl diimidazole (CDI, 1.31 g, 8.01 mmol, 1.5 equiv) was added portion wise. The reaction mixture was stirred at room temperature for 30 min, then diluted with aq. HCl (1 N, 30 mL) and CH_2Cl_2 (30 mL). The phases were separated and the aqueous layer was extracted with CH_2Cl_2 (3 x 50 mL). The combined organic layer was washed with brine (100 mL), dried over anhydrous $MgSO_4$, filtered, and concentrated under reduced pressure. The resulting residue was purified by filtration over a short SiO_2 plug eluting with CH_2Cl_2 to give dioxazolone **4** (1.11 g, 64% over 2 steps) as a white solid.
^{1}H NMR (500 MHz, $CDCl_3$) δ (ppm) 7.42–7.30 (m, 5 H), 5.14 (s, 2 H), 4.20 (br s, 2 H), 3.01 (br s, 2 H), 2.88 (tt, J = 11.0, 3.8 Hz, 1 H), 2.09–1.91 (m, 2 H), 1.84–1.66 (m, 2 H); **^{13}C NMR** (126 MHz, $CDCl_3$) δ (ppm) 167.8, 155.0, 153.8, 136.4, 128.6 (2 C), 128.2, 128.0 (2 C), 67.4, 42.7 (2 C), 32.9, 27.1 (2 C).

The spectroscopic data was consistent with what reported in the literature.[20]

(9*H*-Fluoren-9-yl)methyl (5-(5-oxo-1,4,2-dioxazol-3-yl)pentyl)carbamate (9a)

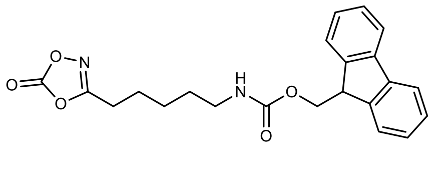

Stepwise procedure.

Step 1: In an oven-dried screw-cap vial, 1,1'-carbonyl diimidazole (CDI, 688 mg, 4.24 mmol, 1.5 equiv) was added to a solution of 6-((((9*H*-fluoren-9-yl)methoxy)carbonyl)amino)hexanoic acid (1.00 g, 2.83 mmol) in dry MeCN (0.5 M, 6.0 mL). After 2 hours of stirring at room temperature under a N_2 atmosphere, hydroxylamine hydrochloride (393 mg, 5.66 mmol, 2.0 equiv) was added and the resulting mixture was stirred overnight at room temperature. Subsequently, the reaction mixture was diluted with 5% aq. $KHSO_4$ (10 mL) and extracted with EtOAc (3 x 30 mL). The combined organic layer was washed with brine (50 mL), dried over $MgSO_4$ and concentrated in vacuo to afford the corresponding hydroxamic acid as a white solid. This was used in the following step without further purification.
Step 2: The crude hydroxamic acid was dissolved in dry EtOAc (0.1 M, 20 mL) and DMF (3.0 mL), and 1,1'-carbonyl diimidazole (CDI, 5.62 mg, 3.47 mmol, 1.5 equiv) was added in one portion. The reaction mixture was stirred at room temperature for 30 min, then diluted with aq. HCl (1 N, 30 mL) and EtOAc (20 mL). The phases were separated and the aqueous layer was extracted with EtOAc (3 x 40 mL). The combined organic layer was washed with brine (100 mL), dried over anhydrous $MgSO_4$, filtered, and concentrated under reduced pressure to afford pure dioxazolone **9a** (879 mg, 79% over 2 steps) as a white solid.
^{1}H NMR (500 MHz, $CDCl_3$, rotameric character of some aliphatic proton observed) δ (ppm) 7.77 (d, *J* = 7.5, 2 H), 7.59 (d, *J* = 7.8 Hz, 2 H), 7.40 (t, *J* = 7.6 Hz, 2 H), 7.32 (td, *J* = 7.4, 1.2 Hz, 2 H), 4.78 (t, *J* = 6.5 Hz, 1 H), 4.60–4.35 (m, 2 H), 4.21 (t, *J* = 6.8 Hz, 1 H), 3.27–2.96 (m, 2 H), 2.63 (t, *J* = 7.5 Hz, 2 H), 1.75 (p, *J* = 7.5 Hz, 2 H), 1.59–1.50 (m, part. overlap with H_2O signal, 2 H), 1.48–1.29 (m, 2 H); **^{13}C NMR** (126 MHz, $CDCl_3$) δ (ppm) 166.4, 156.5, 154.1, 143.9 (2 C), 141.4 (2 C), 127.7 (2 C), 127.0 (2 C), 125.0 (2 C), 120.0 (2 C), 66.5, 47.3, 40.5, 29.5, 25.7, 24.7, 24.1; **HRMS** (m/z): $[M+H]^+$ calcd. for $C_{22}H_{22}N_2O_5$, 395.1595; found, 395.1595.

Benzyl (3-(5-oxo-1,4,2-dioxazol-3-yl)propyl)carbamate (9b)

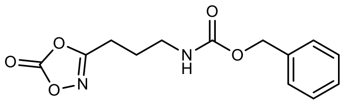

Stepwise procedure.
Step 1: In an oven-dried round bottom flask, 1,1'-carbonyl diimidazole (CDI, 5.13 g, 31.6 mmol, 1.5 equiv) was added to a solution of 4-(((benzyloxy)carbonyl)amino)butanoic acid (5.00 g, 21.1 mmol) in dry MeCN (0.3 M, 70 mL). After 2 hours of stirring at room temperature under a N_2 atmosphere, hydroxylamine hydrochloride (2.93 g, 42.2 mmol, 2.0 equiv) was added and the resulting mixture was stirred overnight at room temperature. Subsequently, the reaction mixture was diluted with 5% aq. $KHSO_4$ (50 mL) and extracted with EtOAc (3 x 80 mL). The combined organic layer was washed with brine (200 mL), dried over $MgSO_4$ and concentrated in vacuo to afford the corresponding hydroxamic acid as a pale blue solid. This was used in the following step without further purification.
Step 2: The crude hydroxamic acid was dissolved in dry CH_2Cl_2 (0.1 M, 150 mL), and 1,1'-carbonyl diimidazole (CDI, 5.07 g, 31.3 mmol, 1.5 equiv) was added portion wise. The reaction mixture was stirred at room temperature for 30 min, then diluted with aq. HCl (1 N, 100 mL) and CH_2Cl_2 (60 mL). The phases were separated and the aqueous layer was extracted with CH_2Cl_2 (3 x 120 mL). The combined organic layer was washed with brine (200 mL), dried over anhydrous $MgSO_4$, filtered, and concentrated under reduced pressure. The resulting residue was purified by filtration over a short SiO_2 plug eluting with CH_2Cl_2 to give dioxazolone **9b** (4.57 g, 78% over 2 steps) as a white solid.
^{1}H NMR (500 MHz, $CDCl_3$) δ (ppm) 7.42–7.29 (m, 5 H), 5.09 (s, 2 H), 4.98 (br s, 1 H), 3.31 (q, *J* = 6.5 Hz, 2 H), 2.67 (t, *J* = 7.5 Hz, 2 H), 1.94 (p, *J* = 7.1 Hz, 2 H); **^{13}C NMR** (126 MHz, $CDCl_3$) δ (ppm) 166.2, 156.6, 154.0, 136.2, 128.6 (2 C), 128.3, 128.2 (2 C), 67.0, 39.7, 24.9, 22.2; **HRMS** (m/z): $[M+H]^+$ calcd. for $C_{13}H_{14}N_2O_5$, 279.0981; found, 279.0971.

***tert*-Butyl (3-(5-oxo-1,4,2-dioxazol-3-yl)propyl)carbamate (9c)**

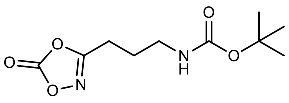

Stepwise procedure.
<u>Step 1:</u> In an oven-dried round bottom flask, 1,1'-carbonyl diimidazole (CDI, 1.22 g, 7.50 mmol, 1.5 equiv) was added to a solution of 4-((*tert*-butoxycarbonyl)amino)butanoic acid (1.02 g, 5.00 mmol) in dry MeCN (0.5 M, 10 mL). After 2 hours of stirring at room temperature under a N_2 atmosphere, hydroxylamine hydrochloride (695 mg, 10.0 mmol, 2.0 equiv) was added and the resulting mixture was stirred overnight at room temperature. Subsequently, the reaction mixture was diluted with 5% aq. $KHSO_4$ (20 mL) and extracted with EtOAc (3 x 40 mL). The combined organic layer was washed with brine (80 mL), dried over $MgSO_4$ and concentrated in vacuo to afford the corresponding hydroxamic acid as a colourless oil. This was used in the following step without further purification.
<u>Step 2:</u> The crude hydroxamic acid was dissolved in dry CH_2Cl_2 (0.1 M, 45 mL), and 1,1'-carbonyl diimidazole (CDI, 1.14 g, 7.06 mmol, 1.5 equiv) was added in one portion. The reaction mixture was stirred at room temperature for 30 min, then diluted with aq. HCl (1 N, 25 mL) and CH_2Cl_2 (20 mL). The phases were separated and the aqueous layer was extracted with CH_2Cl_2 (3 x 40 mL). The combined organic layer was washed with brine (80 mL), dried over anhydrous $MgSO_4$, filtered, and concentrated under reduced pressure. The resulting residue was purified by filtration over a short SiO_2 plug eluting with CH_2Cl_2 to give dioxazolone **9c** (734 mg, 60% over 2 steps) as a colourless oil.
^{1}H NMR (500 MHz, $CDCl_3$) δ (ppm) 4.63 (br s, 1 H), 3.24 (t, J = 6.6 Hz, 2 H), 2.69 (t, J = 7.5 Hz, 2 H), 1.92 (p, J = 7.0 Hz, 2 H), 1.44 (s, 9 H); **^{13}C NMR** (126 MHz, $CDCl_3$) δ (ppm) 166.3, 156.0, 154.0, 79.8, 39.3, 28.3 (3 C), 25.1, 22.3; **HRMS** (m/z): No identifiable peak detected.

***tert*-Butyl 3-(5-oxo-1,4,2-dioxazol-3-yl)azetidine-1-carboxylate (9d)**

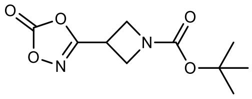

Stepwise procedure.
<u>Step 1:</u> In an oven-dried screw-cap vial, 1,1'-carbonyl diimidazole (CDI, 1.00 g, 6.17 mmol, 1.5 equiv) was added to a solution of 1-(*tert*-butoxycarbonyl)azetidine-3-carboxylic acid (827 mg, 4.11 mmol) in dry MeCN (0.5 M, 8.0 mL). After 2 hours of stirring at room temperature under a N_2 atmosphere, hydroxylamine hydrochloride (571 mg, 8.22 mmol, 2.0 equiv) was added and the resulting mixture was stirred overnight at room temperature. Subsequently, the reaction mixture was diluted with 5% aq. $KHSO_4$ (20 mL) and extracted with EtOAc (3 x 40 mL). The combined organic layer was washed with brine (80 mL), dried over $MgSO_4$ and concentrated in vacuo to afford the corresponding hydroxamic acid as a white solid. This was used in the following step without further purification.
<u>Step 2:</u> The crude hydroxamic acid was dissolved in dry EtOAc (0.1 M, 35 mL), and 1,1'-carbonyl diimidazole (CDI, 877 mg, 5.41 mmol, 1.5 equiv) was added in one portion. The reaction mixture was stirred at room temperature for 30 min, then diluted with aq. HCl (1 N, 30 mL) and EtOAc (20 mL). The phases were separated and the aqueous layer was extracted with EtOAc (3 x 40 mL). The combined organic layer was washed with brine (80 mL), dried over anhydrous $MgSO_4$, filtered, and concentrated under reduced pressure. The resulting residue was purified by filtration over a short SiO_2 plug eluting with CH_2Cl_2 to give dioxazolone **9d** (555 mg, 56% over 2 steps) as a white solid.
^{1}H NMR (500 MHz, $CDCl_3$) δ (ppm) 4.28 (t, J = 8.9 Hz, 2 H), 4.17 (dd, J = 9.0, 5.9 Hz, 2 H), 3.74 (tt, J = 8.9, 5.9 Hz, 1 H), 1.45 (s, 9 H); **^{13}C NMR** (126 MHz, $CDCl_3$) δ (ppm) 165.8, 155.6, 153.6, 80.7, 51.0 (br, 2 C), 28.3 (3 C), 24.2.

The spectroscopic data was consistent with what reported in the literature.[21]

***tert*-Butyl 4-((5-oxo-1,4,2-dioxazol-3-yl)methyl)piperidine-1-carboxylate (9e)**

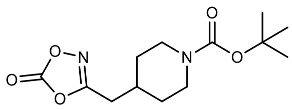

Stepwise procedure.
<u>Step 1:</u> In an oven-dried screw-cap vial, 1,1'-carbonyl diimidazole (CDI, 1.00 g, 6.17 mmol, 1.5 equiv) was added to a solution of 2-(1-(*tert*-butoxycarbonyl)piperidin-4-yl)acetic acid (1.0 g, 4.11 mmol) in dry MeCN (0.5 M, 8.0 mL). After 2 hours of stirring at room temperature under a N_2 atmosphere, hydroxylamine hydrochloride (571 mg, 8.22 mmol, 2.0 equiv) was added and the resulting mixture was stirred overnight at room temperature. Subsequently, the reaction mixture was diluted with 5% aq. $KHSO_4$ (20 mL) and extracted with EtOAc (3 x 40 mL). The combined organic layer was washed with brine (80 mL), dried over $MgSO_4$ and concentrated in vacuo to afford the corresponding hydroxamic acid as a white solid. This was used in the following step without further purification.
<u>Step 2:</u> The crude hydroxamic acid was dissolved in dry EtOAc (0.1 M, 40 mL), and 1,1'-carbonyl diimidazole (CDI, 951 mg, 5.86 mmol, 1.5 equiv) was added in one portion. The reaction mixture was stirred at room temperature for 30 min, then diluted with aq. HCl (1 N, 30 mL) and EtOAc (20 mL). The phases were separated and the aqueous layer was extracted with EtOAc (3 x 50 mL). The combined organic layer was washed with brine (80 mL), dried over anhydrous $MgSO_4$, filtered, and concentrated under reduced pressure. The resulting residue was purified by filtration over a short SiO_2 plug eluting with CH_2Cl_2 to give dioxazolone **9e** (898 mg, 77% over 2 steps) as a white solid.
^{1}H NMR (500 MHz, $CDCl_3$) δ (ppm) 4.13 (br d, *J* = 12.6 Hz, 2 H), 2.72 (br t, *J* = 12.9 Hz, 2 H), 2.57 (d, *J* = 7.0 Hz, 2 H), 1.93 (ttt, *J* = 11.0, 7.2, 3.7 Hz, 1 H), 1.74 (br d, *J* = 13.1 Hz, 2 H), 1.45 (s, 9 H), 1.25 (qd, *J* = 12.7, 4.4 Hz, 2 H); **^{13}C NMR** (126 MHz, $CDCl_3$) δ (ppm) 165.2, 154.6, 154.0, 79.7, 43.4 (br, 2 C), 33.0, 31.6 (2 C), 31.4, 28.4 (3 C); **HRMS** (m/z): No identifiable peak detected.

***tert*-Butyl 3-(5-oxo-1,4,2-dioxazol-3-yl)propanoate (9f)**

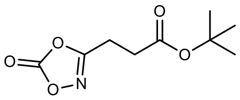

Stepwise procedure.
<u>Step 1:</u> In an oven-dried round bottom flask, 1,1'-carbonyl diimidazole (CDI, 1.22 g, 7.50 mmol, 1.5 equiv) was added to a solution of 4-(*tert*-butoxy)-4-oxobutanoic acid (871 mg, 5.00 mmol) in dry MeCN (0.5 M, 10 mL). After 2 hours of stirring at room temperature under a N_2 atmosphere, hydroxylamine hydrochloride (695 mg, 10.0 mmol, 2.0 equiv) was added and the resulting mixture was stirred overnight at room temperature. Subsequently, the reaction mixture was diluted with 5% aq. $KHSO_4$ (20 mL) and extracted with EtOAc (3 x 40 mL). The combined organic layer was washed with brine (80 mL), dried over $MgSO_4$ and concentrated in vacuo to afford the corresponding hydroxamic acid as a colourless oil. This was used in the following step without further purification.
<u>Step 2:</u> The crude hydroxamic acid was dissolved in dry CH_2Cl_2 (0.1 M, 45 mL), and 1,1'-carbonyl diimidazole (CDI, 1.14 g, 7.06 mmol, 1.5 equiv) was added in one portion. The reaction mixture was stirred at room temperature for 30 min, then diluted with aq. HCl (1 N, 25 mL) and CH_2Cl_2 (20 mL). The phases were separated and the aqueous layer was extracted with CH_2Cl_2 (3 x 40 mL). The combined organic layer was washed with brine (80 mL), dried over anhydrous $MgSO_4$, filtered, and concentrated under reduced pressure. The resulting residue was purified by filtration over a short SiO_2 plug eluting with CH_2Cl_2 to give dioxazolone **9f** (792 mg, 74% over 2 steps) as a colourless oil.
^{1}H NMR (500 MHz, $CDCl_3$) δ (ppm) 2.91 (t, *J* = 7.1 Hz, 2 H), 2.67 (t, *J* = 7.2 Hz, 2 H), 1.46 (s, 9 H); **^{13}C NMR** (126 MHz, $CDCl_3$) δ (ppm) 169.5, 165.7, 150.8, 82.0, 29.5, 28.0 (3 C), 20.6; **HRMS** (m/z): No identifiable peak detected.

tert-Butyl 2-(3-(5-oxo-1,4,2-dioxazol-3-yl)bicyclo[1.1.1]pentan-1-yl)acetate (9g)

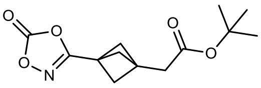

Stepwise procedure.
<u>Step 1:</u> In an oven-dried screw-cap vial, 1,1'-carbonyl diimidazole (CDI, 486 mg, 3.00 mmol, 1.5 equiv) was added to a solution of 3-(2-(_tert_-butoxy)-2-oxoethyl)bicyclo[1.1.1]pentane-1-carboxylic acid (453 mg, 2.00 mmol) in dry MeCN (0.5 M, 4.0 mL). After 2 hours of stirring at room temperature under a N_2 atmosphere, hydroxylamine hydrochloride (571 mg, 8.22 mmol, 2.0 equiv) was added and the resulting mixture was stirred overnight at room temperature. Subsequently, the reaction mixture was diluted with 5% aq. $KHSO_4$ (20 mL) and extracted with EtOAc (3 x 40 mL). The combined organic layer was washed with brine (80 mL), dried over $MgSO_4$ and concentrated in vacuo to afford the corresponding hydroxamic acid as a white solid. This was used in the following step without further purification.
<u>Step 2:</u> The crude hydroxamic acid was dissolved in dry CH_2Cl_2 (0.1 M, 20 mL), and 1,1'-carbonyl diimidazole (CDI, 482 mg, 2.97 mmol, 1.5 equiv) was added in one portion. The reaction mixture was stirred at room temperature for 30 min, then diluted with aq. HCl (1 N, 15 mL) and CH_2Cl_2 (10 mL). The phases were separated and the aqueous layer was extracted with CH_2Cl_2 (3 x 20 mL). The combined organic layer was washed with brine (40 mL), dried over anhydrous $MgSO_4$, filtered, and concentrated under reduced pressure to afford dioxazolone **9g** (504 mg, 94% over 2 steps) as a white solid.
^{1}H NMR (500 MHz, $CDCl_3$) δ (ppm) 2.50 (s, 2 H), 2.23 (s, 6 H), 1.46 (s, 9 H); **^{13}C NMR** (126 MHz, $CDCl_3$) δ (ppm) 169.6, 163.2, 154.1, 81.1, 52.9 (3 C), 39.1, 38.0, 31.2, 28.17 (3 C); **HRMS** (m/z): No identifiable peak detected.

3-(2-(1,3-Dioxolan-2-yl)ethyl)-1,4,2-dioxazol-5-one (9h)

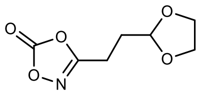

Telescoped procedure.
In an oven-dried screw-cap vial, 1,1'-carbonyl diimidazole (CDI, 1.66 g, 10.3 mmol, 1.5 equiv) was added to a solution of 3-(1,3-dioxolan-2-yl)propanoic acid (1.00 g, 6.84 mmol) in dry MeCN (0.5 M, 13 mL). After 2 hours of stirring at room temperature under a N_2 atmosphere, hydroxylamine hydrochloride (951 mg, 13.7 mmol, 2.0 equiv) was added and the resulting mixture was stirred overnight at room temperature. The solvent was removed under reduced pressure, and the residue was resolubilized in CH_2Cl_2 (0.1 M, 60 ml). 1,1'-Carbonyl diimidazole (CDI, 1.66 g, 10.3 mmol, 1.5 equiv) was added in one portion. The reaction mixture was stirred at room temperature for 30 min, then diluted with aq. HCl (0.01 N, pH ~ 2, 60 mL) and CH_2Cl_2 (50 mL). The phases were separated and the organic layer was washed with further aq. HCl (0.01 N, pH ~ 2, 50 mL) and brine (50 mL), dried over anhydrous $MgSO_4$, filtered, and concentrated under reduced pressure. The resulting residue was purified by filtration over a short SiO_2 plug eluting with CH_2Cl_2 to give dioxazolone **9h** (582 mg, 45% over 2 steps) as a colourless oil.
^{1}H NMR (500 MHz, $CDCl_3$) δ (ppm) 5.00 (t, J = 3.5 Hz, 1 H), 4.02–3.92 (m, 2 H), 3.92–3.83 (m, 2 H), 2.76 (t, J = 7.3 Hz, 2 H), 2.12 (td, J = 7.3, 3.5 Hz, 2 H); **^{13}C NMR** (126 MHz, $CDCl_3$) δ (ppm) 166.6, 154.2, 101.9, 65.3 (2 C), 27.9, 18.6; **HRMS** (m/z): No identifiable peak detected.

3-(But-3-en-1-yl)-1,4,2-dioxazol-5-one (9i)

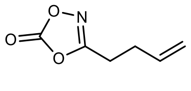

Step-wise procedure.

Step 1: In an oven-dried screw-cap vial, 1,1'-carbonyl diimidazole (CDI, 1.22 g, 7.50 mmol, 1.5 equiv) was added to a solution of pent-4-enoic acid (501 mg, 5.00 mmol) in dry MeCN (0.5 M, 10 mL). After 2 hours of stirring at room temperature under a N_2 atmosphere, hydroxylamine hydrochloride (695 mg, 10.0 mmol, 2.0 equiv) was added and the resulting mixture was stirred overnight at room temperature. Subsequently, the reaction mixture was diluted with aq. HCl (2 N, 10 mL) and extracted with EtOAc (3 x 20 mL). The combined organic layer was washed with brine (50 mL), dried over $MgSO_4$ and concentrated in vacuo to afford the corresponding hydroxamic acid as a colourless oil. This was used in the following step without further purification.
Step 2: The crude hydroxamic acid was dissolved in dry CH_2Cl_2 (0.1 M, 17 mL), and 1,1'-carbonyl diimidazole (CDI, 401 mg, 2.48 mmol, 1.5 equiv) was added in one portion. The reaction mixture was stirred at room temperature for 30 min, then diluted with aq. HCl (1 N, 30 mL) and CH_2Cl_2 (20 mL). The phases were separated and the aqueous layer was extracted with CH_2Cl_2 (3 x 40 mL). The combined organic layer was washed with brine (80 mL), dried over anhydrous $MgSO_4$, filtered, and concentrated under reduced pressure to afford dioxazolone **9i** (183 mg, 26% over 2 steps) as a colourless oil.
^{1}H NMR (500 MHz, $CDCl_3$) δ (ppm) 5.80 (ddt, *J* = 16.9, 10.2, 6.5 Hz, 1 H), 5.20–5.07 (m, 2 H), 2.74 (t, *J* = 7.4 Hz, 2 H), 2.48 (td, *J* = 7.4, 6.3 Hz, 2 H); **^{13}C NMR** (126 MHz, $CDCl_3$) δ (ppm) 166.1, 154.1, 134.2, 117.6, 28.3, 24.3.

The spectroscopic data was consistent with what reported in the literature.[22]

[*Note: handle with care under reduced pressure. Both dioxazolone **9i** and the hydroxamic acid precursor were highly volatile.*]

3-(4-((3a*S*,4*S*,6a*R*)-2-Oxohexahydro-1*H*-thieno[3,4-*d*]imidazol-4-yl)butyl)-1,4,2-dioxazol-5-one (17)

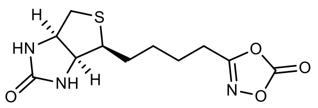

One-pot procedure.
In an oven-dried round bottom flask, 1,1'-carbonyl diimidazole (CDI, 6.08 mg, 3.75 mmol, 1.5 equiv) was added to a solution of 5-((3a*S*,4*S*,6a*R*)-2-oxohexahydro-1*H*-thieno[3,4-*d*]imidazol-4-yl)pentanoic acid (611 mg, 2.50 mmol) in dry DMF (0.1 M, 25 mL). After 2 hours of stirring at room temperature under a N_2 atmosphere, hydroxylamine hydrochloride (347 mg, 5.00 mmol, 2.0 equiv) was added and the resulting mixture was stirred overnight at room temperature. 1,1'-Carbonyl diimidazole (CDI, 811 mg, 5.00 mmol, 2.0 equiv) was then added in one portion. The reaction mixture was stirred at room temperature for 30 min, then diluted with aq. HCl (1 N, 20 mL) and EtOAc (100 mL). The phases were separated and the aqueous layer was extracted with EtOAc (3 x 60 mL). The combined organic layer was washed with brine (2 x 100 mL), dried over anhydrous $MgSO_4$, filtered, and concentrated under reduced pressure to afford dioxazolone **17** (408 mg, 57% over 2 steps) as a white solid.
^{1}H NMR (500 MHz, DMSO-*d*$_6$) δ (ppm) 6.46 (s, 1 H), 6.38 (s, 1 H), 4.31 (dd, *J* = 7.7, 5.1 Hz, 1 H), 4.14 (ddd, *J* = 7.7, 4.4, 1.9 Hz, 1 H), 3.11 (td, *J* = 7.7, 7.2, 4.4 Hz, 1 H), 2.83 (dd, *J* = 12.4, 5.1 Hz, 1 H), 2.70–2.61 (m, 2 H), 2.58 (d, *J* = 12.5 Hz, 1 H), 1.67–1.57 (m, 3 H), 1.54–1.36 (m, 3 H); **^{13}C NMR** (126 MHz, DMSO-*d*$_6$) δ (ppm) 166.7, 162.7, 154.4, 60.9, 59.2, 55.2, 39.9, 27.7, 27.5, 23.9, 23.8; **HRMS** (m/z): $[M+H]^+$ calcd. for $C_{11}H_{15}N_3O_4S$, 286.0862; found, 286.0826.

***tert*-Butyl (2-(2-(2-((5-Oxo-1,4,2-dioxazol-3-yl)methoxy)ethoxy)ethoxy)ethyl)carbamate (18)**

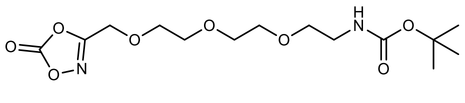

Stepwise procedure.

Step 1: In an oven-dried screw-cap vial, 1,1'-carbonyl diimidazole (CDI, 608 mg, 3.75 mmol, 1.5 equiv) was added to a solution of 2-dimethyl-4-oxo-3,8,11,14-tetraoxa-5-azahexadecan-16-oic acid (768 mg, 2.50 mmol) in dry MeCN (0.5 M, 5.0 mL). After 2 hours of stirring at room temperature under a N_2 atmosphere, hydroxylamine hydrochloride (347 mg, 5.00 mmol, 2.0 equiv) was added and the resulting mixture was stirred overnight at room temperature. Subsequently, the reaction mixture was diluted with 5% aq. $KHSO_4$ (20 mL) and extracted with EtOAc (3 x 40 mL). The combined organic layer was washed with brine (80 mL), dried over $MgSO_4$ and concentrated in vacuo to afford the corresponding hydroxamic acid as a white solid. This was used in the following step without further purification.

Step 2: The crude hydroxamic acid was dissolved in dry EtOAc (0.1 M, 18 mL), and 1,1'-carbonyl diimidazole (CDI, 420 mg, 2.59 mmol, 1.5 equiv) was added in one portion. The reaction mixture was stirred at room temperature for 30 min, then diluted with aq. HCl (1 N, 15 mL) and EtOAc (10 mL). The phases were separated and the aqueous layer was extracted with EtOAc (3 x 20 mL). The combined organic layer was washed with brine (40 mL), dried over anhydrous $MgSO_4$, filtered, and concentrated under reduced pressure to afford dioxazolone **18** (550 mg, 63% over 2 steps) as a colourless oil.

^{1}H NMR (500 MHz, $CDCl_3$) δ (ppm) 4.97 (br s, 1 H), 4.57 (s, 2 H), 3.80–3.75 (m, 2 H), 3.72–3.67 (m, 2 H), 3.67–3.57 (m, 4 H), 3.53 (t, J = 5.2 Hz, 2 H), 3.32 (q, J = 5.5 Hz, 2 H), 1.44 (s, 9 H); **^{13}C NMR** (126 MHz, $CDCl_3$) δ (ppm) 163.3, 156.0, 153.6, 79.3, 71.4, 70.7, 70.6, 70.3, 70.2, 62.1, 40.3, 28.4 (3 C); **HRMS** (m/z): No identifiable peak detected.

3-(4-Bromobutyl)-1,4,2-dioxazol-5-one (19)

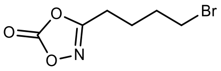

Stepwise procedure.

Step 1: In an oven-dried round bottom flask, 1,1'-carbonyl diimidazole (CDI, 1.22 g, 7.50 mmol, 1.5 equiv) was added to a solution of 5-bromopentanoic acid (905 mg, 5.00 mmol) in dry MeCN (0.5 M, 10 mL). After 2 hours of stirring at room temperature under a N_2 atmosphere, hydroxylamine hydrochloride (695 mg, 10.0 mmol, 2.0 equiv) was added and the resulting mixture was stirred overnight at room temperature. Subsequently, the reaction mixture was diluted with 5% aq. $KHSO_4$ (20 mL) and extracted with EtOAc (3 x 40 mL). The combined organic layer was washed with brine (80 mL), dried over $MgSO_4$ and concentrated in vacuo to afford the corresponding hydroxamic acid as a white solid. This was used in the following step without further purification.

Step 2: The crude hydroxamic acid was dissolved in dry CH_2Cl_2 (0.1 M, 35 mL), and 1,1'-carbonyl diimidazole (CDI, 819 mg, 5.05 mmol, 1.5 equiv) was added in one portion. The reaction mixture was stirred at room temperature for 30 min, then diluted with aq. HCl (1 N, 15 mL) and CH_2Cl_2 (15 mL). The phases were separated and the aqueous layer was extracted with CH_2Cl_2 (3 x 40 mL). The combined organic layer was washed with brine (40 mL), dried over anhydrous $MgSO_4$, filtered, and concentrated under reduced pressure to afford dioxazolone **19** (675 mg, 61% over 2 steps) as a colourless oil.

^{1}H NMR (500 MHz, $CDCl_3$) δ (ppm) 3.44 (t, J = 6.2 Hz, 2 H), 2.68 (t, J = 7.3 Hz, 2 H), 2.02–1.86 (m, 4 H); **^{13}C NMR** (126 MHz, $CDCl_3$) δ (ppm) 166.1, 154.0, 32.0, 31.3, 24.0, 23.0; **HRMS** (m/z): No identifiable peak detected.

3-(Dec-9-yn-1-yl)-1,4,2-dioxazol-5-one (20)

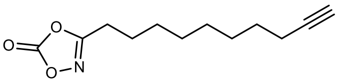

Stepwise procedure.

Step 1: In an oven-dried screw-cap vial, 1,1'-carbonyl diimidazole (CDI, 1.07 g, 6.58 mmol, 1.5 equiv) was added to a solution of undec-10-ynoic acid (800 mg, 4.39 mmol) in dry MeCN (0.5 M, 9.0 mL). After 2 hours of stirring at room temperature under a N_2 atmosphere, hydroxylamine hydrochloride

(610 mg, 6.78 mmol, 2.0 equiv) was added and the resulting mixture was stirred overnight at room temperature. Subsequently, the reaction mixture was diluted with 5% aq. $KHSO_4$ (10 mL) and extracted with EtOAc (3 x 20 mL). The combined organic layer was washed with brine (50 mL), dried over $MgSO_4$ and concentrated in vacuo to afford the corresponding hydroxamic acid as a white solid. This was used in the following step without further purification.
Step 2: The crude hydroxamic acid was dissolved in dry EtOAc (0.1 M, 40 mL), and 1,1'-carbonyl diimidazole (CDI, 1.04 g, 6.39 mmol, 1.5 equiv) was added in one portion. The reaction mixture was stirred at room temperature for 30 min, then diluted with aq. HCl (1 N, 30 mL) and EtOAc (20 mL). The phases were separated and the aqueous layer was extracted with EtOAc (3 x 50 mL). The combined organic layer was washed with brine (80 mL), dried over anhydrous $MgSO_4$, filtered, and concentrated under reduced pressure to afford dioxazolone **20** (949 mg, 98% over 2 steps) as a colourless oil.
^{1}H NMR (500 MHz, $CDCl_3$) δ (ppm) 2.62 (t, *J* = 7.6 Hz, 2 H), 2.18 (td, *J* = 7.1, 2.6 Hz, 2 H), 1.93 (t, *J* = 2.6 Hz, 1 H), 1.71 (p, *J* = 7.6 Hz, 2 H), 1.52 (p, *J* = 6.9 Hz, 2 H), 1.45–1.36 (m, 4 H), 1.36–1.27 (m, 4 H); **^{13}C NMR** (126 MHz, $CDCl_3$) δ (ppm) 166.7, 154.2, 84.6, 68.2, 28.80, 28.76, 28.6, 28.5, 28.3, 24.7, 24.5, 18.4; **HRMS** (m/z): No identifiable peak detected.

Ethyl 4-(5-oxo-1,4,2-dioxazol-3-yl)butanoate (9j)

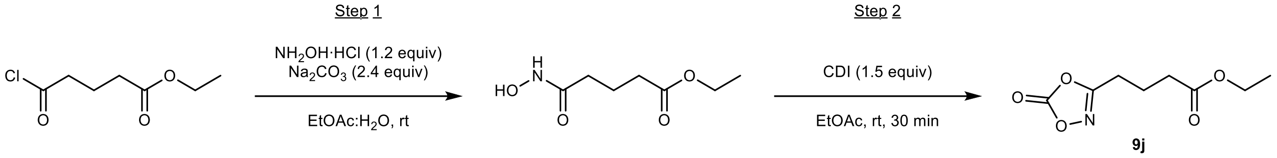

Stepwise procedure.
Step 1: In an oven-dried round bottom flask, ethyl 5-chloro-5-oxopentanoate (1.00 g, 5.60 mmol) was added dropwise to a mixture of hydroxylamine hydrochloride (467 mg, 6.72 mmol) and sodium carbonate (1.42 g, 13.4 mmol) in EtOAc:H_2O (1:1, 0.6 M, 10 mL) at 0 °C. The mixture was then allowed to warm to room temperature and stirred vigorously for 2 hours. The reaction was then diluted with aq. HCl (2 N, 15 mL), the phases were separated and the aqueous layer was extracted with EtOAc (3 x 20 mL). The combined organic layer was dried over $MgSO_4$ and concentrated under reduced pressure to afford the corresponding hydroxamic acid as a red oil. This was used in the following step without further purification.
Step 2: The crude hydroxamic acid was dissolved in dry EtOAc (0.1 M, 40 mL), and 1,1'-carbonyl diimidazole (CDI, 990 mg, 6.11 mmol, 1.5 equiv) was added in one portion. The reaction mixture was stirred at room temperature for 30 min, then diluted with aq. HCl (1 N, 40 mL) and EtOAc (20 mL). The phases were separated and the aqueous layer was extracted with EtOAc (3 x 50 mL). The combined organic layer was washed with brine (100 mL), dried over anhydrous $MgSO_4$, filtered, and concentrated under reduced pressure. The resulting residue was purified by filtration over a short SiO_2 plug eluting with CH_2Cl_2 to give dioxazolone **9j** (650 mg, 79% over 2 steps) as a pale red oil.
^{1}H NMR (500 MHz, $CDCl_3$) δ (ppm) 4.15 (q, *J* = 7.1 Hz, 2 H), 2.73 (t, *J* = 7.4 Hz, 2 H), 2.45 (t, *J* = 7.0 Hz, 2 H), 2.06 (p, *J* = 7.2 Hz, 2 H), 1.27 (t, *J* = 7.1 Hz, 3 H); **^{13}C NMR** (126 MHz, $CDCl_3$) δ (ppm) 172.0, 166.0, 154.0, 60.8, 32.5, 24.1, 19.6, 14.2; **HRMS** (m/z): No identifiable peak detected.

3-(3,3-Dimethoxypropyl)-1,4,2-dioxazol-5-one (21)

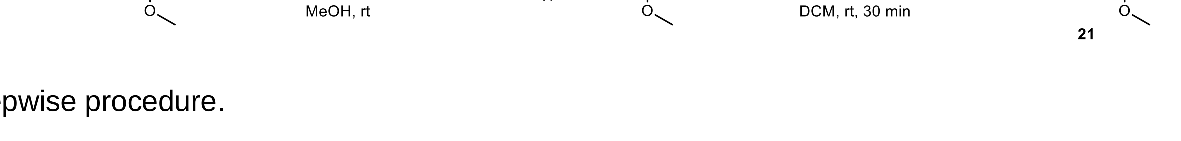

Stepwise procedure.

Step 1: In a screw-cap vial, methyl 4,4-dimethoxybutanoate (853 mg, 5.26 mmol) was stirred in MeOH (10 mL) with aq. hydroxylamine (50 wt.%, 3.5 mL, 57.1 mmol, 11 equiv) at room temperature overnight. The volatiles were then removed under reduced pressure to afford the corresponding hydroxamic acid as a colourless oil. This was used in the following step without further purification.
Step 2: The crude hydroxamic acid was dissolved in dry CH_2Cl_2 (0.1 M, 50 mL), and 1,1'-carbonyl diimidazole (CDI, 1.21 g, 7.44 mmol, 1.5 equiv) was added in one portion. The reaction mixture was stirred at room temperature for 30 min, then diluted with aq. HCl (0.01 N, pH ~ 2, 50 mL) and CH_2Cl_2 (50 mL). The phases were separated and the organic layer was washed with further aq. HCl (0.01 N, pH ~ 2, 50 mL) and brine (50 mL), dried over anhydrous $MgSO_4$, filtered, and concentrated under reduced pressure. The resulting residue was purified by filtration over a short SiO_2 plug eluting with CH_2Cl_2 to give dioxazolone **21** (673 mg, 70% over 2 steps) as a colourless oil.
^{1}H NMR (500 MHz, $CDCl_3$) δ (ppm) 4.43 (t, *J* = 5.2 Hz, 1 H), 3.35 (s, 6 H), 2.71 (t, *J* = 7.5 Hz, 2 H), 2.01 (td, *J* = 7.5, 5.2 Hz, 2 H); **^{13}C NMR** (126 MHz, $CDCl_3$) δ (ppm) 166.5, 154.1, 102.9, 53.8 (2 C), 27.5, 20.1; **HRMS** (m/z): No identifiable peak detected.

(±)-3-(2-(2-((2-(2,6-Dioxopiperidin-3-yl)-1,3-dioxoisoindolin-4-yl)amino)ethoxy)ethoxy)-*N*-hydroxypropanamide (22)

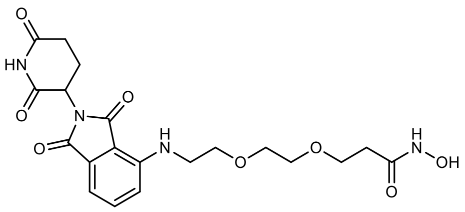

In an oven-dried screw-cap vial, 1,1'-carbonyl diimidazole (CDI, 281 mg, 1.73 mmol, 2.5 equiv) was added to a solution of (±)-3-(2-(2-((2-(2,6-dioxopiperidin-3-yl)-1,3-dioxoisoindolin-4-yl)amino)ethoxy)ethoxy)propanoic acid (300 mg, 0.69 mmol) in dry CH_2Cl_2 (0.3 M, 2.3 mL). After 2 hours of stirring at room temperature under a N_2 atmosphere, hydroxylamine hydrochloride (144 mg, 2.08 mmol, 3.0 equiv) was added and the resulting mixture was stirred overnight at room temperature. The volatiles were removed under reduced pressure and the resulting residue was dissolved in DMSO (5 mL), filtered and purified by preparative reverse phase HPLC (5-50% MeCN in HCO_2H buffer, 254 nm). The product containing fractions were combined and lyophilised to afford hydroxamic acid **22** (214 mg, 69%) as a yellow solid.
^{1}H NMR (500 MHz, CD_3OD) δ (ppm) 7.54 (t, *J* = 7.8 Hz, 1 H), 7.07 (d, *J* = 8.5 Hz, 1 H), 7.04 (d, *J* = 7.0 Hz, 1 H), 5.05 (dd, *J* = 12.6, 5.4 Hz, 1 H), 3.77–3.67 (m, 4 H), 3.67–3.58 (m, 4 H), 3.49 (t, *J* = 5.2 Hz, 2 H), 2.86 (ddd, *J* = 18.3, 13.9, 5.2 Hz, 1 H), 2.79–2.64 (m, 2 H), 2.34 (t, *J* = 6.1 Hz, 2 H), 2.17–2.06 (m, 1 H); **^{13}C NMR** (126 MHz, CD_3OD) δ (ppm) 174.7, 171.7, 170.9, 170.7, 169.3, 148.2, 137.2, 133.8, 118.2, 112.0, 111.3, 71.45, 71.35, 70.6, 67.9, 50.2, 43.2, 34.8, 32.2, 23.8; **HRMS** (m/z): $[M+H]^+$ calcd. for $C_{20}H_{24}N_4O_8$, 449.1672; found, 449.1641.

(±)-2-(2,6-Dioxopiperidin-3-yl)-4-((2-(2-(2-(5-oxo-1,4,2-dioxazol-3-yl)ethoxy)ethoxy)ethyl)amino)isoindoline-1,3-dione (13a)

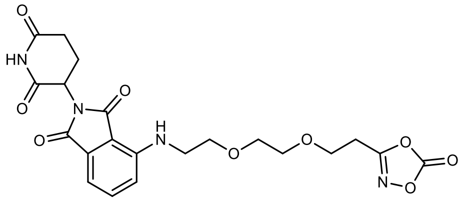

In an oven-dried screw-cap vial, 1,1'-carbonyl diimidazole (CDI, 124 mg, 0.77 mmol, 2.0 equiv) was added to a solution of hydroxamic acid **22** (172 mg, 0.38 mmol) in dry DMF (0.1 M, 4.0 mL). After stirring at room temperature for 30 min, the reaction was quenched with aq. HCl (1 N, 10 mL). The mixture was diluted with EtOAc (20 mL), the phases were separated, and the aqueous layer was extracted with EtOAc (3 x 20 mL). The combined organic extracts were washed with brine (2 x

60 mL), dried over anhydrous $MgSO_4$, filtered, and concentrated under reduced pressure (lyophilisation from $MeCN/H_2O$) to afford pure dioxazolone **13a** (173 mg, 95%) as a yellow solid. ^{1}**H NMR** (500 MHz, CD_3CN) δ (ppm) 8.95 (s, 1 H), 7.54 (dd, *J* = 8.6, 7.1 Hz, 1 H), 7.10–6.98 (m, 2 H), 6.48 (t, *J* = 5.6 Hz, 1 H), 4.93 (dd, *J* = 12.5, 5.3 Hz, 1 H), 3.75 (t, *J* = 6.1 Hz, 2 H), 3.67 (t, *J* = 5.3 Hz, 2 H), 3.60 (s, 4 H), 3.45 (q, *J* = 5.4 Hz, 2 H), 2.91–2.82 (m, 2 H), 2.82–2.61 (m, 3 H), 2.13–2.05 (m, 1 H); ^{13}**C NMR** (126 MHz, CD_3CN) δ (ppm) 173.0, 170.6, 170.5, 168.6, 166.6, 155.6, 147.8, 137.1, 133.5, 118.1, 111.8, 110.9, 71.1, 71.0, 70.0, 65.9, 49.9, 42.9, 32.0, 26.7, 23.3; **HRMS** (m/z): $[M+H]^+$ calcd. for $C_{21}H_{22}N_4O_9$, 475.1465; found, 475.1429.

(±)-6-((2-(2,6-Dioxopiperidin-3-yl)-1,3-dioxoisoindolin-5-yl)oxy)-*N*-hydroxyhexanamide (23)

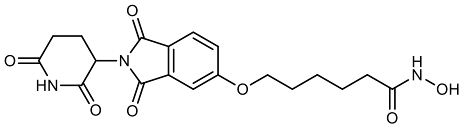

In an oven-dried screw-cap vial, 1,1'-carbonyl diimidazole (CDI, 167 mg, 1.03 mmol, 2.0 equiv) was added to a solution of (±)-6-((2-(2,6-dioxopiperidin-3-yl)-1,3-dioxoisoindolin-5-yl)oxy)hexanoic acid (200 mg, 0.51 mmol) in dry DMF (0.5 M, 1.0 mL). After 2 hours of stirring at room temperature under a N_2 atmosphere, hydroxylamine hydrochloride (107 mg, 1.54 mmol, 3.0 equiv) was added and the resulting mixture was stirred overnight at room temperature. The volatiles were removed under reduced pressure and the resulting residue was dissolved in DMSO (3 mL), filtered and purified by preparative reverse phase HPLC (5-45% MeCN in HCO_2H buffer, 254 nm). The product containing fractions were combined and lyophilised to afford hydroxamic acid **23** (122 mg, 59%) as a white solid.

^{1}**H NMR** (500 MHz, DMSO-*d*$_6$) δ (ppm) 11.12 (s, 1 H), 10.36 (s, 1 H), 8.68 (s, 1 H), 7.83 (d, *J* = 8.2 Hz, 1 H), 7.42 (d, *J* = 2.2 Hz, 1 H), 7.34 (dd, *J* = 8.3, 2.3 Hz, 1 H), 5.12 (dd, *J* = 12.9, 5.4 Hz, 1 H), 4.16 (t, *J* = 6.4 Hz, 2 H), 2.89 (ddd, *J* = 16.8, 13.8, 5.4 Hz, 1 H), 2.64–2.50 (m, part. overlap with solvent signal, 2 H), 2.09–2.00 (m, 1 H), 1.97 (t, *J* = 7.3 Hz, 2 H), 1.75 (p, *J* = 6.7 Hz, 2 H), 1.56 (p, *J* = 7.5 Hz, 2 H), 1.45–1.33 (m, 2 H); ^{13}**C NMR** (126 MHz, DMSO-*d*$_6$) δ (ppm) 172.8, 170.0, 169.0, 166.93, 166.86, 164.1, 134.0, 125.3, 122.9, 120.8, 108.9, 68.7, 49.0, 32.2, 31.0, 28.1, 25.0, 24.8, 22.1; **HRMS** (m/z): $[M+H]^+$ calcd. for $C_{19}H_{21}N_3O_7$, 404.1458; found, 404.1451.

(±)-2-(2,6-Dioxopiperidin-3-yl)-5-((5-(5-oxo-1,4,2-dioxazol-3-yl)pentyl)oxy)isoindoline-1,3-dione (13b)

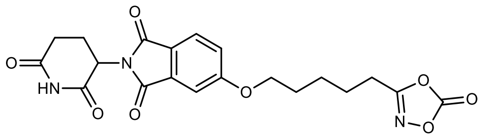

In an oven-dried screw-cap vial, 1,1'-carbonyl diimidazole (CDI, 88.2 mg, 0.55 mmol, 2.0 equiv) was added to a solution of hydroxamic acid **23** (110 mg, 0.27 mmol) in dry DMF (0.1 M, 2.7 mL). After stirring at room temperature for 30 min, the reaction was quenched with aq. HCl (1 N, 10 mL). The mixture was diluted with EtOAc (20 mL), the phases were separated, and the aqueous layer was extracted with EtOAc (3 x 20 mL). The combined organic extracts were washed with brine (2 x 60 mL), dried over anhydrous $MgSO_4$, filtered, and concentrated under reduced pressure (lyophilisation from $MeCN/H_2O$) to afford pure dioxazolone **13b** (110 mg, 94%) as a white solid. ^{1}**H NMR** (500 MHz, $CDCl_3$) δ (ppm) 8.03 (br s, 1 H), 7.79 (d, *J* = 8.3 Hz, 1 H), 7.33 (d, *J* = 2.3 Hz, 1 H), 7.18 (dd, *J* = 8.3, 2.3 Hz, 1 H), 4.96 (dd, *J* = 12.4, 5.3 Hz, 1 H), 4.10 (t, *J* = 6.2 Hz, 2 H), 2.98–2.71 (m, 3 H), 2.69 (t, *J* = 7.5 Hz, 2 H), 2.20–2.10 (m, 1 H), 1.94–1.87 (m, 2 H), 1.83 (p, *J* = 7.6 Hz, 2 H), 1.72–1.53 (m, overlap with H_2O signal, 2 H); ^{13}**C NMR** (126 MHz, $CDCl_3$) δ (ppm) 170.8, 168.0, 167.2, 167.0, 166.3, 164.3, 154.1, 134.4, 125.6, 123.6, 120.8, 108.8, 68.4, 49.3, 31.4, 28.4, 25.3, 24.7, 24.2, 22.7; **HRMS** (m/z): $[M+H]^+$ calcd. for $C_{20}H_{19}N_3O_8$, 430.1250; found, 430.1236.

(±)-8-(4-(2-(2,6-Dioxopiperidin-3-yl)-1,3-dioxoisoindolin-5-yl)piperazin-1-yl)-*N*-hydroxy-8-oxooctanamide (24)

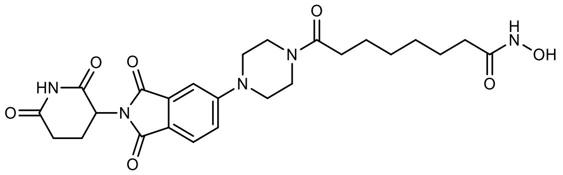

In an oven-dried screw-cap vial, 1,1'-carbonyl diimidazole (CDI, 265 mg, 1.63 mmol, 2.5 equiv) was added to a solution of (±)-8-(4-(2-(2,6-dioxopiperidin-3-yl)-1,3-dioxoisoindolin-5-yl)piperazin-1-yl)-8-oxooctanoic acid – TFA salt (400 mg, 0.65 mmol) in dry CH_2Cl_2 (0.3 M, 2.0 mL). After 2 hours of stirring at room temperature under a N_2 atmosphere, hydroxylamine hydrochloride (136 mg, 1.96 mmol, 3.0 equiv) was added and the resulting mixture was stirred overnight at room temperature. The volatiles were removed under reduced pressure and the resulting residue was dissolved in DMSO (5 mL), filtered and purified by preparative reverse phase HPLC (5-45% MeCN in HCO_2H buffer, 254 nm). The product containing fractions were combined and lyophilised to afford hydroxamic acid **24** (295 mg, 88%) as a yellow solid.

^{1}H NMR (500 MHz, CD_3OD) δ (ppm) 7.66 (d, *J* = 8.5 Hz, 1 H), 7.33 (d, *J* = 2.3 Hz, 1 H), 7.20 (dd, *J* = 8.6, 2.4 Hz, 1 H), 5.07 (dd, *J* = 12.5, 5.5 Hz, 1 H), 3.79–3.69 (m, 4 H), 3.54–3.49 (m, 2 H), 3.49–3.44 (m, 2 H), 2.92–2.81 (m, 1 H), 2.78–2.65 (m, 2 H), 2.44 (t, *J* = 7.6 Hz, 2 H), 2.16–2.06 (m, 3 H), 1.68–1.58 (m, 4 H), 1.44–1.31 (m, 4 H); **^{13}C NMR** (126 MHz, CD_3OD) δ (ppm) 174.7, 174.3, 172.9, 171.6, 169.3, 168.9, 156.7, 135.5, 126.0, 121.0, 119.3, 109.4, 50.4, 48.4, 48.0, 46.1, 42.3, 33.9, 33.7, 32.2, 30.0, 29.8, 26.6, 26.2, 23.8; **HRMS** (m/z): $[M+H]^+$ calcd. for $C_{25}H_{31}N_5O_7$, 514.2302; found: 514.2289.

(±)-2-(2,6-Dioxopiperidin-3-yl)-5-(4-(7-(5-oxo-1,4,2-dioxazol-3-yl)heptanoyl)piperazin-1-yl)isoindoline-1,3-dione (13c)

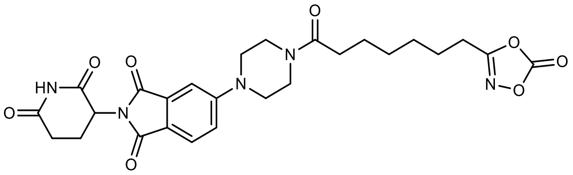

In an oven-dried screw-cap vial, 1,1'-carbonyl diimidazole (CDI, 152 mg, 0.93 mmol, 2.0 equiv) was added to a solution of hydroxamic acid **24** (240 mg, 0.47 mmol) in dry DMF (0.1 M, 5 mL). After stirring at room temperature for 30 min, the reaction was quenched with aq. HCl (1 N, 10 mL). The mixture was diluted with EtOAc (50 mL), the phases were separated, and the aqueous layer was extracted with EtOAc (3 x 20 mL). The combined organic extracts were washed with brine (2 x 60 mL), dried over anhydrous $MgSO_4$, filtered, and concentrated under reduced pressure (lyophilisation from $MeCN/H_2O$) to afford pure dioxazolone **13c** (239 mg, 95%) as a yellow solid. **^{1}H NMR** (500 MHz, DMSO-*d*$_6$) δ (ppm) 11.09 (s, 1 H), 7.70 (d, *J* = 8.5 Hz, 1 H), 7.34 (d, *J* = 2.3 Hz, 1 H), 7.24 (dd, *J* = 8.6, 2.4 Hz, 1 H), 5.08 (dd, *J* = 12.8, 5.4 Hz, 1 H), 3.66–3.56 (br m, 4 H), 3.55–3.48 (m, 2 H), 3.48–3.42 (m, 2 H), 2.88 (ddd, *J* = 16.6, 13.7, 5.2 Hz, 1 H), 2.65 (t, *J* = 7.4 Hz, 2 H), 2.62–2.51 (m, part. overlap with solvent signal, 2 H), 2.35 (t, *J* = 7.4 Hz, 2 H), 2.07–1.97 (m, 1 H), 1.60 (p, *J* = 7.5 Hz, 2 H), 1.51 (p, *J* = 7.5 Hz, 2 H), 1.42–1.25 (m, 4 H); **^{13}C NMR** (126 MHz, DMSO-*d*$_6$) δ (ppm) 172.8, 170.9, 170.1, 167.6, 167.0, 166.8, 154.9, 154.4, 133.9, 125.0, 118.5, 117.8, 107.9, 48.8, 46.8, 46.6, 44.1, 40.4, 32.1, 31.0, 28.2, 27.8, 24.4, 23.9, 23.6, 22.2; **HRMS** (m/z): $[M+H]^+$ calcd. for $C_{26}H_{29}N_5O_8$, 540.2094; found, 540.2104.

(±)-2-(3-(4-(2-(2,6-Dioxopiperidin-3-yl)-1-oxoisoindolin-5-yl)piperazine-1-carbonyl)bicyclo[1.1.1]pentan-1-yl)-*N*-hydroxyacetamide (25)

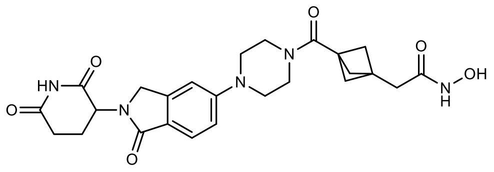

In an oven-dried screw-cap vial, 1,1'-carbonyl diimidazole (CDI, 219 mg, 1.35 mmol, 2.5 equiv) was added to a solution of (±)-2-(3-(4-(2-(2,6-dioxopiperidin-3-yl)-1-oxoisoindolin-5-yl)piperazine-1-carbonyl)bicyclo[1.1.1]pentan-1-yl)acetic acid (260 mg, 0.54 mmol) in dry CH_2Cl_2 (0.3 M, 1.7 mL). After 2 hours of stirring at room temperature under a N_2 atmosphere, hydroxylamine hydrochloride (113 mg, 1.62 mmol, 3.0 equiv) was added and the resulting mixture was stirred overnight at room temperature. The volatiles were removed under reduced pressure and the resulting residue was dissolved in DMSO (3 mL), filtered and purified by preparative reverse phase HPLC (5-55% MeCN in HCO_2H buffer, 254 nm). The product containing fractions were combined and lyophilised to afford hydroxamic acid **25** (221 mg, 83%) as a white solid.
^{1}H NMR (500 MHz, CD_3OD) δ (ppm) 7.65 (dd, *J* = 8.5, 1.7 Hz, 1 H), 7.13–7.05 (m, 2 H), 5.10 (ddd, *J* = 13.4, 5.2, 1.4 Hz, 1 H), 4.47–4.33 (m, 2 H), 3.89–3.80 (m, 2 H), 3.77–7.68 (m, 2 H), 3.41–3.33 (m, 4 H), 2.95–2.84 (m, 1 H), 2.82–2.72 (m, 1 H), 2.52–2.40 (m, 1 H), 2.31 (s, 2 H), 2.20–2.10 (s, 7 H); **^{13}C NMR** (126 MHz, CD_3OD) δ (ppm) 174.8, 172.4, 171.9, 170.1, 169.9, 155.7, 145.9, 125.4, 123.2, 116.7, 109.9, 54.6 (3 C), 53.5, 49.6, 46.4 (2 C), 43.1 (2 C), 40.9, 39.0, 36.4, 32.4, 24.2; **HRMS** (m/z): $[M+H]^+$ calcd. for $C_{25}H_{29}N_5O_6$, 496.2191; found, 496.2199.

(±)-3-(1-Oxo-5-(4-(3-((5-oxo-1,4,2-dioxazol-3-yl)methyl)bicyclo[1.1.1]pentane-1-carbonyl)piperazin-1-yl)isoindolin-2-yl)piperidine-2,6-dione (13d)

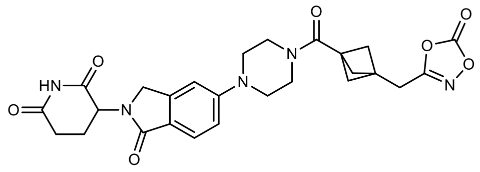

In an oven-dried screw-cap vial, 1,1'-carbonyl diimidazole (CDI, 130 mg, 0.80 mmol, 2.0 equiv) was added to a solution of hydroxamic acid **25** (198 mg, 0.40 mmol) in dry DMF (0.1 M, 4 mL). After stirring at room temperature for 30 min, the reaction was quenched with aq. HCl (1 N, 10 mL). The mixture was diluted with EtOAc (20 mL), the phases were separated, and the aqueous layer was extracted with EtOAc (3 x 20 mL). The combined organic extracts were washed with brine (2 x 60 mL), dried over anhydrous $MgSO_4$, filtered, and concentrated under reduced pressure (lyophilisation from MeCN/H_2O) to afford pure dioxazolone **13d** (161 mg, 77%) as a white solid. **^{1}H NMR** (500 MHz, DMSO-*d*$_6$) δ (ppm) 10.96 (s, 1 H), 7.54 (d, *J* = 8.2 Hz, 1 H), 7.11–7.02 (d, *J* = 8.5 Hz, 2 H), 5.05 (dd, *J* = 13.3, 5.1 Hz, 1 H), 4.34 (d, *J* = 16.9 Hz, 1 H), 4.21 (d, *J* = 16.9 Hz, 1 H), 3.76–3.68 (m, 2 H), 3.62–3.54 (m, 2 H), 3.32–3.24 (m, part. overlap with H_2O signal, 4 H), 2.97 (s, 2 H), 2.90 (ddd, *J* = 17.2, 13.7, 5.4 Hz, 1 H), 2.62–2.55 (m, 1 H), 2.43–2.31 (m, 1 H), 2.12 (s, 6 H), 2.01–1.91 (m, 1H); **^{13}C NMR** (126 MHz, DMSO-*d*$_6$) δ (ppm) 173.0, 171.3, 168.3, 166.5, 164.8, 154.2, 153.4, 144.1, 123.8, 121.9, 115.0, 108.7, 52.9 (3 C), 51.4, 47.9, 47.5, 47.0, 44.5, 41.1, 40.0, 36.0, 31.3, 26.7, 22.6; **HRMS** (m/z): $[M+H]^+$ calcd. for $C_{26}H_{27}N_5O_7$, 522.1983; found, 522.1990.

(±)-3-(1-(2-(2,6-Dioxopiperidin-3-yl)-1,3-dioxoisoindolin-4-yl)piperidin-4-yl)-*N*-hydroxypropanamide (26)

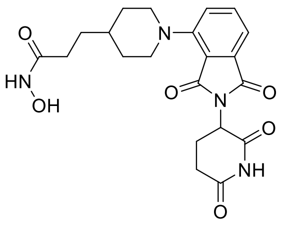

In an oven-dried screw-cap vial, 1,1'-carbonyl diimidazole (CDI, 235 mg, 1.45 mmol, 2.0 equiv) was added to a solution of (±)-3-(1-(2-(2,6-dioxopiperidin-3-yl)-1,3-dioxoisoindolin-4-yl)piperidin-4-yl)propanoic acid (300 mg, 0.73 mmol) in dry DMF (0.5 M, 1.5 mL). After 2 hours of stirring at room temperature under a N_2 atmosphere, hydroxylamine hydrochloride (151 mg, 2.18 mmol, 3.0 equiv) was added and the resulting mixture was stirred overnight at room temperature. The volatiles were removed under reduced pressure and the resulting residue was dissolved in DMSO (3 mL), filtered and purified by preparative reverse phase HPLC (20-60% MeCN in HCO_2H buffer, 254 nm). The product containing fractions were combined and lyophilised to afford hydroxamic acid **26** (245 mg, 79%) as a yellow solid.
^{1}H NMR (500 MHz, CD_3OD) δ (ppm) 7.61 (t, *J* = 7.3 Hz, 1 H), 7.35–7.21 (m, 2 H), 5.08 (dd, *J* = 12.5, 4.9 Hz, 1 H), 3.73 (d, *J* = 11.5 Hz, 2 H), 2.92–2.79 (br m, 3 H), 2.78–2.60 (m, 2 H), 2.17 (t, *J* = 7.4 Hz, 2 H), 2.14–2.06 (br m, 1 H), 1.92–1.72 (br m, 2 H), 1.62 (br s, 2 H), 1.45 (br s, 3 H); **^{13}C NMR** (126 MHz, CD_3OD) δ (ppm) 174.7, 173.0, 171.6, 169.1, 168.0, 152.1, 136.7, 135.4, 124.9, 118.4, 115.8, 52.9, 52.8, 50.4, 36.3, 33.4, 33.2 (2 C), 32.2, 31.1, 23.7; **HRMS** (m/z): $[M+H]^+$ calcd. for $C_{21}H_{24}N_4O_6$, 429.1774; found, 429.1773.

(±)-2-(2,6-Dioxopiperidin-3-yl)-4-(4-(2-(5-oxo-1,4,2-dioxazol-3-yl)ethyl)piperidin-1-yl)isoindoline-1,3-dione (13e)

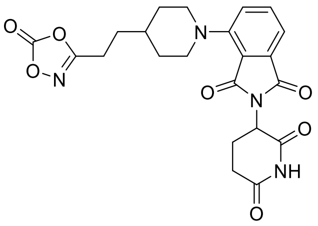

In an oven-dried screw-cap vial, 1,1'-carbonyl diimidazole (CDI, 150 mg, 0.92 mmol, 2.0 equiv) was added to a solution of hydroxamic acid **26** (198 mg, 0.46 mmol) in dry DMF (0.1 M, 4.5 mL). After stirring at room temperature for 30 min, the reaction was quenched with aq. HCl (1 N, 20 mL). The mixture was diluted with EtOAc (40 mL), the phases were separated, and the aqueous layer was extracted with EtOAc (3 x 40 mL). The combined organic extracts were washed with brine (2 x 100 mL), dried over anhydrous $MgSO_4$, filtered, and concentrated under reduced pressure (lyophilisation from MeCN/H_2O) to afford pure dioxazolone **13e** (200 mg, 95%) as a yellow solid. **^{1}H NMR** (600 MHz, DMSO-d_6) δ (ppm) 11.07 (s, 1 H), 7.67 (t, *J* = 7.8 Hz, 1 H), 7.37–7.29 (m, 2 H), 5.08 (dd, *J* = 12.9, 5.4 Hz, 1 H), 3.69 (d, *J* = 11.8 Hz, 2 H), 2.92–2.80 (m, 3 H), 2.74 (t, *J* = 7.5 Hz, 2 H), 2.59 (dt, *J* = 16.9, 3.1 Hz, 1 H), 2.56–2.50 (m, 1 H), 2.02 (ddt, *J* = 12.9, 7.8, 3.8 Hz, 1 H), 1.81 (dd, *J* = 13.1, 3.5 Hz, 2 H), 1.62 (q, *J* = 7.3 Hz, 2 H), 1.59–1.52 (m, 1 H), 1.43–1.31 (m, 2 H); **^{13}C NMR** (151 MHz, DMSO-d_6) δ (ppm) 172.8, 170.0, 167.1, 166.9, 166.3, 154.4, 150.1, 135.8, 133.7, 124.0, 116.4, 114.5, 51.0 (2 C), 48.8, 33.8, 31.3 (2 C), 31.0, 30.1, 22.1, 21.5; **HRMS** (m/z): $[M+H]^+$ calcd. for $C_{22}H_{22}N_4O_7$, 455.1566; found, 455.1566.

9.2 General experimental conditions for late-stage C–H amidation

General procedure A (main text Fig. 4-6)

On the benchtop, an oven-dried microwave vial was charged with the appropriate LSF substrate (0.25 mmol), dioxazolone reagent (0.25-0.28 mmol, 1.0-1.1 equiv), and pivalic acid (PivOH, 4.60 mg, 0.08 mmol, 30 mol%). The vial was moved into a glovebox under N_2 atmosphere, where [Ru(*p*-cymene)Cl_2]$_2$ (7.65 mg, 0.01 mmol, 5 mol%) and silver(I) hexafluorophosphate(V) ($AgPF_6$, 12.6 mg, 0.05 mmol, 20 mol%) were added sequentially. The vial was sealed and taken out of the glovebox. 2,2,2-Trifluoroethanol (TFE, 0.1 M, 2.5 mL) was then added by syringe under N_2 atmosphere, and the vial was heated to 60 °C. After stirring for 16 hours (1000 rpm), the reaction mixture was allowed to cool down to room temperature and analysed by LCMS. The solid material was removed by filtration through a plug of Celite, eluting with EtOAc or EtOAc and MeOH [*In cases of poor solubility of the product, this filtration step was not performed*]. After removal of the volatiles under reduced pressure, the crude material was either purified by automated flash column chromatography, or dissolved in DMSO (3-5 mL) and purified by preparative reverse phase HPLC. The relevant fractions were collected, combined and concentrated or lyophilised to afford the desired product.

[*Note*: *Although all the reactions were set up in glovebox under N_2 atmosphere, no significant drop in product yield was observed setting up the reaction under air with no exclusion of moisture*.]

General procedure B (main text Fig. 4-6)

On the benchtop, an oven-dried microwave vial was charged with the appropriate LSF substrate (0.25 mmol), dioxazolone reagent (0.25-0.28 mmol, 1.0-1.1 equiv), and diphenyl hydrogen phosphate ($(PhO)_2PO_2H$, 18.8 mg, 0.08 mmol, 30 mol%). The vial was moved into a glovebox under N_2 atmosphere, where [Ru(*p*-cymene)Cl_2]$_2$ (7.65 mg, 0.01 mmol, 5 mol%), silver(I) hexafluorostibate(V) ($AgSbF_6$, 17.2 mg, 0.05 mmol, 20 mol%) and 1,2-dimethoxhyethane (DME, 0.1 M, 2.5 mL) were added sequentially. The vial was sealed, taken out of the glovebox and heated to 60 °C. After stirring for 16 hours (1000 rpm), the reaction mixture was allowed to cool down to room temperature and analysed by LCMS. The solid material was removed by filtration through a plug of Celite, eluting with EtOAc or EtOAc and MeOH [*In cases of poor solubility of the product, this filtration step was not performed*]. After removal of the volatiles under reduced pressure, the crude material was either purified by automated flash column chromatography, or dissolved in DMSO (3-5 mL) and purified by preparative reverse phase HPLC. The relevant fractions were collected, combined and concentrated or lyophilised to afford the desired product.

[*Note*: *Although all the reactions were set up in glovebox under N_2 atmosphere, no significant drop in product yield was observed setting up the reaction under air with no exclusion of moisture*.]

9.3 Late-stage functionalisation (8a-8y, 27-28)

Benzyl 4-((2-(5-((4-aminophenyl)sulfonamido)-1*H*-pyrazol-1-yl)phenyl)carbamoyl)piperidine-1-carboxylate (8a)

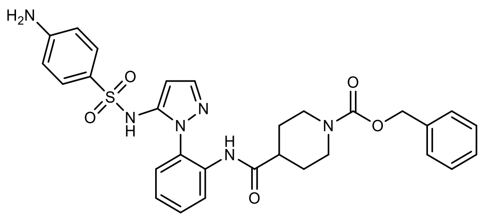

Prepared according to **General procedure A**, using 4-amino-*N*-(1-phenyl-1*H*-pyrazol-5-yl)benzenesulfonamide (*Sulfaphenazole*, 78.6 mg, 0.25 mmol) and benzyl 4-(5-oxo-1,4,2-dioxazol-3-yl)piperidine-1-carboxylate **4** (83.7 mg, 0.28 mmol) as substrates. The crude reaction mixture was analysed by LCMS using acidic mobile phase and the UV chromatogram is shown below:

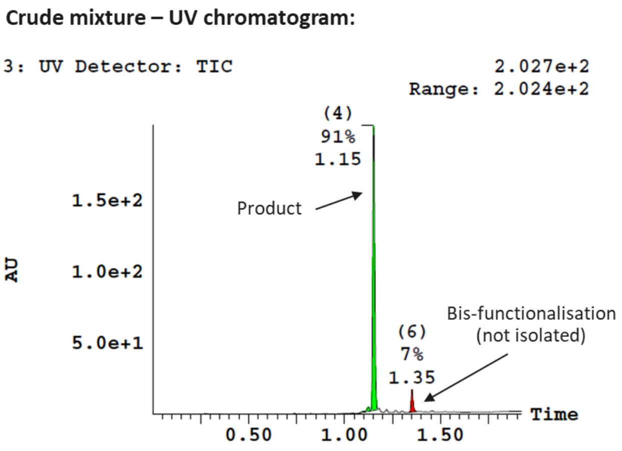

Supplementary Figure 20. LCMS UV chromatogram of the crude reaction mixture forming product **8a**.

Purification by automated flash column chromatography (15-100% EtOAc/heptane) afforded amidated derivative **8a** as a colourless oil (140 mg, 97%). R_f 0.20 (70% EtOAc/heptane); **^{1}H NMR** (500 MHz, $CDCl_3$) δ (ppm) 8.54 (s, 1 H), 8.07 (d, *J* = 8.1 Hz, 1 H), 7.58 (d, *J* = 2.1 Hz, 1 H), 7.42–7.29 (m, 8 H), 7.13 (m, 2 H), 6.97 (dd, *J* = 7.9, 1.5 Hz, 1 H), 6.52–6.44 (m, 2 H), 6.29 (d, *J* = 2.1 Hz, 1 H), 5.12 (s, 2 H), 4.19 (br s, 2 H), 4.09 (br d, *J* = 13.9 Hz, 2 H), 2.74 (br s, 2 H), 2.16 (tt, *J* = 11.5, 3.9 Hz, 1 H), 1.57 (br s, 2 H), 1.40 (br m, 2 H); **^{13}C NMR** (126 MHz, $CDCl_3$) δ (ppm) 172.6, 155.1, 151.5, 141.0, 136.71, 136.68, 134.0, 130.0, 129.5 (2 C), 128.6 (2 C), 128.2, 128.0 (2 C), 127.7, 126.5, 125.9, 125.0, 124.1, 113.9 (2 C), 100.3, 67.2, 43.4, 43.3 (2 C), 28.0 (2 C); **HRMS** (m/z): $[M+H]^+$ calcd. for $C_{29}H_{30}N_6O_5S$, 575.2076; found, 575.2104.

Benzyl 4-((5-sulfamoyl-2-(5-(*p*-tolyl)-3-(trifluoromethyl)-1*H*-pyrazol-1-yl)phenyl)carbamoyl)piperidine-1-carboxylate (8b)

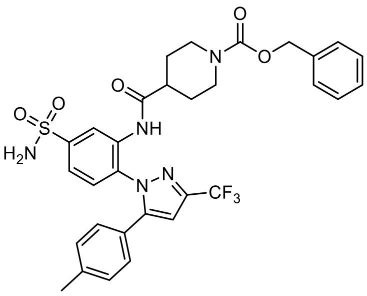

Prepared according to **General procedure A**, using 4-(5-(*p*-tolyl)-3-(trifluoromethyl)-1*H*-pyrazol-1-yl)benzenesulfonamide (*Celecoxib*, 95.3 mg, 0.25 mmol) and benzyl 4-(5-oxo-1,4,2-dioxazol-3-yl)piperidine-1-carboxylate **4** (83.7 mg, 0.28 mmol) as substrates. The crude reaction mixture was analysed by LCMS using acidic mobile phase and the UV chromatogram is shown below:

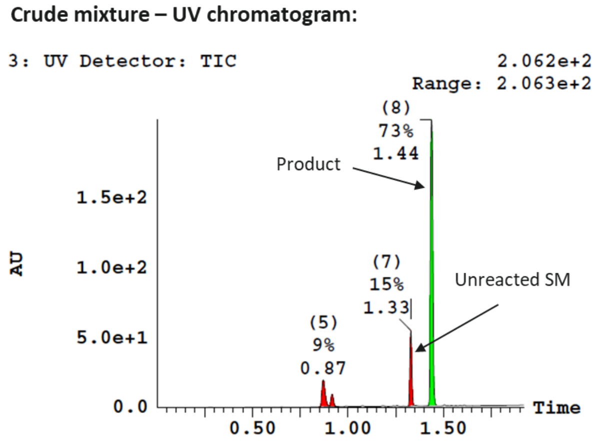

Supplementary Figure 21. LCMS UV chromatogram of the crude reaction mixture forming product **8b**.

Purification by automated flash column chromatography (10-100% EtOAc/heptane) afforded amidated derivative **8b** as a white solid (118 mg, 74%). R_f 0.10 (40% EtOAc/heptane); ^{1}**H NMR** (500 MHz, $CDCl_3$) δ (ppm) 9.21 (s, 1 H), 8.94 (s, 1 H), 7.48 (dd, J = 8.4, 2.1 Hz, 1 H), 7.39–7.27 (m, 5 H), 7.15 (d, J = 7.9 Hz, 2 H), 7.05 (d, J = 8.1 Hz, 2 H), 6.94 (d, J = 8.4 Hz, 1 H), 6.82 (s, 1 H), 5.49–5.37 (m, 2 H), 5.11 (s, 2 H), 4.20 (br s, 2 H), 2.85 (br s, 2 H), 2.44–2.30 (m, 4 H), 1.94–1.81 (br m, 2 H), 1.59 (qd, J = 12.4, 4.2 Hz, 2 H); ^{13}**C NMR** (126 MHz, $CDCl_3$) δ (ppm) 172.9, 155.1, 147.2, 144.5 (q, J_{CF} = 38.5 Hz), 143.0, 140.3, 136.7, 133.5, 130.9, 129.9 (2 C), 128.5 (4 C), 128.1, 127.9 (2 C), 127.5, 124.7, 121.7, 121.5, 120.7 (q, J_{CF} = 269.5 Hz), 106.0, 67.2, 43.9, 43.2 (2 C), 28.1 (2 C), 21.4; ^{19}**F NMR** (471 MHz, $CDCl_3$) δ (ppm) -61.4 (s, 3 F); **HRMS** (m/z): $[M+H]^+$ calcd. for $C_{31}H_{30}F_3N_5O_5S$, 642.1998; found, 642.1980.

Benzyl 4-(3-((4-(5-(*p*-tolyl)-3-(trifluoromethyl)-1*H*-pyrazol-1-yl)phenyl)sulfonyl)ureido)piperidine-1-carboxylate (27)

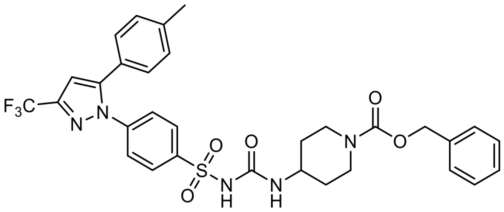

Prepared according to **General procedure B**, using 4-(5-(*p*-tolyl)-3-(trifluoromethyl)-1*H*-pyrazol-1-yl)benzenesulfonamide (*Celecoxib*, 95.3 mg, 0.25 mmol) and benzyl 4-(5-oxo-1,4,2-dioxazol-3-yl)piperidine-1-carboxylate **4** (83.7 mg, 0.28 mmol) as substrates. The crude reaction mixture was analysed by LCMS using acidic mobile phase and the UV chromatogram is shown below:

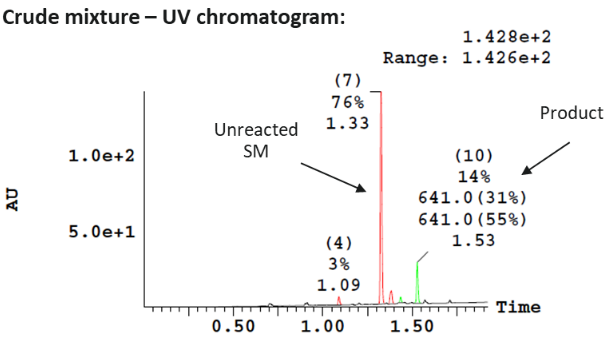

Supplementary Figure 22. LCMS UV chromatogram of the crude reaction mixture forming product **27**.

preparative reverse phase HPLC (25-65% MeCN in HCO_2H buffer, 254 nm) afforded sulfonyl urea derivative **27** as a white solid (21.1 mg, 13%). **^{1}H NMR** (500 MHz, DMSO-d_6) δ (ppm) 10.65 (s, 1 H), 7.96 (d, *J* = 8.7 Hz, 2 H), 7.57 (d, *J* = 8.7 Hz, 2 H), 7.41–7.27 (m, 5 H), 7.25–7.15 (m, 5 H), 6.61 (d, *J* = 7.6 Hz, 1 H), 5.05 (s, 2 H), 3.86 (dt, *J* = 13.5, 3.9 Hz, 2 H), 3.50 (dtd, *J* = 10.9, 7.0, 3.7 Hz, 1 H), 2.89 (br s, 2 H), 2.30 (s, 3 H), 1.71–1.62 (m, 2 H), 1.26 (qd, *J* = 11.5, 4.3 Hz, 2 H); **^{13}C NMR** (126 MHz, DMSO-d_6) δ (ppm) 154.3, 150.6, 145.4, 142.3 (q, *J* = 37.5 Hz), 142.1, 140.0, 139.2, 137.0, 129.4 (2 C), 128.8 (2 C), 128.6 (2 C), 128.4 (2 C), 127.8, 127.5 (2 C), 126.0 (2 C), 125.3, 121.3 (q, *J* = 268.8 Hz), 106.3, 66.2, 46.6, 42.3 (2 C), 31.1 (br, 2 C), 20.8; **^{19}F NMR** (471 MHz, DMSO-d_6) δ (ppm) -60.9 (s, 3 F); **HRMS** (m/z): $[M+H]^+$ calcd. for $C_{31}H_{30}F_3N_5O_5S$, 642.1998; found, 642.2007.

Benzyl 4-((2-(5-methyl-4-(4-(*N*-propionylsulfamoyl)phenyl)isoxazol-3-yl)phenyl)carbamoyl)piperidine-1-carboxylate (8c)

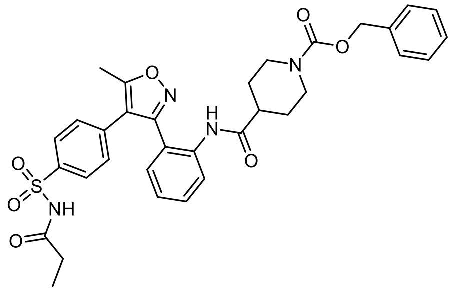

Prepared according to **General procedure A**, using *N*-((4-(5-methyl-3-phenylisoxazol-4-yl)phenyl)sulfonyl)propionamide (*Parecoxib*, 92.6 mg, 0.25 mmol) and benzyl 4-(5-oxo-1,4,2-dioxazol-3-yl)piperidine-1-carboxylate **4** (83.7 mg, 0.28 mmol) as substrates. The crude reaction mixture was analysed by LCMS using acidic mobile phase and the UV chromatogram is shown below:

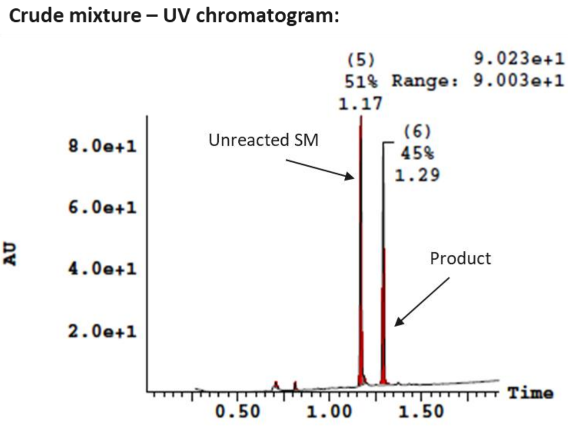

Supplementary Figure 23. LCMS UV chromatogram of the crude reaction mixture forming product **8c**.

Purification by automated flash column chromatography (10-100% EtOAc/heptane) afforded amidated derivative **8c** as a white solid (60.9 mg, 39%). R_f 0.26 (60% EtOAc/heptane); **^{1}H NMR** (500 MHz, $CDCl_3$) δ (ppm) 9.69 (s, 1 H), 8.76 (s, 1 H), 8.41 (d, *J* = 8.4 Hz, 1H), 8.01 (d, *J* = 8.5 Hz, 2 H), 7.40–7.28 (m, 8 H), 6.89 (t, *J* = 7.6 Hz, 1 H), 6.82 (d, *J* = 7.8 Hz, 1 H), 5.15 (s, 2 H), 4.26 (br s, 2 H), 2.92 (br s, 2 H), 2.53 (s, 3 H), 2.47 (tt, *J* = 11.4, 3.6 Hz, 1 H), 2.32 (q, *J* = 7.4 Hz, 2 H), 1.97 (br d, *J* = 13.1 Hz, 2 H), 1.75 (qd, *J* = 12.1, 4.3 Hz, 2 H), 1.10 (t, J = 7.4 Hz, 3 H); **^{13}C NMR** (126 MHz, $CDCl_3$) δ (ppm) 173.1, 171.6, 168.1, 159.4, 155.4, 138.2, 136.8, 136.5, 136.0, 130.8, 130.4, 130.3 (2 C), 129.0 (2 C), 128.7 (2 C), 128.2, 128.0 (2 C), 123.9, 122.7, 117.2, 115.3, 67.4, 44.4, 43.6 (2 C), 29.7, 28.5 (2 C), 12.0, 8.4; **HRMS** (m/z): $[M+H]^+$ calcd. for $C_{33}H_{34}N_4O_7S$, 631.2227; found, 631.2243.

Benzyl 4-((2-(3-(2-(dimethylamino)-2-oxoethyl)-6-methylimidazo[1,2-*a*]pyridin-2-yl)-5-methylphenyl)carbamoyl)piperidine-1-carboxylate (8d)

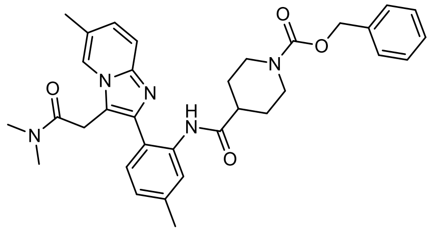

Prepared according to **General procedure B**, using *N,N*-dimethyl-2-(6-methyl-2-(*p*-tolyl)imidazo[1,2-*a*]pyridin-3-yl)acetamide (*Zolpidem*, 76.8 mg, 0.25 mmol) and benzyl 4-(5-oxo-1,4,2-dioxazol-3-yl)piperidine-1-carboxylate **4** (83.7 mg, 0.28 mmol) as substrates. The crude reaction mixture was analysed by LCMS using acidic mobile phase and the UV chromatogram is shown below:

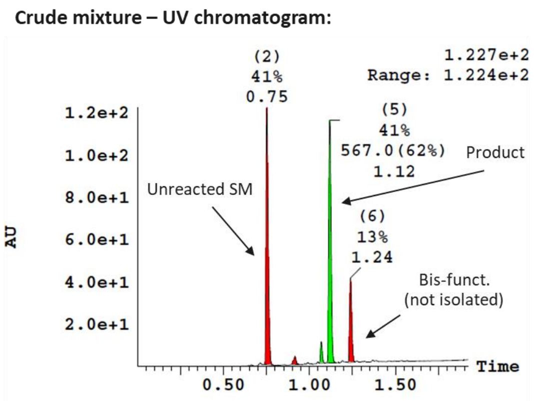

Supplementary Figure 24. LCMS UV chromatogram of the crude reaction mixture forming product **8d**.

Purification by 1st preparative reverse phase HPLC (15-55% MeCN in HCO_2H buffer, 240 nm), followed by a 2nd preparative reverse phase HPLC (35-75% MeCN in NH_4HCO_3 buffer, 240 nm) afforded amidated derivative **8d** as an off-white solid (46.4 mg, 33%). **^{1}H NMR** (500 MHz, DMSO-*d*$_6$) δ (ppm) 11.37 (s, 1 H), 8.21 (s, 1 H), 8.11 (s, 1 H), 7.53 (d, *J* = 9.1 Hz, 1 H), 7.40–7.28 (m, 5 H), 7.20 (dd, *J* = 9.2, 1.6 Hz, 1 H), 7.16 (d, *J* = 7.8 Hz, 1 H), 6.98 (dd, *J* = 7.9, 1.8 Hz, 1 H), 5.09 (s, 2 H), 4.08 (s, 2 H), 4.01 (br dt, *J* = 13.2, 3.6 Hz, 2 H), 3.11 (s, 3 H), 3.02–2.80 (m, 5 H), 2.51–2.44 (m, part. overlap with solvent signal, 1 H), 2.36–2.30 (m, 6 H), 1.86 (br dd, *J* = 13.3, 3.7 Hz, 2 H), 1.48 (qd, *J* = 12.2, 4.2 Hz, 2 H); **^{13}C NMR** (126 MHz, DMSO-*d*$_6$) δ (ppm) 172.1, 168.0, 154.4, 142.3, 140.5, 137.7, 137.0, 136.7, 128.7, 128.4 (2 C), 128.2, 127.8, 127.5 (2 C), 124.0, 122.7, 121.5, 121.3, 119.6, 116.9, 115.4, 66.2, 43.3, 43.1 (2 C), 37.0, 35.3, 29.1, 28.2 (br, 2 C), 21.2, 17.8; **HRMS** (m/z): $[M+H]^+$ calcd. for $C_{33}H_{37}N_5O_4$, 568.2924; found, 568.2954.

3-(4-(2-(1-((Benzyloxy)carbonyl)piperidine-4-carboxamido)phenyl)-5-phenyloxazol-2-yl)-propanoic acid (8e)

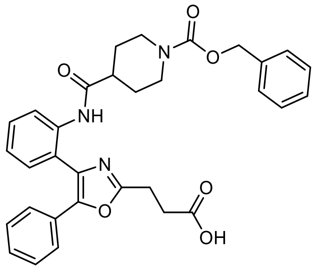

Prepared according to **General procedure B**, using 3-(4,5-diphenyloxazol-2-yl)propanoic acid (*Oxaprozin*, 73.3 mg, 0.25 mmol) and benzyl 4-(5-oxo-1,4,2-dioxazol-3-yl)piperidine-1-carboxylate **4** (83.7 mg, 0.28 mmol) as substrates. The crude reaction mixture was analysed by LCMS using acidic mobile phase and the UV chromatogram is shown below:

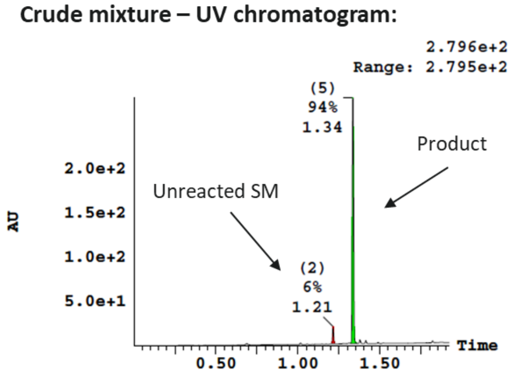

Supplementary Figure 25. LCMS UV chromatogram of the crude reaction mixture forming product **8e**.

Purification by preparative reverse phase HPLC (25-65% MeCN in HCO_2H buffer, 254 nm) afforded amidated derivative **8e** as a white solid (120 mg, 87%). **^{1}H NMR** (500 MHz, $CDCl_3$) δ (ppm) 9.18 (s, 1 H), 8.35 (d, *J* = 8.3 Hz, 1 H), 7.51 (dd, *J* = 7.6, 2.1 Hz, 2 H), 7.39– 7.27 (m, 10 H), 7.01 (t, *J* = 7.6 Hz, 1 H), 5.13 (s, 2 H), 4.24 (br s, 2 H), 3.18 (t, *J* = 6.4 Hz, 2 H), 2.91 (dd, *J* = 7.8, 5.3 Hz, 2 H), 2.84 (br s, 2 H), 2.42 (tt, *J* = 12.0, 3.7 Hz, 1 H), 1.89 (br d, *J* = 12.2 Hz, 2 H), 1.57 (qd, *J* = 12.3, 4.3 Hz, 2 H); **^{13}C NMR** (126 MHz, $CDCl_3$) δ (ppm) 174.1, 172.6, 162.4, 156.0, 147.0, 136.4, 136.1, 132.2, 129.9, 129.5, 128.9, 128.8 (2 C), 128.5 (2 C), 128.2, 128.0 (2 C), 127.9, 126.3 (2 C), 124.1, 122.4, 121.4, 67.6, 44.7, 43.8 (br, 2 C), 29.9, 28.1 (br, 2 C), 23.3; **HRMS** (m/z): $[M+H]^+$ calcd. for $C_{32}H_{31}N_3O_6$, 554.2291; found, 554.2314.

Benzyl 4-((5-((5*S*,8*S*,9*S*)-8-benzyl-5-(*tert*-butyl)-9-hydroxy-11-((*S*)-2-((methoxycarbonyl)amino)-3,3-dimethylbutanamido)-3,6-dioxo-2-oxa-4,7,11-triazadodecan-12-yl)-2-(pyridin-2-yl)phenyl)carbamoyl)piperidine-1-carboxylate (8f)

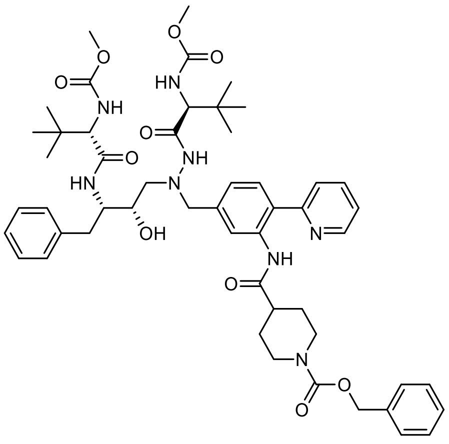

Prepared according to **General procedure B**, using methyl ((5*S*,10*S*,11*S*,14*S*)-11-benzyl-5-(*tert*-butyl)-10-hydroxy-15,15-dimethyl-3,6,13-trioxo-8-(4-(pyridin-2-yl)benzyl)-2-oxa-4,7,8,12-tetraazahexadecan-14-yl)carbamate (*Atazanavir*, 176 mg, 0.25 mmol) and benzyl 4-(5-oxo-1,4,2-dioxazol-3-yl)piperidine-1-carboxylate **4** (83.7 mg, 0.28 mmol) as substrates. The crude reaction mixture was analysed by LCMS using acidic mobile phase and the UV chromatogram is shown below:

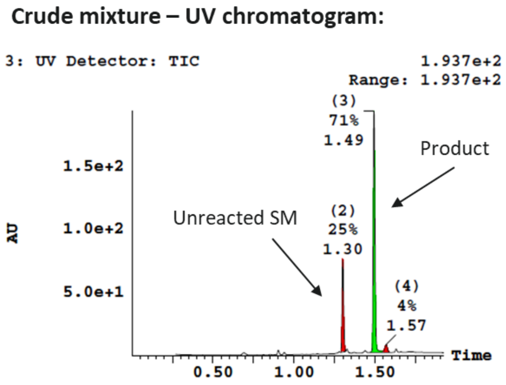

Supplementary Figure 26. LCMS UV chromatogram of the crude reaction mixture forming product **8f**.

Purification by preparative reverse phase HPLC (40-80% MeCN in HCO_2H buffer, 254 nm) afforded amidated derivative **8f** as an off-white solid (159 mg, 66%). **^{1}H NMR** (500 MHz, DMSO-d_6) δ (ppm) 12.07 (br s, 1 H), 9.14 (br s, 1 H), 8.69 (d, *J* = 4.3 Hz, 1 H), 8.21 (br s, 1 H), 8.00 (br t, *J* = 7.8 Hz, 1 H), 7.87 (d, *J* = 8.2 Hz, 1 H), 7.71 (d, *J* = 8.1 Hz, 1 H), 7.54 (d, *J* = 9.1 Hz, 1 H), 7.44 (br t, *J* = 6.3 Hz, 1 H), 7.40–7.34 (m, 4 H), 7.34–7.29 (m, 1 H), 7.26 (d, *J* = 8.1 Hz, 1 H), 7.24–7.16 (m, 4 H), 7.16–7.09 (m, 1 H), 7.00 (d, *J* = 9.4 Hz, 1 H), 6.86 (d, *J* = 9.4 Hz, 1 H), 5.09 (s, 2 H), 5.00 (br s, 1 H), 4.12–3.87 (m, 5 H), 3.83 (d, *J* = 9.4 Hz, 1 H), 3.64 (d, *J* = 9.4 Hz, 1 H), 3.59 (br d, *J* = 9.2 Hz, 1H), 3.56–3.47 (m, 6 H), 3.04–2.84 (br m, 2 H), 2.83–2.66 (m, 3 H), 2.66–2.58 (br m, 1 H), 2.58–2.50 (m, part. overlap with solvent signal, 1 H), 1.84 (br d, *J* = 13.1 Hz, 2 H), 1.54–1.40 (br m, 2 H), 0.74 (s, 9 H), 0.63 (s, 9 H); **^{13}C NMR** (126 MHz, DMSO-d_6) δ (ppm) 172.2, 170.2, 170.0, 156.5 (3 C), 154.4, 147.5 (br), 139.5 (br), 139.0 (2 C), 137.0, 136.9, 129.1 (2 C), 129.0, 128.5 (2 C), 128.0 (2 C), 127.9, 127.5 (2 C), 125.8, 125.0, 123.9 (br), 123.1 (br), 122.5 (br), 122.1 (br), 68.0, 66.2, 63.0, 61.3, 61.1, 60.8, 51.7, 51.4 (2 C), 43.1 (3 C), 37.7, 33.6, 33.4, 28.2 (br, 2 C), 26.7 (3 C), 26.3 (3 C); **HRMS** (m/z): $[M+H]^+$ calcd. for $C_{52}H_{68}N_8O_{10}$, 965.5137; found, 965.5114.

Benzyl 4-((5-(2-(methyl(4-methyl-5-sulfamoylthiazol-2-yl)amino)-2-oxoethyl)-2-(pyridin-2-yl)phenyl)carbamoyl)piperidine-1-carboxylate (8g)

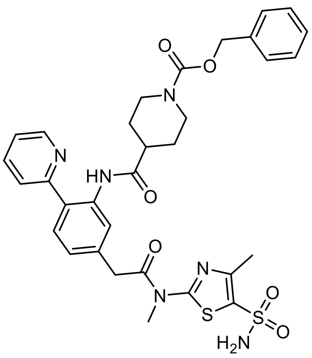

Prepared according to **General procedure B**, using *N*-methyl-*N*-(4-methyl-5-sulfamoylthiazol-2-yl)-2-(4-(pyridin-2-yl)phenyl)acetamide (*Pritelivir*, 60.4 mg, 0.15 mmol) and benzyl 4-(5-oxo-1,4,2-dioxazol-3-yl)piperidine-1-carboxylate **4** (50.2 mg, 0.17 mmol) as substrates. The crude reaction mixture was analysed by LCMS using acidic mobile phase and the UV chromatogram is shown below:

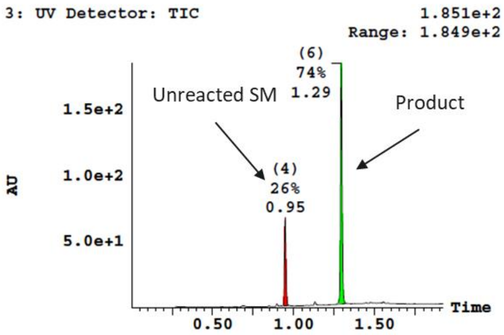

Supplementary Figure 27. LCMS UV chromatogram of the crude reaction mixture forming product **8g**.

Purification by preparative reverse phase HPLC (35-75% MeCN in NH_4HCO_3 buffer, 254 nm) afforded amidated derivative **8g** as an off-white solid (63.3 mg, 64%). **^{1}H NMR** (500 MHz, DMSO-*d*$_6$) δ (ppm) 12.13 (s, 1 H), 8.69 (dd, *J* = 4.9, 1.8 Hz, 1 H), 8.27 (s, 1 H), 7.98 (td, *J* = 7.7, 1.9 Hz, 1 H), 7.92 (d, *J* = 8.1 Hz, 1 H), 7.81 (d, *J* = 8.1 Hz, 1 H), 7.66 (br s, 2 H), 7.43 (dd, *J* = 7.4, 4.9 Hz, 1 H), 7.40–7.34 (m, 4 H), 7.35–7.28 (m, 1 H), 7.12 (dd, *J* = 8.0, 1.9 Hz, 1 H), 5.09 (s, 2 H), 4.21 (s, 2 H), 4.04 (dt, *J* = 13.5, 3.7 Hz, 2 H), 3.71 (s, 3 H), 2.93 (br s, 2 H), 2.55 (tt, *J* = 11.4, 3.8 Hz, 1 H), 2.48 (s, 3 H), 1.94–1.80 (br m, 2 H), 1.49 (qd, *J* = 12.3, 4.2 Hz, 2 H); **^{13}C NMR** (126 MHz, DMSO-*d*$_6$) δ (ppm) 172.4, 171.7, 158.5, 156.8, 154.4, 148.2, 147.9, 138.2, 137.1, 137.0, 135.8, 129.3, 128.5 (2 C), 128.3, 127.9, 127.6 (2 C), 125.3, 125.1, 123.1, 123.0, 122.5, 66.2, 43.15 (2 C), 43.07, 40.5, 34.3, 28.2 (br, 2C), 16.2; **HRMS** (m/z): $[M+H]^+$ calcd. for $C_{32}H_{34}N_6O_6S_2$, 663.2059; found, 663.2062.

Benzyl 4-((2-(9-(2-(benzyl(ethyl)amino)-2-oxoethyl)-7-methyl-8-oxo-8,9-dihydro-7*H*-purin-2-yl)phenyl)carbamoyl)piperidine-1-carboxylate (8h)

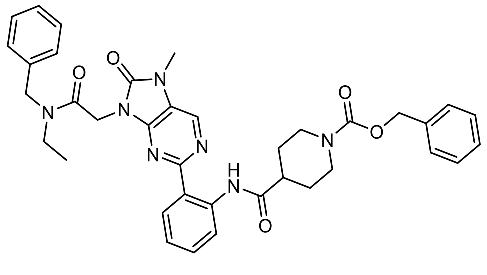

Prepared according to **General procedure B**, using *N*-benzyl-*N*-ethyl-2-(7-methyl-8-oxo-2-phenyl-7,8-dihydro-9*H*-purin-9-yl)acetamide (*Emapunil*, 72.3 mg, 0.18 mmol) and benzyl 4-(5-oxo-1,4,2-dioxazol-3-yl)piperidine-1-carboxylate **4** (60.3 mg, 0.20 mmol) as substrates. The crude reaction mixture was analysed by SFC-MS and the UV chromatogram is shown below:

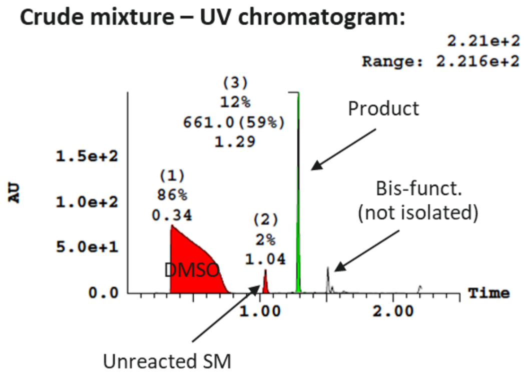

Supplementary Figure 28. LCMS UV chromatogram of the crude reaction mixture forming product **8h**.

Purification by supercritical fluid chromatography SFC (15-20% MeOH/H_2O/NH_3 97/3/0.2 in supercritical CO_2, 254 nm) afforded amidated derivative **8h** as a white solid (54.3 mg, 46%). **^{1}H NMR** (500 MHz, DMSO-d_6, observed as a 1:1 mixture of E:Z amide isomers) δ (ppm) 12.85 (s, 0.5 H), 12.83 (s, 0.5 H), 8.65–8.54 (m, 2 H), 8.43 (dd, *J* = 7.9, 1.5 Hz, 0.5 H), 8.35 (dd, *J* = 7.9, 1.5 Hz, 0.5 H), 7.49–7.33 (m, 7 H), 7.33–7.28 (m, 1 H), 7.27–7.12 (m, 4 H), 5.10 (s, 2 H), 4.94 (s, 1 H), 4.84 (s, 1 H), 4.76 (s, 1 H), 4.53 (s, 1 H), 4.09 (br d, *J* = 13.0 Hz, 2 H), 3.53–3.47 (m, 1 H), 3.45 (s, 1.5 H), 3.43 (s, 1.5 H), 3.29 (q, *J* = 7.0 Hz, 1 H), 2.95 (br s, 2 H), 2.68–2.56 (m, 1 H), 1.96 (br d, *J* = 12.6 Hz, 2 H), 1.65–1.48 (m, 2 H), 1.22 (t, *J* = 7.0 Hz, 1.5 H), 1.00 (t, *J* = 7.0 Hz, 1.5 H); **^{13}C NMR** (126 MHz, DMSO-d_6, observed as a 1:1 mixture of E:Z amide isomers) δ (ppm) 172.61 and 172.58 (1 C), 165.4 and 165.2 (1 C), 156.4 and 156.3 (1 C), 154.5 (1 C), 152.99 and 152.96 (1 C), 149.65 and 149.59 (1 C), 138.71 and 138.68 (1 C), 137.7 and 137.2 (1 C), 137.0 (1 C), 130.7–130.5 (m, 2 C), 129.6 and 129.5 (1 C), 128.8 and 128.37 (2 C), 128.44 (2 C), 127.8 (1 C), 127.53 (2 C), 127.48 and 127.1 (1 C), 127.4 and 126.5 (2 C), 122.9 and 122.8 (1 C), 122.6 and 122.7 (1 C), 121.7 (1 C), 120.3 (1 C), 66.2 (1 C), 49.1 and 47.9 (1 C), 43.4 (1 C), 43.2 (2 C), 41.4 and 40.9 (1 C), 41.21 and 41.16 (1 C), 28.3 (br, 2 C), 27.52 and 27.48 (1 C), 13.8 and 12.6 (1 C); **HRMS** (m/z): $[M+H]^+$ calcd. for $C_{37}H_{39}N_7O_5$, 662.3091; found, 662.3099.

Benzyl 4-((2-(5-(*iso*-propyl(4-(2-(methylsulfonamido)-2-oxoethoxy)butyl)amino)-3-phenylpyrazin-2-yl)phenyl)carbamoyl)piperidine-1-carboxylate (8i)

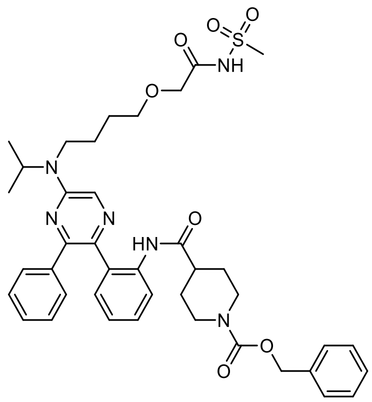

Prepared according to **General procedure B**, using 2-(4-((5,6-diphenylpyrazin-2-yl)(*iso*-propyl)amino)butoxy)-*N*-(methylsulfonyl)acetamide (*Selexipag*, 74.5 mg, 0.15 mmol) and benzyl 4-(5-oxo-1,4,2-dioxazol-3-yl)piperidine-1-carboxylate **4** (50.2 mg, 0.17 mmol) as substrates. The crude reaction mixture was analysed by LCMS using basic mobile phase and the UV chromatogram is shown below:

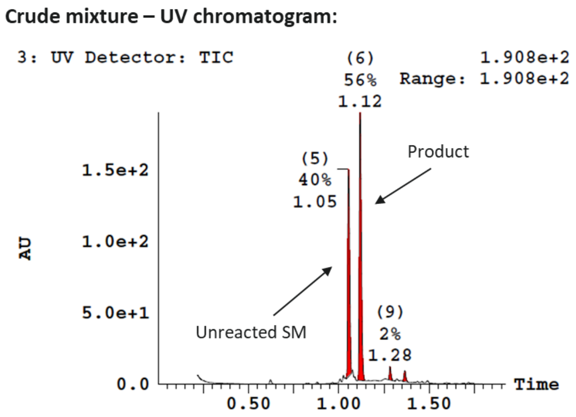

Supplementary Figure 29. LCMS UV chromatogram of the crude reaction mixture forming product **8i**.

Purification by preparative reverse phase HPLC (20-65% MeCN in NH_4HCO_3 buffer, 240 nm) afforded amidated derivative **8i** as a pale yellow solid (50.8 mg, 45%). **^{1}H NMR** (500 MHz, DMSO-*d*$_6$) δ (ppm) 11.72 (br s, 1 H), 9.27 (s, 1 H), 8.12 (s, 1 H), 7.72 (d, *J* = 8.1 Hz, 1 H), 7.45–7.39 (br m, 2 H), 7.39–7.28 (br m, 5 H), 7.28–7.16 (br m, 4 H), 6.98–6.89 (br m, 2 H), 5.06 (s, 2 H), 4.87–4.71 (br m, 1 H), 4.01 (s, 2 H), 3.95 (br d, *J* = 12.9 Hz, 2 H), 3.52 (br t, *J* = 6.3 Hz, 2 H), 3.44 (br t, J = 7.5 Hz, 2 H), 3.20 (s, 3 H), 2.94–2.69 (m, 2 H), 2.42 (br t, *J* = 11.3 Hz, 1 H), 1.74–1.55 (br m, 6 H), 1.34 (br q, *J* = 12.2 Hz, 2 H), 1.22 (d, *J* = 6.4 Hz, 6 H); **^{13}C NMR** (126 MHz, DMSO-*d*$_6$) δ (ppm) 172.3, 170.3, 154.4, 151.4, 148.7, 139.1, 137.0, 136.4, 136.2, 131.9, 131.3, 129.1 (2 C), 128.4 (2 C), 128.0, 127.8 (2 C), 127.7, 127.53 (2 C), 127.49, 127.2, 123.9, 123.8, 70.6, 69.1, 66.1, 45.6, 43.0 (2 C), 41.7 (2 C), 41.1, 28.0 (2 C), 26.7, 25.6, 20.0 (2 C); **HRMS** (m/z): $[M+H]^+$ calcd. for $C_{40}H_{48}N_6O_7S$, 757.3383; found, 757.3384.

Benzyl 4-((2-(7-chloro-1-methyl-2-oxo-2,3-dihydro-1*H*-benzo[*e*][1,4]diazepin-5-yl)phenyl)carbamoyl)piperidine-1-carboxylate (8j)

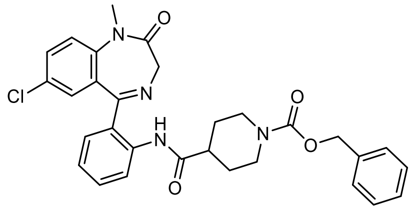

Prepared according to **General procedure B**, using 7-chloro-1-methyl-5-phenyl-1,3-dihydro-2*H*-benzo[e][1,4]diazepin-2-one (*Diazepam,* 71.2 mg, 0.25 mmol) and benzyl 4-(5-oxo-1,4,2-dioxazol-3-yl)piperidine-1-carboxylate **4** (83.7 mg, 0.28 mmol) as substrates. The crude reaction mixture was analysed by LCMS using acidic mobile phase and the UV chromatogram is shown below:

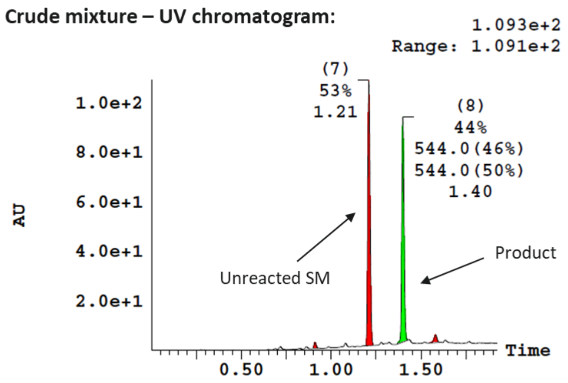

Supplementary Figure 30. LCMS UV chromatogram of the crude reaction mixture forming product **8j**.

Purification by automated flash column chromatography (15-100% EtOAc/heptane) afforded amidated derivative **8j** as a pale yellow oil (65.3 mg, 48%). R_f 0.35 (60% EtOAc/heptane); ^{1}**H NMR** (500 MHz, $CDCl_3$) δ (ppm) 12.07 (s, 1 H), 8.62 (dd, *J* = 8.4, 1.2 Hz, 1 H), 7.54 (dd, *J* = 8.8, 2.5 Hz, 1 H), 7.44 (ddd, *J* = 8.6, 7.1, 1.8 Hz, 1 H), 7.40–7.28 (m, 6 H), 7.27–7.24 (m, part. overlap with solvent signal, 1 H), 7.07 (dd, *J* = 8.0, 1.8 Hz, 1 H), 7.03 (td, *J* = 7.8, 1.3 Hz, 1 H), 5.14 (s, 2 H), 4.78 (d, *J* = 11.0 Hz, 1 H), 4.25 (br s, 2 H), 3.86 (d, *J* = 11.1 Hz, 1 H), 3.39 (s, 3 H), 2.93 (br s, 2 H), 2.45 (tt, *J* = 11.4, 3.7 Hz, 1 H), 1.99 (br s, 2 H), 1.85–1.67 (m, 2 H); ^{13}**C NMR** (126 MHz, $CDCl_3$) δ (ppm) 173.3, 170.9, 169.7, 155.3, 142.6, 139.6, 136.8, 133.2, 132.0 (2 C), 130.4, 130.3, 129.9, 128.6 (2 C), 128.1, 128.0 (2 C), 123.3, 122.8, 122.6, 121.7, 67.3, 56.3, 44.7 (br), 43.7 and 43.6 (2 C), 34.9, 28.6 (2 C); **HRMS** (m/z): $[M+H]^+$ calcd. for $C_{30}H_{29}{}^{35}ClN_4O_4$, 545.1956; found, 545.196.

Benzyl 4-((2-((3-((2,4-difluorophenyl)carbamoyl)pyridin-2-yl)oxy)-4-(trifluoromethyl)phenyl)carbamoyl)piperidine-1-carboxylate (8k)

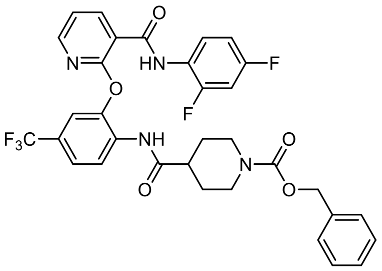

Prepared according to **General procedure A**, using *N*-(2,4-difluorophenyl)-2-(3-(trifluoromethyl)phenoxy)nicotinamide (*Diflufenican,* 98.6 mg, 0.25 mmol) and benzyl 4-(5-oxo-1,4,2-dioxazol-3-yl)piperidine-1-carboxylate **4** (83.7 mg, 0.28 mmol) as substrates. The crude reaction mixture was analysed by LCMS using acidic mobile phase and the UV chromatogram is shown below:

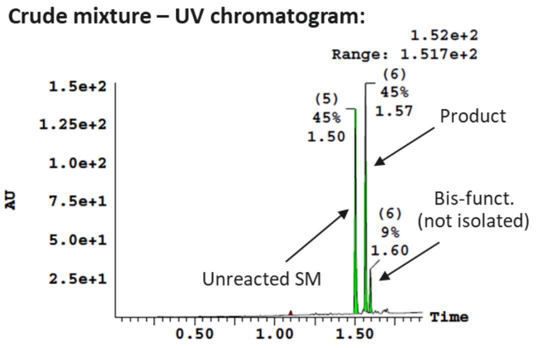

Supplementary Figure 31. LCMS UV chromatogram of the crude reaction mixture forming product **8k**.

Purification by preparative reverse phase HPLC (40-85% MeCN in HCO_2H buffer, 240 nm) afforded amidated derivative **8k** as a white solid (63.7 mg, 39%). ^{1}**H NMR** (500 MHz, DMSO-d_6) δ (ppm) 10.33 (s, 1 H), 9.92 (s, 1 H), 8.36–8.25 (m, 3 H), 8.04 (td, *J* = 8.9, 6.1 Hz, 1 H), 7.79 (d, *J* = 2.1 Hz, 1 H), 7.60 (dd, *J* = 8.8, 2.2 Hz, 1 H), 7.44–7.27 (m, 7 H), 7.19–7.11 (m, 1 H), 5.07 (s, 2 H), 3.95 (br dt, *J* = 13.6, 3.6 Hz, 2 H), 2.84–2.67 (br m, 2 H), 2.63 (tt, *J* = 11.4, 3.7 Hz, 1 H), 1.60 (br dd, *J* = 13.5, 3.7 Hz, 2 H), 1.38 (qd, *J* = 12.3, 4.3 Hz, 2 H); ^{13}**C NMR** (126 MHz, DMSO-d_6) δ (ppm) 173.7, 163.2, 158.9 (dd, J_{CF} = 244.5, 11.6 Hz), 158.7, 154.3, 154.2 (dd, J_{CF} = 248.8, 12.6 Hz), 149.6, 142.9, 140.9, 137.0, 135.1, 128.4 (2 C), 127.8, 127.5 (2 C), 125.5 (dd, J_{CF} = 9.5, 2.7 Hz), 124.1 (q, J_{CF} = 32.6 Hz), 123.9 (q, J_{CF} = 271.7 Hz), 122.9, 122.4 (q, J_{CF} = 4.1 Hz), 122.3 (dd, J_{CF} = 11.7, 3.7 Hz), 120.5 (q, J_{CF} = 3.8 Hz), 119.9, 119.1, 111.4 (dd, J_{CF} = 22.2, 3.6 Hz), 104.4 (dd, J_{CF} = 27.0, 23.8 Hz), 66.2, 42.9 (2 C), 41.8, 27.9 (br, 2 C); ^{19}**F NMR** (471 MHz, DMSO-d_6) δ (ppm) -60.3 (s, 3 F), -113.8 (d, *J* = 5.4 Hz, 1 F), -119.6 (d, *J* = 5.4 Hz, 1 F); **HRMS** (m/z): $[M+H]^+$ calcd. for $C_{33}H_{27}F_5N_4O_5$, 655.1980; found, 655.1990.

3-Oxo-1,3-dihydroisobenzofuran-1-yl 2-((2-(1-((benzyloxy)carbonyl)piperidine-4-carboxamido)-5-(trifluoromethyl)phenyl)amino)nicotinate (8l)

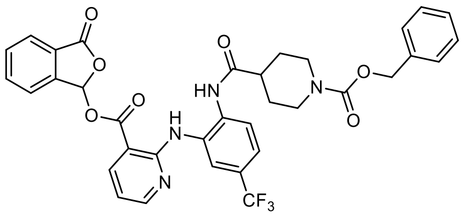

Prepared according to **General procedure B**, using 3-oxo-1,3-dihydroisobenzofuran-1-yl 2-((3-(trifluoromethyl)phenyl)amino)nicotinate (*Talniflumate,* 104 mg, 0.25 mmol) and benzyl 4-(5-oxo-1,4,2-dioxazol-3-yl)piperidine-1-carboxylate **4** (83.7 mg, 0.28 mmol) as substrates. The crude reaction mixture was analysed by LCMS using acidic mobile phase and the UV chromatogram is shown below:

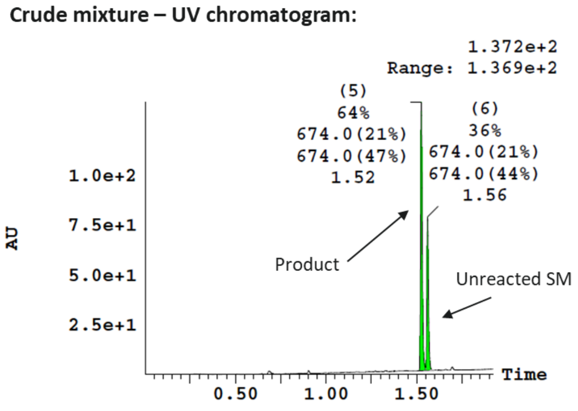

Supplementary Figure 32. LCMS UV chromatogram of the crude reaction mixture forming product **8l**.

Purification by preparative reverse phase HPLC (45-85% MeCN in HCO_2H buffer, 240 nm) afforded amidated derivative **8l** as a pale yellow solid (100 mg, 59%). **^{1}H NMR** (500 MHz, DMSO-d_6) δ (ppm) 10.01 (s, 1 H), 9.94 (s, 1 H), 8.63 (s, 1 H), 8.47 (d, *J* = 5.7 Hz, 1 H), 8.21 (d, *J* = 7.8 Hz, 1 H), 7.99 (d, *J* = 7.6 Hz, 1 H), 7.96–7.97 (m, 2 H), 7.79 (dt, *J* = 8.0, 4.2 Hz, 1 H), 7.71 (s, 1 H), 7.57 (d, *J* = 8.3 Hz, 1 H), 7.44 (d, *J* = 7.4 Hz, 1 H), 7.39–7.24 (m, 5 H), 6.91 (dd, *J* = 7.9, 4.7 Hz, 1 H), 5.06 (s, 2 H), 4.18–4.00 (m, 2 H), 3.08–2.79 (m, 2 H), 2.75–2.64 (m, 1 H), 2.05–1.88 (m, 2 H), 1.69–1.49 (m, 2 H); **^{13}C NMR** (126 MHz, DMSO-d_6) δ (ppm) 174.0, 167.7, 165.3, 155.4, 154.4, 154.2, 144.2, 140.9, 137.0, 135.4, 134.9, 132.6, 131.7, 128.4 (2 C), 127.8, 127.5 (2 C), 127.1, 126.2 (q, J_{CF} = 31.6 Hz), 125.8, 125.3, 124.5, 122.1 (q, J_{CF} = 272.0 Hz), 119.7 (br), 119.5 (br), 114.9, 106.2, 93.5, 66.1, 43.1 (2 C), 41.9, 28.0 (br, 2 C); **^{19}F NMR** (471 MHz, DMSO-d_6) δ (ppm) -60.7 (s, 3 F); **HRMS** (m/z): $[M+H]^+$ calcd. for $C_{35}H_{29}F_3N_4O_7$, 675.2067; found, 675.2087.

[*Note: Decomposition during preparative reverse phase HPLC was observed in basic buffer*.]

(*E*)-3-(1-((Benzyloxy)carbonyl)piperidine-4-carboxamido)-2-(3-(3,4-dimethoxyphenyl)acrylamido)benzoic acid (8m)

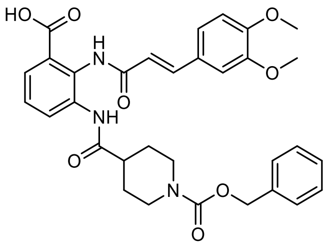

Prepared according to **General procedure A**, using (*E*)-2-(3-(3,4-dimethoxyphenyl)acrylamido)benzoic acid (*Tranilast,* 81.8 mg, 0.25 mmol) and benzyl 4-(5-oxo-1,4,2-dioxazol-3-yl)piperidine-1-carboxylate **4** (83.7 mg, 0.28 mmol) as substrates. The crude reaction mixture was analysed by LCMS using acidic mobile phase and the UV chromatogram is shown below:

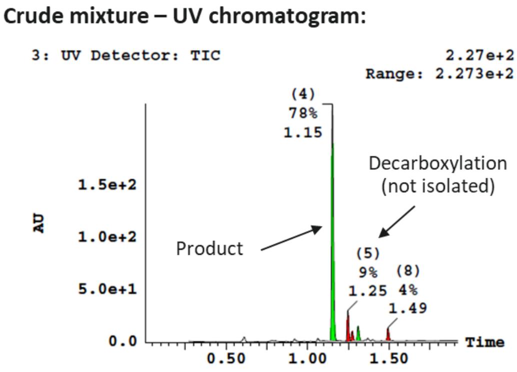

Supplementary Figure 33. LCMS UV chromatogram of the crude reaction mixture forming product **8m**.

Purification by preparative reverse phase HPLC (30-80% MeCN in HCO_2H buffer, 240 nm) afforded amidated derivative **8m** as a pale yellow solid (93.7 mg, 64%). **^{1}H NMR** (500 MHz, DMSO-d_6) δ (ppm) 12.91 (br s, 1 H), 9.69 (s, 1 H), 9.31 (s, 1 H), 7.82 (dd, *J* = 8.1, 1.5 Hz, 1 H), 7.60 (dd, *J* = 7.7, 1.6 Hz, 1 H), 7.51 (d, *J* = 15.7 Hz, 1 H), 7.40–7.28 (m, 6 H), 7.26 (d, *J* = 2.0 Hz, 1 H), 7.20 (dd, *J* = 8.3, 2.0 Hz, 1 H), 7.01 (d, *J* = 8.3 Hz, 1 H), 6.80 (d, *J* = 15.7 Hz, 1 H), 5.06 (s, 2 H), 4.07–3.97 (m, 2 H), 3.82 (s, 3 H), 3.80 (s, 3 H), 3.03–2.73 (br m, 2 H), 2.62 (tt, *J* = 11.3, 3.8 Hz, 1 H), 1.89–1.74 (br m, 2 H), 1.52 (qd, *J* = 12.8, 4.2 Hz, 2 H); **^{13}C NMR** (126 MHz, DMSO-d_6) δ (ppm) 173.1, 167.7, 164.8, 154.4, 150.5, 149.0, 140.7, 137.0, 133.6, 129.5, 128.7, 128.4 (2 C), 128.2, 127.8, 127.5 (2 C), 127.4, 126.3, 125.5, 122.0, 119.5, 111.7, 110.2, 66.2, 55.6, 55.5, 43.0 (2 C), 41.9, 28.2 (br, 2 C); **HRMS** (m/z): $[M+H]^+$ calcd. for $C_{32}H_{33}N_3O_8$, 588.2346; found, 588.2366.

[*Note: small amount of decarboxylated product was observed by crude LCMS (< 10% by UV, not isolated)*.]

4-((3-(1-((Benzyloxy)carbonyl)piperidine-4-carboxamido)-5,5,8,8-tetramethyl-5,6,7,8-tetrahydronaphthalen-2-yl)carbamoyl)benzoic acid (8n)

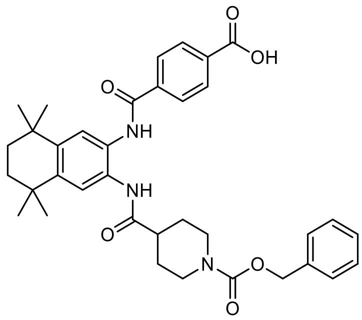

Prepared according to **General procedure B**, using 4-((5,5,8,8-tetramethyl-5,6,7,8-tetrahydronaphthalen-2-yl)carbamoyl)benzoic acid (*Tamibarotene,* 87.9 mg, 0.25 mmol) and benzyl 4-(5-oxo-1,4,2-dioxazol-3-yl)piperidine-1-carboxylate **4** (83.7 mg, 0.28 mmol) as substrates, and PivOH (7.66 mg, 0.08 mmol) in place of $(PhO)_2PO_2H$. The crude reaction mixture was analysed by LCMS using acidic mobile phase and the UV chromatogram is shown below:

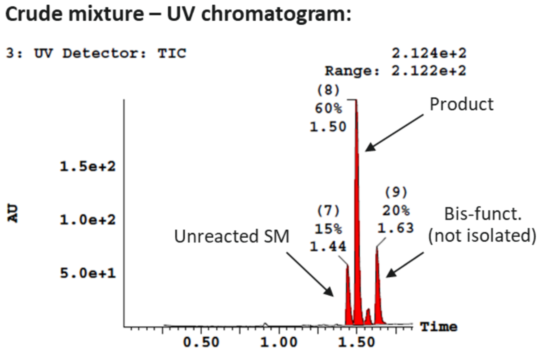

Supplementary Figure 34. LCMS UV chromatogram of the crude reaction mixture forming product **8n**.

Purification by preparative reverse phase HPLC (40-80% MeCN in HCO_2H buffer, 254 nm) afforded amidated derivative **8n** as a white solid (85.0 mg, 56%). **^{1}H NMR** (500 MHz, DMSO-*d*$_6$) δ (ppm) 9.91 (s, 1 H), 9.59 (s, 1 H), 8.07 (d, *J* = 8.2 Hz, 2 H), 8.01 (d, *J* = 8.2 Hz, 2 H), 7.54 (s, 1 H), 7.41 (s, 1 H), 7.39–7.28 (m, 5 H), 5.07 (s, 2 H), 3.99 (br dt, *J* = 13.2, 3.7 Hz, 2 H), 3.01–2.72 (br m, 2 H), 2.60 (tt, *J* = 11.3, 3.5 Hz, 1 H), 1.83–1.72 (br m, 2 H), 1.65 (s, 4 H), 1.49 (qd, *J* = 12.2, 4.3 Hz, 2 H), 1.24 (s, 12 H); **^{13}C NMR** (126 MHz, DMSO-*d*$_6$) δ (ppm) 173.6, 166.8, 164.1, 154.4, 142.1, 141.6, 138.0, 137.0, 133.6, 129.4 (2 C), 129.1, 128.4 (2 C), 128.2, 127.8 (2 C), 127.6, 127.5 (2 C), 123.6, 122.3, 66.2, 43.0 (2 C), 41.8, 34.5 (2 C), 33.8, 38.7, 31.6 (4 C), 28.1 (br, 2 C); **HRMS** (m/z): $[M+H]^+$ calcd. for $C_{36}H_{41}N_3O_6$, 612.3074; found, 612.3060.

[*Note: the signal from acidic -CO_2H proton was not observed in the ^{1}H NMR*.]

(*S*)-4-(2-Benzamido-3-(4-hydroxyphenyl)propanamido)-3-(1-((benzyloxy)carbonyl)piperidine-4-carboxamido)benzoic acid (8o)

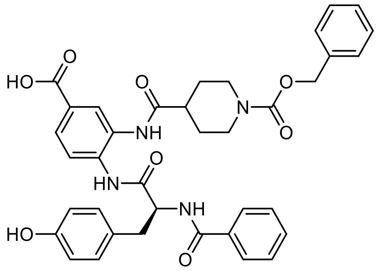

Prepared according to **General procedure A**, using (*S*)-4-(2-benzamido-3-(4-hydroxyphenyl)propanamido)benzoic acid (*Bentiromide*, 101 mg, 0.25 mmol) and benzyl 4-(5-oxo-1,4,2-dioxazol-3-yl)piperidine-1-carboxylate **4** (83.7 mg, 0.28 mmol) as substrates. The crude reaction mixture was analysed by LCMS using acidic mobile phase and the UV chromatogram is shown below:

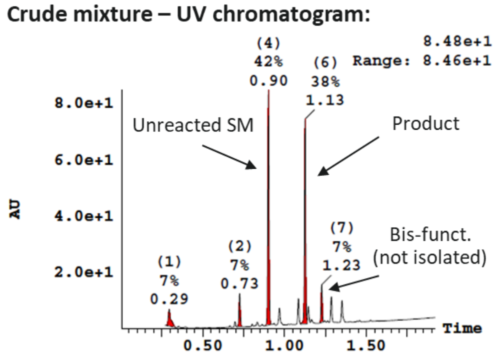

Supplementary Figure 35. LCMS UV chromatogram of the crude reaction mixture forming product **8o**.

Purification by preparative reverse phase HPLC (15-65% MeCN in HCO_2H buffer, 240 nm) afforded amidated derivative **8o** as a white solid (47.1 mg, 28%). **^{1}H NMR** (500 MHz, DMSO-d_6) δ (ppm) 12.97 (br s, 1 H), 9.72 (s, 1 H), 9.46 (s, 1 H), 9.21 (br s, 1 H), 8.86 (d, J = 7.3 Hz, 1 H), 8.07 (s, 1 H), 7.86 (d, J = 7.5 Hz, 2 H), 7.79–7.66 (m, 2 H), 7.49 (t, J = 7.3 Hz, 1 H), 7.46–7.41 (m, 2 H), 7.41–7.28 (m, 5 H), 7.15 (d, J = 8.0 Hz, 2 H), 6.66 (d, J = 8.0 Hz, 2 H), 5.08 (s, 2 H), 4.72 (q, J = 7.0 Hz, 1 H), 4.07–3.88 (br m, 2 H), 3.17 (dd, J = 14.0, 5.2 Hz, 1 H), 3.02 (dd, J = 13.8, 9.7 Hz, 1 H), 2.88–2.61 (m, 2 H), 2.56–2.50 (m, part. overlap with solvent signal, 1 H), 1.91–1.63 (br m, 2 H), 1.53–1.27 (br m, 2 H); **^{13}C NMR** (126 MHz, DMSO-d_6) δ (ppm) 173.6, 170.8, 166.9, 166.7, 155.9, 154.3, 137.0, 134.6, 133.6, 131.6, 130.2 (2 C), 130.1, 128.5 (2 C), 128.2 (2 C), 128.0, 127.9, 127.6 (4 C), 127.2, 126.3, 126.1, 124.1, 115.0 (2 C), 66.2, 56.5, 42.9 (2 C), 42.1, 35.7, 27.9 (br, 2 C); **HRMS** (m/z): $[M+H]^+$ calcd. for $C_{37}H_{36}N_4O_8$, 665.2611; found, 665.2589.

(3*R*,5*R*)-7-(3-((2-(1-((Benzyloxy)carbonyl)piperidine-4-carboxamido)phenyl)carbamoyl)-5-(4-fluorophenyl)-2-isopropyl-4-phenyl-1*H*-pyrrol-1-yl)-3,5-dihydroxyheptanoic acid (8p)

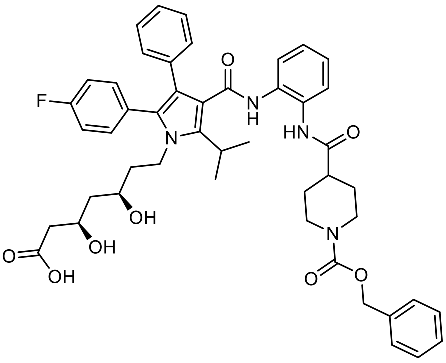

Prepared according to **General procedure A**, using (3*R*,5*R*)-7-[2-(4-fluorophenyl)-3-phenyl-4-(phenylcarbamoyl)-5-propan-2-ylpyrrol-1-yl]-3,5-dihydroxyheptanoic acid (*Atorvastatin*, 140 mg, 0.25 mmol) and benzyl 4-(5-oxo-1,4,2-dioxazol-3-yl)piperidine-1-carboxylate **4** (83.7 mg, 0.28 mmol) as substrates. The crude reaction mixture was analysed by LCMS using acidic mobile phase and the UV chromatogram is shown below:

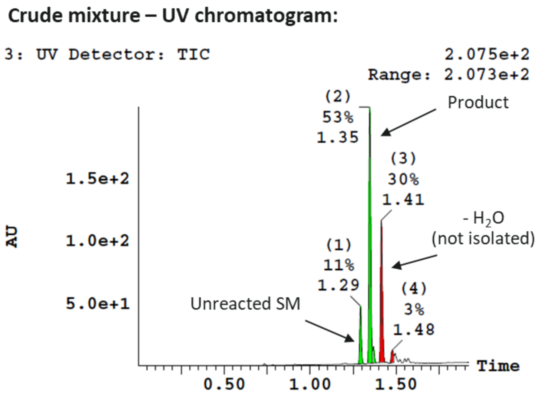

Supplementary Figure 36. LCMS UV chromatogram of the crude reaction mixture forming product **8p**.

Purification by preparative reverse phase HPLC (25-65% MeCN in HCO_2H buffer, 254 nm) afforded amidated derivative **8p** as a white solid (98.3 mg, 48%). **^{1}H NMR** (500 MHz, DMSO-d_6) δ (ppm) 12.04 (br s, 1 H), 9.39 (s, 1 H), 9.06 (s, 1 H), 7.58 (dd, J = 7.7, 2.0 Hz, 1 H), 7.41–7.34 (m, 4 H), 7.34–7.29 (m, 2 H), 7.29–7.23 (m, 2 H), 7.19 (t, J = 8.8 Hz, 2 H), 7.15–7.01 (m, 7 H), 5.08 (s, 2 H), 4.71 (br s, 1 H), 4.64 (d, J = 4.9 Hz, 1 H), 4.04–3.90 (m, 3 H), 3.89–3.82 (m, 1 H), 3.82–3.72 (m, 1 H), 3.60–3.49 (m, 1 H), 3.27 (h, J = 7.2 Hz, 1 H), 3.01–2.69 (br m, 2 H), 2.29 (dd, J = 15.0, 5.0 Hz, 1 H), 2.25–2.14 (m, 2 H), 1.74–1.60 (m, 3 H), 1.60–1.50 (m, 1 H), 1.50–1.29 (m, 10 H); **^{13}C NMR** (126 MHz, DMSO-d_6) δ (ppm) 172.9, 172.4, 167.0, 161.7 (d, J_{CF} = 245.4 Hz), 154.4, 137.0, 136.5, 134.7, 133.5 (d, J_{CF} = 8.2 Hz, 2 C), 130.4, 129.9, 129.2 (2 C), 128.54 (d, J_{CF} = 3.3 Hz), 128.46 (2 C), 127.9, 127.8

(2 C), 127.6 (2 C), 127.5, 125.6, 124.9, 124.6, 124.5, 124.2, 120.6, 116.5, 115.4 (d, J_{CF} = 21.3 Hz, 2 C), 66.2, 65.9, 65.4, 43.7, 42.9 (2 C), 42.5, 42.2, 40.9, 38.8, 28.0 (br, 2 C), 25.7, 22.5, 22.4; ^{19}**F NMR** (471 MHz, DMSO-d_6) δ (ppm) -113.8 (s, 1 F); **HRMS** (m/z): $[M+H]^+$ calcd. for $C_{47}H_{51}FN_4O_8$, 819.3769; found, 819.3788.

[*Note: dehydration of the desired product, likely to form the α,β-unsaturated derivative, was observed by crude LCMS (approx. 30% by UV, not isolated)*.]

Benzyl (*R*)-4-((5-(1-(2,2-difluorobenzo[*d*][1,3]dioxol-5-yl)cyclopropane-1-carboxamido)-1-(2,3-dihydroxypropyl)-6-fluoro-2-(1-hydroxy-2-methylpropan-2-yl)-1*H*-indol-4-yl)carbamoyl)piperidine-1-carboxylate (8q)

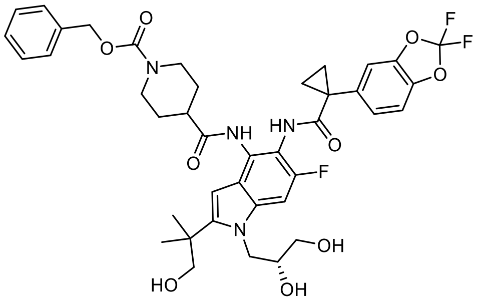

Prepared according to **General procedure A**, using (*R*)-1-(2,2-difluorobenzo[*d*][1,3]dioxol-5-yl)-*N*-(1-(2,3-dihydroxypropyl)-6-fluoro-2-(1-hydroxy-2-methylpropan-2-yl)-1*H*-indol-5-yl)cyclopropane-1-carboxamide (*Tezacaftor,* 130 mg, 0.25 mmol) and benzyl 4-(5-oxo-1,4,2-dioxazol-3-yl)piperidine-1-carboxylate **4** (83.7 mg, 0.28 mmol) as substrates. The crude reaction mixture was analysed by LCMS using acidic mobile phase and the UV chromatogram is shown below:

Crude mixture – UV chromatogram:

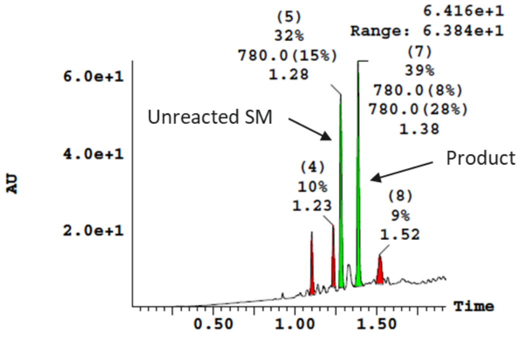

Supplementary Figure 37. LCMS UV chromatogram of the crude reaction mixture forming product **8q**.

Purification by preparative reverse phase HPLC (30-75% MeCN in HCO_2H buffer, 240 nm) afforded amidated derivative **8q** as an off-white solid (52.4 mg, 27%). ^{1}**H NMR** (500 MHz, DMSO-d_6) δ (ppm) 10.02 (s, 1 H), 7.62 (s, 1 H), 7.50 (d, *J* = 1.7 Hz, 1 H), 7.43–7.35 (m, 5 H), 7.35–7.25 (m, 3 H), 6.31 (s, 1 H), 5.17–5.08 (m, 2 H), 5.08–5.02 (m, 1 H), 4.93 (t, *J* = 5.5 Hz, 1 H), 4.74 (t, *J* = 5.8 Hz, 1 H), 4.41 (dd, *J* = 15.3, 3.0 Hz, 1 H), 4.18–4.07 (m, 3 H), 3.96–3.87 (m, 1 H), 3.64 (dd, *J* = 10.9, 5.0 Hz, 1 H), 3.57 (dd, *J* = 10.9, 5.5 Hz, 1 H), 3.47 (dt, *J* = 9.9, 4.7 Hz, 1 H), 3.43–3.37 (m, part. overlap with H_2O signal, 1 H), 3.02–2.76 (br m, 2 H), 2.67 (tt, *J* = 11.8, 3.8 Hz, 1 H), 1.87–1.69 (br m, 2 H), 1.45–1.29 (m, 10 H), 1.16–1.06 (m, 2 H); ^{13}**C NMR** (126 MHz, DMSO-d_6) δ (ppm) 173.6, 170.4, 154.4, 153.4 (d, J_{CF} = 238.8 Hz), 146.6 (d, J_{CF} = 3.3 Hz), 142.9, 142.3, 137.0, 136.1, 135.8 (d, J_{CF} = 13.6 Hz), 131.3 (t, J_{CF} = 252.6 Hz), 128.4 (2 C), 127.8, 127.5 (2 C), 127.4, 123.5 (d, J_{CF} = 5.3 Hz), 118.1, 112.9 (d, J_{CF} = 17.3 Hz), 112.8, 110.2, 98.5, 96.2 (d, J_{CF} = 25.9 Hz), 71.0, 69.2, 66.2, 64.0, 48.6, 43.0 (2 C), 41.5, 38.3, 30.2, 28.3 (br, 2 C), 26.2, 25.7, 15.9, 15.8; ^{19}**F NMR** (471 MHz, DMSO-d_6) δ (ppm) -48.9 (s, 2 F), -125.5 (s, 1 F); **HRMS** (m/z): $[M+H]^+$ calcd. for $C_{40}H_{43}F_3N_4O_9$, 781.3055; found, 781.3074.

[*Note: other small side products observed by crude LCMS were not identified*.]

Benzyl (±)-4-((5-(7-chloro-5-hydroxy-2,3,4,5-tetrahydro-1*H*-benzo[*b*]azepine-1-carbonyl)-4-methyl-2-(2-methylbenzamido)phenyl)carbamoyl)piperidine-1-carboxylate (8r)

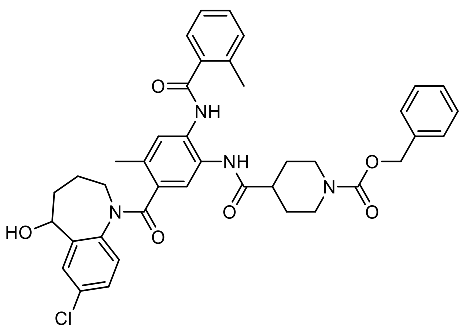

Prepared according to **General procedure B**, using (±)-*N*-(4-(7-chloro-5-hydroxy-2,3,4,5-tetrahydro-1*H*-benzo[*b*]azepine-1-carbonyl)-3-methylphenyl)-2-methylbenzamide (*Tolvaptan,* 112 mg, 0.25 mmol) and benzyl 4-(5-oxo-1,4,2-dioxazol-3-yl)piperidine-1-carboxylate **4** (83.7 mg, 0.28 mmol) as substrates, and PivOH (7.66 mg, 0.08 mmol) in place of $(PhO)_2PO_2H$. The crude reaction mixture was analysed by LCMS using acidic mobile phase and the UV chromatogram is shown below:

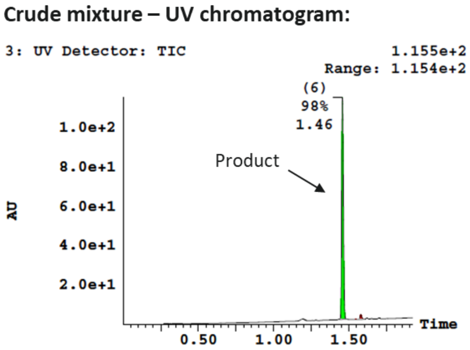

Supplementary Figure 38. LCMS UV chromatogram of the crude reaction mixture forming product **8r**.

Purification by preparative reverse phase HPLC (35-75% MeCN in HCO_2H buffer, 240 nm) afforded amidated derivative **8r** as a white solid (171 mg, 97%). **^{1}H NMR** (500 MHz, DMSO-d_6) δ (ppm) 9.85–9.15 (m, 2 H), 7.79–6.67 (m, 14 H), 5.64 (br s, 1 H), 5.08 (s, 2 H), 4.92–4.53 (m, 2 H), 4.11–3.90 (m, 2 H), 3.14–2.55 (m, 4 H), 2.47–2.20 (m, 6 H), 2.19–1.36 (m, 8 H); **^{13}C NMR** (126 MHz, DMSO-d_6, only major peaks are reported) δ (ppm) 173.3, 173.1, 169.3, 167.9, 167.8, 154.41, 154.38, 144.9, 138.4, 137.0, 136.4, 136.3, 135.7, 133.2, 131.7, 131.5, 130.8, 130.7, 130.6, 130.04, 129.95, 129.6, 128.5, 127.9, 127.5, 127.3, 126.7, 126.3, 125.75, 125.66, 125.1, 123.0, 69.6, 66.2, 46.0, 43.0, 42.9, 42.1, 41.9, 35.6, 28.1, 25.8, 19.49, 19.45, 18.9; **HRMS** (m/z): $[M+H]^+$ calcd. for $C_{40}H_{41}{}^{35}ClN_4O_6$, 709.2793; found, 709.2754.

[*Note: Compound **8r** was observed as a complex mixture of interconverting isomers by ^{1}H and ^{13}C NMR, as confirmed by ROESY NMR. Characterisation was based on 2D NMR experiments, including COSY, HSQC, HMBC and ROESY NMR, as well as X-ray crystallography (see Supplementary Section 6).*]

Benzyl 4-((2-acetamido-4-(3-cyclopropyl-5-((2-fluoro-4-iodophenyl)amino)-6,8-dimethyl-2,4,7-trioxo-3,4,6,7-tetrahydropyrido[4,3-*d*]pyrimidin-1(2*H*)-yl)phenyl)carbamoyl)piperidine-1-carboxylate (8s)

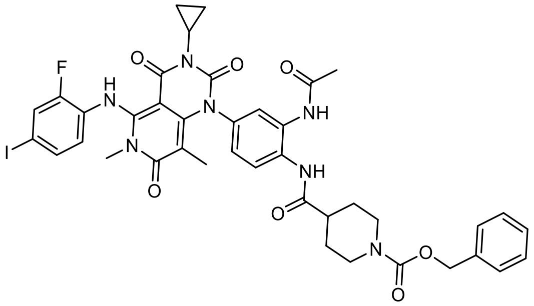

Prepared according to **General procedure A**, using *N*-(3-(3-cyclopropyl-5-((2-fluoro-4-iodophenyl)amino)-6,8-dimethyl-2,4,7-trioxo-3,4,6,7-tetrahydropyrido[4,3-*d*]pyrimidin-1(2*H*)-yl)phenyl)acetamide (*Trametinib,* 154 mg, 0.25 mmol) and benzyl 4-(5-oxo-1,4,2-dioxazol-3-yl)piperidine-1-carboxylate **4** (83.7 mg, 0.28 mmol) as substrates. The crude reaction mixture was analysed by LCMS using acidic mobile phase and the UV chromatogram is shown below:

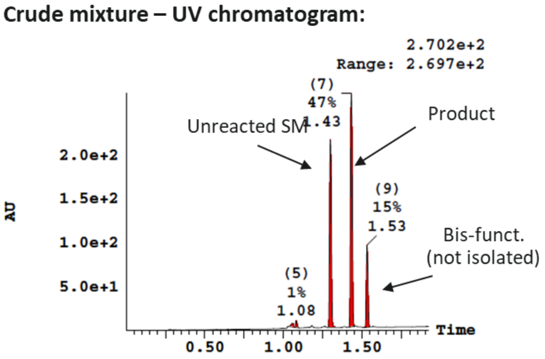

Supplementary Figure 39. LCMS UV chromatogram of the crude reaction mixture forming product **8s**.

Purification by preparative reverse phase HPLC (35-75% MeCN in HCO_2H buffer, 254 nm) afforded amidated derivative **8s** as a white solid (104 mg, 47%). **^{1}H NMR** (500 MHz, DMSO-d_6) δ (ppm) 11.08 (s, 1 H), 9.50 (s, 1 H), 9.38 (s, 1 H), 7.78 (dd, *J* = 10.3, 1.9 Hz, 1 H), 7.65 (d, *J* = 8.7 Hz, 1 H), 7.54 (dd, *J* = 8.3, 1.9 Hz, 1 H), 7.47 (d, *J* = 2.4 Hz, 1 H), 7.41–7.35 (m, 4 H), 7.35–7.29 (m, 1 H), 7.16 (dd, *J* = 8.7, 2.5 Hz, 1 H), 6.92 (t, *J* = 8.6 Hz, 1 H), 5.09 (s, 2 H), 4.07 (dt, *J* = 13.5, 3.7 Hz, 2 H), 3.07 (s, 3 H), 3.02–2.78 (br m, 2 H), 2.70–2.57 (m, 2 H), 2.06 (s, 3 H), 1.87 (br d, *J* = 12.8 Hz, 2 H), 1.54 (qd, *J* = 12.4, 4.2 Hz, 2 H), 1.29 (s, 3 H), 1.02–0.89 (m, 2 H), 0.73–0.62 (m, 2 H); **^{13}C NMR** (126 MHz, DMSO-d_6) δ (ppm) 173.1, 168.9, 164.2, 162.9, 154.4, 154.1 (d, J_{CF} = 250.5 Hz), 151.1, 150.8, 144.8, 137.0, 136.0, 134.0 (d, J_{CF} = 3.6 Hz), 130.3, 130.1, 128.5 (2 C), 128.2 (d, J_{CF} = 11.3 Hz), 127.8, 127.6 (2 C), 126.0, 125.8, 125.1–124.7 (m, 2 C), 124.3, 101.9, 90.3, 88.2 (d, J_{CF} = 7.3 Hz), 66.2, 43.1 (2 C), 42.1, 34.0, 28.2 (br, 2 C), 24.9, 23.6, 13.2, 8.2 (2 C); **^{19}F NMR** (471 MHz, DMSO-d_6) δ (ppm) -124.4 (s, 1 F); **HRMS** (m/z): $[M+H]^+$ calcd. for $C_{40}H_{39}F^{127}IN_7O_7$, 876.2018; found, 876.2058.

2-(4-(2-(2-(1-((Benzyloxy)carbonyl)piperidine-4-carboxamido)-4-chlorobenzamido)ethyl)phenoxy)-2-methylpropanoic acid (8t)

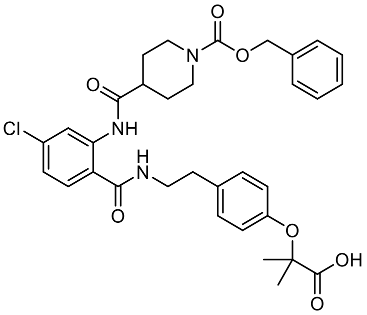

Prepared according to **General procedure A**, using 2-(4-(2-(4-chlorobenzamido)ethyl)phenoxy)-2-methylpropanoic acid (*Bezafibrate,* 90.5 mg, 0.25 mmol) and benzyl 4-(5-oxo-1,4,2-dioxazol-3-yl)piperidine-1-carboxylate **4** (83.7 mg, 0.28 mmol) as substrates. The crude reaction mixture was analysed by LCMS using acidic mobile phase and the UV chromatogram is shown below:

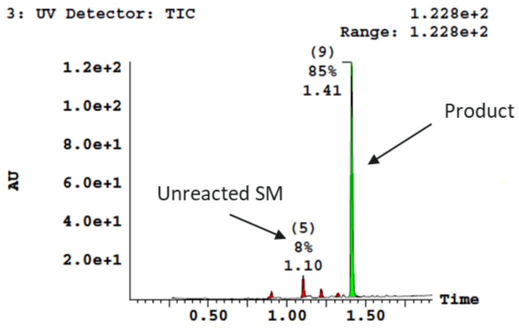

Supplementary Figure 40. LCMS UV chromatogram of the crude reaction mixture forming product **8t**.

Purification by preparative reverse phase HPLC (35-80% MeCN in HCO_2H buffer, 254 nm) afforded amidated derivative **8t** as a white solid (126 mg, 81%). **^{1}H NMR** (500 MHz, DMSO-d_6) δ (ppm) 13.00 (br s, 1 H), 11.62 (s, 1 H), 8.93 (t, *J* = 5.5 Hz, 1 H), 8.55 (d, *J* = 2.2 Hz, 1 H), 7.71 (d, *J* = 8.5 Hz, 1 H), 7.42–7.27 (m, 5 H), 7.22 (dd, *J* = 8.5, 2.2 Hz, 1 H), 7.12 (d, *J* = 8.2 Hz, 2 H), 6.76 (d, *J* = 8.2 Hz, 2 H), 5.08 (s, 2 H), 4.05 (dt, *J* = 13.4, 3.6 Hz, 2 H), 3.46 (q, *J* = 7.2 Hz, 2 H), 3.04–2.83 (br m, 2 H), 2.78 (t, *J* = 7.3 Hz, 2 H), 2.59–2.50 (m, part. overlap with solvent signal, 1 H), 1.93–1.77 (m, 2 H), 1.58–1.40 (s, 8 H); **^{13}C NMR** (126 MHz, DMSO-d_6) δ (ppm) 175.1, 173.0, 167.4, 154.4, 153.7, 140.3, 137.0, 136.4, 132.4, 129.7, 129.4 (2 C), 128.4 (2 C), 127.8, 127.5 (2 C), 122.3, 119.6, 119.0, 118.5 (2 C), 78.3, 66.2, 43.3 (2 C), 42.9, 40.9, 33.9, 28.0 (br, 2 C), 25.1 (2 C); **HRMS** (m/z): $[M+H]^+$ calcd. for $C_{33}H_{36}{}^{35}ClN_3O_7$, 622.232; found, 622.2322.

(2a*R*,4*S*,4a*S*,6*R*,9*S*,11*S*,12*S*,12a*R*,12b*S*)-12-(Benzoyloxy)-9-(((2*R*,3*S*)-3-(2-(1-((benzyloxy)carbonyl)piperidine-4-carboxamido)benzamido)-2-hydroxy-3-phenylpropanoyl)oxy)-4,11-dihydroxy-4a,8,13,13-tetramethyl-5-oxo-3,4,4a,5,6,9,10,11,12,12a-decahydro-1*H*-7,11-methanocyclodeca[3,4]benzo[1,2-*b*]oxete-6,12b(2a*H*)-diyl diacetate (8u) and (2a*R*,4*S*,4a*S*,6*R*,9*S*,11*S*,12*S*,12a*R*,12b*S*)-9-(((2*R*,3*S*)-3-benzamido-2-(((1-((benzyloxy)carbonyl)piperidin-4-yl)carbamoyl)oxy)-3-phenylpropanoyl)oxy)-12-(benzoyloxy)-4,11-dihydroxy-4a,8,13,13-tetramethyl-5-oxo-3,4,4a,5,6,9,10,11,12,12a-decahydro-1*H*-7,11-methanocyclodeca[3,4]benzo[1,2-*b*]oxete-6,12b(2a*H*)-diyl diacetate (28)

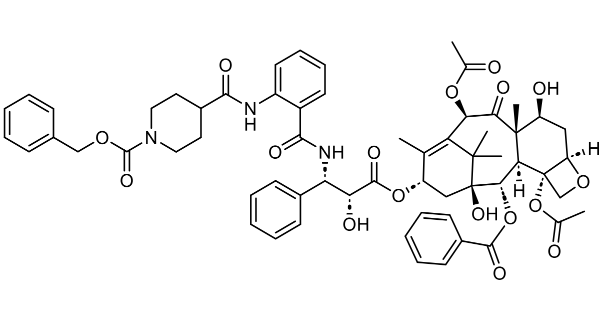

8u

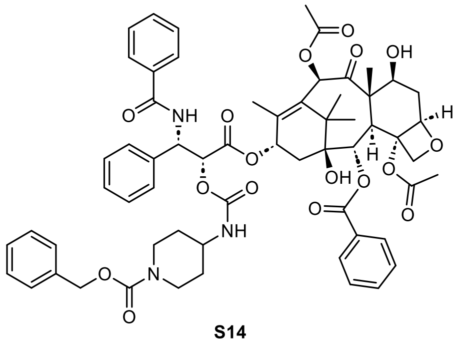

S14

Prepared according to **General procedure B**, (2a*R*,4*S*,4a*S*,6*R*,9*S*,11*S*,12*S*,12a*R*,12b*S*)-9-(((2*R*,3*S*)-3-benzamido-2-hydroxy-3-phenylpropanoyl)oxy)-12-(benzoyloxy)-4,11-dihydroxy-4a,8,13,13-tetramethyl-5-oxo-3,4,4a,5,6,9,10,11,12,12a-decahydro-1*H*-7,11-methanocyclodeca[3,4]benzo[1,2-*b*]oxete-6,12b(2a*H*)-diyl diacetate (*Paclitaxel,* 128 mg, 0.15 mmol) and benzyl 4-(5-oxo-1,4,2-dioxazol-3-yl)piperidine-1-carboxylate **4** (54.8 mg, 0.18 mmol) as substrate, PivOH (4.60 mg, 0.05 mmol) in place of $(PhO)_2PO_2H$, and a 9:1 mixture of DME:DMA (0.1 M, 1.50 mL) as solvent. The crude reaction mixture was analysed by LCMS using acidic mobile phase and the UV chromatogram is shown below:

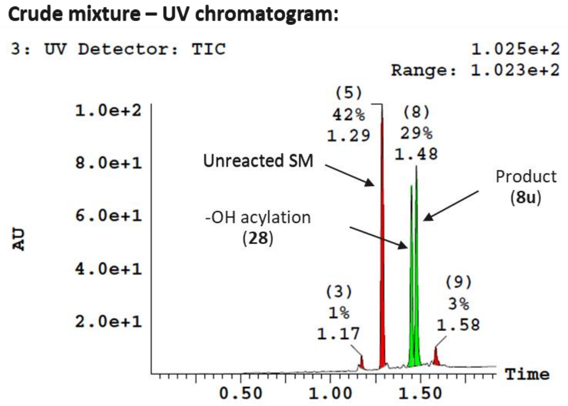

Supplementary Figure 41. LCMS UV chromatogram of the crude reaction mixture forming products **8u** and **28**.

Purification by preparative reverse phase HPLC (35-75% MeCN in HCO_2H buffer, 240 nm) afforded C–H amidated derivative **8u** (31.8 mg, 19%), followed by -OH acylation product **28** (37.3 mg, 22%) as white solids.

8u: ^{1}**H NMR** (500 MHz, DMSO-*d*$_6$) δ (ppm) 10.89 (s, 1 H), 9.28 (d, *J* = 8.4 Hz, 1 H), 8.35 (d, *J* = 8.3 Hz, 1 H), 7.95 (d, *J* = 7.7 Hz, 2 H), 7.84 (d, *J* = 7.2 Hz, 1 H), 7.72 (t, *J* = 7.3 Hz, 1 H), 7.63 (t, *J* = 7.6 Hz, 2 H), 7.52 (t, *J* = 8.6 Hz, 1 H), 7.46–7.27 (m, 10 H), 7.26–7.17 (m, 2 H), 6.50–6.17 (br m, 2 H), 5.88 (t, *J* = 9.1 Hz, 1 H), 5.41 (d, *J* = 7.1 Hz, 1 H), 5.33 (t, *J* = 8.5 Hz, 1 H), 5.08 (s, 2 H), 4.90 (d, *J* = 10.7 Hz, 1 H), 4.69 (br s, 1 H), 4.55 (d, *J* = 8.6 Hz, 1 H), 4.09 (dd, *J* = 10.8, 6.8 Hz, 1 H), 4.05–3.91 (m, 4 H), 3.59 (d, *J* = 7.2 Hz, 1 H), 2.98–2.73 (br m, 2 H), 2.50–2.43 (m, part. overlap with solvent signal, 1 H), 2.31 (ddd, *J* = 15.3, 9.4, 6.0 Hz, 1 H), 2.16 (s, 3 H), 2.11 (s, 3 H), 1.86–1.69 (m, 6 H), 1.69–1.58 (dd, *J* = 14.9, 8.3 Hz, 2 H), 1.50 (s, 3 H), 1.47–1.34 (m, 2 H), 1.03 (s, 3 H), 1.01 (s,

3 H); **^{13}C NMR** (126 MHz, DMSO-d_6) δ (ppm) 202.4, 172.8, 172.6, 169.9, 168.8, 168.1, 165.2, 154.4, 139.2, 138.5, 138.4, 137.0, 133.5, 133.4, 131.9, 130.0, 129.6 (2 C), 128.7 (2 C), 128.5 (2 C), 128.4 (2 C), 128.3, 127.9 (2 C), 127.7, 127.6, 127.5 (2 C), 122.9, 121.9, 120.8, 83.6, 80.3, 76.7, 75.4, 74.8, 74.5, 73.6, 70.5, 69.6, 66.2, 57.5, 56.7, 46.1, 43.1, 43.0, 42.9 (2 C), 36.6, 34.6, 28.1 (br, 2 C), 26.3, 22.6, 21.3, 20.7, 13.9, 9.8; **HRMS** (m/z): $[M+H]^+$ calcd. for $C_{61}H_{67}N_3O_{17}$, 1114.4548; found, 1114.4573.

28: ^{1}H NMR (500 MHz, DMSO-d_6, observed as a 8:2 rotameric mixture) δ (ppm) 9.36–9.15 (m, 1 H), 7.97 (d, J = 7.5 Hz, 2 H), 7.88 (d, J = 7.8 Hz, 2 H), 7.77–7.67 (m, 2 H), 7.67–7.61 (m, 2 H), 7.57 (t, J = 7.3 Hz, 1 H), 7.51 (t, J = 7.4 Hz, 2 H), 7.48–7.39 (m, 4 H), 7.39–7.28 (m, 5 H), 7.14 (t, J = 6.9 Hz, 1 H), 6.29 (s, 1 H), 5.89–5.67 (m, 1 H), 5.64–5.34 (m, 2 H), 5.34–5.16 (m, 1 H), 5.06 (s, 2 H), 4.91 (d, J = 9.6 Hz, 1 H), 4.57 (br s, 1 H), 4.18–4.06 (m, 1 H), 4.00 (s, 2 H), 3.95–3.75 (m, 2 H), 3.63–3.53 (m, 1 H), 3.53–3.44 (m, part. overlap with H_2O signal, 1 H), 3.01–2.73 (br s, 2 H), 2.39–2.21 (m, 4 H), 2.10 (s, 3 H), 1.84–1.59 (m, 7 H), 1.49 (s, 3 H), 1.41–1.21 (m, 3 H), 1.02 (s, 3 H), 0.99 (s, 3 H); **^{13}C NMR** (126 MHz, DMSO-d_6, only the signals from the major rotamer are reported) δ (ppm) 202.5, 170.2, 169.8, 168.8, 165.9, 165.2, 154.45, 154.35, 139.8, 137.8, 137.0, 134.3, 133.5, 133.1, 131.6, 129.9, 129.6 (2 C), 128.7 (2 C), 128.5 (2 C), 128.4 (2 C), 128.2, 127.9 (2 C), 127.8, 127.6 (4 C), 127.4 (2 C), 83.7, 80.2, 76.7, 75.3, 74.8, 74.7, 74.5, 70.6, 70.5, 66.2, 57.4, 54.7, 47.8, 46.1, 42.9, 42.4 (2 C), 36.6, 34.3, 31.3 (br, 2 C), 26.3, 22.7, 21.4, 20.7, 14.2, 9.8; **HRMS** (m/z): $[M+H]^+$ calcd. for $C_{61}H_{67}N_3O_{17}$, 1114.4548; found, 1114.4559.

2-(5-(2-(1-((Benzyloxy)carbonyl)piperidine-4-carboxamido)-4-methylbenzoyl)-1-methyl-1*H*-pyrrol-2-yl)acetic acid (8v)

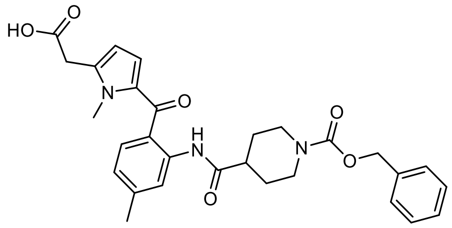

Prepared according to **General procedure A**, using 2-(1-methyl-5-(4-methylbenzoyl)-1H-pyrrol-2-yl)acetic acid (*Tolmetin,* 64.3 mg, 0.25 mmol) and benzyl 4-(5-oxo-1,4,2-dioxazol-3-yl)piperidine-1-carboxylate **4** (83.7 mg, 0.28 mmol) as substrates. The crude reaction mixture was analysed by LCMS using acidic mobile phase and the UV chromatogram is shown below:

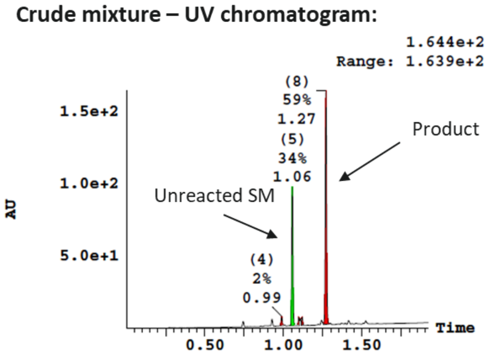

Supplementary Figure 42. LCMS UV chromatogram of the crude reaction mixture forming product **8v**.

An additional aqueous work-up was performed prior to HPLC purification.
After the reaction, the solid residue was removed by filtration through a short plug of Celite eluting with EtOAc (3 x 5 mL). The solvent was then removed under reduced pressure. 1 N NaOH (10 mL) and EtOAc (15 mL) were added, the phases were separated and the organic layer was extracted with 1 N NaOH (4 x 20 mL). The combined aqueous layers were then acidified to pH = 1 with conc. HCl and extracted with EtOAc (3 x 100 mL). The combined organic extracts were washed with brine

(200 mL), dried over anhydrous $MgSO_4$, filtered, and concentrated under reduced pressure. The resulting residue was solubilized in DMSO (3 mL), filtered and purified by preparative reverse phase HPLC (20-60% MeCN in HCO_2H buffer, 240 nm). The product containing fractions were then lyophilised to afford amidated derivative **8v** as a pale brown solid (57.3 mg, 44%). **^{1}H NMR** (500 MHz, DMSO-d_6) δ (ppm) 12.67 (br s, 1 H), 10.03 (s, 1 H), 7.68 (s, 1 H), 7.44 (d, J = 7.9 Hz, 1 H), 7.40–7.28 (m, 5 H), 7.02 (dd, J = 7.9, 1.7 Hz, 1 H), 6.35 (d, J = 4.0 Hz, 1 H), 6.05 (d, J = 4.0 Hz, 1 H), 5.07 (s, 2 H), 3.99 (dt, J = 13.3, 3.6 Hz, 2 H), 3.80 (s, 3 H), 3.76 (s, 2 H), 2.94–2.72 (br m, 2 H), 2.42 (tt, J = 11.7, 3.9 Hz, 1 H), 2.34 (s, 3 H), 1.69–1.58 (m, 2 H), 1.36 (qd, J = 12.4, 4.3 Hz, 2 H); **^{13}C NMR** (126 MHz, DMSO-d_6) δ (ppm) 184.8, 172.6, 170.9, 154.4, 141.3, 137.0, 136.8, 136.6, 131.0, 130.6, 128.4 (2 C), 127.9, 127.8, 127.5 (2 C), 124.2, 123.4, 121.5, 109.4, 66.1, 43.0 (2 C), 42.3, 32.9, 32.2, 27.9 (br, 2 C), 21.2; **HRMS** (m/z): $[M+H]^+$ calcd. for $C_{29}H_{31}N_3O_6$, 518.2291; found, 518.2299.

Benzyl 4-((5-chloro-2-(4-((1-*iso*-propoxy-2-methyl-1-oxopropan-2-yl)oxy)benzoyl)phenyl)carbamoyl)piperidine-1-carboxylate (8x) and benzyl 4-((2-(4-chlorobenzoyl)-5-((1-*iso*-propoxy-2-methyl-1-oxopropan-2-yl)oxy)phenyl)carbamoyl)piperidine-1-carboxylate (8y)

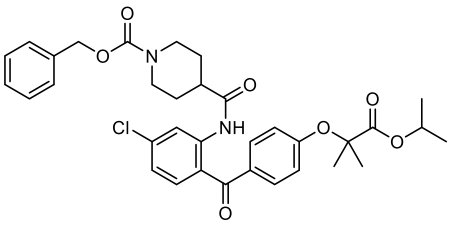

8x

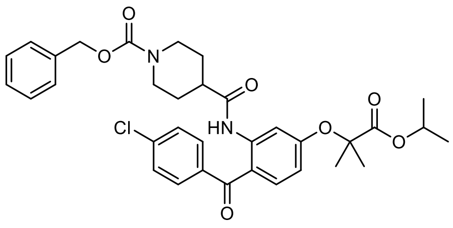

8y

Prepared according to **General procedure A**, using *iso*-propyl 2-(4-(4-chlorobenzoyl)phenoxy)-2-methylpropanoate (*Fenofibrate,* 90.2 mg, 0.25 mmol) and benzyl 4-(5-oxo-1,4,2-dioxazol-3-yl)piperidine-1-carboxylate **4** (83.7 mg, 0.28 mmol) as substrates. The crude reaction mixture was analysed by LCMS using acidic mobile phase and the UV chromatogram is shown below:

Crude mixture – UV chromatogram:

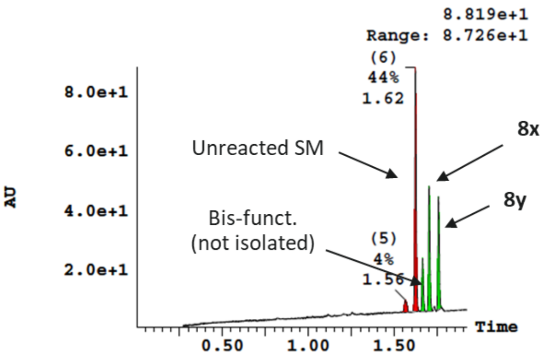

Supplementary Figure 43. LCMS UV chromatogram of the crude reaction mixture forming products **8x** and **8y**.

Purification by preparative reverse phase HPLC (50-90% MeCN in HCO_2H buffer, 240 nm) afforded amidated derivative **8x** (29.5 mg, 19%), followed by derivative **8y** (26.9 mg, 17%) as white solids.

8x: ^{1}H NMR (500 MHz, DMSO-d_6) δ (ppm) 10.05 (s, 1 H), 7.66 (d, J = 2.0 Hz, 1 H), 7.56 (d, J = 8.5 Hz, 2 H), 7.43 (d, J = 8.3 Hz, 1 H), 7.40–7.28 (m, 6 H), 6.80 (d, J = 8.6 Hz, 2 H), 5.05 (s, 2 H), 4.95 (h, J = 6.3 Hz, 1 H), 3.90 (d, J = 13.1 Hz, 2 H), 2.87–2.64 (br m, 2 H), 2.31 (tt, J = 11.6, 3.8 Hz, 1 H), 1.54 (s, 6 H), 1.43 (br s, 2 H), 1.24–1.09 (d, J = 6.3 Hz, 8 H); **^{13}C NMR** (126 MHz, DMSO-d_6) δ (ppm) 192.7, 172.6, 172.1, 159.1, 154.3, 137.4, 137.0, 135.6, 131.7, 131.5 (2 C), 130.1, 129.7, 128.4 (2 C), 127.8, 127.5 (2 C), 124.3, 123.2, 116.9 (2 C), 78.9, 68.9, 66.2, 42.8 (2 C), 41.7, 27.5 (br, 2 C), 24.9 (2 C), 21.2 (2 C); **HRMS** (m/z): $[M+H]^+$ calcd. for $C_{34}H_{37}{}^{35}ClN_2O_7$, 621.2368; found, 621.2366.

8y: **^{1}H NMR** (500 MHz, DMSO-d_6) δ (ppm) 10.54 (s, 1 H), 7.60 (d, *J* = 8.3 Hz, 2 H), 7.56 (d, *J* = 8.4 Hz, 2 H), 7.44 (d, *J* = 2.6 Hz, 1 H), 7.42–7.26 (m, 6 H), 6.61 (dd, *J* = 8.8, 2.6 Hz, 1 H), 5.07 (s, 2 H), 4.98 (h, *J* = 6.3 Hz, 1 H), 4.02–2.89 (m, 2 H), 2.95–2.70 (m, 2 H), 2.41 (tt, *J* = 11.7, 3.8 Hz, 1 H), 1.68–1.52 (m, 8 H), 1.31 (qd, *J* = 12.2, 4.1 Hz, 2 H), 1.15 (d, *J* = 6.3 Hz, 6 H); **^{13}C NMR** (126 MHz, DMSO-d_6) δ (ppm) 194.6, 172.8, 172.0, 158.9, 154.3, 139.6, 137.0 (2 C), 136.9, 133.4, 131.1 (2 C), 128.43 (2 C), 128.37 (2 C), 127.8, 127.5 (2 C), 120.4, 112.6, 110.3, 79.1, 69.0, 66.2, 42.8 (2 C), 42.4, 27.7 (br, 2 C), 25.1 (2 C), 21.2 (2 C); **HRMS** (m/z): $[M+H]^+$ calcd. for $C_{34}H_{37}{}^{35}ClN_2O_7$, 621.2368; found, 621.2391.

9.4 Late-stage linker installation (10a-10k) and derivatisation (11, 12)

3-(4-(2-(6-((((9*H*-Fluoren-9-yl)methoxy)carbonyl)amino)hexanamido)phenyl)-5-phenyloxazol-2-yl)propanoic acid (10a)

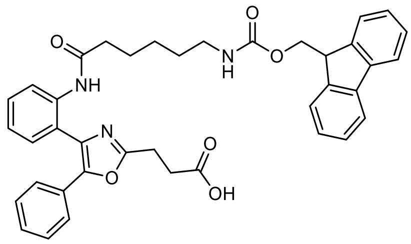

Prepared according to **General procedure B**, using 3-(4,5-diphenyloxazol-2-yl)propanoic acid (*Oxaprozin*, 73.3 mg, 0.25 mmol) and (9*H*-fluoren-9-yl)methyl (5-(5-oxo-1,4,2-dioxazol-3-yl)pentyl)carbamate **9a** (98.6 mg, 0.25 mmol) as substrates. The crude reaction mixture was analysed by LCMS using acidic mobile phase and the UV chromatogram is shown below:

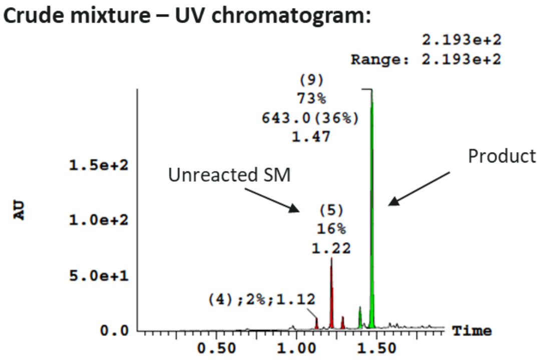

Supplementary Figure 44. LCMS UV chromatogram of the crude reaction mixture forming product **10a**.

Purification by preparative reverse phase HPLC (40-85% MeCN in HCO_2H buffer, 254 nm) afforded amidated derivative **10a** as a white solid (127 mg, 79%). **^{1}H NMR** (500 MHz, DMSO-*d*$_6$) δ (ppm) 12.42 (br s, 1 H), 9.13 (s, 1 H), 7.97 (d, *J* = 8.3 Hz, 1 H), 7.88 (d, *J* = 7.6 Hz, 2 H), 7.68 (d, *J* = 7.5 Hz, 2 H), 7.45–7.27 (m, 11 H), 7.24 (t, *J* = 5.8 Hz, 1 H), 7.15 (t, *J* = 7.5 Hz, 1 H), 4.29 (d, *J* = 7.0 Hz, 2 H), 4.20 (t, *J* = 6.9 Hz, 1 H), 3.10 (t, *J* = 7.1 Hz, 2 H), 2.92 (q, *J* = 6.6 Hz, 2 H), 2.80 (t, *J* = 7.1 Hz, 2 H), 1.89 (t, *J* = 7.4 Hz, 2 H), 1.36–1.27 (m, 4 H), 1.10 (p, *J* = 7.8 Hz, 2 H); **^{13}C NMR** (126 MHz, DMSO-*d*$_6$) δ (ppm) 173.2, 170.9, 162.3, 156.1, 145.4, 144.0 (2 C), 140.8 (2 C), 136.2, 131.6, 130.1, 128.8, 128.7 (2 C), 128.4, 128.2, 127.6 (2 C), 127.1 (2 C), 125.2 (2 C), 125.1 (2 C) 123.9, 123.5, 123.4, 120.1 (2 C), 65.2, 46.8, 40.1, 35.8, 30.3, 29.2, 25.9, 24.5, 23.0; **HRMS** (m/z): $[M+H]^+$ calcd. for $C_{39}H_{37}N_3O_6$, 644.2761; found, 644.2800.

(*E*)-3-(4-(((Benzyloxy)carbonyl)amino)butanamido)-2-(3-(3,4-dimethoxyphenyl)acrylamido)benzoic acid (10b)

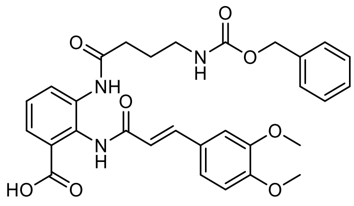

Prepared according to **General procedure A**, using (*E*)-2-(3-(3,4-dimethoxyphenyl)acrylamido)benzoic acid (*Tranilast,* 81.8 mg, 0.25 mmol) and benzyl (3-(5-oxo-1,4,2-dioxazol-3-yl)propyl)carbamate **9b** (77.0 mg, 0.28 mmol) as substrates. The crude reaction

mixture was analysed by LCMS using acidic mobile phase and the UV chromatogram is shown below:

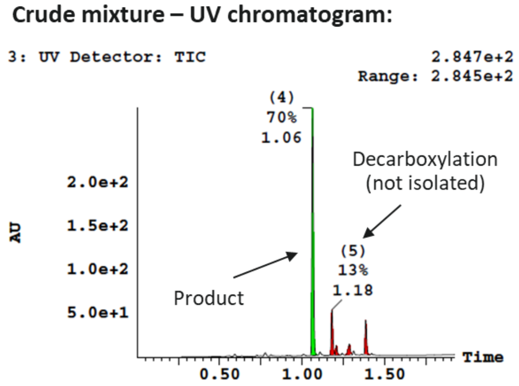

Supplementary Figure 45. LCMS UV chromatogram of the crude reaction mixture forming product **10b**.

Purification by preparative reverse phase HPLC (15-5% MeCN in HCO_2H buffer, 240 nm) afforded amidated derivative **10b** as an off-white solid (68.7 mg, 49%). ^{1}**H NMR** (500 MHz, DMSO-*d*$_6$) δ (ppm) 12.83 (br s, 1 H), 9.65 (s, 1 H), 9.33 (s, 1 H), 7.83 (d, *J* = 8.1 Hz, 1 H), 7.57 (d, *J* = 7.8 Hz, 1 H), 7.50 (d, *J* = 15.6 Hz, 1 H), 7.38–7.27 (m, 7 H), 7.25 (d, *J* = 2.0 Hz, 1 H), 7.19 (dd, J = 8.3, 2.0 Hz, 1H), 7.00 (d, *J* = 8.4 Hz, 1 H), 6.79 (d, *J* = 15.7 Hz, 1 H), 4.98 (s, 2 H), 3.81 (s, 3 H), 3.80 (s, 3 H), 3.06 (q, *J* = 6.5 Hz, 2 H), 2.37 (t, *J* = 7.4 Hz, 2 H), 1.73 (p, *J* = 7.2 Hz, 2 H); ^{13}**C NMR** (126 MHz, DMSO-*d*$_6$) δ (ppm) 171.2, 167.8, 164.8, 156.2, 150.5, 149.0, 140.6, 137.2, 133.5, 129.1, 129.0, 128.4 (2 C), 127.9, 127.78, 127.76 (2 C), 127.4, 126.0, 125.4, 122.0, 119.6, 111.7, 110.1, 65.2, 55.6, 55.5, 39.6, 33.4, 25.5; **HRMS** (m/z): $[M+H]^+$ calcd. for $C_{30}H_{31}N_3O_8$, 562.2189; found, 562.2206.

[*Note: small amount of decarboxylated product was observed by crude LCMS (13% by UV, not isolated)*.]

Methyl ((5*S*,10*S*,11*S*,14*S*)-11-benzyl-8-(3-(4-((*tert*-butoxycarbonyl)amino)butanamido)-4-(pyridin-2-yl)benzyl)-5-(tert-butyl)-10-hydroxy-15,15-dimethyl-3,6,13-trioxo-2-oxa-4,7,8,12-tetraazahexadecan-14-yl)carbamate (10c)

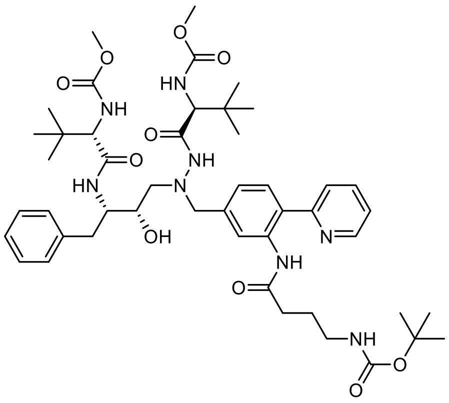

Prepared according to **General procedure B**, using methyl ((5*S*,10*S*,11*S*,14*S*)-11-benzyl-5-(*tert*-butyl)-10-hydroxy-15,15-dimethyl-3,6,13-trioxo-8-(4-(pyridin-2-yl)benzyl)-2-oxa-4,7,8,12-tetraazahexadecan-14-yl)carbamate (*Atazanavir*, 106 mg, 0.15 mmol) and *tert*-butyl (3-(5-oxo-1,4,2-dioxazol-3-yl)propyl)carbamate **9c** (40.3 mg, 0.17 mmol) as substrates. The crude reaction mixture was analysed by LCMS using acidic mobile phase and the UV chromatogram is shown below:

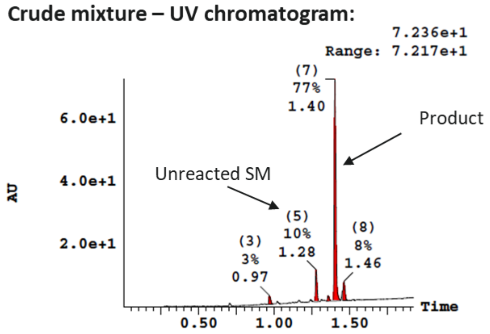

Supplementary Figure 46. LCMS UV chromatogram of the crude reaction mixture forming product **10c**.

Purification by preparative reverse phase HPLC (35-80% MeCN in HCO_2H buffer, 254 nm) afforded amidated derivative **10c** as an off-white solid (95.1 mg, 70%). **^{1}H NMR** (500 MHz, DMSO-*d*$_6$) δ (ppm) 11.96 (s, 1 H), 9.14 (s, 1 H), 8.69 (d, *J* = 5.1 Hz, 1 H), 8.24 (s, 1 H), 7.96 (td, *J* = 7.8, 1.8 Hz, 1 H), 7.85 (d, *J* = 8.2 Hz, 1 H), 7.70 (d, *J* = 8.1 Hz, 1 H), 7.55 (d, *J* = 9.1 Hz, 1 H), 7.41 (dd, *J* = 7.5, 4.9 Hz, 1 H), 7.24 (d, *J* = 8.2 Hz, 1 H), 7.22–7.16 (m, 4 H), 7.15–7.07 (m, 1 H), 6.99 (d, *J* = 9.4 Hz, 1 H), 6.92–6.81 (m, 2 H), 5.01 (s, 1 H), 4.06–3.87 (m, 3 H), 3.83 (d, *J* = 9.4 Hz, 1 H), 3.64 (d, *J* = 9.4 Hz, 1 H), 3.62–3.55 (m, 1 H), 3.56–3.46 (m, 6 H), 2.95 (q, *J* = 6.6 Hz, 2 H), 2.84–2.67 (m, 3 H), 2.67–2.57 (m, 1 H), 2.31 (t, *J* = 7.6 Hz, 2 H), 1.70 (p, *J* = 7.3 Hz, 2 H), 1.36 (s, 9 H), 0.74 (s, 9 H), 0.62 (s, 9 H); **^{13}C NMR** (126 MHz, DMSO-*d*$_6$) δ (ppm) 170.4, 170.2, 170.0, 157.1 (2 C), 156.5, 155.6, 147.8, 139.3, 139.0, 138.1, 137.0, 129.1 (2 C), 128.9, 128.0 (2 C), 125.8, 124.9, 123.6, 122.9, 122.2, 121.8, 77.5, 68.0, 63.1, 61.2, 61.1, 60.8, 51.7, 51.4 (2 C), 39.4, 37.7, 34.7, 33.6, 33.4, 28.3 (3 C), 26.7 (3 C), 26.3 (3 C), 25.5; **HRMS** (m/z): $[M+H]^+$ calcd. for $C_{47}H_{68}N_3O_{10}$, 905.5137; found, 905.5148.

N-(5-Sulfamoyl-2-(5-(*p*-tolyl)-3-(trifluoromethyl)-1*H*-pyrazol-1-yl)phenyl)azetidine-3-carboxamide (10d)

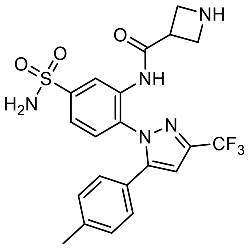

Prepared according to **General procedure A**, using 4-(5-(*p*-tolyl)-3-(trifluoromethyl)-1*H*-pyrazol-1-yl)benzenesulfonamide (*Celecoxib*, 95.3 mg, 0.25 mmol) and *tert*-butyl 3-(5-oxo-1,4,2-dioxazol-3-yl)azetidine-1-carboxylate **9d** (66.6 mg, 0.28 mmol) as substrates. The crude reaction mixture was analysed by LCMS using acidic mobile phase and the UV chromatogram is shown below:

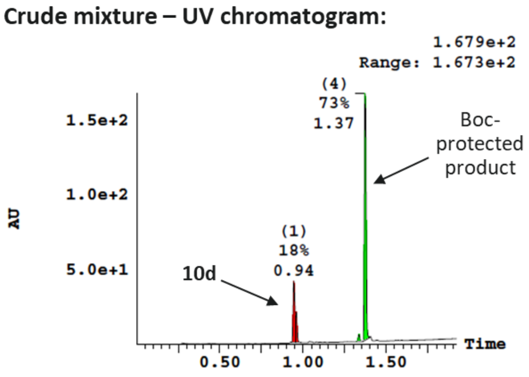

Supplementary Figure 47. LCMS UV chromatogram of the crude reaction mixture forming product **10d**.

After the reaction, 2,2,2-trifluoroacetic acid (0.38 mL, 5.00 mmol) was added by syringe and the mixture was stirred at rt for 5 h. The solvent was then removed under reduced pressure and the resulting residue was solubilized in DMSO (3 mL), filtered and purified by preparative reverse phase HPLC (20-65% MeCN in NH_4HCO_3 buffer, 254 nm), affording amidated derivative **10d** as an off-white solid after lyophilisation (105 mg, 88%). **^{1}H NMR** (500 MHz, CD_3CN) δ (ppm) 8.72 (s, 1 H), 7.57 (dd, *J* = 8.4, 2.1 Hz, 1 H), 7.31 (d, *J* = 8.3 Hz, 1 H), 7.14 (s, 4 H), 6.97 (s, 1 H), 3.63 (t, *J* = 8.0 Hz, part. overlap with H_2O signal, 2 H), 3.51 (t, *J* = 6.7 Hz, part. overlap with H_2O signal, 2 H), 3.28 (t, *J* = 7.4 Hz, 1 H), 2.29 (s, 3 H); **^{13}C NMR** (126 MHz, CD_3CN) δ (ppm) 173.2, 147.8, 145.5, 144.7 (q, J_{CF} = 38.1 Hz), 140.8, 135.9, 133.1, 130.4 (2 C), 130.0, 129.2 (2 C), 126.3, 122.6, 122.4 (q, J_{CF} = 268.3 Hz), 122.0, 106.3 (q, J_{CF} = 2.0 Hz), 50.1 (2 C), 41.4, 21.3; **^{19}F NMR** (471 MHz, CD_3CN) δ (ppm) -62.9 (s, 3 F); **HRMS** (m/z): $[M+H]^+$ calcd. for $C_{21}H_{20}F_3N_5O_3S$, 480.1317; found, 480.1299.

[*Note: partial Boc deprotection was observed after C–H amidation (approx. 20% by UV). One-pot treatment with trifluoroacetic acid led to full conversion to the free NH azetidine product **10d**.*]

2-(4-(2-(2-(2-(1-(*tert*-Butoxycarbonyl)piperidin-4-yl)acetamido)-4-chlorobenzamido)ethyl)phenoxy)-2-methylpropanoic acid (10e)

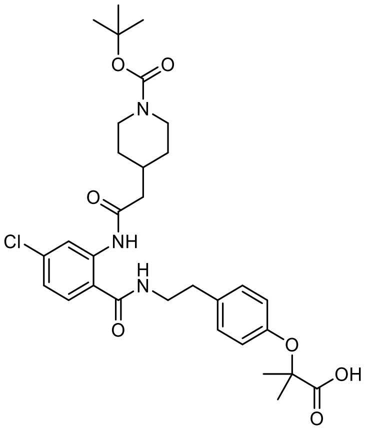

Prepared according to **General procedure A**, using 2-(4-(2-(4-chlorobenzamido)ethyl)phenoxy)-2-methylpropanoic acid (*Bezafibrate,* 90.5 mg, 0.25 mmol) and *tert*-butyl 4-((5-oxo-1,4,2-dioxazol-3-yl)methyl)piperidine-1-carboxylate **9e** (78.1 mg, 0.28 mmol) as substrates. The crude reaction mixture was analysed by LCMS using acidic mobile phase and the UV chromatogram is shown below:

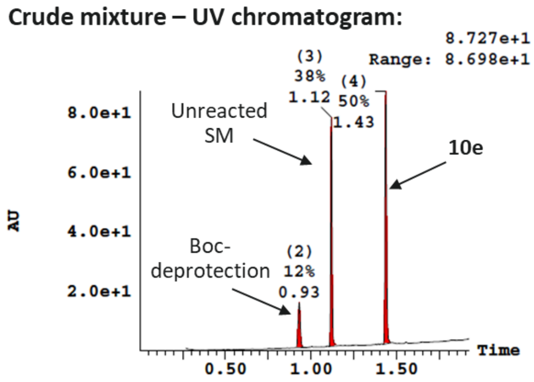

Supplementary Figure 48. LCMS UV chromatogram of the crude reaction mixture forming product **10e**.

After the reaction, di-*tert*-butyl dicarbonate (Boc_2O, 65.5 mg, 0.30 mmol) and *N*-ethyl-*N*-*iso*-propylpropan-2-amine (DIPEA, 65 μl, 0.38 mmol) were added and the mixture was stirred at rt for 1 h. 1H-imidazole (25.5 mg, 0.38 mmol) was added and the reaction was stirred at rt for additional 1 h to quench the excess of Boc_2O. The solvent was removed under reduced pressure and the resulting residue was solubilized in DMSO (3 mL), filtered and purified by preparative reverse phase

HPLC (35-80% MeCN in HCO_2H buffer, 254 nm), affording amidated derivative **10e** as a white solid (76.8 mg, 51%). **^{1}H NMR** (500 MHz, DMSO-d_6) δ (ppm) 13.01 (br s, 1 H), 11.41 (s, 1 H), 8.91 (t, J = 5.6 Hz, 1 H), 8.51 (d, J = 2.2 Hz, 1 H), 7.68 (d, J = 8.5 Hz, 1 H), 7.22 (dd, J = 8.5, 2.2 Hz, 1 H), 7.12 (d, J = 8.2 Hz, 2 H), 6.75 (d, J = 8.5 Hz, 2 H), 4.02–3.78 (br m, 2 H), 3.44 (q, J = 7.5 Hz, 2 H), 2.87–2.57 (br m, 4 H), 2.29 (d, J = 7.1 Hz, 2 H), 1.91 (ttt, J = 10.8, 6.9, 3.3 Hz, 1 H), 1.71–1.60 (m, 2 H), 1.47 (s, 6 H), 1.37 (s, 9 H), 1.08 (qd, J = 12.2, 4.3 Hz, 2 H); **^{13}C NMR** (126 MHz, DMSO-d_6) δ (ppm) 175.1, 170.3, 167.4, 153.8, 153.7, 140.1, 136.2, 132.3, 129.7, 129.4 (2 C), 122.3, 119.7, 119.3, 118.5 (2 C), 78.5, 78.3, 44.1, 43.2 (br, 2 C), 40.9, 33.9, 32.8, 31.3 (br, 2C), 28.1 (3 C), 25.1 (2 C); **HRMS** (m/z): $[M+H]^+$ calcd. for $C_{31}H_{40}{}^{35}ClN_3O_7$, 602.2633; found, 602.2652.

[*Note: partial Boc deprotection was observed after C–H amidation (approx. 10% by UV). One-pot treatment with Boc_2O led to full conversion to the N-Boc piperidine product **10e**.*]

***tert*-Butyl 4-((2-(9-(2-(benzyl(ethyl)amino)-2-oxoethyl)-7-methyl-8-oxo-8,9-dihydro-7*H*-purin-2-yl)phenyl)amino)-4-oxobutanoate (10f)**

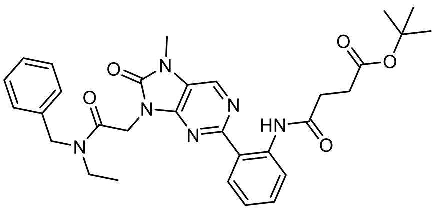

Prepared according to **General procedure B**, using *N*-benzyl-*N*-ethyl-2-(7-methyl-8-oxo-2-phenyl-7,8-dihydro-9*H*-purin-9-yl)acetamide (*Emapunil*, 60.2 mg, 0.15 mmol) and *tert*-butyl 3-(5-oxo-1,4,2-dioxazol-3-yl)propanoate **9f** (35.5 mg, 0.17 mmol) as substrates. The crude reaction mixture was analysed by SFC-MS and the UV chromatogram is shown below:

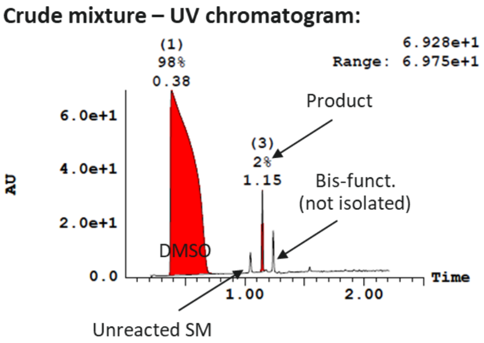

Supplementary Figure 49. LCMS UV chromatogram of the crude reaction mixture forming product **10f**.

Purification by preparative reverse phase HPLC (5-95% MeCN in 10 mM NH_4HCO_3 buffer, 254 nm, XBridge C18 ODB column - 5 μm, 19x150 ID mm) afforded amidated derivative **10f** as an off-white solid (32.5 mg, 38%). **^{1}H NMR** (500 MHz, DMSO-d_6, observed as a 1:1 mixture of E:Z amide isomers) δ (ppm) 12.64 (s, 0.5 H), 12.58 (s, 0.5 H), 8.64 (s, 0.5 H), 8.61 (s, 0.5 H), 8.55–8.49 (m, 1 H), 8.39 (dd, J = 8.0, 1.7 Hz, 0.5 H), 8.33 (dd, J = 8.0, 1.7 Hz, 0.5 H), 7.49–7.34 (m, 3 H), 7.28–7.12 (m, 4 H), 4.95 (s, 1 H), 4.85 (s, 1 H), 4.76 (s, 1 H), 4.52 (s, 1 H), 3.52–3.42 (m, 4 H), 3.29 (q, J = 7.1 Hz, 1 H), 2.64 (t, J = 6.1 Hz, 2 H), 2.58–2.42 (m, 2 H), 1.34 (s, 4.5 H), 1.34 (s, 4.5 H), 1.22 (t, J = 7.0 Hz, 1.5 H), 1.00 (t, J = 7.0 Hz, 1.5 H); **^{13}C NMR** (126 MHz, DMSO-d_6, observed as a 1:1 mixture of E:Z amide isomers) δ (ppm) 171.5 (1 C), 169.9 (1 C), 165.4 and 165.2 (1 C), 156.5 and 156.4 (1 C), 153.00 and 152.98 (1 C), 149.48 and 149.47 (1 C), 138.6 and 138.5 (1 C), 137.7 and 137.2 (1 C), 131.0 and 130.9 (1 C), 130.7 and 130.6 (1 C), 129.7 and 129.6 (1 C), 128.8 and 128.4 (2 C), 127.5 and 127.1 (1 C), 127.4 and 126.6 (2 C), 123.0 and 122.8 (1 C), 122.7 and 122.6 (1 C), 121.7 (1 C), 120.3 and 120.2 (1 C), 79.8 (1 C), 49.1 and 48.0 (1 C), 41.5 and 40.9 (1 C), 41.22 and 41.18 (1 C), 32.3 (1 C), 30.0 (1 C), 27.7 (3 C), 27.50 and 27.46 (1 C), 13.8 and 12.6 (1 C). **HRMS** (m/z): $[M+H]^+$ calcd. for $C_{31}H_{36}N_6O_5$, 573.2825; found, 573.2834.

(±)-2-(3-((5-(7-Chloro-5-hydroxy-2,3,4,5-tetrahydro-1*H*-benzo[*b*]azepine-1-carbonyl)-4-methyl-2-(2-methylbenzamido)phenyl)carbamoyl)bicyclo[1.1.1]pentan-1-yl)acetic acid (10g)

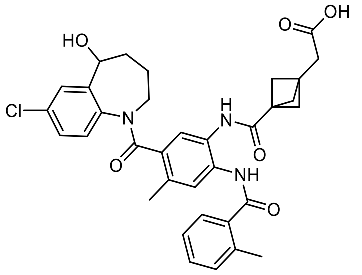

Prepared according to **General procedure B**, using (±)-*N*-(4-(7-chloro-5-hydroxy-2,3,4,5-tetrahydro-1*H*-benzo[*b*]azepine-1-carbonyl)-3-methylphenyl)-2-methylbenzamide (*Tolvaptan,* 112 mg, 0.25 mmol) and *tert*-butyl 2-(3-(5-oxo-1,4,2-dioxazol-3-yl)bicyclo[1.1.1]pentan-1-yl)acetate **9g** (73.5 mg, 0.28 mmol) as substrates, and PivOH (7.66 mg, 0.08 mmol) in place of $(PhO)_2PO_2H$. The crude reaction mixture was analysed by LCMS using acidic mobile phase and the UV chromatogram is shown below:

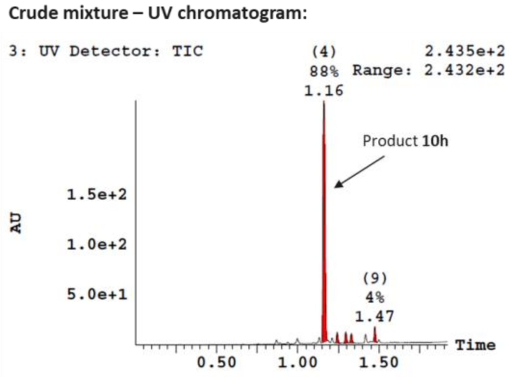

Supplementary Figure 50. LCMS UV chromatogram of the crude reaction mixture forming product **10g**.

Purification by preparative reverse phase HPLC (15-55% MeCN in HCO_2H buffer, 240 nm) afforded amidated derivative **10g** as a white solid (131 mg, 85%). **^{1}H NMR** (500 MHz, DMSO-d_6) δ (ppm) 12.14 (br s, 1 H), 10.12–9.73 (m, 1 H), 9.29–8.81 (m, 1 H), 7.68–7.20 (m, 8 H), 7.11–6.71 (m, 1 H), 5.92–5.01 (br m, 1 H), 4.93–4.53 (m, 2 H), 2.75–2.61 (m, 1 H), 2.48–2.19 (m, 8 H), 2.16–2.05 (m, 1 H), 2.05–1.87 (m, 7 H), 1.83–1.57 (m, 1 H), 1.57–1.39 (m, 1 H); **^{13}C NMR** (126 MHz, DMSO-d_6, only major peaks are reported) δ (ppm) 172.18, 172.16, 169.2, 168.4, 168.3, 168.0, 167.9, 167.7, 144.9, 138.2, 136.4, 136.3, 135.4, 133.9, 131.7, 131.4, 130.8, 130.7, 130.13, 130.09, 129.6, 127.7, 127.3, 126.9, 126.8, 126.6, 125.84, 125.77, 125.2, 69.6, 51.7, 51.6, 46.1, 40.2, 36.52, 36.5, 35.6, 35.2, 35.1, 25.8, 19.51, 19.49, 18.9; **HRMS** (m/z): $[M+H]^+$ calcd. for $C_{34}H_{34}{}^{35}ClN_3O_6$, 616.2214; found, 616.2225.

[*Note 1: Full in-situ deprotection of the tert-butyl ester was observed at the end of the reaction, forming exclusively free acid product* ***10g***.]

[*Note 2: Compound* ***10g*** *was observed as a complex mixture of interconverting isomers by* ^{1}*H and* ^{13}*C NMR, as confirmed by ROESY NMR. Characterisation was based on 2D NMR experiments, including COSY, HSQC, HMBC and ROESY NMR, as well as on analogy with analogue derivative* ***8r*** *(see Supplementary Section 6)*.]

Ethyl 5-((2-acetamido-4-(3-cyclopropyl-5-((2-fluoro-4-iodophenyl)amino)-6,8-dimethyl-2,4,7-trioxo-3,4,6,7-tetrahydropyrido[4,3-*d*]pyrimidin-1(2*H*)-yl)phenyl)amino)-5-oxopentanoate – NH_3 salt (10i)

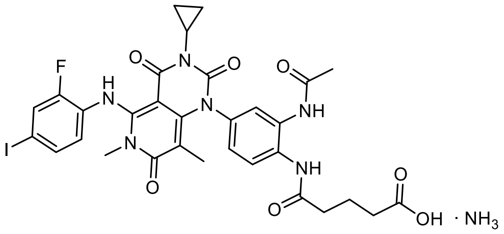

Prepared according to **General procedure A**, using *N*-(3-(3-cyclopropyl-5-((2-fluoro-4-iodophenyl)amino)-6,8-dimethyl-2,4,7-trioxo-3,4,6,7-tetrahydropyrido[4,3-*d*]pyrimidin-1(2*H*)-yl)phenyl)acetamide (*Trametinib,* 154 mg, 0.25 mmol) and ethyl 4-(5-oxo-1,4,2-dioxazol-3-yl)butanoate **9j** (55.3 mg, 0.28 mmol) as substrates. The crude reaction mixture was analysed by LCMS using acidic mobile phase and the UV chromatogram is shown below:

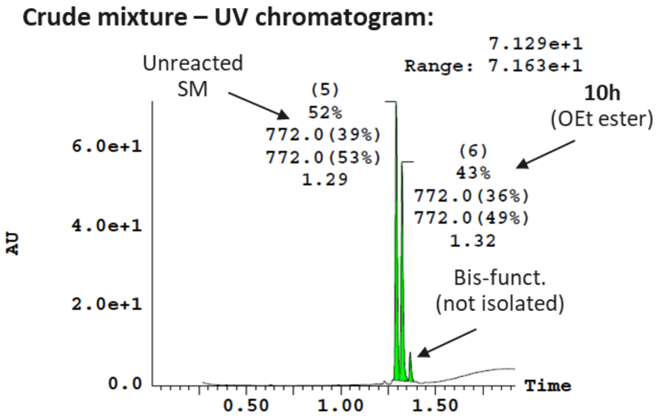

Supplementary Figure 51. LCMS UV chromatogram of the crude reaction mixture forming ethyl ester product **10h**.

After the reaction, the solid material was removed by filtration through a plug of Celite, eluting with EtOAc. The volatiles were removed under reduced pressure and the resulting residue was solubilised in THF (2.50 mL). A solution of NaOH (40.0 mg, 1.00 mmol) in H_2O (2.50 mL) was added dropwise at 0 °C and the reaction was then stirred at rt for 1 h, after which full hydrolysis of the ethyl ester to observed by LCMS. The reaction mixture was diluted with water (5 mL) and EtOAc (10 mL).This was acidified with 1 N HCl to pH ~ 1–2 and extracted with EtOAc (4 x 10 mL). The combined organic layers were dried over $MgSO_4$ and concentrated under vacuum. The residue was resolubilised in DMSO (3 mL) and purified by preparative reverse phase HPLC (20-60% MeCN in NH_4HCO_3 buffer, 254 nm), affording amidated derivative **10i** as a white solid after lyophilisation (ammonium salt, 58.7 mg, 31%). **^{1}H NMR** (500 MHz, CD_3OD) δ (ppm) 7.79–7.44 (br m, 4 H), 7.33–7.11 (br m, 1 H), 6.85 (br t, *J* = 8.2 Hz, 1 H), 3.19 (br s, 3 H), 2.70 (br s, 1 H), 2.52 (br t, *J* = 7.7 Hz, 2 H), 2.39 (br t, *J* = 7.4 Hz, 2 H), 2.16 (br s, 3 H), 2.09–1.92 (br m, 2 H), 1.42 (br s, 3 H), 1.04 (br s, 2 H), 0.75 (br s, 2 H); **^{13}C NMR** (126 MHz, CD_3OD) δ (ppm) 178.2, 174.5, 172.2, 166.1, 165.6, 156.2 (d, J_{CF} = 252.2 Hz), 153.4, 152.9, 147.3, 138.4, 135.6, 132.3, 131.3, 129.6 (d, J_{CF} = 11.6 Hz), 127.6, 126.9, 126.6 (d, J_{CF} = 22.1 Hz), 126.2 (2 C), 104.6, 92.5, 88.7 (d, J_{CF} = 7.1 Hz), 36.8, 35.1 (2 C), 26.2, 23.6, 22.4, 13.8, 9.1 (2 C); **^{19}F NMR** (471 MHz, CD_3OD) δ (ppm) -125.4 (s, 1 F); **HRMS** (m/z): $[M+H]^+$ calcd. for $C_{31}H_{30}F^{127}IN_6O_7$, 745.1283; found, 745.1287.

[*Note: Ethyl ester product **10h** partially coeluted with the unreacted starting material by preparative reverse phase HPLC under both acidic and basic conditions. Telescoped hydrolysis led to full conversion to carboxylate **10i**.*]

Methyl ((5*S*,10*S*,11*S*,14*S*)-8-(3-(3-(1,3-dioxolan-2-yl)propanamido)-4-(pyridin-2-yl)benzyl)-11-benzyl-5-(*tert*-butyl)-10-hydroxy-15,15-dimethyl-3,6,13-trioxo-2-oxa-4,7,8,12-tetraazahexadecan-14-yl)carbamate (10j)

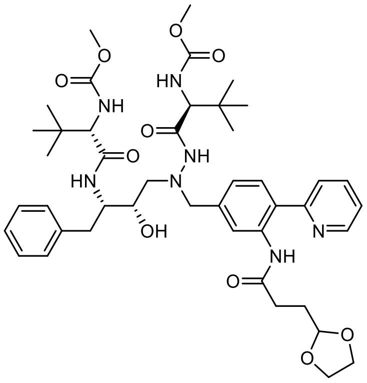

Prepared according to **General procedure B**, using methyl ((5*S*,10*S*,11*S*,14*S*)-11-benzyl-5-(*tert*-butyl)-10-hydroxy-15,15-dimethyl-3,6,13-trioxo-8-(4-(pyridin-2-yl)benzyl)-2-oxa-4,7,8,12-tetraazahexadecan-14-yl)carbamate (*Atazanavir*, 106 mg, 0.15 mmol) and 3-(2-(1,3-dioxolan-2-yl)ethyl)-1,4,2-dioxazol-5-one **9h** (30.9 mg, 0.17 mmol) as substrates. The crude reaction mixture was analysed by LCMS using acidic mobile phase and the UV chromatogram is shown below:

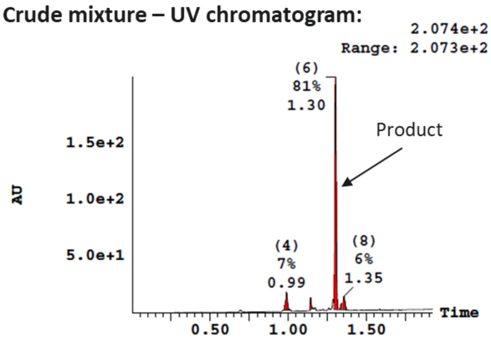

Supplementary Figure 52. LCMS UV chromatogram of the crude reaction mixture forming product **10j**.

Purification by preparative reverse phase HPLC (25-65% MeCN in HCO_2H buffer, 254 nm) afforded amidated derivative **10j** as a white solid (92.3 mg, 73%). **^{1}H NMR** (500 MHz, DMSO-d_6) δ (ppm) 11.90 (s, 1 H), 9.14 (s, 1 H), 8.69 (dd, *J* = 5.1, 1.8 Hz, 1 H), 8.20 (s, 1 H), 7.96 (td, *J* = 7.8, 1.9 Hz, 1 H), 7.84 (d, *J* = 8.1 Hz, 1 H), 7.69 (d, *J* = 8.1 Hz, 1 H), 7.54 (d, *J* = 9.0 Hz, 1 H), 7.41 (dd, *J* = 7.5, 4.9 Hz, 1 H), 7.24 (dd, *J* = 8.1, 1.5 Hz, 1H), 7.22–7.16 (m, 4 H), 7.15–7.09 (m, 1 H), 7.00 (d, *J* = 9.4 Hz, 1 H), 6.86 (d, *J* = 9.4 Hz, 1 H), 5.01 (s, 1 H), 4.83 (t, *J* = 4.6 Hz, 1 H), 4.07–3.88 (m, 3 H), 3.87–3.78 (m, 3 H), 3.78–3.70 (m, 2 H), 3.64 (d, *J* = 9.4 Hz, 1 H), 3.61–3.56 (m, 1 H), 3.56–3.46 (m, 6 H), 2.85–2.67 (m, 3 H), 2.66–2.57 (m, 1 H), 2.39 (t, *J* = 7.5 Hz, 2 H), 1.88 (td, *J* = 7.6, 4.6 Hz, 2 H), 0.74 (s, 9 H), 0.62 (s, 9 H); **^{13}C NMR** (126 MHz, DMSO-d_6) δ (ppm) 170.2, 170.0 (2 C), 157.1, 156.5 (2 C), 147.9, 139.3, 139.0, 138.1, 136.8, 129.1 (2 C), 129.0, 128.0 (2 C), 125.8, 125.2, 123.7, 122.9, 122.2, 122.0, 102.7, 68.0, 64.3 (2 C), 63.1, 61.2, 61.1, 60.8, 51.7, 51.4 (2 C), 37.7, 33.6, 33.4, 31.6, 29.0, 26.7 (3 C), 26.3 (3 C); **HRMS** (m/z): $[M+H]^+$ calcd. for $C_{44}H_{61}N_7O_{10}$, 848.4558; found, 848.4597.

***N*-(2-(5-((4-Aminophenyl)sulfonamido)-1*H*-pyrazol-1-yl)phenyl)pent-4-enamide (10k)**

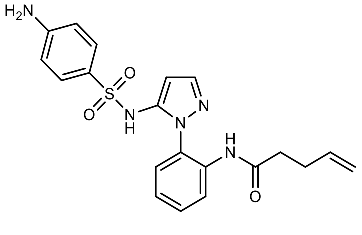

Prepared according to **General procedure A**, using 4-amino-*N*-(1-phenyl-1*H*-pyrazol-5-yl)benzenesulfonamide (*Sulfaphenazole*, 78.6 mg, 0.25 mmol) and 3-(but-3-en-1-yl)-1,4,2-dioxazol-5-one **9i** (38.8 mg, 0.28 mmol) as substrates. The crude reaction mixture was analysed by LCMS using acidic mobile phase and the UV chromatogram is shown below:

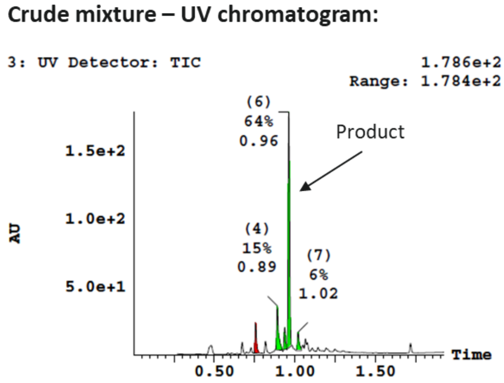

Supplementary Figure 53. LCMS UV chromatogram of the crude reaction mixture forming product **10k**.

Purification by preparative reverse phase HPLC (5-50% MeCN in HCO_2H buffer, 254 nm) afforded amidated derivative **10k** as a white solid (44.5 mg, 43%). **^{1}H NMR** (500 MHz, DMSO-d_6) δ (ppm) 9.81 (br s, 1 H), 8.70 (br s, 1 H), 7.99 (d, *J* = 8.2 Hz, 1 H), 7.62 (d, *J* = 2.0 Hz, 1 H), 7.44 (t, *J* = 7.8 Hz, 1 H), 7.36 (d, *J* = 8.5 Hz, 2 H), 7.20 (t, *J* = 7.6 Hz, 1 H), 7.08 (dd, *J* = 7.9, 1.5 Hz, 1 H), 6.60 (d, *J* = 8.5 Hz, 2 H), 6.10 (br s, 2 H), 5.86 (d, *J* = 2.0 Hz, 1 H), 5.74 (ddt, *J* = 16.7, 10.1, 6.3 Hz, 1 H), 4.99 (dd, *J* = 17.3, 2.0 Hz, 1 H), 4.91 (dd, *J* = 10.2, 2.0 Hz, 1 H), 2.28 (t, *J* = 7.7 Hz, 2 H), 2.20 (q, *J* = 7.1 Hz, 2 H); **^{13}C NMR** (126 MHz, DMSO-d_6) δ (ppm) 170.4, 153.3, 140.3, 137.2, 137.0, 134.2, 129.2, 129.05, 128.99 (2 C), 127.8, 124.2, 124.1, 123.8, 115.4, 112.6 (2 C), 101.0, 35.5, 28.9; **HRMS** (m/z): $[M+H]^+$ calcd. for $C_{20}H_{21}N_5O_3S$, 412.1443; found, 412.1435.

1-(3-(5,5-Difluoro-7,9-dimethyl-5*H*-5λ^4,6λ^4-dipyrrolo[1,2-c:2',1'-*f*][1,3,2]diazaborinin-3-yl)propanoyl)-*N*-(5-sulfamoyl-2-(5-(*p*-tolyl)-3-(trifluoromethyl)-1*H*-pyrazol-1-yl)phenyl)azetidine-3-carboxamide (11)

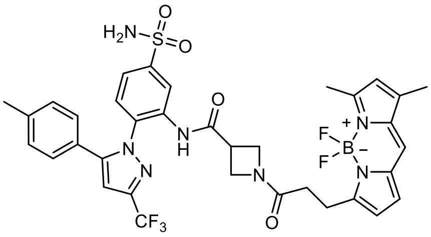

N-Ethyl-*N*-isopropylpropan-2-amine (DIPEA, 4.8 μL, 0.03 mmol) was added to a solution of 2,5-dioxopyrrolidin-1-yl 3-(5,5-difluoro-7,9-dimethyl-5*H*-5λ^4,6λ^4-dipyrrolo[1,2-c:2',1'-*f*][1,3,2]diazaborinin-3-yl)propanoate (9.0 mg, 0.02 mmol) and *N*-(5-sulfamoyl-2-(5-(*p*-tolyl)-3-(trifluoromethyl)-1*H*-pyrazol-1-yl)phenyl)azetidine-3-carboxamide **10d** (13.3 mg, 0.03 mmol) in anhydrous acetonitrile (1.0 mL), and the reaction was stirred for 3 hours in the dark. The volatiles were removed under vaccum and the crude mixture was purified by flash column chromatography (25-100% EtOAc/heptane). The product containing fractions were combined and the solvent removed under vacuum to afford labelled derivative **11** as a red solid (11.7 mg, 67 %). R_f 0.39 (90% EtOAc/heptane); **^{1}H NMR** (500 MHz, DMSO-d_6) δ (ppm) 9.92 (s, 1 H), 8.19 (d, *J* = 2.0 Hz, 1 H), 7.69 (s, 1 H), 7.63 (dd, *J* = 8.4, 2.1 Hz, 1 H), 7.59 (br s, 2 H), 7.39 (d, *J* = 8.4 Hz, 1 H), 7.21 (d, *J* = 8.3 Hz, 2 H), 7.18 (s, 1 H), 7.15 (d, *J* = 7.9 Hz, 2 H), 7.09 (d, *J* = 4.0 Hz, 1 H), 6.36 (d, *J* = 4.0 Hz, 1 H), 6.31 (s, 1 H), 4.14 (t, *J* = 8.5 Hz, 1 H), 3.96–3.85 (m, 2 H), 3.69 (dd, *J* = 9.3, 5.6 Hz, 1 H), 3.41–3.36 (m, 1 H, overlapping with H_2O peak), 3.02 (t, *J* = 7.8 Hz, 2 H), 2.47 (s, 3 H), 2.45–2.37 (m, 2 H), 2.26 (m, 6 H); **^{13}C NMR** (126 MHz, DMSO-d_6) δ (ppm) 170.8, 170.3, 159.3, 157.5, 145.8, 144.7, 144.2, 142.4

(q, J_{CF} = 37.4 Hz), 138.8, 134.5, 133.9, 133.5, 133.0, 129.3 (2 C), 129.2, 128.9, 128.1 (2 C), 125.5, 125.4, 123.2, 122.7, 121.4 (q, J_{CF} = 269.0 Hz), 120.3, 116.9, 105.2, 51.8, 49.8, 32.2, 29.4, 23.2, 20.8, 14.6, 11.0; **^{19}F NMR** (471 MHz, DMSO-*d*$_6$) δ (ppm) -60.8 (s, 3 F), -143.2 (dd, $J_{F\text{-}B}$ = 66.8, 33.1 Hz, 2 F); **^{11}B NMR** (160 MHz, DMSO-*d*$_6$) δ (ppm) 0.78 (t, $J_{F\text{-}B}$ = 33.4 Hz); **HRMS** (m/z): $[M–H]^−$ calcd. for $C_{35}H_{33}BF_5N_7O_4S$, 752.2365; found, 752.2261.

***N*^{1}-(2-Acetamido-4-(3-cyclopropyl-5-((2-fluoro-4-iodophenyl)amino)-6,8-dimethyl-2,4,7-trioxo-3,4,6,7-tetrahydropyrido[4,3-*d*]pyrimidin-1(2*H*)-yl)phenyl)-*N*^{5}-((*S*)-1-((2*S*,4*R*)-4-hydroxy-2-((4-(4-methylthiazol-5-yl)benzyl)carbamoyl)pyrrolidin-1-yl)-3,3-dimethyl-1-oxobutan-2-yl)glutaramide (12)**

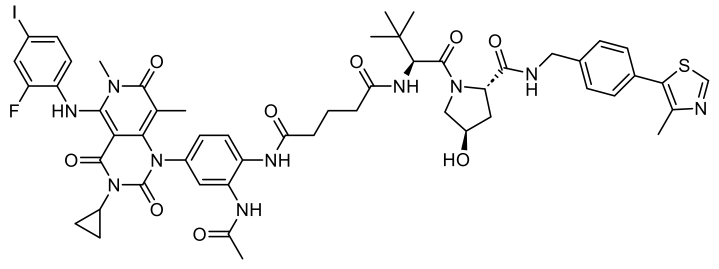

An oven-dried vial was charged with 5-((2-acetamido-4-(3-cyclopropyl-5-((2-fluoro-4-iodophenyl)amino)-6,8-dimethyl-2,4,7-trioxo-3,4,6,7-tetrahydropyrido[4,3-*d*]pyrimidin-1(2*H*)-yl)phenyl)amino)-5-oxopentanoic acid · NH_3 **10g** (19.6 mg, 0.03 mmol), (2*S*,4*R*)-1-((*S*)-2-amino-3,3-dimethylbutanoyl)-4-hydroxy-*N*-(4-(4-methylthiazol-5-yl)benzyl)pyrrolidine-2-carboxamide · 2.2 HCl (14.5 mg, 0.03 mmol) and 2-(3*H*-[1,2,3]triazolo[4,5-b]pyridin-3-yl)-1,1,3,3-tetramethylisouronium hexafluorophosphate(V) (HATU, 11.7 mg, 0.03 mmol). Anhydrous DMF (250 μL) was added, followed by *N*-ethyl-*N*-isopropylpropan-2-amine (DIPEA, 13.5 μL, 0.08 mmol). After stirring at room temperature for 2 h, the mixture was diluted with CH_2Cl_2 (1 mL) and sat. aq. sol. $NaHCO_3$ (1 mL). The phases were separated through phase separator, and the aqueous phase was extracted with further CH_2Cl_2 (3 x 1 mL). The phases were separated through phase separator, and the volatiles were removed under vacuum. The residue was resolubilised in DMSO (2 mL) and purified by preparative reverse phase HPLC (25-70% MeCN in HCO_2H buffer, 254 nm). The product containing fractions were then concentrated by freeze drying to afford VHL conjugate **12** as a white solid (22.0 mg, 74 %). **^{1}H NMR** (500 MHz, $CDCl_3$) δ (ppm) 11.20 (s, 1 H), 9.13–8.77 (m, 2 H), 8.74–7.99 (m, 1 H), 7.64–7.21 (m, 9 H), 7.12–6.83 (m, 1 H), 6.71 (br s, 1 H), 6.62 (t, *J* = 8.3 Hz, 1 H), 4.73–4.09 (m, 5 H), 4.00–3.73 (m, 1 H), 3.52 (br s, 1 H), 3.08 (s, 3 H), 2.61 (tt, *J* = 7.2, 4.0 Hz, 1 H), 2.41 (s, 3 H), 2.32–2.24 (br m, 2 H), 2.24–2.09 (br m, 4 H), 2.09–1.96 (br m, 4 H), 1.92–1.76 (br m, 2 H), 1.31 (s, 3 H), 1.06–1.96 (br m, 2 H), 0.87 (s, 9 H), 0.67 (br s, 2 H); **^{13}C NMR** (126 MHz, $CDCl_3$) δ (ppm) 173.6, 172.7, 171.6, 171.3, 170.2 (br), 164.8, 164.0, 155.2 (d, *J* = 254.3 Hz), 152.1, 151.8, 150.6 (br), 148.4 (br), 145.0, 138.5, 137.4 (br, 2 C), 134.2 (d, *J* = 3.6 Hz), 131.8 (br), 131.4 (br), 130.8 (br), 129.6 (2 C), 128.2, 128.1 (2 C), 126.4 (br), 126.1 (br), 125.9 (d, *J* = 21.4 Hz), 125.7 (br), 125.3, 103.6, 90.0, 88.4 (d, *J* = 6.9 Hz), 70.2, 58.9, 58.2, 57.1, 43.2, 37.0 (br), 35.6 (br), 35.4 (br), 35.0, 34.8, 26.6 (3 C), 25.4, 24.1, 21.9, 16.1, 13.8, 8.6 (2 C); **^{19}F NMR** (471 MHz, $CDCl_3$) δ (ppm) -121.9 (s, 1 F); **HRMS** (m/z): $[M+H]^+$ calcd. for $C_{53}H_{58}F^{127}IN_{10}O_9S$, 1157.3215; found, 1157.3223.

[*Note: Compound **12** was observed as a complex rotameric mixture by ^{1}H as confirmed by ROESY NMR. Characterisation was based on 2D NMR experiments, including COSY, HSQC, HMBC and ROESY NMR].*

9.5 Single-step conjugation (14a-14g)

(±)-*N*-(2-(5-((4-Aminophenyl)sulfonamido)-1*H*-pyrazol-1-yl)phenyl)-3-(2-(2-((2-(2,6-dioxopiperidin-3-yl)-1,3-dioxoisoindolin-4-yl)amino)ethoxy)ethoxy)propanamide (14a)

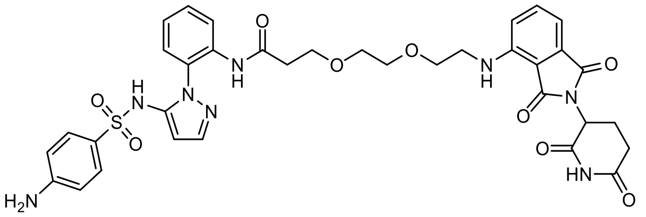

Prepared according to **General procedure A**, using 4-amino-*N*-(1-phenyl-1*H*-pyrazol-5-yl)benzenesulfonamide (*Sulfaphenazole*, 47.2 mg, 0.15 mmol) and (±)-2-(2,6-dioxopiperidin-3-yl)-4-((2-(2-(2-(5-oxo-1,4,2-dioxazol-3-yl)ethoxy)ethoxy)ethyl)amino)isoindoline-1,3-dione **13a** (71.2 mg, 0.15 mmol) as substrates. The crude reaction mixture was analysed by LCMS using acidic mobile phase and the UV chromatogram is shown below:

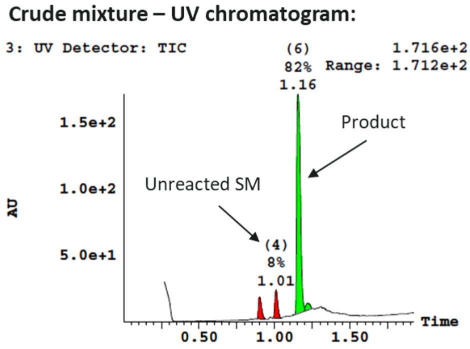

Supplementary Figure 54. LCMS UV chromatogram of the crude reaction mixture forming product **14a**.

Purification by preparative reverse phase HPLC (15-55% MeCN in HCO_2H buffer, 254 nm) afforded amidated derivative **14a** as a yellow solid (75.2 mg, 67%). **^{1}H NMR** (500 MHz, DMSO-d_6) δ (ppm) 11.10 (s, 1 H), 9.85 (br s, 1 H), 8.74 (br s, 1 H), 8.08 (d, *J* = 8.3 Hz, 1 H), 7.61 (d, *J* = 2.0 Hz, 1 H), 7.56 (t, *J* = 7.8 Hz, 1 H), 7.43 (t, *J* = 7.8 Hz, 1 H), 7.35 (d, *J* = 8.5 Hz, 2 H), 7.17 (t, *J* = 7.7 Hz, 1 H), 7.11 (d, *J* = 8.6 Hz, 1 H), 7.08–7.00 (m, 2 H), 6.64–6.53 (m, 3 H), 6.09 (br s, 2 H), 5.87 (d, *J* = 2.0 Hz, 1 H), 5.05 (dd, *J* = 12.7, 5.4 Hz, 1 H), 3.63–3.53 (m, 4 H), 3.49 (t, *J* = 4.6 Hz, 2 H), 3.47–3.92 (m, 4 H), 2.87 (ddd, *J* = 16.6, 13.7, 5.4 Hz, 1 H), 2.63–2.51 (m, part. overlap with solvent signal, 2 H), 2.42 (t, *J* = 6.0 Hz, 2 H), 2.06–1.96 (m, 1 H); **^{13}C NMR** (126 MHz, DMSO-d_6) δ (ppm) 172.9, 170.1, 169.2, 169.0, 167.3, 153.3, 146.4, 140.4, 137.0, 136.2, 134.2, 132.1, 129.04, 128.95 (2 C), 128.6, 127.8, 124.1, 124.0, 123.1, 117.5, 112.6 (2 C), 110.7, 109.2, 101.0, 69.6 (2 C), 68.9, 66.3, 48.6, 41.7, 37.3, 31.0, 22.2; **HRMS** (m/z): $[M+H]^+$ calcd. for $C_{35}H_{36}N_8O_9S$, 745.2404; found, 745.2421.

(±)-*N*-(2-(9-(2-(Benzyl(ethyl)amino)-2-oxoethyl)-7-methyl-8-oxo-8,9-dihydro-7*H*-purin-2-yl)phenyl)-3-(2-(2-((2-(2,6-dioxopiperidin-3-yl)-1,3-dioxoisoindolin-4-yl)amino)ethoxy)ethoxy)propanamide – TFA salt (14b)

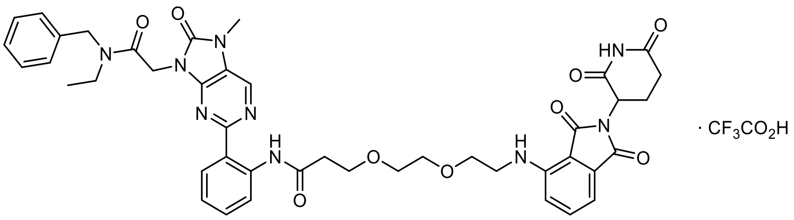

Prepared according to **General procedure B**, using *N*-benzyl-*N*-ethyl-2-(7-methyl-8-oxo-2-phenyl-7,8-dihydro-9*H*-purin-9-yl)acetamide (*Emapunil*, 60.2 mg, 0.15 mmol) and (±)-2-(2,6-dioxopiperidin-3-yl)-4-((2-(2-(2-(5-oxo-1,4,2-dioxazol-3-yl)ethoxy)ethoxy)ethyl)amino)isoindoline-1,3-dione **13a** 71.2 mg, 0.15 mmol) as substrates. The crude reaction mixture was analysed by LCMS using acidic mobile phase and the UV chromatogram is shown below:

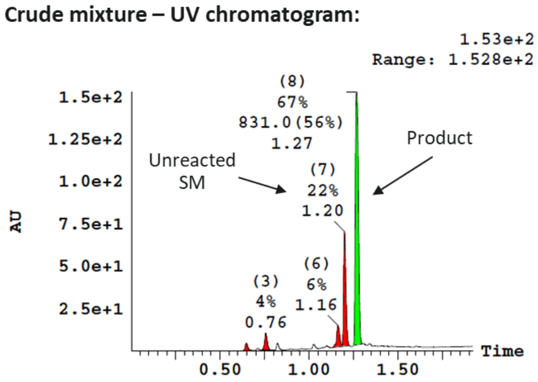

Supplementary Figure 55. LCMS UV chromatogram of the crude reaction mixture forming product **14b**.

Purification by preparative reverse phase HPLC (5-95% MeCN in 0.15 M CF_3CO_2H buffer, 254 nm, XSelect CSH Fluoro Phenyl OBD column - 5μm, 19x150mm) afforded amidated derivative **14b** as a yellow solid (TFA salt, 59.0 mg, 42%). **^{1}H NMR** (500 MHz, DMSO-*d*$_6$, observed as a 1:1 mixture of E:Z amide isomers) δ (ppm) 12.64 (s, 0.5 H), 12.58 (s, 0.5 H), 11.09 (s, 1 H), 8.64 (s, 0.5 H), 8.62 (s, 0.5 H), 8.60–8.52 (m, 1 H), 8.39 (d, *J* = 8.0 Hz, 0.5 H), 8.33 (d, *J* = 8.0 Hz, 0.5 H), 7.54–7.47 (m, 1 H), 7.47–7.34 (m, 3 H), 7.29–7.12 (m, 4 H), 7.05–6.94 (m, 2 H), 6.52 (br s, 1 H), 5.07–4.98 (m, 1 H), 4.95 (s, 1 H), 4.85 (s, 1 H), 4.76 (s, 1 H), 4.52 (s, 1 H), 3.81–3.71 (m, 2 H), 3.60–3.46 (m, 7 H), 3.45 (s, 1.5 H), 3.43 (s, 1.5 H), 3.36–3.25 (m, 3 H), 2.85 (ddd, *J* = 18.2, 13.8, 5.4 Hz, 1 H), 2.68–2.60 (m, 2 H), 2.60–2.51 (m, part. overlap with solvent signal, 2 H), 2.04–1.94 (m, 1 H), 1.22 (t, *J* = 7.0 Hz, 1.5 H), 1.00 (t, *J* = 7.1 Hz, 1.5 H); **^{13}C NMR** (126 MHz, DMSO-*d*$_6$, observed as a 1:1 mixture of E:Z amide isomers) δ (ppm) 172.8 (1 C), 170.1 (1 C), 169.2 (1 C), 168.9 (1 C), 167.3 (1 C), 165.4 and 165.2 (1 C), 158.4 (q, J_{CF} = 37.5 Hz, $CF_3\mathit{C}O_2H$), 156.42 and 156.35 (1 C), 153.0 (1 C), 149.51 and 149.49 (1 C), 146.3 (1 C), 138.55 and 138.48 (1 C), 137.7 and 137.2 (1 C), 136.2 (1 C), 132.0 (1 C), 131.0 and 130.9 (1 C), 130.7 and 130.6 (1 C), 129.8 and 129.6 (1 C), 128.8 and 128.4 (2 C), 127.5 and 127.1 (1 C), 127.4 and 126.5 (2 C), 123.1 and 122.9 (1 C), 122.73 and 127.66 (1 C), 121.6 (1 C), 120.31 and 120.27 (1 C), 117.3 (1 C), 115.3 (q, J_{CF} = 289.9 Hz, $\mathit{C}F_3CO_2H$), 110.6 (1 C), 109.2 (1 C), 69.9 (1 C), 69.7 (1 C), 68.8 (1 C), 66.6 (1 C), 48.6 (1 C), 49.1 and 48.0 (1 C), 41.6 (1 C), 41.2 (1 C), 41.5 and 40.9 (1 C), 38.70 and 38.65 (1 C), 31.0 (1 C), 27.49 and 27.45 (1 C), 22.1 (1 C), 13.8 and 12.6 (1 C); **^{19}F NMR** (471 MHz, DMSO-*d*$_6$) δ (ppm) -75.0 (s, $C\mathit{F}_3CO_2H$); **HRMS** (m/z): $[M+H]^+$ calcd. for $C_{43}H_{45}N_9O_9$, 832.3418; found, 832.3448.

(±)-6-((2-(2,6-Dioxopiperidin-3-yl)-1,3-dioxoisoindolin-5-yl)oxy)-*N*-(5-sulfamoyl-2-(5-(*p*-tolyl)-3-(trifluoromethyl)-1*H*-pyrazol-1-yl)phenyl)hexanamide (14c)

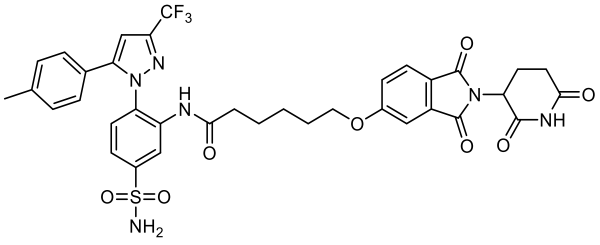

Prepared according to **General procedure A**, using 4-(5-(*p*-tolyl)-3-(trifluoromethyl)-1*H*-pyrazol-1-yl)benzenesulfonamide (57.2 mg, 0.15 mmol) and (±)-2-(2,6-dioxopiperidin-3-yl)-5-((5-(5-oxo-1,4,2-dioxazol-3-yl)pentyl)oxy)isoindoline-1,3-dione **13b** (64.4 mg, 0.15 mmol) as substrates. The crude

reaction mixture was analysed by LCMS using acidic mobile phase and the UV chromatogram is shown below:

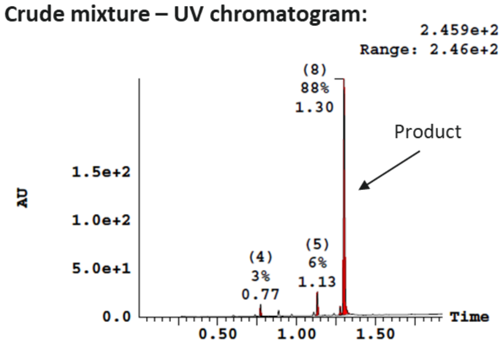

Supplementary Figure 56. LCMS UV chromatogram of the crude reaction mixture forming product **14c**.

Purification by preparative reverse phase HPLC (20-65% MeCN in HCO_2H buffer, 254 nm) afforded amidated derivative **14c** as a white solid (106 mg, 92%). **^{1}H NMR** (500 MHz, DMSO-d_6) δ (ppm) 11.12 (s, 1 H), 9.68 (s, 1 H), 8.24 (d, *J* = 2.1 Hz, 1 H), 7.82 (d, *J* = 8.3 Hz, 1 H), 7.63–7.52 (m, 3 H), 7.40 (d, *J* = 2.2 Hz, 1 H), 7.35–7.27 (m, 2 H), 7.24 (d, *J* = 8.0 Hz, 2 H), 7.21–7.11 (m, 3 H), 5.12 (dd, *J* = 12.8, 5.4 Hz, 1 H), 4.14 (t, *J* = 6.4 Hz, 2 H), 2.89 (ddd, *J* = 16.8, 13.8, 5.4 Hz, 1 H), 2.60 (ddd, *J* = 17.4, 4.7, 2.5 Hz, 1 H), 2.56–2.51 (m, part overlap with solvent signal, 1 H), 2.27 (s, 3 H), 2.18 (t, *J* = 7.3 Hz, 2 H), 2.10–2.00 (m, 1 H), 1.73 (p, *J* = 6.9 Hz, 2 H), 1.49 (p, *J* = 7.5 Hz, 2 H), 1.41–1.30 (m, 2 H); **^{13}C NMR** (126 MHz, DMSO-d_6) δ (ppm) 172.8, 171.1, 170.0, 166.92, 166.85, 164.1, 145.8, 144.7, 142.3 (q, J_{CF} = 37.4 Hz), 138.8, 134.3, 134.0, 133.6, 129.2 (2 C), 129.1, 128.2 (2 C), 125.5, 125.3, 122.9, 122.8, 122.0, 121.4 (q, J_{CF} = 268.8 Hz), 120.7, 108.8, 105.2, 68.6, 49.0, 35.5, 31.0, 28.2, 25.0, 24.6, 22.1, 20.8; **^{19}F NMR** (471 MHz, $CDCl_3$) δ (ppm) -60.8 (s, 3 F); **HRMS** (m/z): $[M+H]^+$ calcd. for $C_{36}H_{33}F_3N_6O_8S$, 767.2111; found, 767.2123.

(±)-3-(4-(2-(8-(4-(2-(2,6-Dioxopiperidin-3-yl)-1,3-dioxoisoindolin-5-yl)piperazin-1-yl)-8-oxooctanamido)phenyl)-5-phenyloxazol-2-yl)propanoic acid (14d)

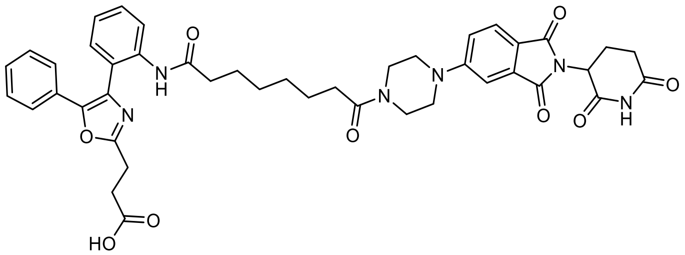

Prepared according to **General procedure B**, using 3-(4,5-diphenyloxazol-2-yl)propanoic acid (*Oxaprozin*, 44.0 mg, 0.15 mmol) and (±)-2-(2,6-dioxopiperidin-3-yl)-5-(4-(7-(5-oxo-1,4,2-dioxazol-3-yl)heptanoyl)piperazin-1-yl)isoindoline-1,3-dione **13c** (80.9 mg, 0.15 mmol) as substrates. The crude reaction mixture was analysed by LCMS using acidic mobile phase and the UV chromatogram is shown below:

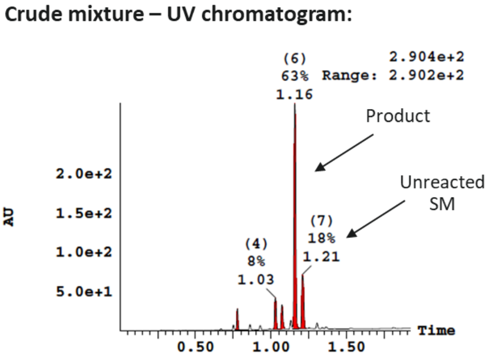

Supplementary Figure 57. LCMS UV chromatogram of the crude reaction mixture forming product **14d**.

Purification by preparative reverse phase HPLC (15-55% MeCN in HCO_2H buffer, 254 nm) afforded amidated derivative **14d** as a yellow solid (72.6 mg, 61%). **^{1}H NMR** (500 MHz, DMSO-*d*$_6$) δ (ppm) 12.41 (br s, 1 H), 11.10 (s, 1 H), 9.15 (s, 1 H), 7.97 (d, *J* = 8.2 Hz, 1 H), 7.70 (d, *J* = 8.5 Hz, 1 H), 7.41–7.26 (m, 8 H), 7.23 (dd, *J* = 8.6, 2.3 Hz, 1 H), 7.14 (t, *J* = 7.5 Hz, 1 H), 5.08 (dd, *J* = 12.8, 5.4 Hz, 1 H), 3.65–3.54 (br m, 4 H), 3.53–3.47 (br m, 2 H), 3.47–3.41 (br m, 2 H), 3.10 (t, *J* = 7.1 Hz, 2 H), 2.89 (ddd, *J* = 16.7, 13.7, 5.3 Hz, 1 H), 2.80 (t, *J* = 7.1 Hz, 2 H), 2.63–2.51 (m, part. overlap with solvent signal, 2 H), 2.31 (t, *J* = 7.4 Hz, 2 H), 2.06–1.97 (m, 1 H), 1.92 (t, *J* = 7.4 Hz, 2 H), 1.45 (p, *J* = 7.5 Hz, 2 H), 1.31 (p, *J* = 7.5 Hz, 2 H), 1.21 (p, *J* = 7.0 Hz, 2 H), 1.17–1.09 (m, 2 H); **^{13}C NMR** (126 MHz, DMSO-*d*$_6$) δ (ppm) 173.2, 172.8, 171.0, 170.9, 170.1, 167.6, 167.0, 162.3, 154.9, 145.4, 136.3, 133.9, 131.7, 130.1, 128.8, 128.7 (2 C), 128.4, 128.1, 125.2 (2 C), 125.0, 123.9, 123.5, 123.4, 118.5, 117.8, 107.9, 48.8, 46.8, 46.6, 44.1, 40.4, 35.9, 32.2, 31.0, 30.3, 28.5, 28.4, 24.7, 24.5, 23.0, 22.2; **HRMS** (m/z): $[M+H]^+$ calcd. for $C_{43}H_{44}N_6O_9$, 789.3248; found, 789.3287.

Methyl ((5*S*,10*S*,11*S*,14*S*)-11-benzyl-5-(*tert*-butyl)-8-(3-(2-(3-(4-(2-((*RS*)-2,6-dioxopiperidin-3-yl)-1-oxoisoindolin-5-yl)piperazine-1-carbonyl)bicyclo[1.1.1]pentan-1-yl)acetamido)-4-(pyridin-2-yl)benzyl)-10-hydroxy-15,15-dimethyl-3,6,13-trioxo-2-oxa-4,7,8,12-tetraazahexadecan-14-yl)carbamate – TFA salt (14e)

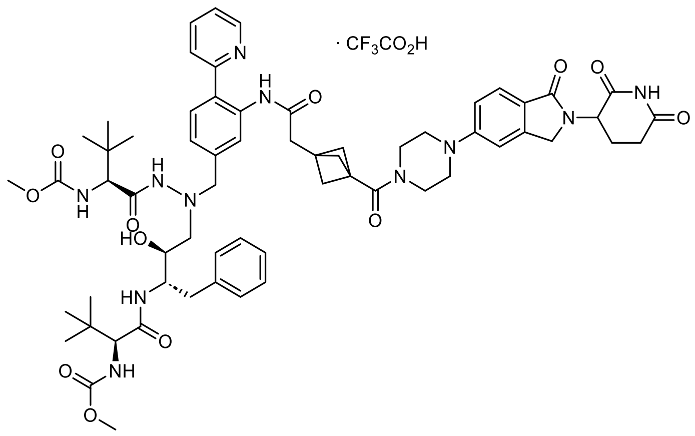

Prepared according to **General procedure B**, using methyl ((5*S*,10*S*,11*S*,14*S*)-11-benzyl-5-(*tert*-butyl)-10-hydroxy-15,15-dimethyl-3,6,13-trioxo-8-(4-(pyridin-2-yl)benzyl)-2-oxa-4,7,8,12-tetraazahexadecan-14-yl)carbamate (*Atazanavir*, 65.0 mg, 0.09 mmol) and (±)-3-(1-oxo-5-(4-(3-((5-oxo-1,4,2-dioxazol-3-yl)methyl)bicyclo[1.1.1]pentane-1-carbonyl)piperazin-1-yl)isoindolin-2-yl)piperidine-2,6-dione **13d** (53.1 mg, 0.09 mmol) as substrates. The crude reaction mixture was analysed by LCMS using acidic mobile phase and the UV chromatogram is shown below:

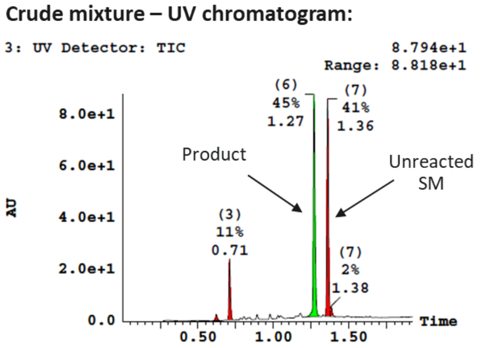

Supplementary Figure 58. LCMS UV chromatogram of the crude reaction mixture forming product **14e**.

Purification by preparative reverse phase HPLC (5-95% MeCN in 0.15 M CF_3CO_2H buffer, 254 nm, XSelect CSH Fluoro Phenyl OBD column - 5µm, 19x150mm) afforded amidated derivative **14e** as a white solid (TFA salt, 40.9 mg, 34%). **^{1}H NMR** (500 MHz, DMSO-d_6) δ (ppm) 11.29 (br s, 1 H), 10.95 (s, 1 H), 9.17 (s, 1 H), 8.79 (dd, J = 5.3, 1.8 Hz, 1 H), 8.17 (t, J = 7.8 Hz, 1 H), 8.01 (s, 1 H), 7.88 (d, J = 8.1 Hz, 1 H), 7.68–7.58 (m, 2 H), 7.58–7.49 (m, 2 H), 7.30 (d, J = 7.5 H-z, 1 H), 7.23–15 (m, 4 H), 7.15–7.09 (m, 1 H), 7.08–7.03 (m, 2 H), 6.99 (d, J = 9.3 Hz, 1 H), 6.88 (d, J = 9.4 Hz, 1 H), 5.04 (dd, J = 13.3, 5.1 Hz, part. overlap with H_2O broad signal, 1 H), 4.31 (d, J = 16.9 Hz, 1 H), 4.19 (d, J = 17.2 Hz, 1 H), 4.09–3.90 (m, 3 H), 3.83 (d, J = 9.4 Hz, 1 H), 3.71–3.55 (m, 6 H), 3.53 (s, 3 H), 3.49 (s, 3 H), 3.45–3.20 (m, 4 H), 2.89 (ddd, J = 17.2, 13.6, 5.4 Hz, 1 H), 2.84–2.89 (m, 3 H), 2.68–2.61 (m, 1 H), 2.61–2.54 (m, 1 H), 2.50–2.44 (m, overlap with solvent signal, 2 H), 2.40–2.26 (m, 1 H), 2.03–1.89 (s, 7 H), 0.75 (s, 9 H), 0.61 (s, 9 H); **^{13}C NMR** (126 MHz, DMSO-d_6) δ (ppm) 173.0, 171.3, 170.3, 170.1, 168.30, 168.26, 166.9, 158.4 (q, J_{CF} = 37.3 Hz, CF_3CO_2H), 156.6, 156.5, 155.5 (br), 153.5, 146.1 (br), 144.1, 140.7 (br), 140.2 (br), 139.0, 136.4, 129.4, 129.1 (2 C), 128.0 (2 C), 125.9, 125.2, 124.4 (br, 2 C), 123.9, 123.2 (br), 122.9 (br), 121.9, 115.5 (q, J_{CF} = 289.4 Hz, CF_3CO_2H), 115.0, 108.7, 68.1, 63.2, 61.3, 61.0, 60.9, 53.1 (3 C), 51.6, 51.5–51.4 (m, 4 C), 47.9, 47.5, 47.0, 44.5, 41.1, 39.9, 37.7, 37.5, 33.6, 33.4, 31.3, 26.7 (3 C), 26.3 (3 C), 22.6; **^{19}F NMR** (471 MHz, DMSO-d_6) δ (ppm) -74.9 (s, CF_3CO_2H); **HRMS** (m/z): $[M+H]^+$ calcd. for $C_{63}H_{79}N_{11}O_{12}$, 1182.5983; found, 1182.6001.

(±)-*N*-(4-(7-Chloro-5-hydroxy-2,3,4,5-tetrahydro-1*H*-benzo[*b*]azepine-1-carbonyl)-2-(3-(1-(2-(2,6-dioxopiperidin-3-yl)-1,3-dioxoisoindolin-4-yl)piperidin-4-yl)propanamido)-5-methylphenyl)-2-methylbenzamide (14f)

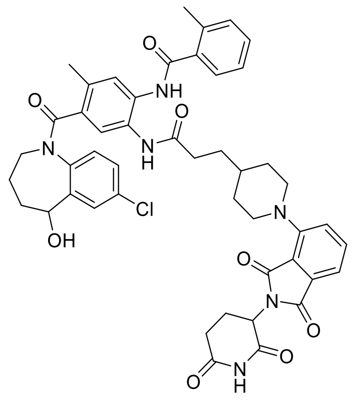

Prepared according to **General procedure B**, using (±)-*N*-(4-(7-chloro-5-hydroxy-2,3,4,5-tetrahydro-1*H*-benzo[*b*]azepine-1-carbonyl)-3-methylphenyl)-2-methylbenzamide (*Tolvaptan,* 41.0 mg, 0.09 mmol) and (±)-2-(2,6-dioxopiperidin-3-yl)-4-(4-(2-(5-oxo-1,4,2-dioxazol-3-yl)ethyl)piperidin-1-yl)isoindoline-1,3-dione **13e** (41.5 mg, 0.09 mmol) as substrates, and PivOH (2.80 mg, 0.03 mmol) in place of $(PhO)_2PO_2H$. The crude reaction mixture was analysed by LCMS using acidic mobile phase and the UV chromatogram is shown below:

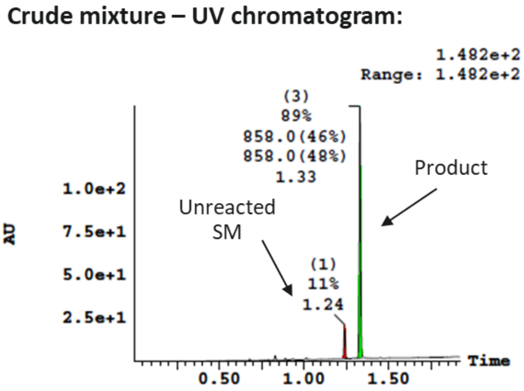

Supplementary Figure 59. LCMS UV chromatogram of the crude reaction mixture forming product **14f**.

Purification by preparative reverse phase HPLC (30-90% MeCN in HCO_2H buffer, 254 nm) afforded amidated derivative **14f** as a yellow solid (69.5 mg, 89%). **^{1}H NMR** (600 MHz, DMSO-*d*$_6$) δ (ppm) 11.07 (s, 1 H), 9.76–9.18 (m, 2 H), 7.79–7.44 (m, 4 H), 6.44–7.67 (m, 8 H), 5.78–5.19 (m, 1 H), 5.09 (dd, *J* = 12.8, 5.5 Hz, 1 H), 4.93–4.57 (m, 2 H), 3.64 (br s, 2 H), 2.87 (ddd, *J* = 17.6, 14.1, 5.5 Hz, 1 H), 2.82–2.64 (m, 3 H), 2.63–2.56 (br m, 1 H), 2.56–2.52 (m, 1 H), 2.47–2.34 (m, 5 H), 2.31 (br s, 3 H), 2.18–2.09 (br m, 1 H), 2.02 (dt, *J* = 11.8, 5.7 Hz, 1H), 1.99–1.88 (m, 1 H), 1.83–1.77 (m, 3 H), 1.67–1.45 (m, 3 H), 1.45–1.22 (m, 3 H); **^{13}C NMR** (151 MHz, DMSO-*d*$_6$, only major peaks are reported) δ (ppm) 172.8, 171.9, 171.6, 170.0, 169.3, 167.9, 167.7, 167.1, 166.3, 150.1, 144.8, 138.4, 136.2, 135.7, 133.7, 133.2, 131.6, 130.8, 130.0, 129.5, 127.3, 126.7, 126.4, 125.7, 125.2, 123.9, 122.5, 116.3, 114.4, 69.6, 51.0, 48.8, 46.0, 35.6, 34.3, 34.1, 33.3, 31.7, 31.0, 25.8, 22.1, 19.5, 18.9; **HRMS** (m/z): $[M+H]^+$ calcd. for $C_{47}H_{47}{}^{35}ClN_6O_8$, 859.3222; found, 859.3235.

[*Note: Compound **14f** was observed as a complex mixture of interconverting isomers by ^{1}H and ^{13}C NMR, as confirmed by ROESY NMR. Characterisation was based on 2D NMR experiments, including COSY, HSQC, HMBC and ROESY NMR, as well as on analogy with analogue derivative **8r** (see Supplementary Section 6)*.]

3-(4-(2-(5-((3a*S*,4*S*,6a*R*)-2-Oxohexahydro-1*H*-thieno[3,4-*d*]imidazol-4-yl)pentanamido)phenyl)-5-phenyloxazol-2-yl)propanoic acid (14g)

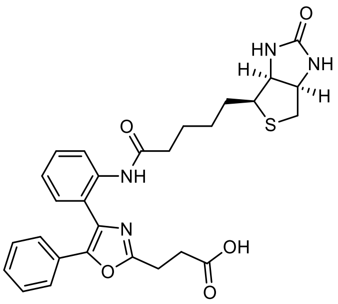

Prepared according to **General procedure B**, using 3-(4,5-diphenyloxazol-2-yl)propanoic acid (*Oxaprozin*, 58.7 mg, 0.20 mmol) and 3-(4-((3a*S*,4*S*,6a*R*)-2-oxohexahydro-1*H*-thieno[3,4-*d*]imidazol-4-yl)butyl)-1,4,2-dioxazol-5-one **17** (62.8 mg, 0.22 mmol) as substrates. The crude reaction mixture was analysed by LCMS using acidic mobile phase and the UV chromatogram is shown below:

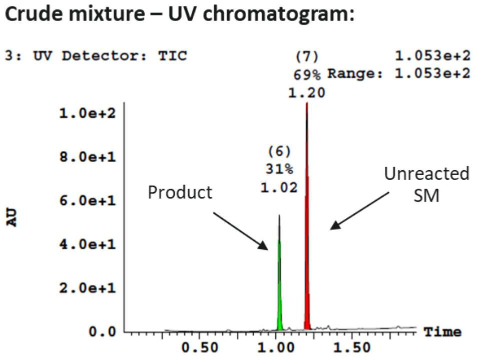

Supplementary Figure 60. LCMS UV chromatogram of the crude reaction mixture forming product **14g**.

Purification by preparative reverse phase HPLC (15-60% MeCN in HCO_2H buffer, 254 nm) afforded amidated derivative **14g** as an off-white solid (33.8 mg, 32%). **^{1}H NMR** (500 MHz, DMSO-d_6) δ (ppm) 9.14 (s, 1 H), 7.95 (d, *J* = 8.2 Hz, 1 H), 7.44–7.25 (m, 7 H), 7.15 (t, *J* = 7.5 Hz, 1 H), 6.42 (br s, 2 H), 4.29 (dd, *J* = 7.7, 4.9 Hz, 1 H), 4.09 (dd, *J* = 7.7, 4.4 Hz, 2 H), 3.10 (t, *J* = 7.1 Hz, 2 H),3.07–2.99 (m, 1 H), 2.84–2.76 (m, 3 H), 2.56 (d, *J* = 12.4 Hz, 1 H), 1.91 (t, *J* = 7.4 Hz, 2 H), 1.59–1.47 (m, 1 H), 1.43–1.25 (m, 3 H), 1.25–1.11 (m, 2 H); **^{13}C NMR** (126 MHz, DMSO-d_6) δ (ppm) 173.2, 170.9, 162.8, 162.3, 145.4, 136.3, 131.7, 130.1, 128.8, 128.7 (2 C), 128.4, 128.2, 125.2 (2 C), 124.0, 123.6, 123.5, 61.1, 59.2, 55.4, 39.4, 35.7, 30.3, 28.2, 28.0, 24.8, 23.0; **HRMS** (m/z): $[M+H]^+$ calcd. for $C_{28}H_{30}N_4O_5S$, 535.2015; found, 535.2028.

10. ^{1}H and ^{13}C NMR spectra for novel compounds

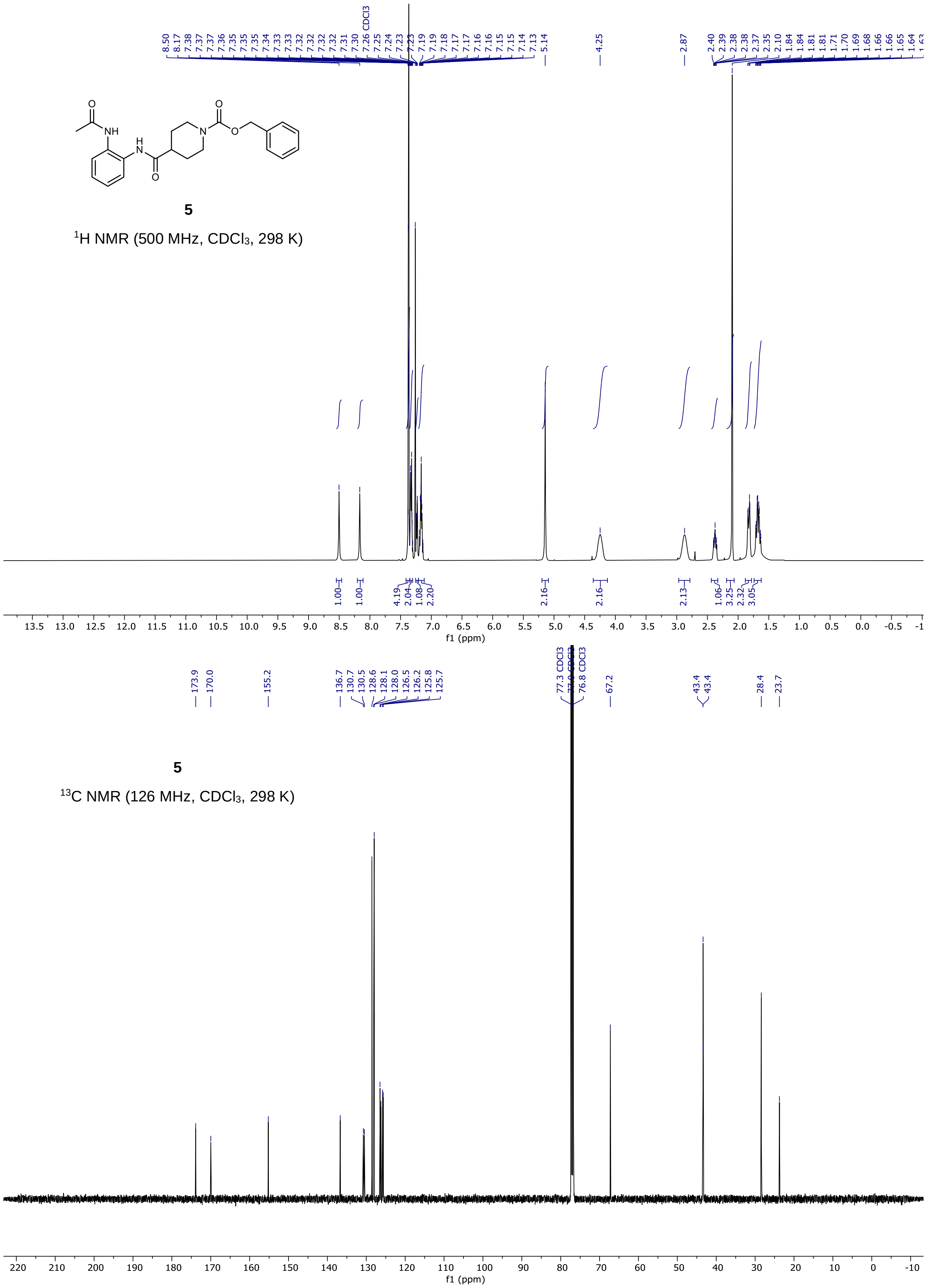

Supplementary Figure 61. ^{1}H NMR (top) and ^{13}C NMR (bottom) of compound **5**. Frequency, temperature and solvent of measurement are indicated on each spectra.

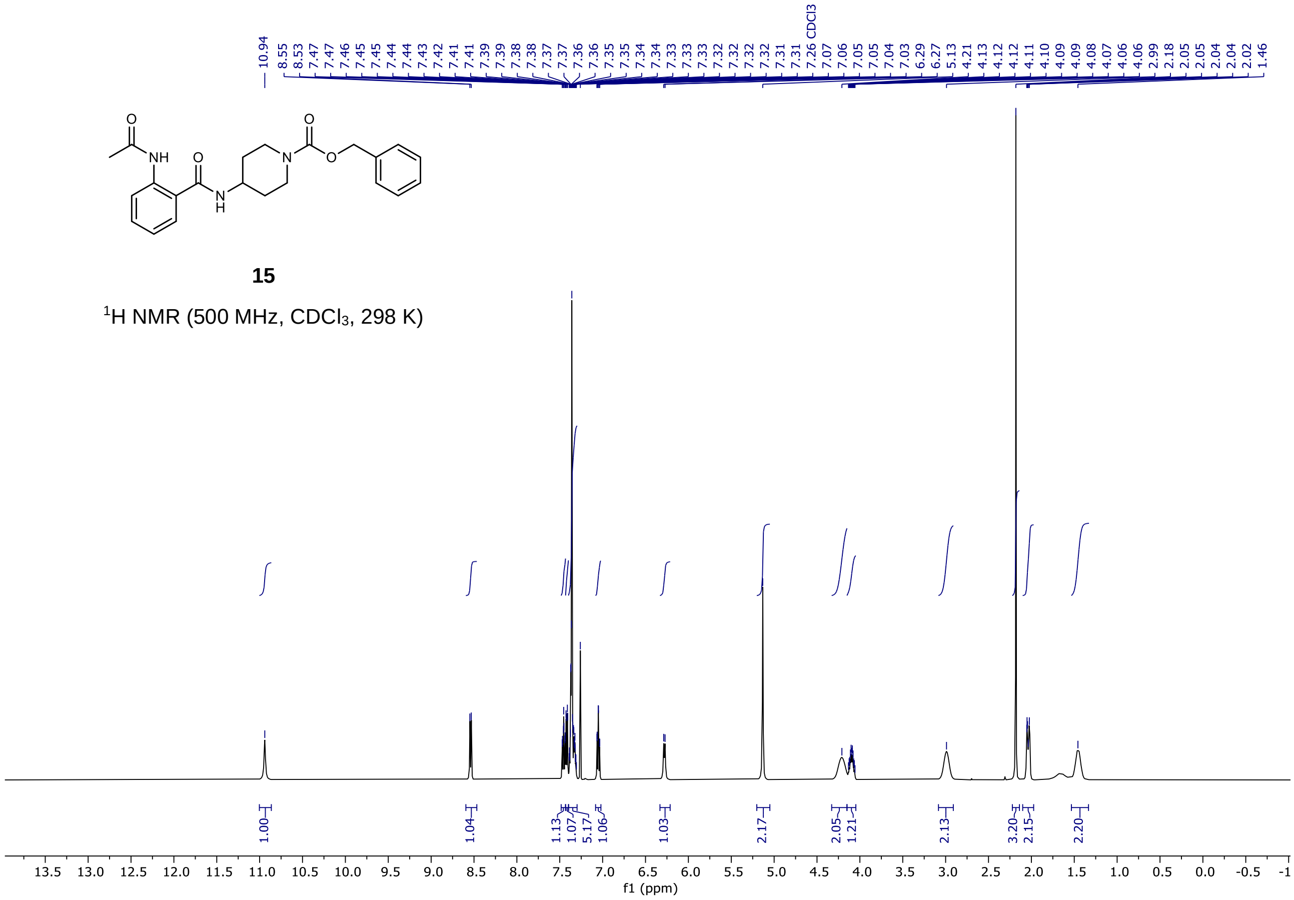

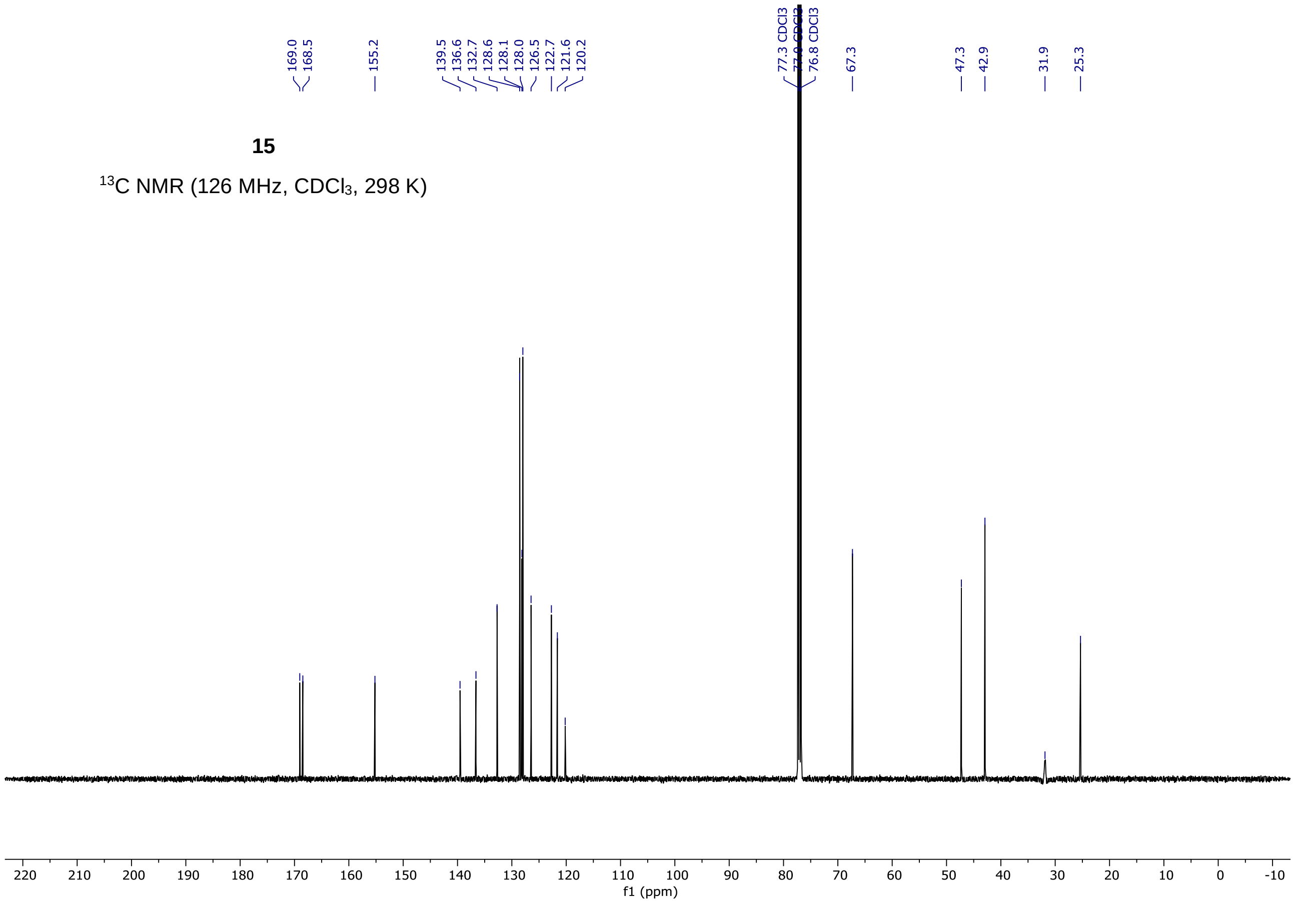

Supplementary Figure 62. ^{1}H NMR (top) and ^{13}C NMR (bottom) spectra of compound **15**. Frequency, temperature and solvent of measurement are indicated on each spectra.

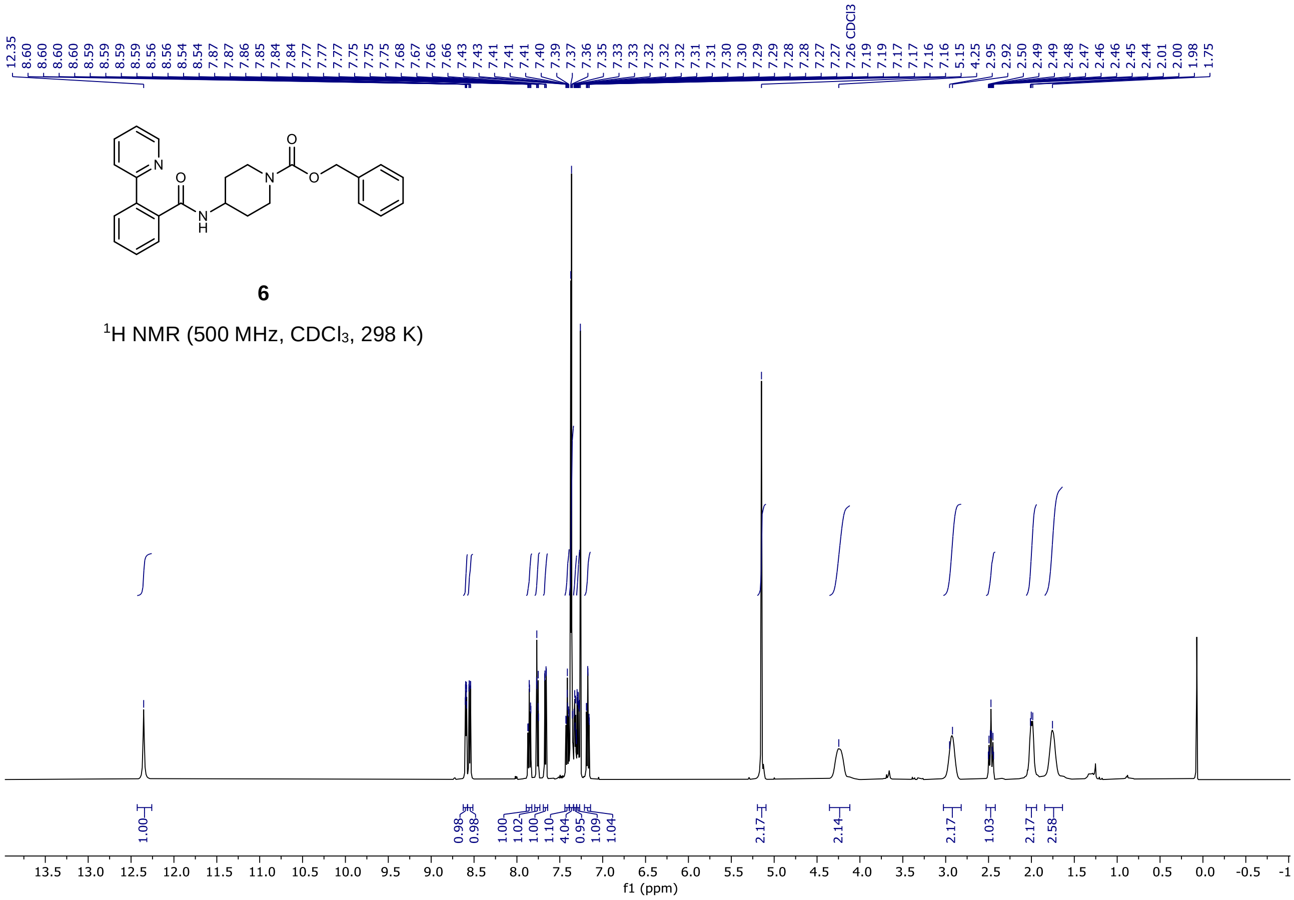

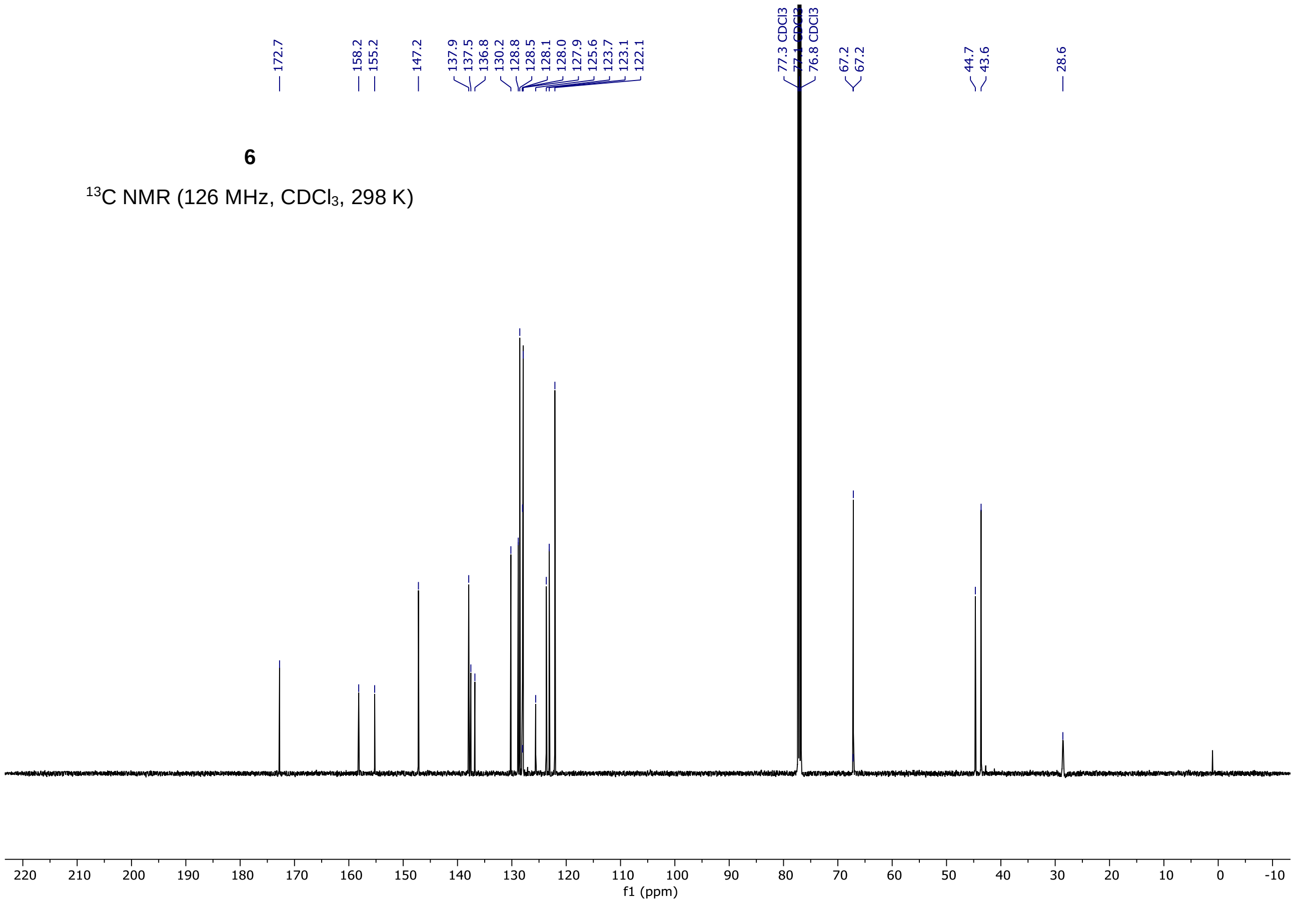

Supplementary Figure 63. ^{1}H NMR (top) and ^{13}C NMR (bottom) spectra of compound **6**. Frequency, temperature and solvent of measurement are indicated on each spectra.

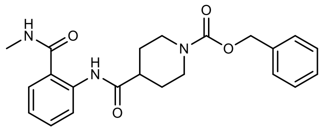

16

^{1}H NMR (500 MHz, $CDCl_3$, 298 K)

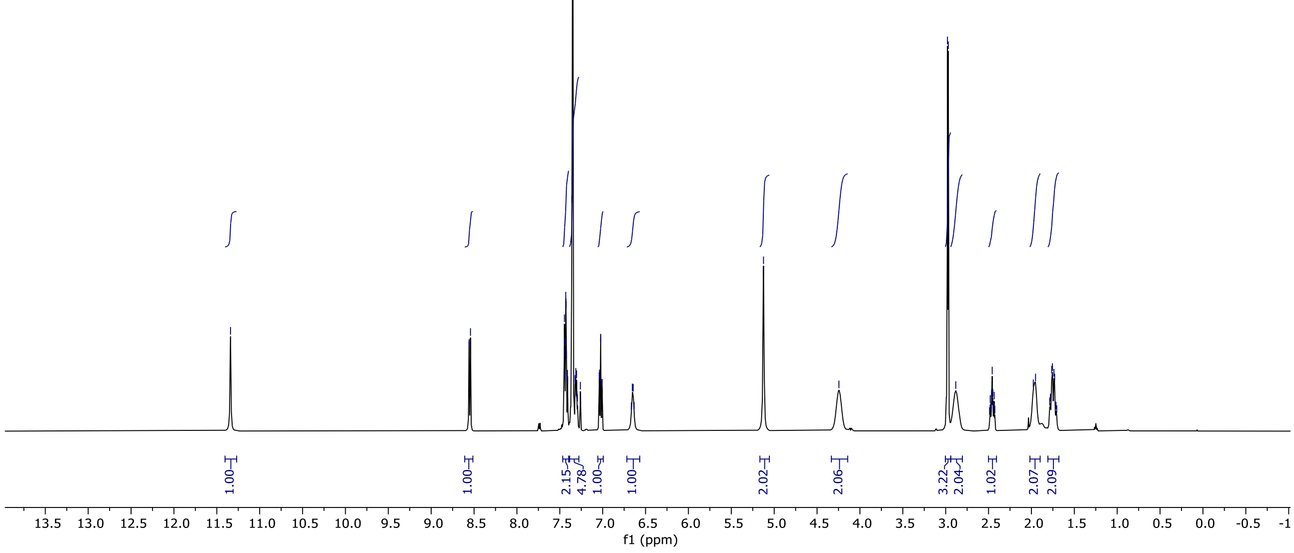

16

^{13}C NMR (126 MHz, $CDCl_3$, 298 K)

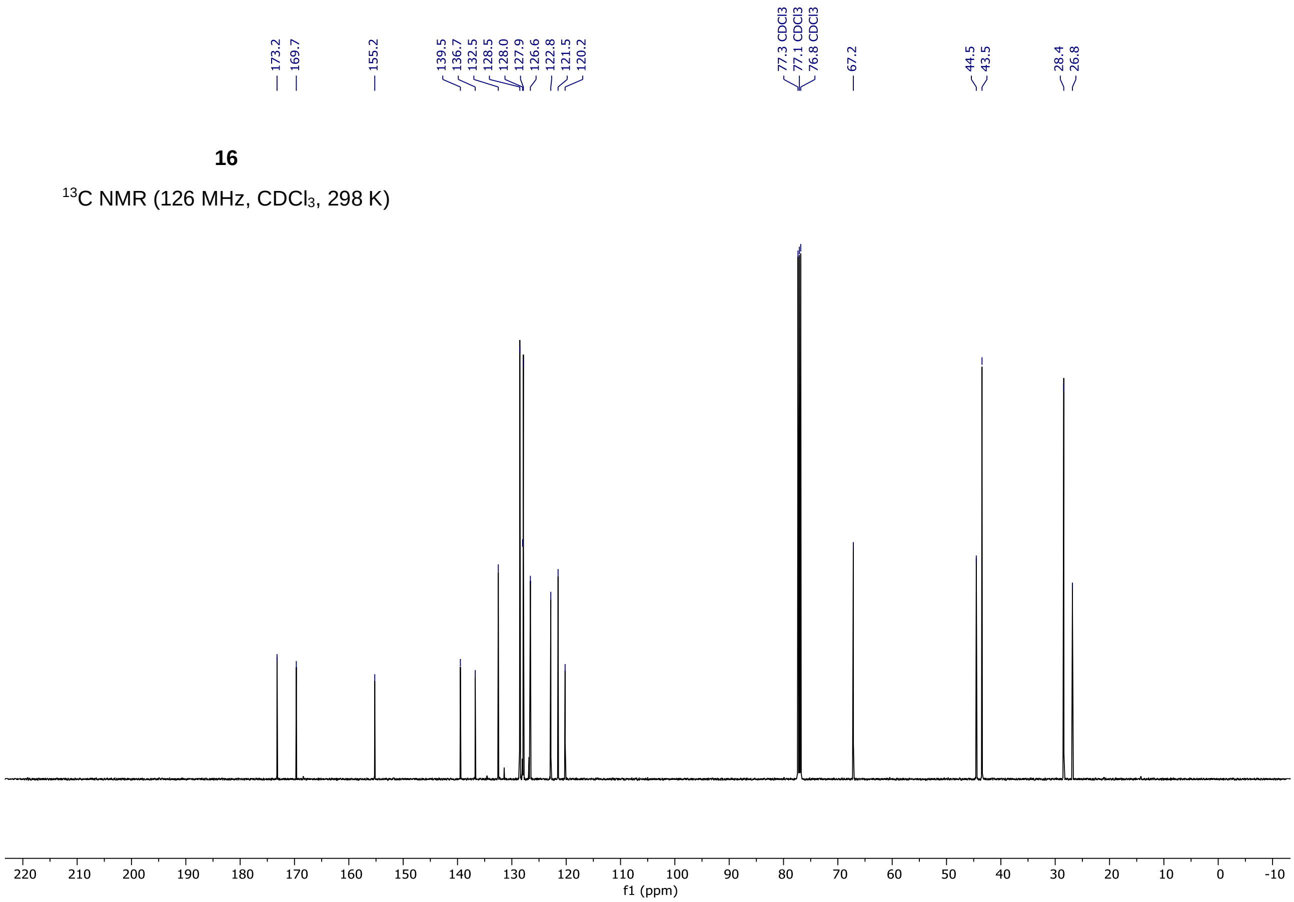

Supplementary Figure 64. ^{1}H NMR (top) and ^{13}C NMR (bottom) spectra of compound **16**. Frequency, temperature and solvent of measurement are indicated on each spectra.

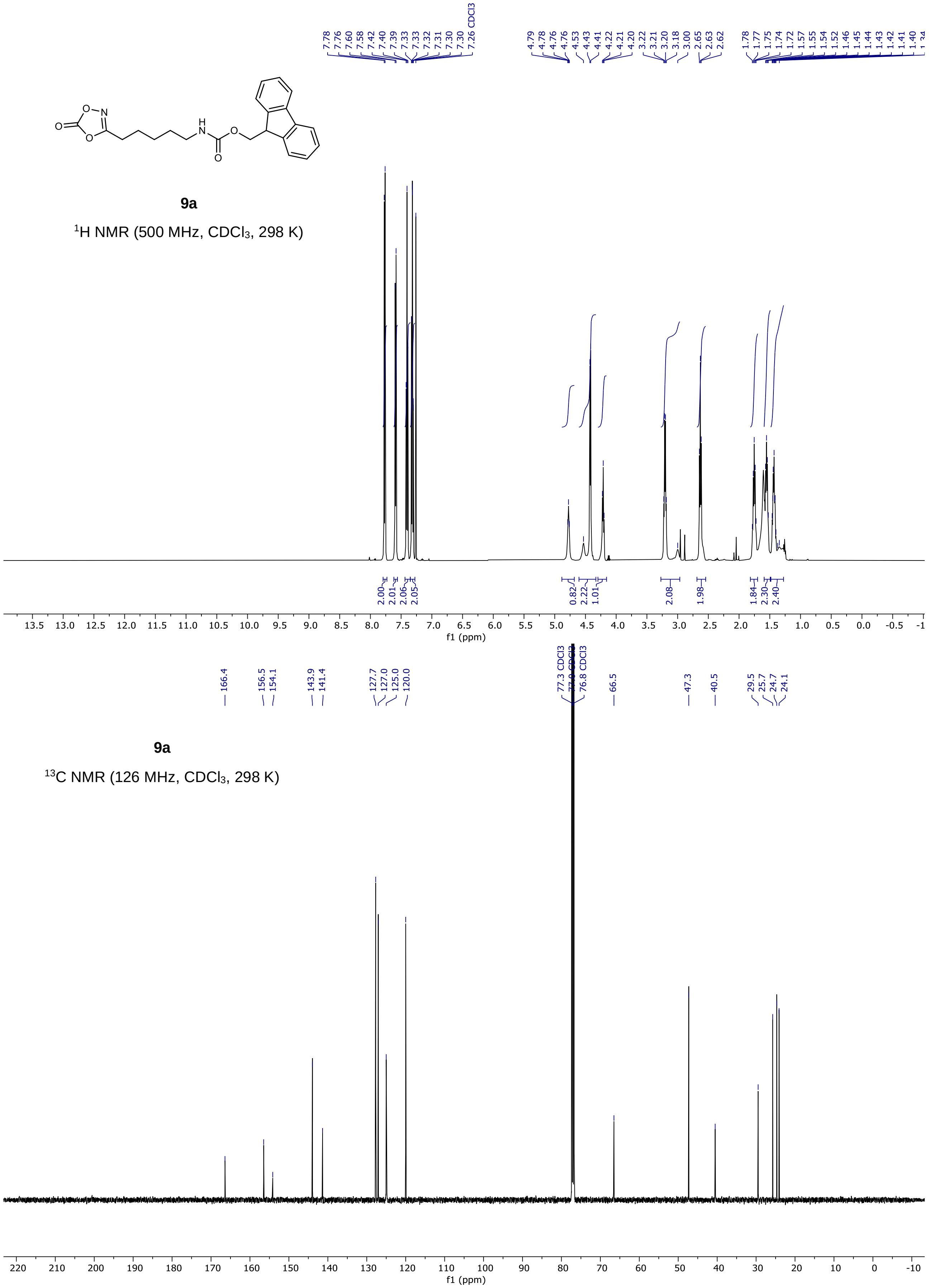

Supplementary Figure 65. ^{1}H NMR (top) and ^{13}C NMR (bottom) spectra of compound **9a**. Frequency, temperature and solvent of measurement are indicated on each spectra.

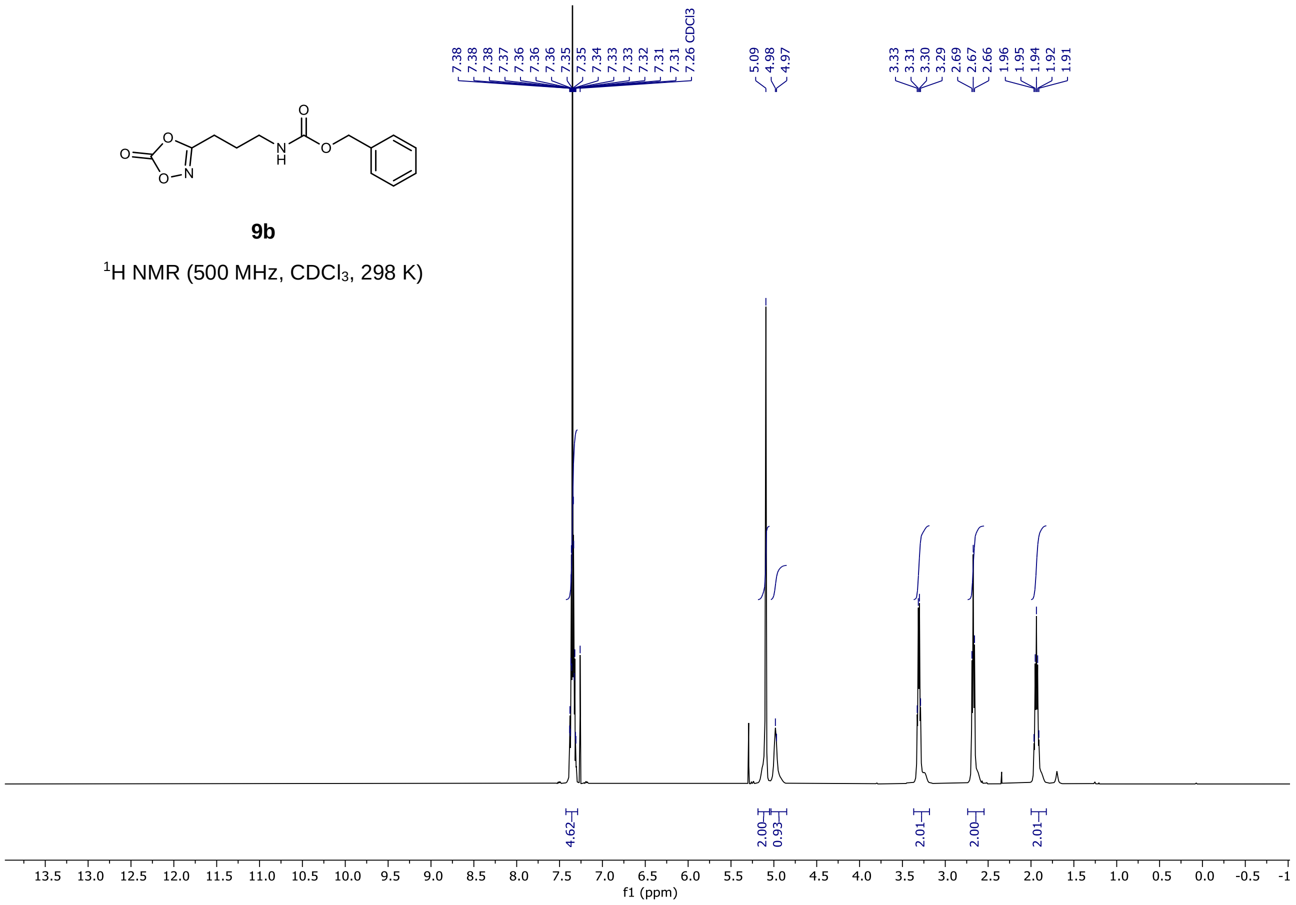

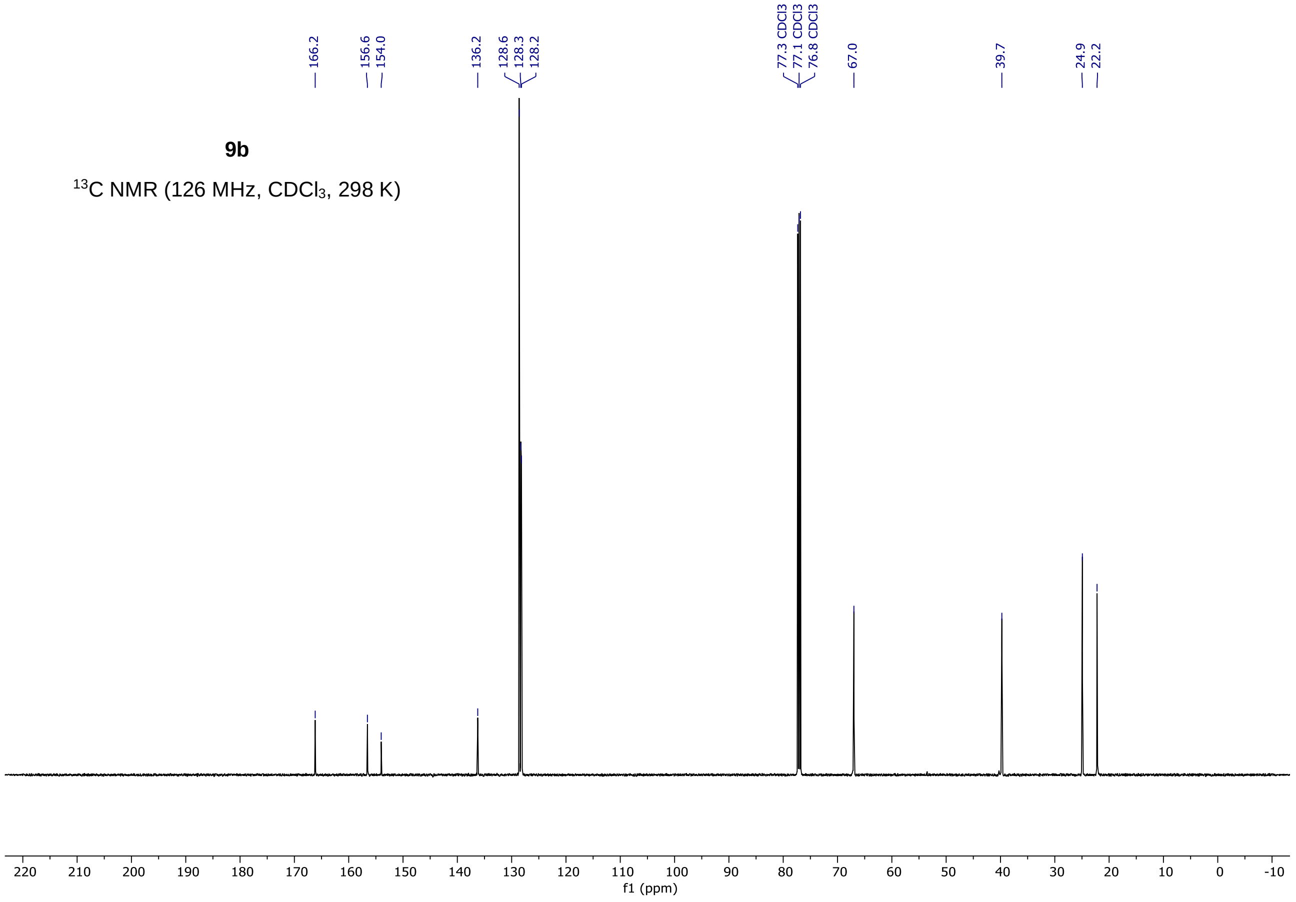

Supplementary Figure 66. ^{1}H NMR (top) and ^{13}C NMR (bottom) spectra of compound **9b**. Frequency, temperature and solvent of measurement are indicated on each spectra.

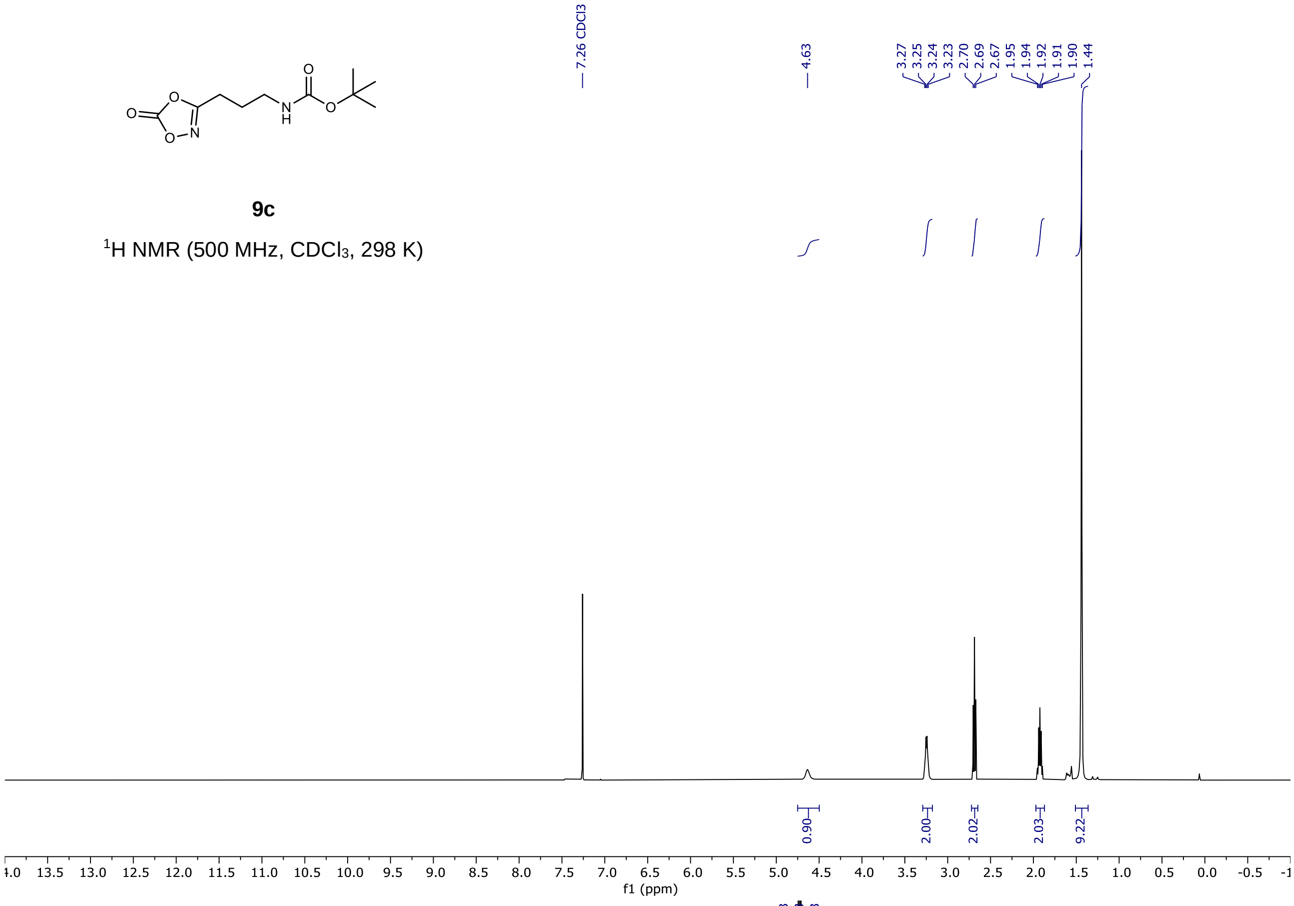

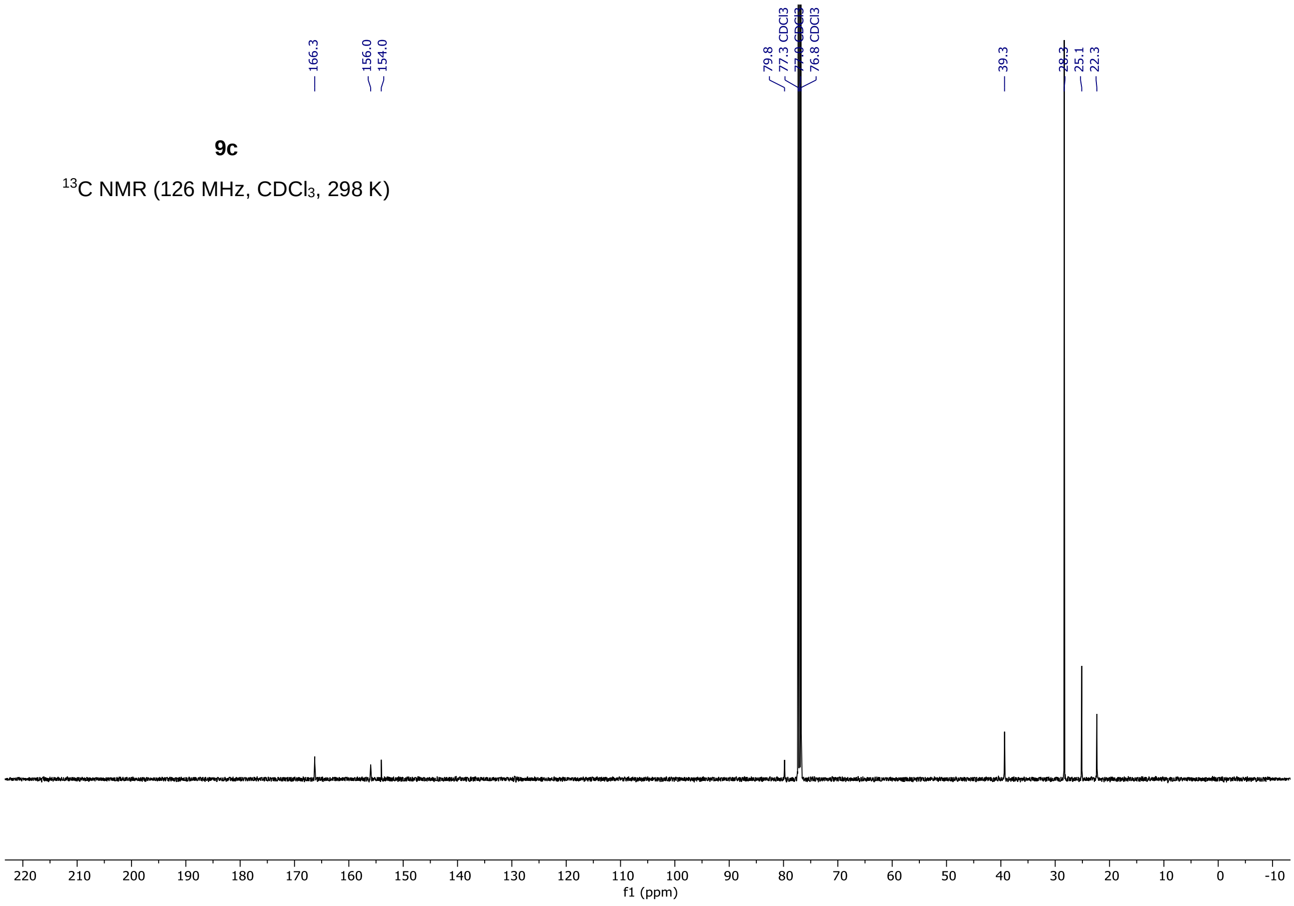

Supplementary Figure 67. ^{1}H NMR (top) and ^{13}C NMR (bottom) spectra of compound **9c**. Frequency, temperature and solvent of measurement are indicated on each spectra.

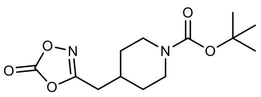

9e

^1H NMR (500 MHz, $CDCl_3$, 298 K)

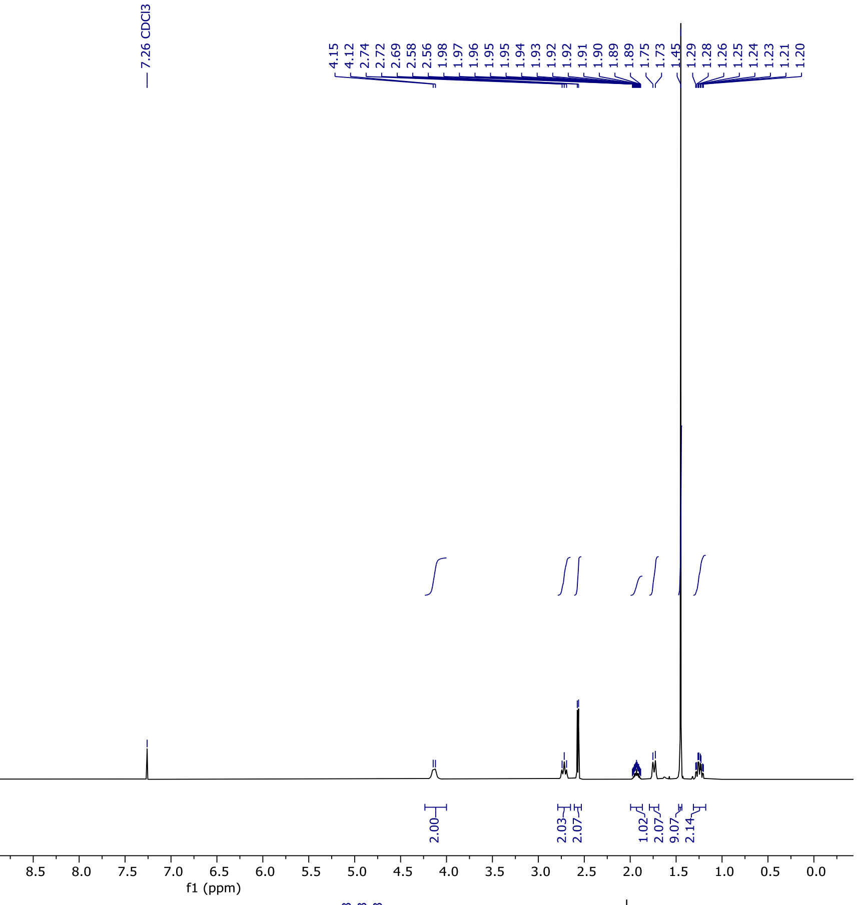

9e

^{13}C NMR (126 MHz, $CDCl_3$, 298 K)

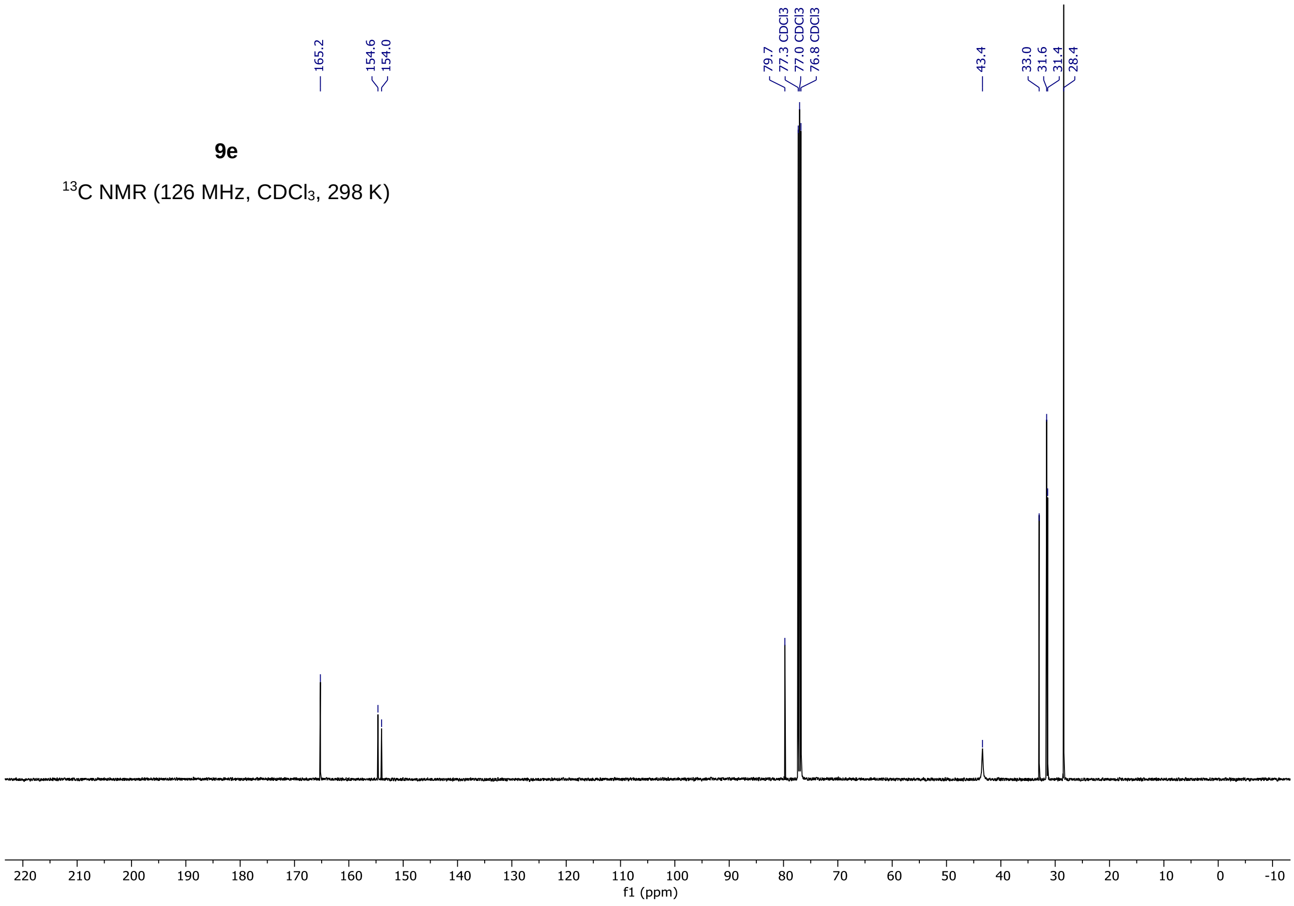

Supplementary Figure 68. ^1H NMR (top) and ^{13}C NMR (bottom) spectra of compound **9e**. Frequency, temperature and solvent of measurement are indicated on each spectra.

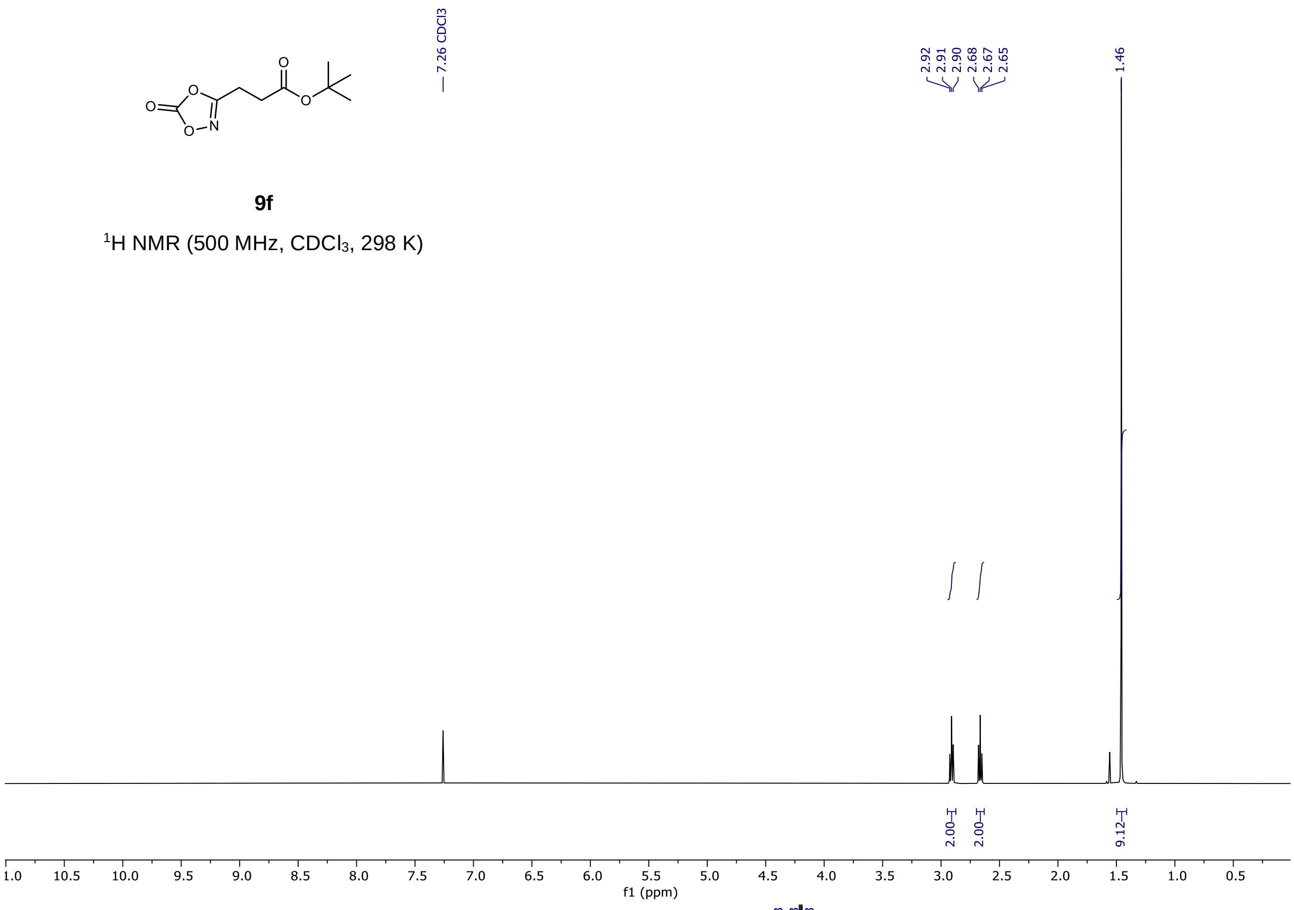

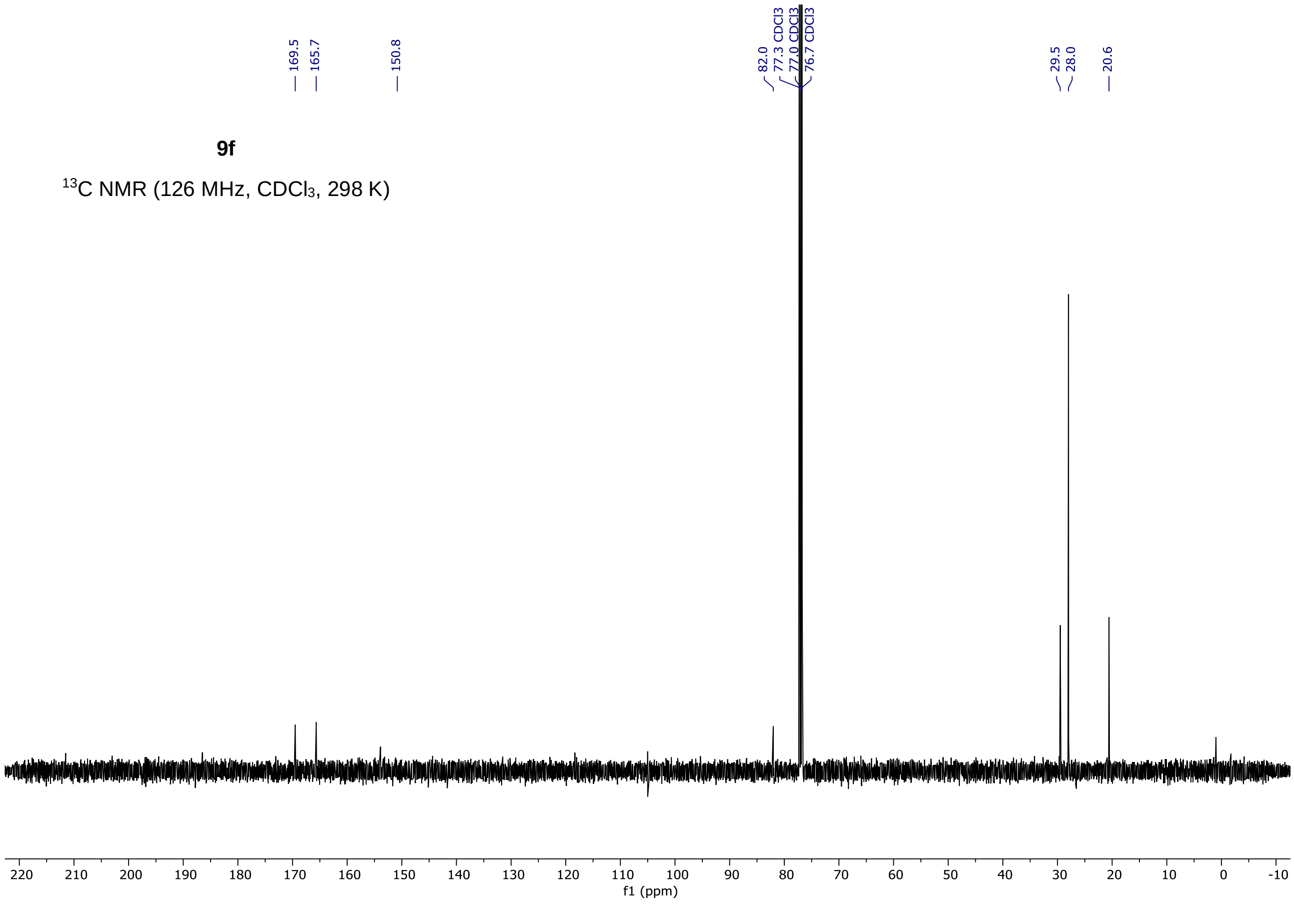

Supplementary Figure 69. ^{1}H NMR (top) and ^{13}C NMR (bottom) spectra of compound **9f**. Frequency, temperature and solvent of measurement are indicated on each spectra.

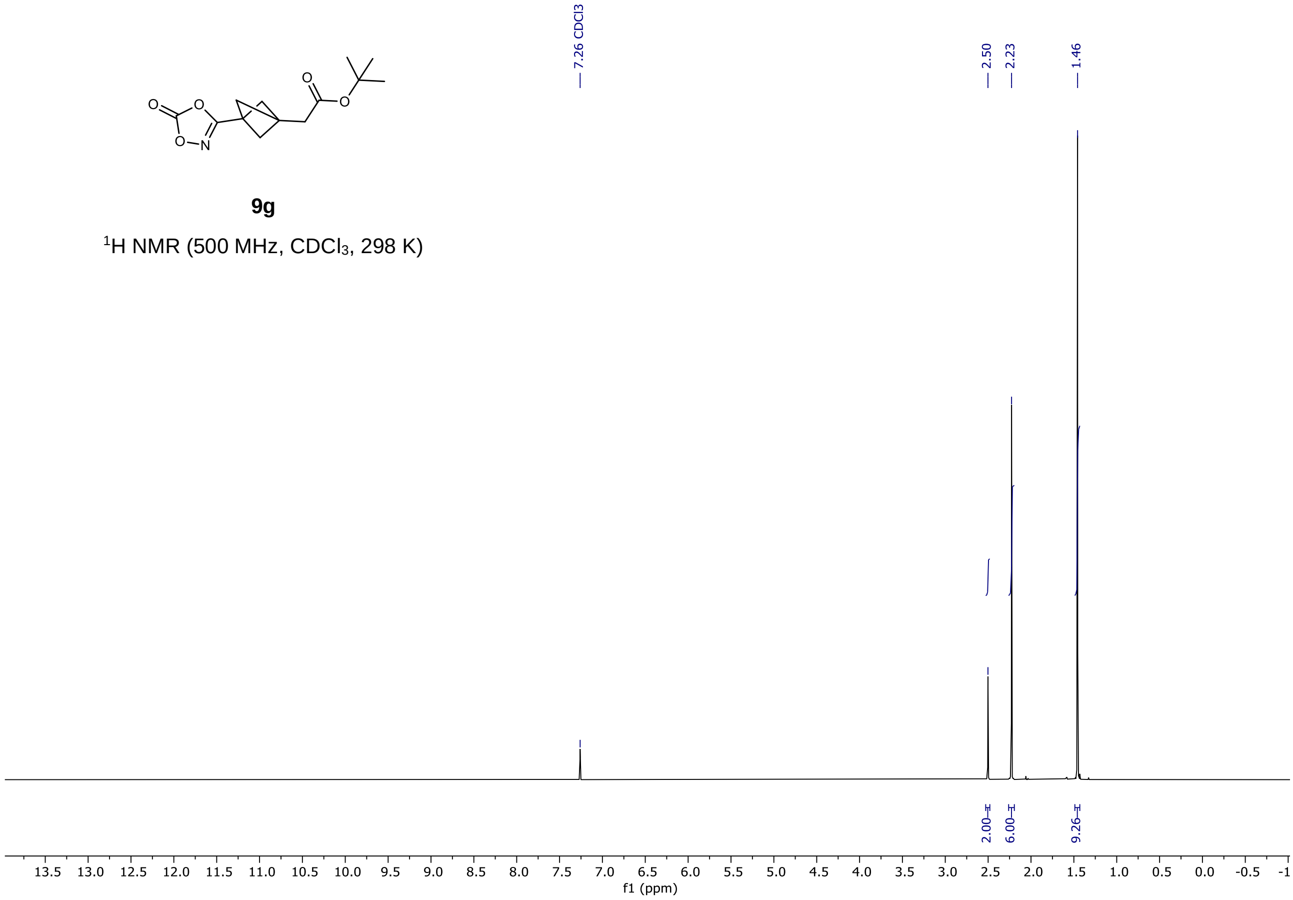

9g

^{1}H NMR (500 MHz, $CDCl_3$, 298 K)

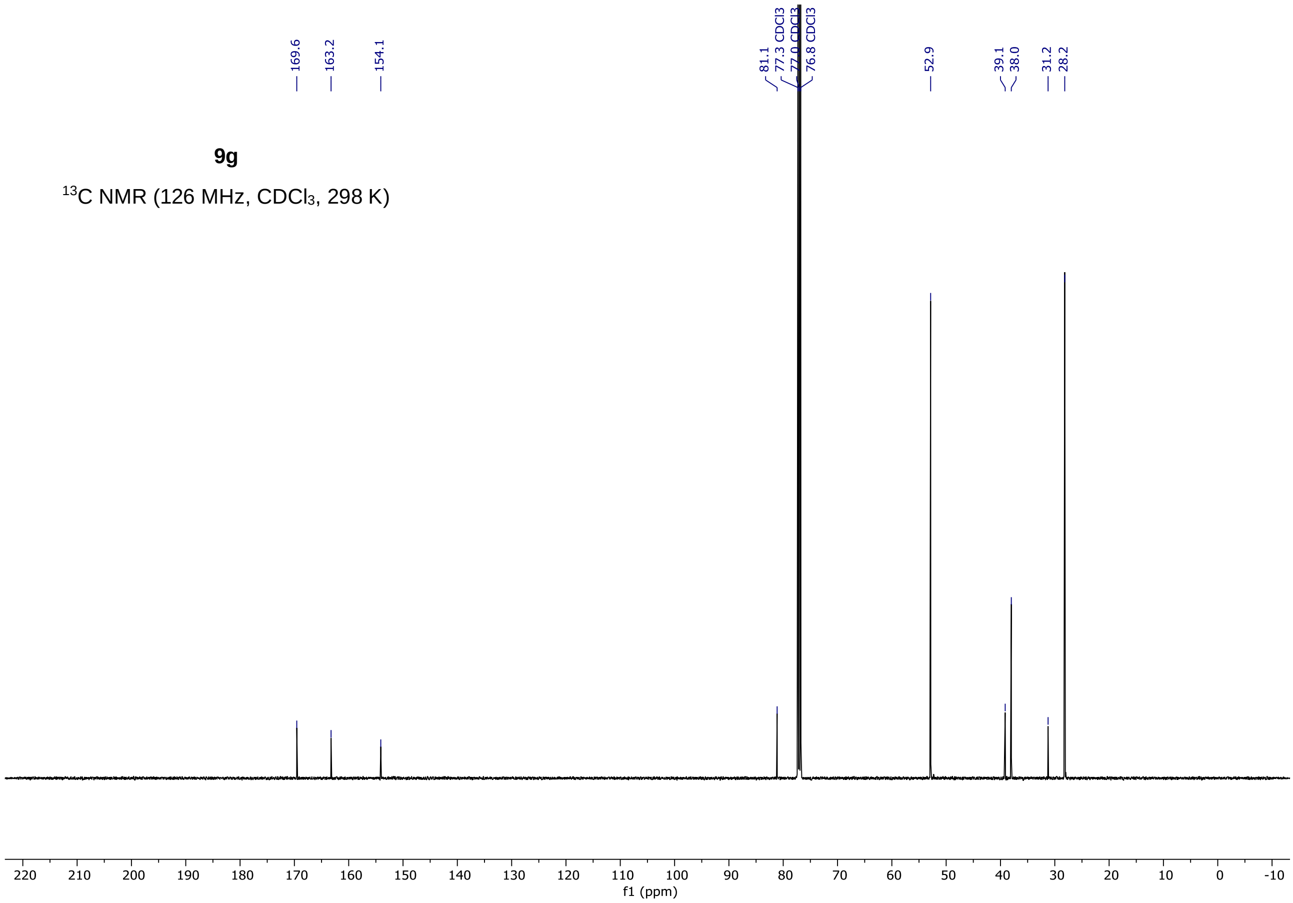

9g

^{13}C NMR (126 MHz, $CDCl_3$, 298 K)

Supplementary Figure 70. ^{1}H NMR (top) and ^{13}C NMR (bottom) spectra of compound **9g**. Frequency, temperature and solvent of measurement are indicated on each spectra.

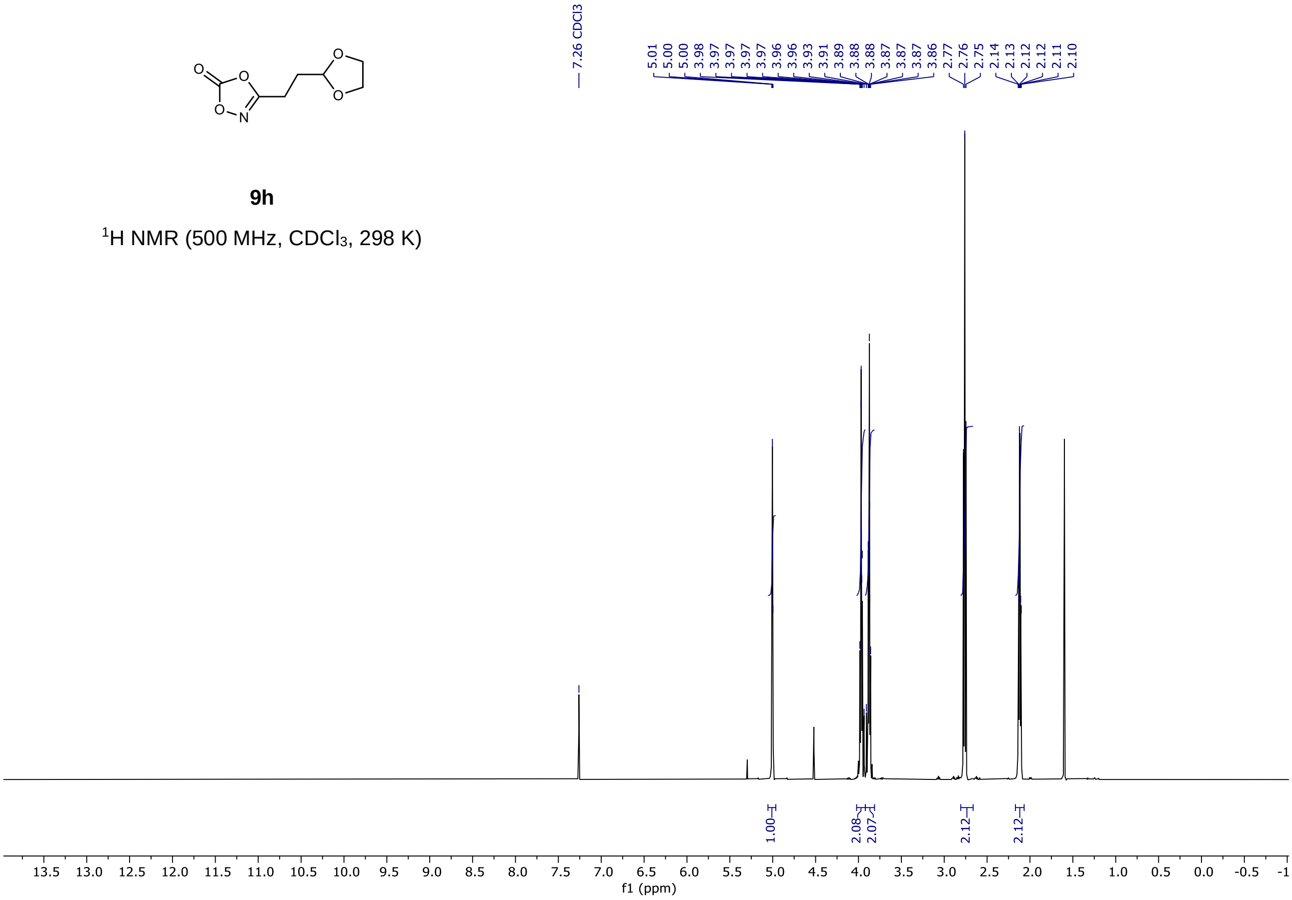

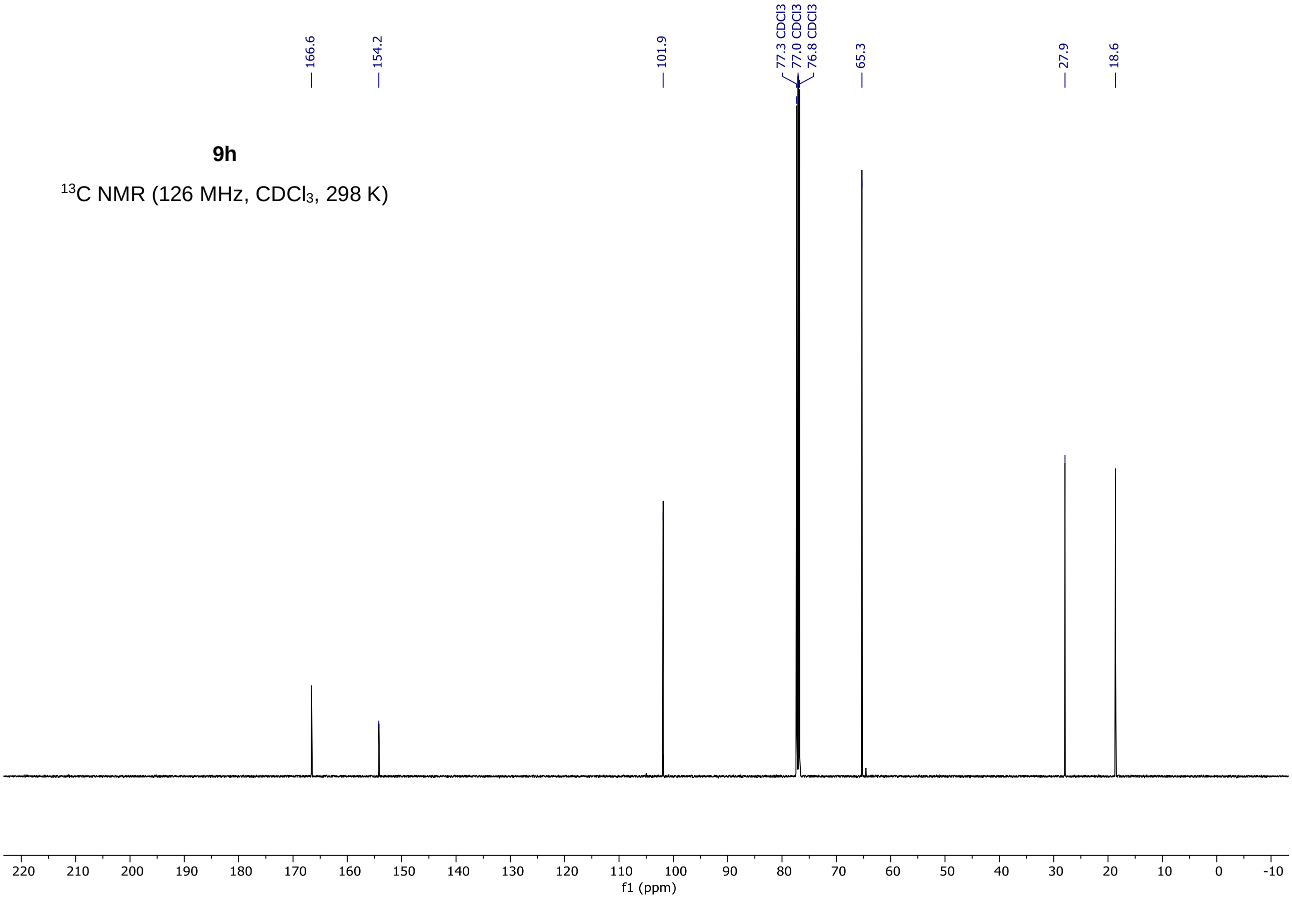

Supplementary Figure 71. ^{1}H NMR (top) and ^{13}C NMR (bottom) spectra of compound **9h**. Frequency, temperature and solvent of measurement are indicated on each spectra.

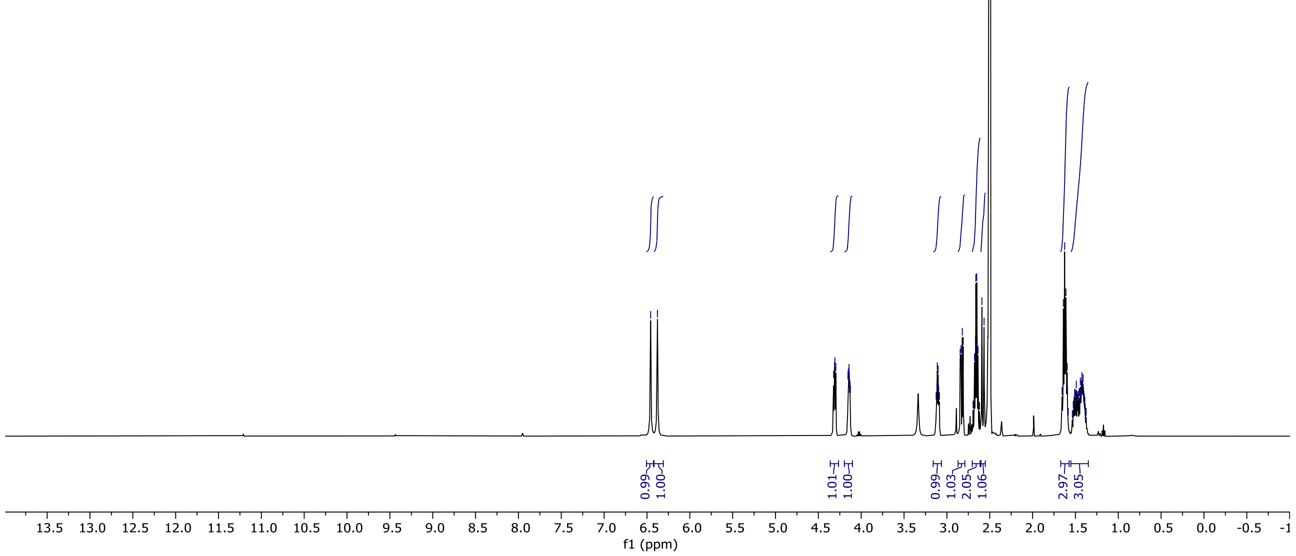

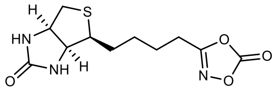

17

^{1}H NMR (500 MHz, DMSO-d_6, 298 K)

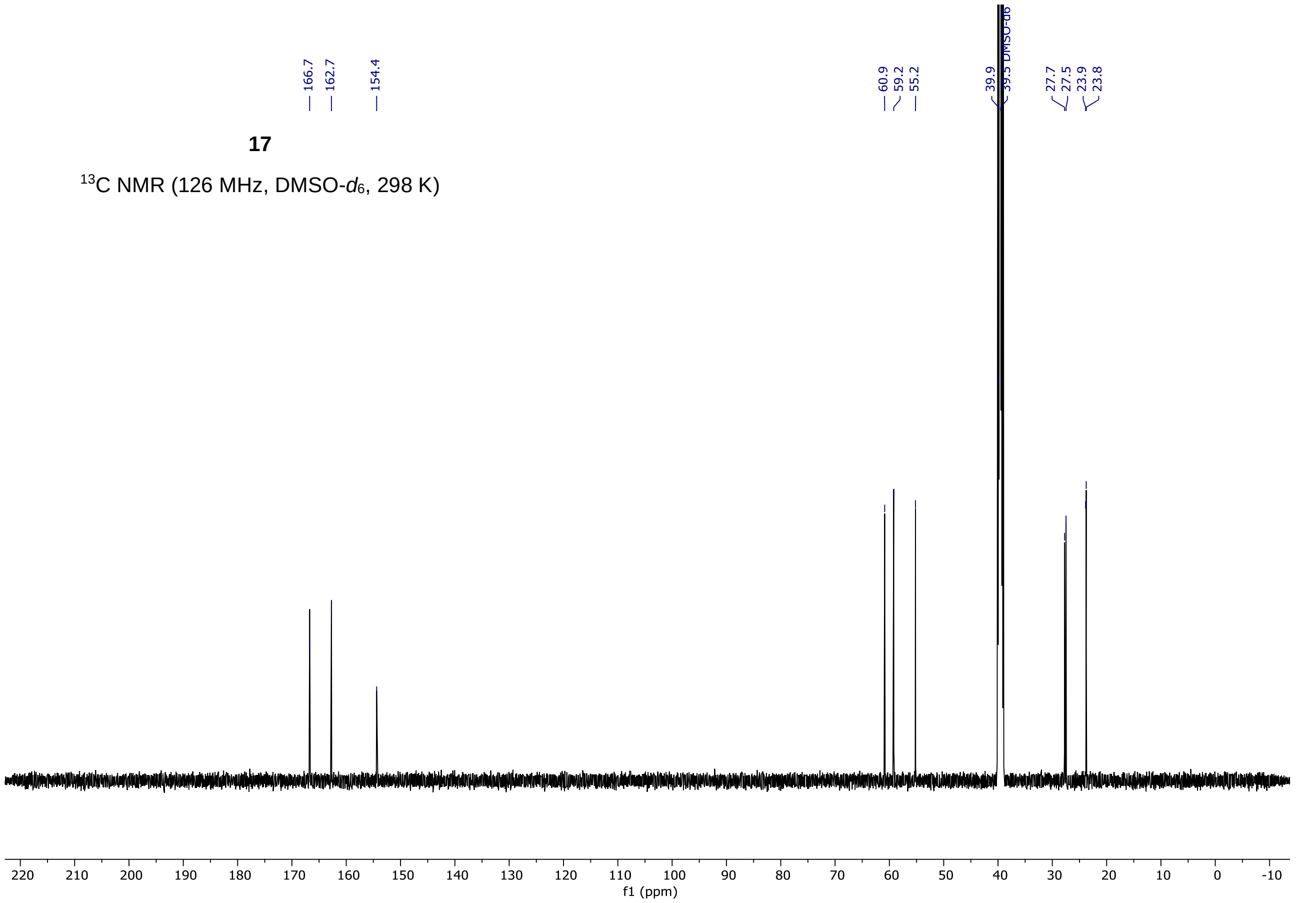

17

^{13}C NMR (126 MHz, DMSO-d_6, 298 K)

Supplementary Figure 72. ^{1}H NMR (top) and ^{13}C NMR (bottom) spectra of compound **17**. Frequency, temperature and solvent of measurement are indicated on each spectra.

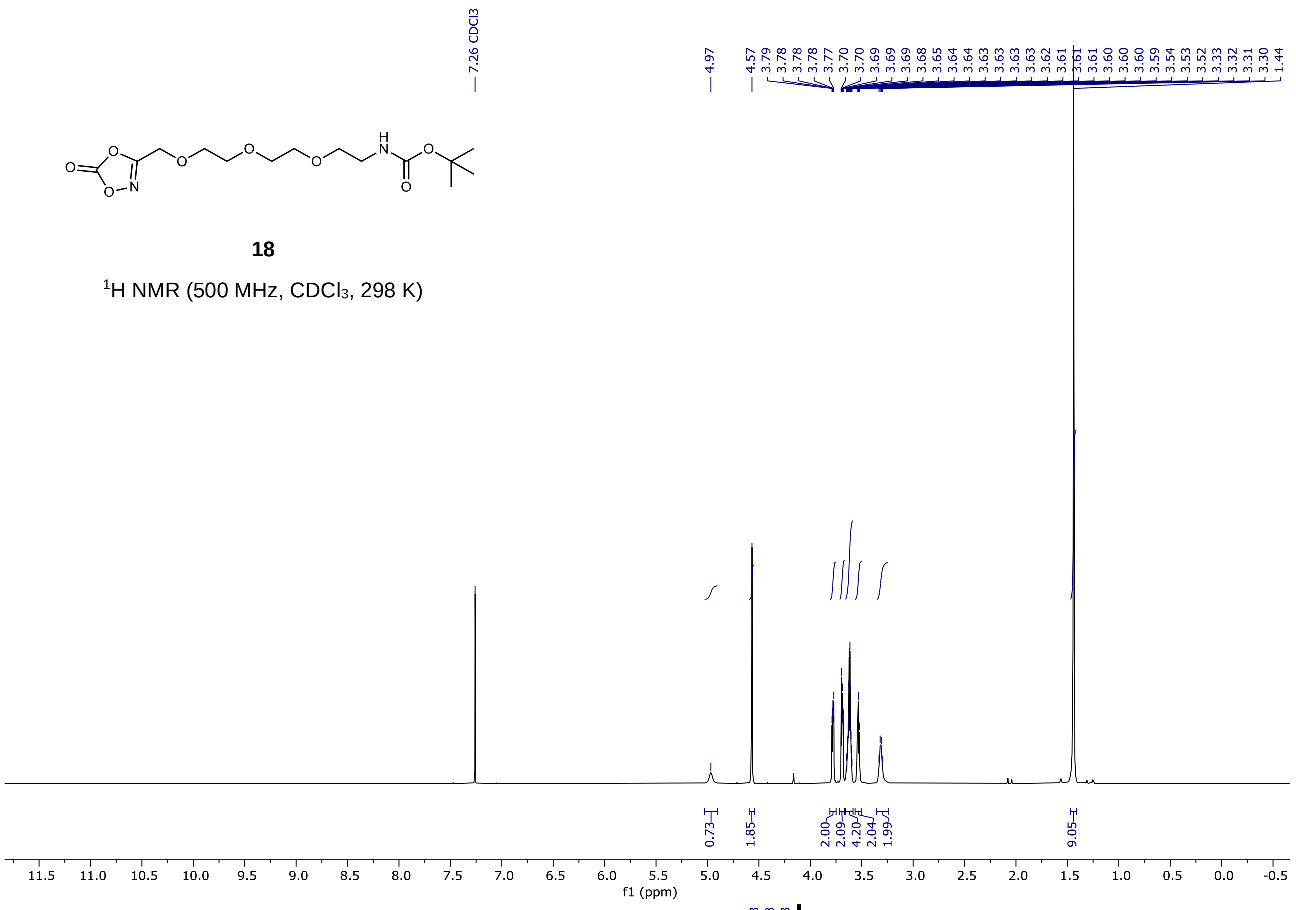

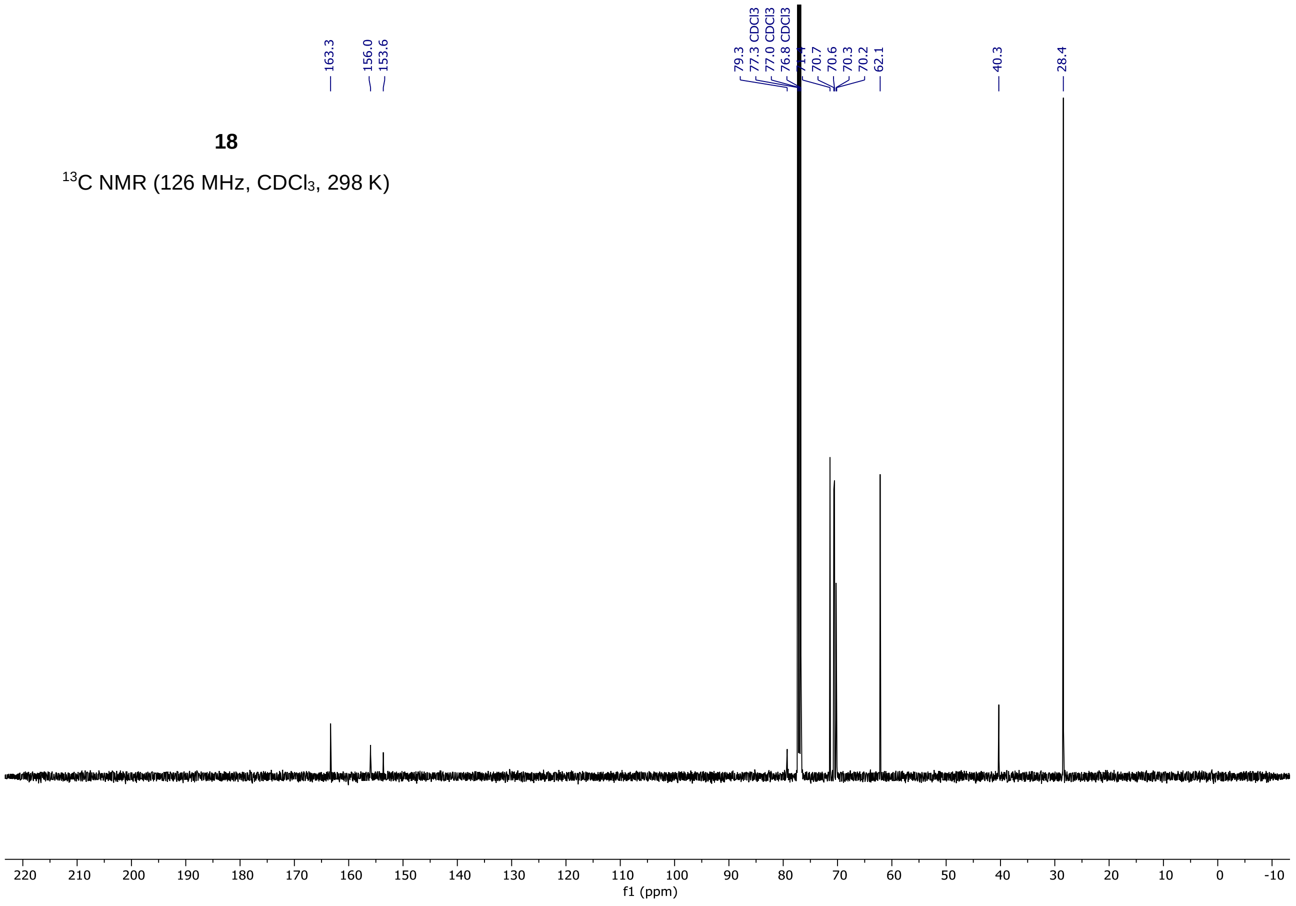

Supplementary Figure 73. ^{1}H NMR (top) and ^{13}C NMR (bottom) spectra of compound **18**. Frequency, temperature and solvent of measurement are indicated on each spectra.

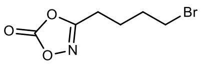

19

^{1}H NMR (500 MHz, $CDCl_3$, 298 K)

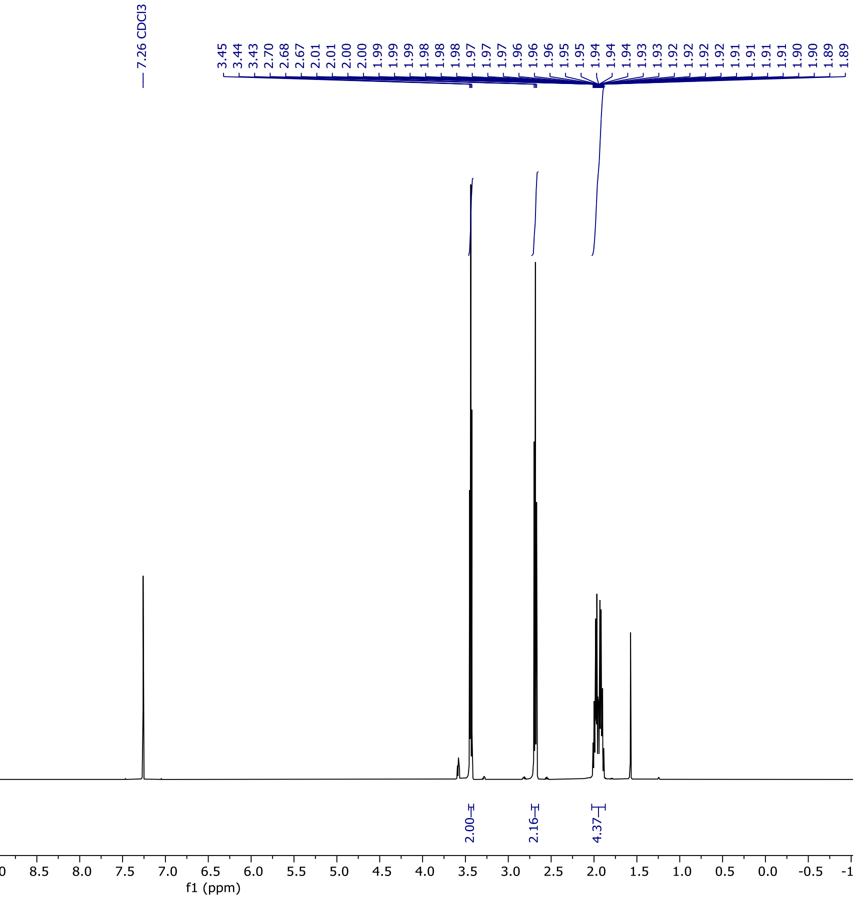

19

^{13}C NMR (126 MHz, $CDCl_3$, 298 K)

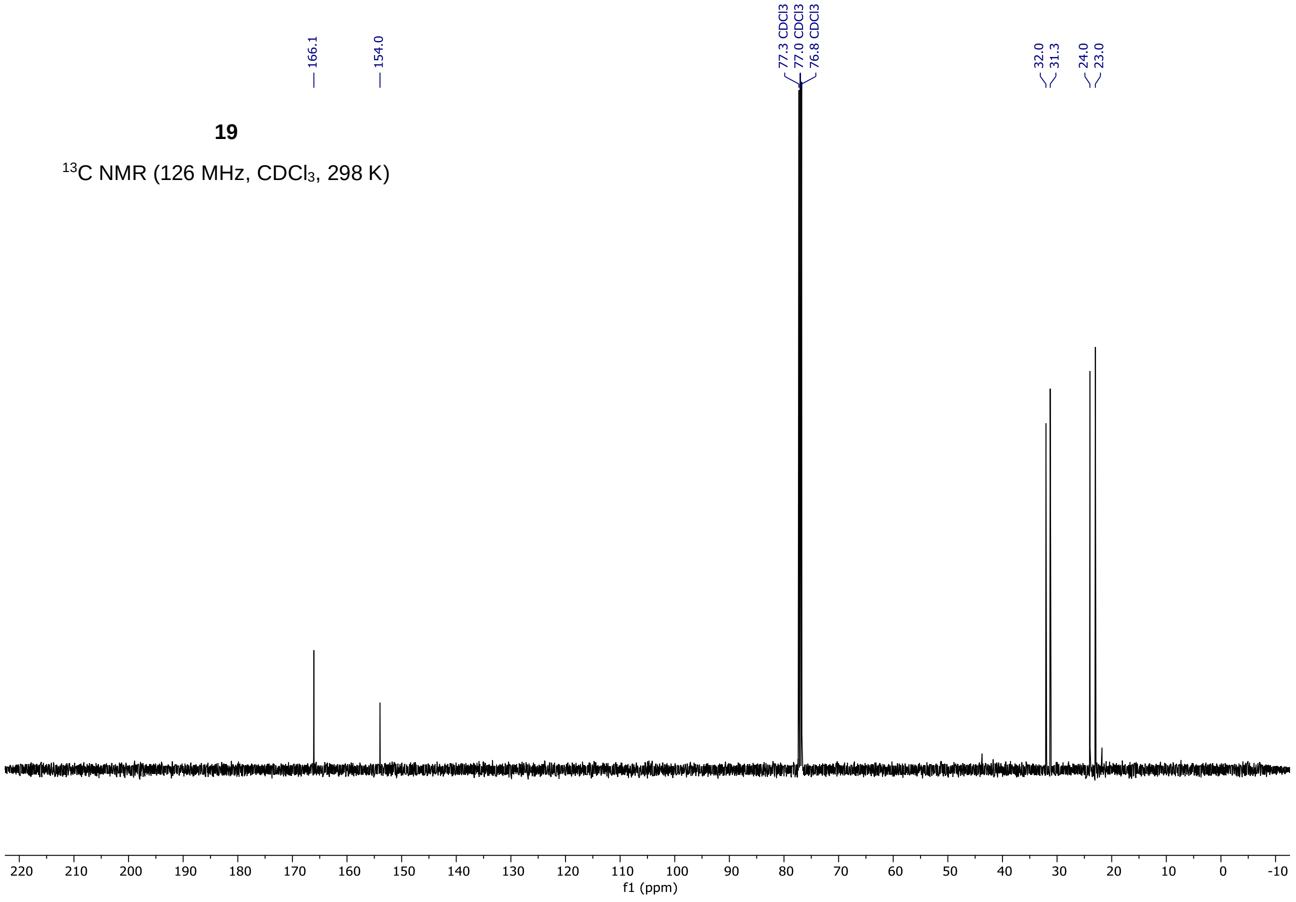

Supplementary Figure 74. ^{1}H NMR (top) and ^{13}C NMR (bottom) spectra of compound **19**. Frequency, temperature and solvent of measurement are indicated on each spectra.

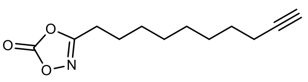

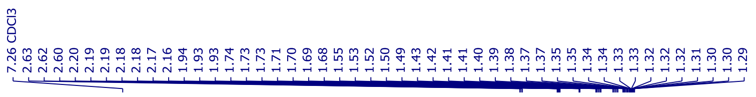

20

^{1}H NMR (500 MHz, $CDCl_3$, 298 K)

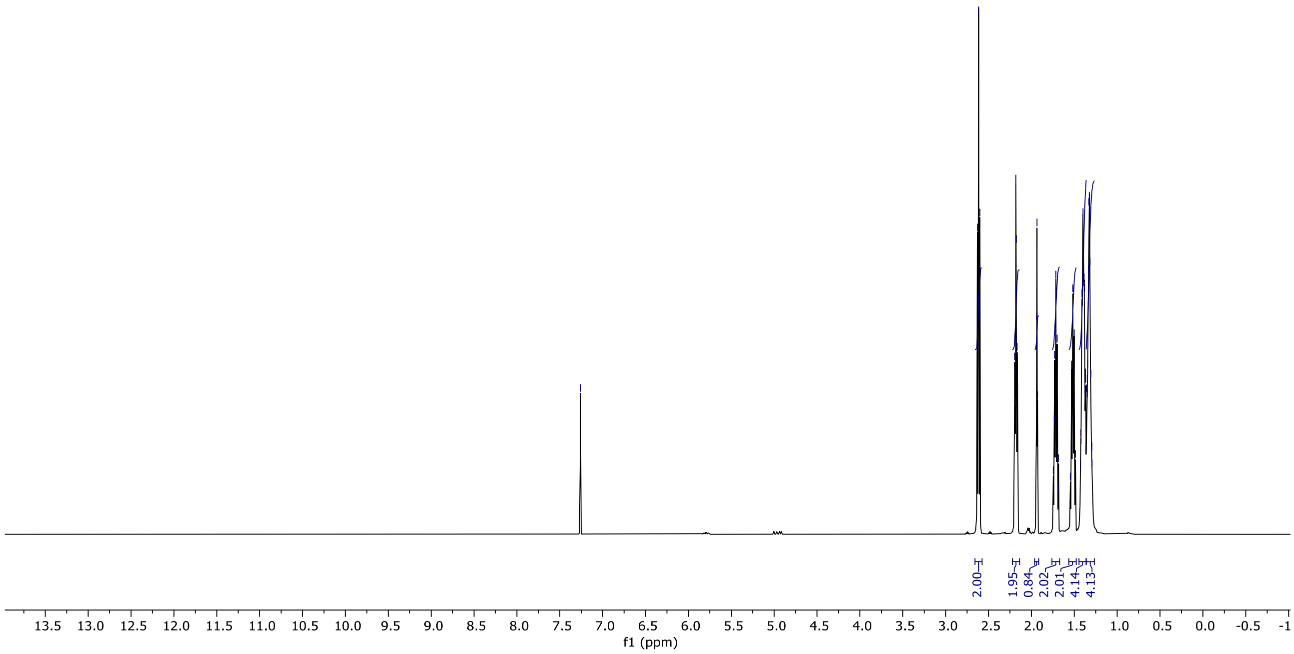

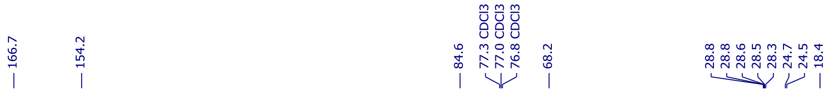

20

^{13}C NMR (126 MHz, $CDCl_3$, 298 K)

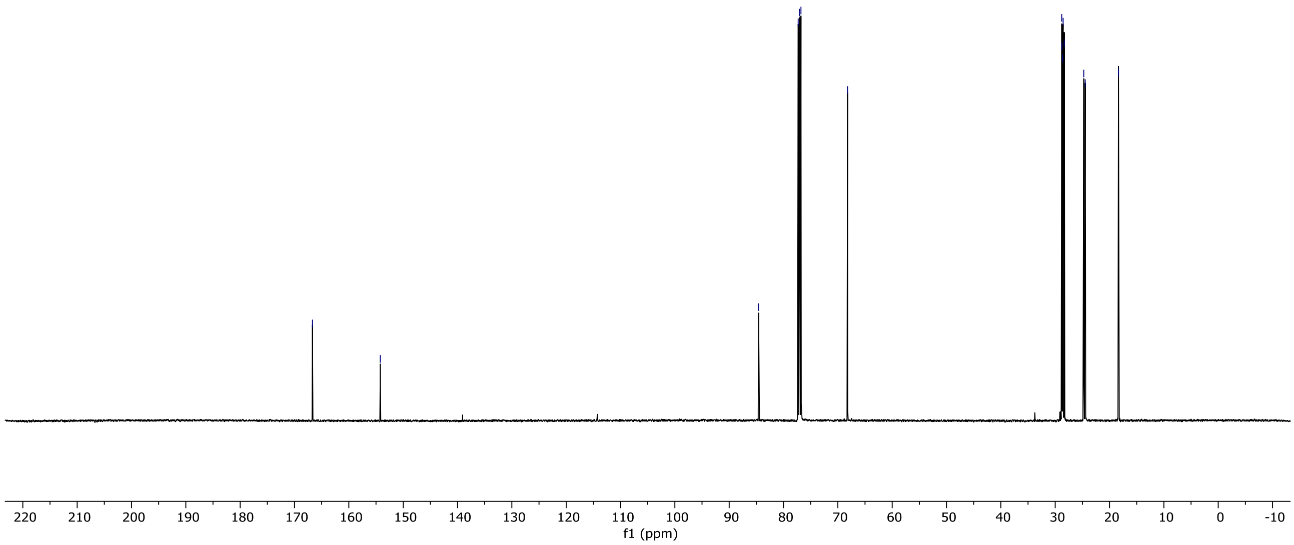

Supplementary Figure 75. ^{1}H NMR (top) and ^{13}C NMR (bottom) spectra of compound **20**. Frequency, temperature and solvent of measurement are indicated on each spectra.

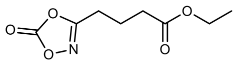

9j

^{1}H NMR (500 MHz, $CDCl_3$, 298 K)

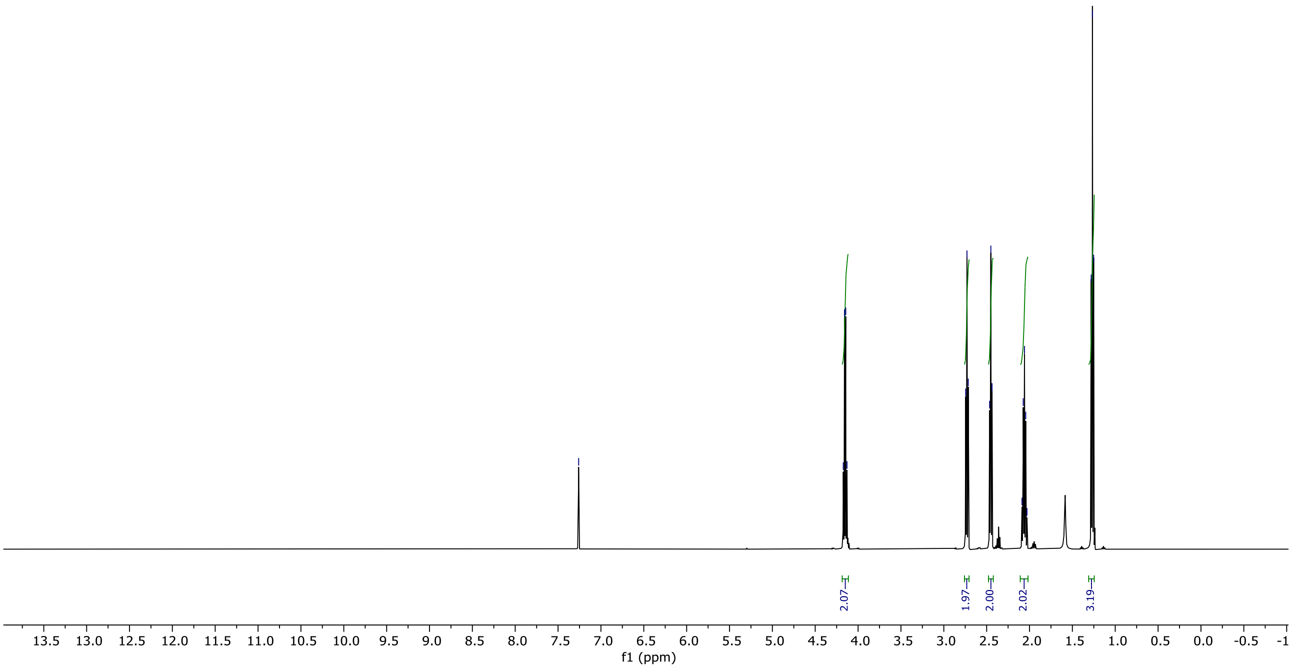

9j

^{13}C NMR (126 MHz, $CDCl_3$, 298 K)

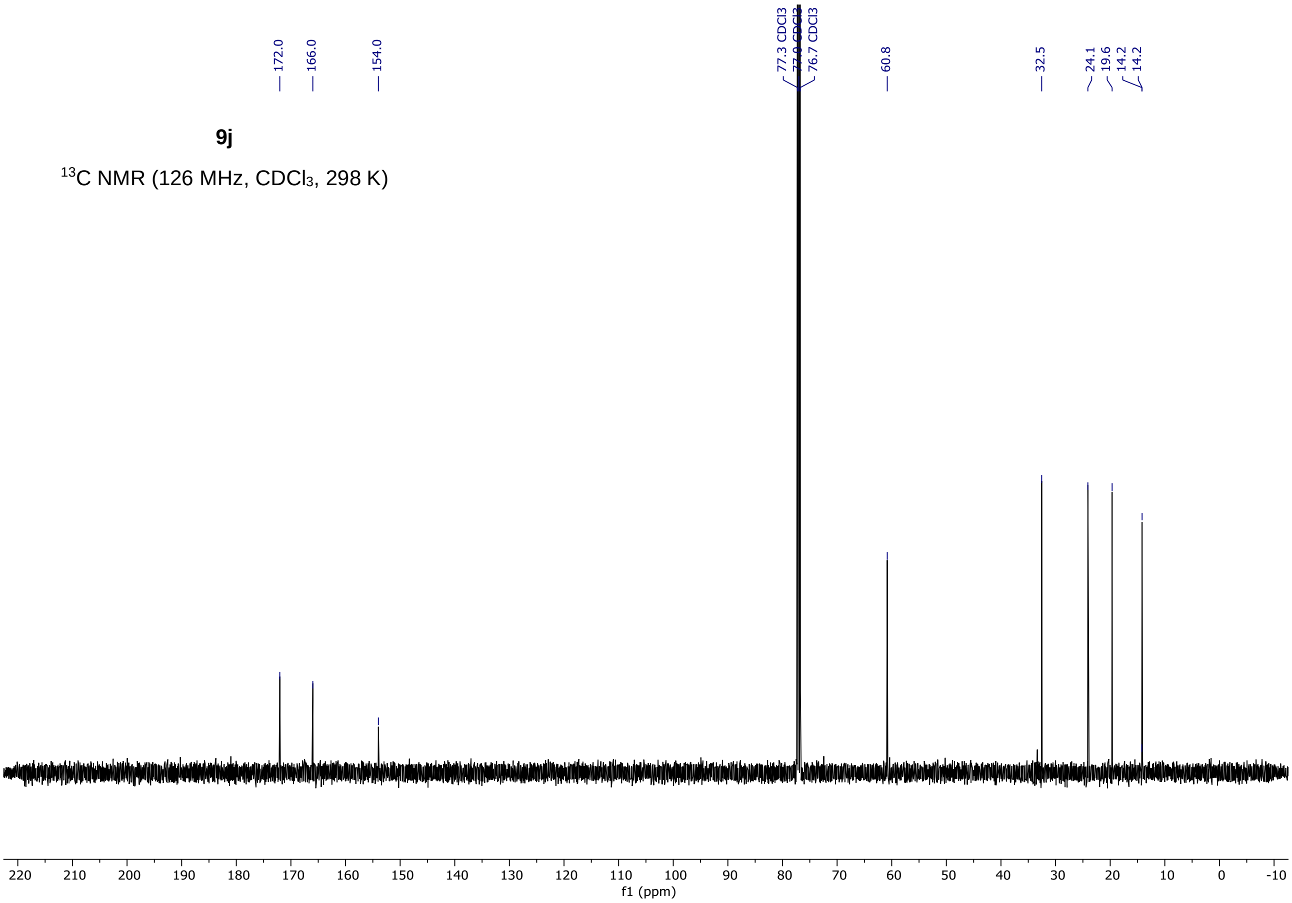

Supplementary Figure 76. ^{1}H NMR (top) and ^{13}C NMR (bottom) spectra of compound **9j**. Frequency, temperature and solvent of measurement are indicated on each spectra.

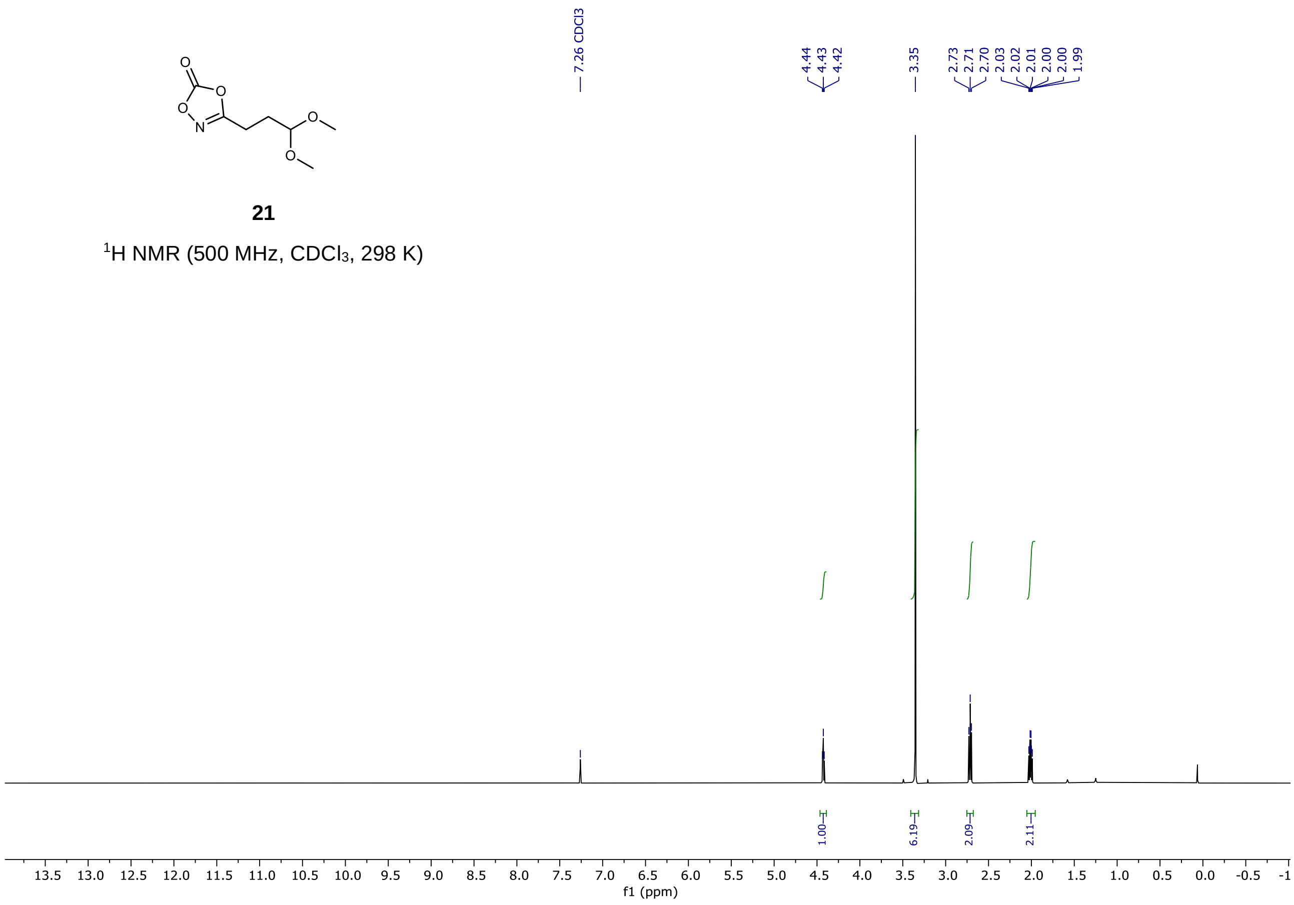

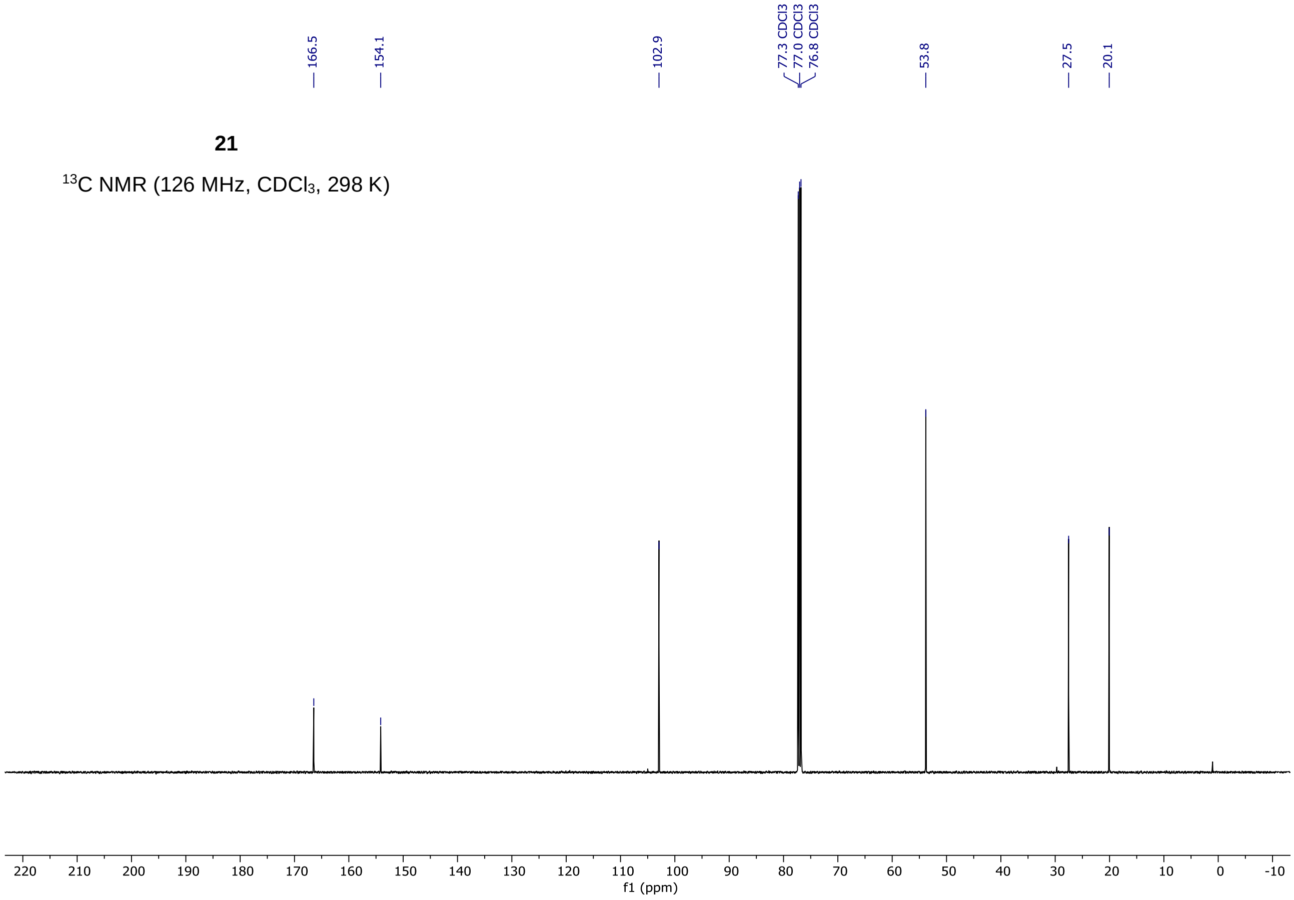

Supplementary Figure 77. ^{1}H NMR (top) and ^{13}C NMR (bottom) spectra of compound **21**. Frequency, temperature and solvent of measurement are indicated on each spectra.

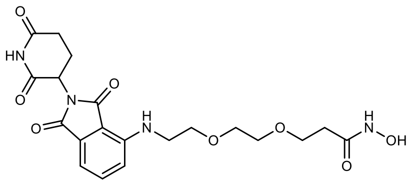

22

^{1}H NMR (500 MHz, CD_3OD, 298 K)

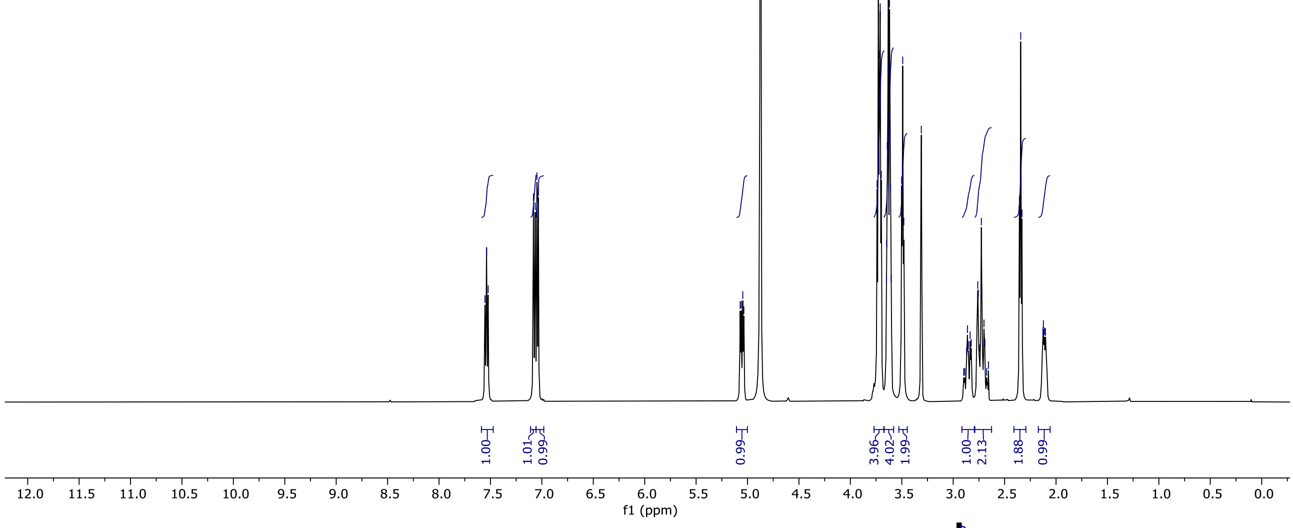

22

^{13}C NMR (126 MHz, CD_3OD, 298 K)

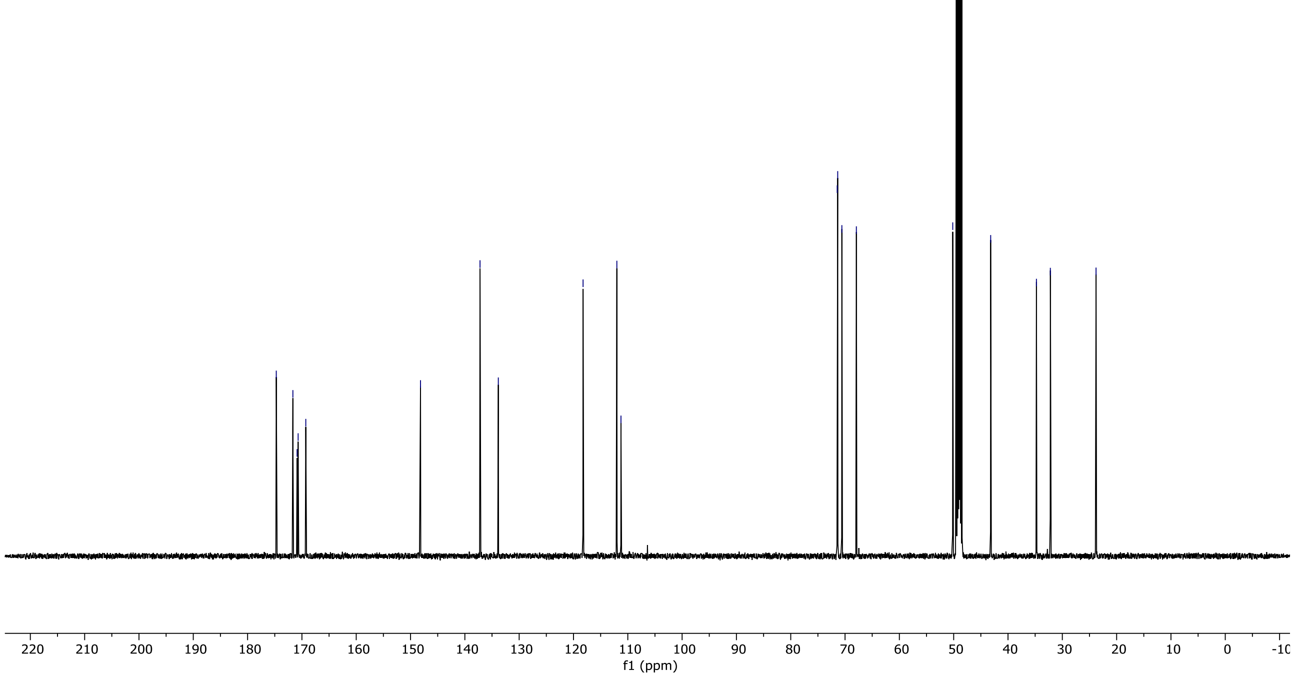

Supplementary Figure 78. ^{1}H NMR (top) and ^{13}C NMR (bottom) spectra of compound **22**. Frequency, temperature and solvent of measurement are indicated on each spectra.

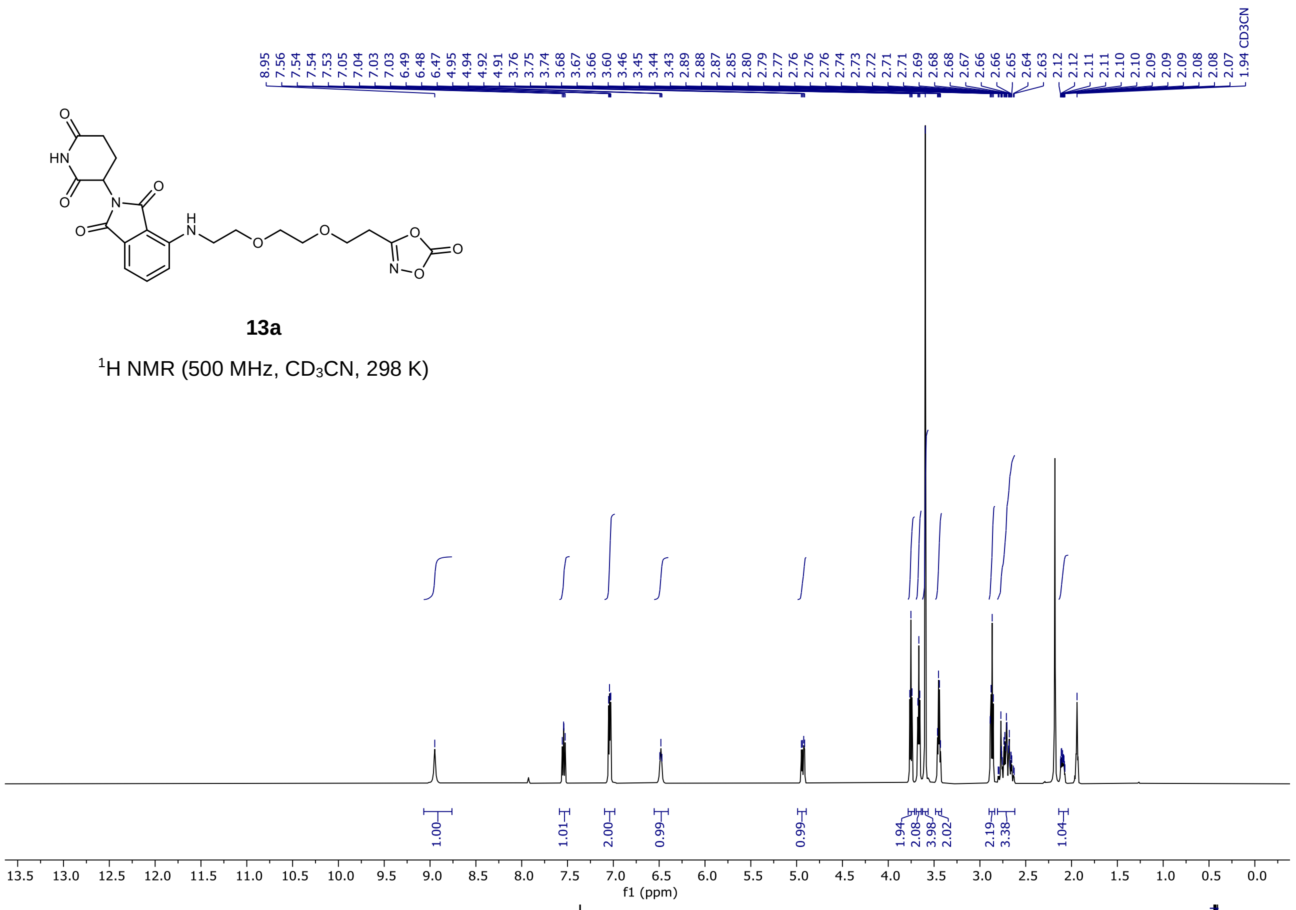

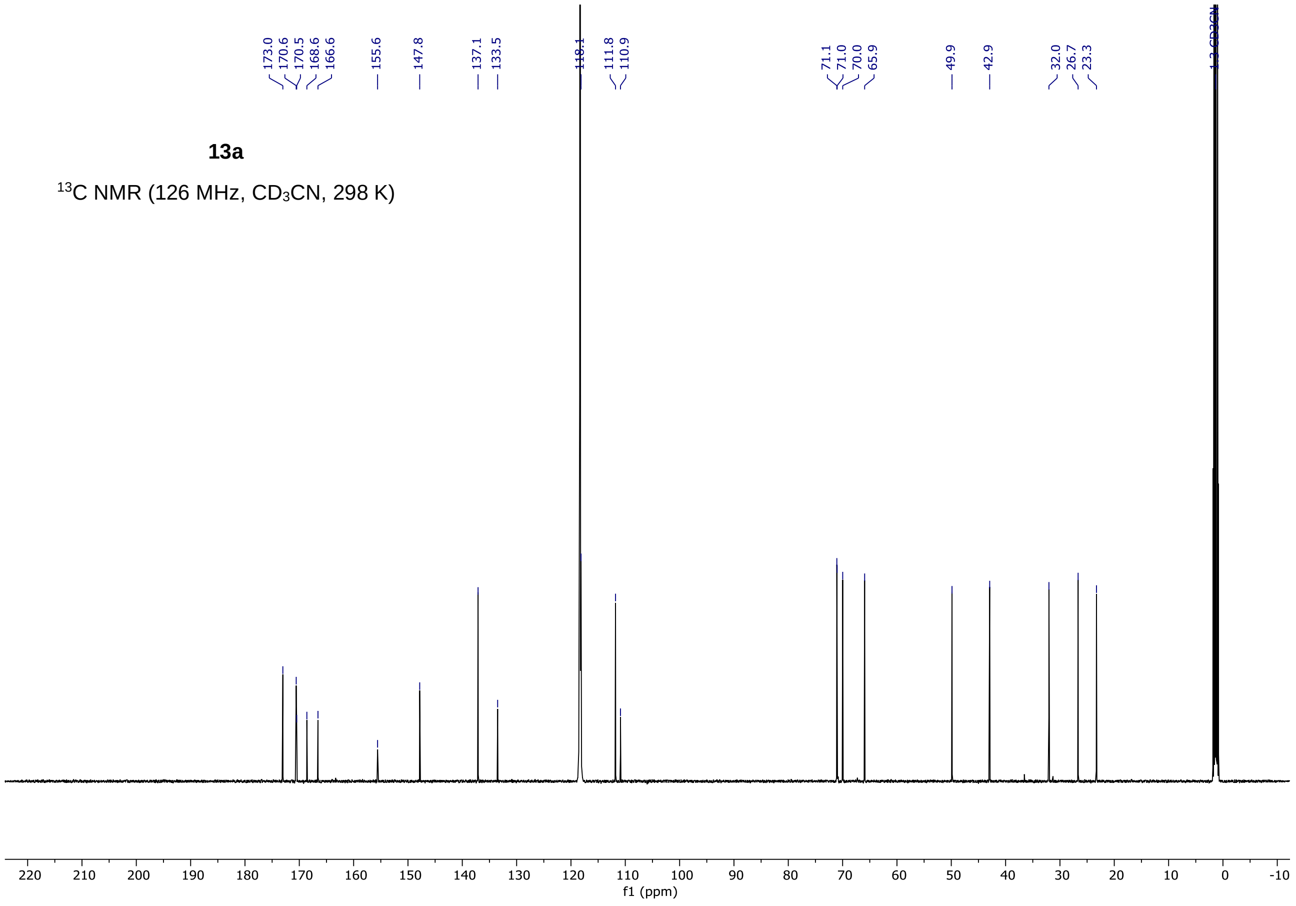

Supplementary Figure 79. ^{1}H NMR (top) and ^{13}C NMR (bottom) spectra of compound **13a**. Frequency, temperature and solvent of measurement are indicated on each spectra.

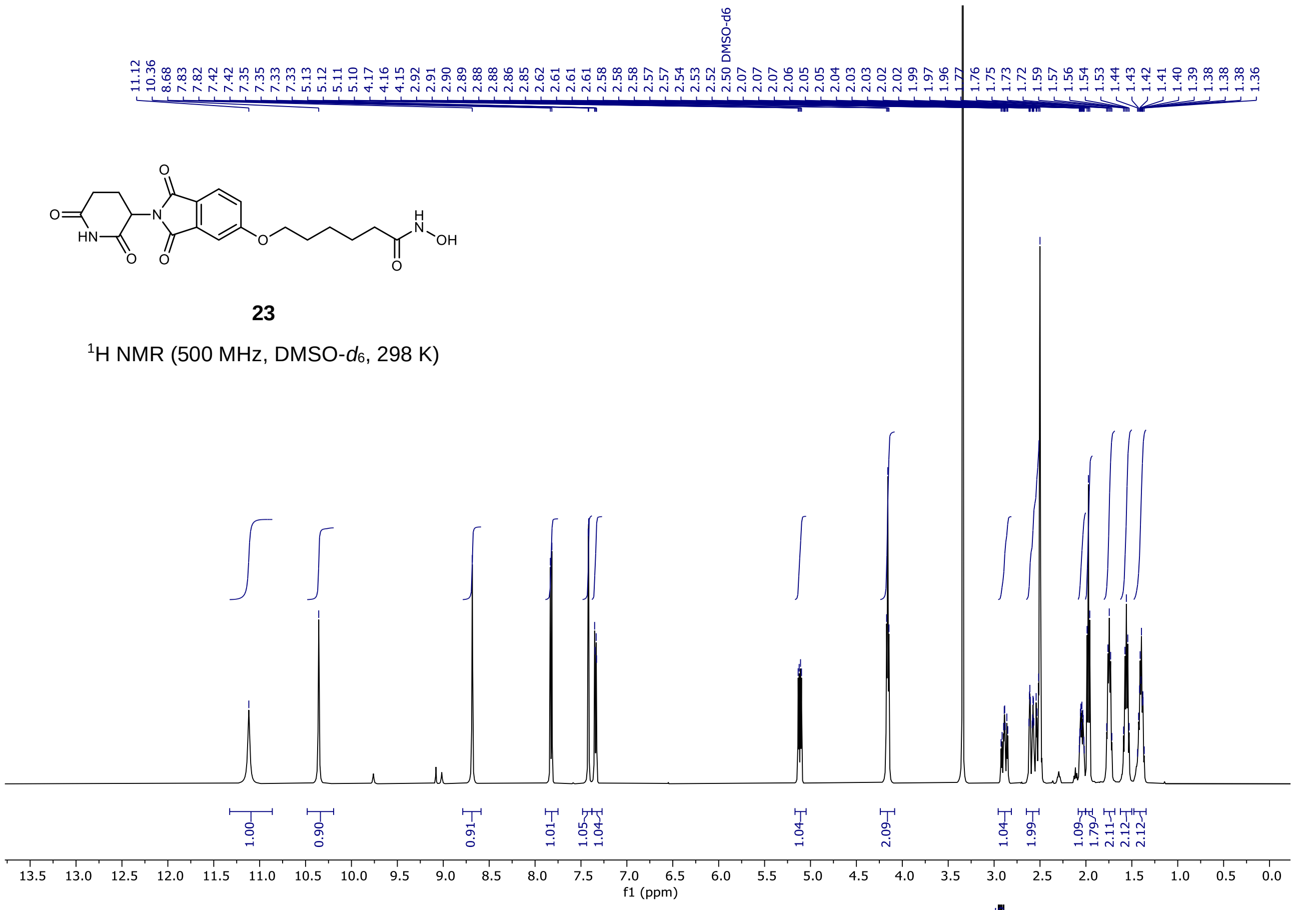

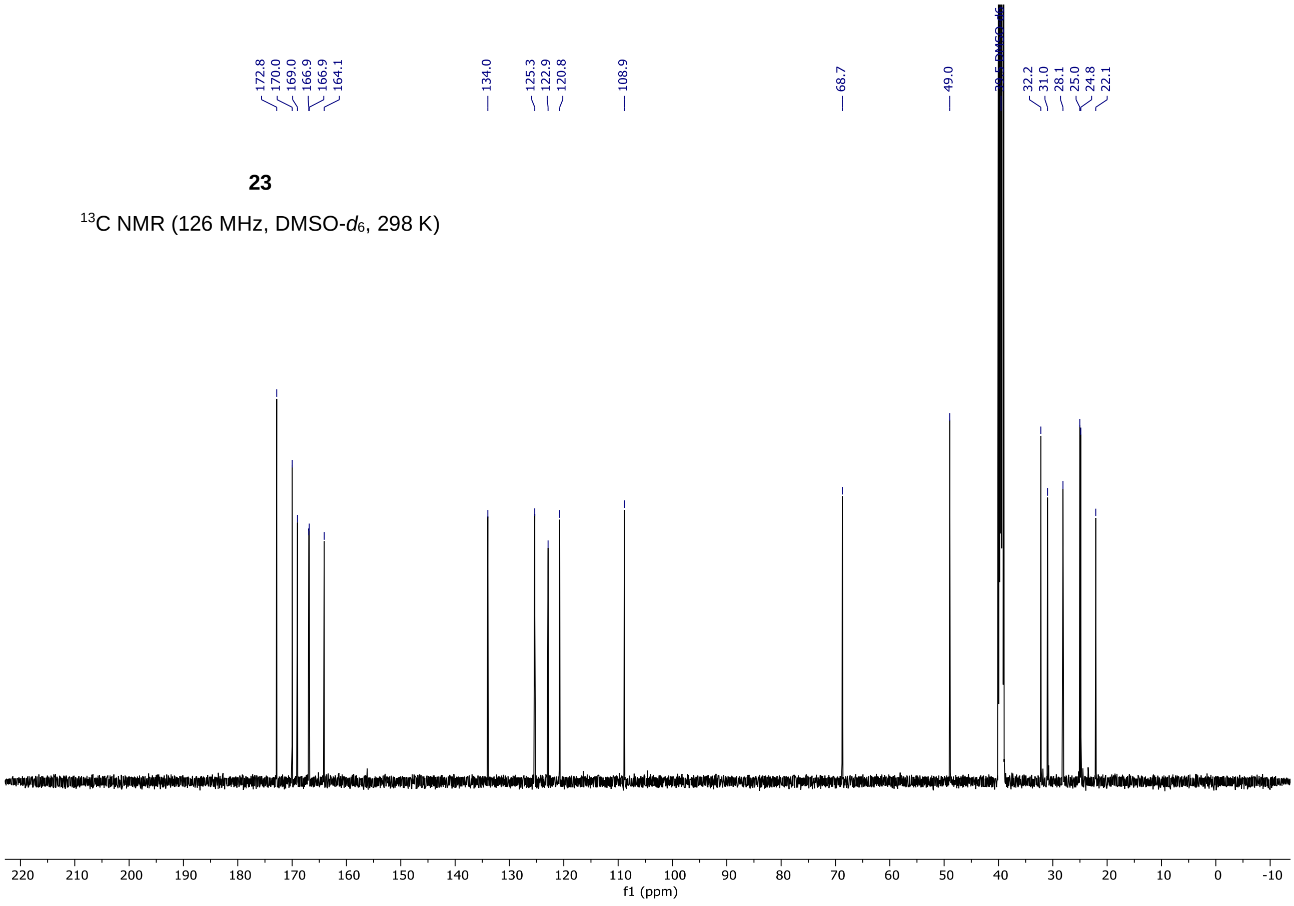

Supplementary Figure 80. ^{1}H NMR (top) and ^{13}C NMR (bottom) spectra of compound **23**. Frequency, temperature and solvent of measurement are indicated on each spectra.

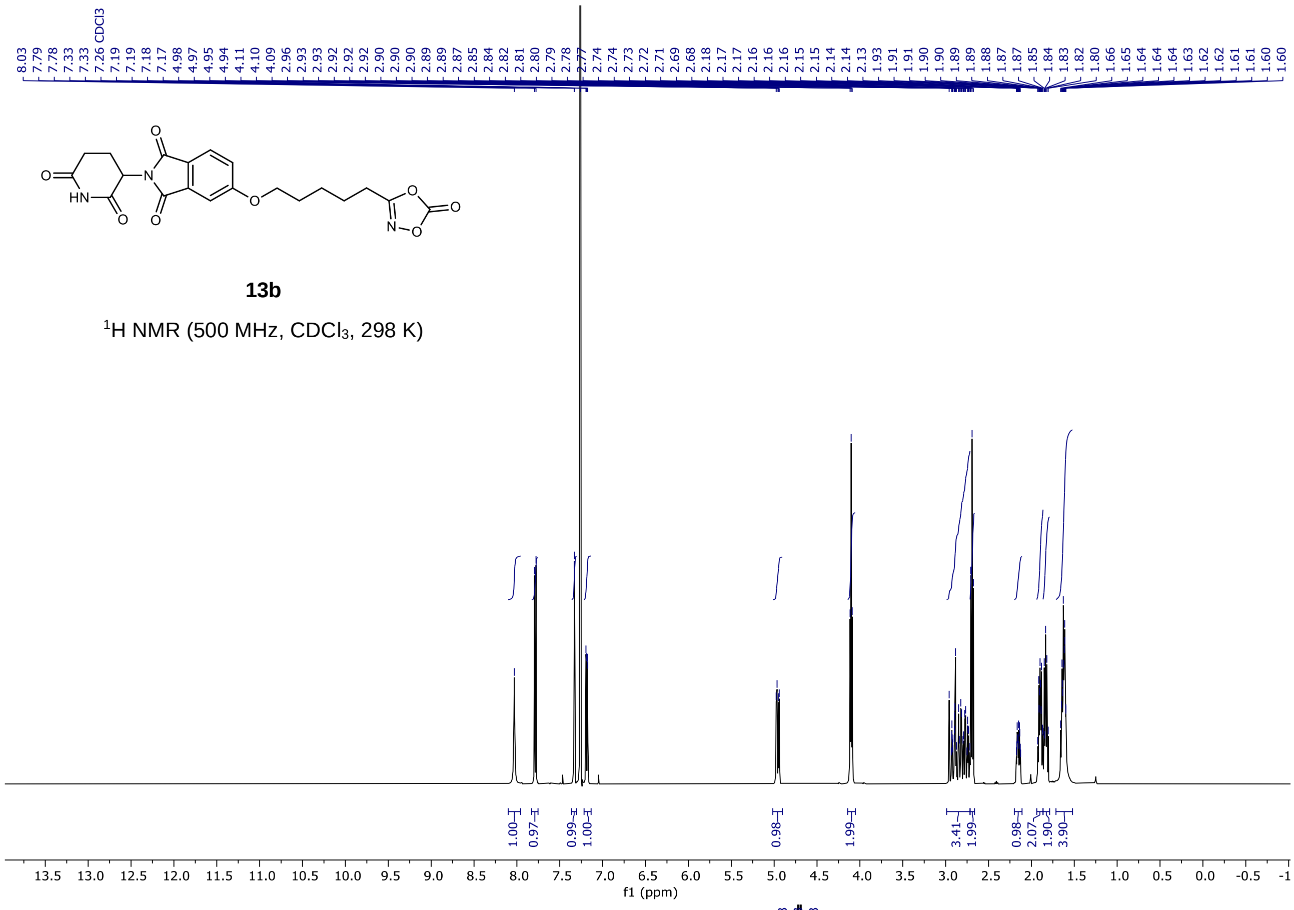

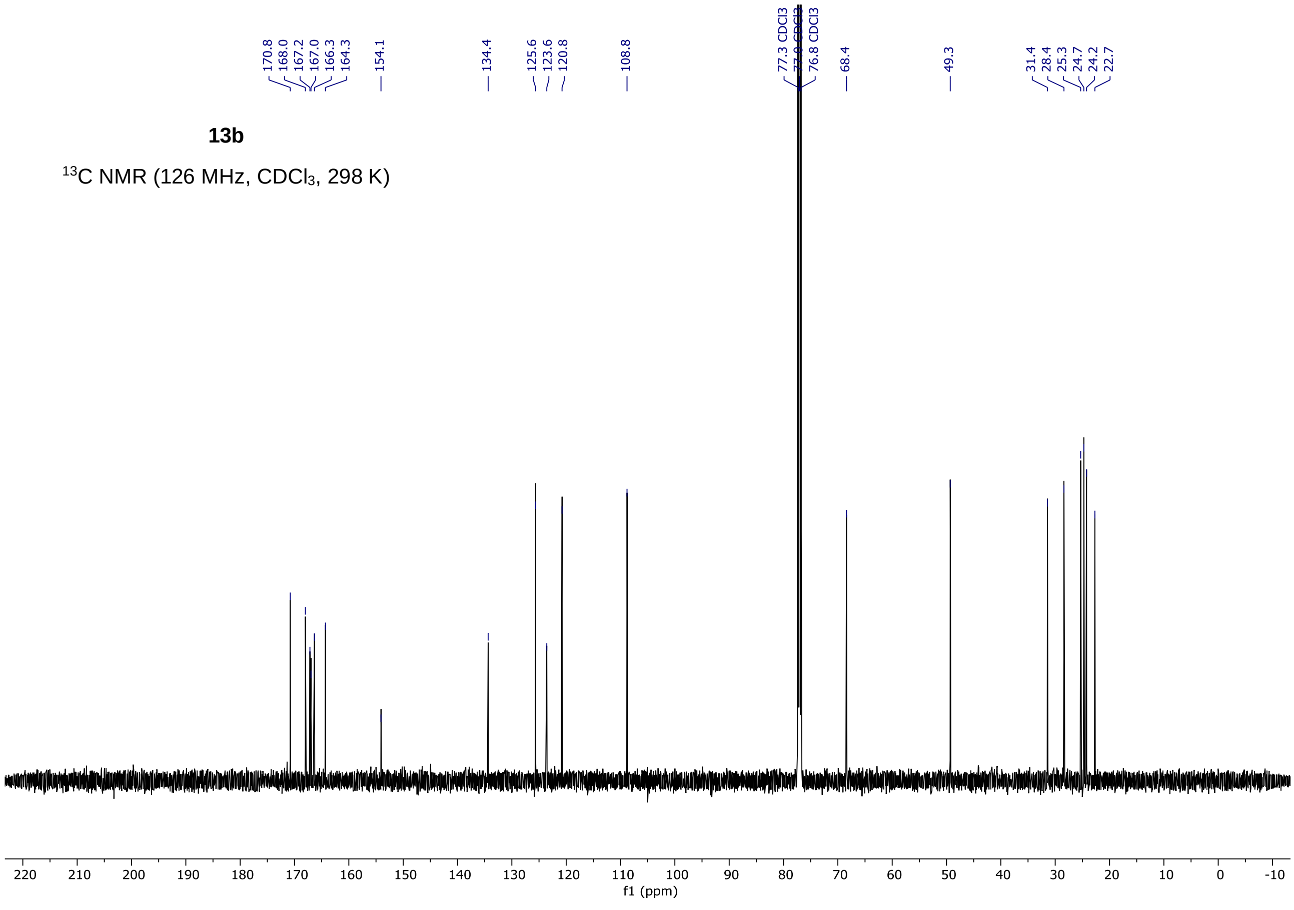

Supplementary Figure 81. ^{1}H NMR (top) and ^{13}C NMR (bottom) spectra of compound **13b**. Frequency, temperature and solvent of measurement are indicated on each spectra.

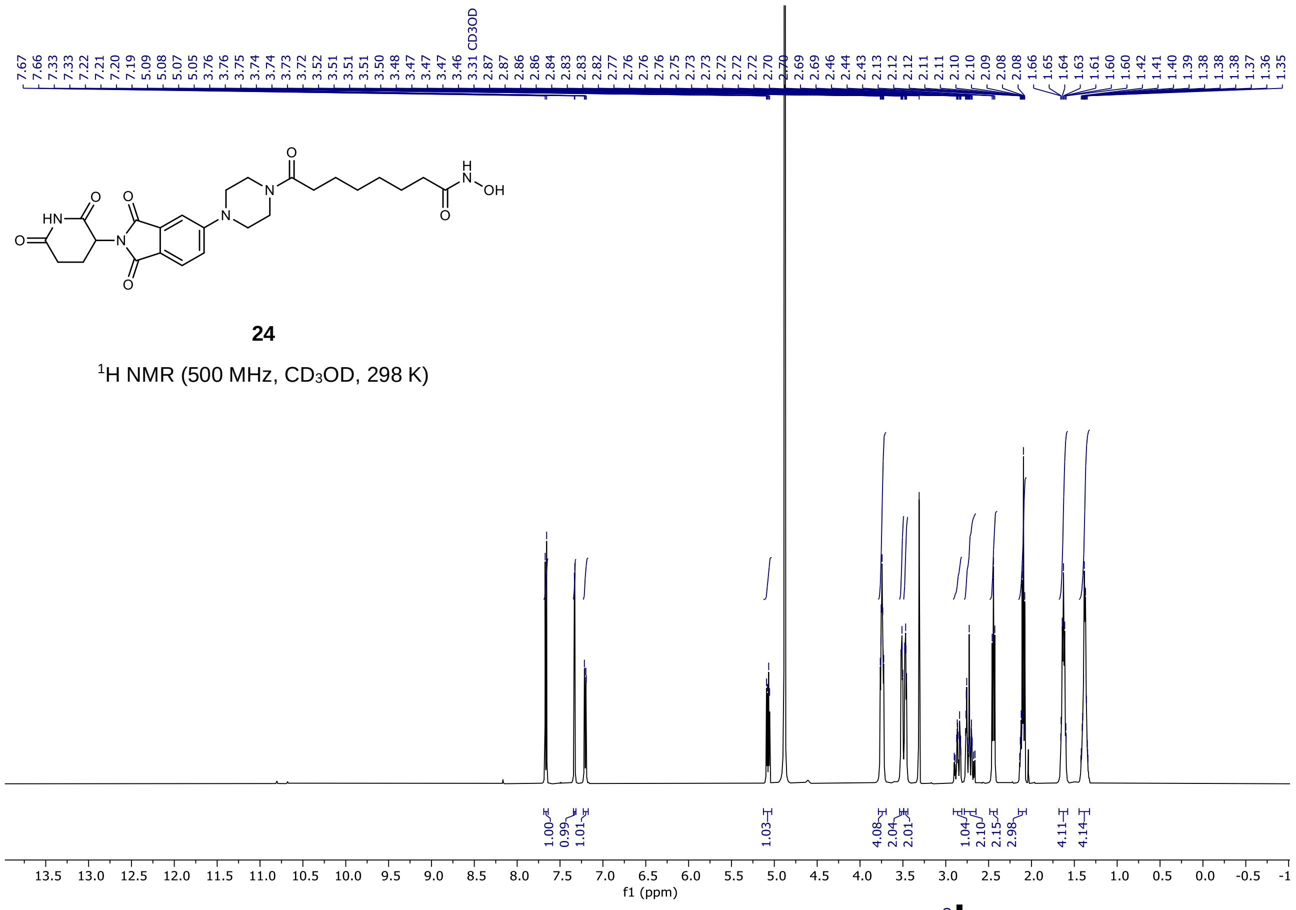

24

^{1}H NMR (500 MHz, CD_3OD, 298 K)

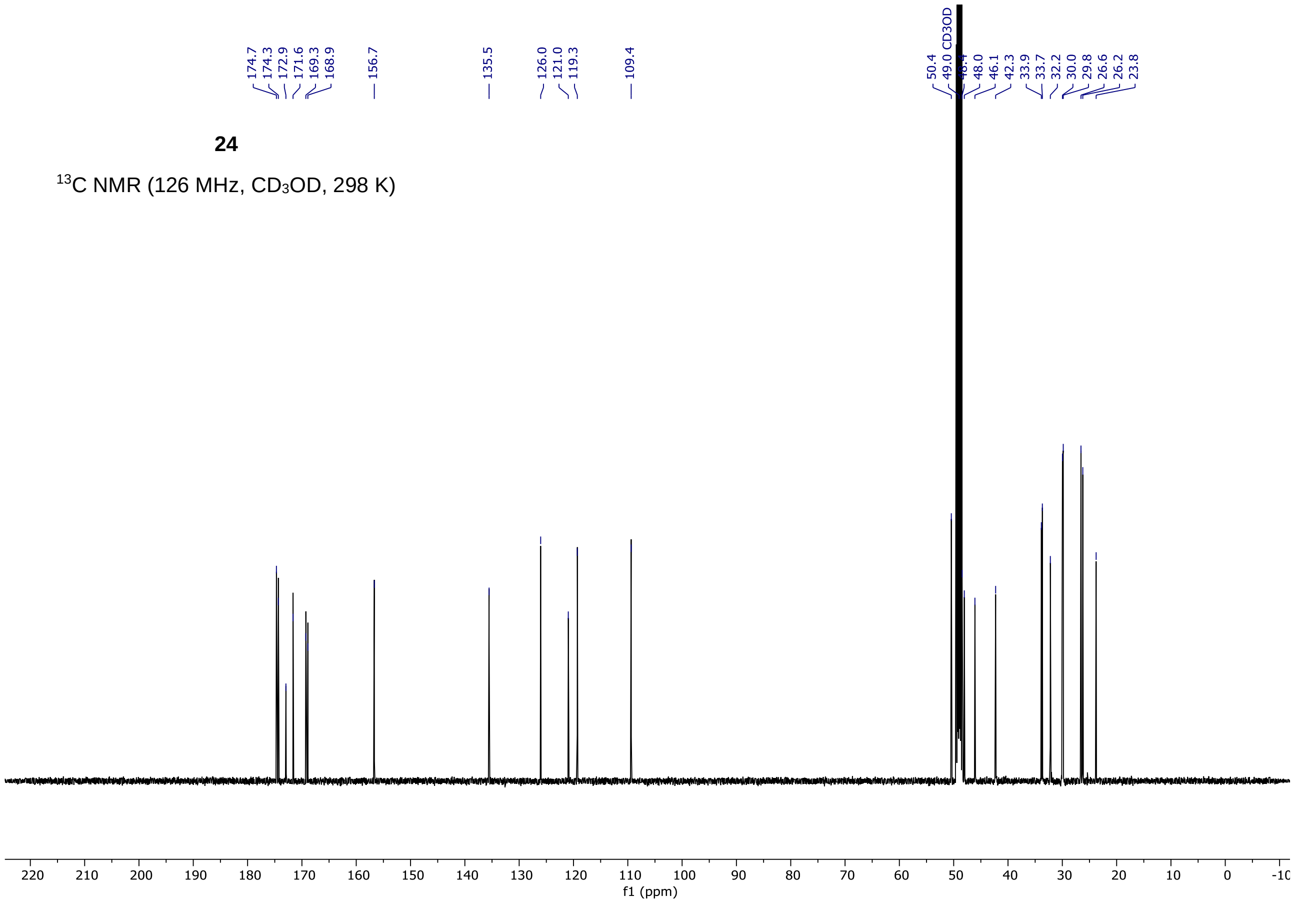

24

^{13}C NMR (126 MHz, CD_3OD, 298 K)

Supplementary Figure 82. ^{1}H NMR (top) and ^{13}C NMR (bottom) spectra of compound **24**. Frequency, temperature and solvent of measurement are indicated on each spectra.

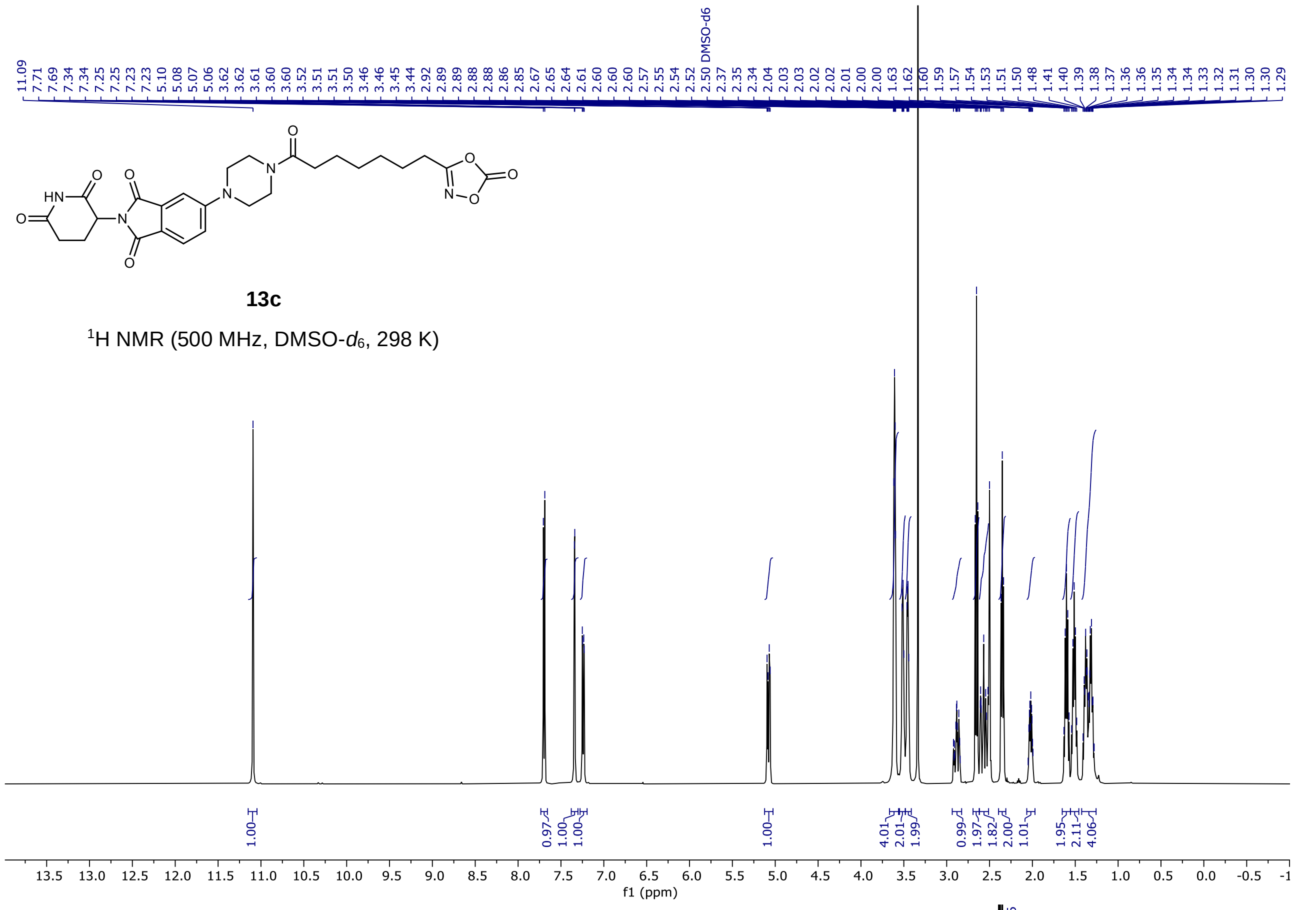

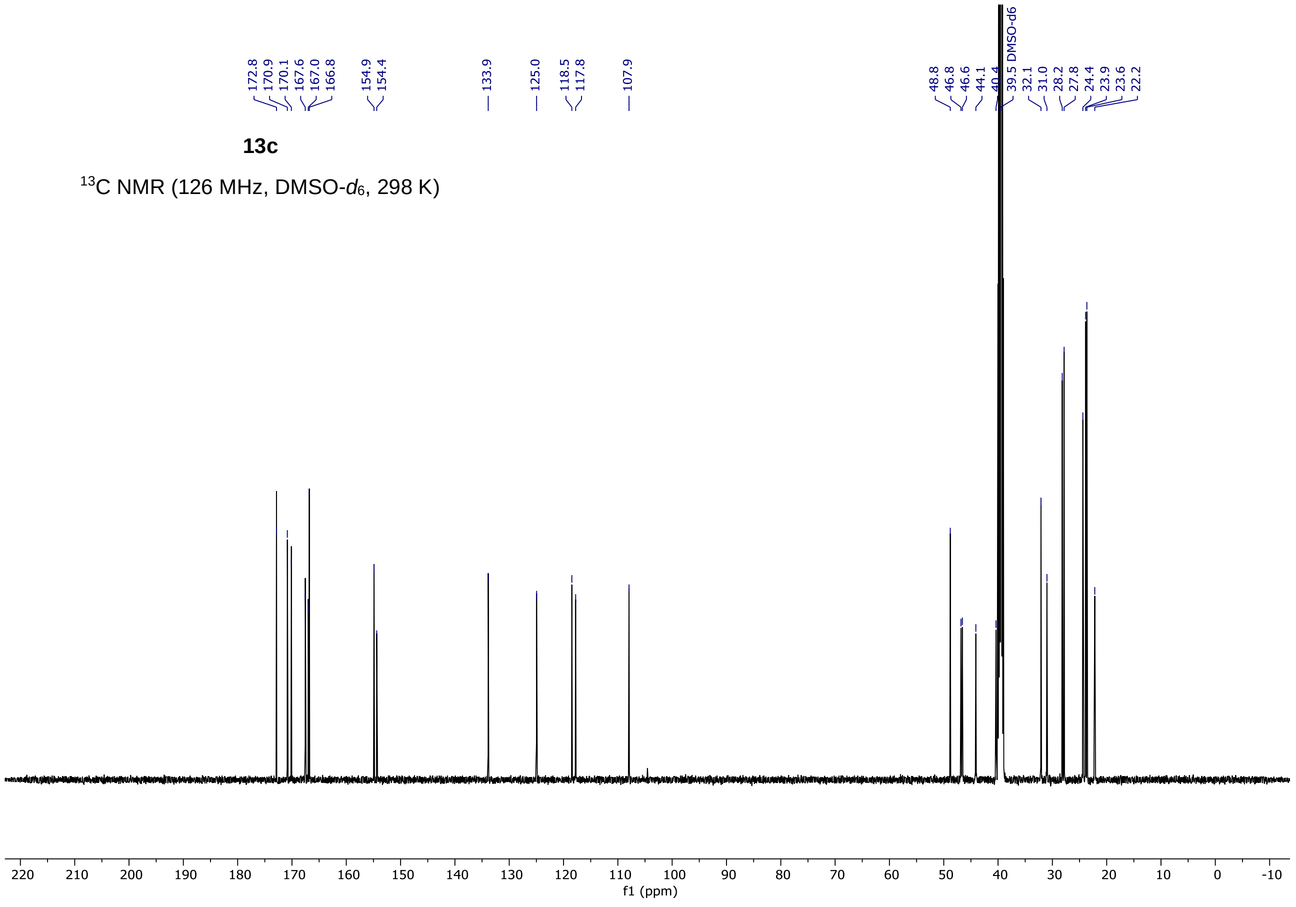

Supplementary Figure 83. ^{1}H NMR (top) and ^{13}C NMR (bottom) spectra of compound **13c**. Frequency, temperature and solvent of measurement are indicated on each spectra.

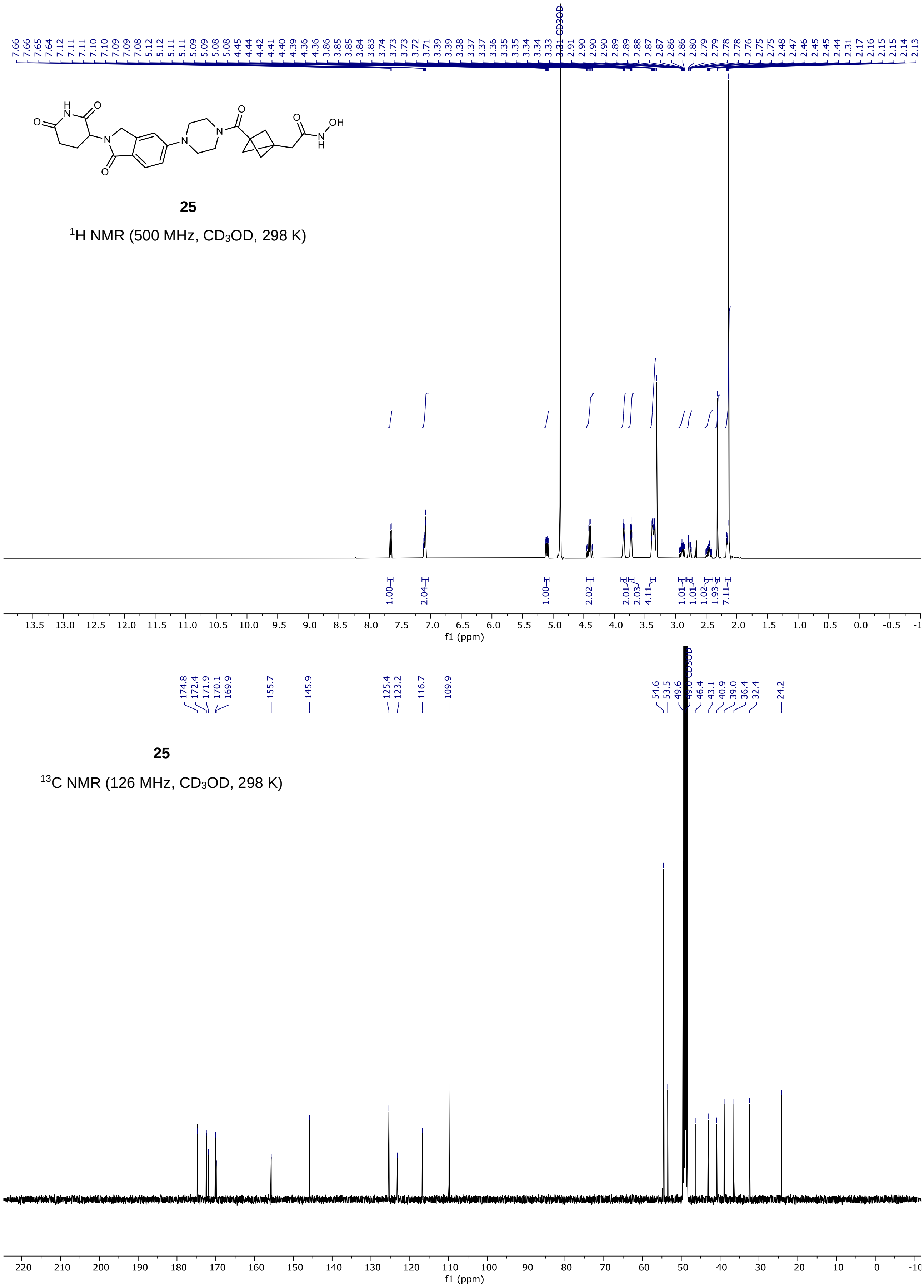

Supplementary Figure 84. ^{1}H NMR (top) and ^{13}C NMR (bottom) spectra of compound **25**. Frequency, temperature and solvent of measurement are indicated on each spectra.

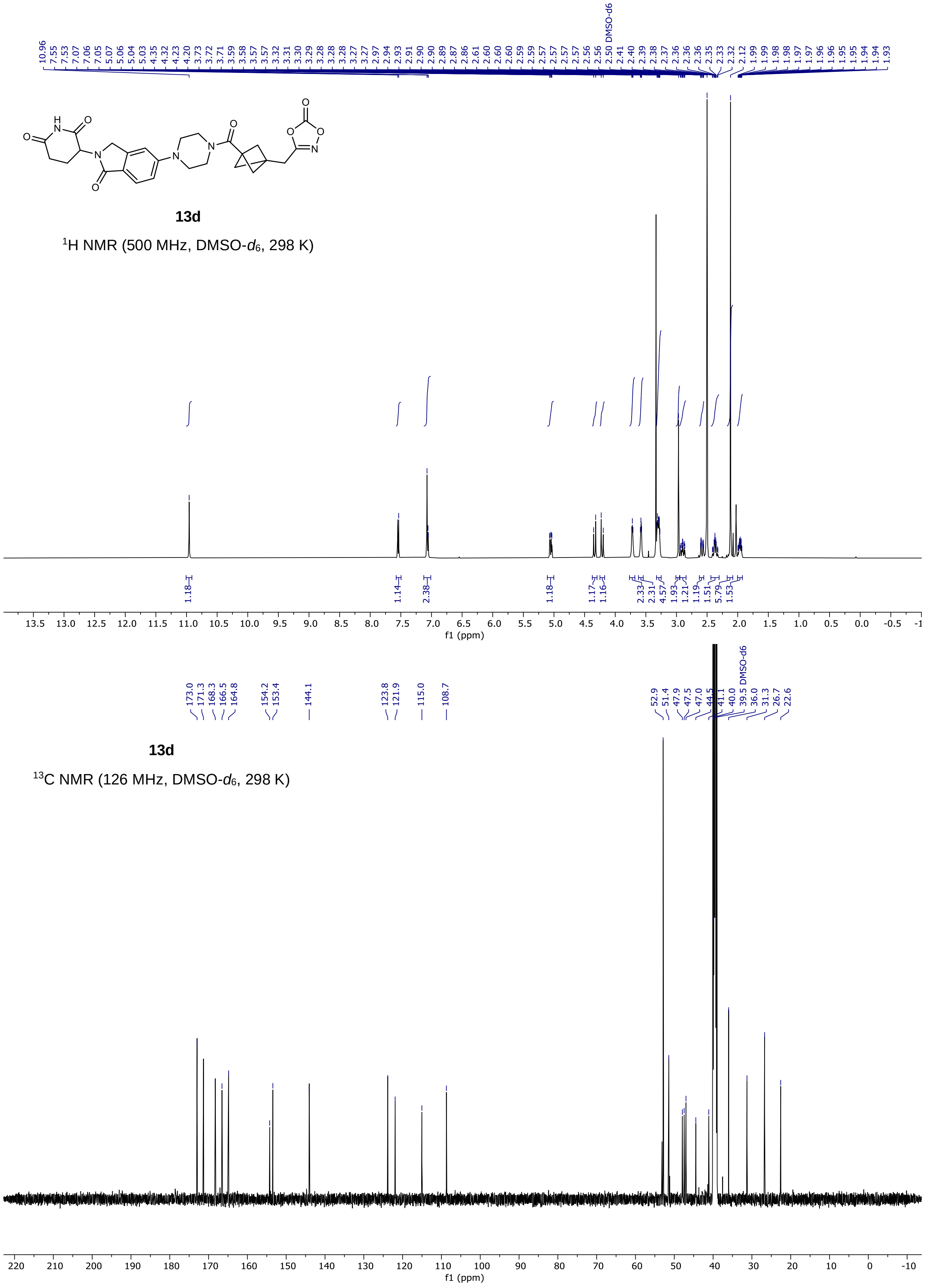

Supplementary Figure 85. ^{1}H NMR (top) and ^{13}C NMR (bottom) spectra of compound **13d**. Frequency, temperature and solvent of measurement are indicated on each spectra.

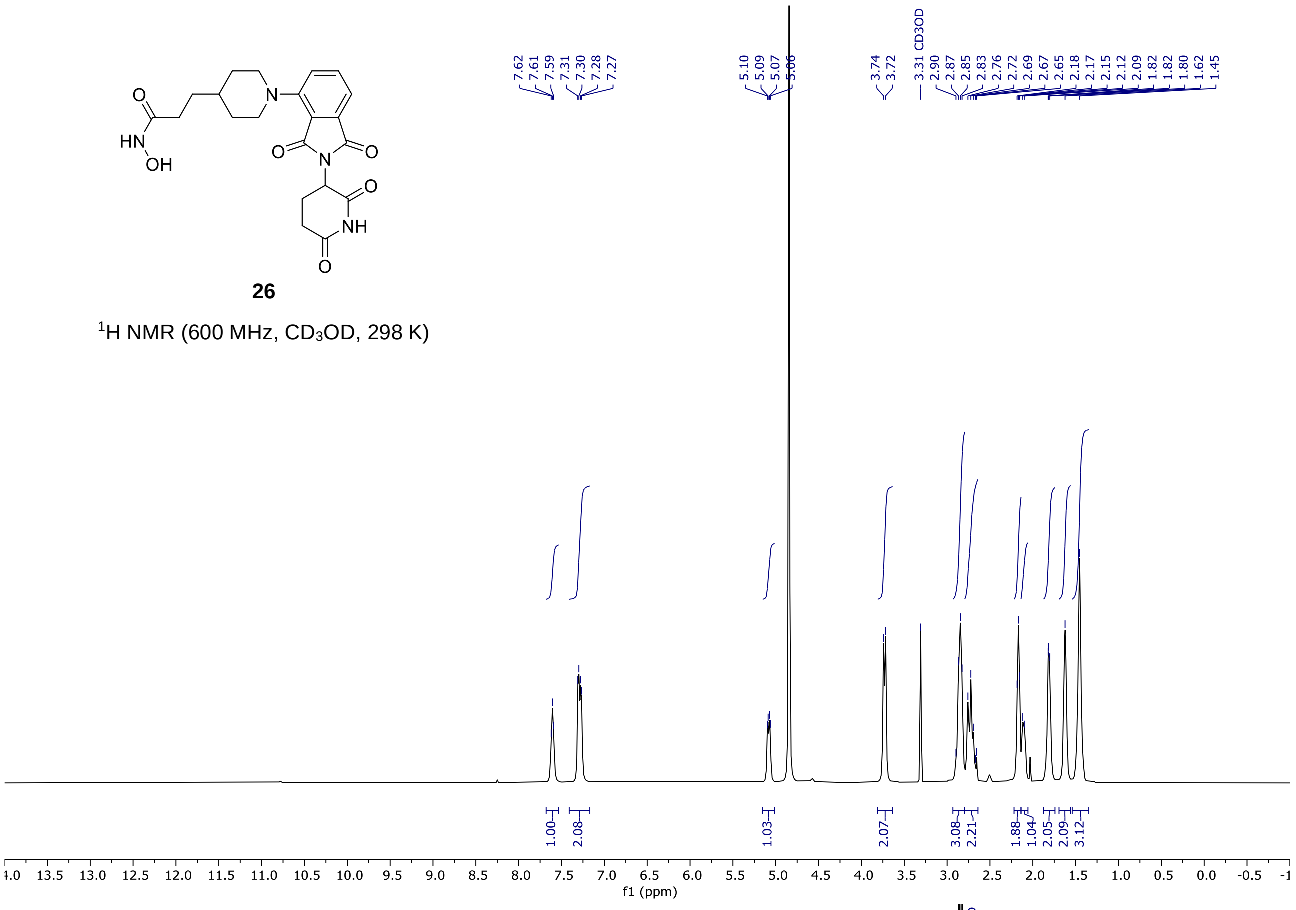

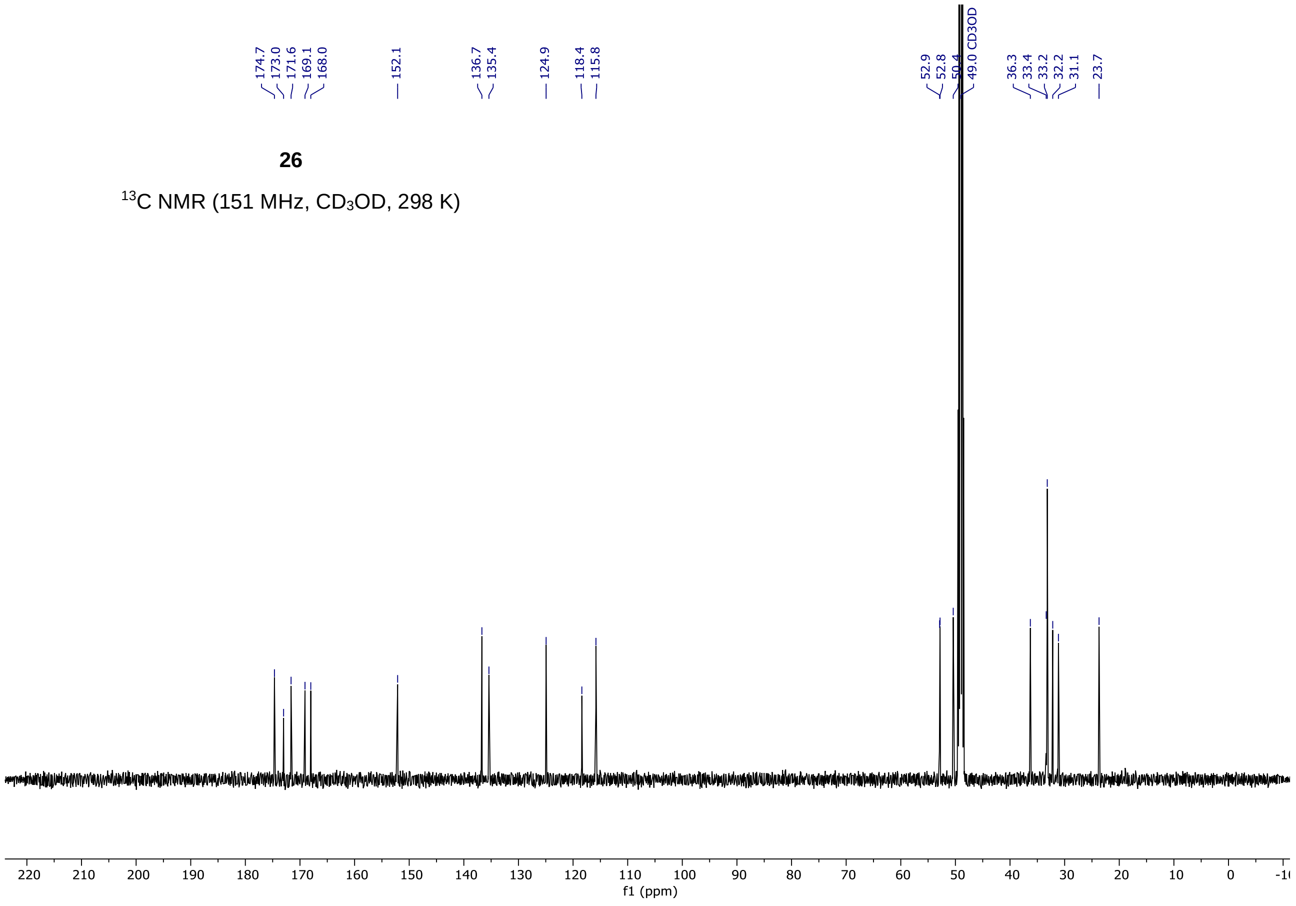

Supplementary Figure 86. ^{1}H NMR (top) and ^{13}C NMR (bottom) spectra of compound **26**. Frequency, temperature and solvent of measurement are indicated on each spectra.

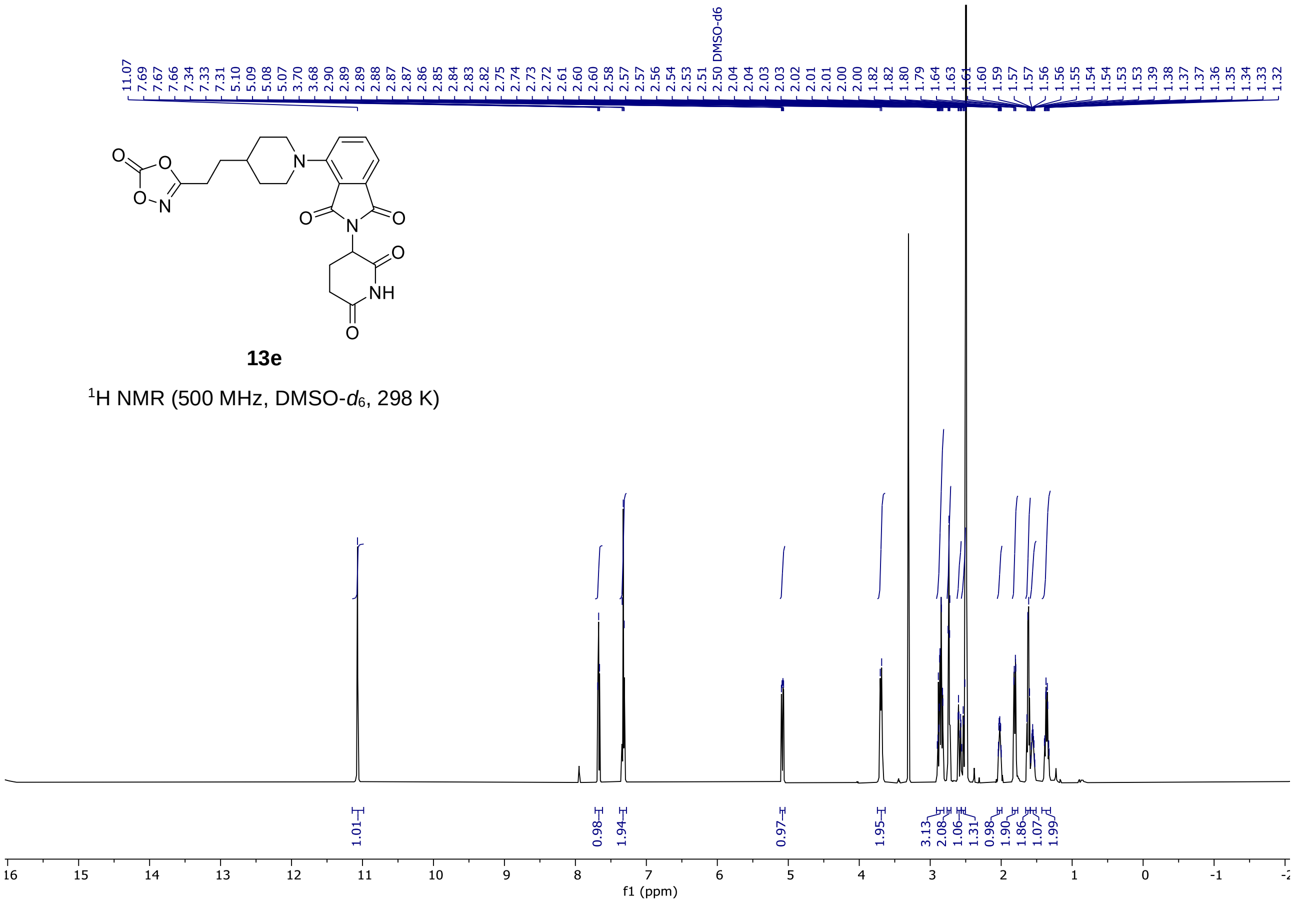

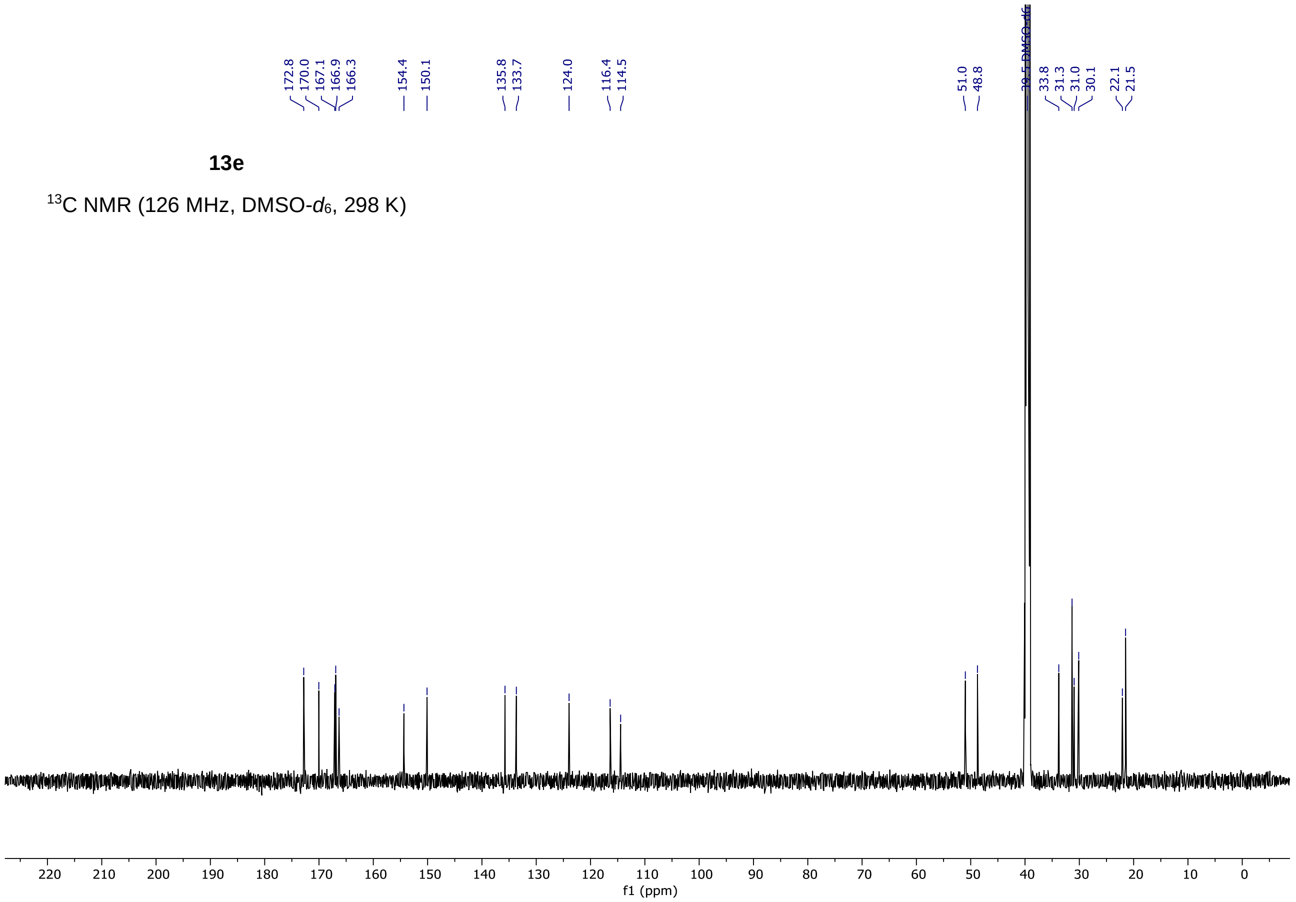

Supplementary Figure 87. ^{1}H NMR (top) and ^{13}C NMR (bottom) spectra of compound **13e**. Frequency, temperature and solvent of measurement are indicated on each spectra.

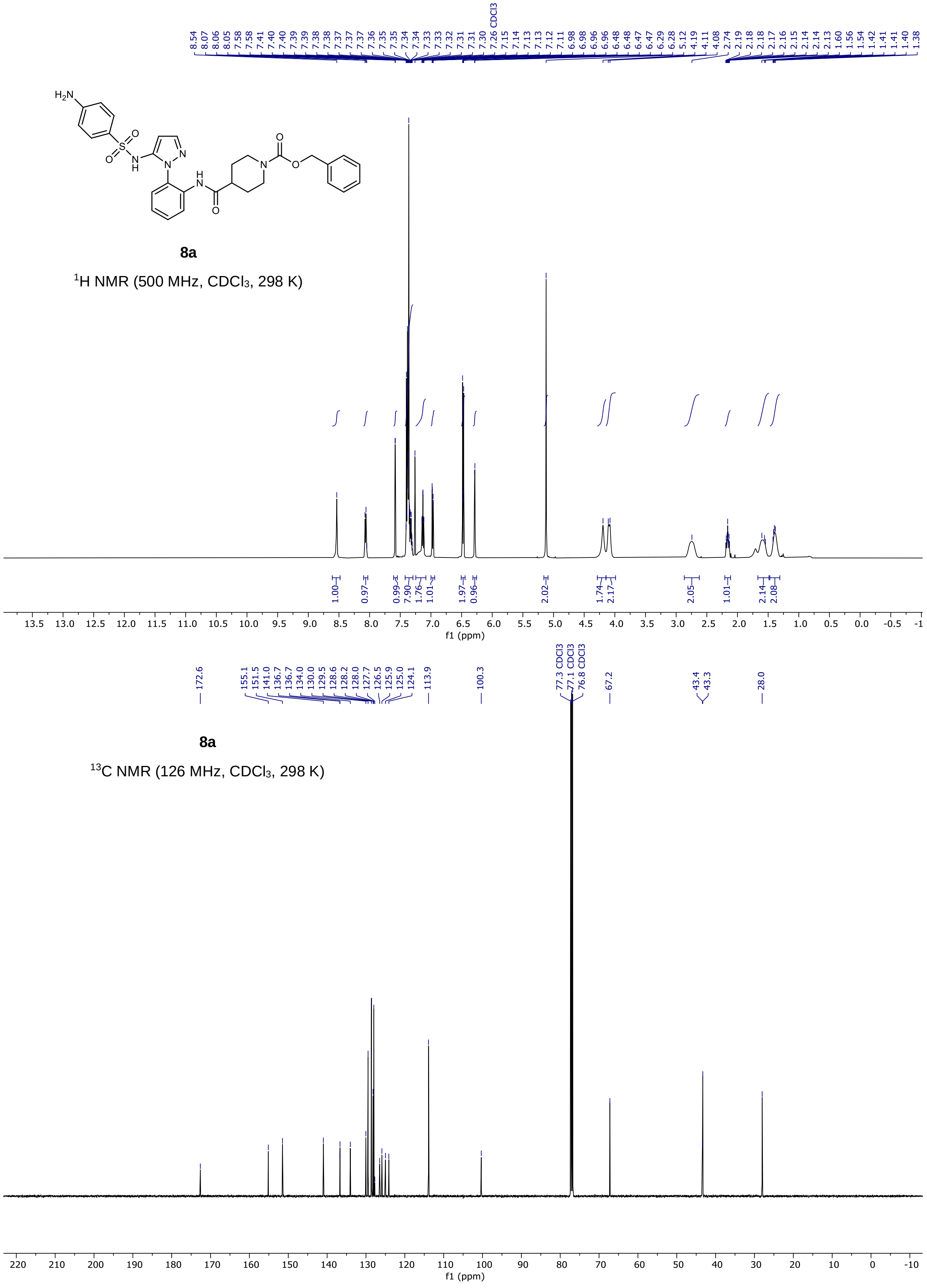

Supplementary Figure 88. ^{1}H NMR (top) and ^{13}C NMR (bottom) spectra of compound **8a**. Frequency, temperature and solvent of measurement are indicated on each spectra.

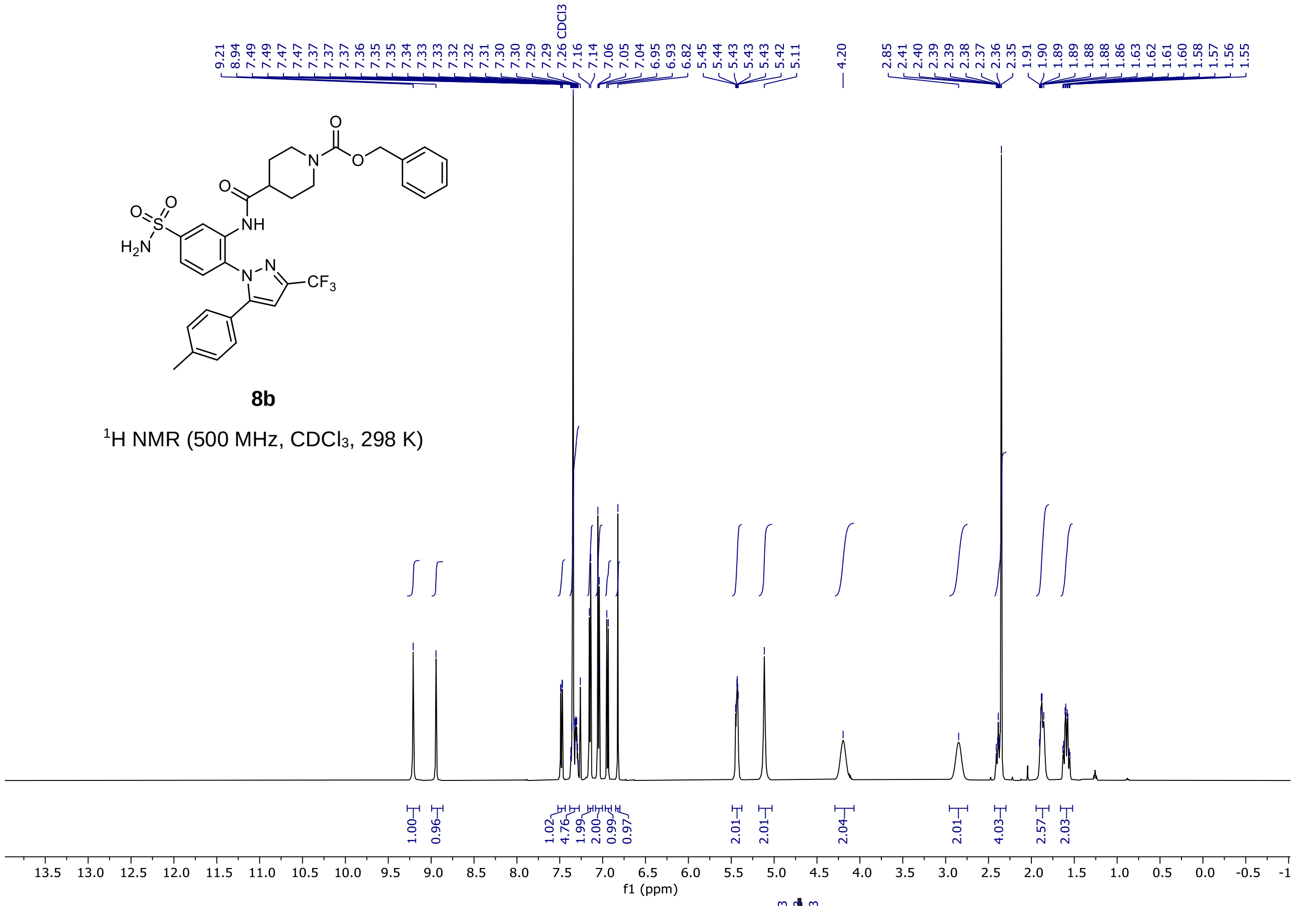

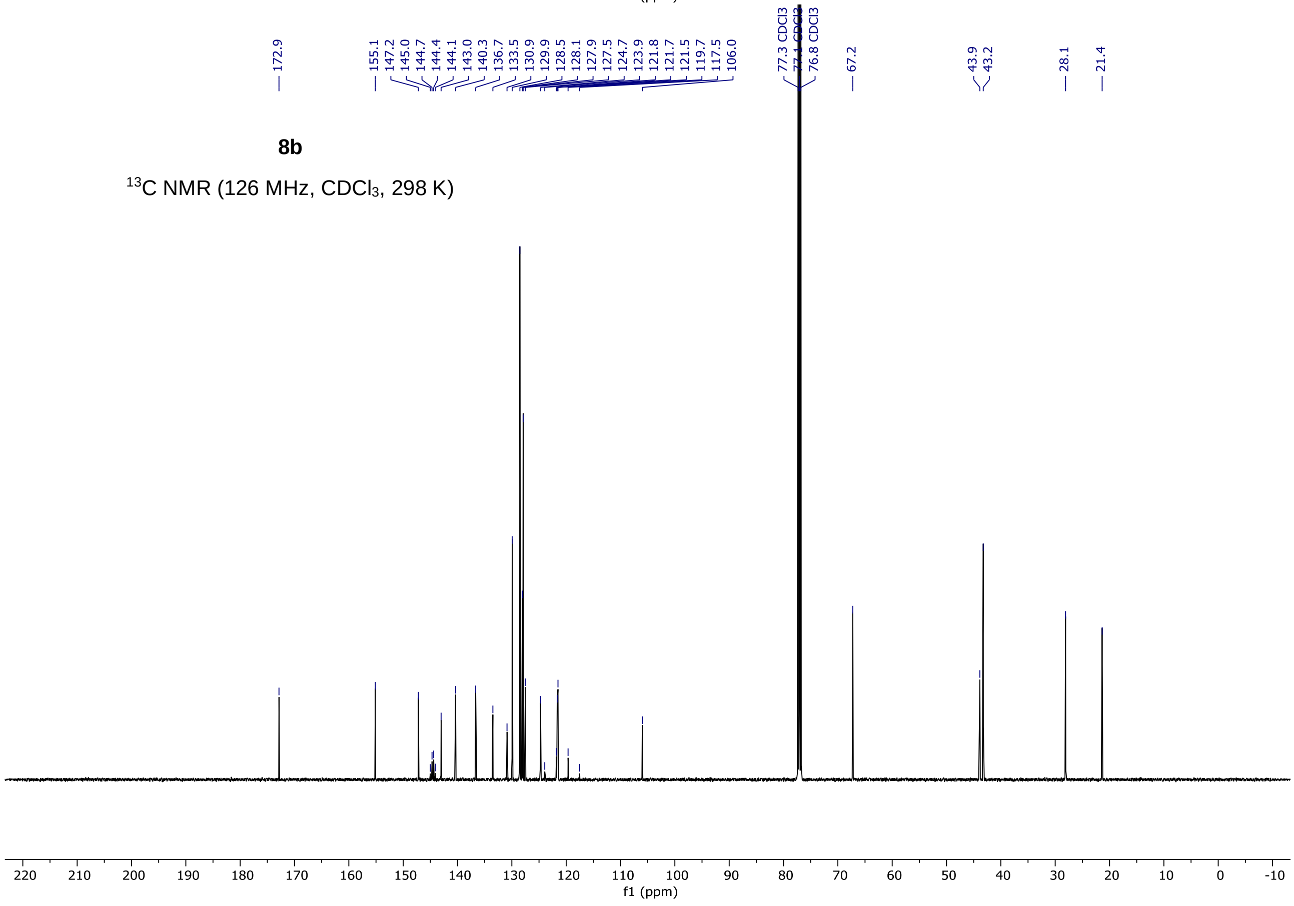

Supplementary Figure 89. ^{1}H NMR (top) and ^{13}C NMR (bottom) spectra of compound **8b**. Frequency, temperature and solvent of measurement are indicated on each spectra.

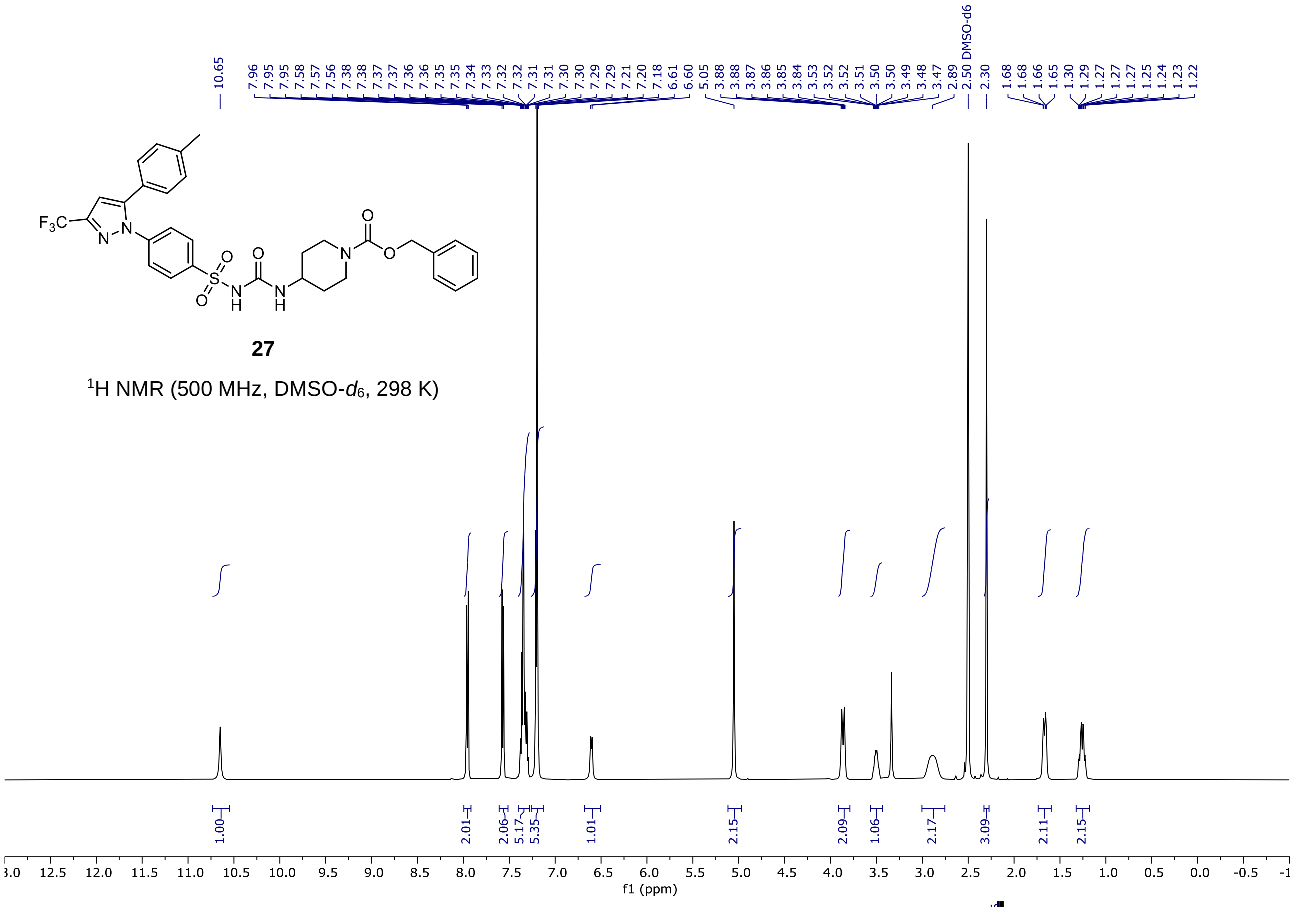

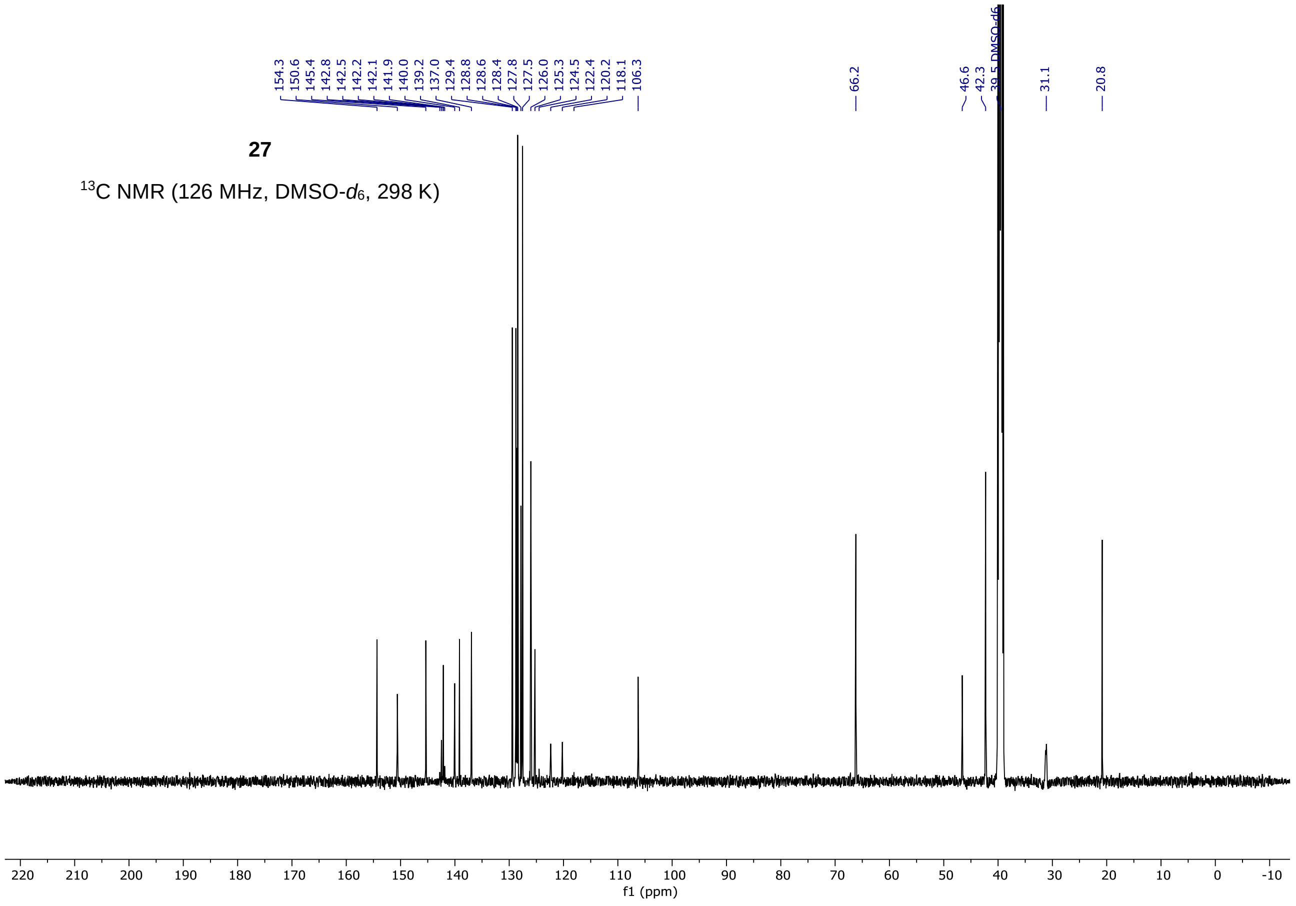

Supplementary Figure 90. ^{1}H NMR (top) and ^{13}C NMR (bottom) spectra of compound **27**. Frequency, temperature and solvent of measurement are indicated on each spectra.

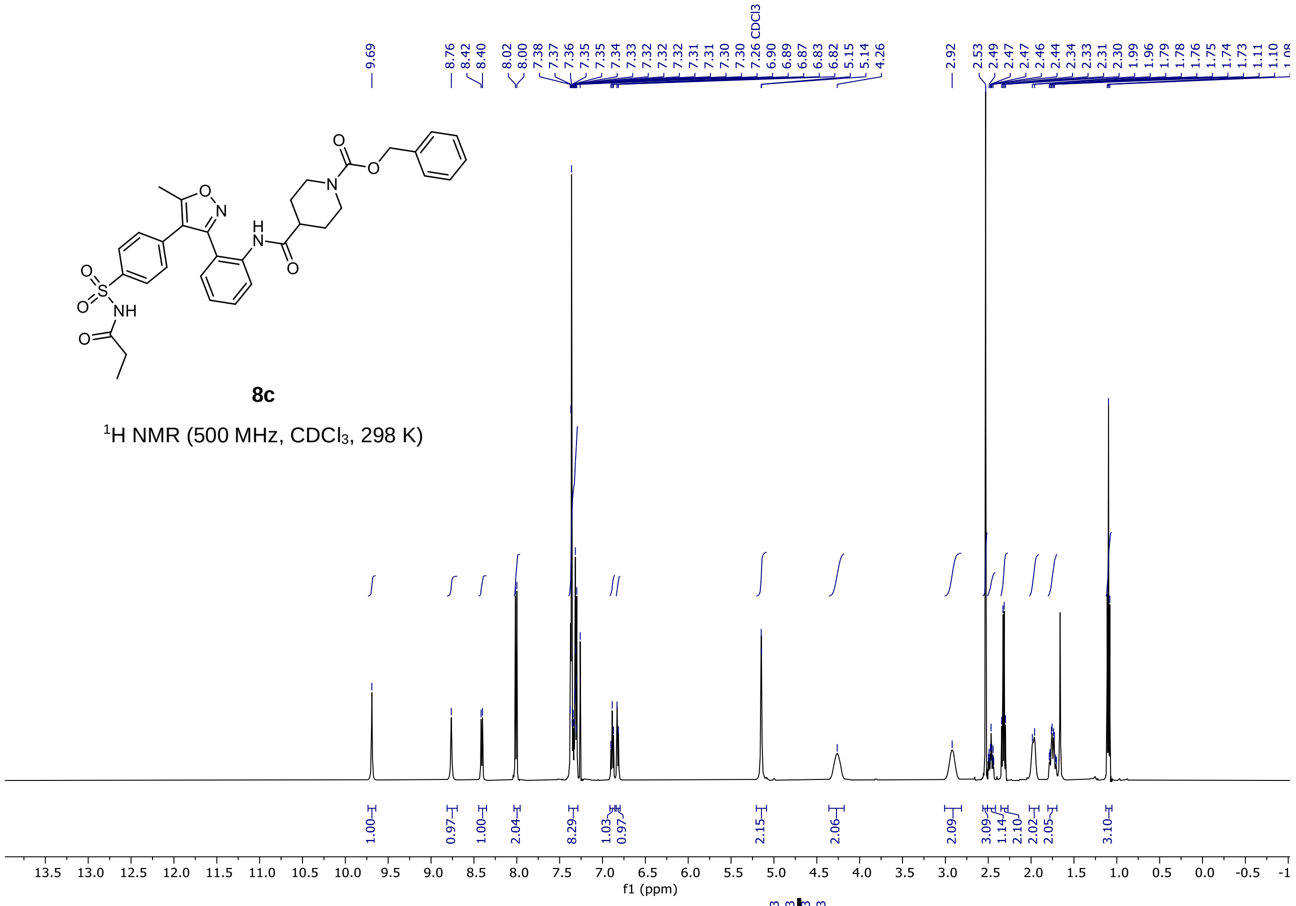

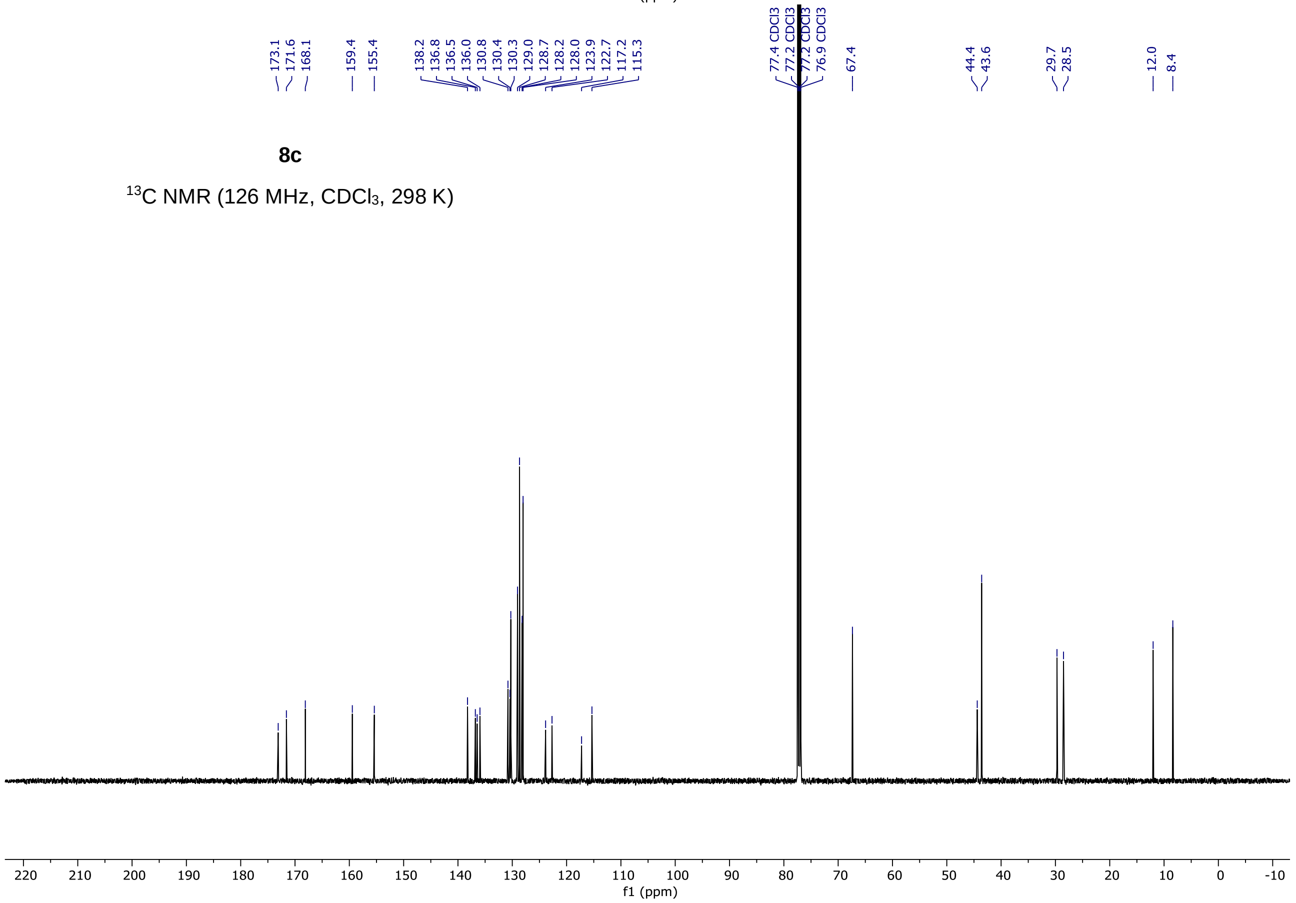

Supplementary Figure 91. ^{1}H NMR (top) and ^{13}C NMR (bottom) spectra of compound **8c**. Frequency, temperature and solvent of measurement are indicated on each spectra.

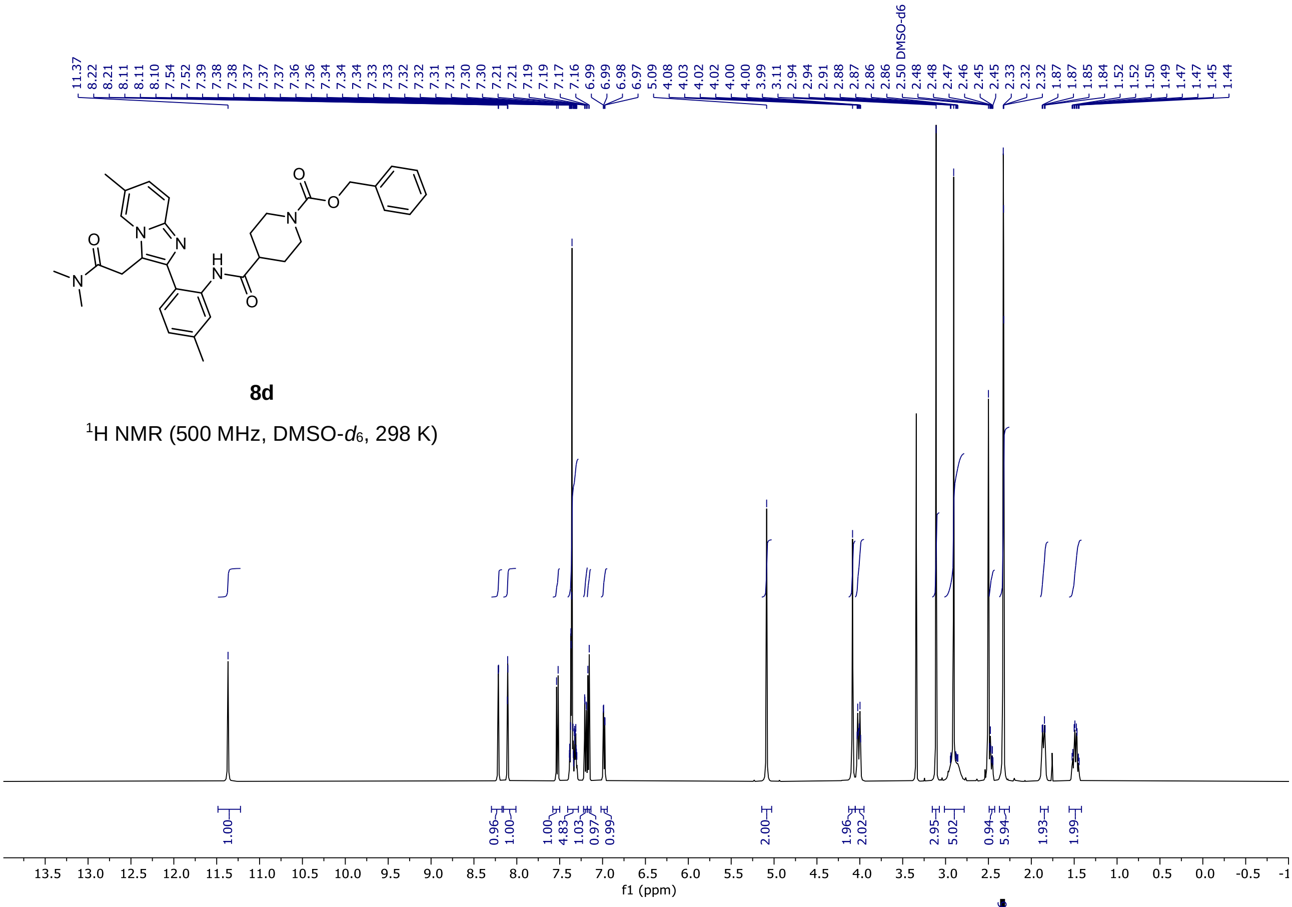

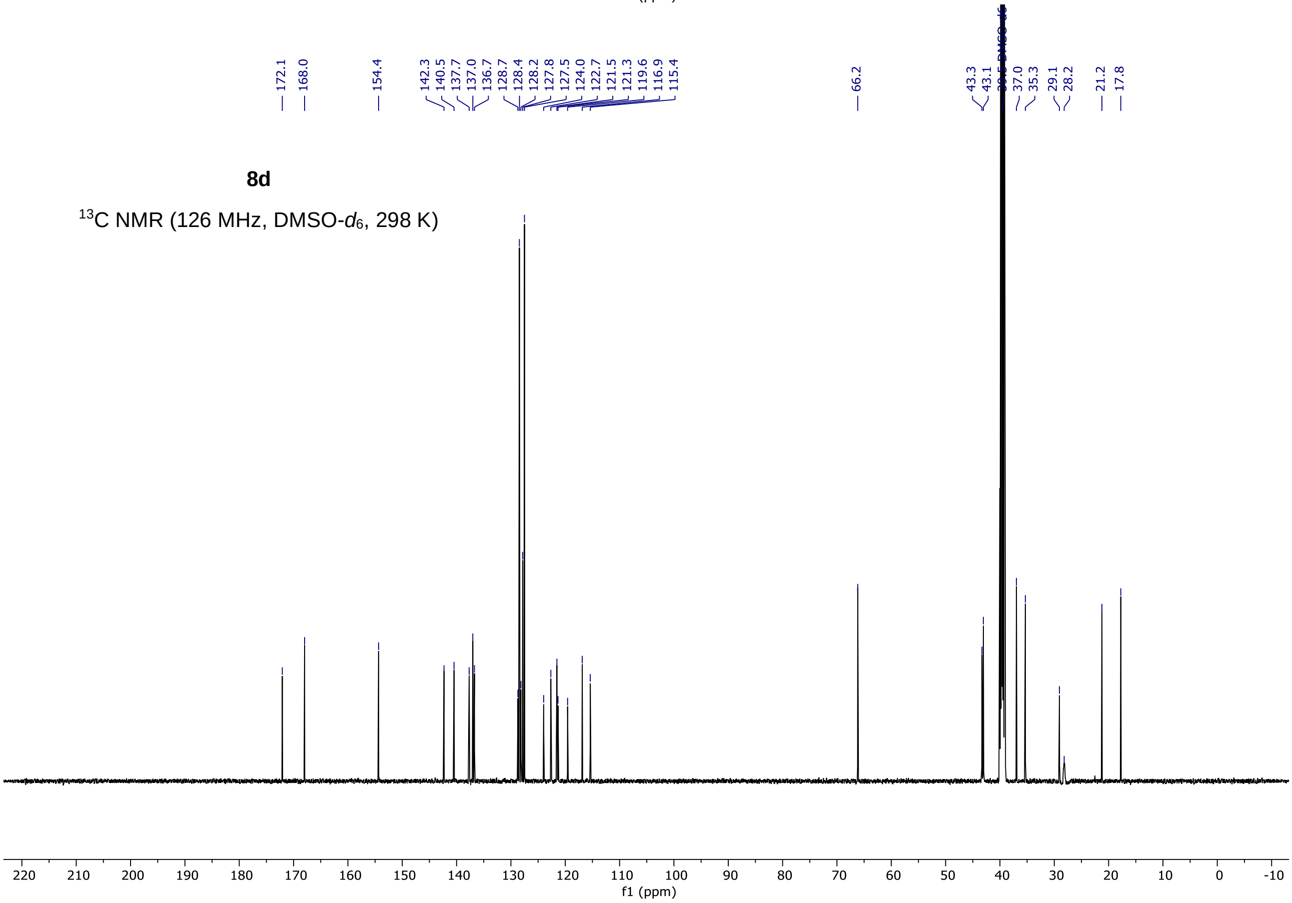

Supplementary Figure 92. ^{1}H NMR (top) and ^{13}C NMR (bottom) spectra of compound **8d**. Frequency, temperature and solvent of measurement are indicated on each spectra.

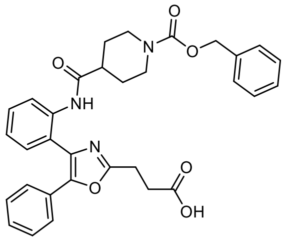

8e

^{1}H NMR (500 MHz, $CDCl_3$, 298 K)

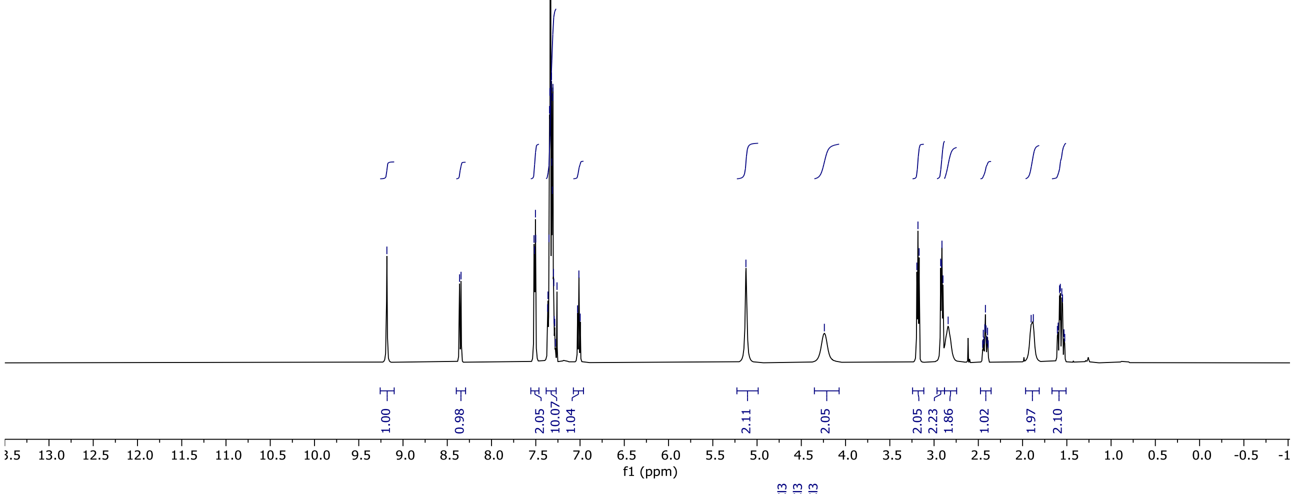

8e

^{13}C NMR (126 MHz, $CDCl_3$, 298 K)

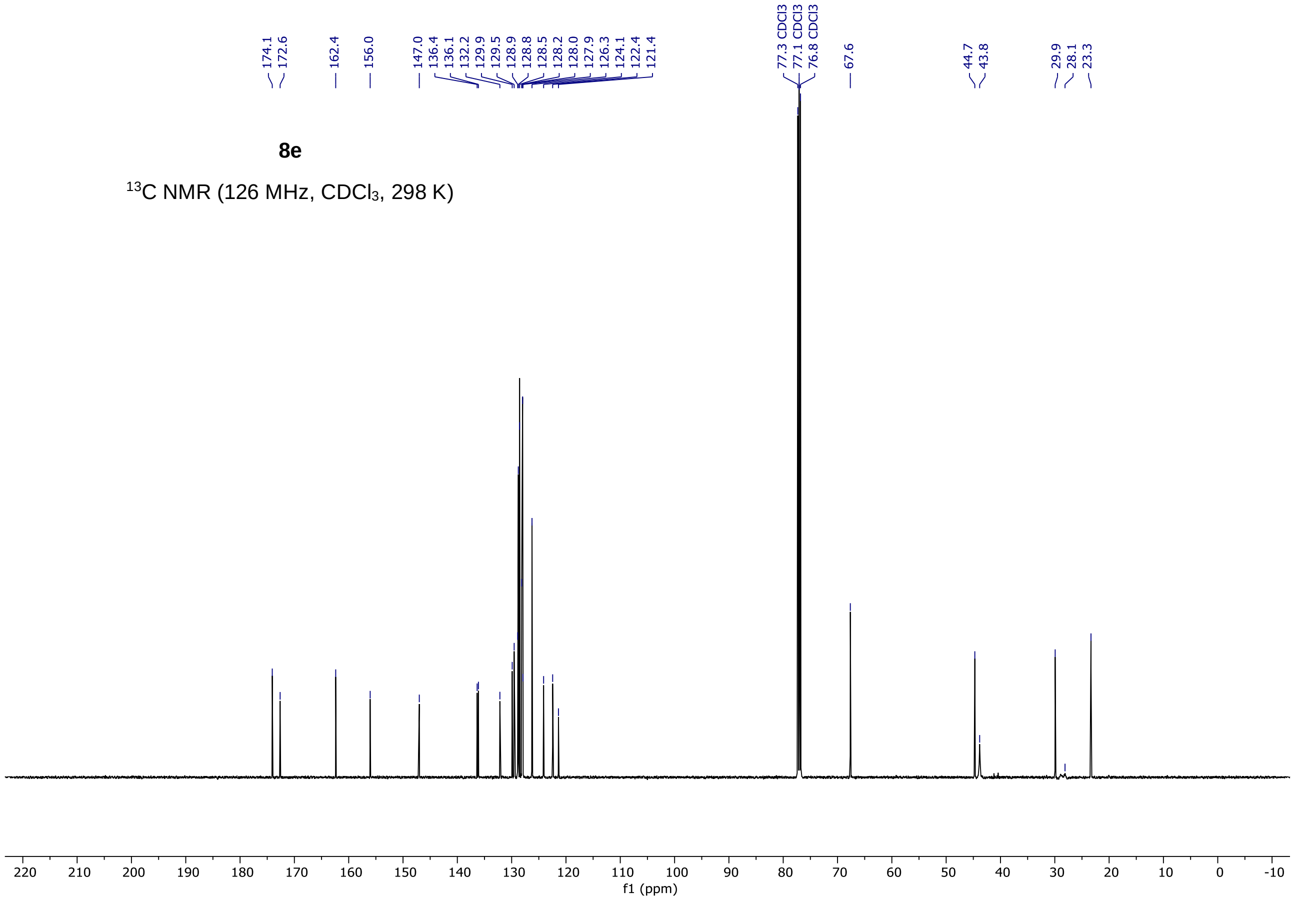

Supplementary Figure 93. ^{1}H NMR (top) and ^{13}C NMR (bottom) spectra of compound **8e**. Frequency, temperature and solvent of measurement are indicated on each spectra.

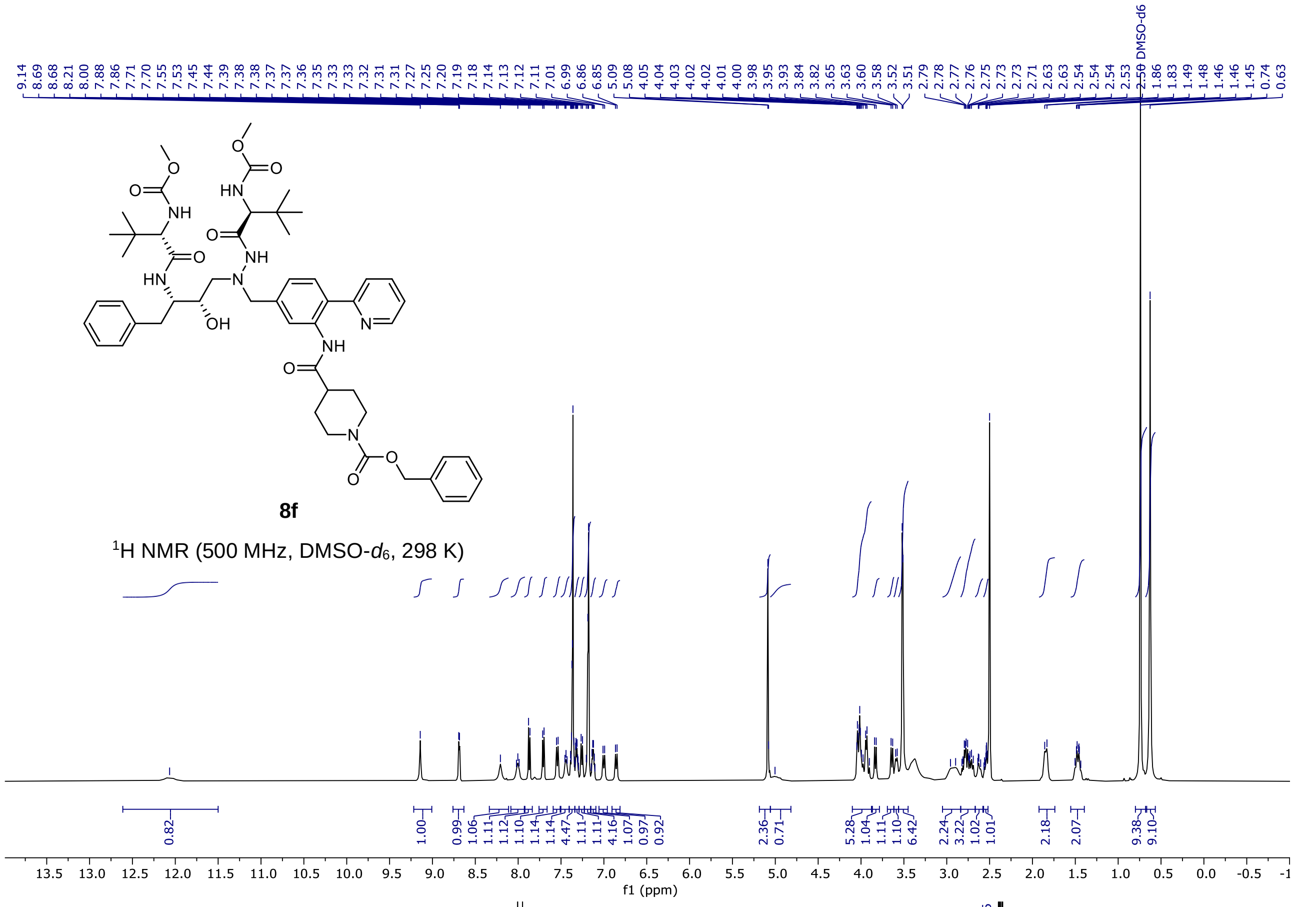

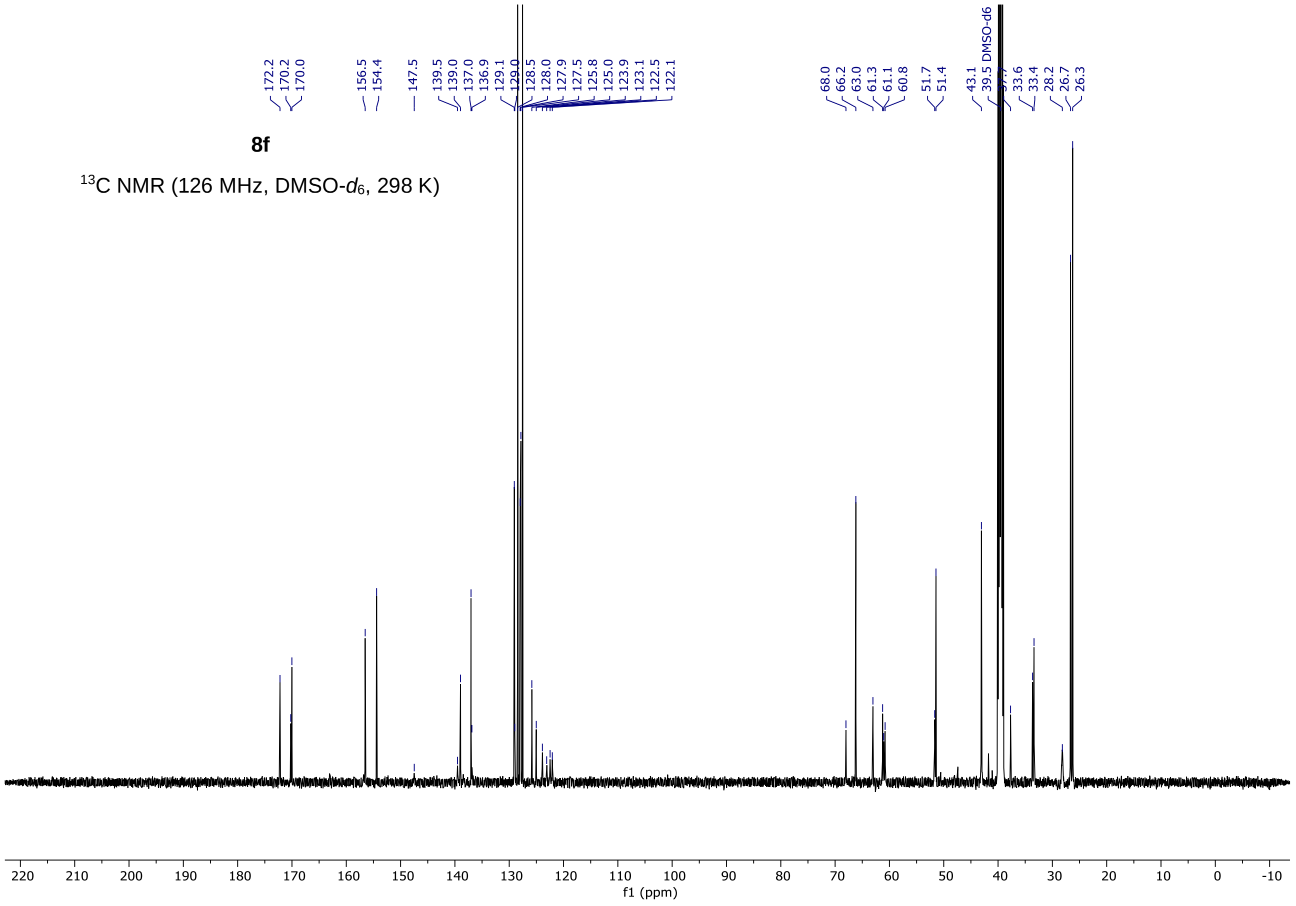

Supplementary Figure 94. ^{1}H NMR (top) and ^{13}C NMR (bottom) spectra of compound **8f**. Frequency, temperature and solvent of measurement are indicated on each spectra.

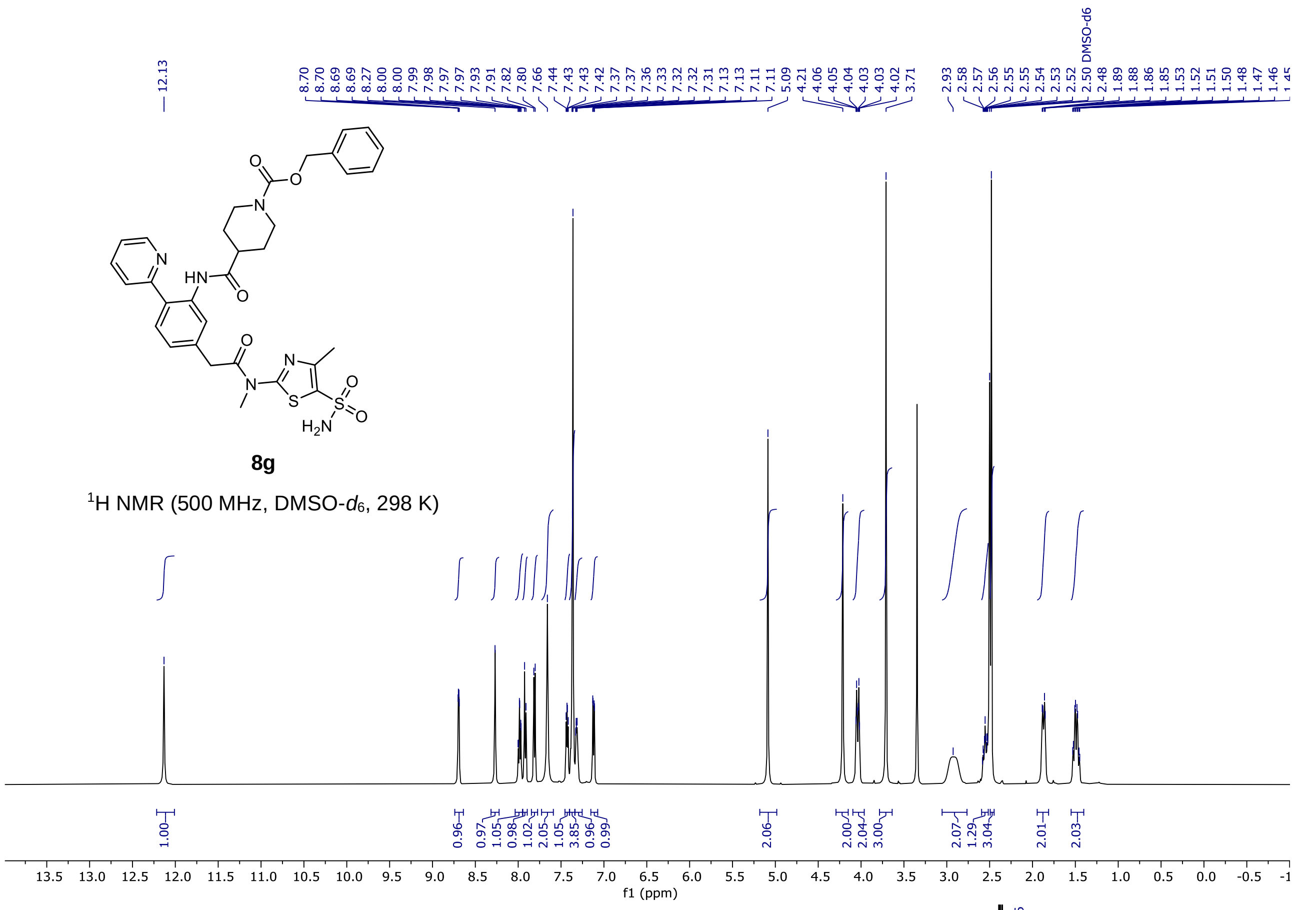

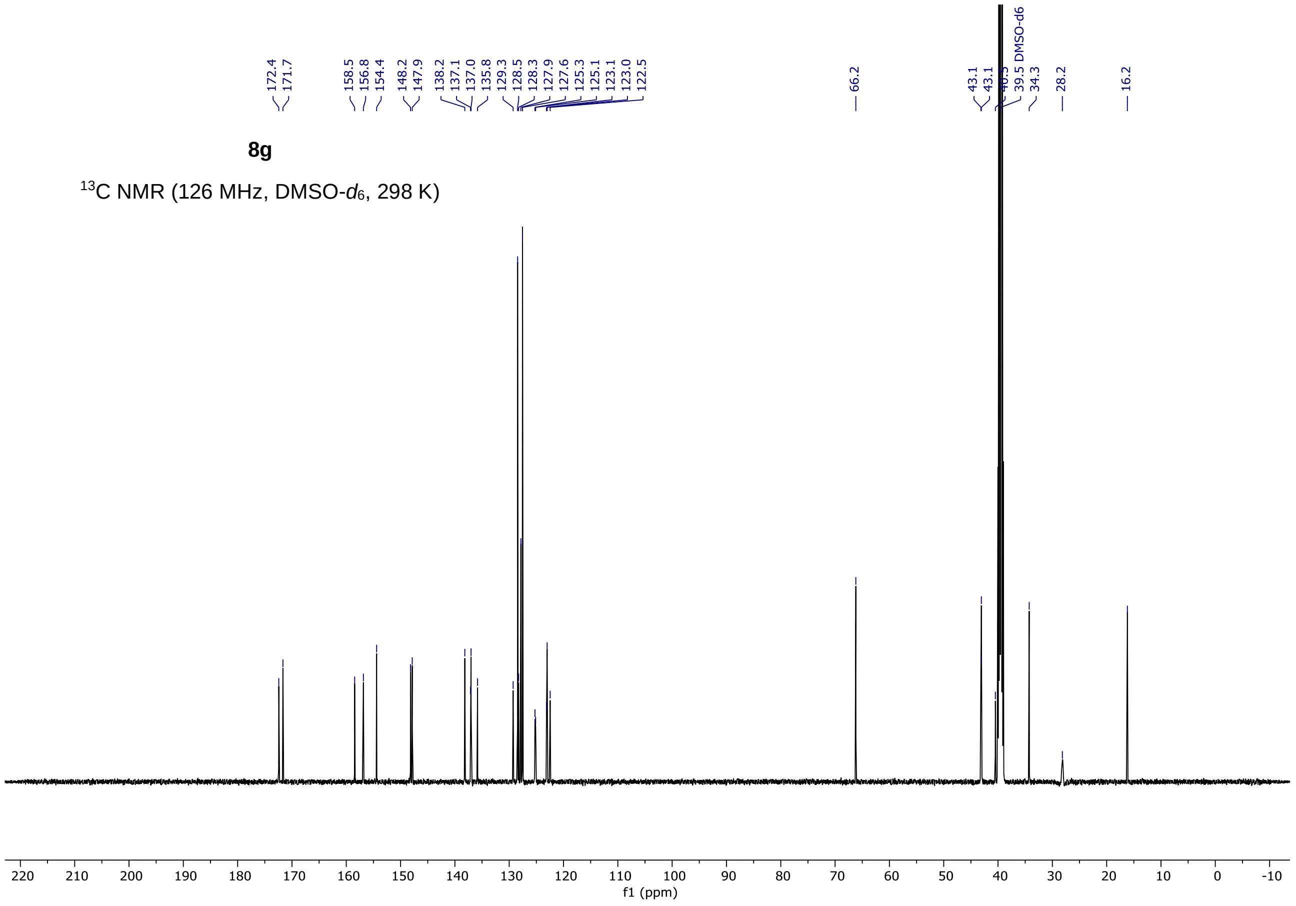

Supplementary Figure 95. ^{1}H NMR (top) and ^{13}C NMR (bottom) spectra of compound **8g**. Frequency, temperature and solvent of measurement are indicated on each spectra.

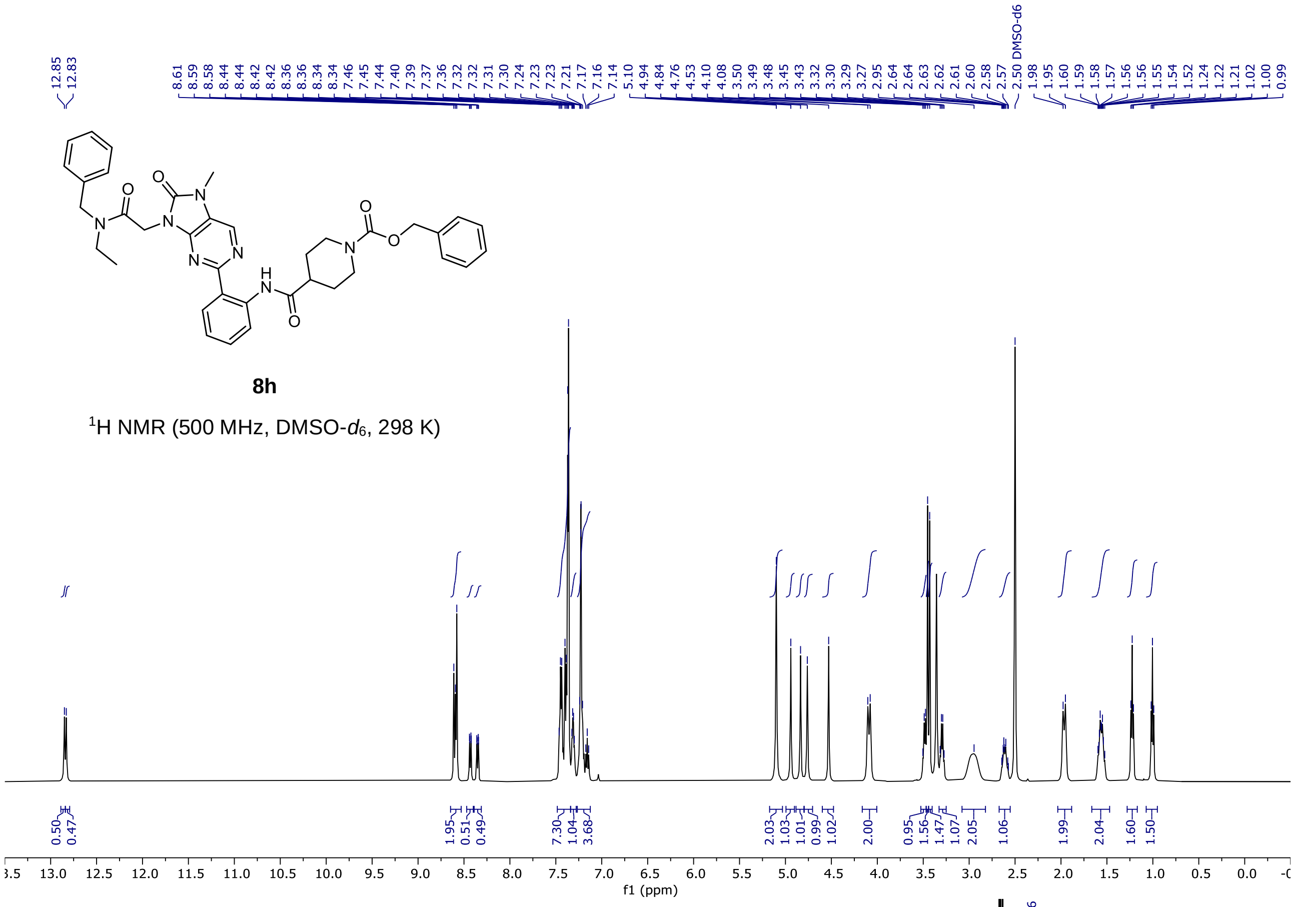

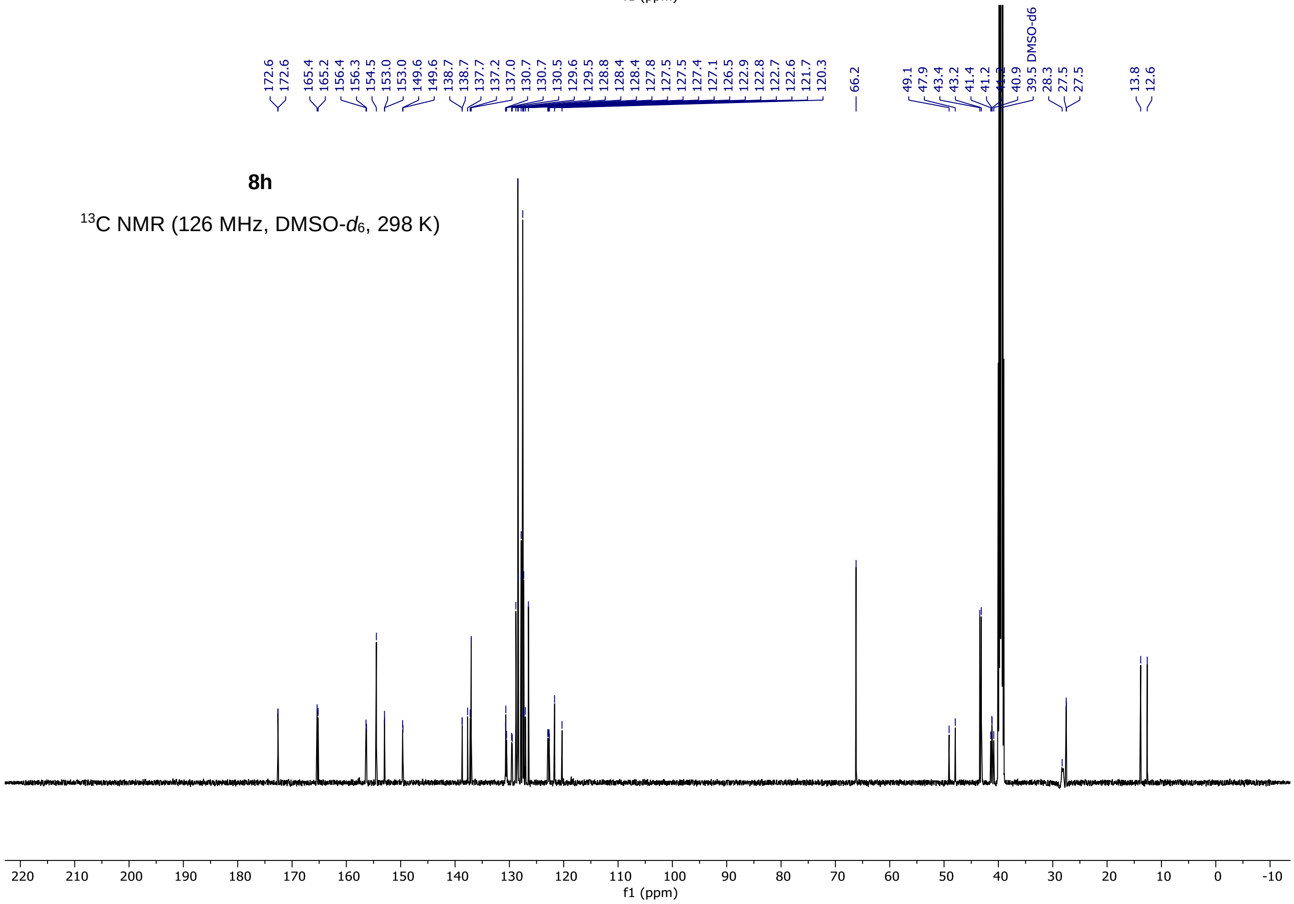

Supplementary Figure 96. ^{1}H NMR (top) and ^{13}C NMR (bottom) spectra of compound **8h**. Frequency, temperature and solvent of measurement are indicated on each spectra.

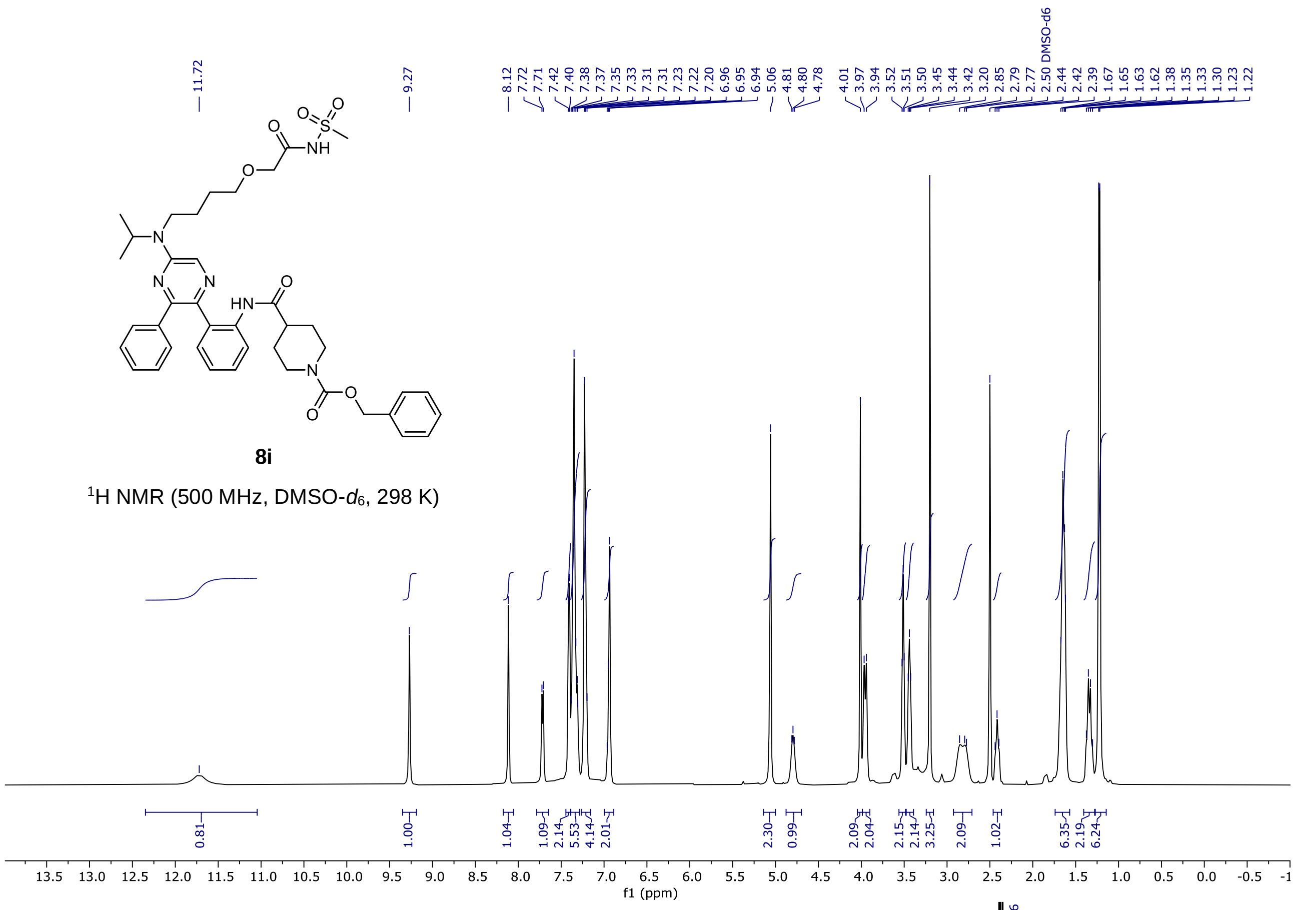

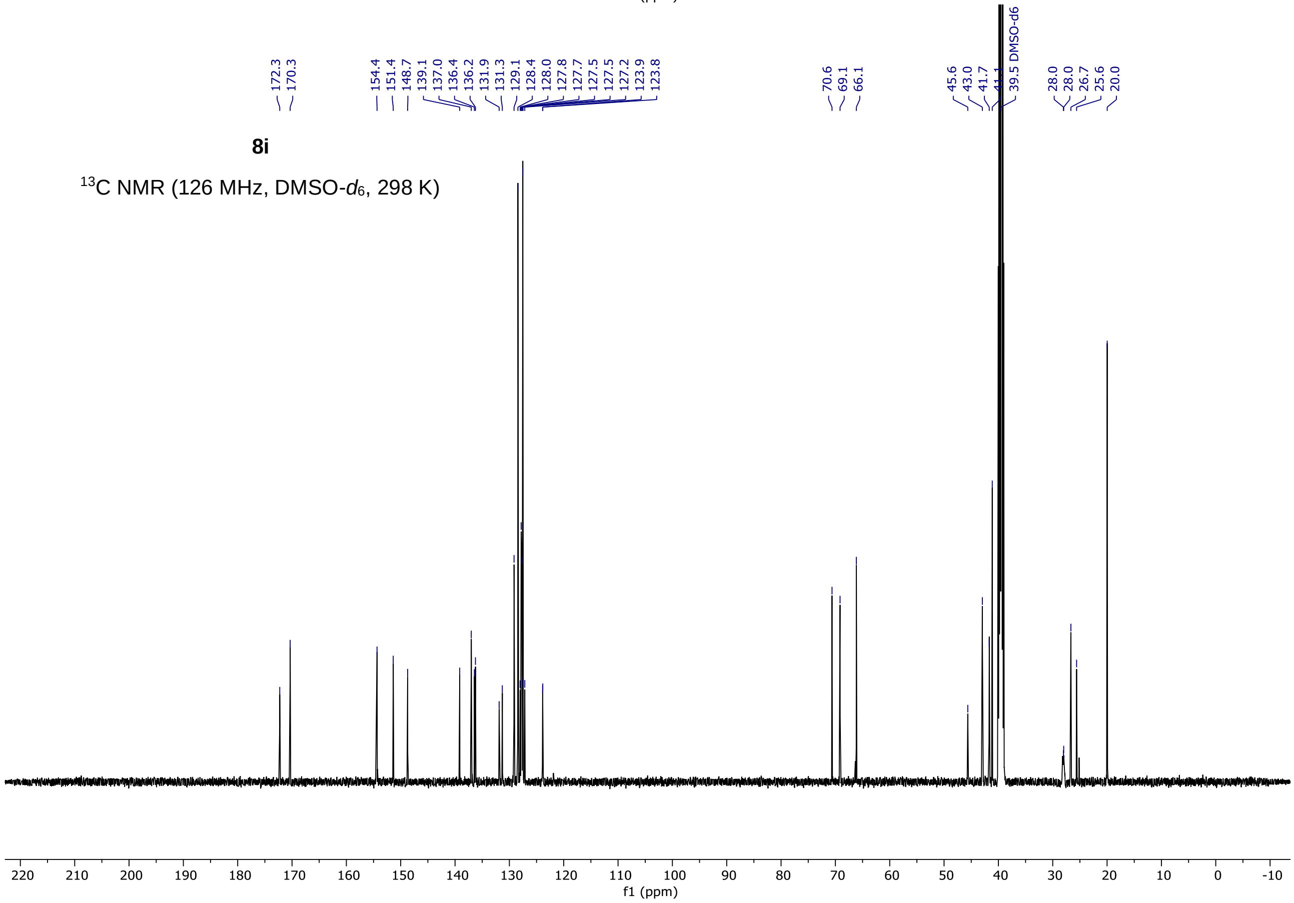

Supplementary Figure 97. ^{1}H NMR (top) and ^{13}C NMR (bottom) spectra of compound **8i**. Frequency, temperature and solvent of measurement are indicated on each spectra.

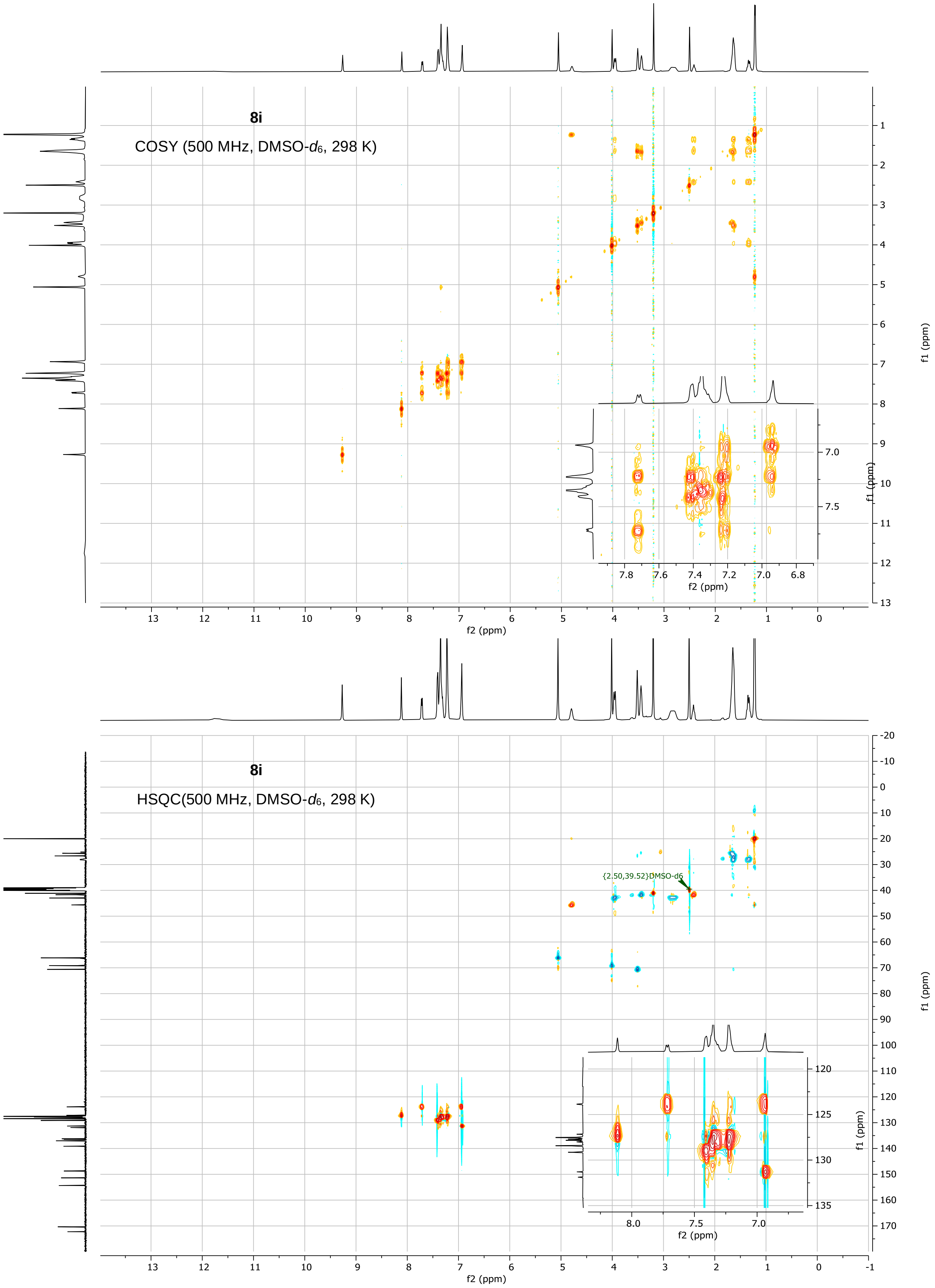

Supplementary Figure 98. COSY (top) and HSQC NMR (bottom) spectra of compound **8i**. Frequency, temperature and solvent of measurement are indicated on each spectra.

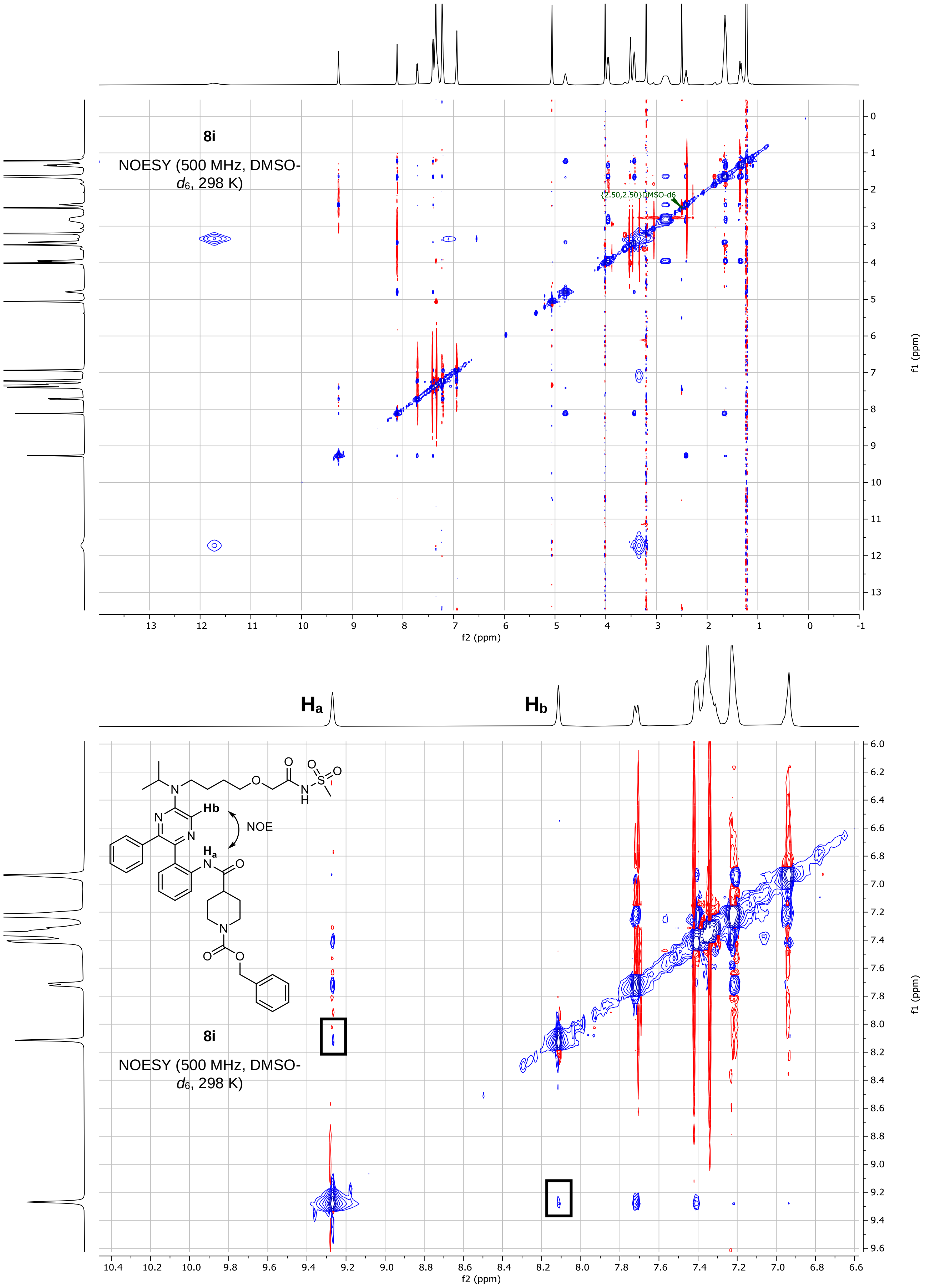

Supplementary Figure 99. NOESY spectrum of compound **8i**. Frequency, temperature and solvent of measurement are indicated on the spectrum.

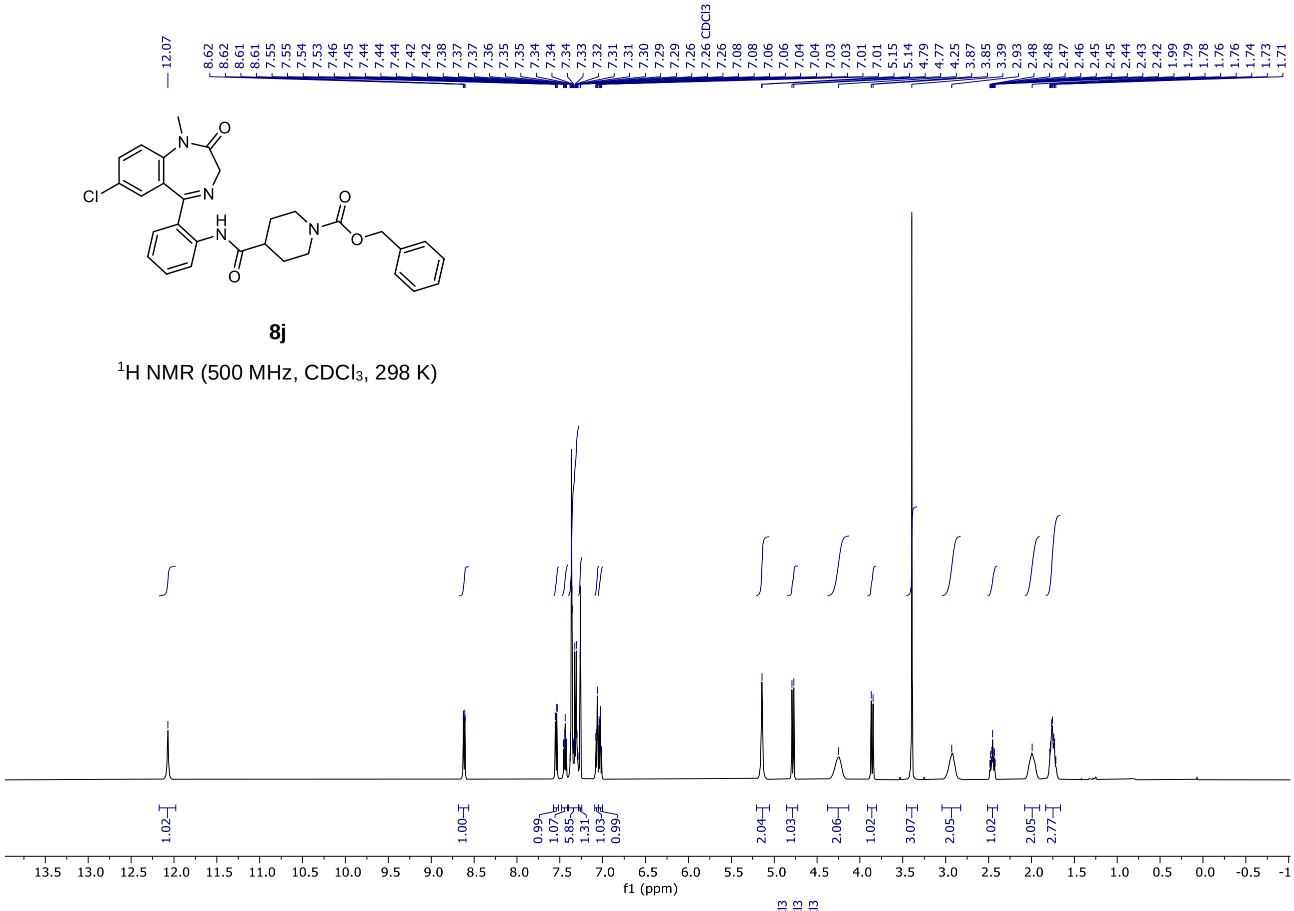

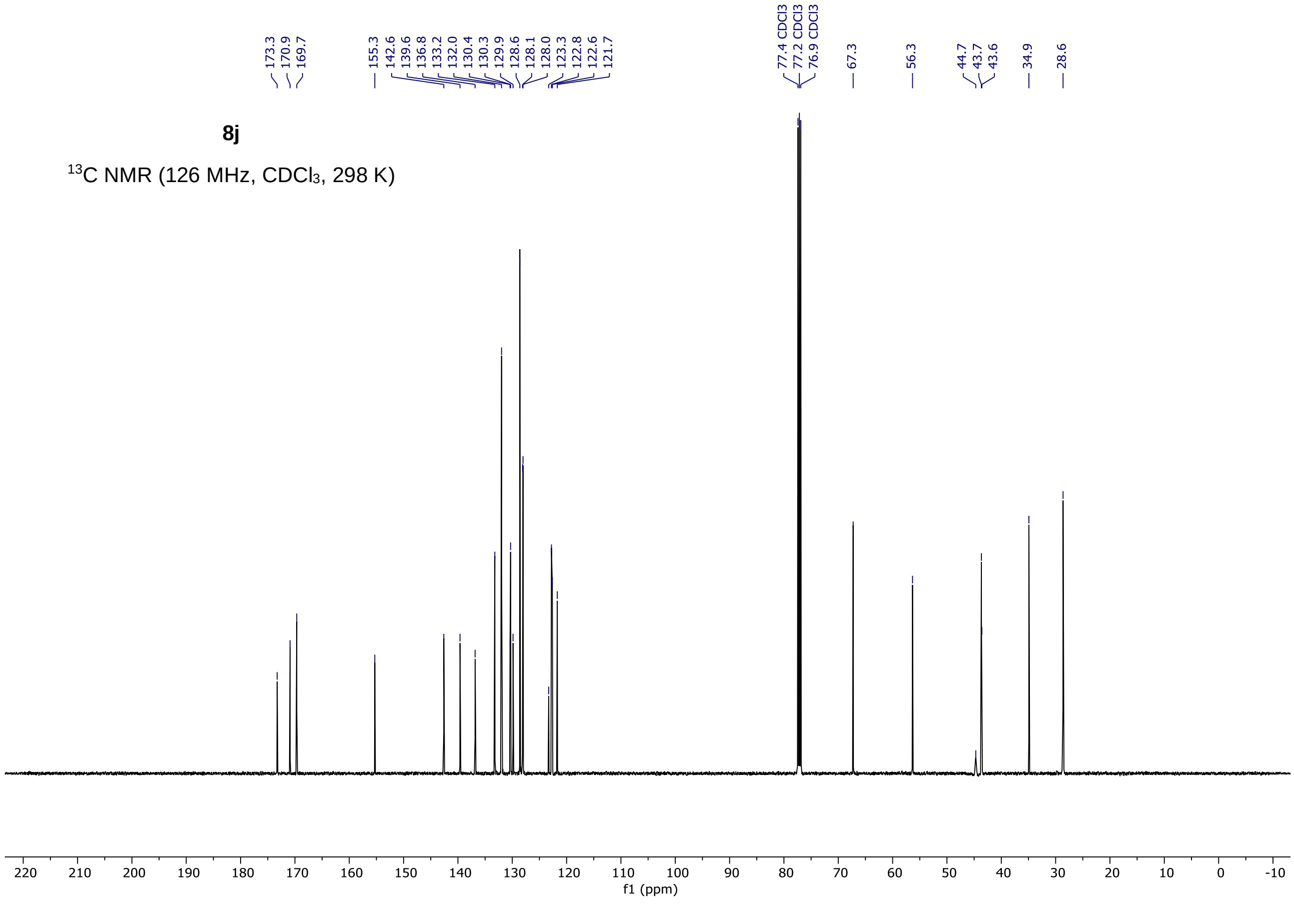

Supplementary Figure 100. ^{1}H NMR (top) and ^{13}C NMR (bottom) spectra of compound **8j**. Frequency, temperature and solvent of measurement are indicated on each spectra.

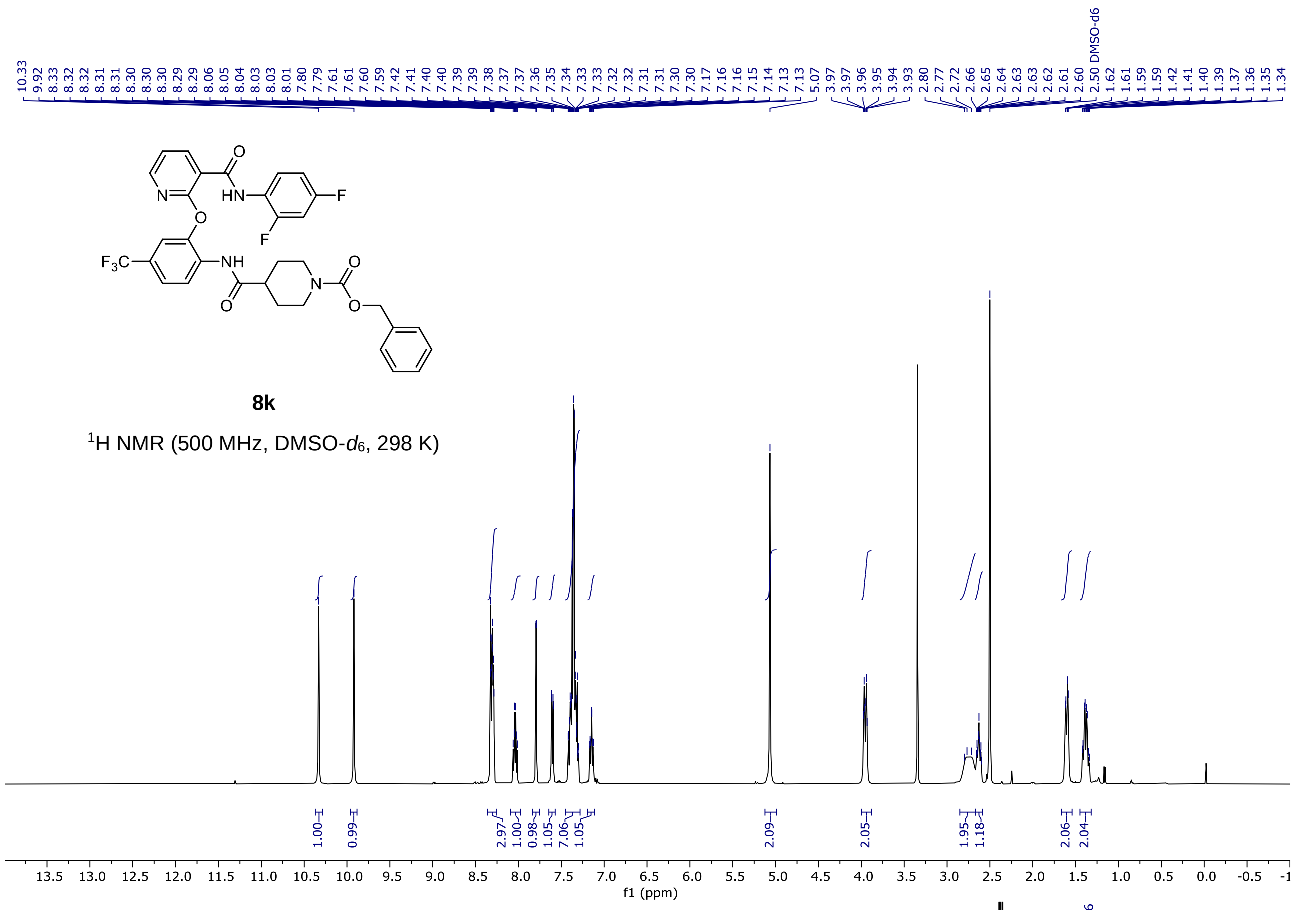

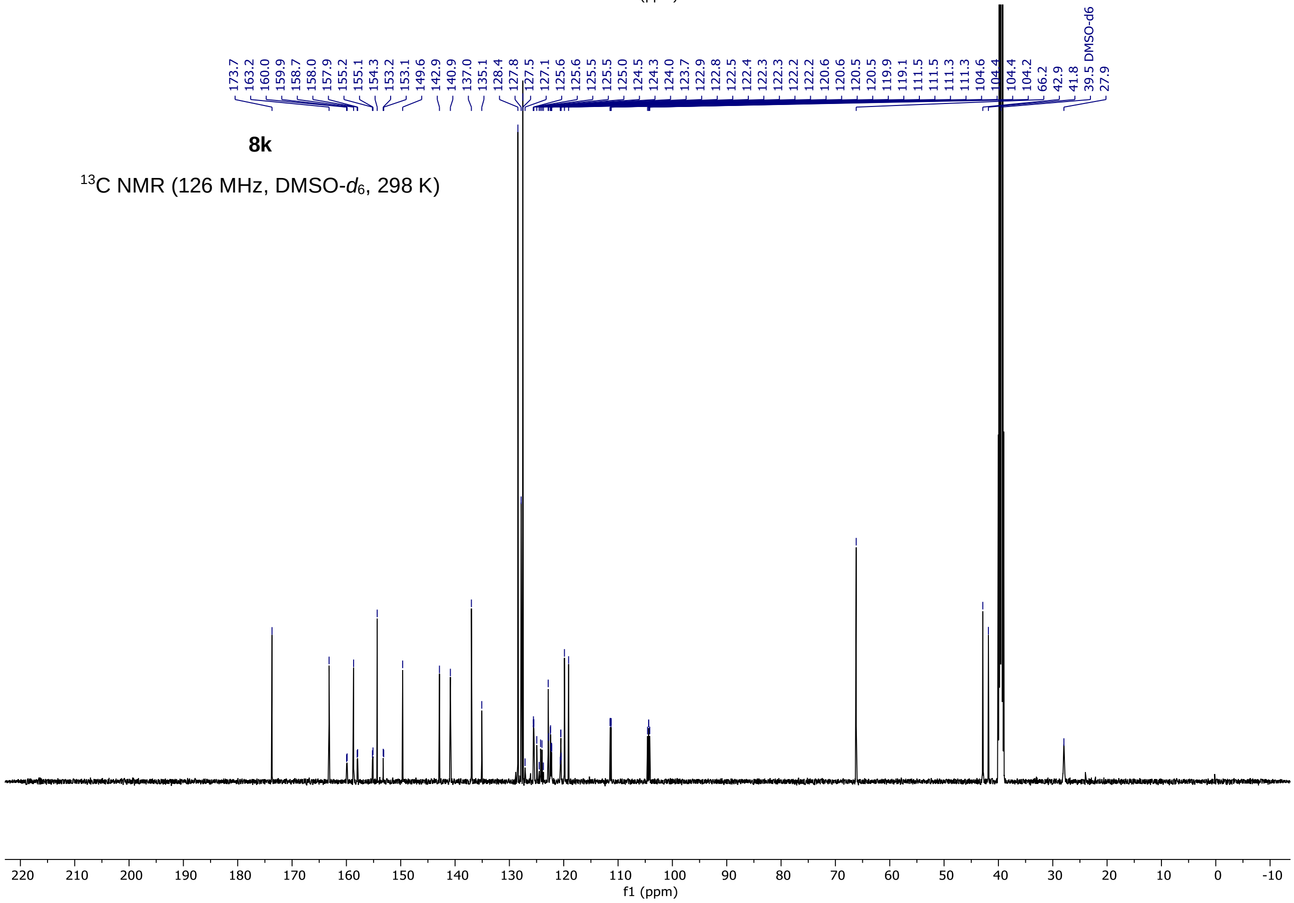

Supplementary Figure 101. ^{1}H NMR (top) and ^{13}C NMR (bottom) spectra of compound **8k**. Frequency, temperature and solvent of measurement are indicated on each spectra.

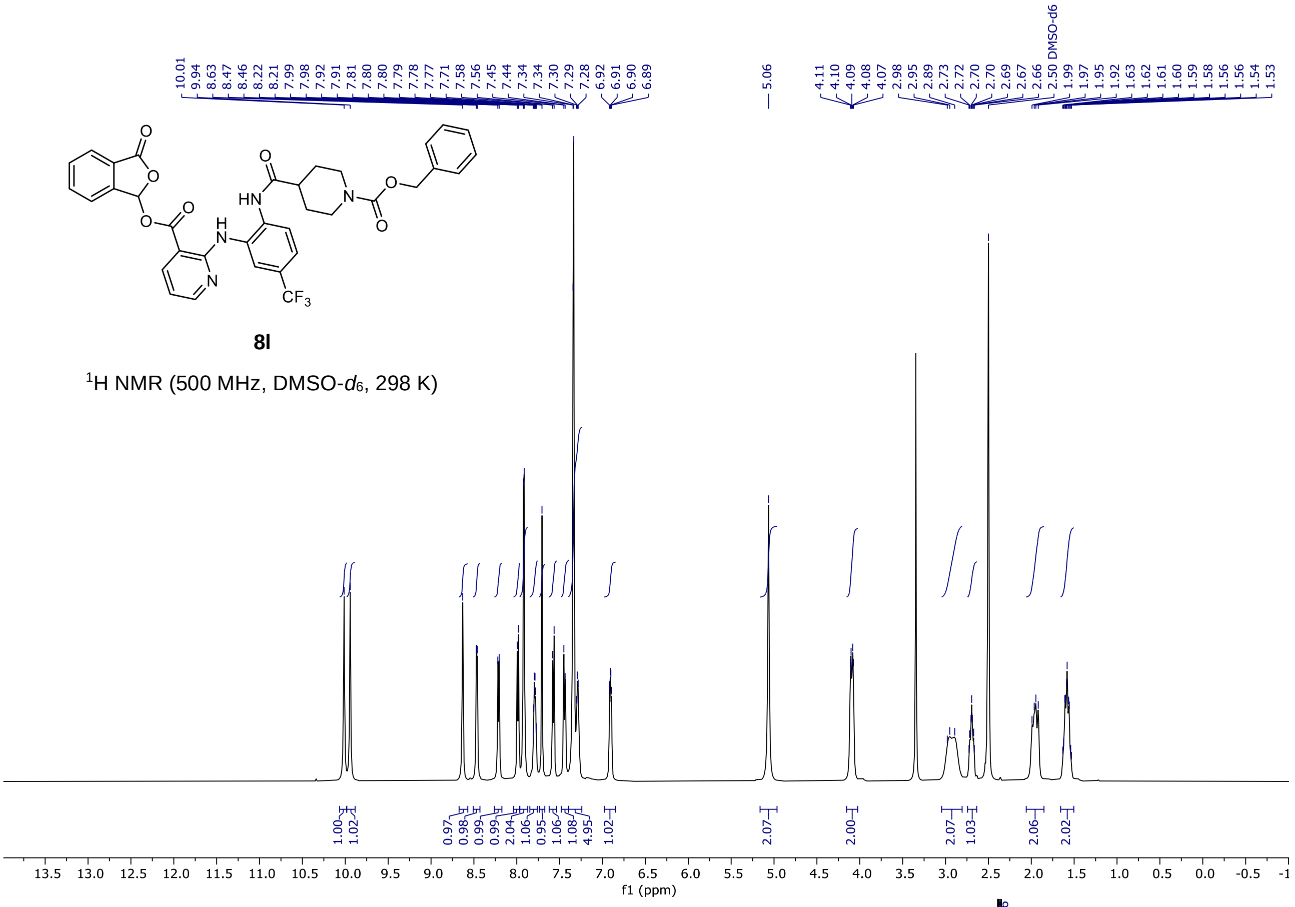

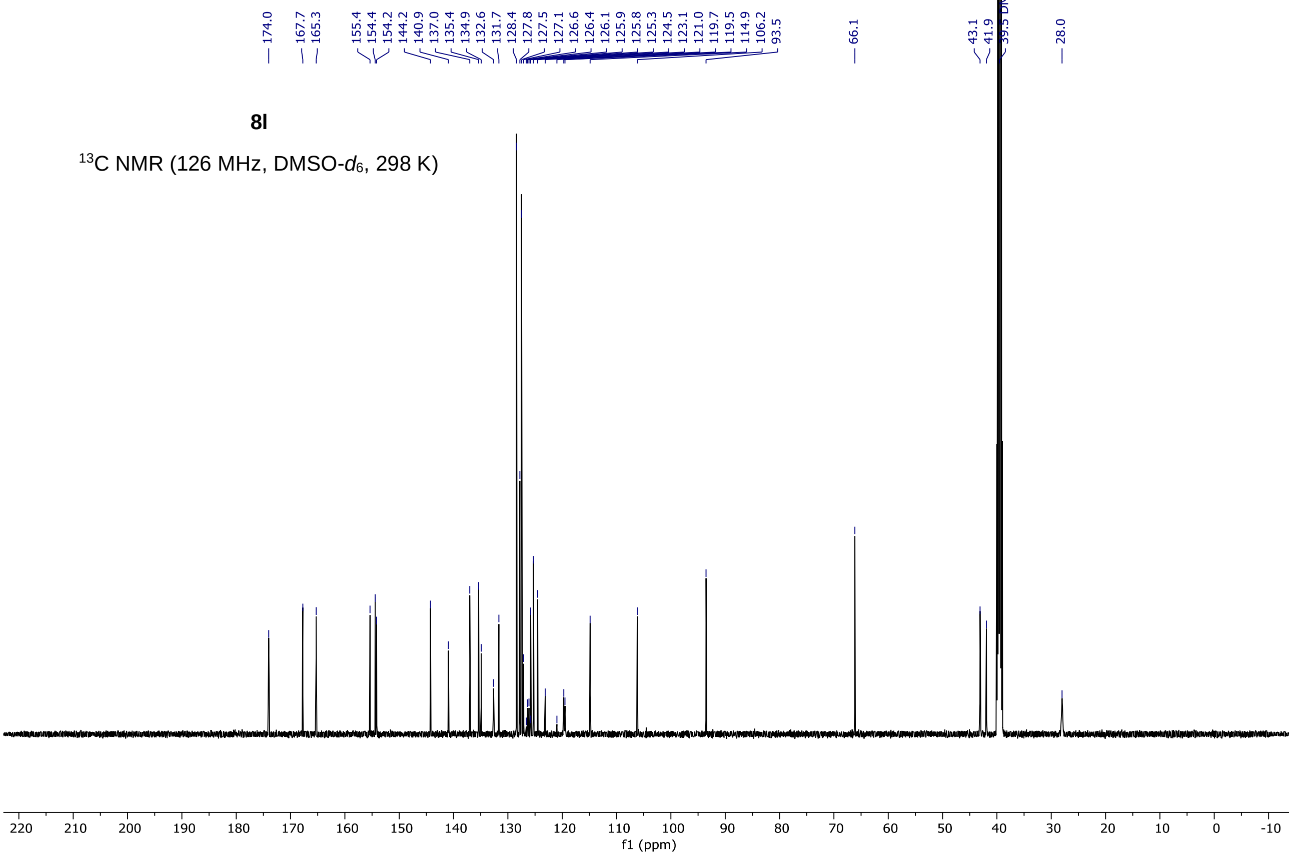

Supplementary Figure 102. ^{1}H NMR (top) and ^{13}C NMR (bottom) spectra of compound **8l**. Frequency, temperature and solvent of measurement are indicated on each spectra.

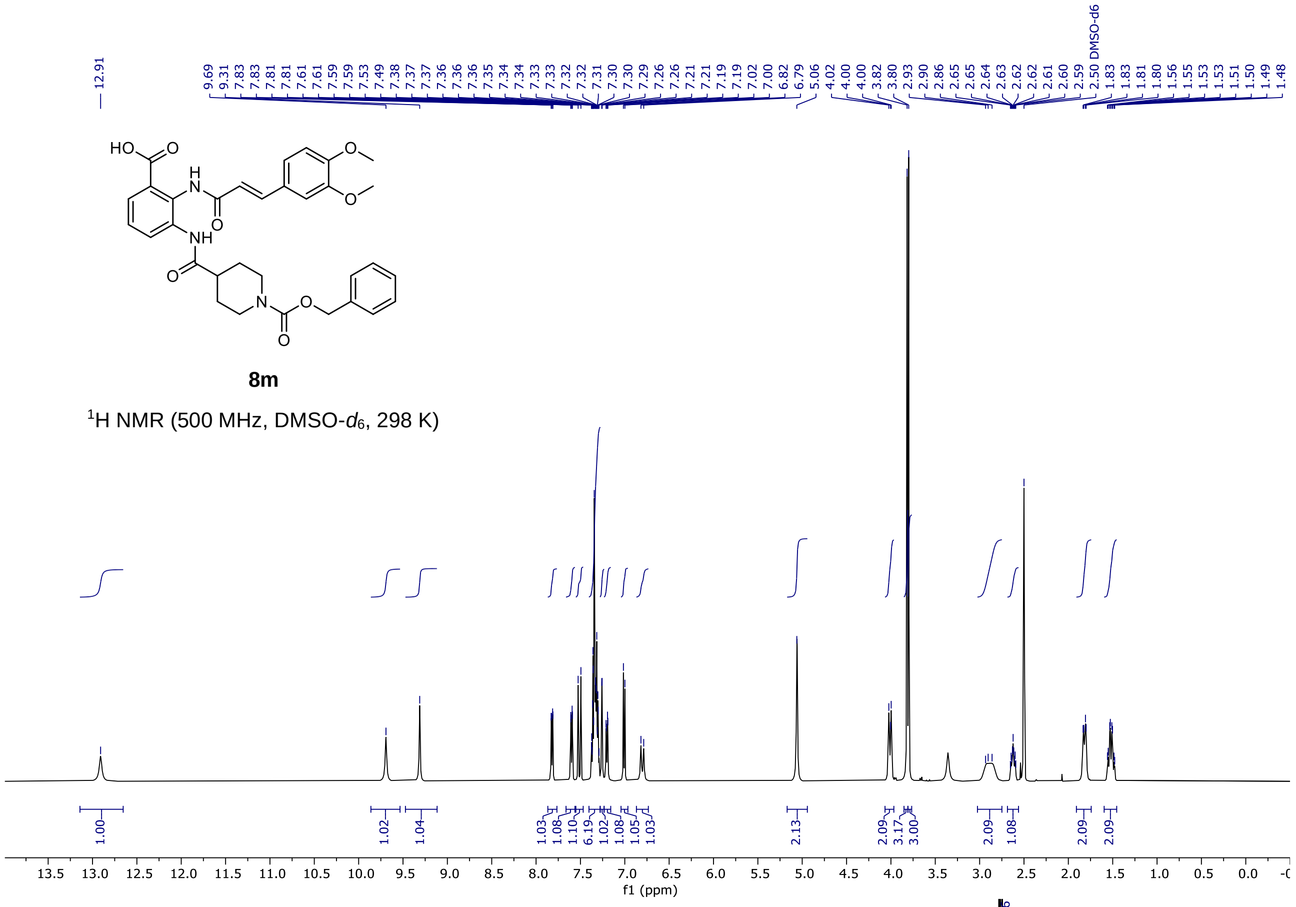

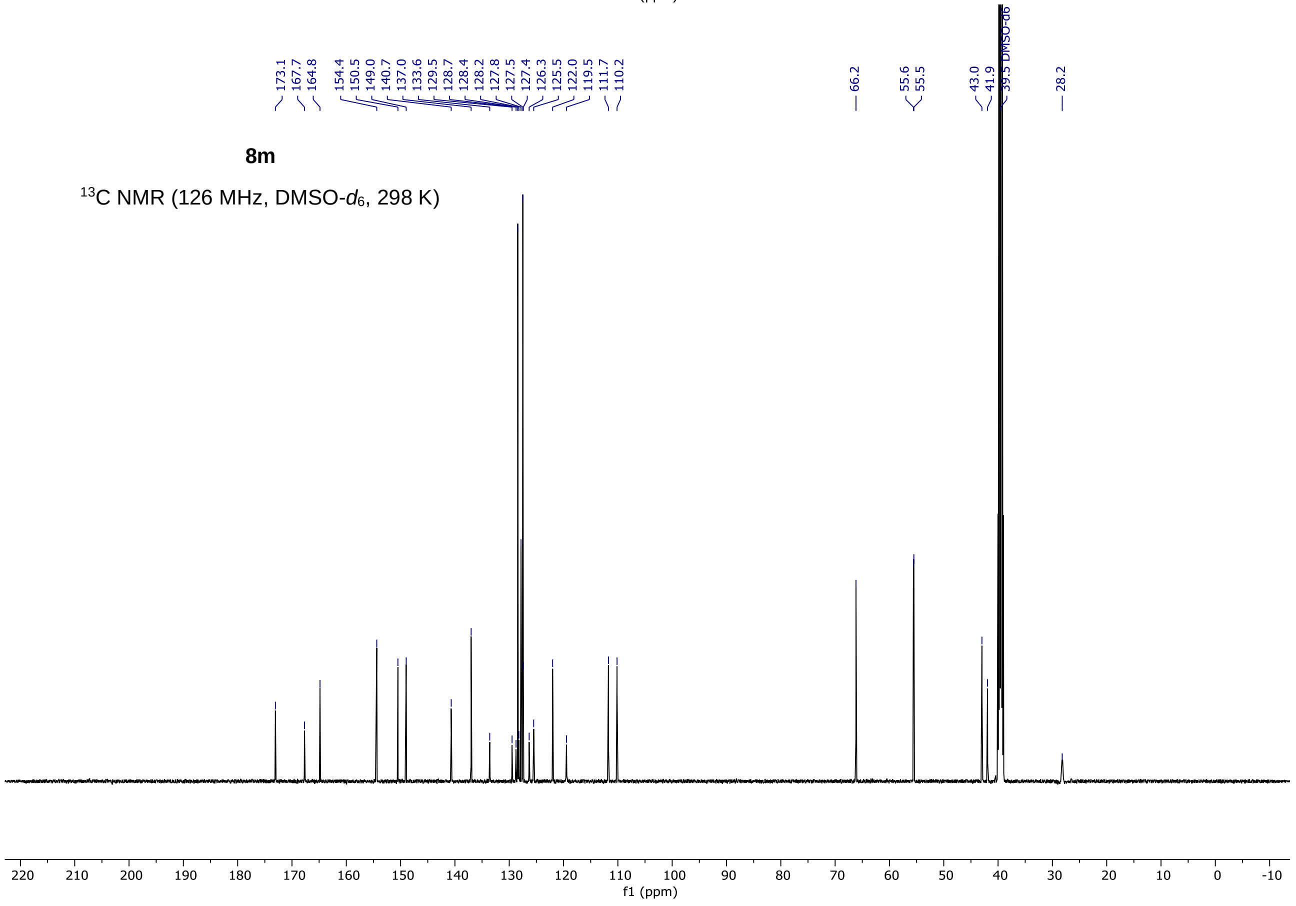

Supplementary Figure 103. ^{1}H NMR (top) and ^{13}C NMR (bottom) spectra of compound **8m**. Frequency, temperature and solvent of measurement are indicated on each spectra.

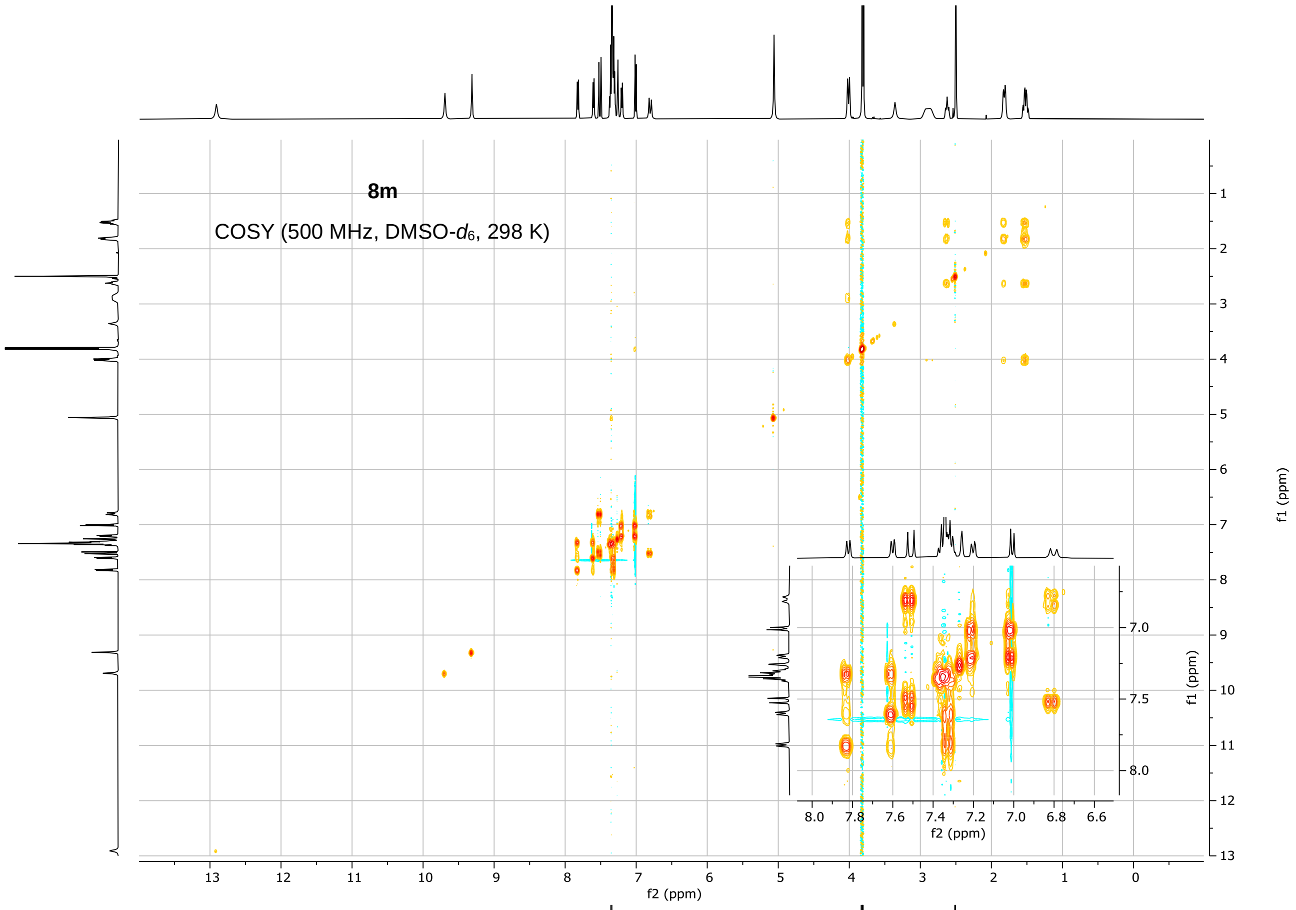

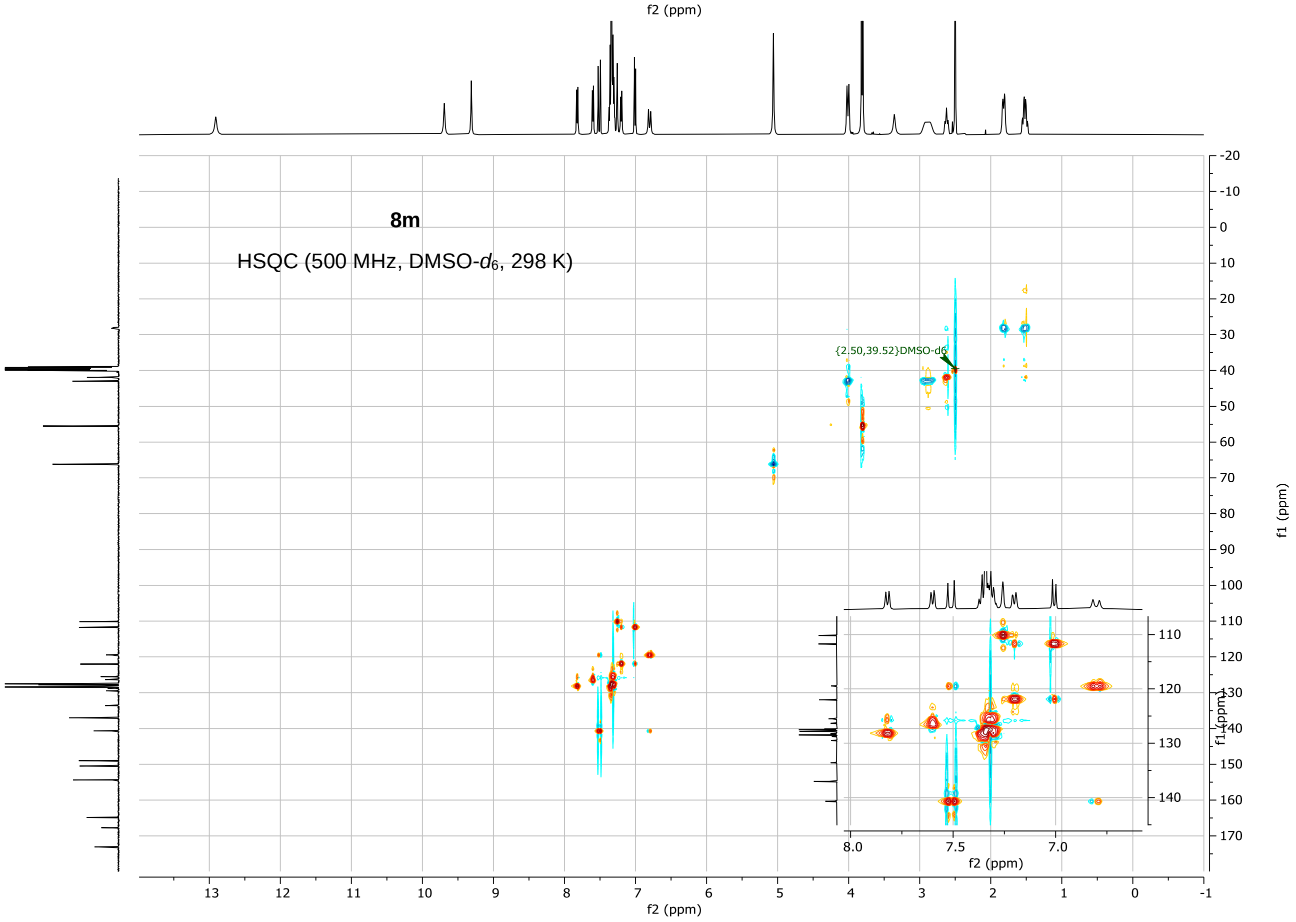

Supplementary Figure 104. COSY (top) and HSQC (bottom) spectra of compound **8m**. Frequency, temperature and solvent of measurement are indicated on each spectra.

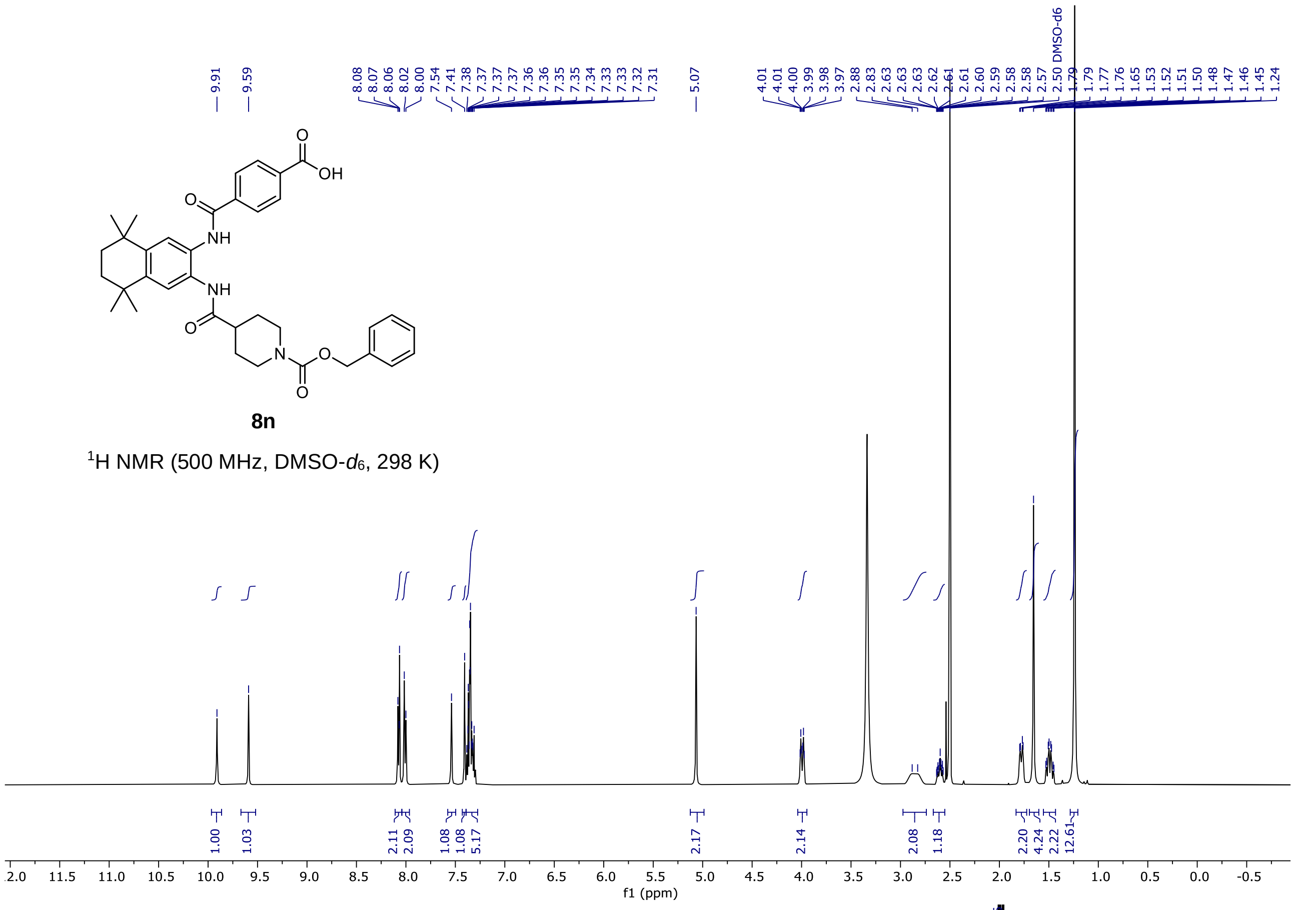

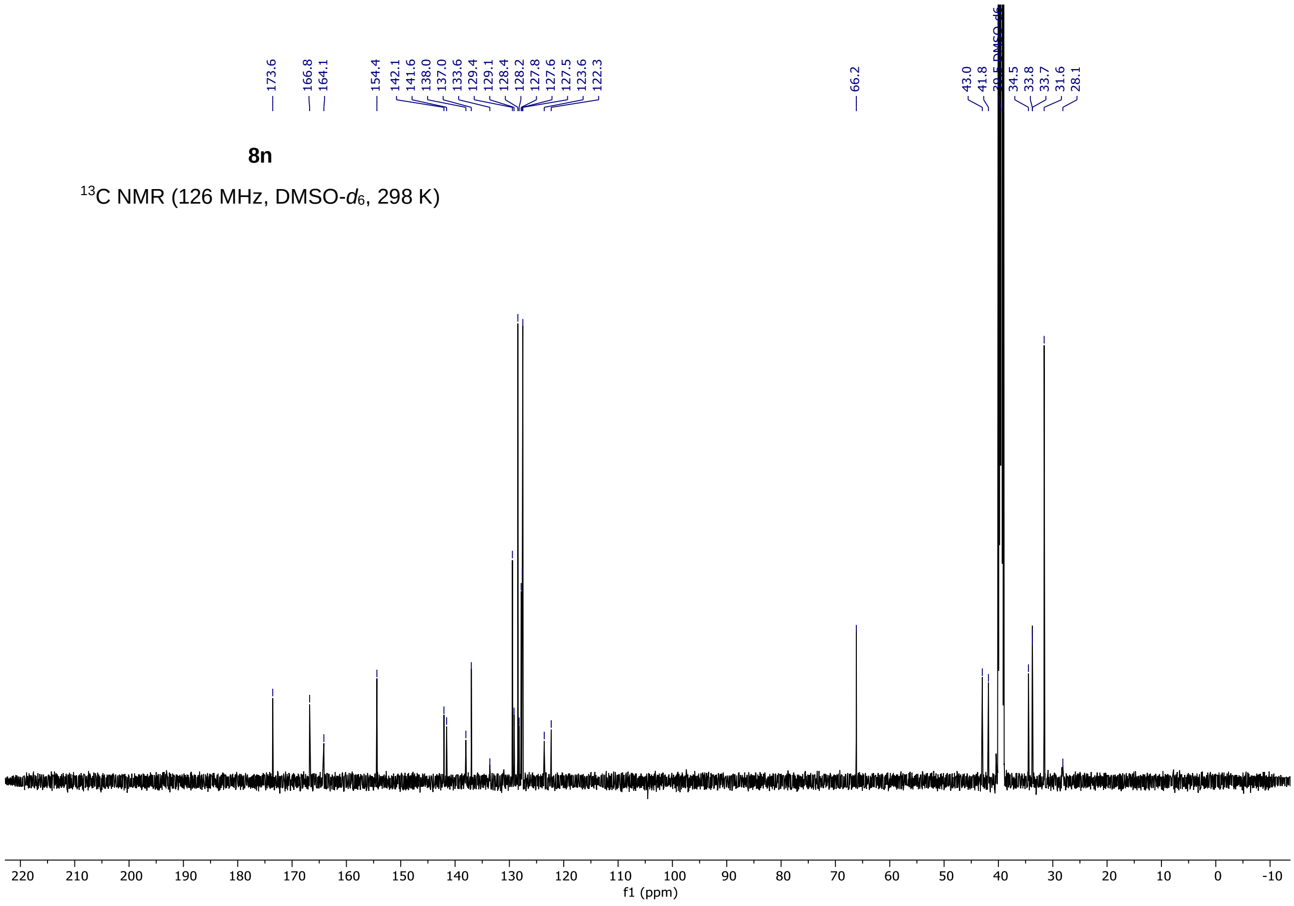

Supplementary Figure 105. ^{1}H NMR (top) and ^{13}C NMR (bottom) spectra of compound **8n**. Frequency, temperature and solvent of measurement are indicated on each spectra.

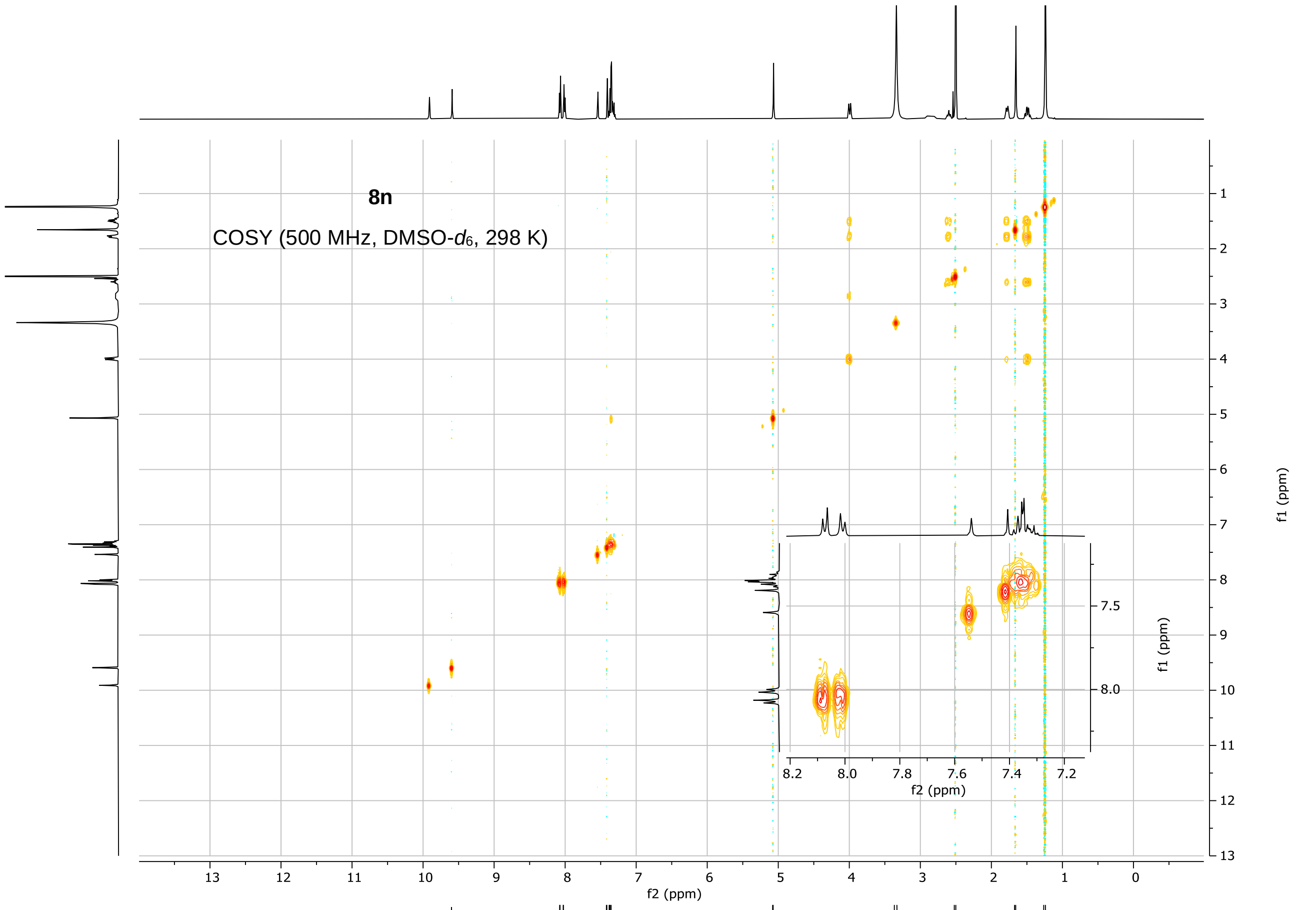

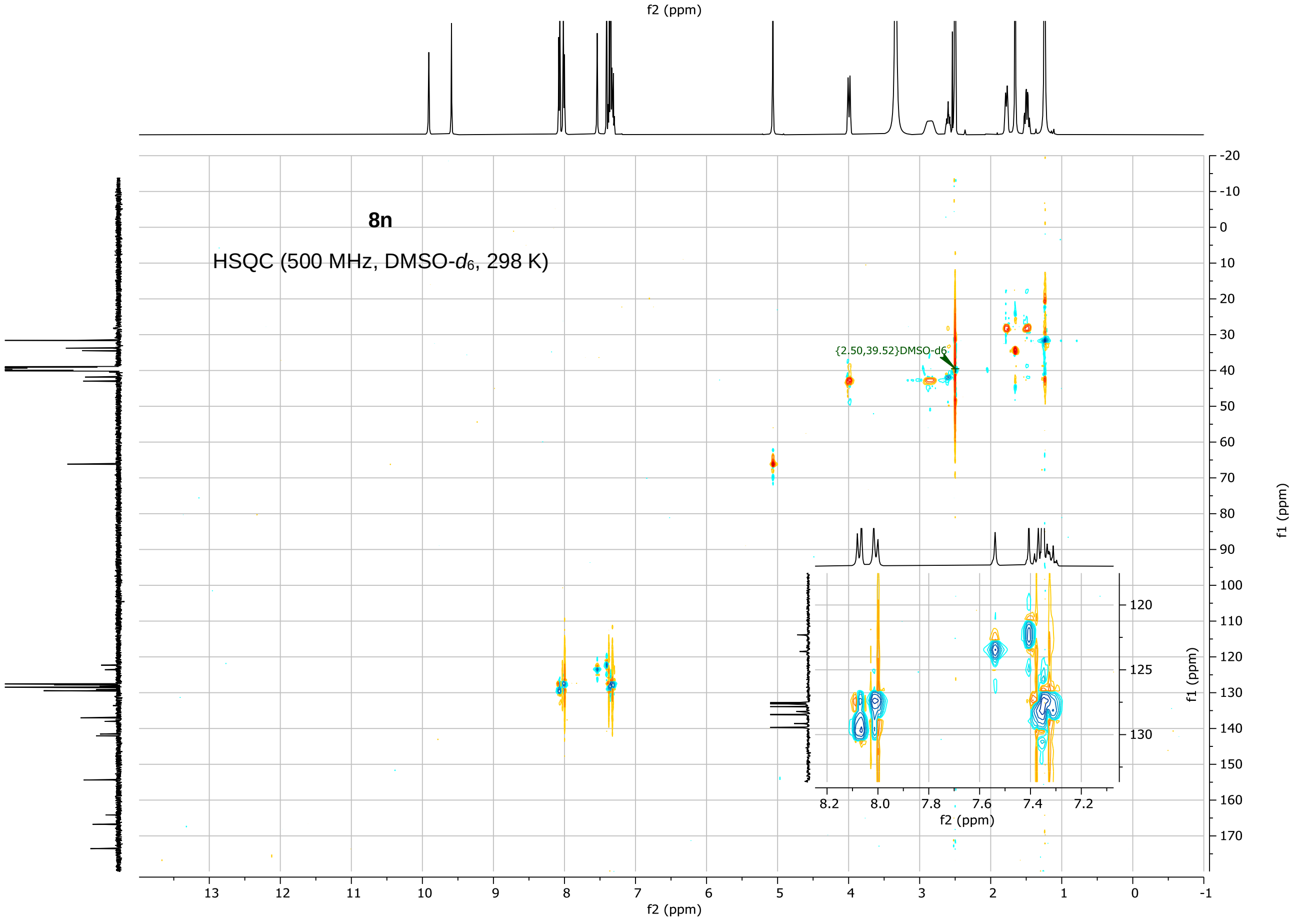

Supplementary Figure 106. COSY (top) and HSQC (bottom) spectra of compound **8n**. Frequency, temperature and solvent of measurement are indicated on each spectra.

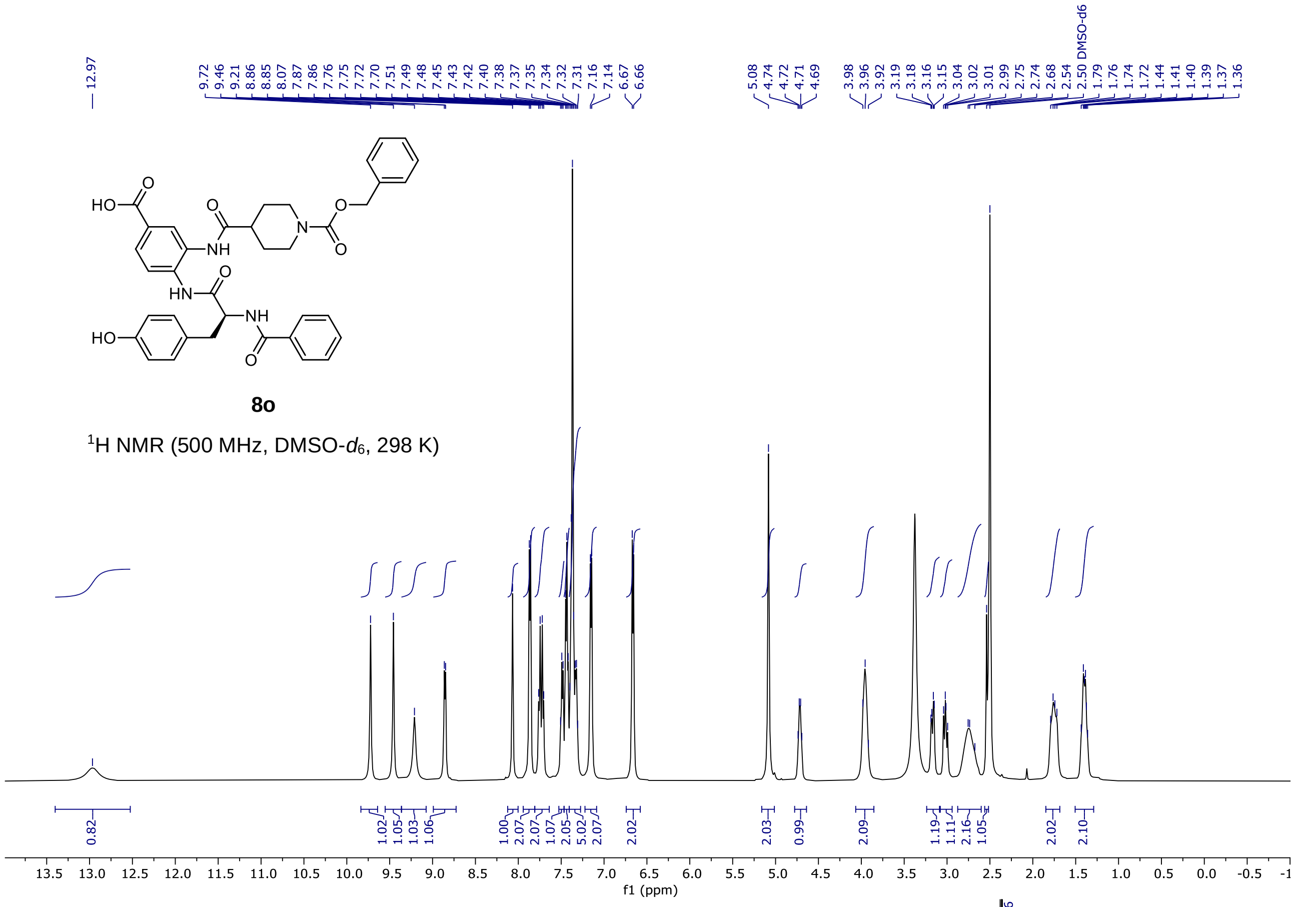

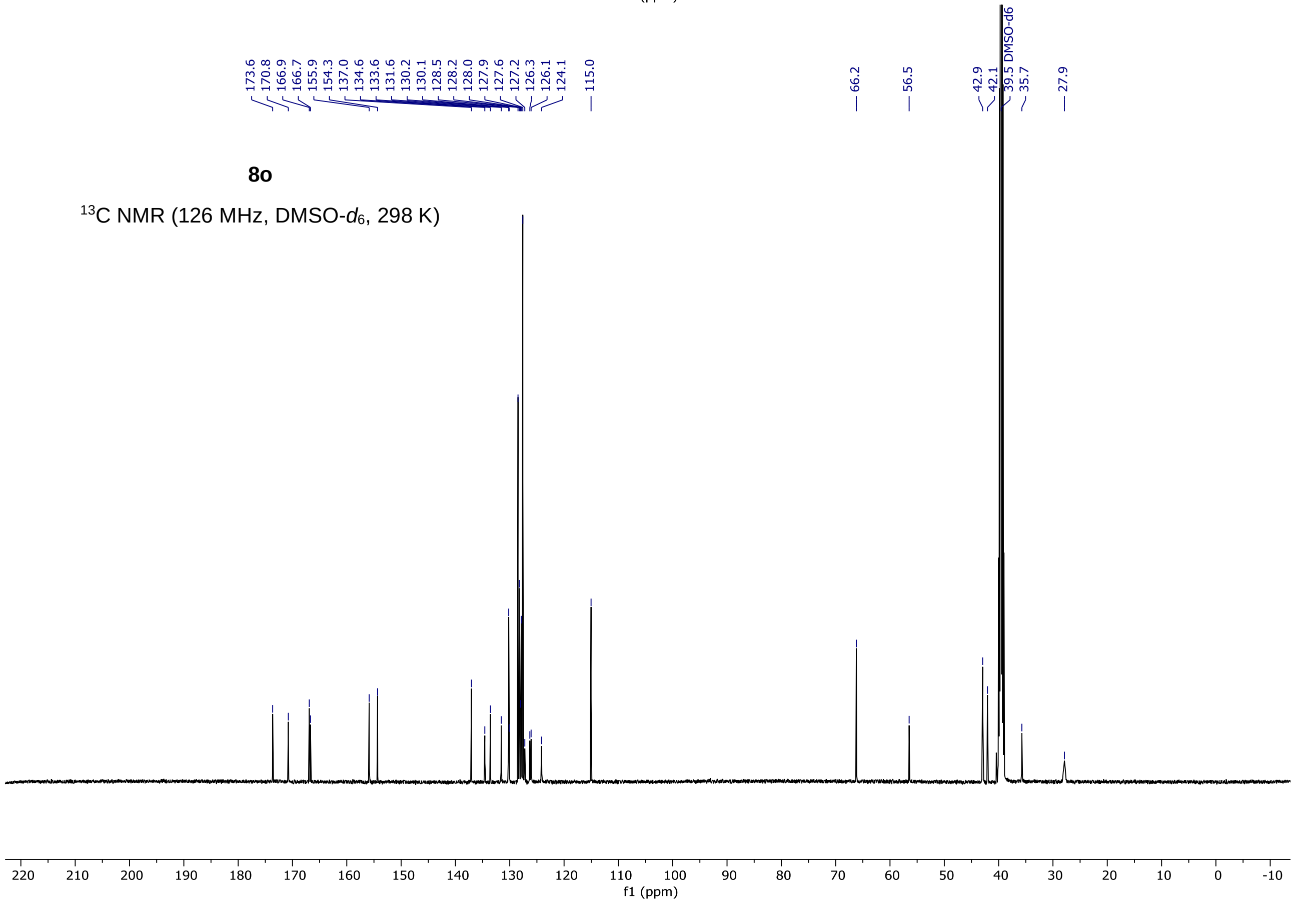

Supplementary Figure 107. ^{1}H NMR (top) and ^{13}C NMR (bottom) spectra of compound **8o**. Frequency, temperature and solvent of measurement are indicated on each spectra.

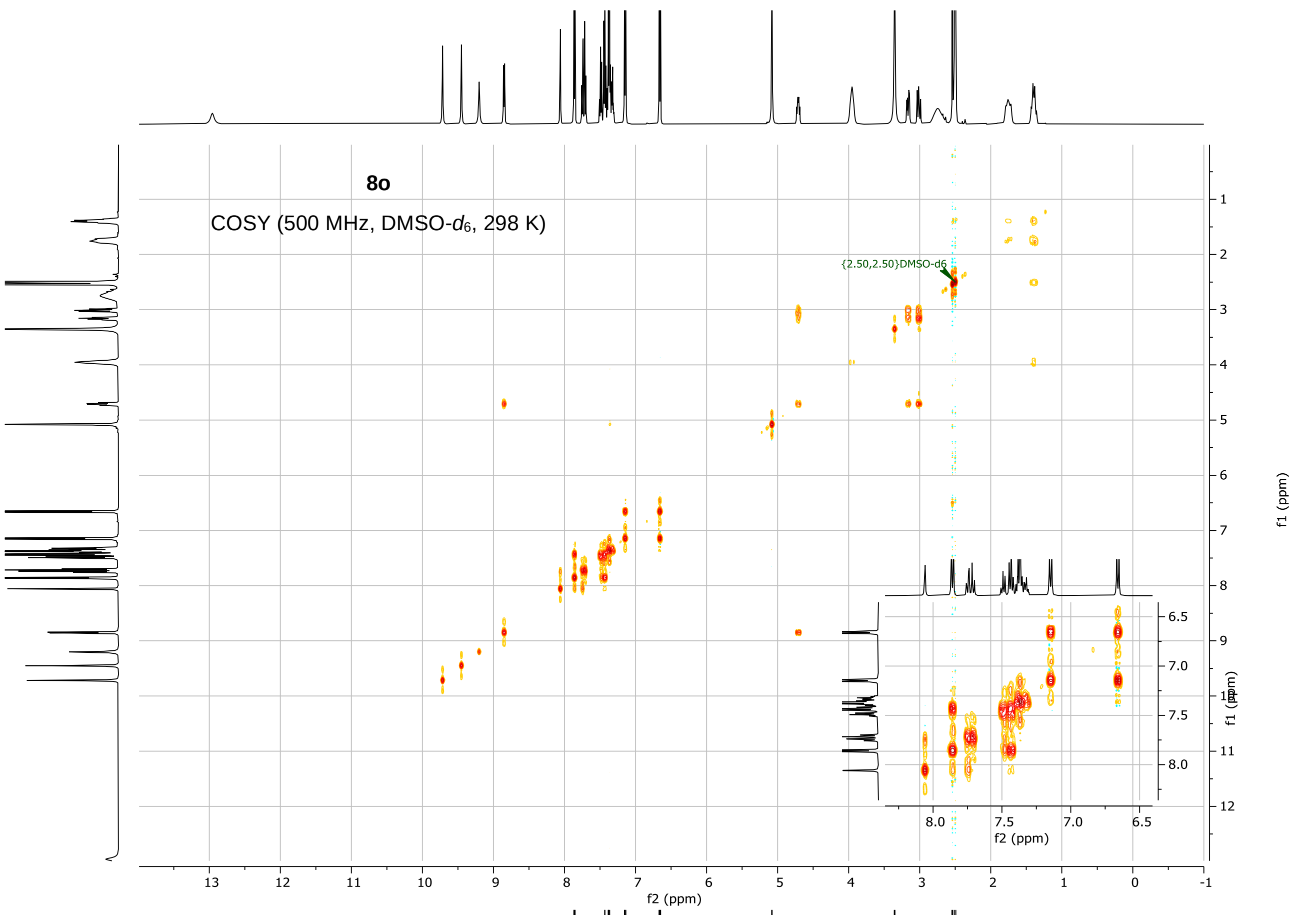

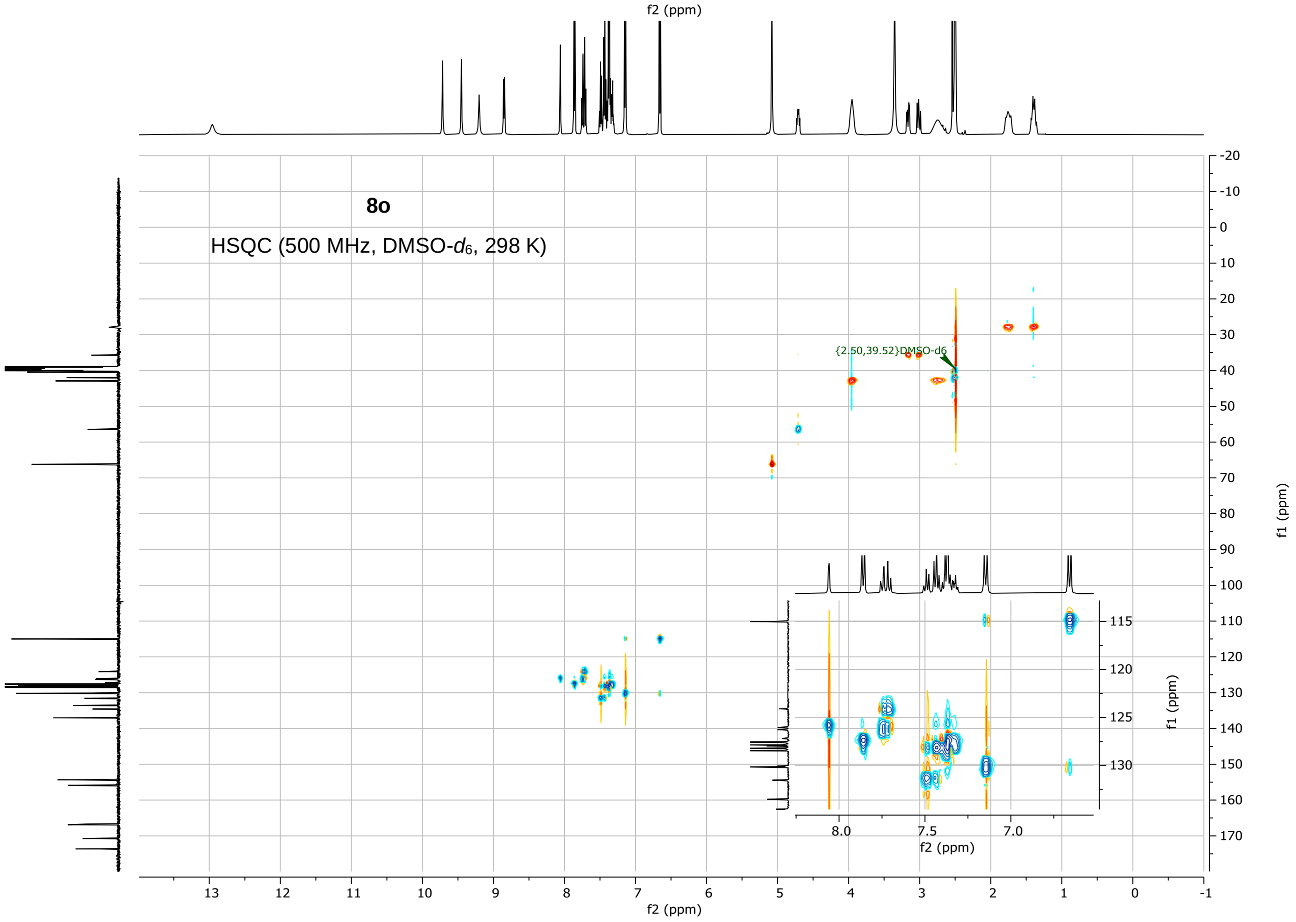

Supplementary Figure 108. COSY (top) and HSQC (bottom) spectra of compound **8o**. Frequency, temperature and solvent of measurement are indicated on each spectra.

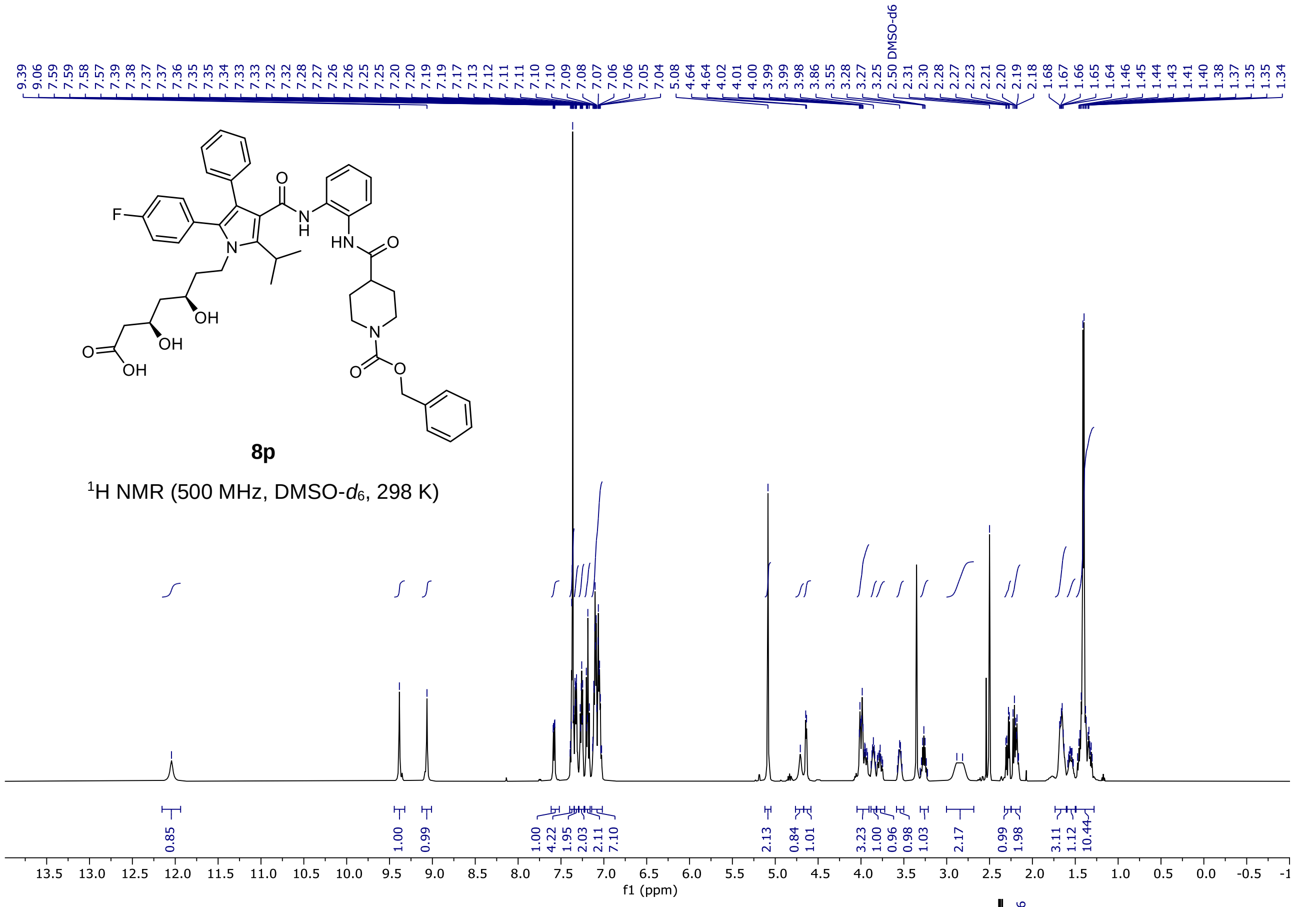

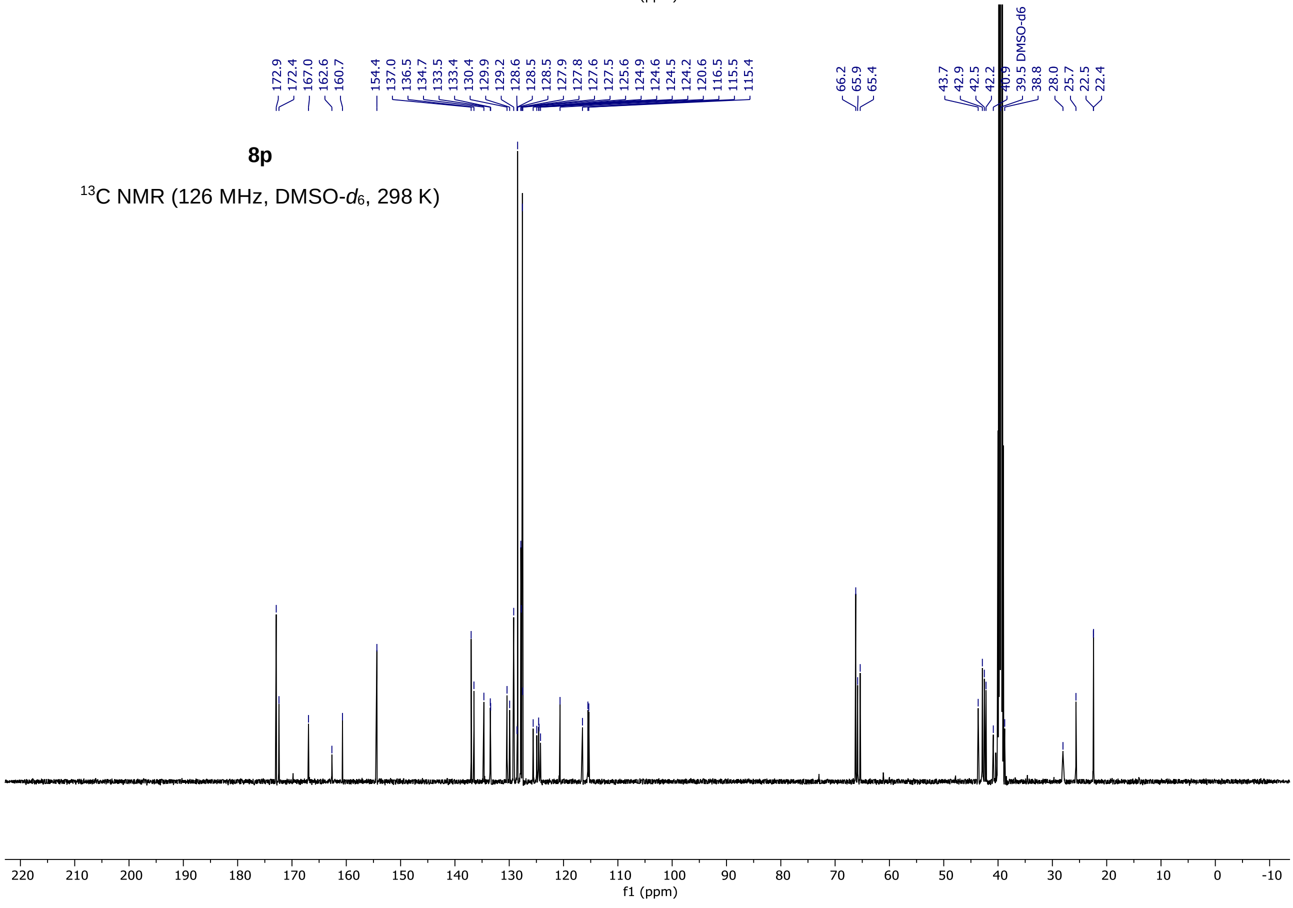

Supplementary Figure 109. ^{1}H NMR (top) and ^{13}C NMR (bottom) spectra of compound **8p**. Frequency, temperature and solvent of measurement are indicated on each spectra.

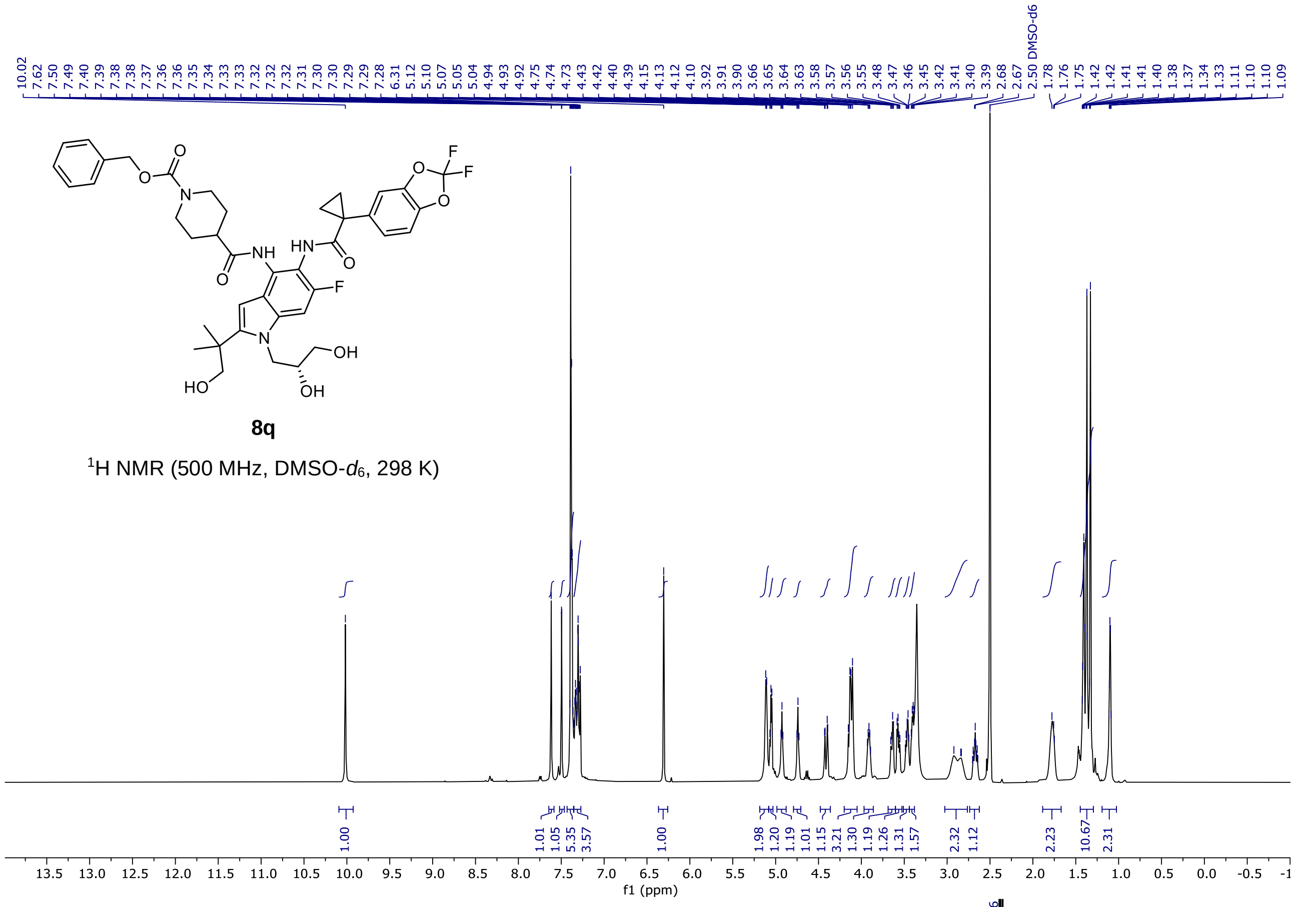

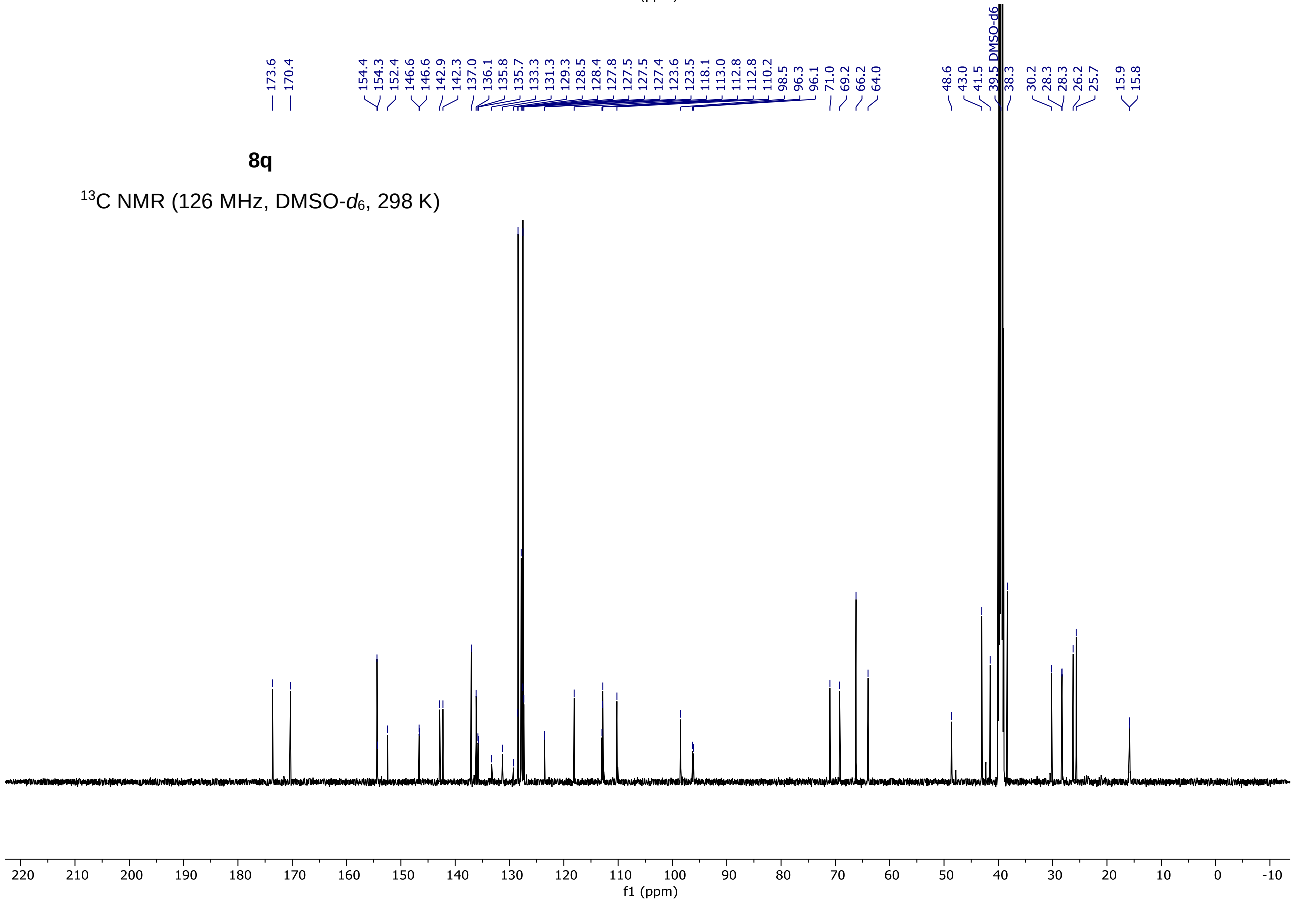

Supplementary Figure 110. ^{1}H NMR (top) and ^{13}C NMR (bottom) spectra of compound **8q**. Frequency, temperature and solvent of measurement are indicated on each spectra.

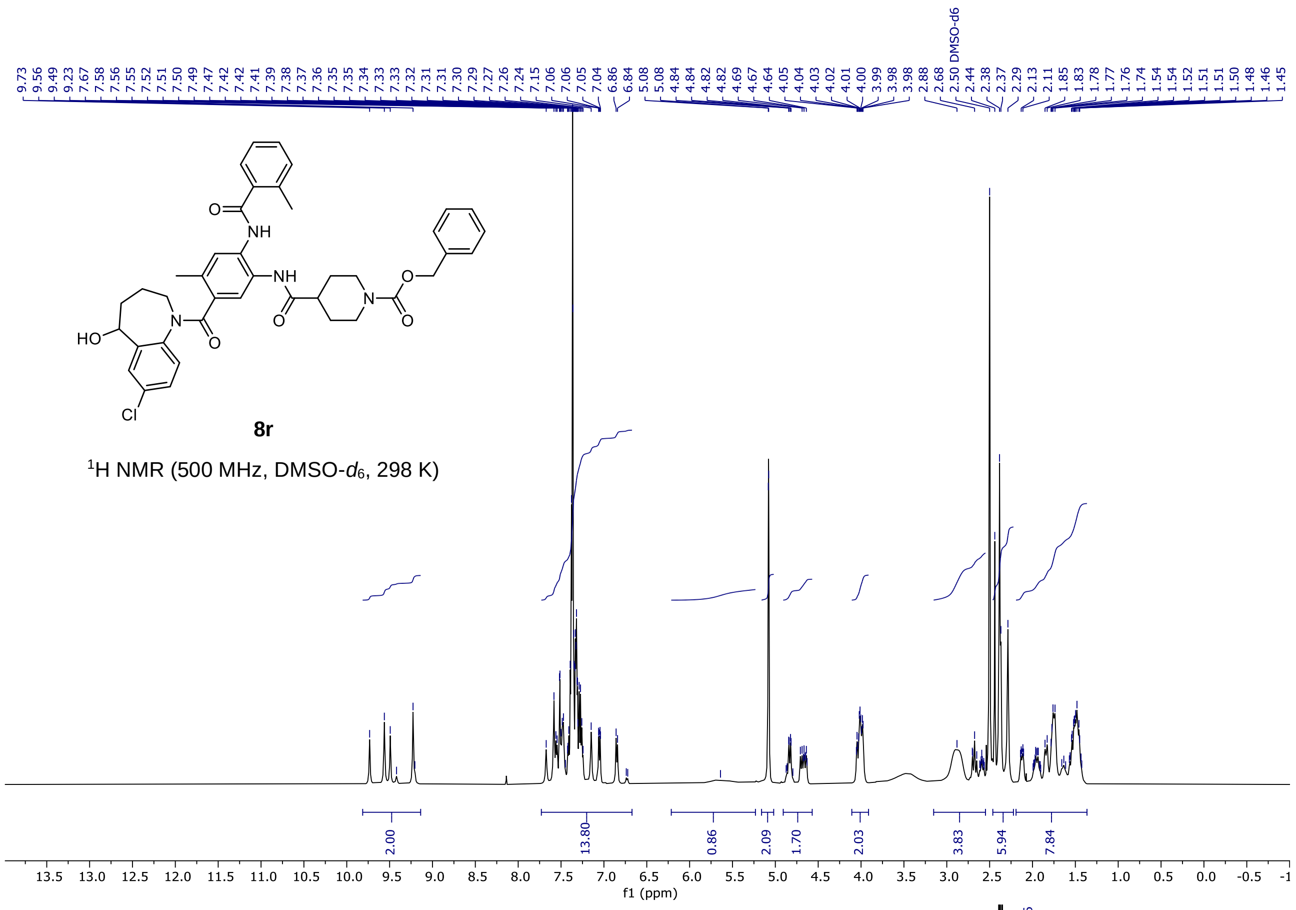

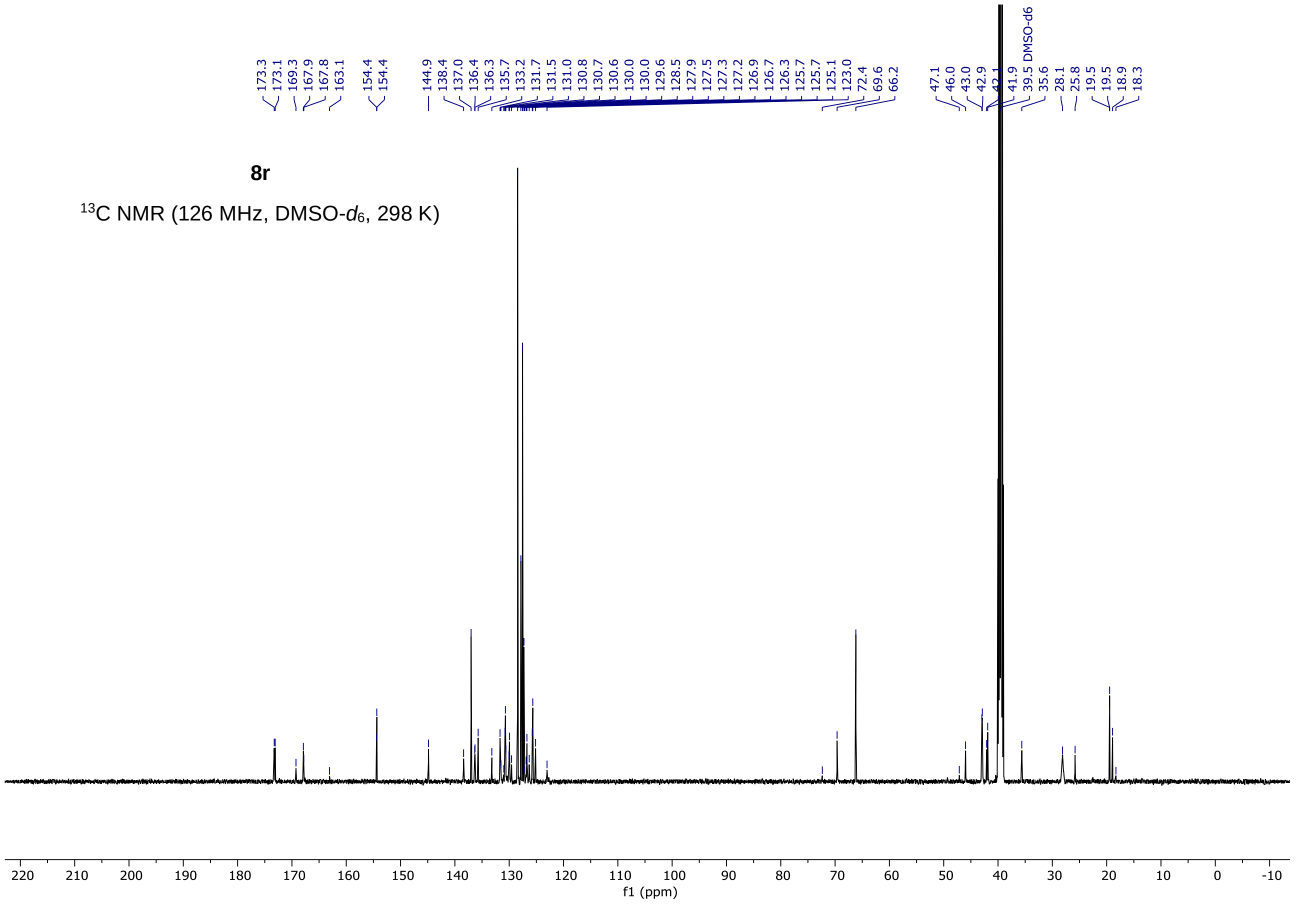

Supplementary Figure 111. ^{1}H NMR (top) and ^{13}C NMR (bottom) spectra of compound **8r**. Frequency, temperature and solvent of measurement are indicated on each spectra.

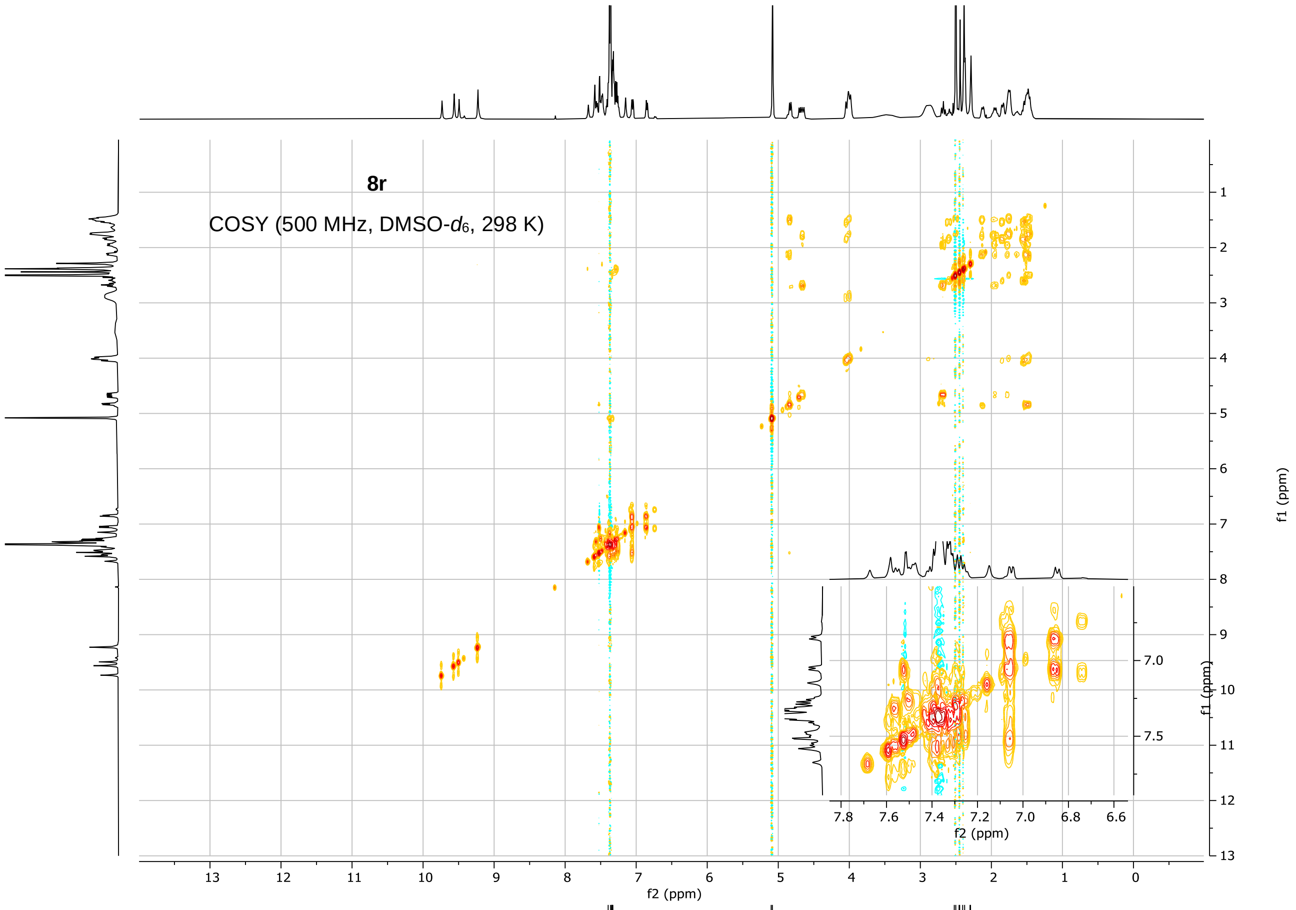

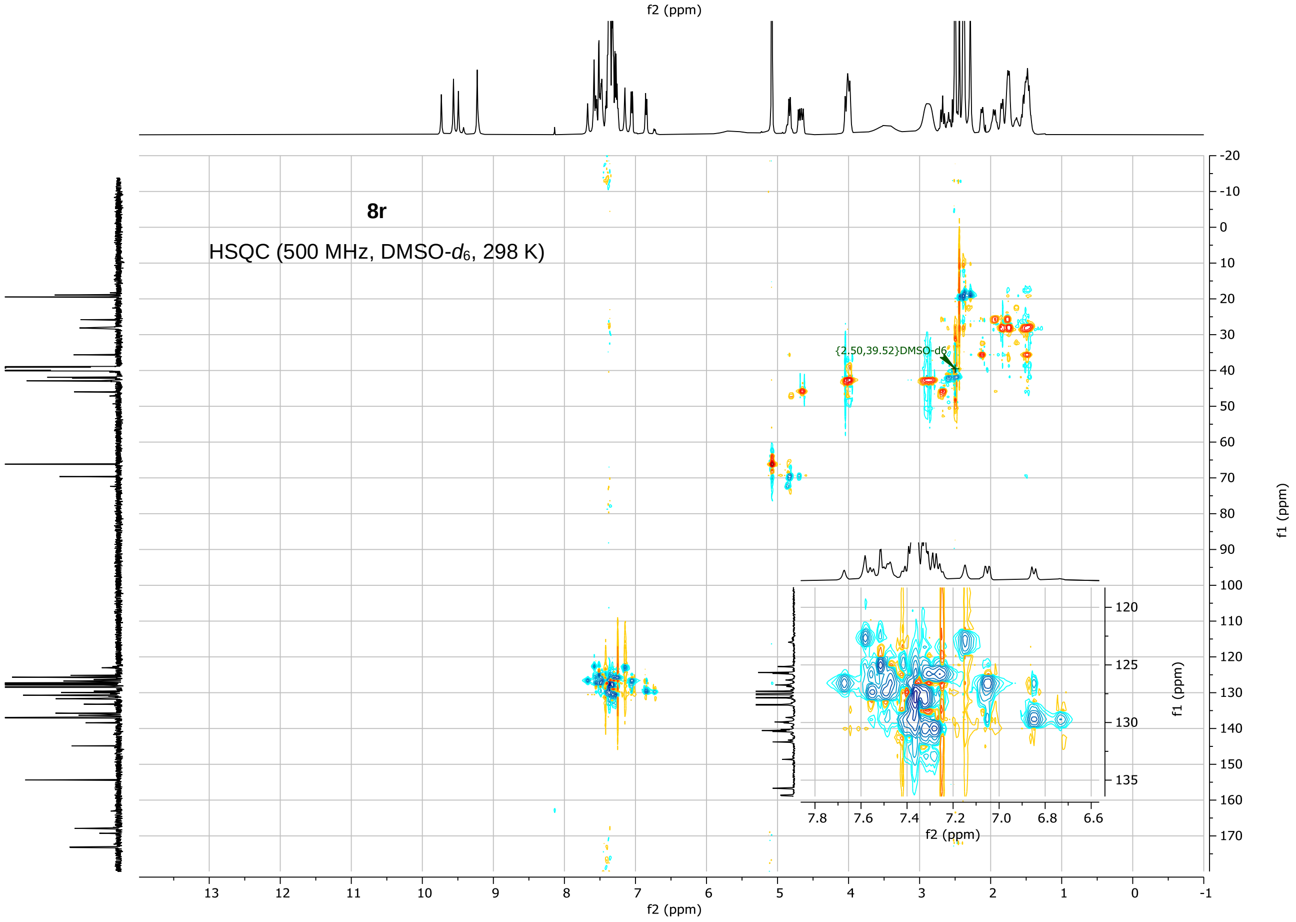

Supplementary Figure 112. COSY (top) and HSQC (bottom) spectra of compound **8r**. Frequency, temperature and solvent of measurement are indicated on each spectra.

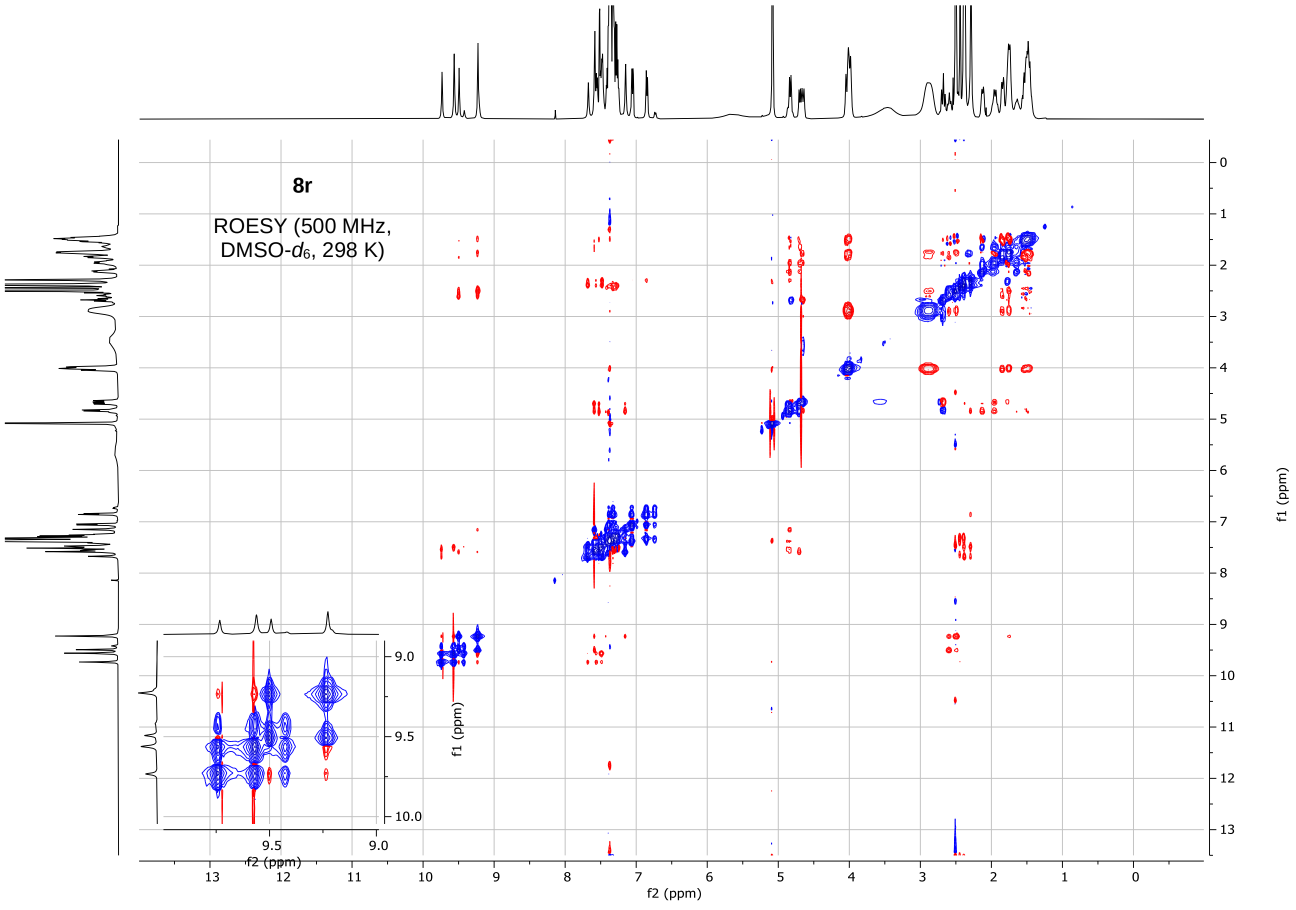

Supplementary Figure 113. ROESY spectrum of compound **8r**. Frequency, temperature and solvent of measurement are indicated on the spectrum.

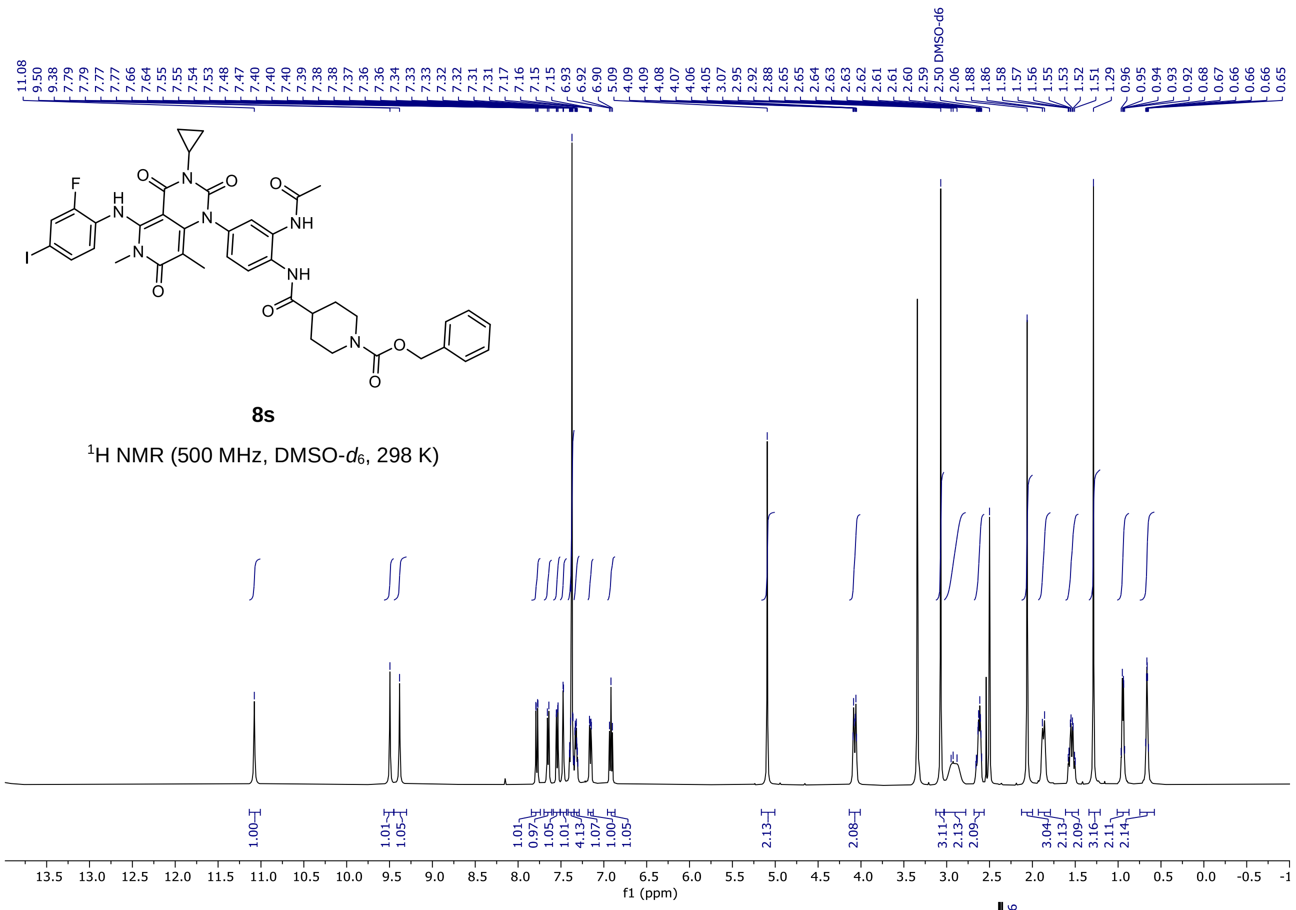

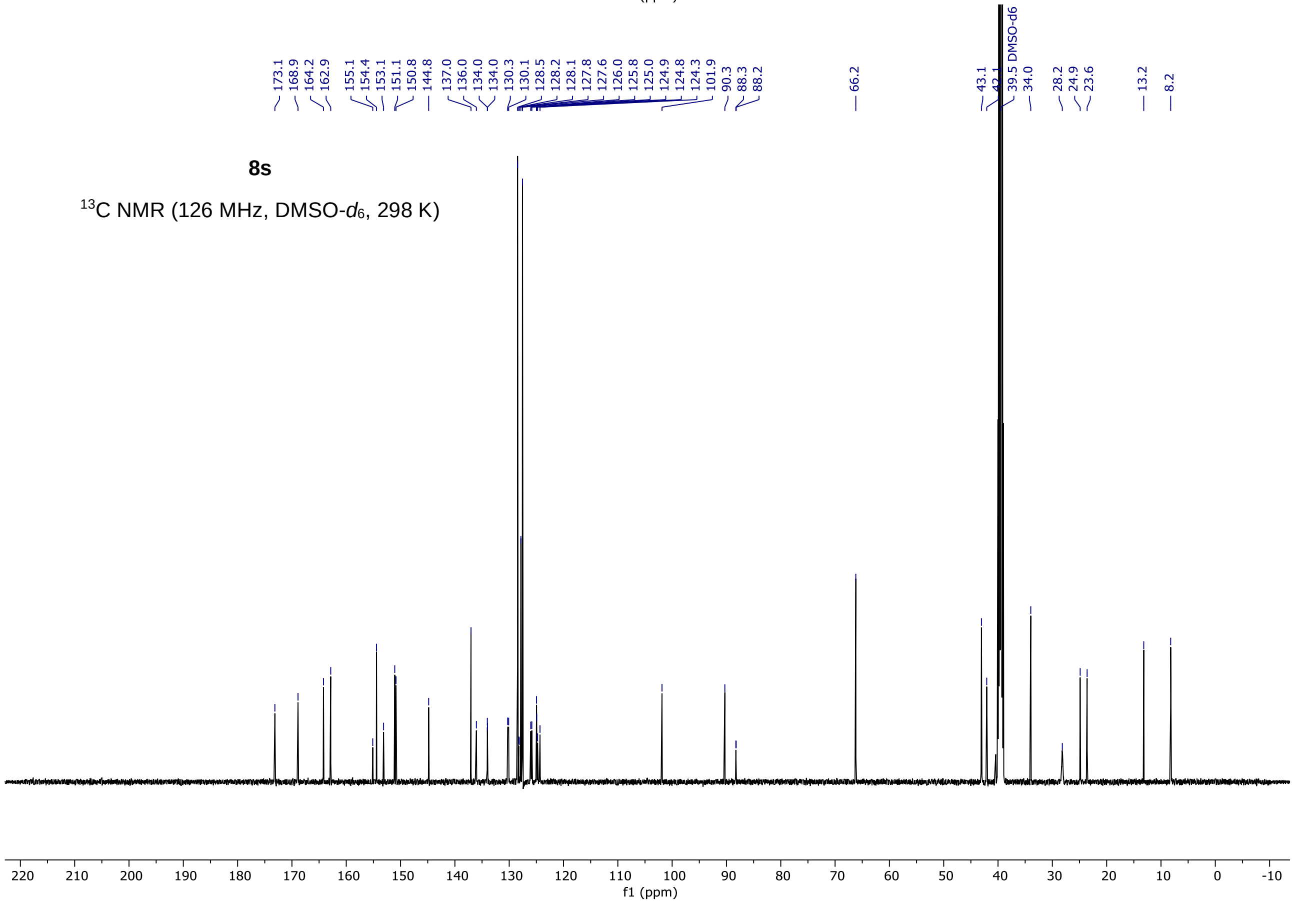

Supplementary Figure 114. ^{1}H NMR (top) and ^{13}C NMR (bottom) spectra of compound **8s**. Frequency, temperature and solvent of measurement are indicated on each spectra.

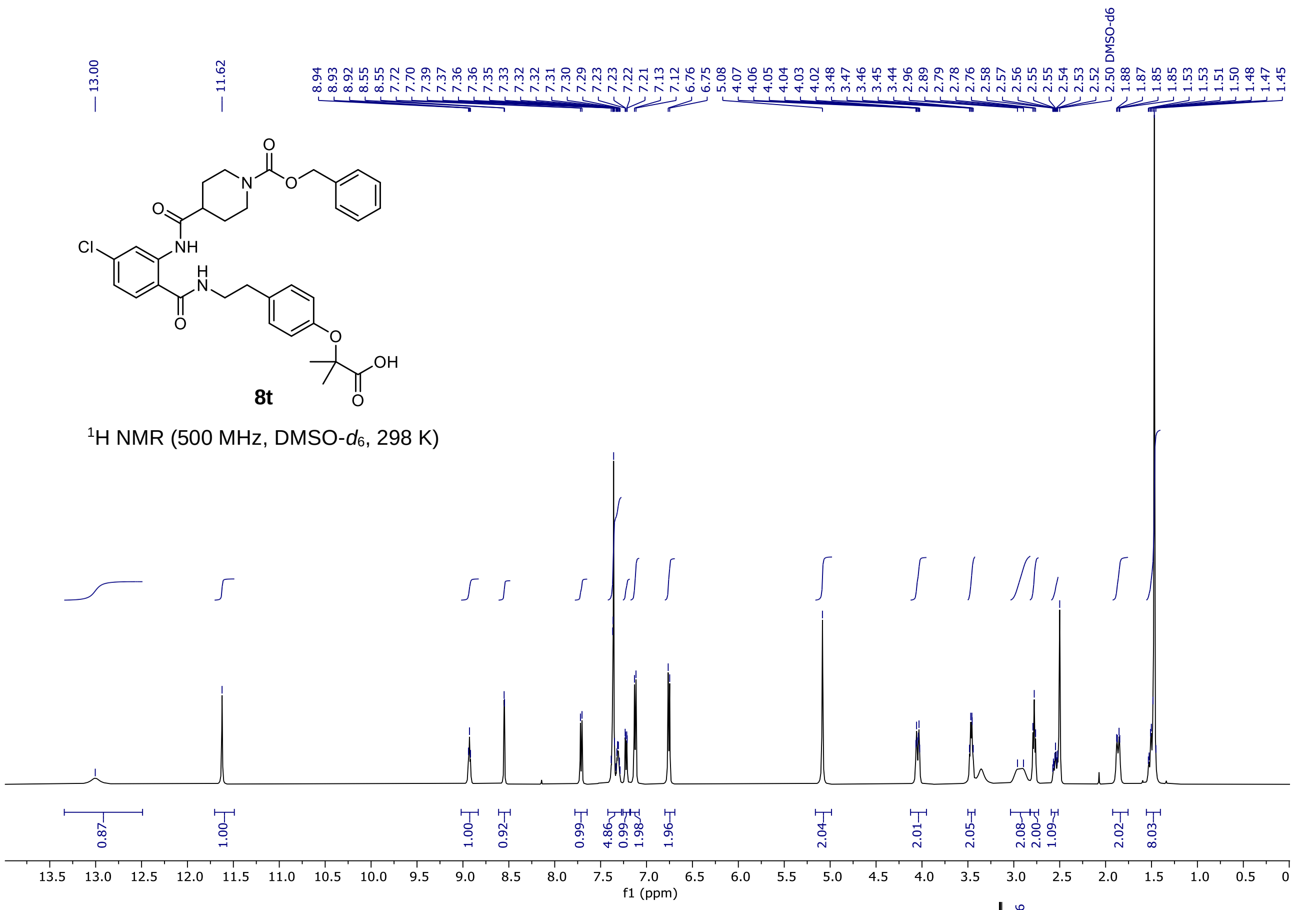

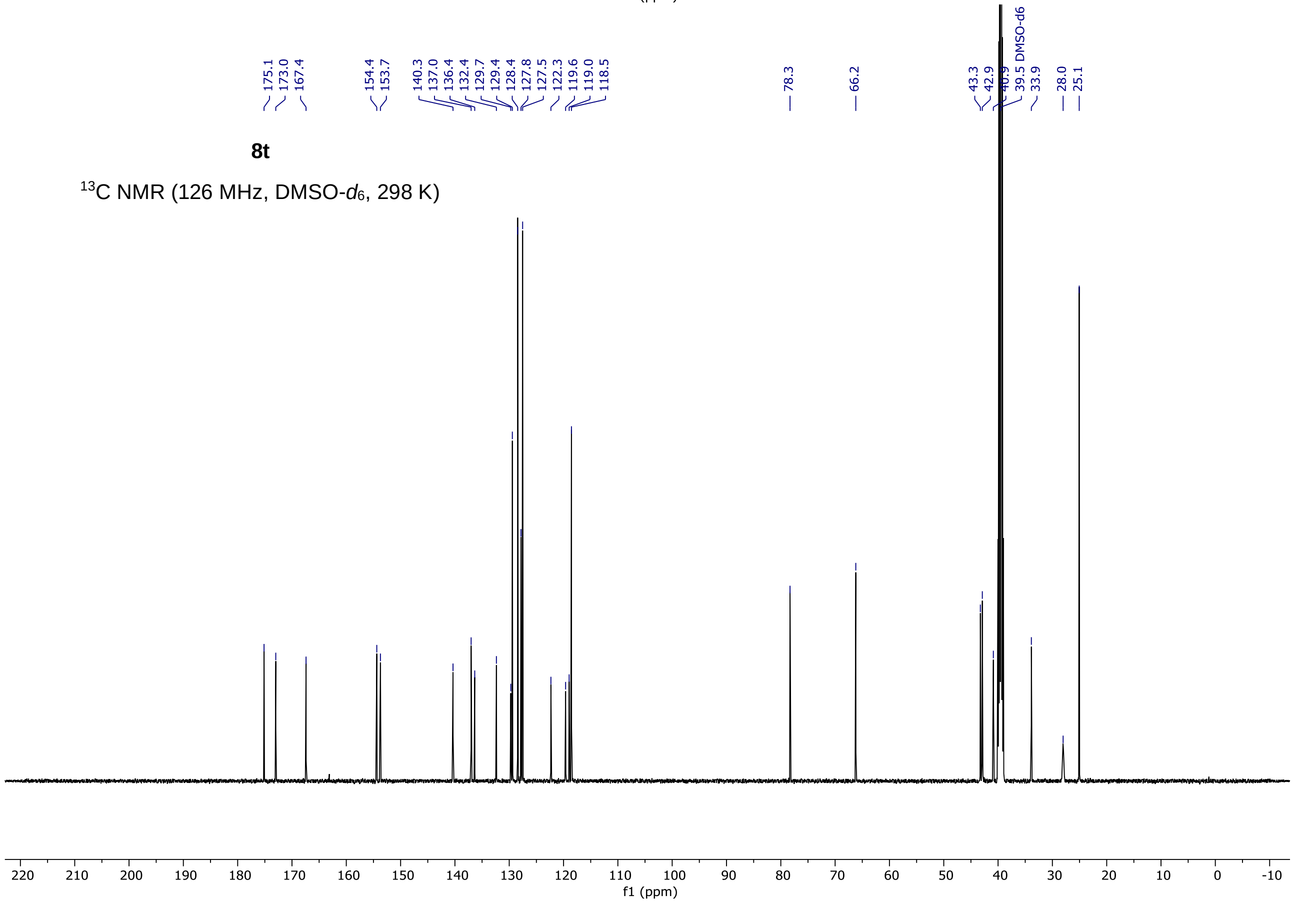

Supplementary Figure 115. ^{1}H NMR (top) and ^{13}C NMR (bottom) spectra of compound **8t**. Frequency, temperature and solvent of measurement are indicated on each spectra.

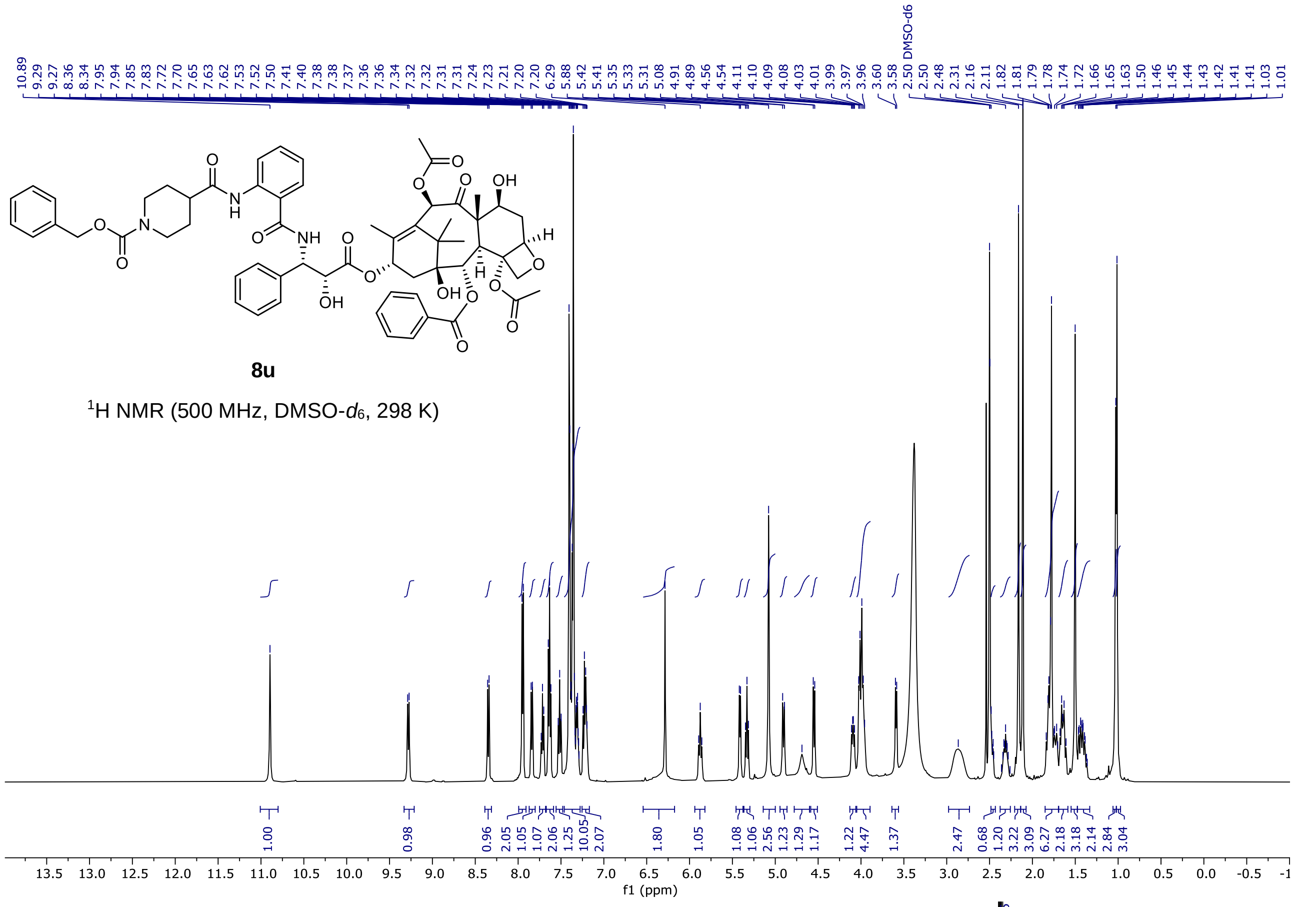

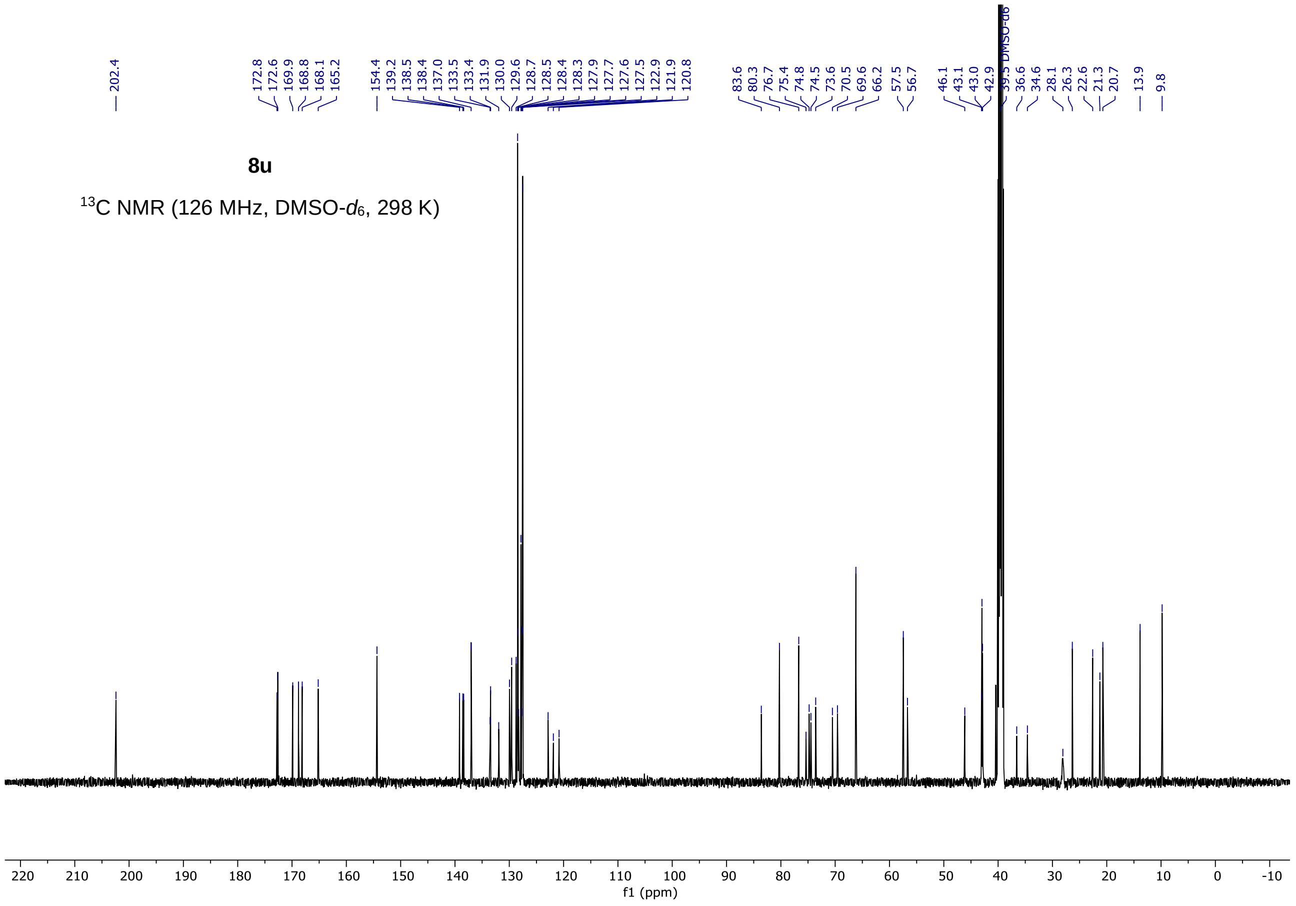

Supplementary Figure 116. ^{1}H NMR (top) and ^{13}C NMR (bottom) spectra of compound **8u**. Frequency, temperature and solvent of measurement are indicated on each spectra.

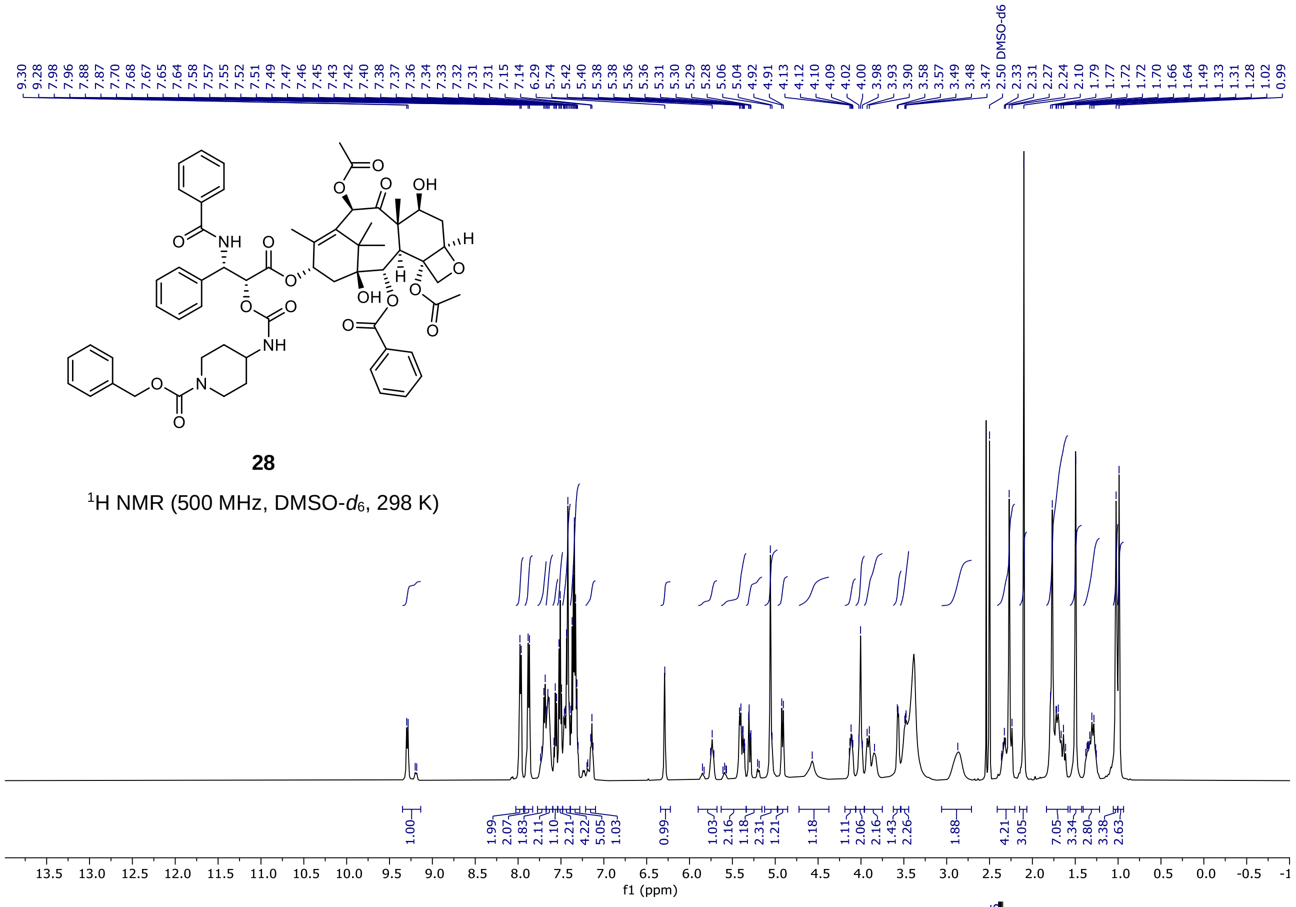

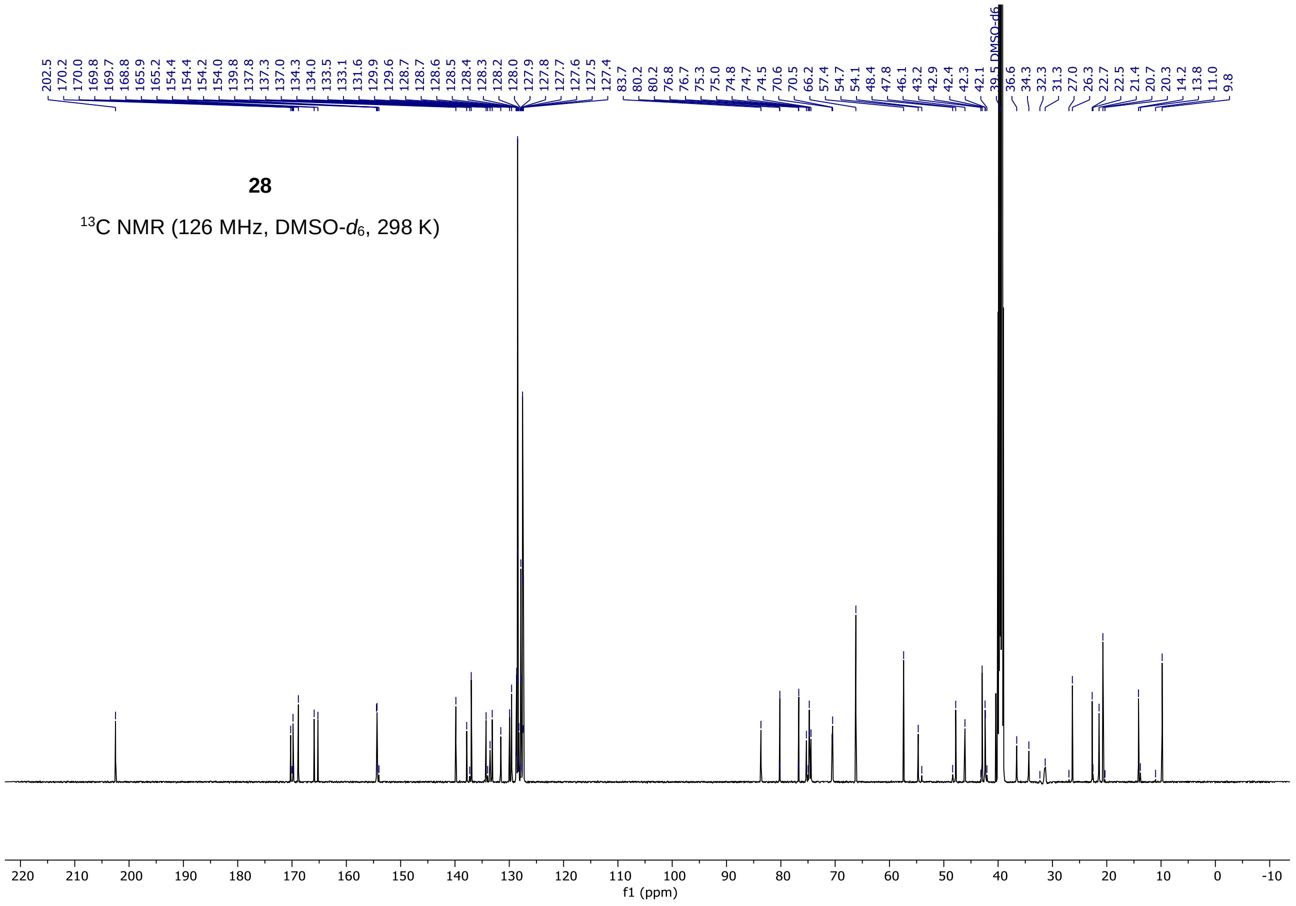

Supplementary Figure 117. ^{1}H NMR (top) and ^{13}C NMR (bottom) spectra of compound **28**. Frequency, temperature and solvent of measurement are indicated on each spectra.

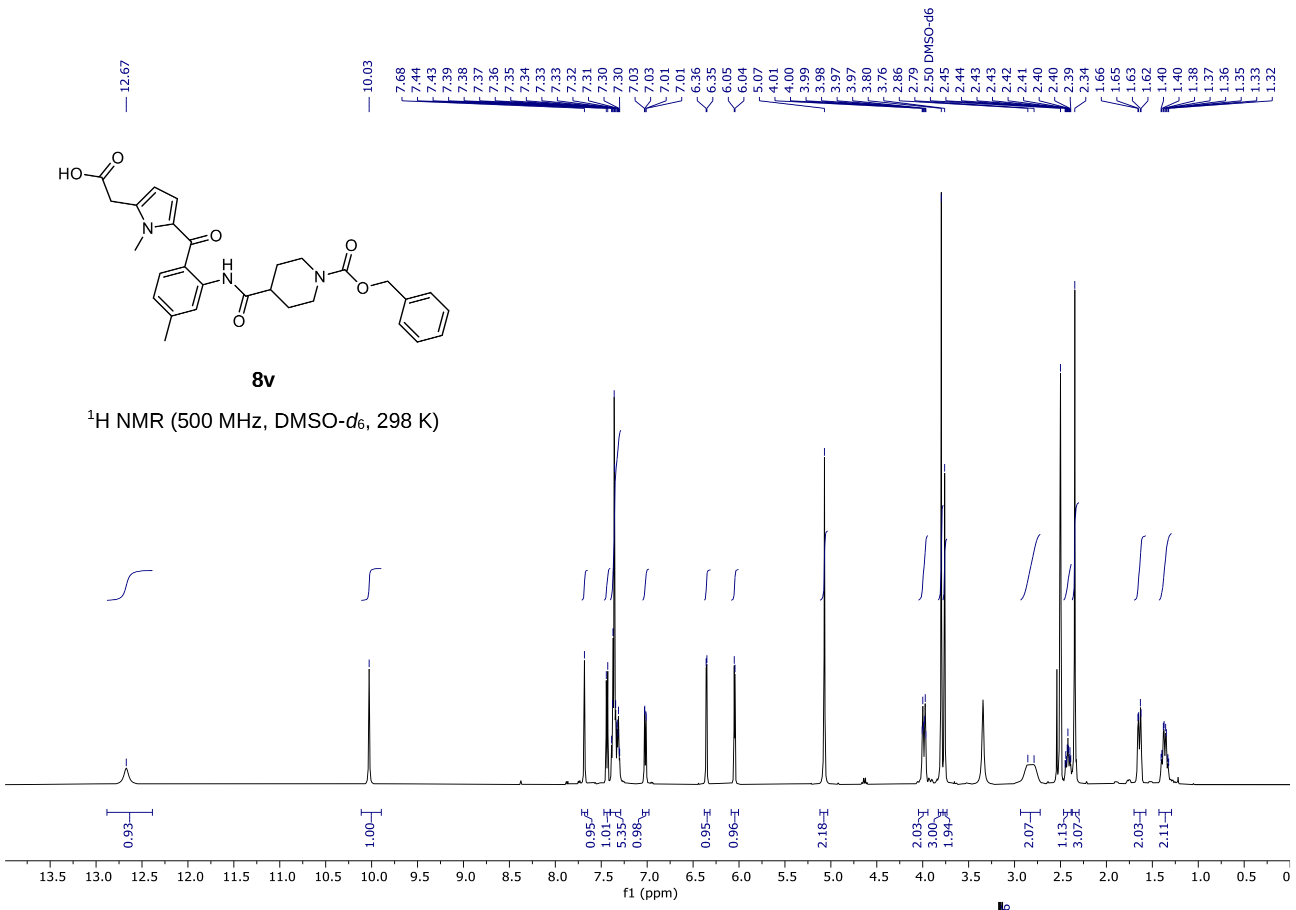

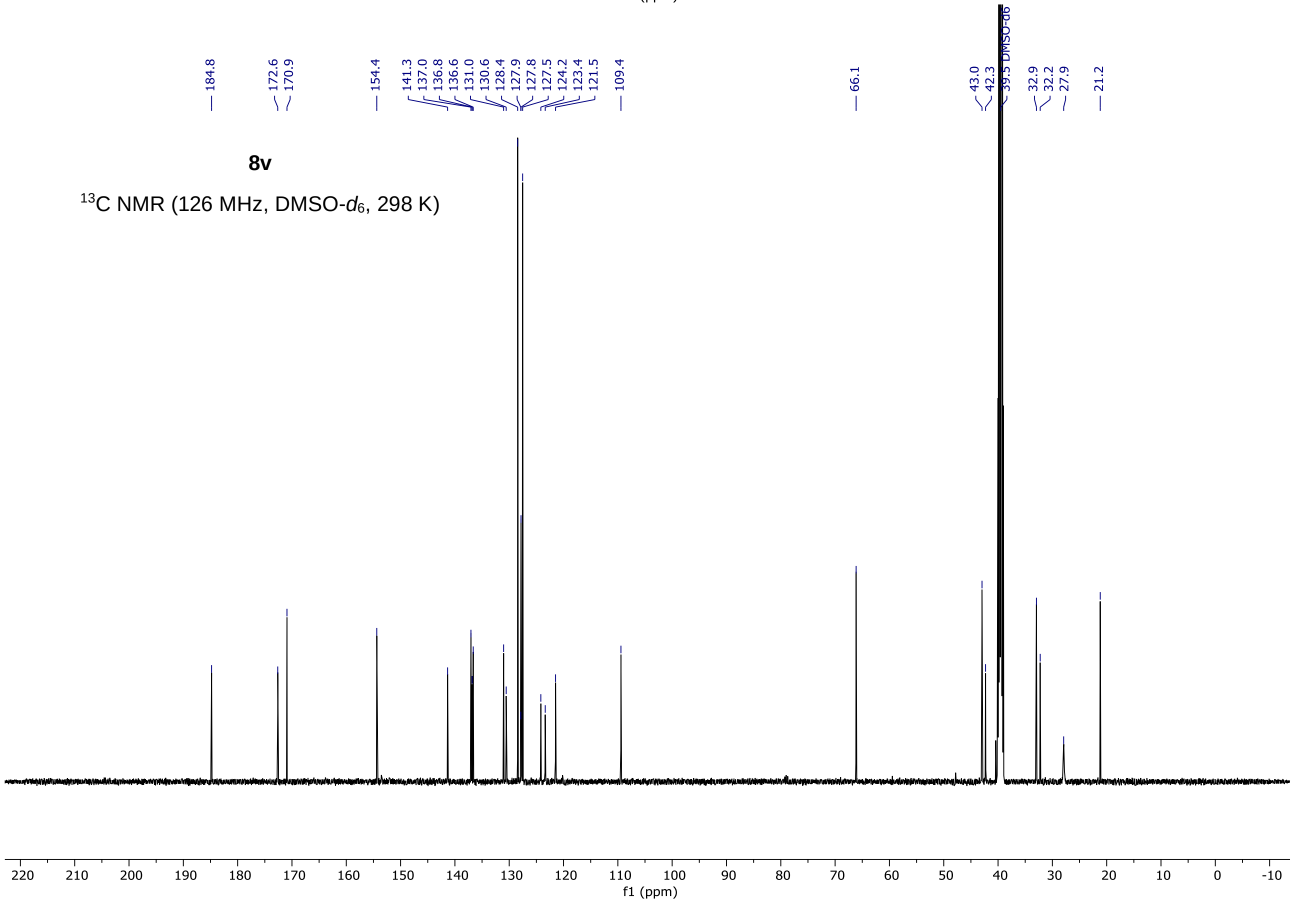

Supplementary Figure 118. ^{1}H NMR (top) and ^{13}C NMR (bottom) spectra of compound **8v**. Frequency, temperature and solvent of measurement are indicated on each spectra.

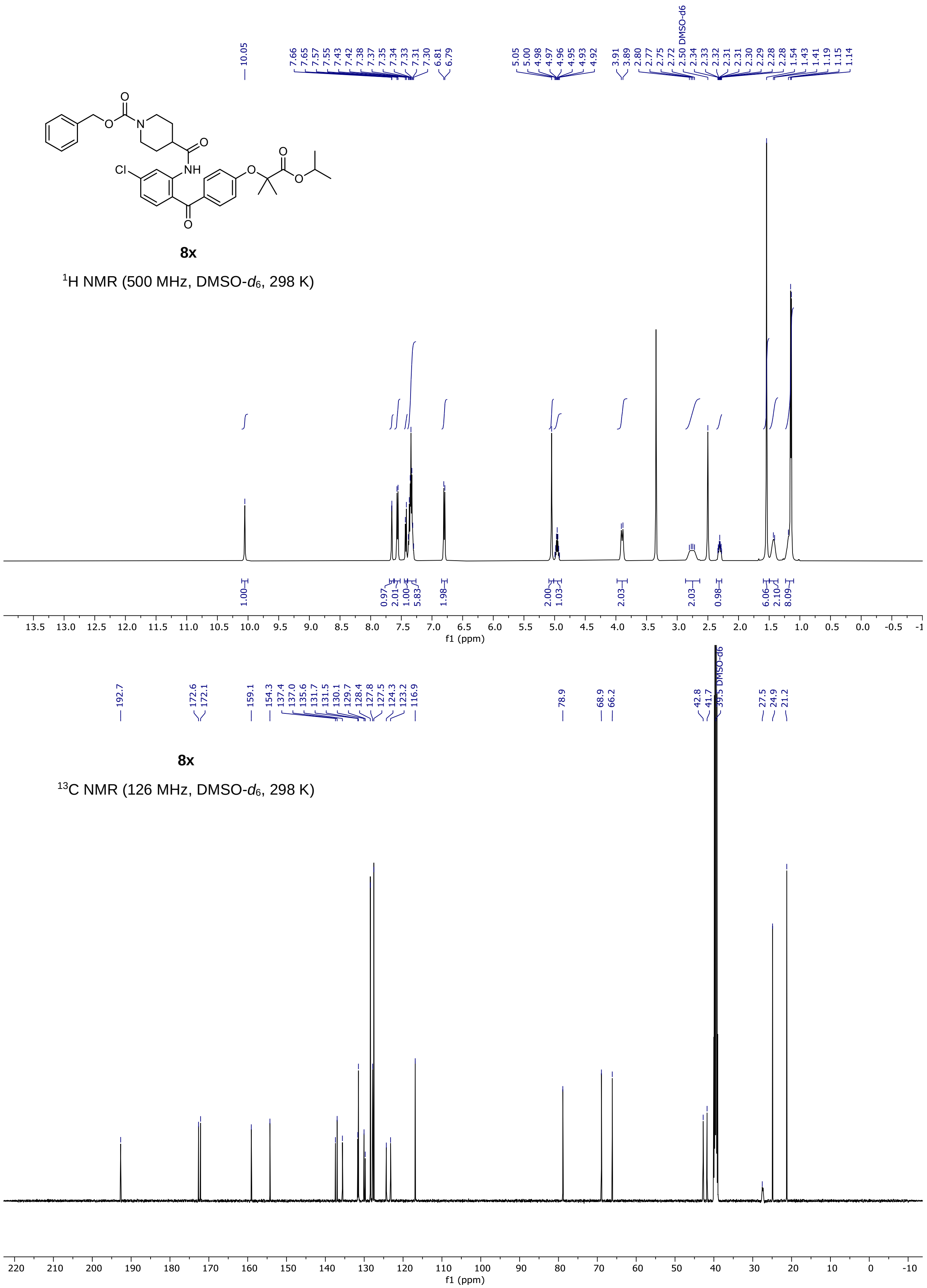

Supplementary Figure 119. ^{1}H NMR (top) and ^{13}C NMR (bottom) spectra of compound **8x**. Frequency, temperature and solvent of measurement are indicated on each spectra.

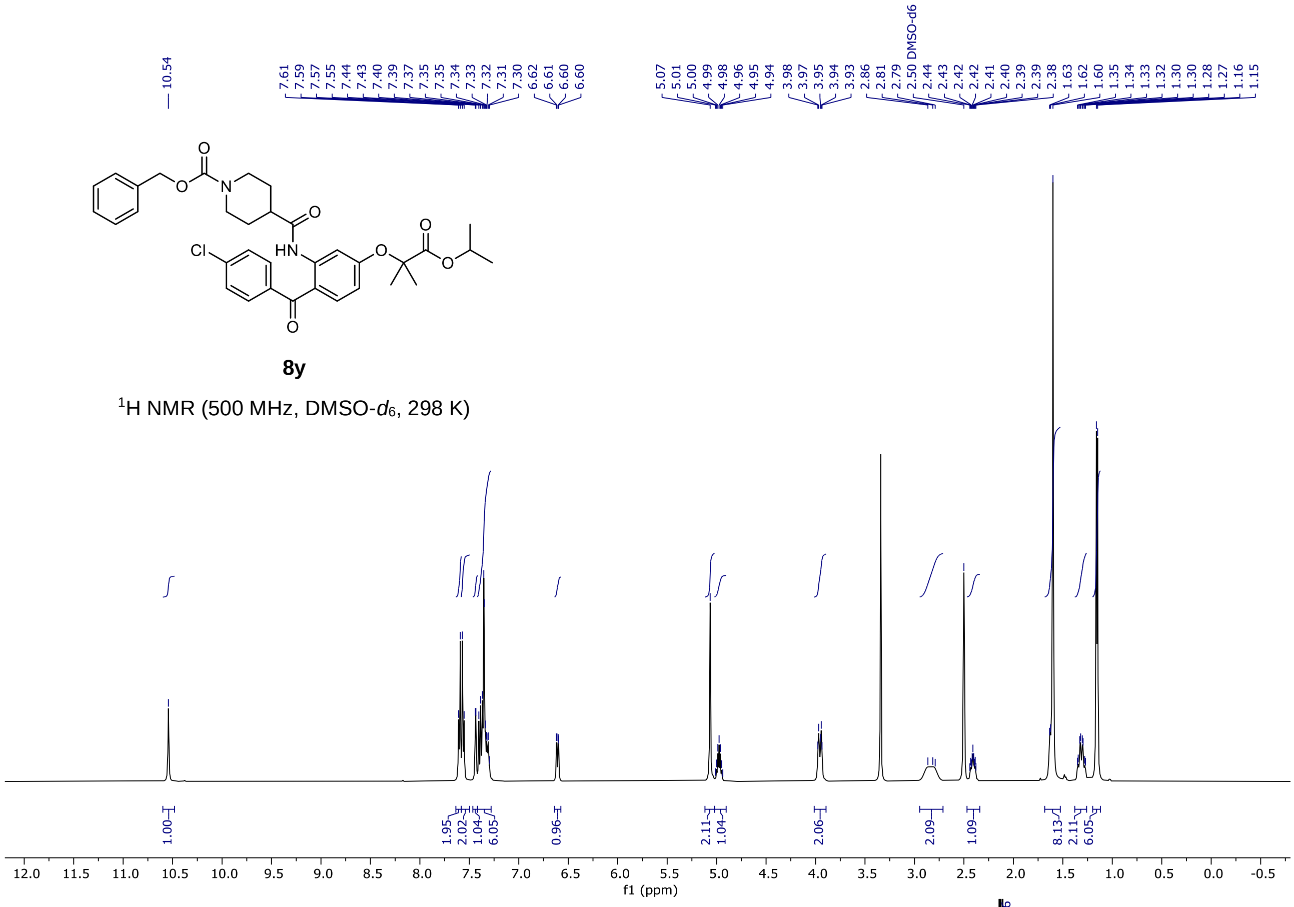

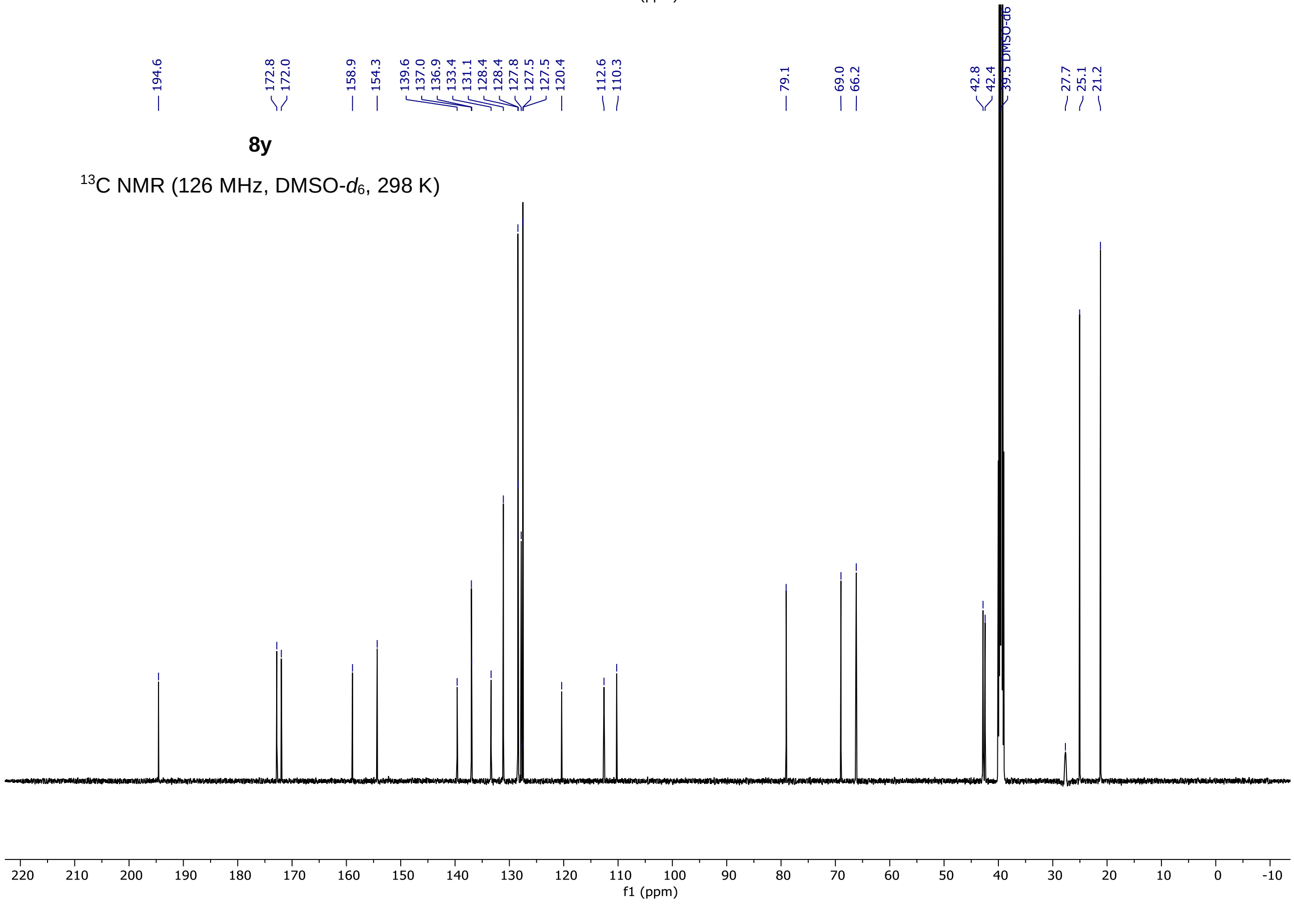

Supplementary Figure 120. ^{1}H NMR (top) and ^{13}C NMR (bottom) spectra of compound **8y**. Frequency, temperature and solvent of measurement are indicated on each spectra.

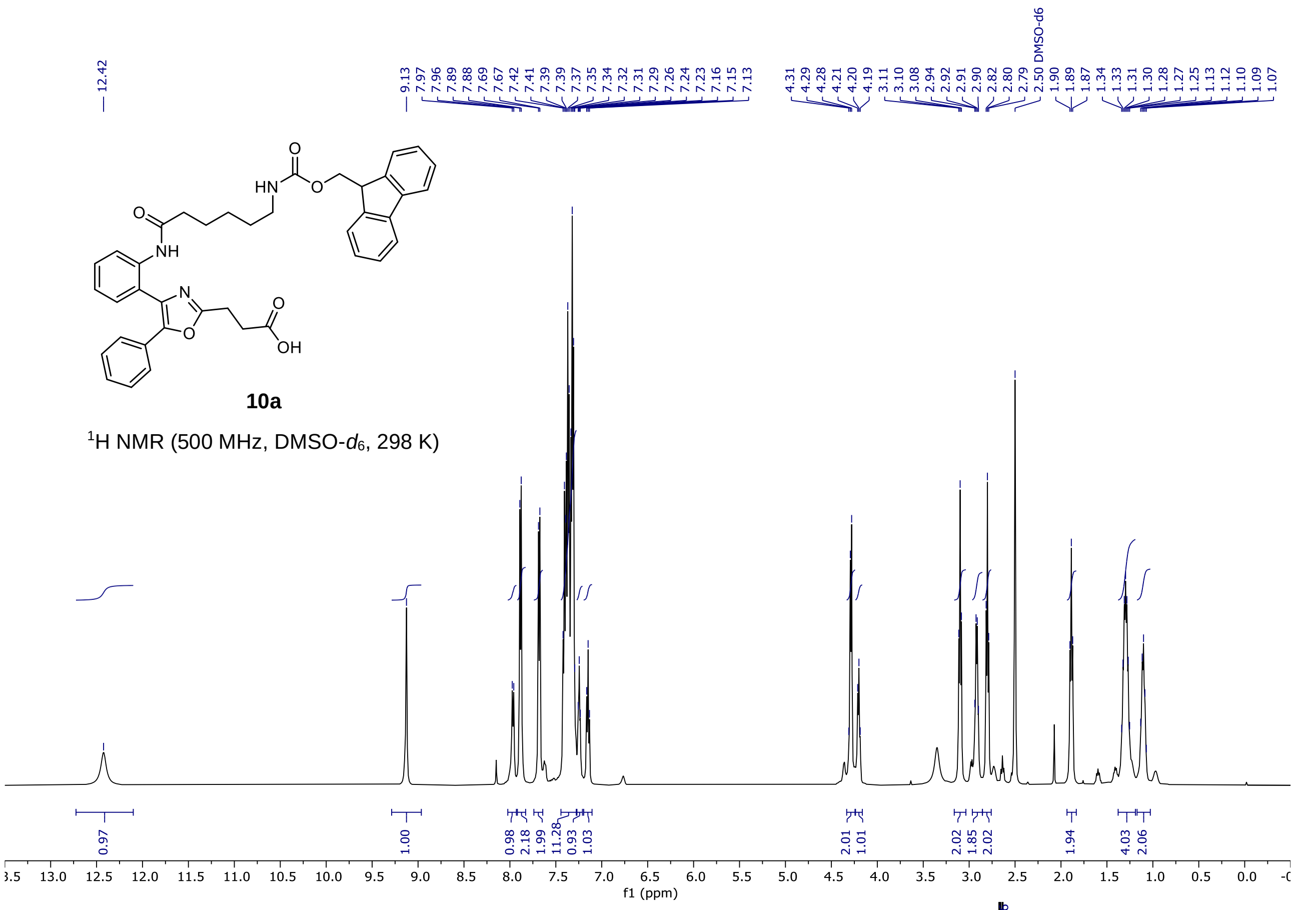

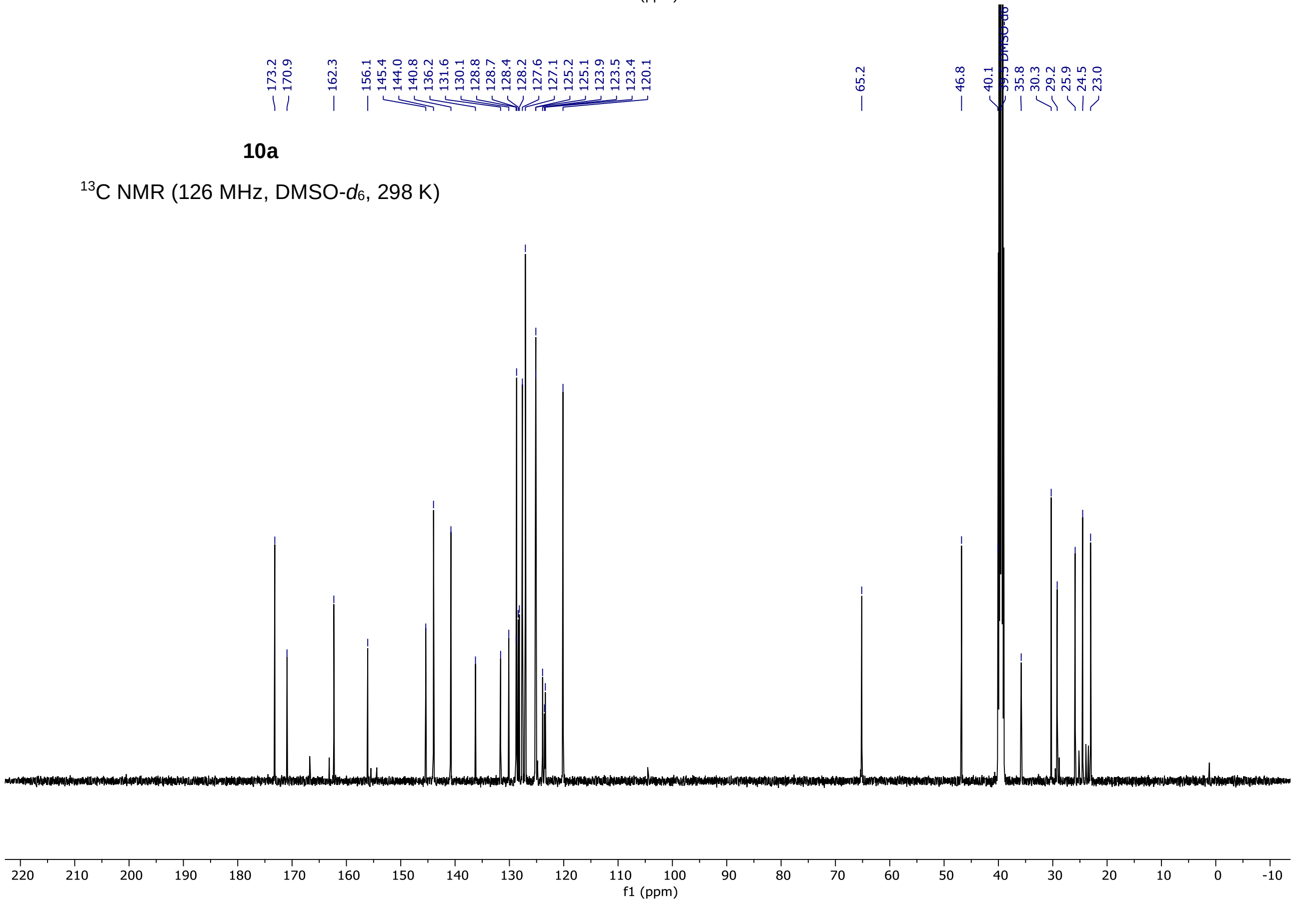

Supplementary Figure 121. ^{1}H NMR (top) and ^{13}C NMR (bottom) spectra of compound **10a**. Frequency, temperature and solvent of measurement are indicated on each spectra.

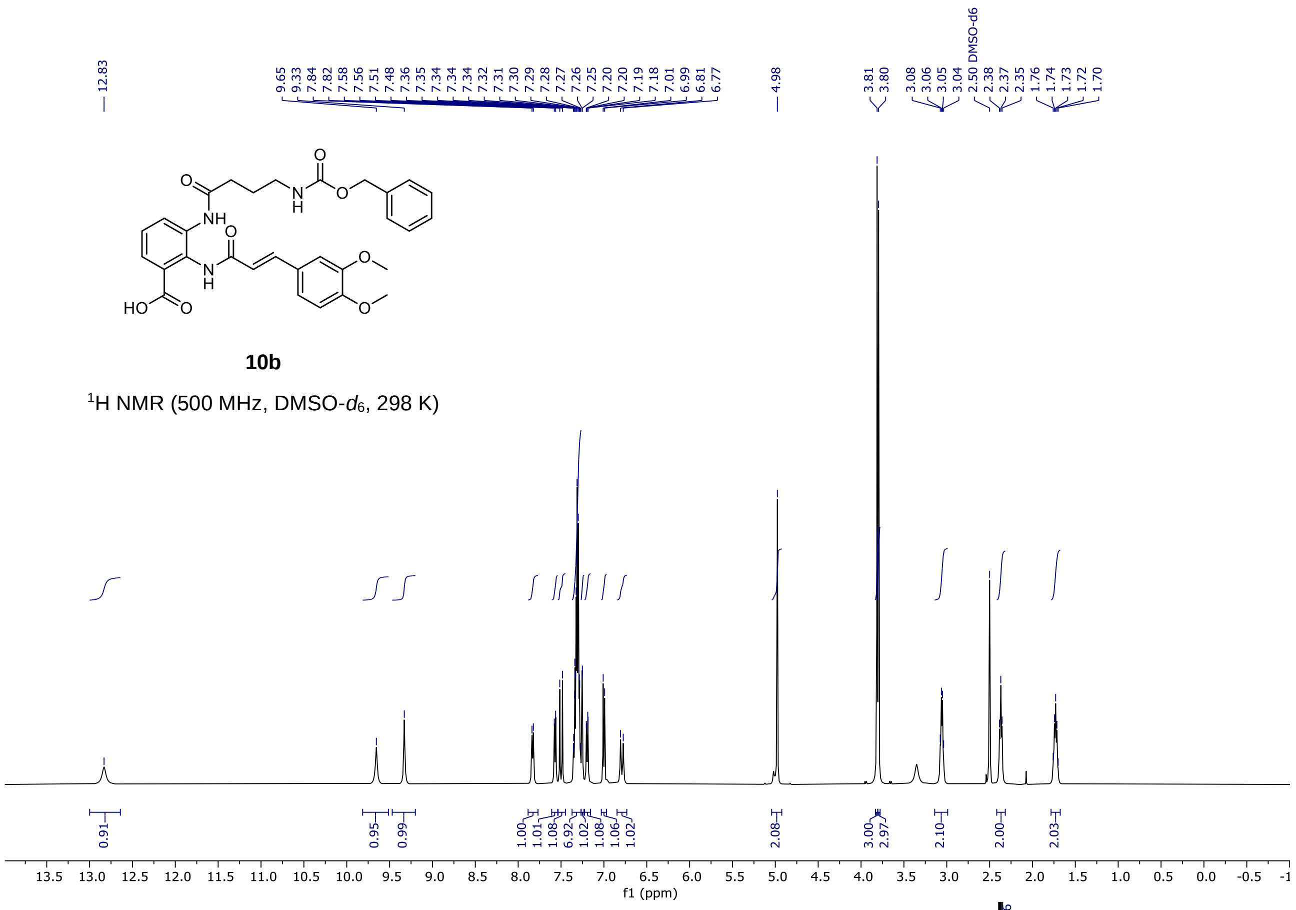

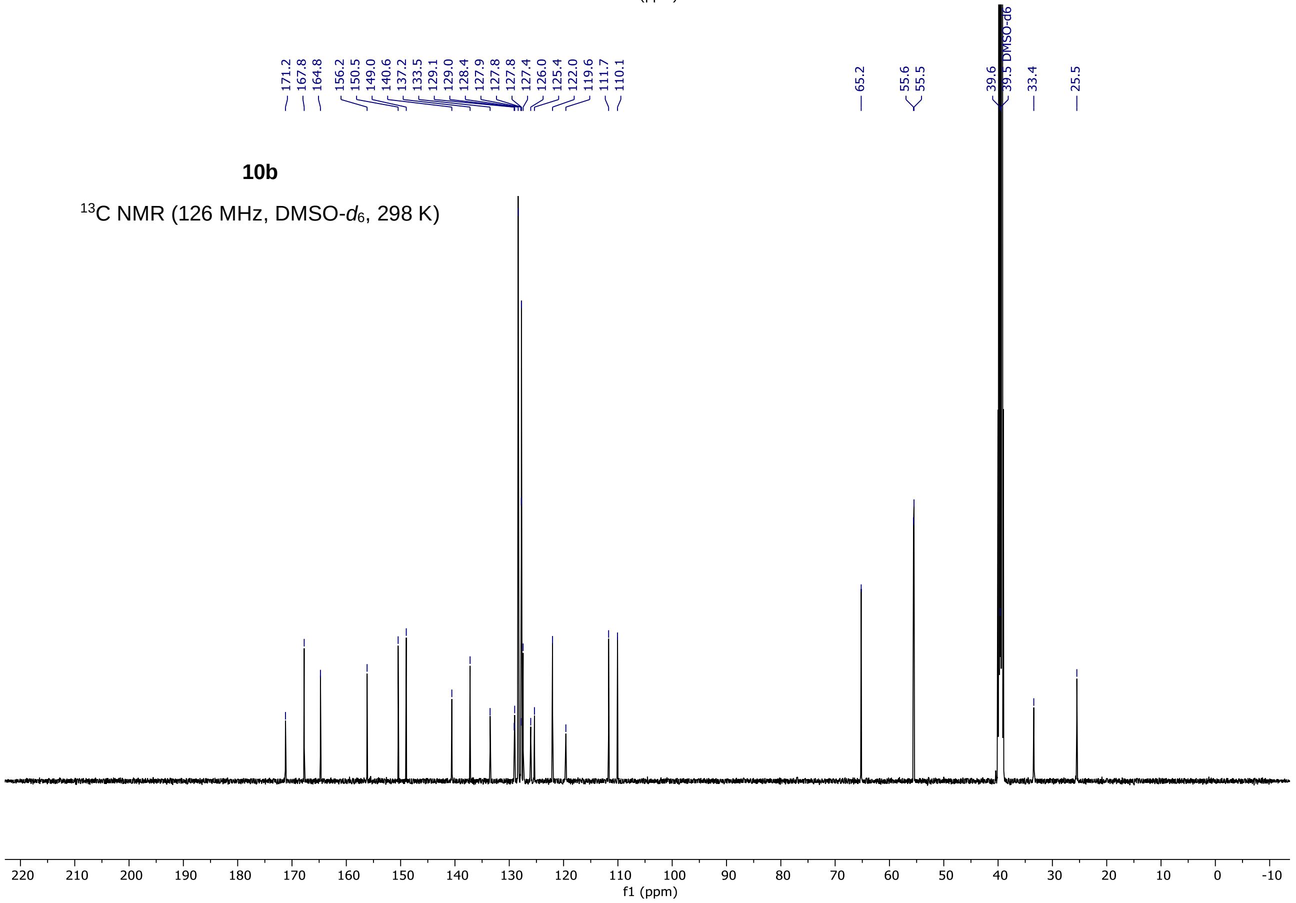

Supplementary Figure 122. ^{1}H NMR (top) and ^{13}C NMR (bottom) spectra of compound **10b**. Frequency, temperature and solvent of measurement are indicated on each spectra.

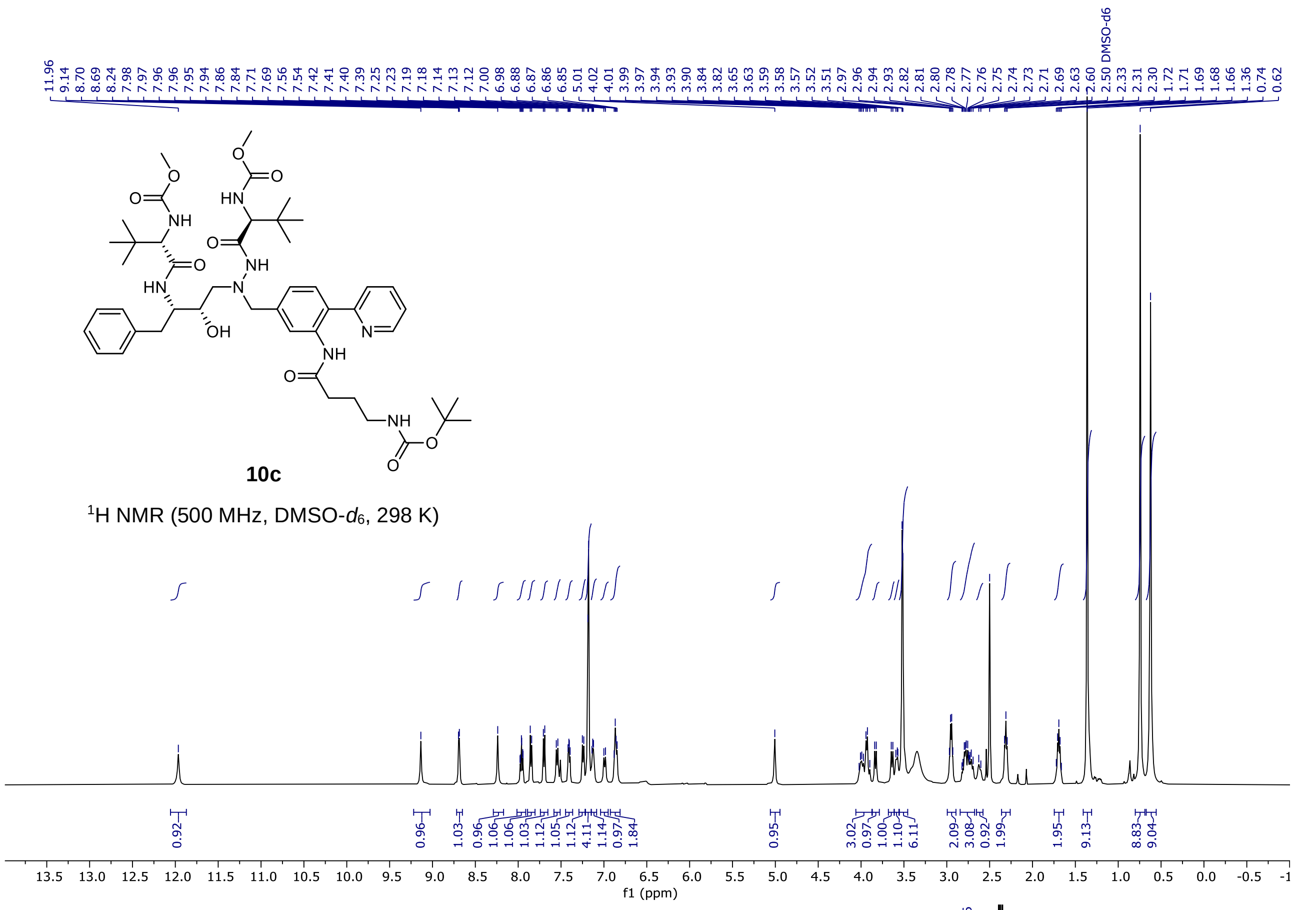

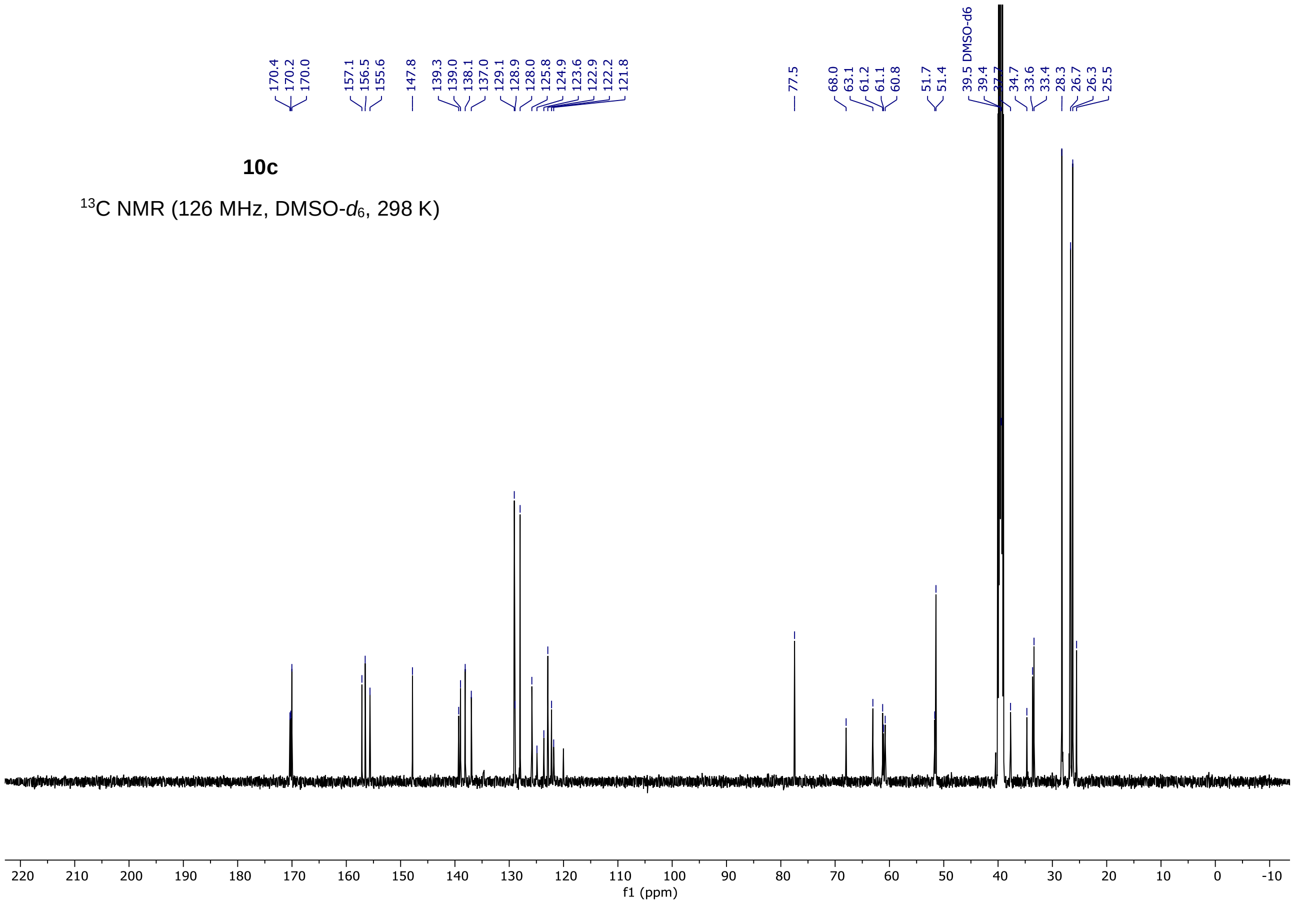

Supplementary Figure 123. ^{1}H NMR (top) and ^{13}C NMR (bottom) spectra of compound **10c**. Frequency, temperature and solvent of measurement are indicated on each spectra.

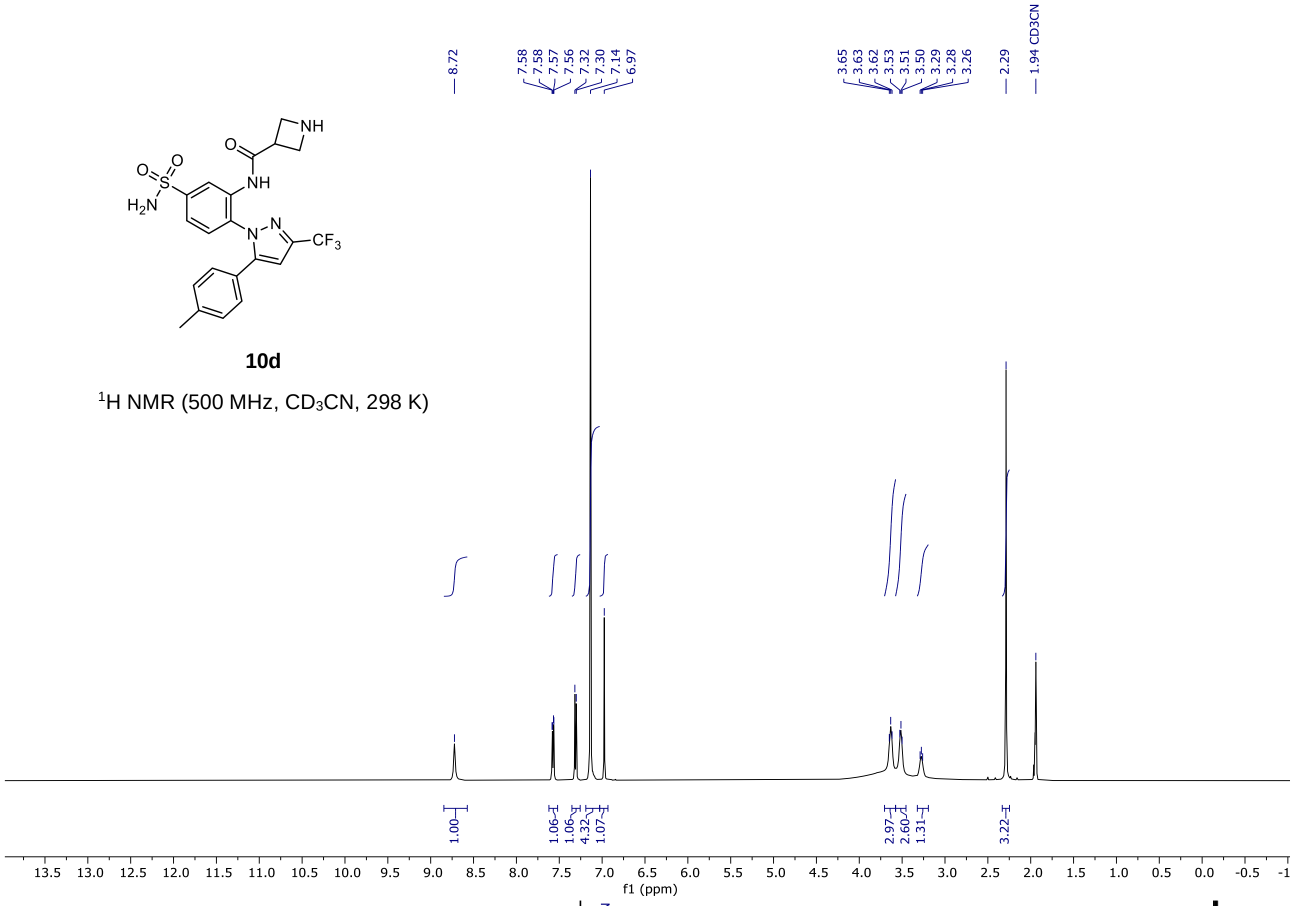

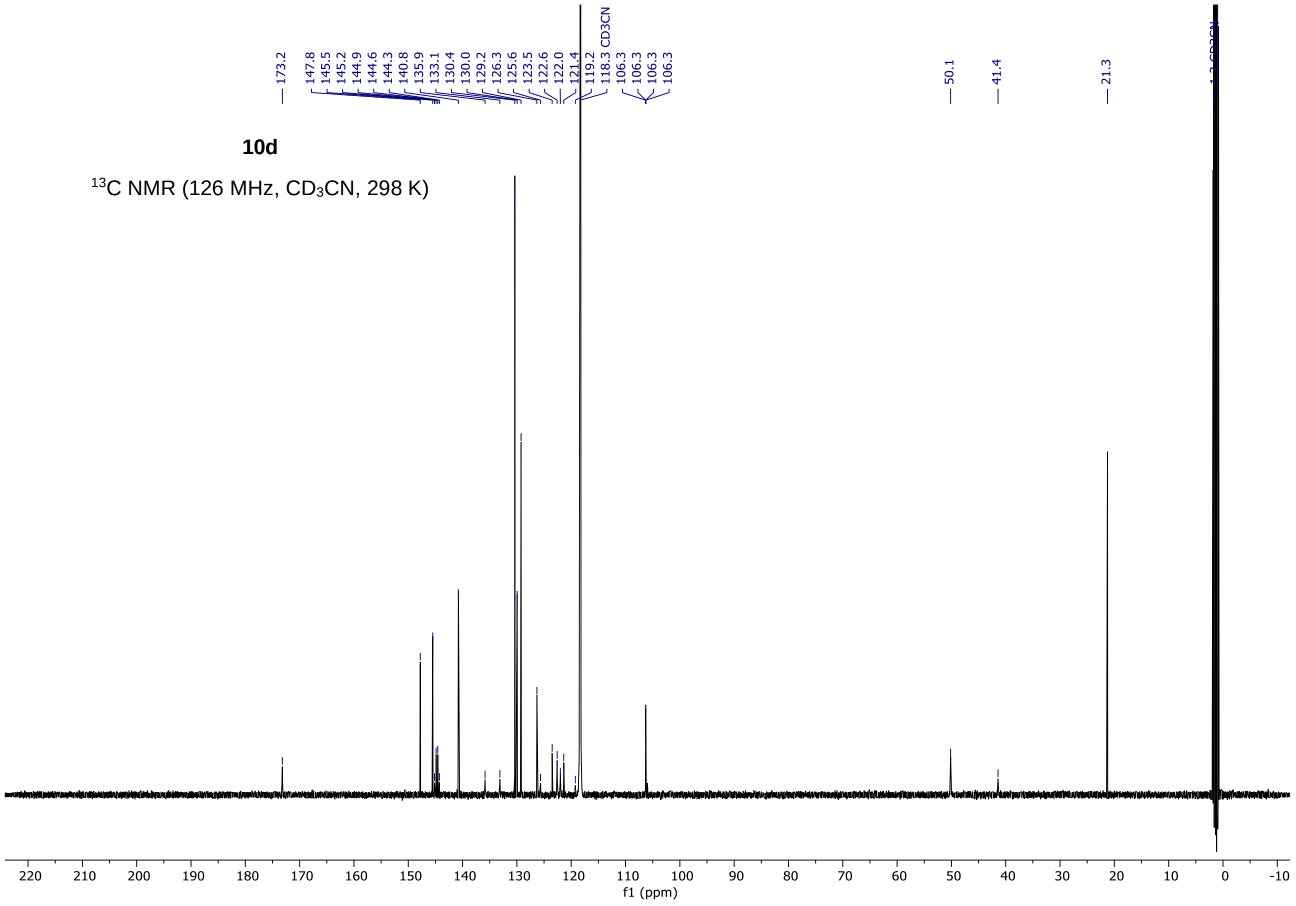

Supplementary Figure 124. ^{1}H NMR (top) and ^{13}C NMR (bottom) spectra of compound **10d**. Frequency, temperature and solvent of measurement are indicated on each spectra.

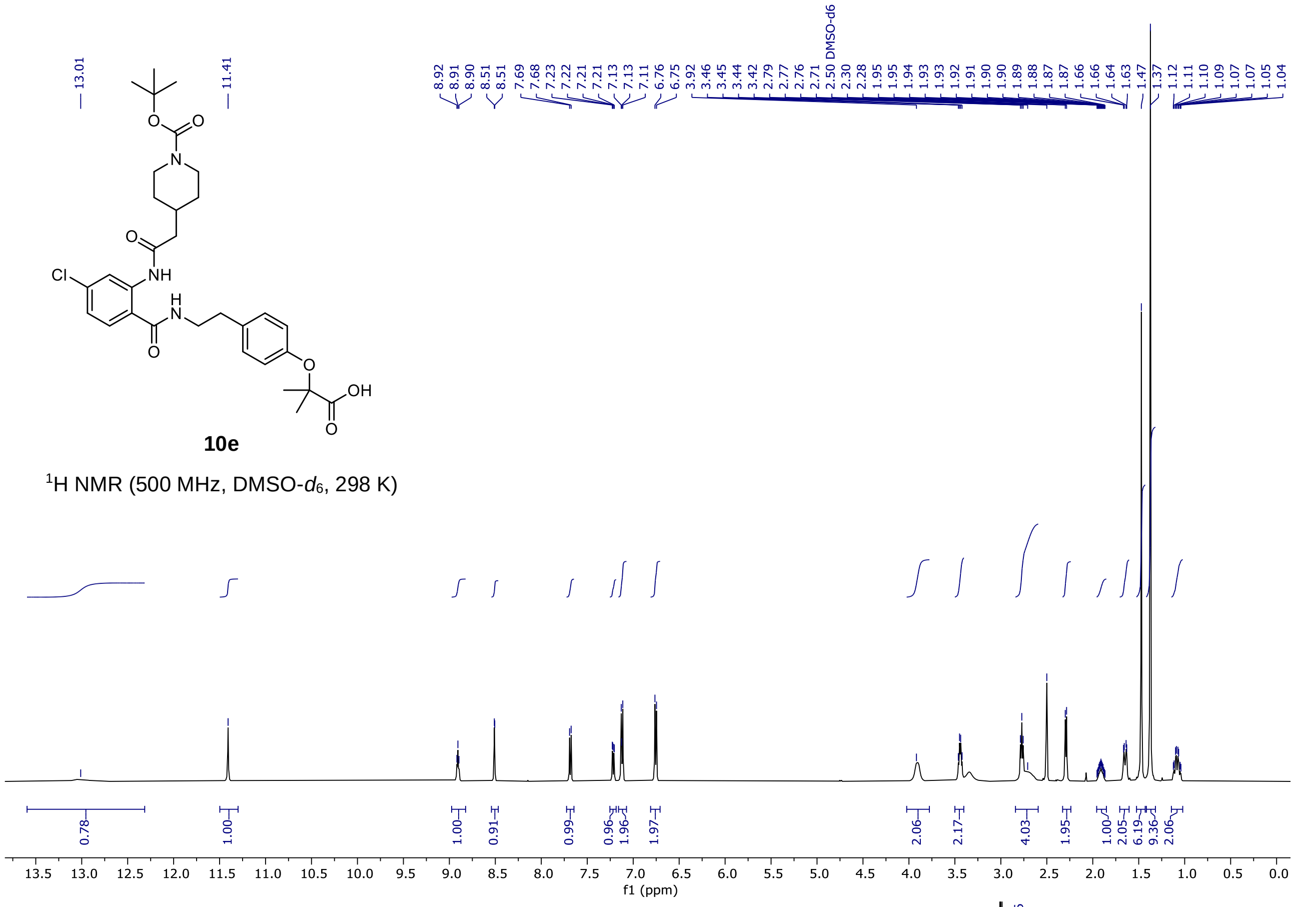

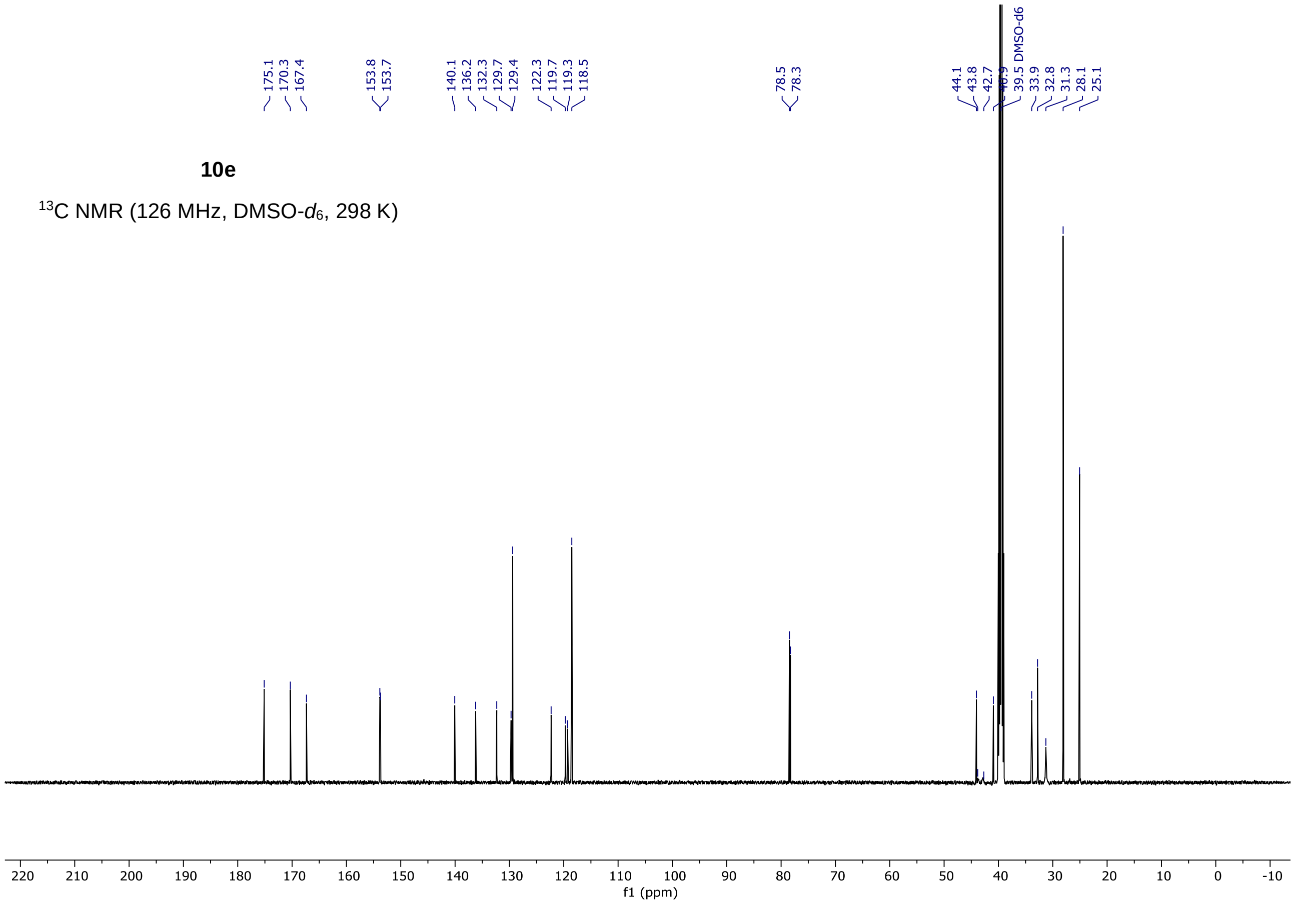

Supplementary Figure 125. ^{1}H NMR (top) and ^{13}C NMR (bottom) spectra of compound **10e**. Frequency, temperature and solvent of measurement are indicated on each spectra.

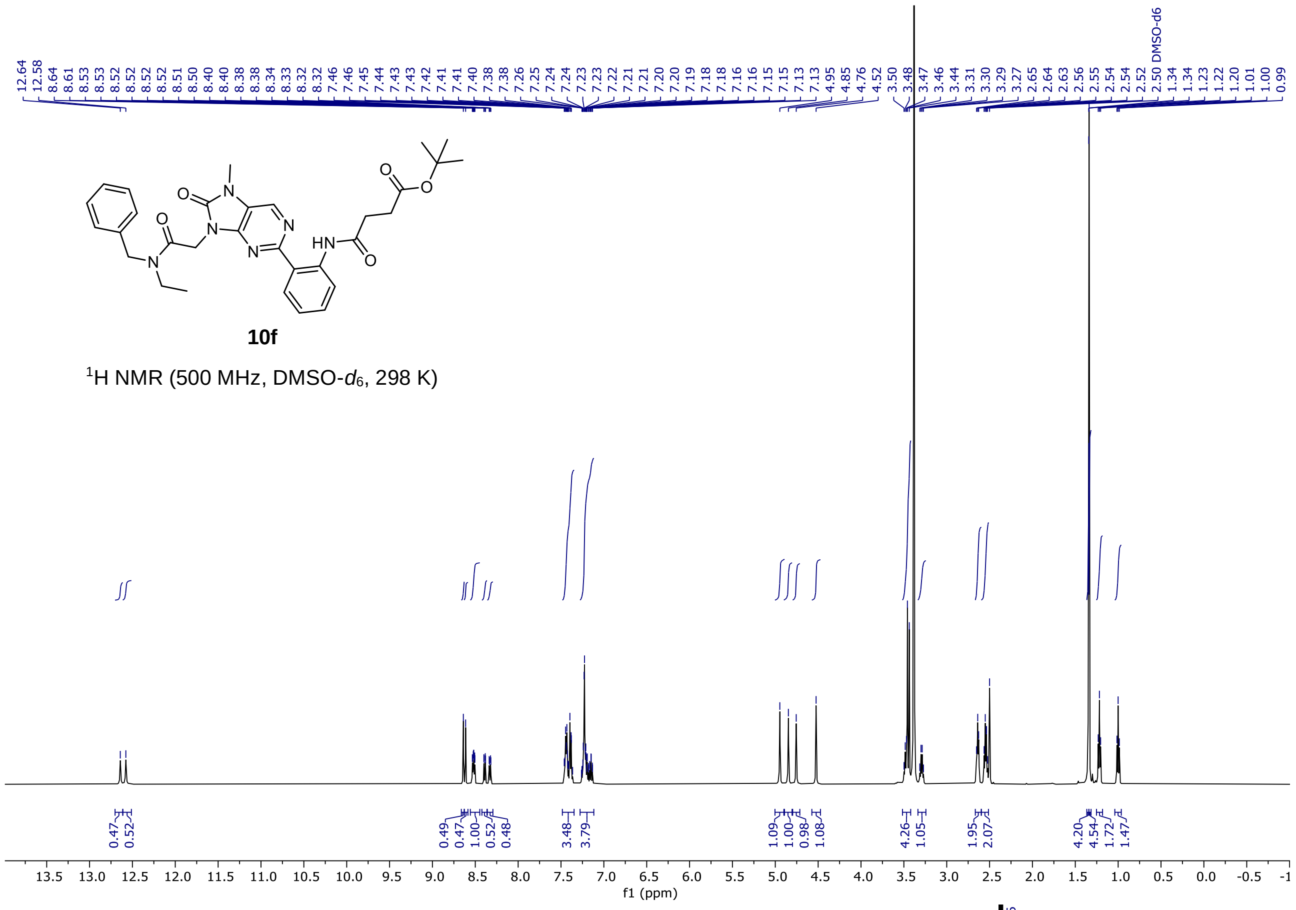

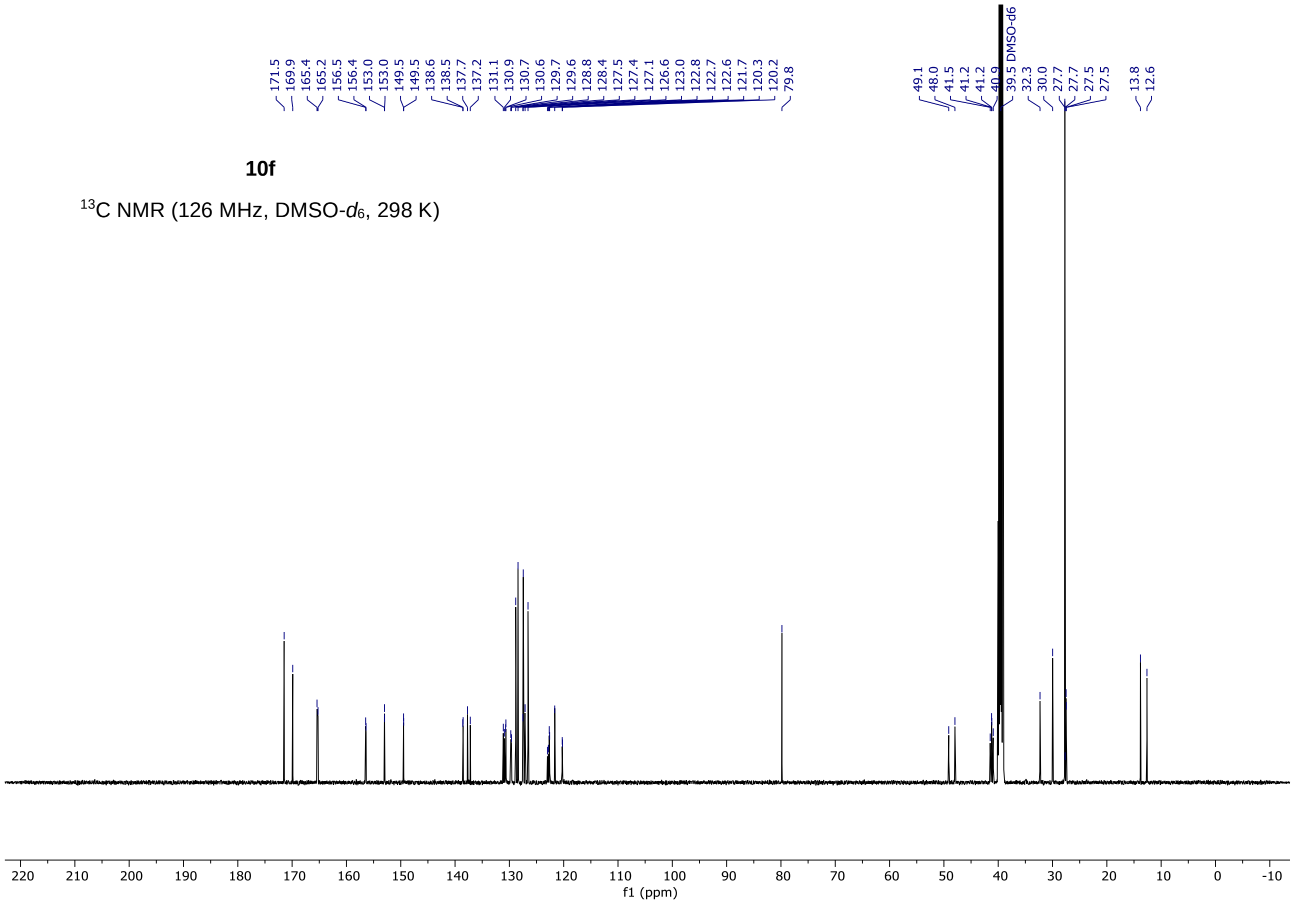

Supplementary Figure 126. ^{1}H NMR (top) and ^{13}C NMR (bottom) spectra of compound **10f**. Frequency, temperature and solvent of measurement are indicated on each spectra.

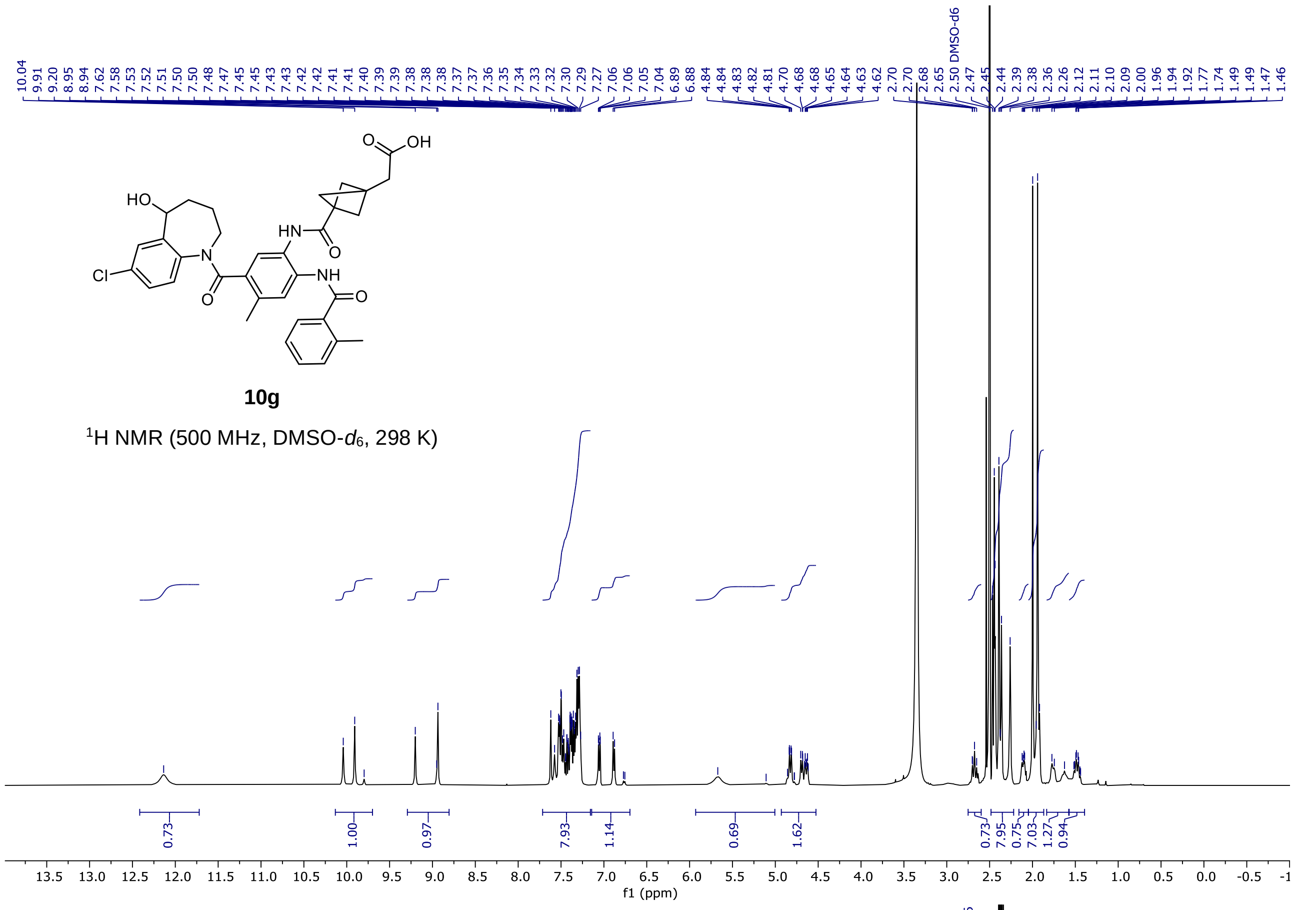

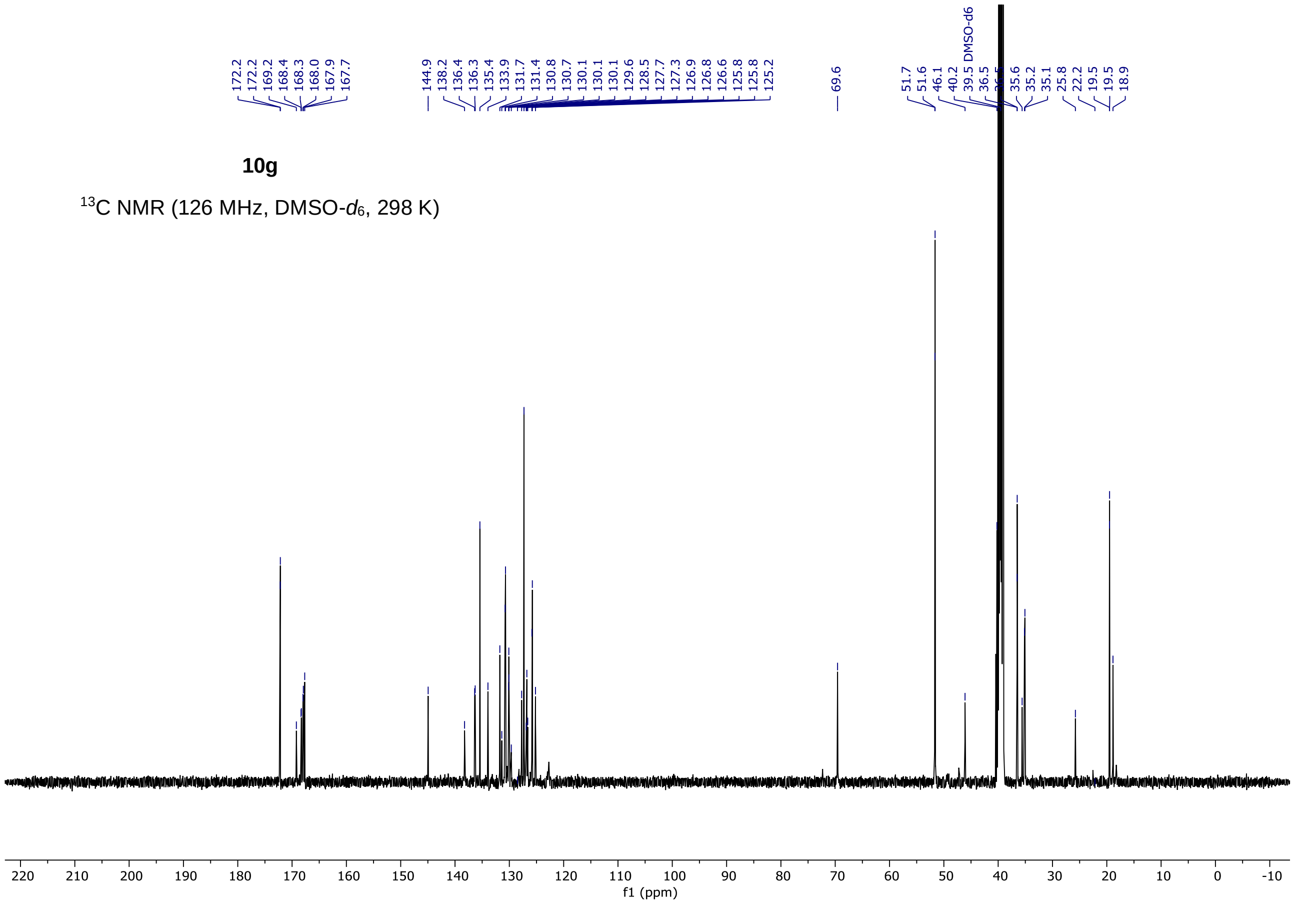

Supplementary Figure 127. ^{1}H NMR (top) and ^{13}C NMR (bottom) spectra of compound **10g**. Frequency, temperature and solvent of measurement are indicated on each spectra.

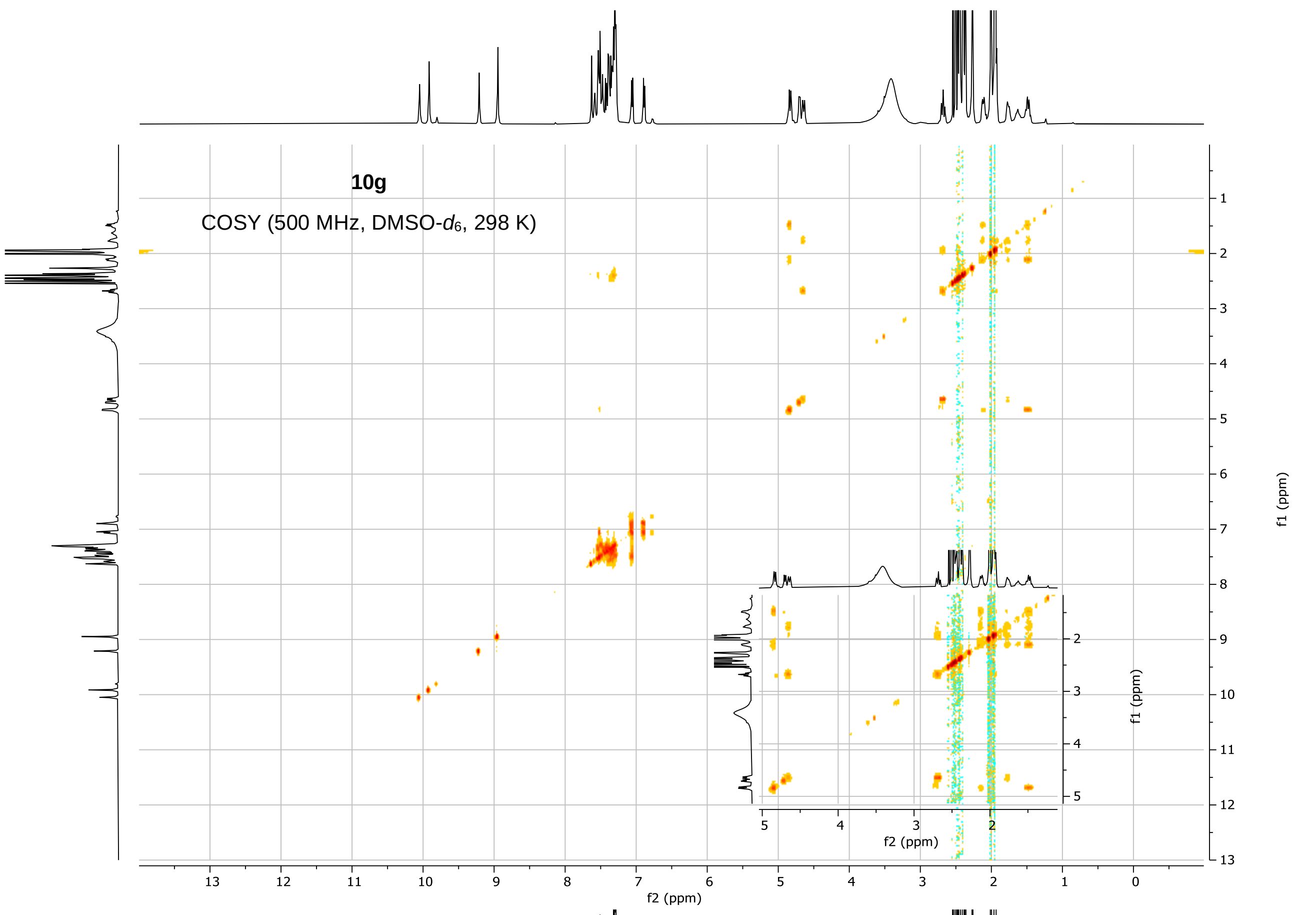

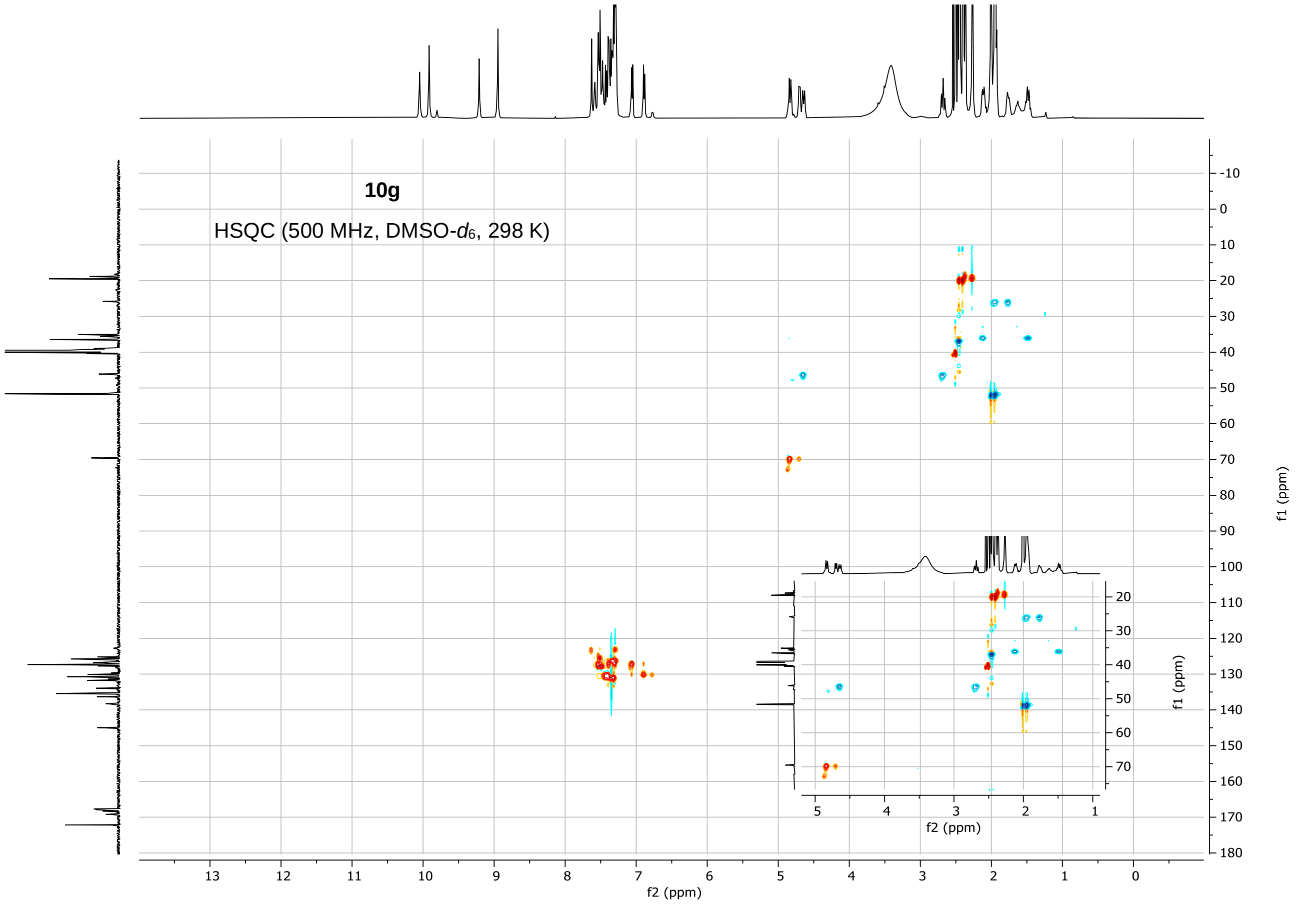

Supplementary Figure 128. COSY (top) and HSQC (bottom) spectra of compound **10g**. Frequency, temperature and solvent of measurement are indicated on each spectra.

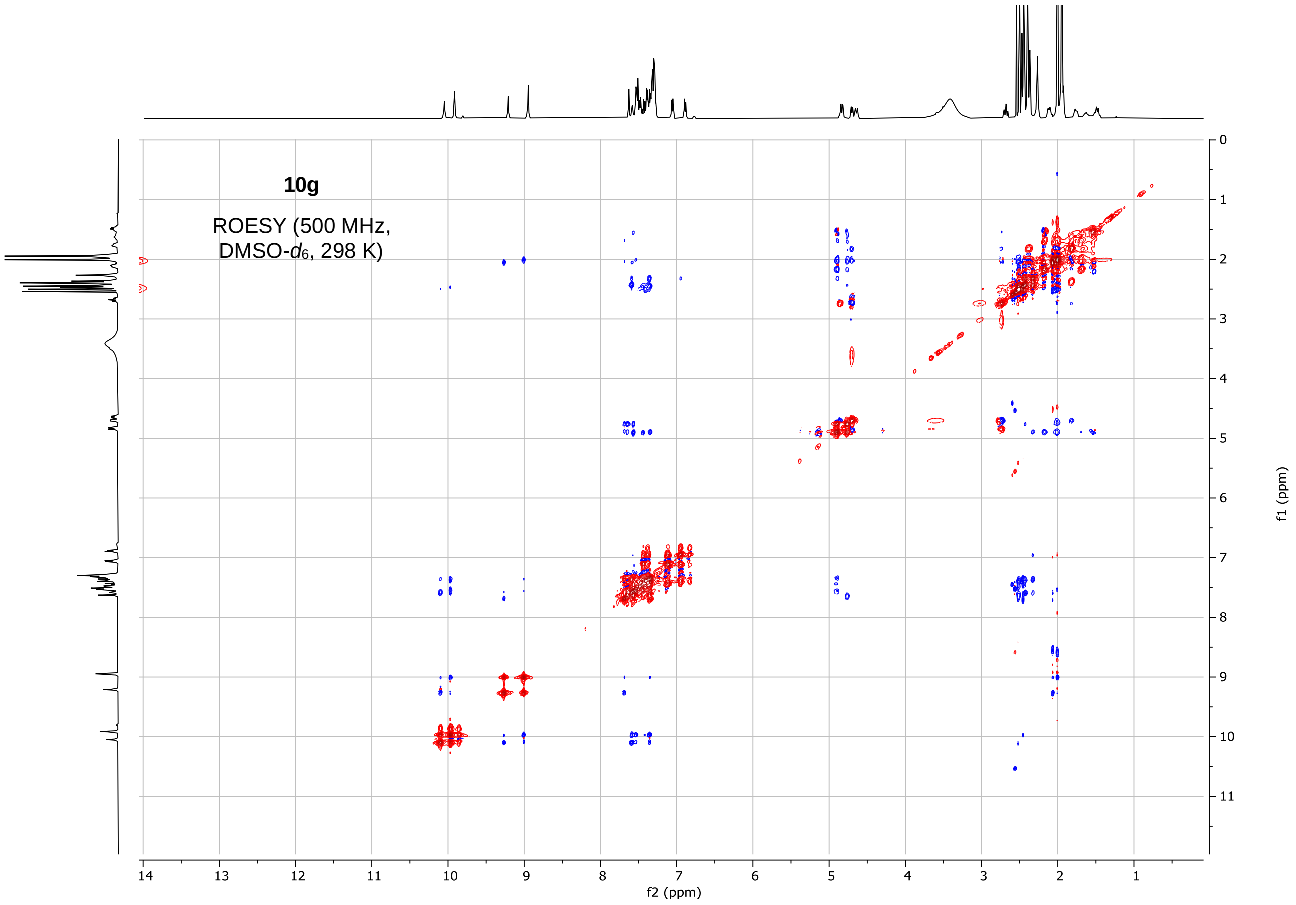

Supplementary Figure 129. ROESY spectrum of compound **10g**. Frequency, temperature and solvent of measurement are indicated on the spectrum.

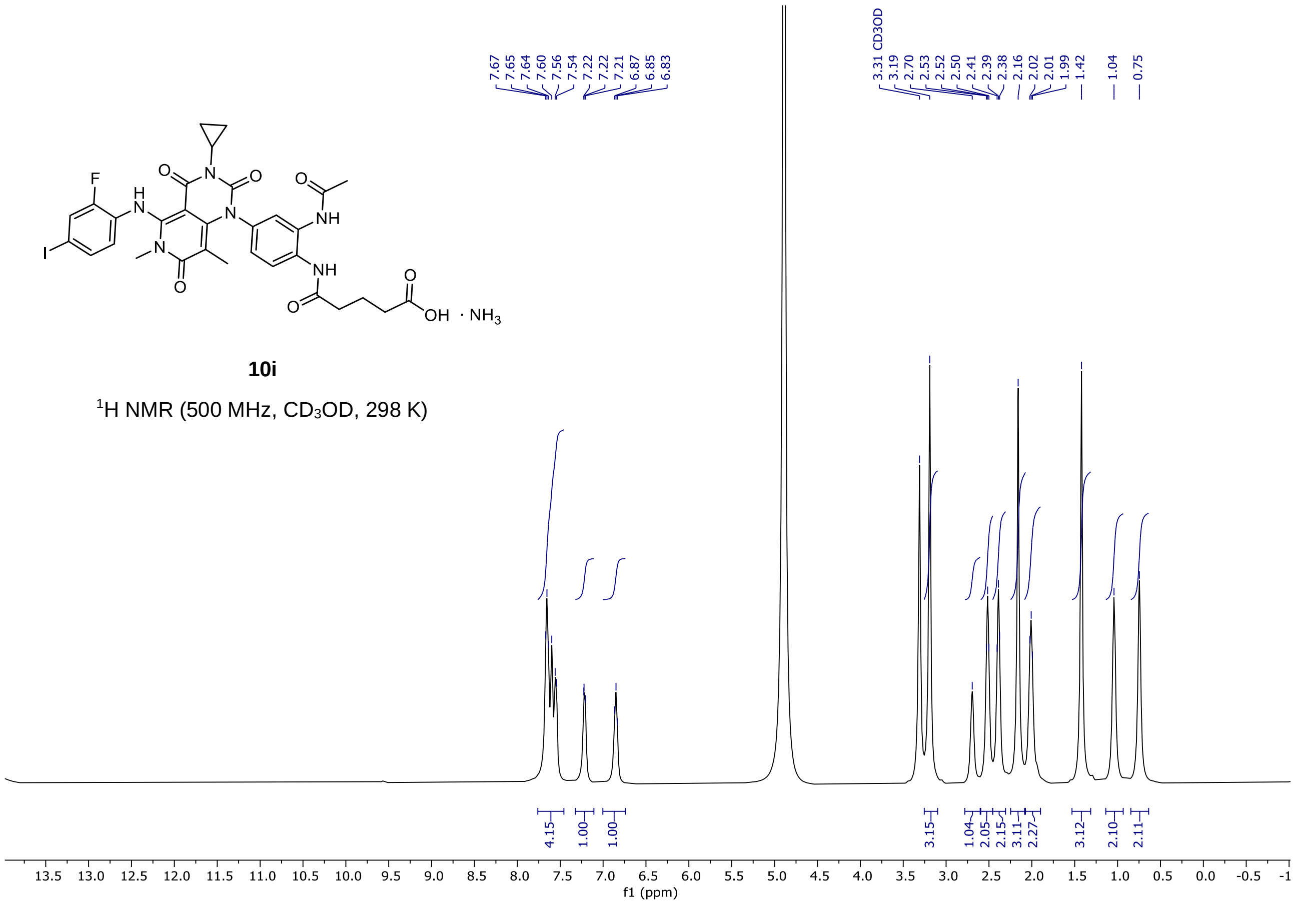

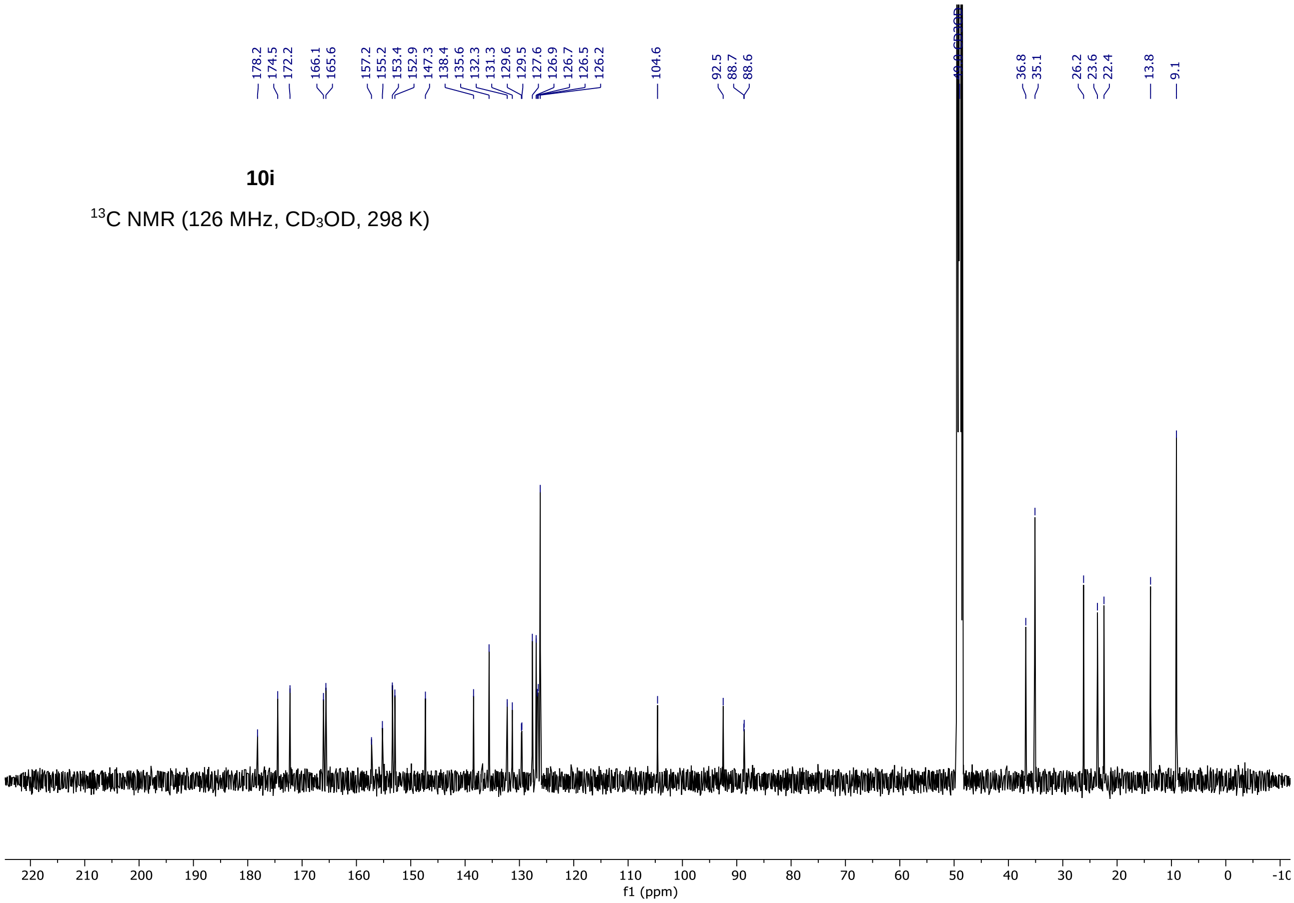

Supplementary Figure 130. ^{1}H NMR (top) and ^{13}C NMR (bottom) spectra of compound **10i**. Frequency, temperature and solvent of measurement are indicated on each spectra.

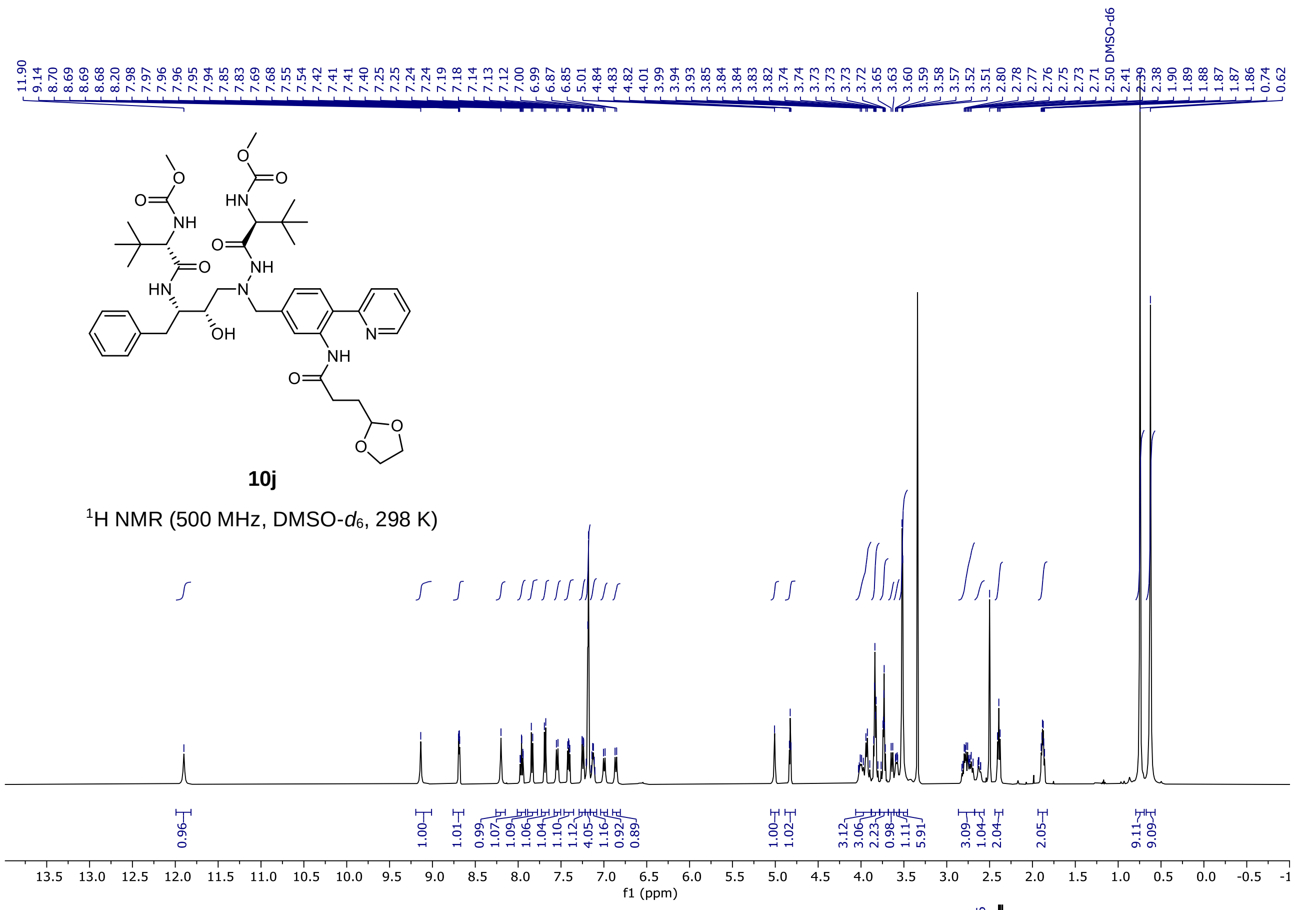

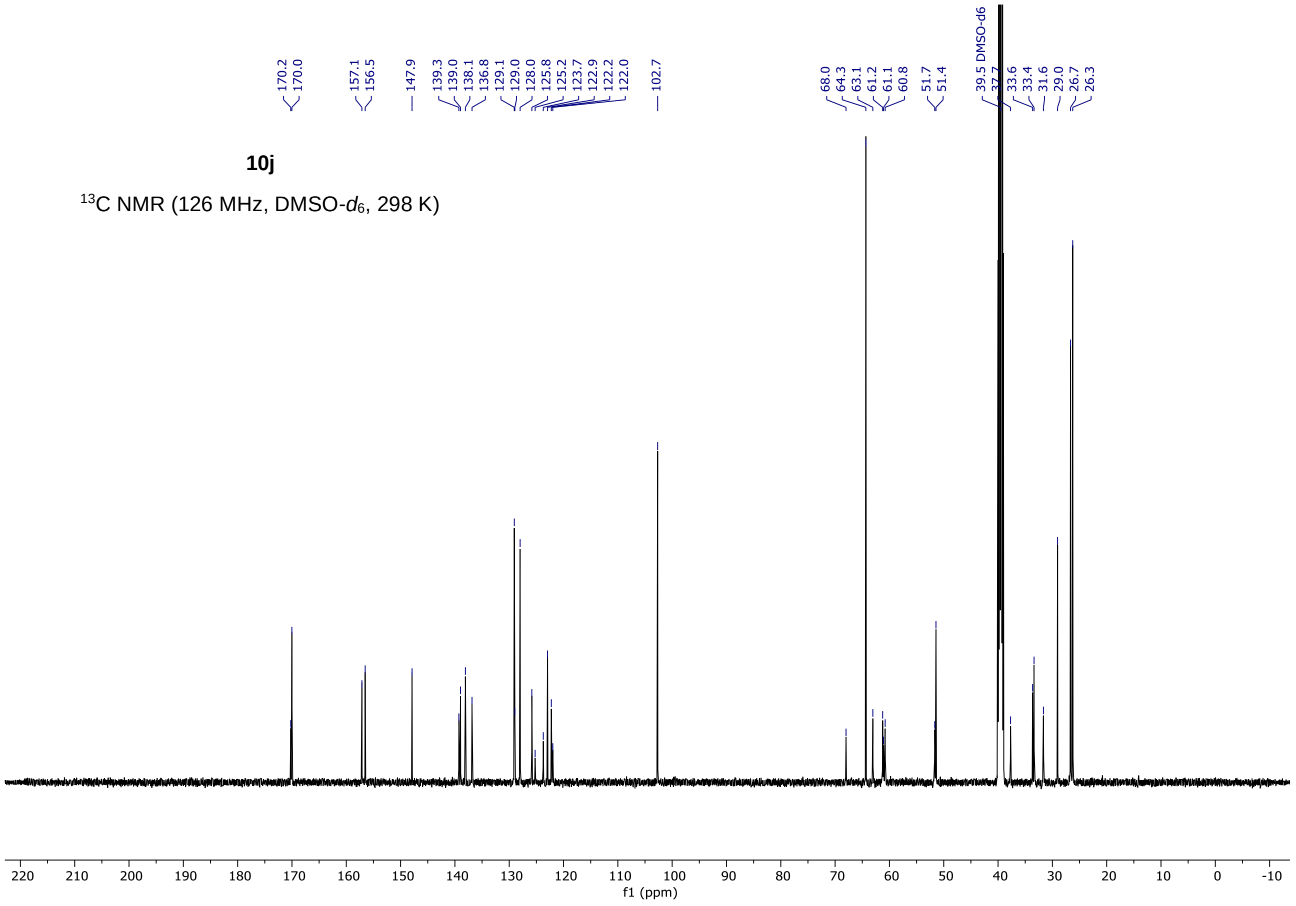

Supplementary Figure 131. ^{1}H NMR (top) and ^{13}C NMR (bottom) spectra of compound **10j**. Frequency, temperature and solvent of measurement are indicated on each spectra.

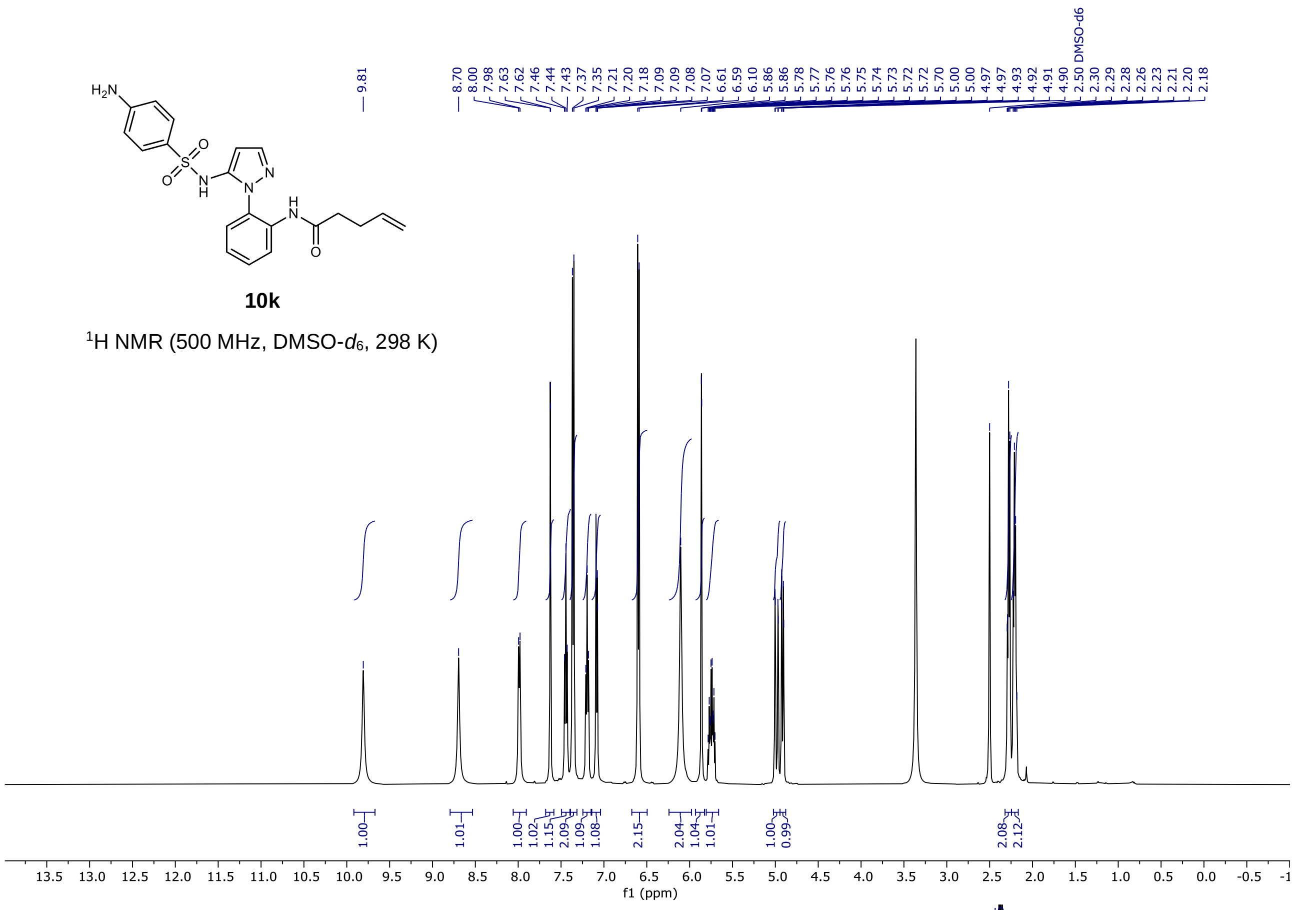

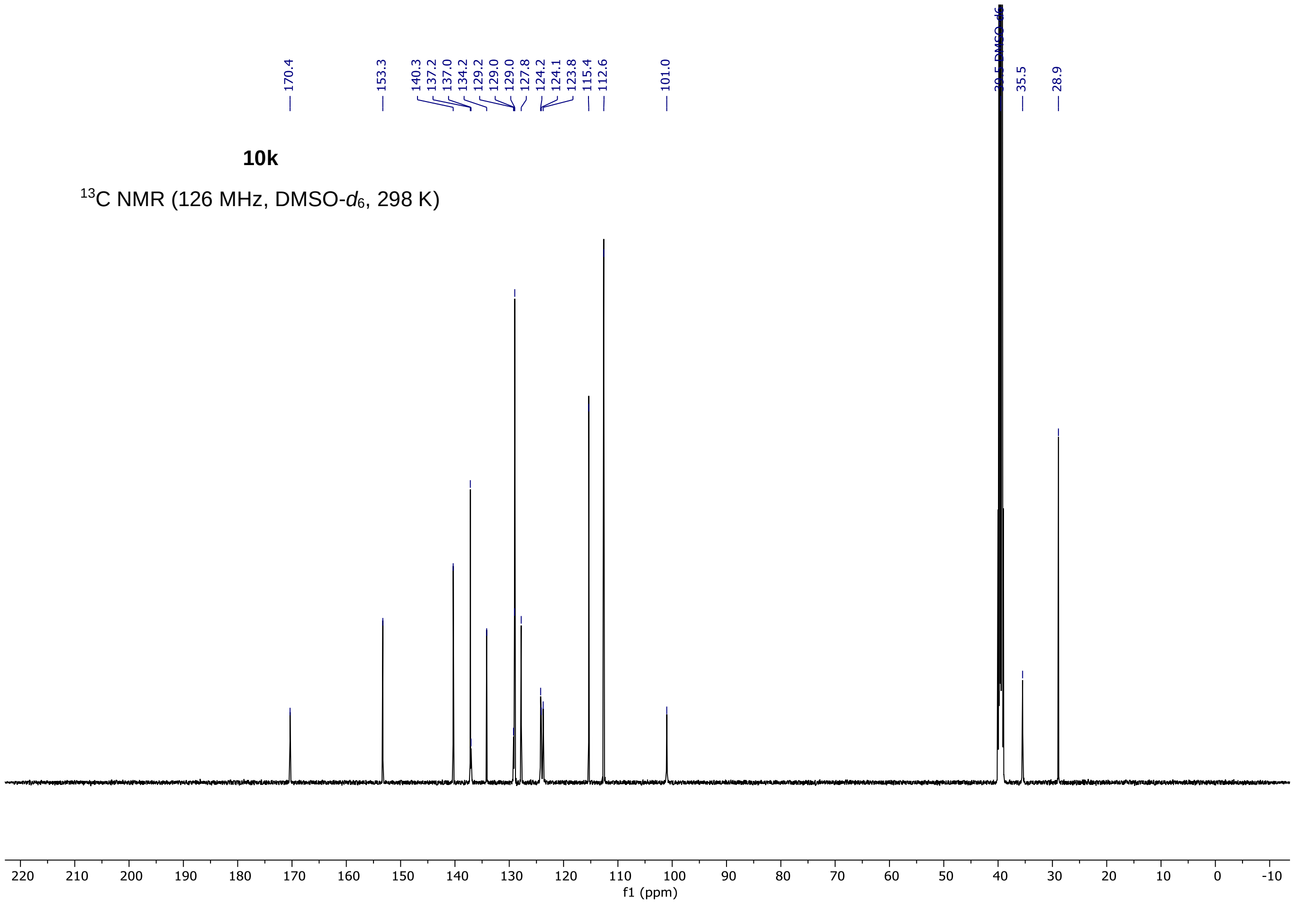

Supplementary Figure 132. ^{1}H NMR (top) and ^{13}C NMR (bottom) spectra of compound **10k**. Frequency, temperature and solvent of measurement are indicated on each spectra.

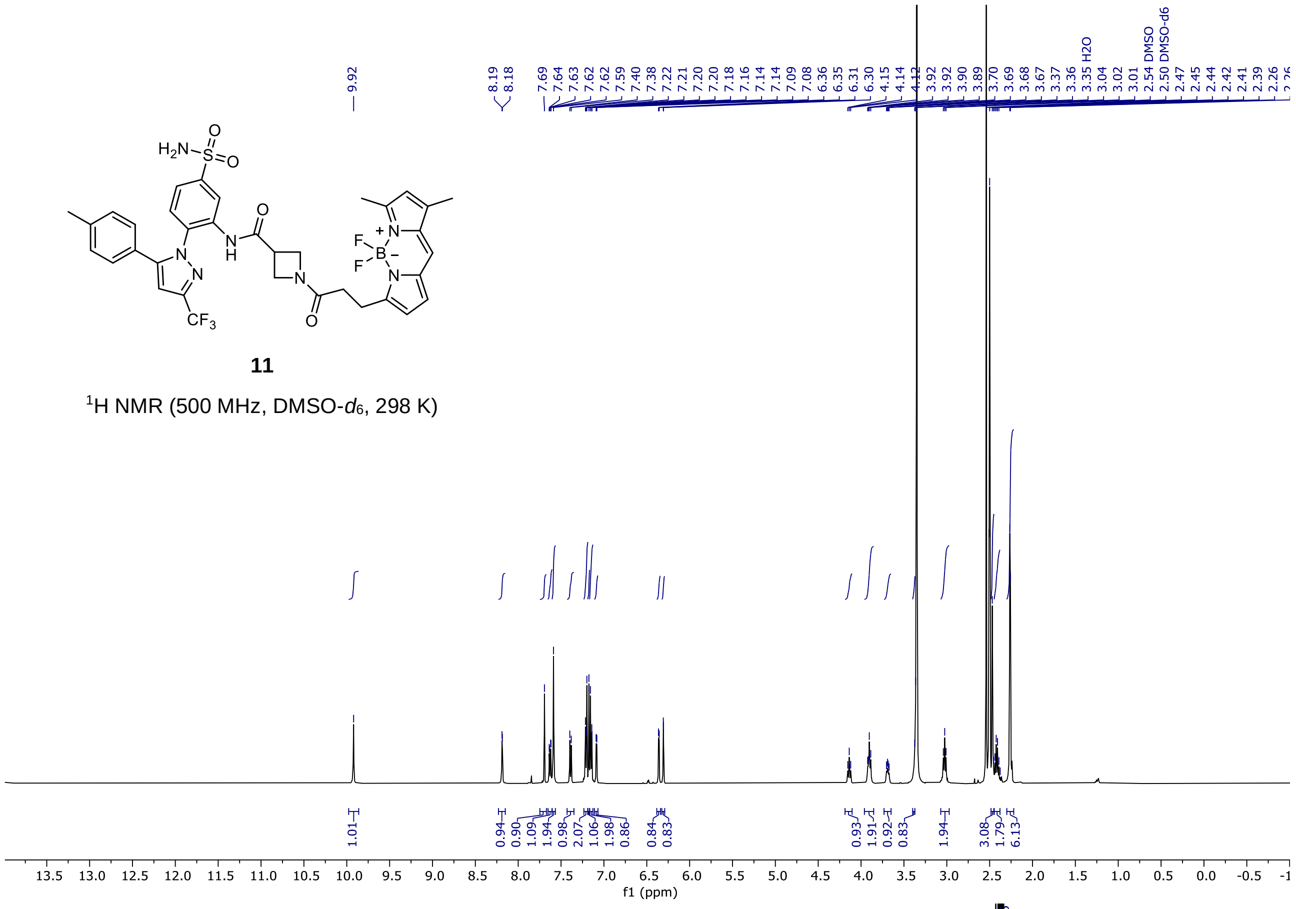

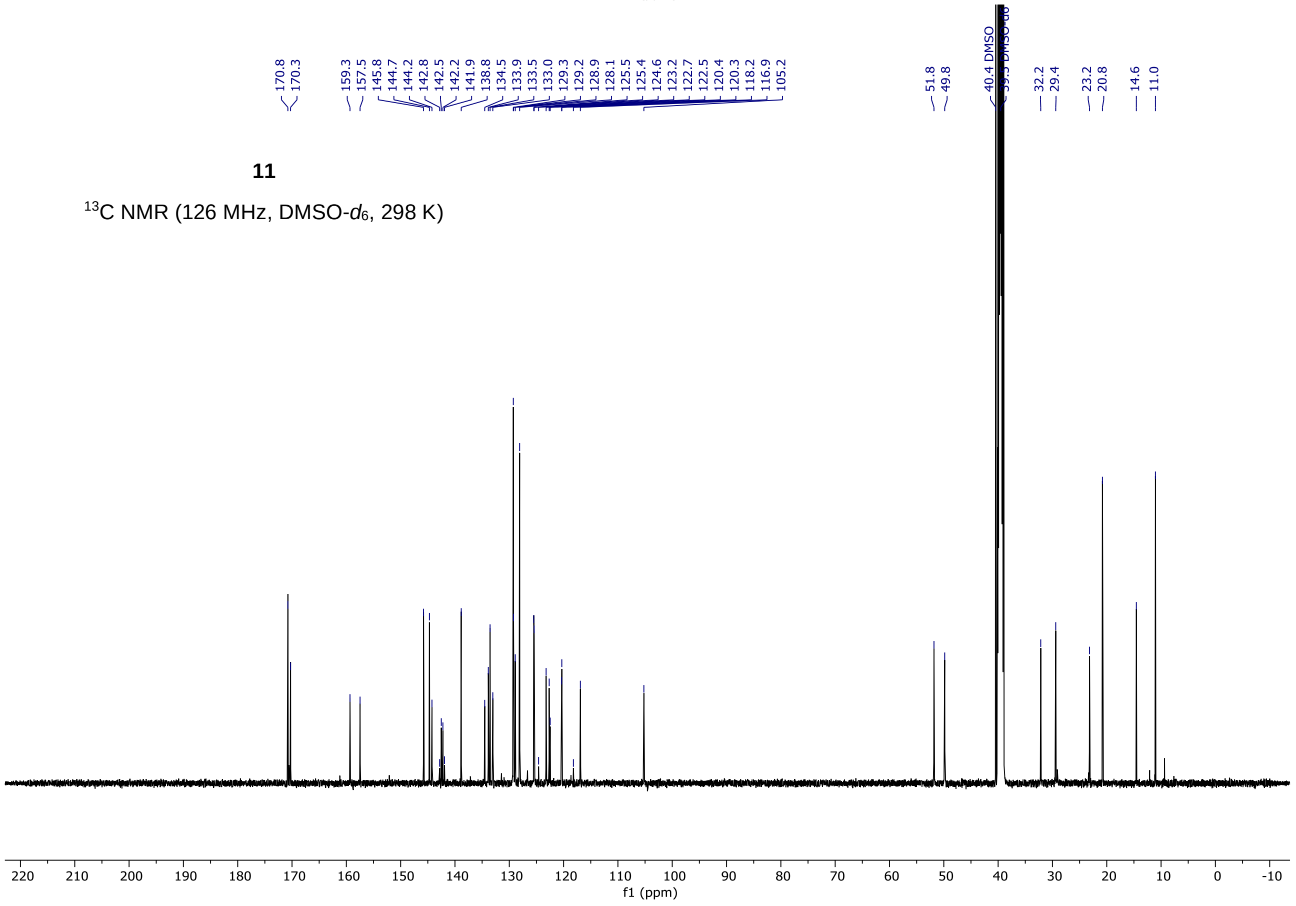

Supplementary Figure 133. ^{1}H NMR (top) and ^{13}C NMR (bottom) spectra of compound **11**. Frequency, temperature and solvent of measurement are indicated on each spectra.

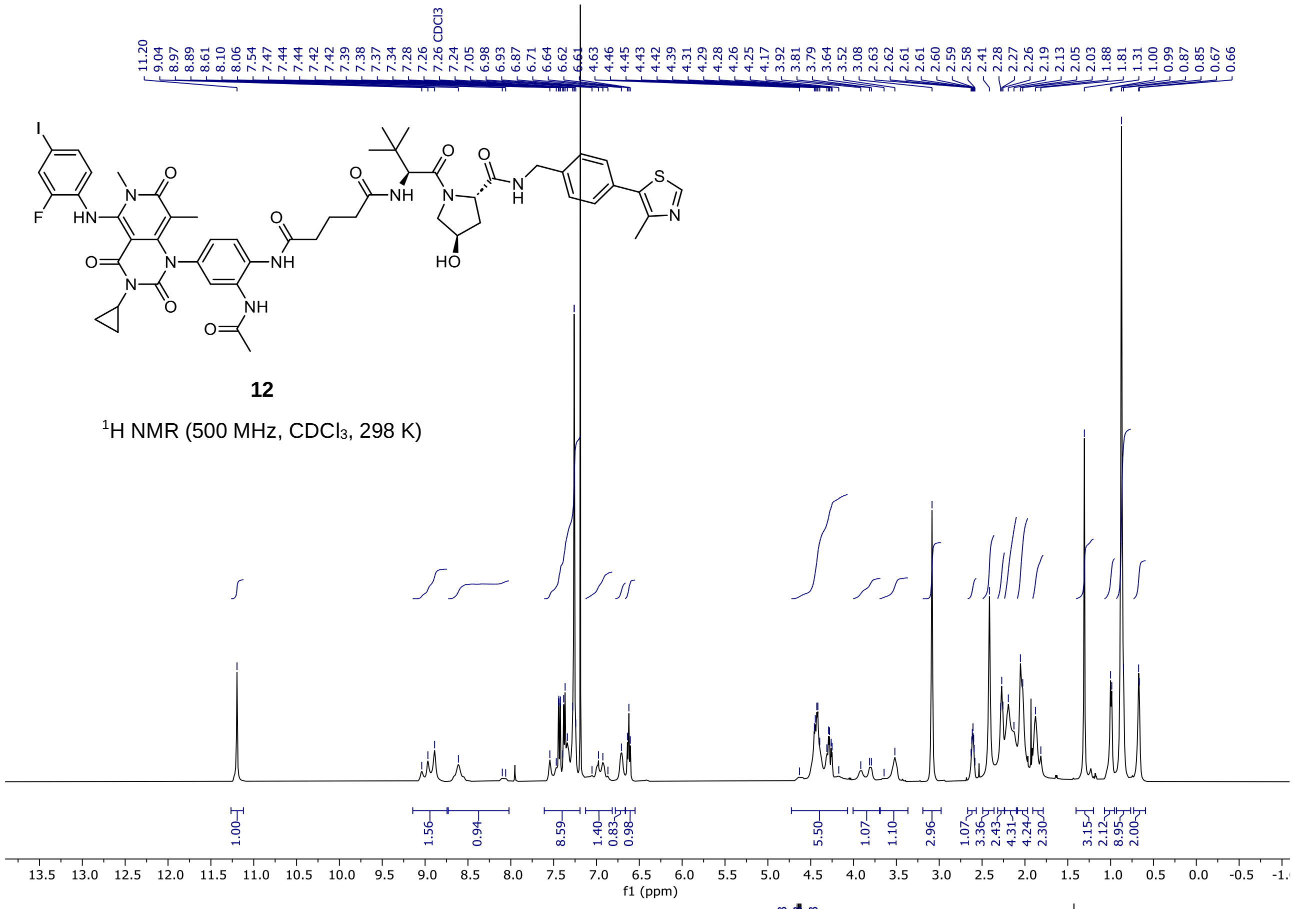

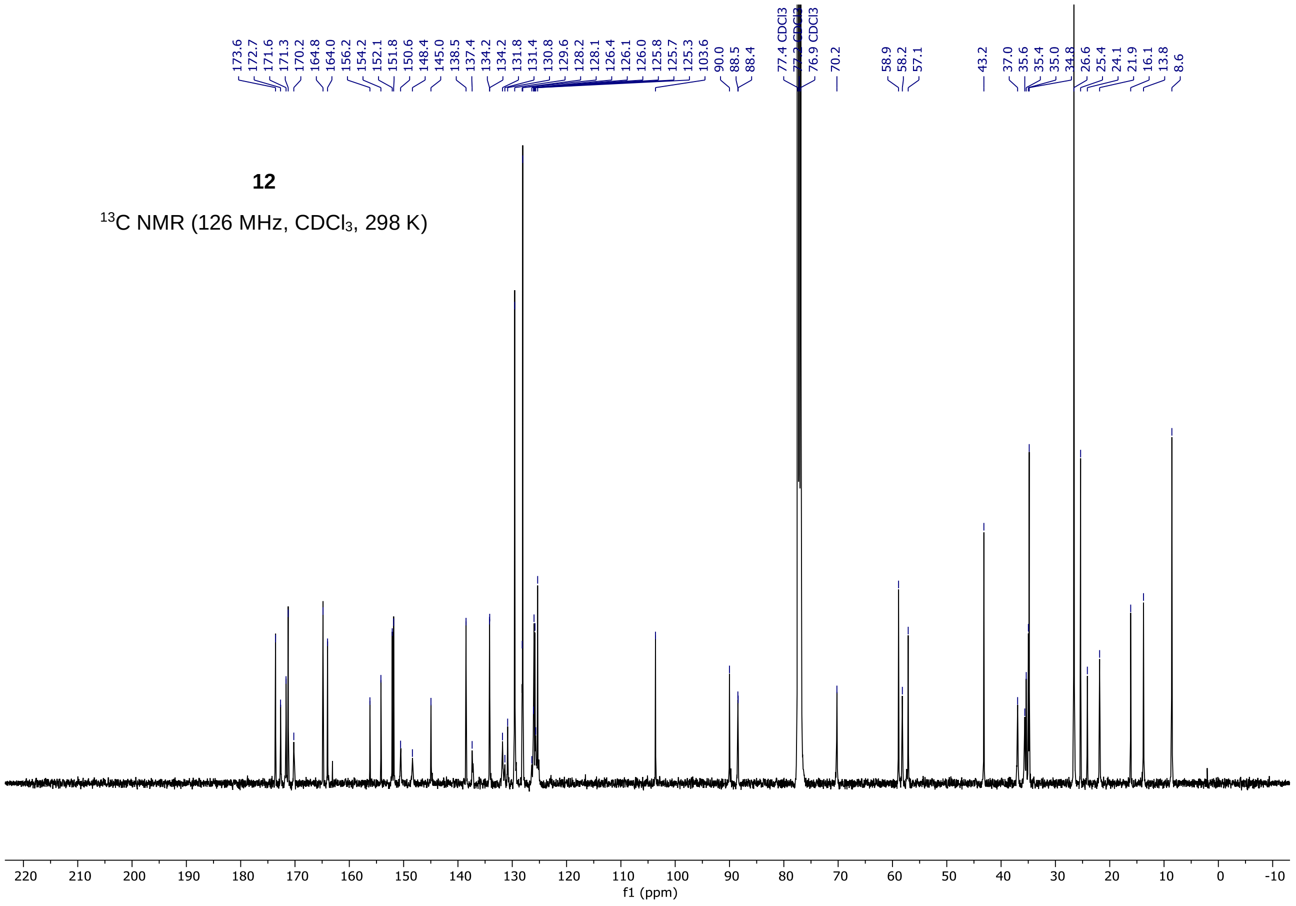

Supplementary Figure 134. ^{1}H NMR (top) and ^{13}C NMR (bottom) spectra of compound **12**. Frequency, temperature and solvent of measurement are indicated on each spectra.

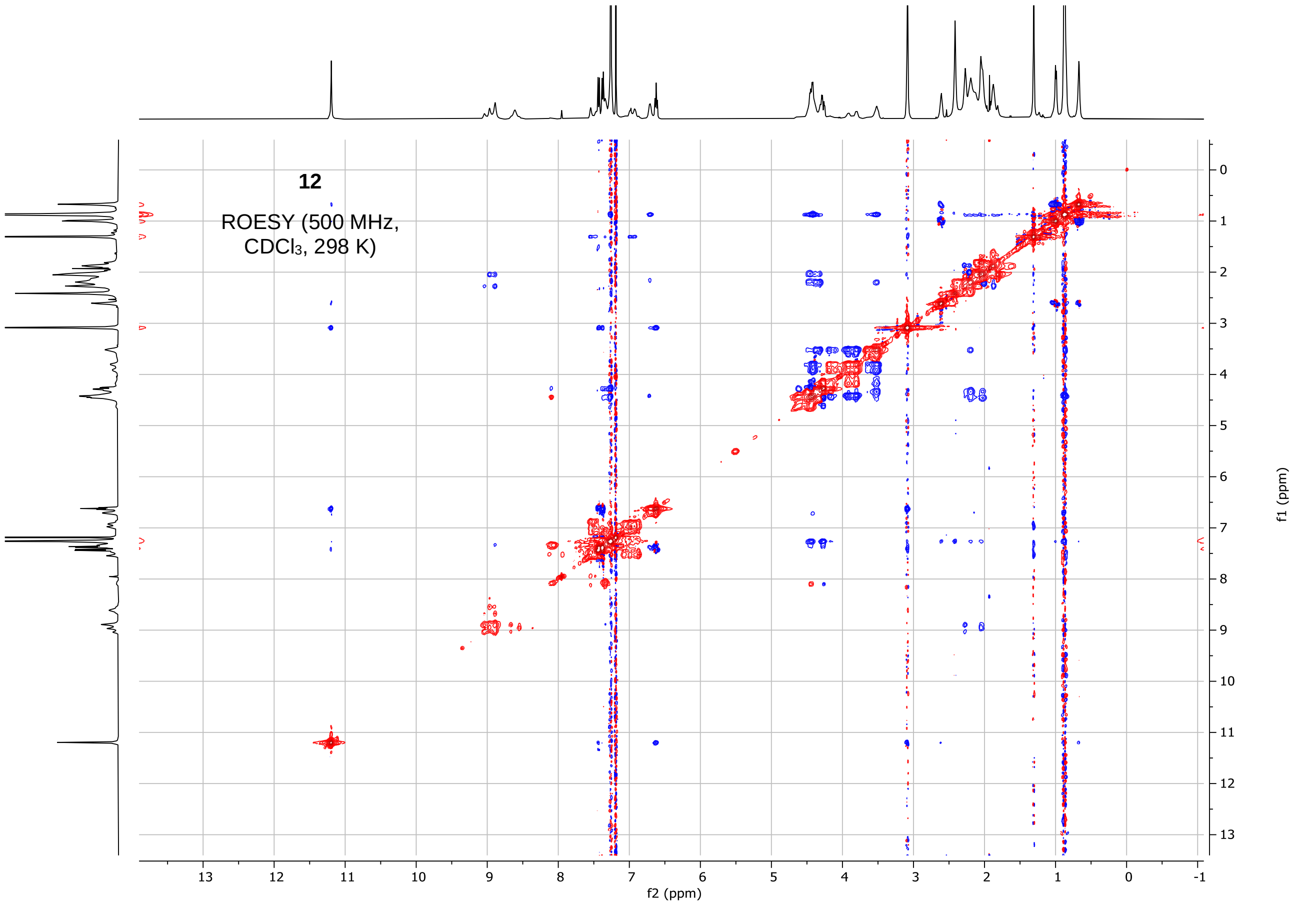

Supplementary Figure 135. ROESY spectrum of compound **12**. Frequency, temperature and solvent of measurement are indicated on the spectrum.

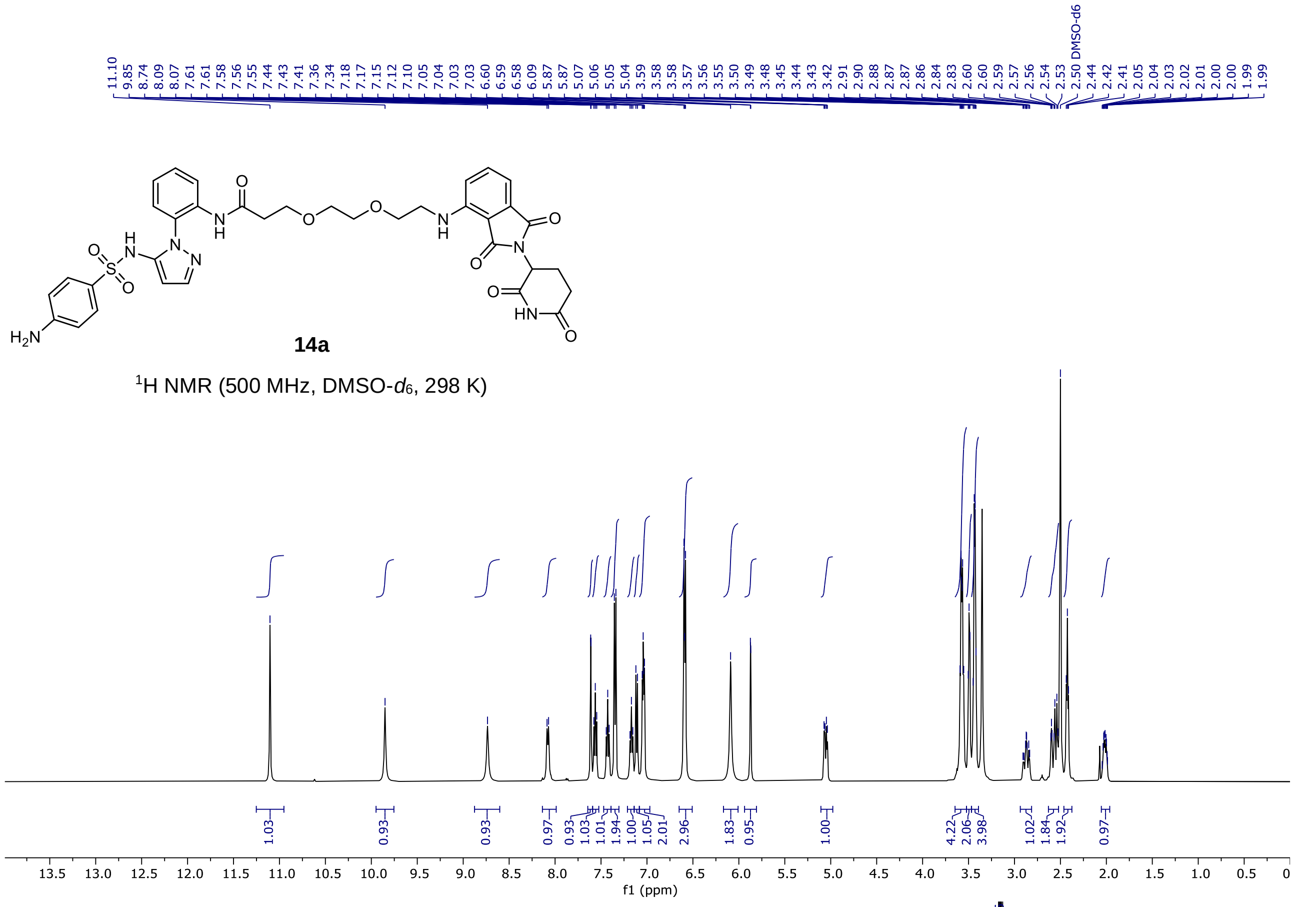

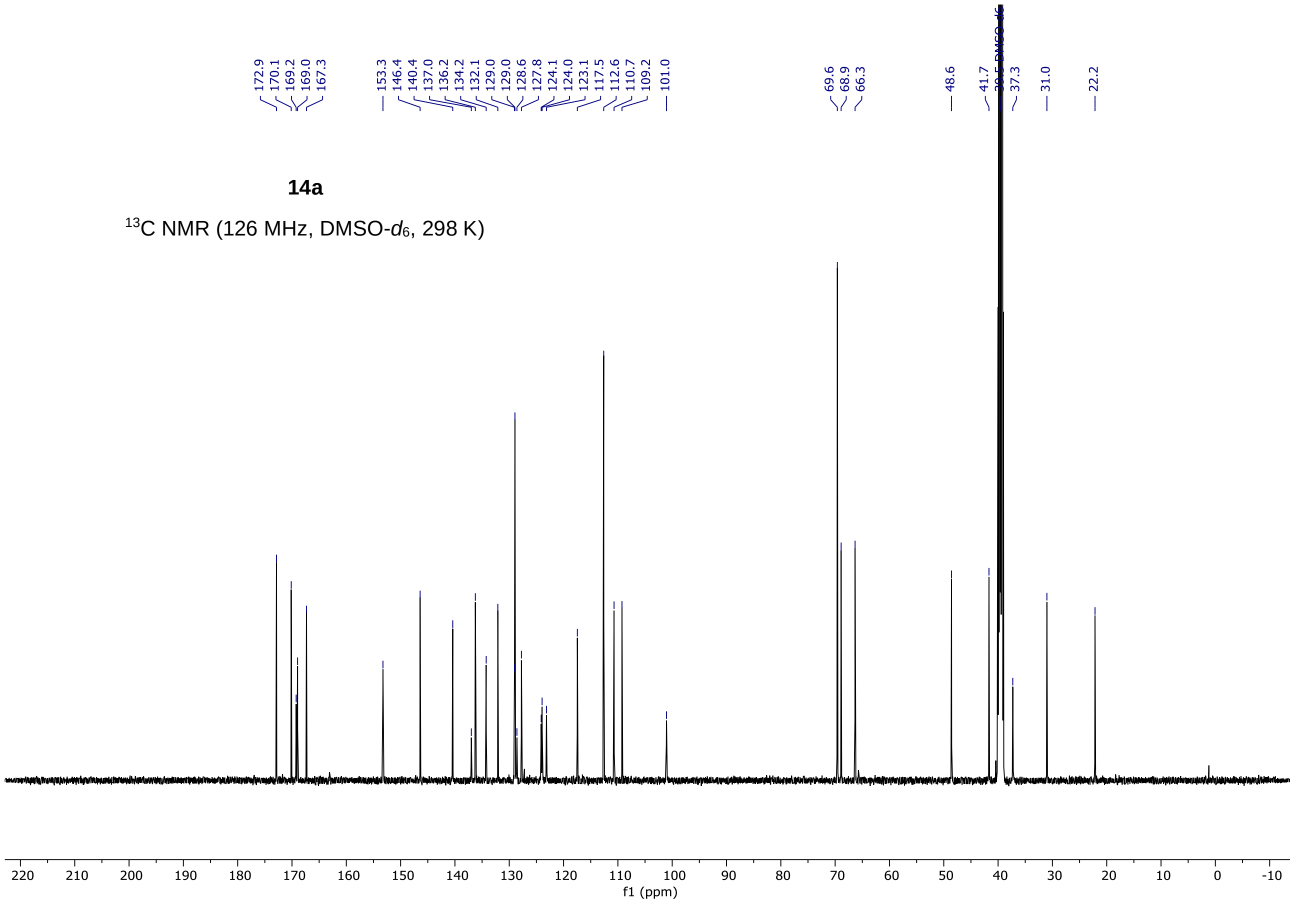

Supplementary Figure 136. ^{1}H NMR (top) and ^{13}C NMR (bottom) spectra of compound **14a**. Frequency, temperature and solvent of measurement are indicated on each spectra.

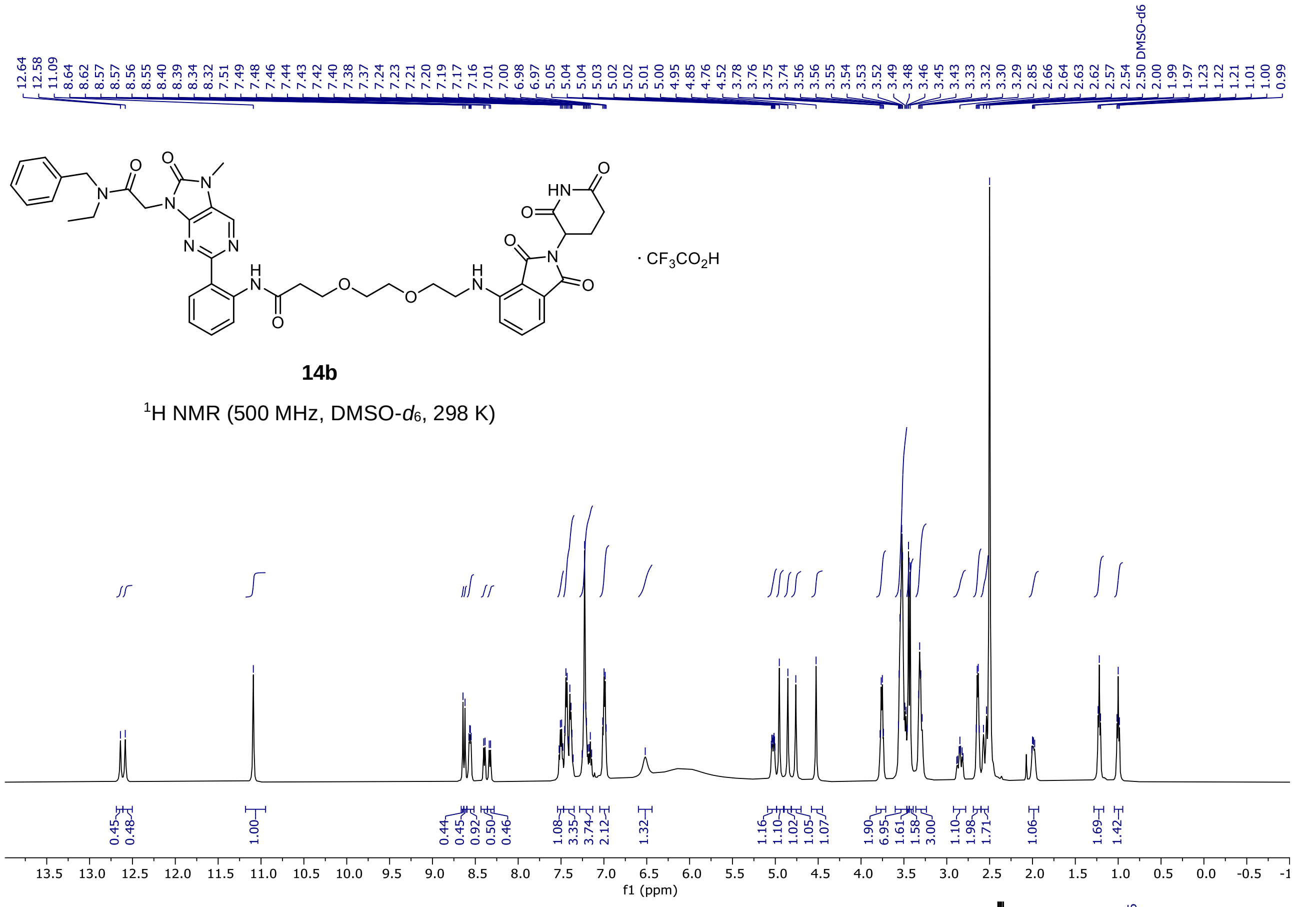

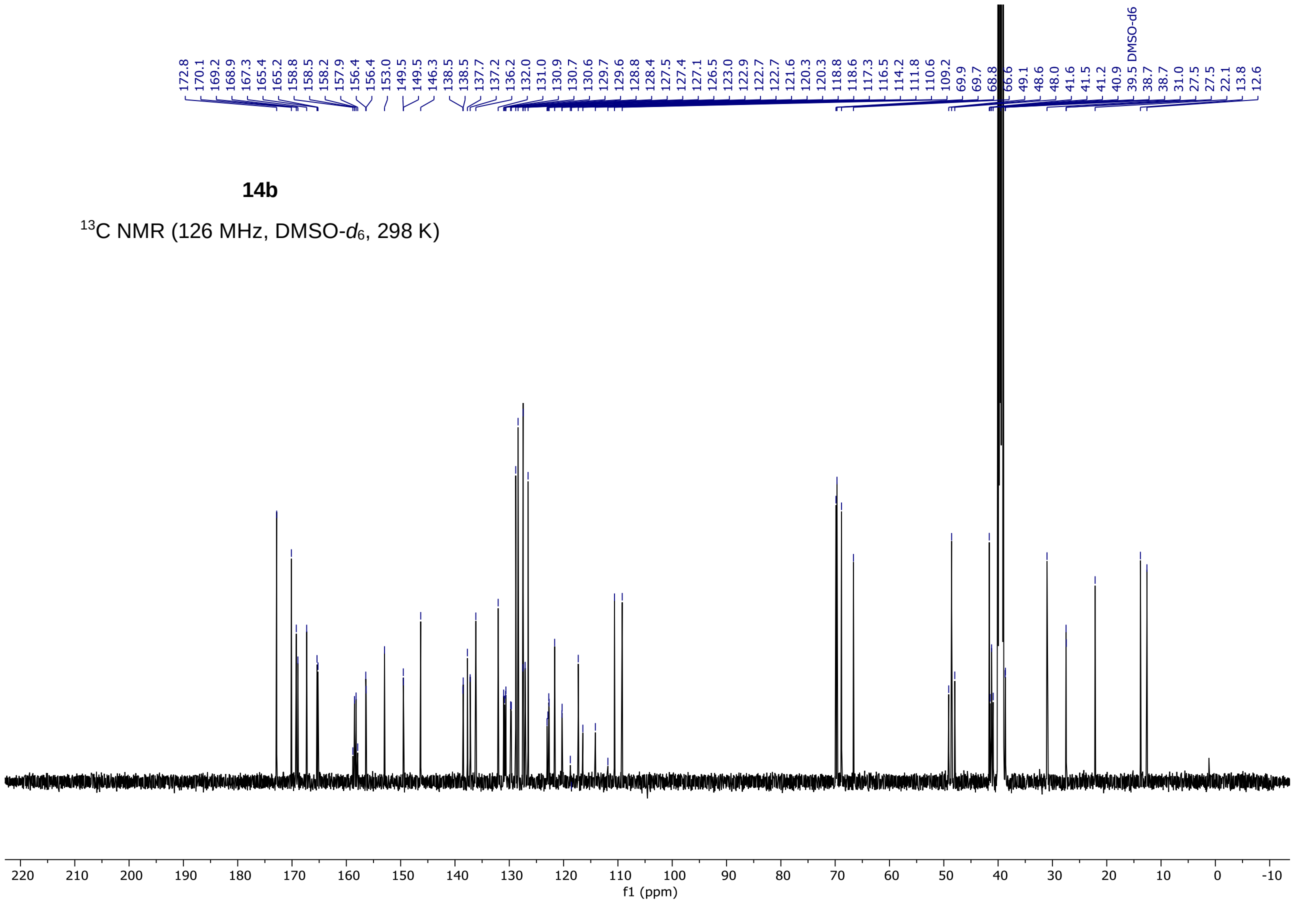

Supplementary Figure 137. ^{1}H NMR (top) and ^{13}C NMR (bottom) spectra of compound **14b**. Frequency, temperature and solvent of measurement are indicated on each spectra.

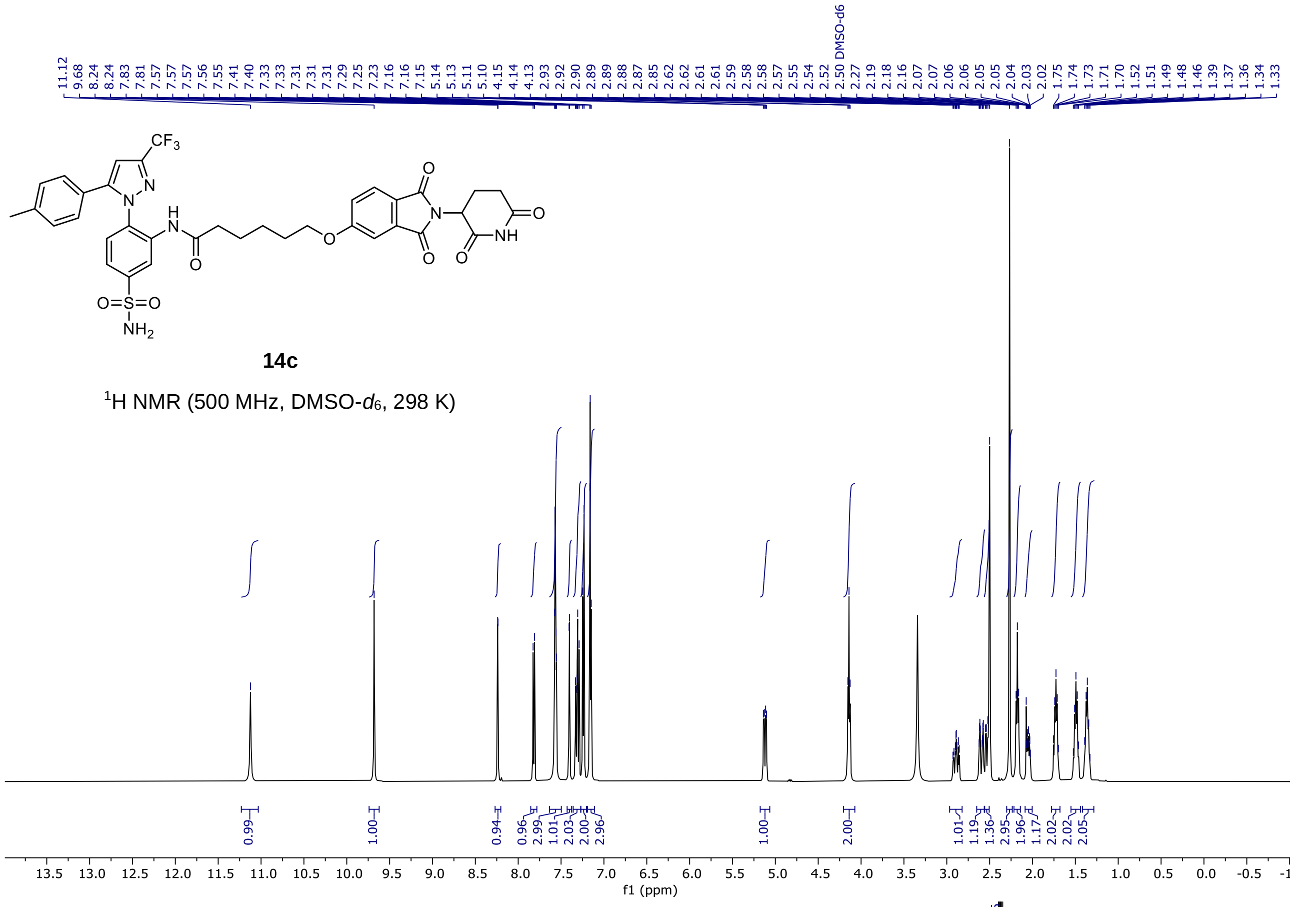

14c

^{1}H NMR (500 MHz, DMSO-d_6, 298 K)

14c

^{13}C NMR (126 MHz, DMSO-d_6, 298 K)

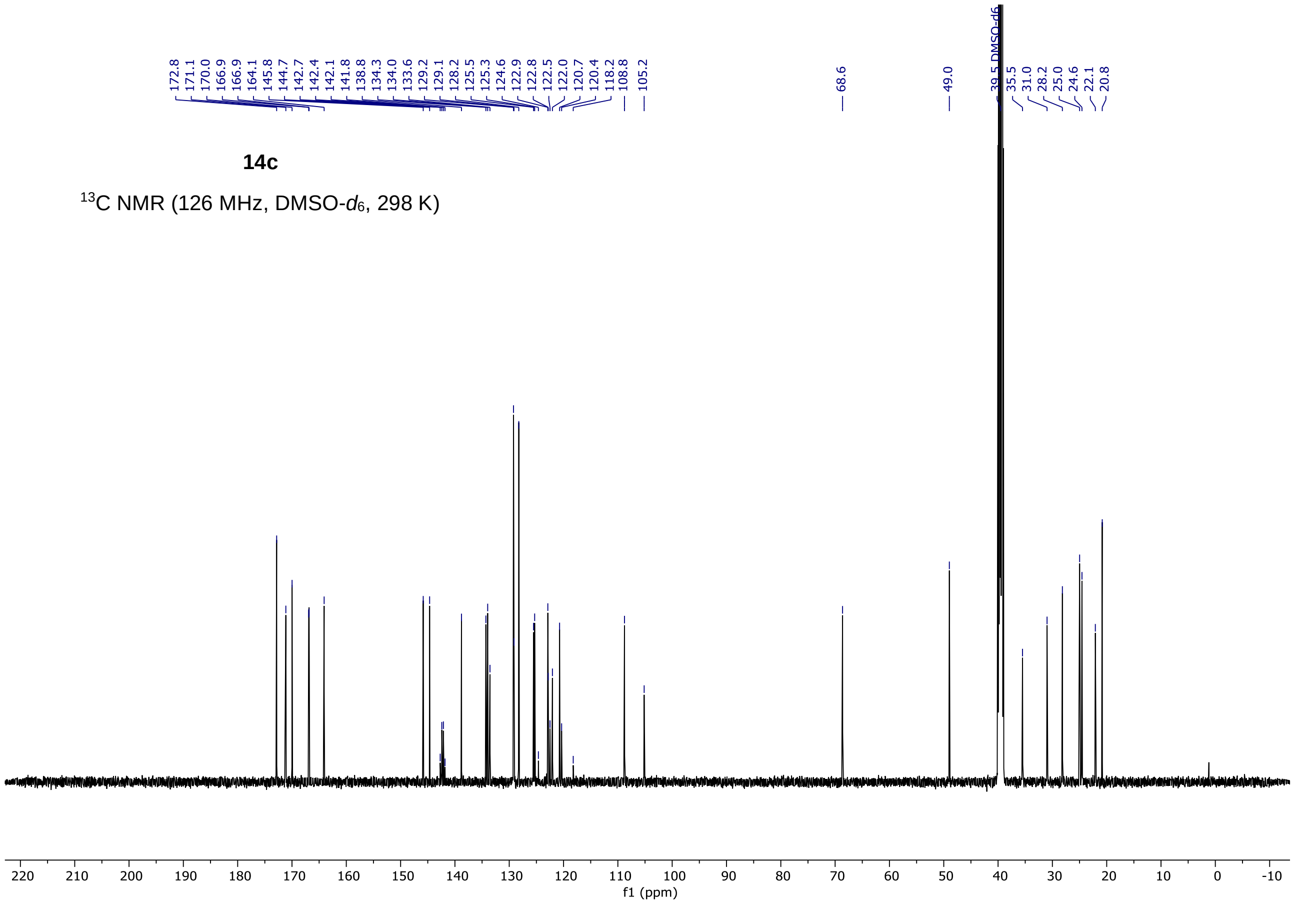

Supplementary Figure 138. ^{1}H NMR (top) and ^{13}C NMR (bottom) spectra of compound **14c**. Frequency, temperature and solvent of measurement are indicated on each spectra.

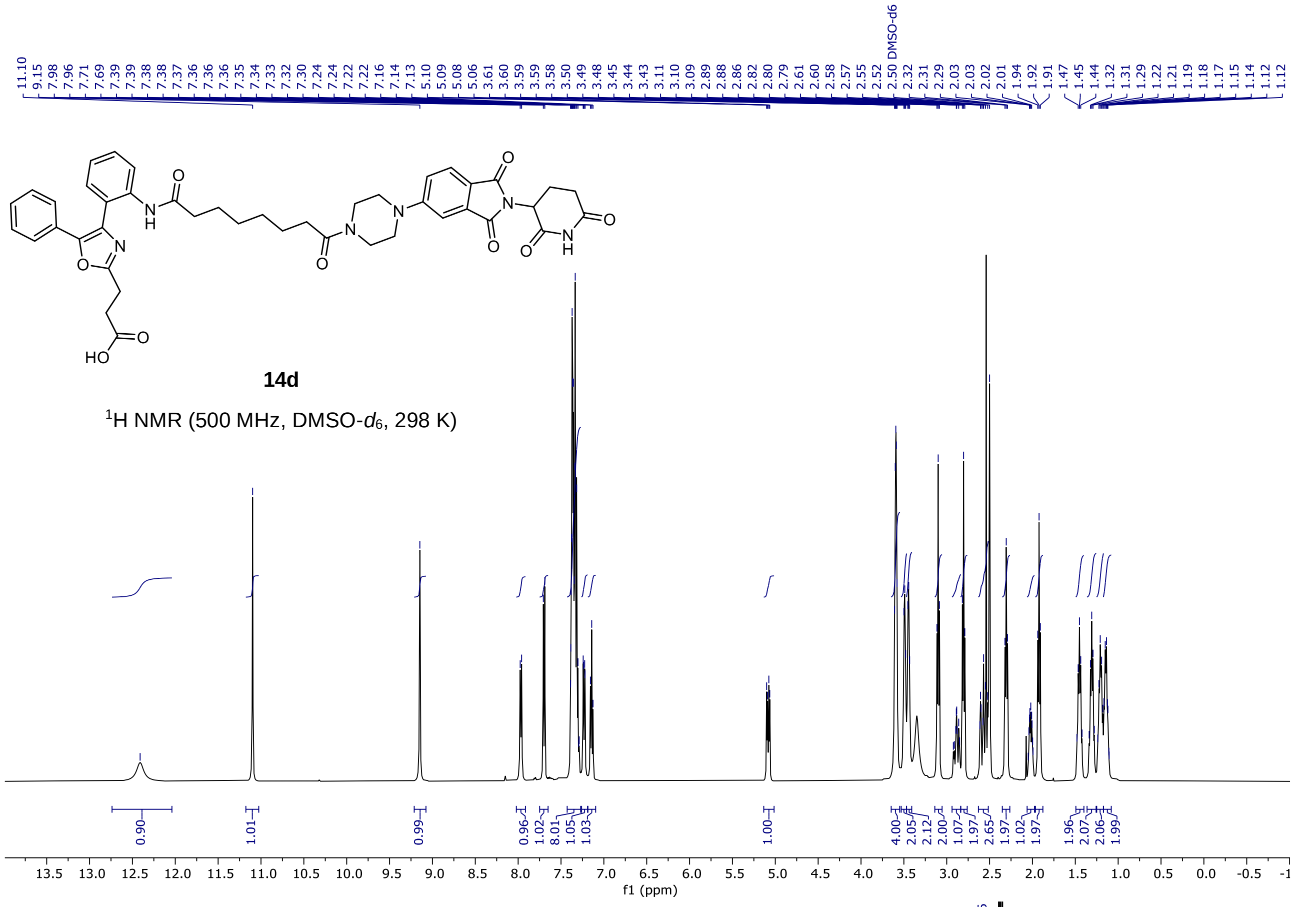

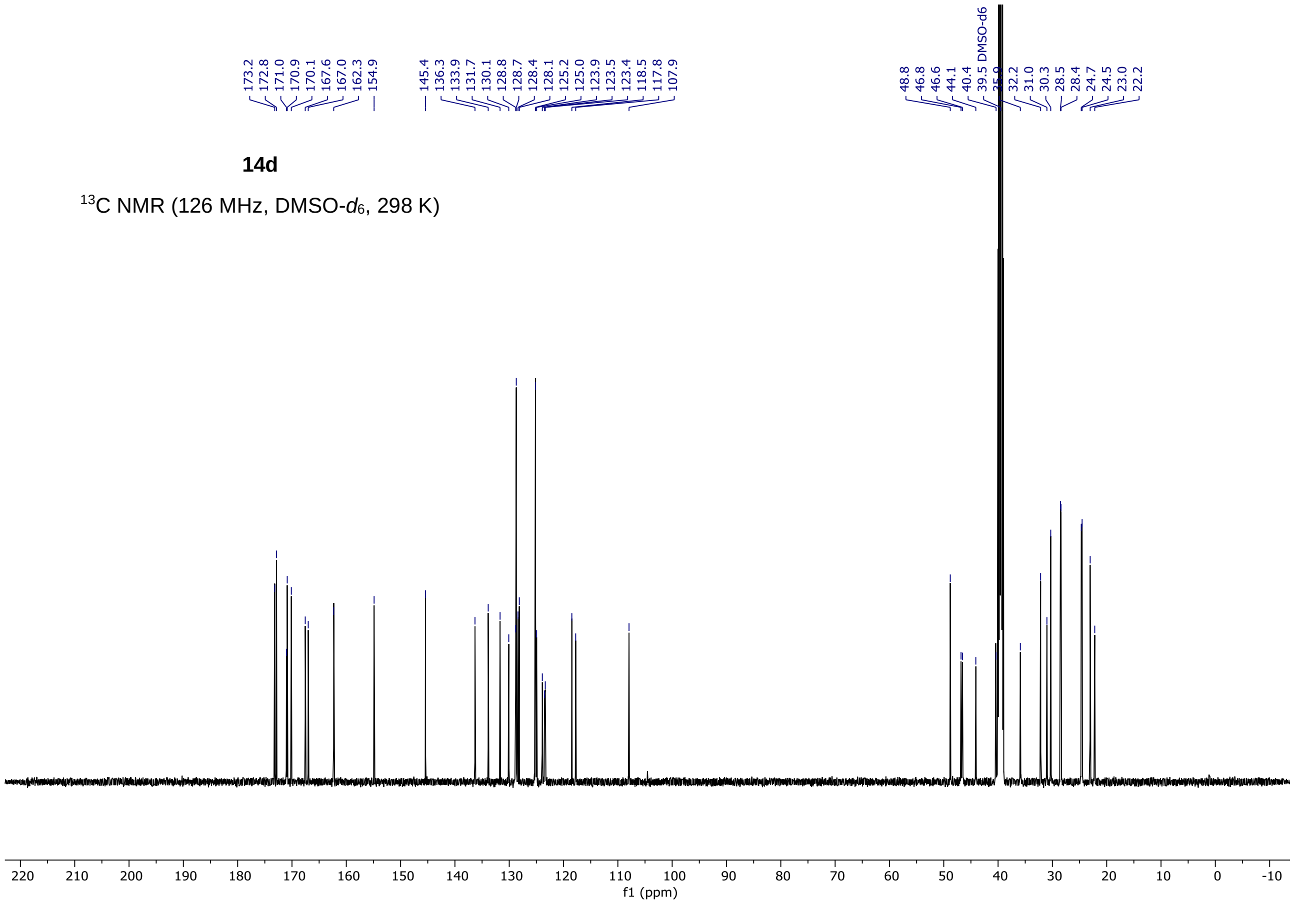

Supplementary Figure 139. ^{1}H NMR (top) and ^{13}C NMR (bottom) spectra of compound **14d**. Frequency, temperature and solvent of measurement are indicated on each spectra.

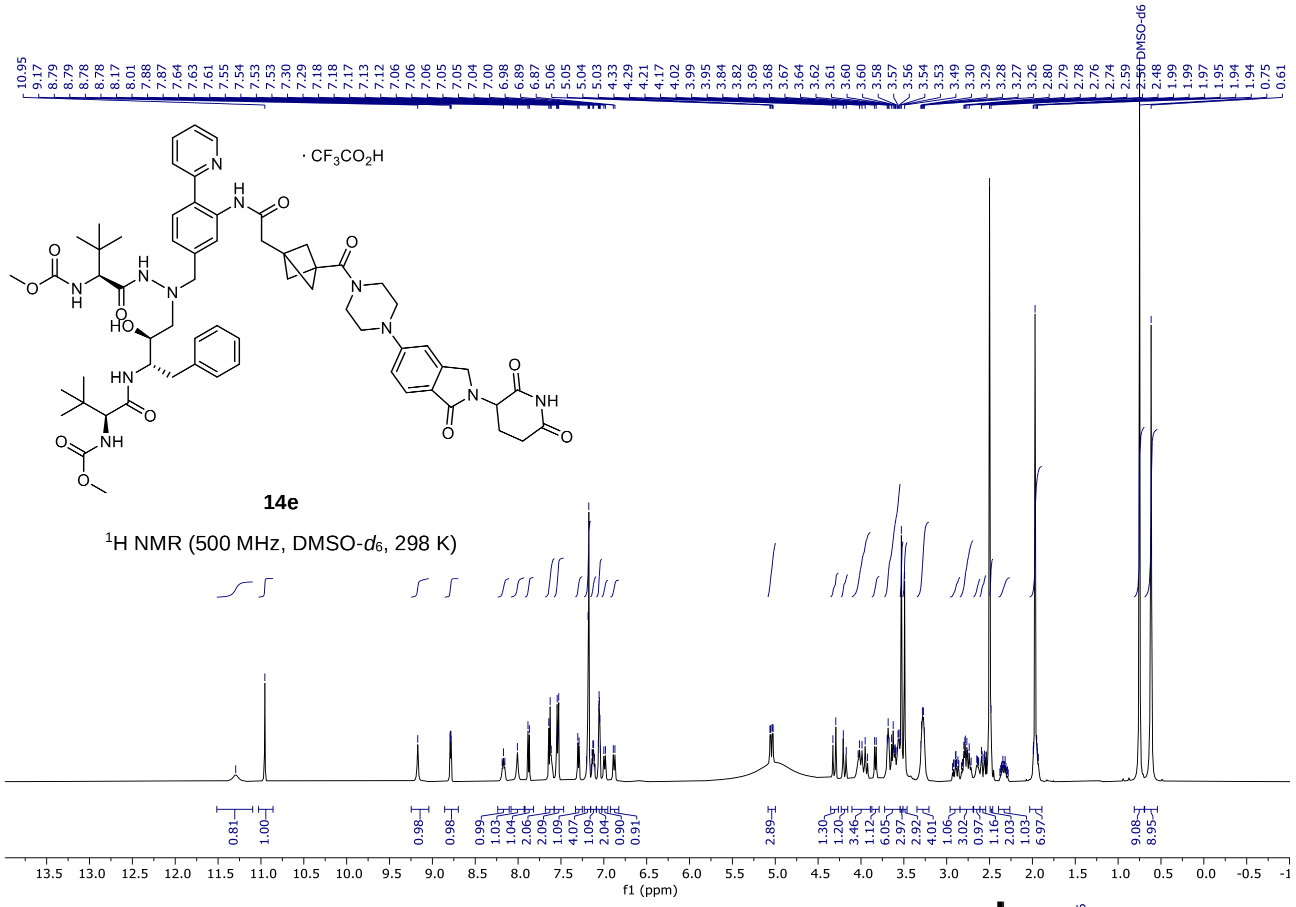

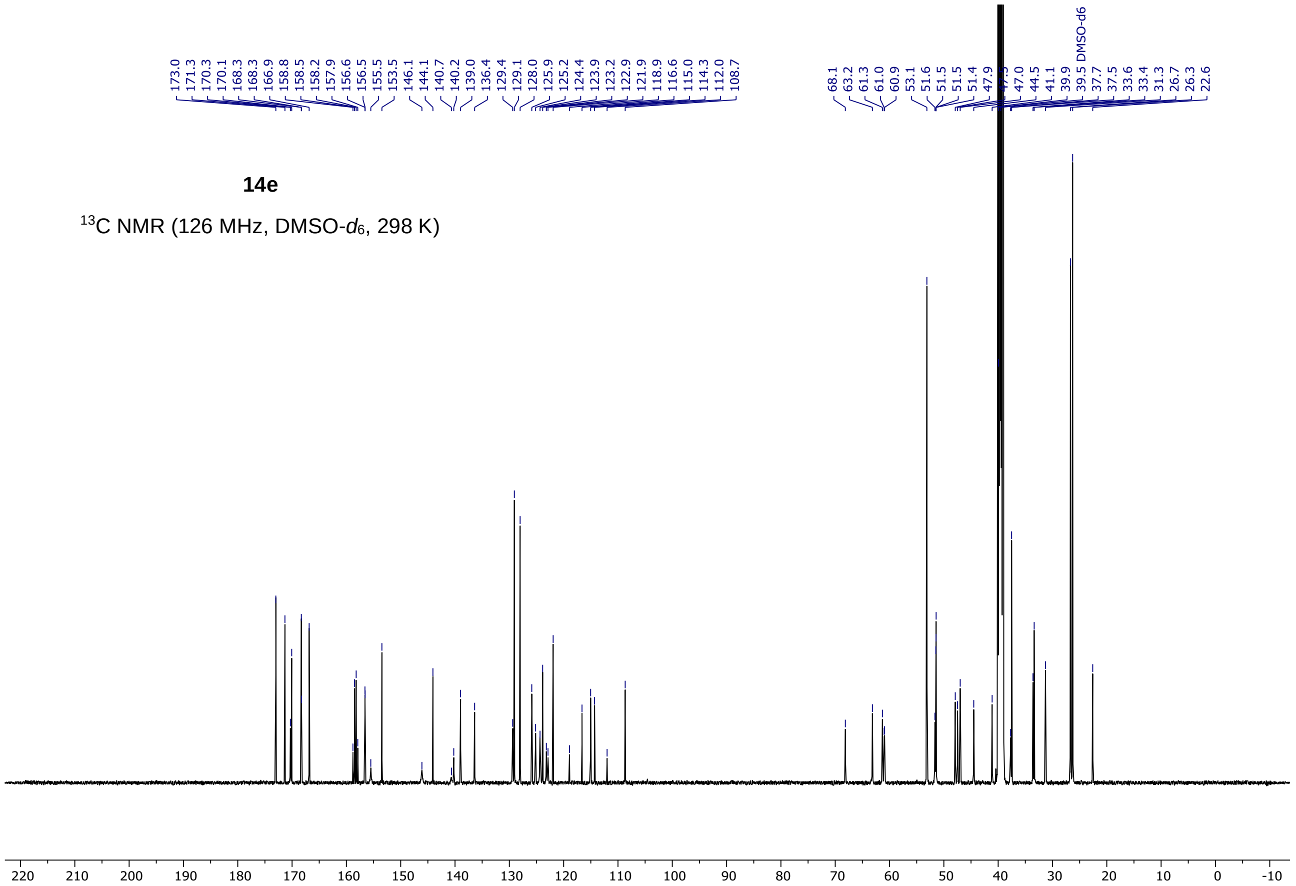

Supplementary Figure 140. ^{1}H NMR (top) and ^{13}C NMR (bottom) spectra of compound **14e**. Frequency, temperature and solvent of measurement are indicated on each spectra.

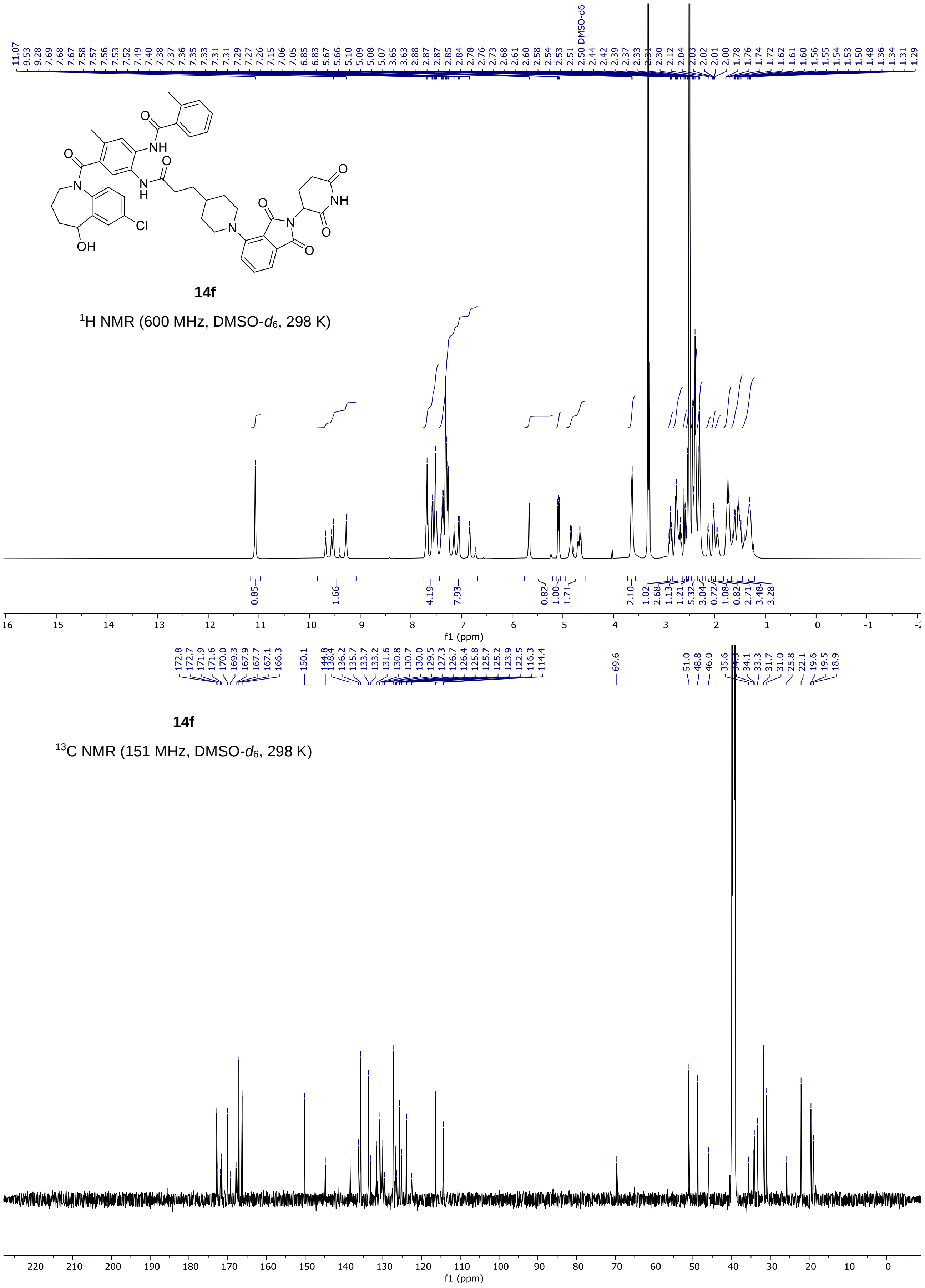

Supplementary Figure 141. ^{1}H NMR (top) and ^{13}C NMR (bottom) spectra of compound **14f**. Frequency, temperature and solvent of measurement are indicated on each spectra.

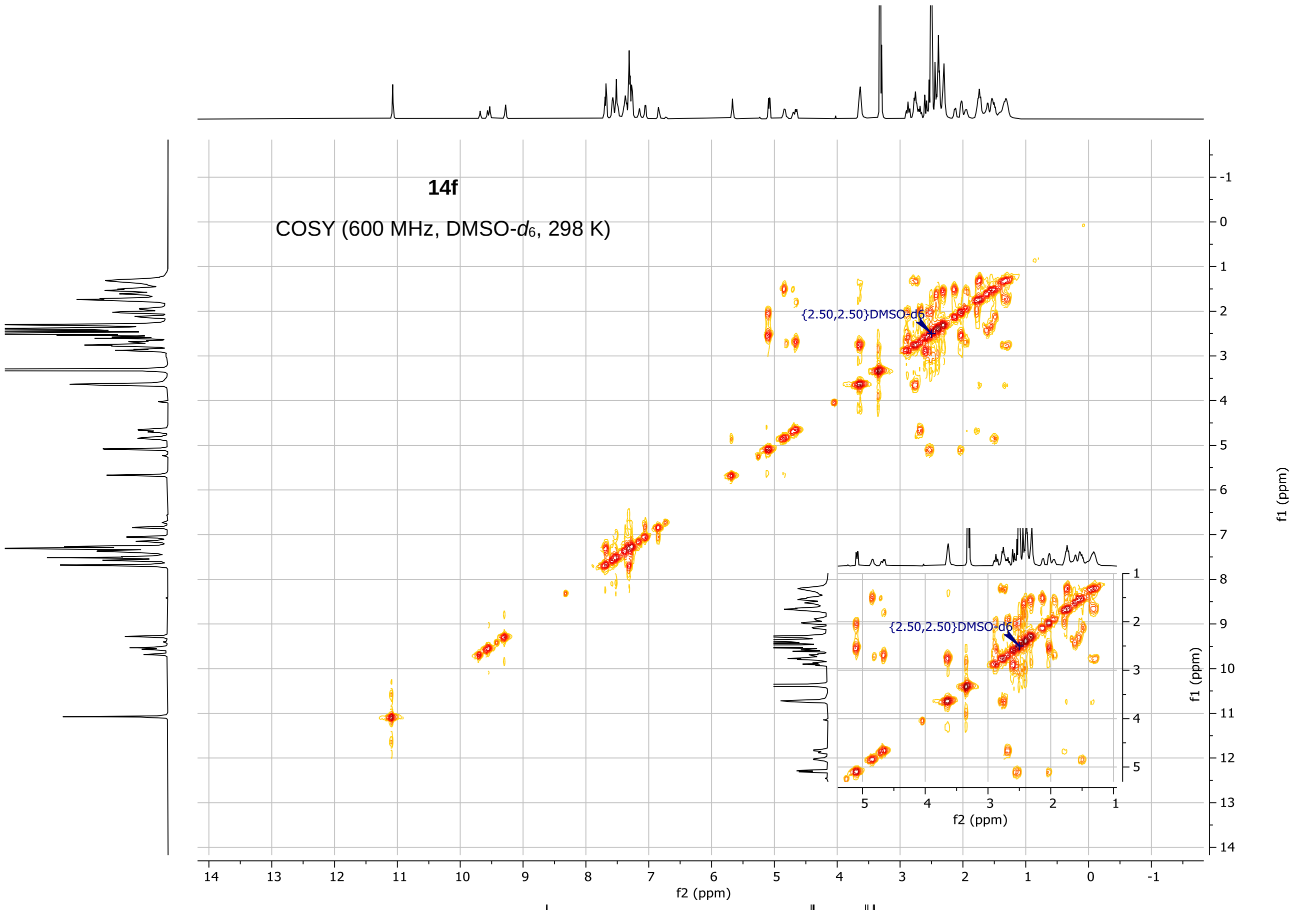

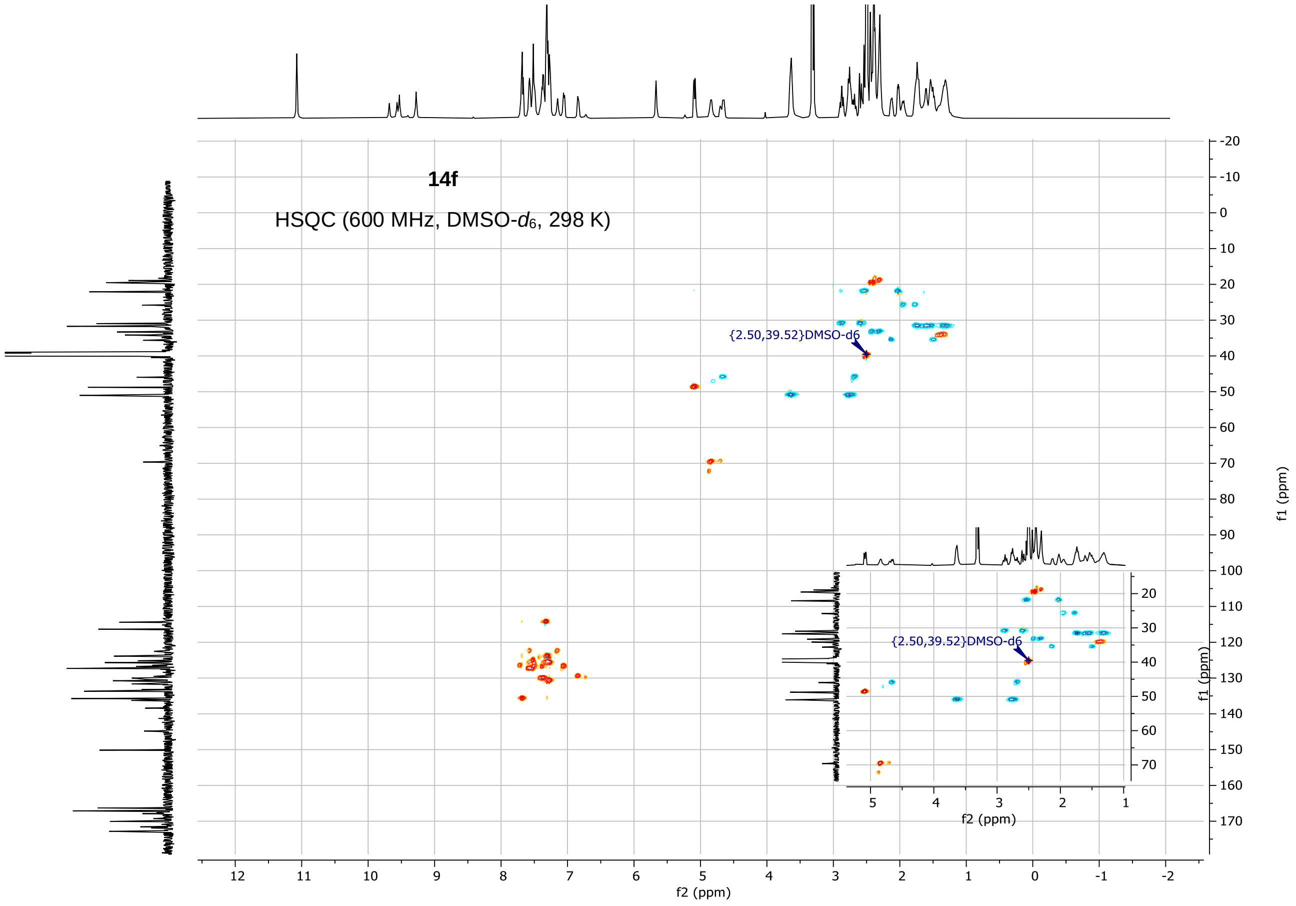

Supplementary Figure 142. COSY (top) and HSQC (bottom) spectra of compound **14f**. Frequency, temperature and solvent of measurement are indicated on each spectra.

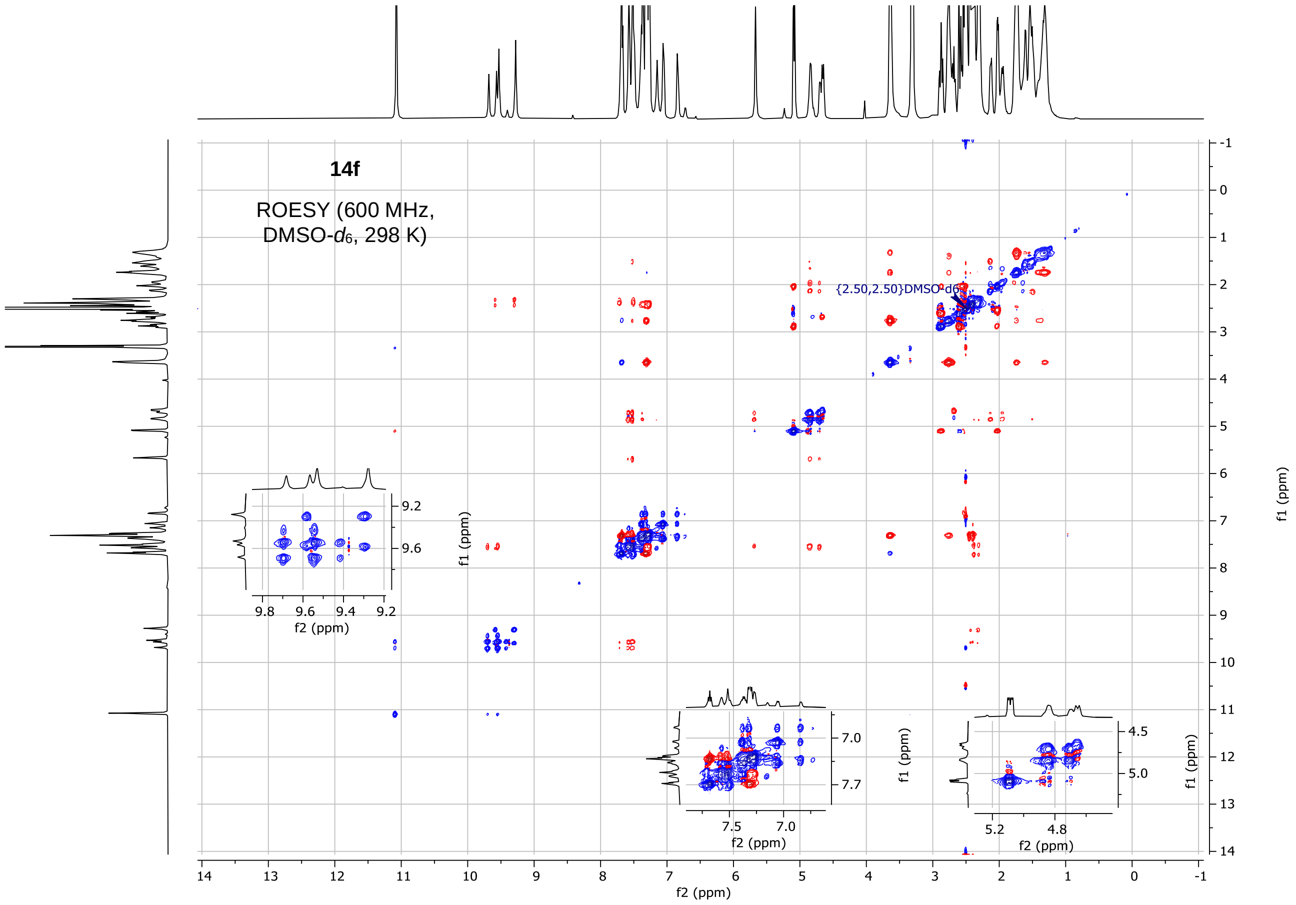

Supplementary Figure 143. ROESY spectrum of compound **14f**. Frequency, temperature and solvent of measurement are indicated on the spectrum.

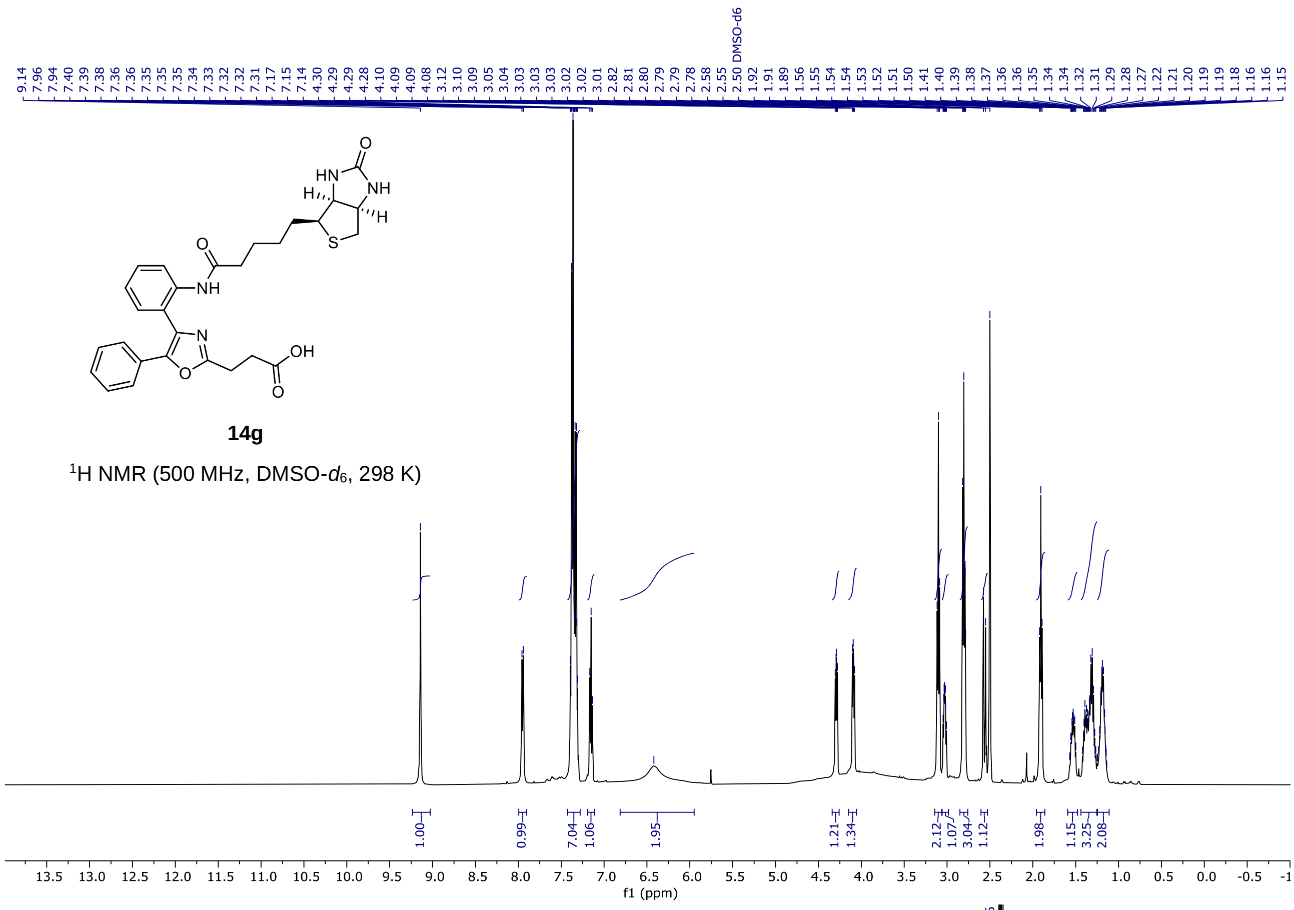

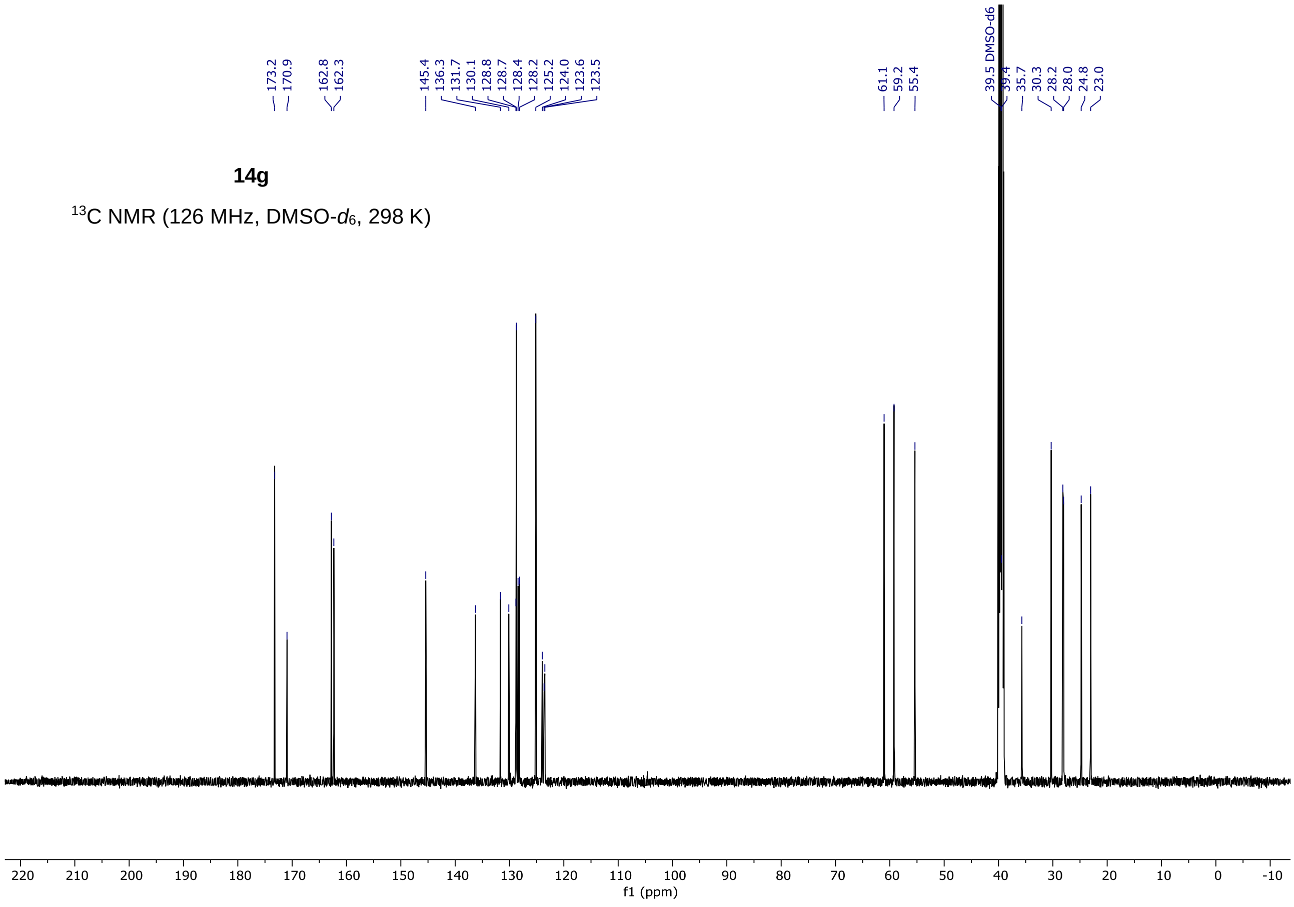

Supplementary Figure 144. ^{1}H NMR (top) and ^{13}C NMR (bottom) spectra of compound **14g**. Frequency, temperature and solvent of measurement are indicated on each spectra.

Supplementary References

1. McKay, A. I. *et al.* Identification of the Side Products That Diminish the Yields of the Monoamidated Product in Metal-Catalyzed C-H Amidation of 2-Phenylpyridine with Arylisocyanates. *J. Org. Chem.* **85**, 2680–2687 (2020).
2. Rigaku Oxford Diffraction. CrysAlisPro. 2014. Rigaku Oxford Diffraction, Yarnton, Oxfordshire, England.
3. Bourhis, L. J., Dolomanov, O. V., Gildea, R. J., Howard, J. A. K. & Puschmann, H. The anatomy of a comprehensive constrained, restrained, refinement program for the modern computing environment - Olex2 dissected. *Acta Cryst.* **A71**, 59–71 (2015).
4. Dolomanov, O. V.; Bourhis, L. J.; Gildea, R. J.; Howard, J. A. K. & Puschmann, H. OLEX2: A complete structure solution, refinement and analysis program. *J. Appl. Cryst.* **42**, 339–341 (2009).
5. Sheldrick, G. M. A short history of SHELX. *Acta Cryst*. **A64**, 112–122 (2008).
6. Di Fiore, A. *et al.* Carbonic anhydrase inhibitors: Valdecoxib binds to a different active site region of the human isoform II as compared to the structurally related cyclooxygenase II 'selective' inhibitor celecoxib. *Bioorg. Med. Chem. Lett.* **16**, 437–442 (2006).
7. Clemente, J. C. *et al.* Analysis of HIV-1 CRF_01 A/E Protease Inhibitor Resistance: Structural Determinants for Maintaining Sensitivity and Developing Resistance to Atazanavir. *Biochemistry* **45**, 5468–5477 (2006).
8. Istvan, E. S. & Deisenhofer, J. Structural Mechanism for Statin Inhibition of HMG-CoA Reductase. *Science* **292**, 1160–1164 (2001).
9. Zheng, X. *et al.* The molecular basis for inhibition of sulindac and its metabolites towards human aldose reductase. *FEBS Letters* **586**, 55–59 (2012).
10. Ha-Duong, N.-T. *et al.* Synthesis of Sulfaphenazole Derivatives and Their Use as Inhibitors and Tools for Comparing the Active Sites of Human Liver Cytochromes P450 of the 2C Subfamily. *J. Med. Chem.* **44**, 3622–3631 (2001).
11. Chen, H. *et al.* The optimization and characterization of functionalized sulfonamides derived from sulfaphenazole against Mycobacterium tuberculosis with reduced CYP 2C9 inhibition. *Bioorg. Med. Chem. Lett.* **40**, 127924 (2021).
12. Costanzo, A. *et al.* Synthesis of derivatives of pyrazolo[1,5-a]pyrrolo[1,2-c][1,3,6]-benzotriazocine, a new class of compounds with potential CNS activity. *J. Heterocyclic Chem.* **29**, 1499–1505 (1992).
13. Murata, T.; Masumoto, K.; Kondo, K.; Furukawa, K.; Oka, M. (Dainippon Sumitomo Pharma Co., Ltd.) 2-aryl-8-oxodihydropurine derivative, process for the producing the same, medicinal compositions containing the same, and intermediates thereof. US6372740, 2002, B1.
14. Kumata, K. *et al.* Synthesis and Evaluation of Novel Carbon-11 Labeled Oxopurine Analogues for Positron Emission Tomography Imaging of Translocator Protein (18 kDa) in Peripheral Organs. *J. Med. Chem.* **54**, 6040–6049 (2011).
15. Abdellatif, K. R. A. *et al.* Diazen-1-ium-1,2-diolated nitric oxide donor ester prodrugs of 5-(4-hydroxymethylphenyl)-1-(4-aminosulfonylphenyl)-3-trifluoromethyl-1H-pyrazole and its methanesulfonyl analog: Synthesis, biological evaluation and nitric oxide release studies. *Bioorg. Med. Chem.* **16**, 9694–9698 (2008).
16. Kramer, J. S. *et al.* Discovery of the First in Vivo Active Inhibitors of the Soluble Epoxide Hydrolase Phosphatase Domain. *J. Med. Chem.* **62**, 8443–8460 (2019).
17. Bold, G. *et al.* New Aza-Dipeptide Analogues as Potent and Orally Absorbed HIV-1 Protease Inhibitors: Candidates for Clinical Development. *J. Med. Chem.* **41**, 3387–3401 (1998).
18. Zhao, H.-Y. *et al.* Discovery of 2-(pyridin-2-yl)aniline as a directing group for the sp2 C–H bond amination mediated by cupric acetate. *Org. Biomol. Chem.* **15**, 6622–6631 (2017).
19. Sethi, M. K., Singh Rawat, V., Thirunavukarasu, J., Yerramalla, R. & Kumar, A. Synthesis and Characterization of Tolvaptan Impurities. *Advances in Chemistry* **2014**, 471950 (2014).
20. Farr, C. M. B. *et al.* Designing a Planar Chiral Rhodium Indenyl Catalyst for Regio- and Enantioselective Allylic C–H Amidation. *J. Am Chem. Soc.* **142**, 13996–14004 (2020).
21. Burman, J. S., Harris, R. J., Farr, C. M. B., Bacsa, J. & Blakey, S. B. Rh(III) and Ir(III)Cp* Complexes Provide Complementary Regioselectivity Profiles in Intermolecular Allylic C–H Amidation Reactions. *ACS Catal.* **9**, 5474–5479 (2019).
22. Hong, S. Y. & Chang, S. Stereodefined Access to Lactams via Olefin Difunctionalization: Iridium Nitrenoids as a Motif of LUMO-Controlled Dipoles. *J. Am. Chem. Soc.* **141**, 10399–10408 (2019).
