# Supplementary material for: Late-stage synthesis of heterobifunctional molecules for PROTAC applications via ruthenium-catalysed C‒H amidation
Source: Nat Commun. 2023 Dec 12;14:8222. doi: 10.1038/s41467-023-43789-9 (PMC10716378; doi:10.1038/s41467-023-43789-9)
Supplement: Supplementary file 3 — Reporting Summary [file 41467_2023_43789_MOESM3_ESM.pdf]

nature portfolio

Corresponding author(s): Magnus J. Johansson and Lutz Ackermann

Last updated by author(s): Nov 2, 2023

Reporting Summary

Nature Portfolio wishes to improve the reproducibility of the work that we publish. This form provides structure for consistency and transparency in reporting. For further information on Nature Portfolio policies, see our Editorial Policies and the Editorial Policy Checklist.

Statistics

For all statistical analyses, confirm that the following items are present in the figure legend, table legend, main text, or Methods section.

n/a	Confirmed	
☐	☒	The exact sample size (*n*) for each experimental group/condition, given as a discrete number and unit of measurement
☒	☐	A statement on whether measurements were taken from distinct samples or whether the same sample was measured repeatedly
☒	☐	The statistical test(s) used AND whether they are one- or two-sided<br>*Only common tests should be described solely by name; describe more complex techniques in the Methods section.*
☒	☐	A description of all covariates tested
☒	☐	A description of any assumptions or corrections, such as tests of normality and adjustment for multiple comparisons
☒	☐	A full description of the statistical parameters including central tendency (e.g. means) or other basic estimates (e.g. regression coefficient) AND variation (e.g. standard deviation) or associated estimates of uncertainty (e.g. confidence intervals)
☒	☐	For null hypothesis testing, the test statistic (e.g. *F*, *t*, *r*) with confidence intervals, effect sizes, degrees of freedom and *P* value noted<br>*Give P values as exact values whenever suitable.*
☒	☐	For Bayesian analysis, information on the choice of priors and Markov chain Monte Carlo settings
☒	☐	For hierarchical and complex designs, identification of the appropriate level for tests and full reporting of outcomes
☒	☐	Estimates of effect sizes (e.g. Cohen's *d*, Pearson's *r*), indicating how they were calculated

Our web collection on statistics for biologists contains articles on many of the points above.

Software and code

Policy information about availability of computer code

Data collection	TopSpin v3 and IconNMR v5 for collection of NMR data; MassLynx v4 for collection of UPLC-MS data; CrysAlisPro 1.171.42.35a, Olex2.solve, Olex2, ShelXL for collection and refinement of X-ray diffraction data.
Data analysis	MesReNova v14 for analysis of NMR data; MassLinx v4 for analysis of UPLC-MS data; TIBCO Spotfire v11 for data visualisation; Mercury v4 for X-ray structure visualization; MOE 2022.02 for analysis and visualization of X-Ray data, and modeling of protein surfaces.

For manuscripts utilizing custom algorithms or software that are central to the research but not yet described in published literature, software must be made available to editors and reviewers. We strongly encourage code deposition in a community repository (e.g. GitHub). See the Nature Portfolio guidelines for submitting code & software for further information.

Data

Policy information about availability of data

All manuscripts must include a data availability statement. This statement should provide the following information, where applicable:

- Accession codes, unique identifiers, or web links for publicly available datasets
- A description of any restrictions on data availability
- For clinical datasets or third party data, please ensure that the statement adheres to our policy

The data generated in this study are provided within the paper and the Supplementary Information file. This includes additional structures; unsuccessful dioxazolones; extended optimization data; full details on LSF informer library screen; proposed de novo syntheses; crystallographic data for compound 8r; analysis of accessible exit vectors (the crystallographic data used are available free of charge in the PDB database, under PDB accession codes: 2AW1 [https://

doi.org/10.2210/pdb2AW1/pdb], 2AQU [https://doi.org/10.2210/pdb2aqu/pdb], 1HWK [https://doi.org/10.2210/pdb1hwk/pdb] and 3S3G [https://doi.org/10.2210/pdb3s3g/pdb]); experimental details and characterization data; NMR spectra for novel compounds. Crystallographic data for compound 8r has been deposited at the Cambridge Crystallographic Data Centre, under deposition number CCDC 2251355 [https://www.ccdc.cam.ac.uk/structures/Search?Ccdcid=2251355].

Research involving human participants, their data, or biological material

Policy information about studies with human participants or human data. See also policy information about sex, gender (identity/presentation), and sexual orientation and race, ethnicity and racism.

Reporting on sex and gender	N/A
Reporting on race, ethnicity, or other socially relevant groupings	N/A
Population characteristics	N/A
Recruitment	N/A
Ethics oversight	N/A

Note that full information on the approval of the study protocol must also be provided in the manuscript.

Field-specific reporting

Please select the one below that is the best fit for your research. If you are not sure, read the appropriate sections before making your selection.

[x] Life sciences [ ] Behavioural & social sciences [ ] Ecological, evolutionary & environmental sciences

For a reference copy of the document with all sections, see nature.com/documents/nr-reporting-summary-flat.pdf

Life sciences study design

All studies must disclose on these points even when the disclosure is negative.

Sample size	Compounds 14a-14e were subjected to standard PhysChem, DMPK and CRBN binding assays. As such the sample size was determined by the number and diversity of synthesised compounds which provided a good understanding of the reaction scope. Non PROTAC-like molecules synthesised were not included since any data generated would not be meaningful in making statements on PROTAC properties.
Data exclusions	No data was excluded.
Replication	For all data, n = or > 3 unless otherwise noted. All assays are thoroughly validated in-house or by a third party CRO to reliably provide high quality data sufficient for our drug discovery efforts.
Randomization	This is not relevant, as all assays are thoroughly validated in-house or by a third party CRO to reliably provide high quality data sufficient for our drug discovery efforts. While the samples were technically not randomized, they were tested alongside many unrelated samples in high throughput PhysChem-, DMPK, and in vitro pharmacological assays.
Blinding	This is not relevant, as all assays are thoroughly validated in-house or by a third party CRO to reliably provide high quality data sufficient for our drug discovery efforts. While the samples were technically not blinded, they were tested alongside many unrelated samples in high throughput PhysChem-, DMPK, and in vitro pharmacological assays.

Reporting for specific materials, systems and methods

We require information from authors about some types of materials, experimental systems and methods used in many studies. Here, indicate whether each material, system or method listed is relevant to your study. If you are not sure if a list item applies to your research, read the appropriate section before selecting a response.

Materials & experimental systems

n/a	Involved in the study	
[x]	[ ]	Antibodies
[ ]	[x]	Eukaryotic cell lines
[x]	[ ]	Palaeontology and archaeology
[x]	[ ]	Animals and other organisms
[x]	[ ]	Clinical data
[x]	[ ]	Dual use research of concern
[x]	[ ]	Plants

Methods

n/a	Involved in the study	
[x]	[ ]	ChIP-seq
[x]	[ ]	Flow cytometry
[x]	[ ]	MRI-based neuroimaging

Eukaryotic cell lines

Policy information about cell lines and Sex and Gender in Research

Cell line source(s)	Rat Clint: Rat Han Wistar Hepatocytes, supplied by BioIVT.
Authentication	Rat Han Wistar Hepatocytes: Harvested and supplied by BioIVT; not kept in continuous culture. Authentication not relevant.
Mycoplasma contamination	Rat Han Wistar Hepatocytes: Not kept in continuous culture. Mycoplasma testing not relevant.
Commonly misidentified lines (See ICLAC register)	No commonly misidentified cell lines were used.
